# Australian Sphingidae – DNA Barcodes Challenge Current Species Boundaries and Distributions

**DOI:** 10.1371/journal.pone.0101108

**Published:** 2014-07-02

**Authors:** Rodolphe Rougerie, Ian J. Kitching, Jean Haxaire, Scott E. Miller, Axel Hausmann, Paul D. N. Hebert

**Affiliations:** 1 University of Guelph, Biodiversity Institute of Ontario, Guelph, Ontario, Canada; 2 Natural History Museum, Department of Life Sciences, London, United Kingdom; 3 Honorary Attaché, Muséum National d’Histoire Naturelle de Paris, Le Roc, Laplume, France; 4 National Museum of Natural History, Smithsonian Institution, Washington, DC, United States of America; 5 Bavarian State Collection of Zoology, Section Lepidoptera, Munich, Germany; Onderstepoort Veterinary Institute, South Africa

## Abstract

**Main Objective:**

We examine the extent of taxonomic and biogeographical uncertainty in a well-studied group of Australian Lepidoptera, the hawkmoths (Sphingidae).

**Methods:**

We analysed the diversity of Australian sphingids through the comparative analysis of their DNA barcodes, supplemented by morphological re-examinations and sequence information from a nuclear marker in selected cases. The results from the analysis of Australian sphingids were placed in a broader context by including conspecifics and closely related taxa from outside Australia to test taxonomic boundaries.

**Results:**

Our results led to the discovery of six new species in Australia, one case of erroneously synonymized species, and three cases of synonymy. As a result, we establish the occurrence of 75 species of hawkmoths on the continent. The analysis of records from outside Australia also challenges the validity of current taxonomic boundaries in as many as 18 species, including *Agrius convolvuli* (Linnaeus, 1758), a common species that has gained adoption as a model system. Our work has revealed a higher level of endemism than previously recognized. Most (90%) Australian sphingids are endemic to the continent (45%) or to Australia, the Pacific Islands and the Papuan and Wallacean regions (45%). Only seven species (10%) have ranges that extend beyond this major biogeographical boundary toward SE Asia and other regions of the Old World.

**Main Conclusions:**

This study has established that overlooked cryptic diversity and inaccurate species delineation produced significant misconceptions concerning diversity and distribution patterns in a group of insects that is considered well known taxonomically. Because DNA barcoding represents a straightforward way to test taxonomic boundaries, its implementation can improve the accuracy of primary diversity data in biogeography and conservation studies.

## Introduction

*“The crux of the problem is that if we don’t know what is out there or how widely species are distributed, how can we convince people about the reality and form of the biodiversity crisis?”* Riddle et al. [1].

As in many other groups of Australian organisms, the insect fauna of this continent is renowned for its diversity, uniqueness, and many iconic endemics [2]. However, in contrast to plants and vertebrates, the diversity of Australian insects remains poorly documented. There are about 62,000 described species of insects on the continent, representing from 15% to 50% of the total fauna depending on estimates [3]–[5]. The level of species endemism is difficult to evaluate and undoubtedly varies among insect orders [2], but is generally considered to be very high. The overall value may reach 90% [3], and many families include only endemics [2]. However, much effort is still needed to fill gaps in taxonomy and to extend understanding of taxon distributions, deficits that have come to be termed respectively the Linnean and Wallacean shortfalls [6].

The integration of molecular methods into taxonomic practice has improved our capacity to understand and describe diversity [7], [8] and has been proposed as a way to help increase the pace of species discovery and description [9], [10]. Although the application of integrative approaches to the Australian fauna is still in its infancy, studies have already revealed spectacular levels of overlooked and cryptic species in some groups of vertebrates [11], [12]. Similar cases have also been reported in invertebrates, typically revealing species that raise the already high level of endemism in Australia [13]–[15]. Particularly comprehensive efforts have been directed toward Australian Lepidoptera. Much of this information is available in an indexed public reference library of DNA barcode sequences [16], [17] (database accessible in BOLD at www.boldsystems.org) and via the current taxonomic system for the complete Australian fauna [18] (see also http://www.lepbarcoding.org/australia).

The order Lepidoptera includes about 130 families whose diversity varies dramatically – from a single to more than 24,000 species [19]. Because the current count of approximately 160,000 described species is thought to reflect less than one-third of the total [20], diversity estimates for some families, especially smaller-bodied taxa, are very uncertain. However, other families, such as the Sphingidae, have attracted so much taxonomic interest that present estimates of diversity are thought to be comprehensive except for ‘frontier’ regions. The last global conspectus on the Sphingidae recognized 1278 species [21], but this count has now risen by about 200 species [19] with most new taxa deriving from hyperdiverse tropical faunas. In Australia, Europe and North America, the pace of species discovery has slowed, suggesting that species counts are complete or nearly so.

### The Sphingidae of Australia

Australia provides a good example of a continental fauna of sphingids with a mature taxonomy. Collection programs have been wide ranging, morphological studies have been careful, and species discovery has slowed: 53 species were described by the end of the 19^th^ century, 62 by 1927, but only two more taxa were added during the next 70 years [22]. Although many families of Australian Lepidoptera are dominated by endemic species [2], the sphingid fauna is thought to include a mix of endemics and species with distributions extending into the Pacific islands, Southeast Asia and even into Europe and Africa.

The descriptions of some wide-ranging species in the Australian fauna date back to the launch of Linnaean nomenclature (e.g. *Agrius convolvuli* (Linnaeus, 1758) and *Hippotion celerio* (Linnaeus, 1758)). Other species were described only slightly later from Asia (e.g. *Theretra nessus* (Drury, 1773) and *Nephele hespera* (Fabricius, 1775), both described from India). The first sphingids to be described from Australia were the endemics *Coequosa australasiae* (Donovan, 1805), *Coequosa triangularis* (Donovan, 1805) and *Cizara ardeniae* (Lewin, 1805). Moulds [22] provided an historical overview of work on Australian sphingids, listing 64 species in 22 genera for the continent. Although only two species (*Imber tropicus* (Moulds, 1983) and *Psilogramma argos* Moulds & Lane, 1999) were described from 1930–2000, eleven species and one subspecies have been described since then, including seven species of *Psilogramma*, a complex genus whose species count exploded globally from four to more than 60 over the past decade [21], [23]–[25], although perhaps only about half of these are actually valid species (I.J. Kitching, unpublished data). Three new genera, *Imber* Moulds *et al.*, 2010, *Pseudoangonyx* Eitschberger, 2011, and *Cerberonoton* Zolotuhin & Ryabov, 2012 were recently erected for rather distinctive species, with a fourth new genus being established for a newly encountered species (*Zacria vojtechi* Haxaire & Melichar, 2003) demonstrating that striking discoveries can still occur in this fauna. Recent nomenclatural changes within the genus *Macroglossum* also affected the names of two Australian species [26]. In his checklist of Australian Sphingidae, Moulds [22] omitted subspecies names, concealing the distinctiveness of the Australian fauna from those of Neighbouring Southeast Asia and Pacifica. In actuality, many Australian sphingids have been assigned to an endemic subspecies because of their morphological divergence from the nominotypical subspecies. At least some of these cases may reflect cases of long separation that merit recognition as distinct species, but their status has not yet been investigated in detail.

The delineation of species/subspecies and the existence of cryptic or overlooked undescribed species can be a serious impediment for biogeography and conservation studies [6]. This “Linnean shortfall” represents a formidable challenge for research in these disciplines [27]. Building on recent access to molecular tools for species identification and discrimination [28], [29] and the success of integrative approaches in taxonomy [30]–[32], the present study employs sequence diversity in the DNA barcode region of the mitochondrial gene COI to investigate the diversity of Australian sphingids and their distributions. We begin by examining patterns of sequence diversity within the Australian fauna to ascertain the efficacy of DNA barcodes to discriminate known species and reveal overlooked diversity. We then place these results in a broader geographic context by examining patterns of sequence divergence between Australian populations and their conspecific lineages outside the continent.

## Material and Methods

### Specimen Sampling

More than 1200 Australian sphingids were sampled, most from three collections – the Biodiversity Institute of Ontario (361), the Zoologische Staatssammlung München (300), and the Australian National Insect Collection (112). The remaining specimens derive from 17 other collections (see details in Table S1). All institutions and collections granted us permission to access and study the material used in this work. Sampling aimed to maximize the geographic coverage for each species or subspecies to examine the extent of barcode variation across its distribution. In the most common species, a large number of specimens were sampled (more than 30 for 11 species, 113 for *Agrius convolvuli*), but fewer than five specimens were examined for 20 species/subspecies that are rare. Each record was given a unique specimen identifier (SampleID) and sequence identifier (ProcessID) to provide a direct link between voucher specimens and DNA barcode records. Collection data, a photograph and ancillary information, such as the collection holding each specimen, were compiled in BOLD for each individual record.

### DNA Sequencing

The methods used for generation of DNA sequences follow standard protocols designed for amplification and sequencing of the standard DNA barcode. In four species, the same DNA extracts were also used for the sequencing of the D2 expansion segment of the 28S rDNA gene. Details of these methods are given as Supporting Information (Appendix S1).

### Data Analysis

A nearest Neighbour analysis was conducted with BOLD using either a dataset restricted to sphingid records from Australia or the full database for world sphingids, including all COI barcode sequences longer than 500 bp. The latter database includes about 23 K sequences, 1.8 K species and subspecies (as of September 12^th^, 2013). BOLD was also used for sequence alignment and calculation of genetic divergences and Neighbour Joining (NJ) trees using a K2P distance model [33] after alignment. These trees were imported in iTOL [34] to exploit its capacity for the visualization of large trees. In two cases where DNA barcode analysis suggested potential cases of overlooked diversity, we also generated “geo-phylogenies” using GenGIS 2.2.0 [35] to better visualize the geographical structure of genetic variation. MEGA 5.2.2 [36] was also used to produce character-based trees using the maximum parsimony (MP) criterion (all settings at default, with the complete deletion option for missing data) in cases of species-complexes where genetic distances are shallow and require a more rigorous method for reconstructing relationships between terminals. The topology of these DNA barcode “gene trees” was compared with the “species tree” inferred from current taxonomy based on morphology. Attention was directed toward an examination of the incidence of reciprocal monophyly rather than relationships between species. The division of a species into two or more clusters of specimens was considered a potential indicator of cryptic diversity (candidate species) when the species included two or more monophyletic clusters with more than 2% divergence [37], [38] in their DNA barcode sequences. This threshold was used as an operational criterion to trigger in-depth study (comparative morphology, sequencing of a nuclear marker), but cases of shallower divergences are also reported and discussed when the genetic clusters match existing or previously proposed taxonomic divisions (at species or subspecies level). Candidate species were considered as Confirmed Candidate Species (CCS) when independent evidence (e.g. morphology, nuclear gene) also supported their distinction, or as Deep Conspecific Lineages (DCL) when no morphological differences or divergence at 28S rDNA were detected [39], [40]. We note, however, that there is no objective way to confirm the status of any geographically isolated CCS as distinct species (or subspecies). As pointed out by Mutanen et al. [38], taxonomists working on Lepidoptera when dealing with closely related but allopatric populations have traditionally assigned them to different subspecies when slight morphological differences are apparent (usually in wing patterns), but to distinct species when morphological differences are greater (usually both in wing patterns and genitalia). The consideration of genetic differences as a basis for species delineation in Lepidoptera is recent. As a result there is no established practice regarding the taxonomic treatment of units currently diagnosed only through DNA data, whether their divisions are derived from the analyses of a single or multiple DNA markers. Our strategy has involved close collaboration with taxonomists studying Australian hawkmoths, highlighting all potential or confirmed candidate species for further morphological, ecological and biogeographical investigation.

### Data Accessibility (BOLD, GenBank)

Data on specimen vouchers, sequence data including trace files and registered primer pairs are available on BOLD within the datasets SPH01AUS (dx.doi.org/10.5883/DATASET-SPH01AUS) for Australian records, and SPH02AUS (dx.doi.org/10.5883/DS-SPH02AUS) for conspecifics and most closely related taxa outside Australia. All sequences were also deposited in GenBank for COI DNA barcodes and for 28S rDNA sequences (see Tables S1–S3).

## Results

### Diversity of Australian Sphingids

**Figure 1:**
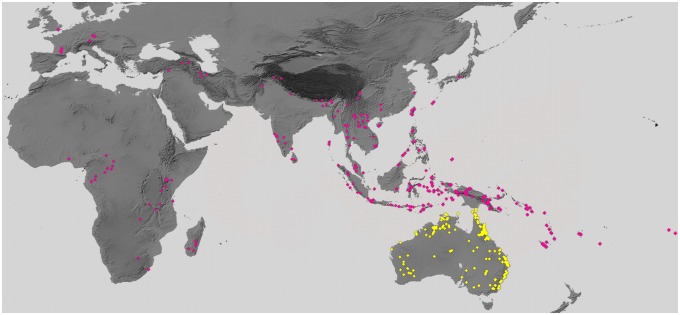
Geographical sampling. Distribution of the 1054 specimens of Australian Sphingidae with DNA barcodes analysed in this study (yellow) and of the 735 additional specimens of conspecifics (including hetero-subspecific taxa) from outside Australia and relevant closely related species with DNA barcodes (see details of record lists in Table S1–S3).

In total, DNA barcodes were obtained from 1054 specimens representing 70 of the 72 valid sphingid species currently recognized from Australia (Table 1). Belonging to 25 genera, these specimens were sampled from across the continent with the majority from Queensland (56%) and New South Wales (24%), where diversity is the highest and collecting efforts have been greatest (Fig. 1). This total includes four species (*Psilogramma exigua*, *P. penumbra*, *Hopliocnema ochra*, *H. lacunosa*) that were recently described on the basis of their DNA barcode divergence and correlated morphological differences [23], [41], [42]. The genus *Psilogramma* requires a global taxonomic and nomenclatural revision [41]. Nevertheless, but for the possible exceptions of *P. menephron* and *P. papuensis*, the nomenclature of the seven species found in Australia should remain stable if considering the three cases of synonymy revealed here by the analysis of the relevant type specimens (*P. hausmanni* syn. nov., *P. gloriosa* syn. nov., *P. koalae* syn. nov.; see Table 1, footnotes 4–6).

**Table 1:** Sampling coverage and genetic distances for the Sphingidae of Australia.

#	Species	Type Locality	N	Australia	EM	Pi	WM	SEA	OW	δA	dA	sd	δG	dG	sd
**1**	*Acosmeryx anceus* (Stoll, 1781)	Indo.: Maluku	59	47 [QLD,NSW]	12	-	-	-	-	**6.41**	2.86	0	**6.61**	2.86	0
**2**	*Acosmeryx miskini* (Murray, 1873)	Austr.: QLD	13	13 [QLD,NSW]	-	-	-	-	-	0.93	0.35	0	N/A	N/A	N/A
**3**	*Angonyx papuana* Rothschild & Jordan, 1903	Austr.: QLD	11	4 [QLD]	7	-	-	-	-	0.15	0.03	0.01	1.08	0.38	0
**4**	*Cephonodes hylas australis* Kitching & Cadiou, 2000	Austr.: QLD	4	4 [QLD,NT]	-	-	-	-	-	0.51	0.25	0.03	N/A	N/A	N/A
**5**	*Cephonodes janus* Miskin, 1891	Austr.: QLD	2	2 [QLD]	-	-	-	-	-	0.17	N/A	N/A	N/A	N/A	N/A
**6**	*Cephonodes kingii* (W.S. Macleay, 1826)	Austr.	6	6 [QLD]	-	-	-	-	-	0.33	0.18	0.01	N/A	N/A	N/A
**7**	*Cephonodes picus* (Cramer, 1777)	India	11	5 [QLD]	1	0	3	2	-	1.39	0.57	0.05	*3.59*	1.37	0.02
**8**	*Cizara ardeniae* (Lewin, 1805)	Austr.: NSW	4	4 [QLD]	-	-	-	-	-	0.85	0.4	0.04	N/A	N/A	N/A
**9**	*Daphnis dohertyi* Rothschild, 1897	Indo.: Papua Barat	4	1 [QLD]	3	-	-	-	-	N/A	N/A	N/A	0.38	0.29	0.02
**10**	*Daphnis moorei* (Macleay, 1866)	Austr.: QLD	16	10 [QLD,WA]	5	1	-	-	-	0.46	0.18	0	1.03	0.27	0
**11**	*Daphnis placida* (Walker, 1856)	Indo.: Sumatra	25	12 [QLD,NSW]	5	1	4	3	-	0.77	0.22	0	*2.13*	0.68	0
**12**	*Daphnis protrudens* Felder, C. & Felder R., 1874	Indo.: Maluku	6	3 [QLD]	3	-	-	-	-	0	0	0	0.61	0.26	0.01
**13**	*Eupanacra splendens* (Rothschild, 1894)	Austr.: QLD	27	15 [QLD]	10	2	-	-	-	*2.19*	0.97	0.01	**6.87**	2.17	0
**14**	*Gnathothlibus australiensis* Lachlan, 2004	Austr.: QLD	5	5 [QLD]	-	-	-	-	-	0.61	0.34	0.02	N/A	N/A	N/A
**15**	*Gnathothlibus eras* (Boisduval, 1832)	French Polynesia	39	30 [QLD,NT]	3	6	-	-	-	0.92	0.28	0	*3.47*	0.58	0
**16**	*Hippotion brennus* (Stoll, 1782)	Indo.: Maluku	38	8 [QLD,NSW]	30	-	-	-	-	1.39	0.9	0.02	**3.96**	1.93	0
**17**	*Hippotion celerio* (Linnaeus, 1758)	?	92	42 [QLD,CT,NSW,WA]	7	1	5	0	37	1.09	0.07	0	1.58	0.34	0
**18**	*Hippotion rosetta* (Swinhoe, 1892)	Indo.: Maluku	32	27 [QLD]	2	-	2	1	-	0.77	0.30	0	1	0.35	0
**19**	*Hippotion scrofa* (Boisduval, 1832)	Austr.	66	63 [QLD,NSW,CT,WA,NT]	-	3	-	-	-	0.68	0.08	0	1.49	0.16	0
**20**	*Hippotion velox* (Fabricius, 1793)	“India orientali”	24	7 [QLD]	10	2	1	4	-	0.31	0,09	0	1.55	0.22	0
**21**	*Hyles livornicoides* (Lucas, 1892)	Austr.: QLD	39	39 [QLD,NSW,WA]	-	-	-	-	-	0.66	0.21	0	N/A	N/A	N/A
**22**	*Macroglossum alcedo* Boisduval, 1832	Irian Jaya	2	2 [QLD]	0	-	-	-	-	0.48	N/A	N/A	N/A	N/A	N/A
**23**	*Macroglossum corythus* Walker, 1856	Sri Lanka	34	1 [QLD]	15	6	3	9	-	N/A	N/A	N/A	*3.48*	1.66	0
**24**	*Macroglossum dohertyi doddi* Clark, 1922	Austr.: QLD	7	5 [QLD]	2	-	-	-	-	0.33	0.13	0,01	0.33	0.13	0
**25**	*Macroglossum divergens queenslandi* Clark, 1927^1^	Austr.: QLD	-	0	-	-	-	-	-	-	-	-	N/A	N/A	N/A
**26**	*Macroglossum hirundo errans* Walker, 1856	Austr.	13	11 [Q.NSW]	0	2	-	-	-	1.03	0.33	0	1.37	0.53	0
**27**	*Macroglossum joannisi* Rothschild & Jordan, 1903	Austr.: QLD	1	1 [QLD]	-	-	-	-	-	N/A	N/A	N/A	N/A	N/A	N/A
**28**	*Macroglossum micacea* Walker, 1856	Austr.: QLD	6	6 [QLD,NSW]	0	-	-	-	-	0.86	0.48	0.02	N/A	N/A	N/A
**29**	*Macroglossum nubilum* Rothschild & Jordan, 1903	PNG: Milne Bay	4	3 [QLD]	1	-	-	-	-	0.21	0.13	0.03	1.04	0.38	0.05
**30**	*Macroglossum prometheus lineata* Lucas, 1891	Austr.: QLD	8	4 [QLD]	4	-	-	-	-	0.48	0.34	0.02	1.34	0.7	0.01
**31**	*Macroglossum rectans* Rothschild & Jordan, 1903	Indo.: Maluku	9	5 [QLD]	4	-	-	-	-	1.04	0.5	0.03	1.04	0.53	0,01
**32**	*Macroglossum tenebrosa* Lucas, 1891	Austr.: QLD	10	4 [QLD]	6	-	-	-	-	0.46	0.23	0.04	*2.98*	1.17	0.02
**33**	*Macroglossum troglodytus* Boisduval, [1875]^2^	India: Darjeeling	1	1 [QLD]	-	-	-	-	-	N/A	N/A	N/A	N/A	N/A	N/A
**34**	*Macroglossum vacillans* Walker, 1865	“Timor”	22	13 [QLD,NT,WA]	9	-		-	-	0.83	0.27	0	1.24	0.36	0
**35**	*Nephele hespera* (Fabricius, 1775)	“India orientali”	11	1 [QLD]	-	-	-	10	-	N/A	N/A	N/A	*2.32*	0.48	0.01
**36**	*Nephele subvaria* (Walker, 1856)	Austr.	15	15 [QLD,WA,NT]	-	0	-	-	-	0.26	0.042	0	N/A	N/A	N/A
**37**	*Pseudoangonyx excellens* (Rothschild, 1911)	Indo.: Papua	4	2 [QLD]	2	-	-	-	-	0	N/A	N/A	0.53	0.4	0.03
**38**	*Theretra celata* (Butler, 1877)	Austr.: QLD	25	17 [QLD, NSW]	8	-	-	-	-	0.59	0.14	0	0.79	0.16	0
**39**	*Theretra indistincta* (Butler, 1877)	Austr.: QLD	12	12 [QLD, NT]	-	-	-	-	-	0.36	0.06	0	N/A	N/A	N/A
**40**	*Theretra inornata* (Walker, 1865)	“N. Austr.”	12	12 [QLD, NT]	-	-	-	-	-	1.24	0.47	0.01	N/A	N/A	N/A
**41**	*Theretra latreillii* (W.S. Macleay, 1826)	Austr.	58	56 [QLD,NSW,WA]	2	-	-	-	-	0.47	0.025	0	0.62	0.04	0
**42**	*Theretra margarita* (Kirby, 1877)	Austr.: QLD	30	30 [QLD,WA,NT]	-	-	-	-	-	0.89	0.22	0	N/A	N/A	N/A
**43**	*Theretra nessus* (Drury, 1773)	India: Tamil Nadu	31	16 [QLD,NSW]	6	1	0	8	-	0.37	0.15	0	**4.97**	2,2	0
**44**	*Theretra oldenlandiae lewini* (Thon, 1828)	Austr.: NSW	82	81 [QLD,NSW,NT,WA]	1	-	-	-	-	0.86	0.24	0	0.86	0.24	0
**45**	*Theretra queenslandi* (Lucas, 1891)	Austr.: QLD	15	15 [QLD,NSW]	-	-	-	-	-	0.18	0.02	0	N/A	N/A	N/A
**46**	*Theretra radiosa* Rothschild & Jordan, 1916	PNG	4	0	4	-	-	-	-	-	-	-	*4.42*	2.73	0.32
**47**	*Theretra silhetensis intersecta* (Butler, [1876])	Austr.: QLD	24	20 [QLD,NT,WA]	0	2	2	-	-	0.31	0.03	0	0.31	0.05	0
**48**	*Theretra tryoni* (Miskin, 1891)	Austr.: QLD	10	5 [QLD,NSW]	5	-	-	-	-	0.31	0.093	0.01	0.84	0.44	0.01
**49**	*Theretra turneri* (Lucas, 1891)	Austr.: QLD	7	7 [QLD]	-	-	-	-	-	0.63	0.22	0.01	N/A	N/A	N/A
**50**	*Zacria vojtechi* Haxaire & Melichar 2003	Austr.: WA	4	4 [WA]	-	-	-	-	-	0.17	0.06	0.01	N/A	N/A	N/A
**51**	*Ambulyx dohertyi queenslandi* Clark, 1928	Austr.: QLD	11	4 [QLD]	7	-	-	-	-	0.15	0.02	0.01	0.82	0.31	0
**52**	*Ambulyx wildei* Miskin, 1891	Austr.: QLD	20	11 [QLD]	9	-	-	-	-	0	0	0	0.78	0.28	0
**53**	*Coequosa australasiae* (Donovan, 1805)	Austr.	8	8 [QLD,NSW,NT]	-	-	-	-	-	1.17	0.62	0.01	N/A	N/A	N/A
**54**	*Coequosa triangularis* (Donovan, 1805)	Austr.	5	5 [QLD,NSW]	-	-	-	-	-	0.5	0.32	0.02	N/A	N/A	N/A
**55**	*Imber tropicus* Moulds, 1983	Austr.: NT	7	7 [QLD,WA]	-	-	-	-	-	*2.02*	1.21	0.03	N/A	N/A	N/A
**56**	*Agrius convolvuli* (Linnaeus, 1758)	?	174	113 [QLD,CT,NSW,WA,SA,NT]	4	3	0	14	40	0.52	0.06	0	**5.37**	1.79	0
**57**	*Agrius godarti* (W.S. Macleay, 1826)	Austr.	16	16 [QLD,NSW]	-	-	-	-	-	0.98	0.23	0	N/A	N/A	N/A
**58**	*Cerberonoton rubescens severina* (Miskin, 1891)	Austr.: QLD	8	6 [QLD]	2	-	-	-	-	0.17	0.05	0	1.08	0.39	0.01
**59**	*Coenotes eremophilae* (Lucas, 1891)^3^	Austr.: QLD	17	17 [QLD,WA,NT]	-	-	-	-	-	1.28	0.31	0	N/A	N/A	N/A
**60**	*Hopliocnema brachycera* (Lower, 1897)	Austr.: NSW	13	13 [NT,WA,NSW,SA,VIC]	-	-	-	-	-	0.5	0.15	0	N/A	N/A	N/A
**61**	*Hopliocnema lacunosa* Tuttle et al. 2012	Austr.: WA	5	5 [WA]	-	-	-	-	-	0.93	0.4	0.03	N/A	N/A	N/A
**62**	*Hopliocnema ochra* Tuttle et al. 2012	Austr.: WA	6	6 [WA]	-	-	-	-	-	0.93	0.54	0.01	N/A	N/A	N/A
**63**	*Leucomonia bethia* (Kirby, 1877)	Austr.: QLD	6	6 [QLD,WA,NT]	-	-	-	-	-	0.31	0.16	0.01	N/A	N/A	N/A
**64**	*Psilogramma argos* Moulds & Lane, 1999	Austr.: QLD	3	3 [NT]	-	-	-	-	-	0.61	0.41	0.1	N/A	N/A	N/A
**65**	*Psilogramma casuarinae* (Walker, 1856)^4^	Austr.: NSW	32	32 [QLD,NSW,VIC]	-	-	-	-	-	1.24	0.43	0	N/A	N/A	N/A
**66**	*Psilogramma exigua* Brechlin et al. 2010	Austr.: WA	16	16 [QLD,WA,NT]	-	-	-	-	-	0.46	0.13	0	N/A	N/A	N/A
**67**	*Psilogramma maxmouldsi* Eitschberger, 2001	Austr.: QLD	9	9 [QLD,NSW]	-	-	-	-	-	0.31	0.15	0	N/A	N/A	N/A
**68**	*Psilogramma menephron* (Cramer, 1780)^5^	Indo.: Maluku	114	34 [QLD,NSW]	30	-	21	29	-	0.78	0.2	0	*8.25*	3.97	0
**69**	*Psilogramma papuensis* Brechlin, 2001^6^	PNG: Western	37	25 [QLD]	12	-	-	-	-	0.35	0.11	0	1.17	0.34	0
**70**	*Psilogramma penumbra* Lane et al. 2011	Austr.: NT	4	4 [NT]	-		-	-	-	0	0	0	N/A	N/A	N/A
**71**	*Synoecha marmorata* (Lucas, 1891)^3^	Austr.: QLD	14	14 [QLD,NSW,NT]	-	-	-	-	-	**9,74**	2.72	0.04	N/A	N/A	N/A
**72**	*Tetrachroa edwardsi* (Olliff, 1890)	Austr.: QLD/NSW	4	4 [QLD]	-	-	-	-	-	0	0	0	N/A	N/A	N/A

**Figure 2:**
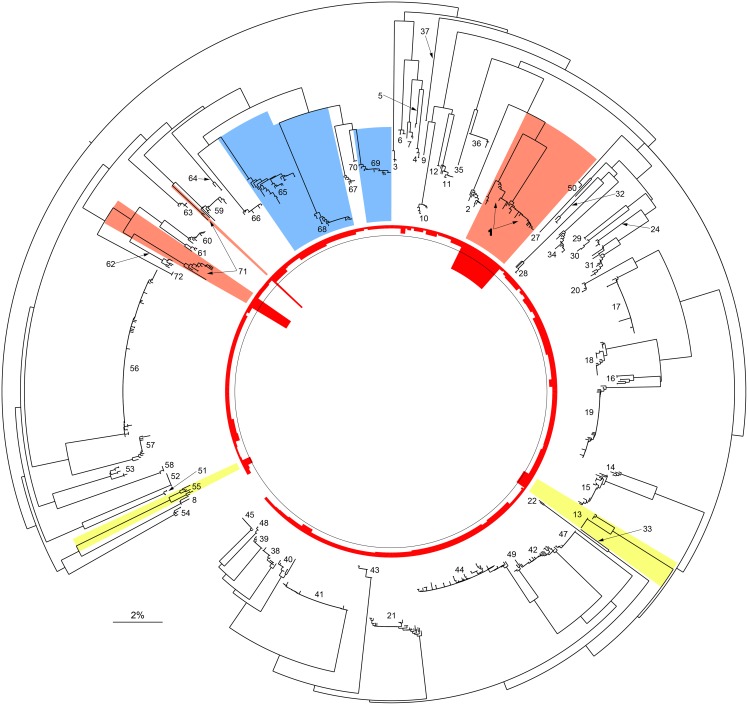
DNA barcode variation in Australian Sphingidae. Neighbour Joining tree based on K2P distances for 1054(ranging from 0 to 9.7%, the circle line mark the 2% threshold); colour ranges highlight cases of synonymy (in blue), cryptic diversity (CCS, in red) as well as these species with 2% or more intraspecific distance (DCL, yellow) but not proven to represent two different species. Numbers refer to species numbers as listed in Table 1. An interactive and fully explorable version of the tree is available at http://itol.embl.de/shared/rodroug. (See also Fig. S1.).

**Figure 3:**
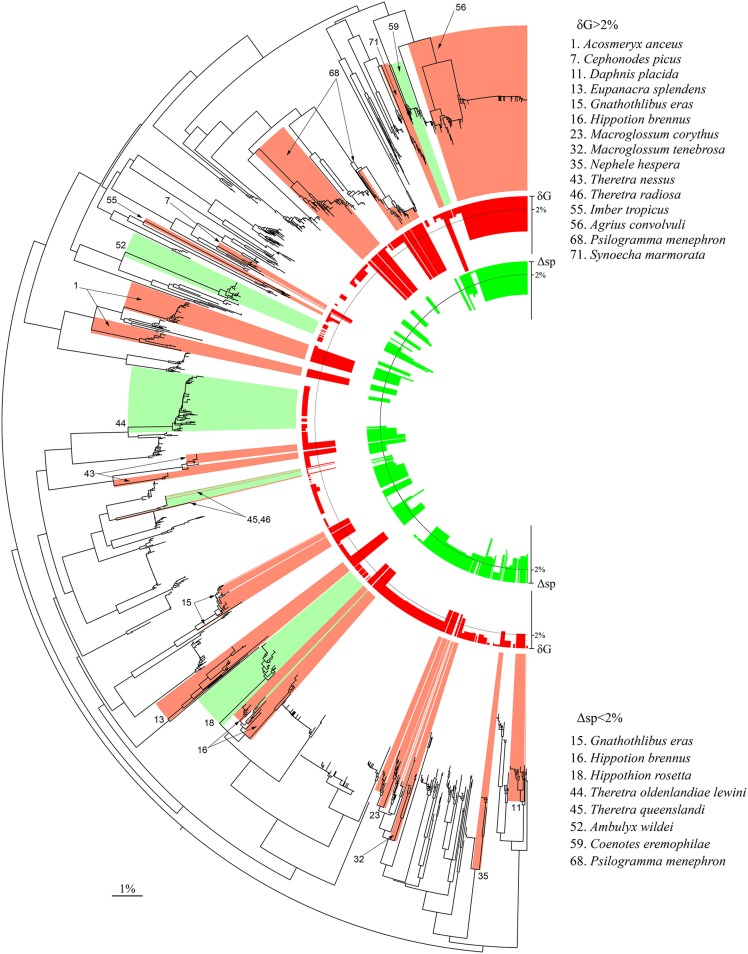
DNA barcode variation in the geographically extended dataset. Neighbour Joining tree based on K2P distances for the 1054 Australian sphingid records analysed in Fig. 2 augmented by 735 records for conspecifics, co-subspecifics and closely related species from outside Australia. In the centre of the tree, the inner (red) and outer (green) histograms represent the maximum intraspecific distance (δG) and the distance to the nearest heterospecific Neighbour on BOLD (Δsp). The species with δG>2% and Δsp<2% are highlighted in the tree with red and green colour ranges, respectively; those species are also listed with numbers following species numbers in Table 1. An interactive and fully explorable and searchable version of this tree can be accessed at http://itol.embl.de/shared/rodroug. (See also Fig. S5.).

Taxonomic checklist, type localities, sample size (N), geographical origin and DNA barcode variations for analysed samples; underlined names highlight taxa with no DNA barcode available from Australia. CT = Capital Territory, EM = Eastern Malesia (Wallacea + Papuan region), NSW = New South Wales, NT = Northern Territory, OW = other regions of the world, Pi. = Pacific islands, QLD = Queensland, SA = South Australia, SEA = continental Southeast Asia, VIC = Victoria, WA = Western Australia, WM = Malesia West of the Wallacean region; δA, dA, δG and dG = maximum (δ) and mean (d) intraspecific K2P distance (%) within Australia (A) and globally (G); sd = standard deviation; numbers in the first column match numbered clusters in Figs 2 and 3. The δ distances over 2% are highlighted in bold characters in cases involving at least one Confirmed Candidate Species (CCS), and in italics in cases where there is no complementary evidence available (Deep Conspecific Lineages, DCL). Species are given a null sample size in areas where they are known to occur but from where no sample was analyzed whereas empty cells mark areas where the species are considered absent in our current state of knowledge. Taxa are sorted by subfamily as follow: 1–50 = Macroglossinae, 51–55 = Smerinthinae, 56–72 = Sphinginae.

1 This species is only known to us from specimens bred or collected in the 1910’s. Previously referred as *M. heliophila queenslandi*. See Holloway (2011)

2 Previously referred as *M. insipida* Butler, 1875. See Holloway (2011)

3 A new species, *Coenotes arida Moulds & Melichar*, [2014], was described during the review of this study and does not appear in this table. Its original generic and specific identifications as *Synochea marmorata* were erroneous, and the affinity of these specimens with the genus *Coenotes* has been confirmed by morphology.

4 *P. hausmanni* Eitschberger, 2001 syn. nov. (holotype sequenced) is here confirmed a synonym of *P. casuarinae*. It is neither morphologically nor genetically distinct from this species.

5 *P. gloriosa* Eitschberger, 2001 syn. nov. (holotype sequenced) is here considered a synonym of *P. menephron*. It is neither morphologically nor genetically distinct. *P. menephron* represents a vast species complex in Asia and the Indo-Australian region, and a revision is needed to sort out the divisions within this complex (e.g. DNA barcodes reveal nine distinct clusters).

6 *P. koalae* Eitschberger, 2001 syn. nov. (holotype sequenced) is here considered a synonym of *P. papuensis* (holotype sequenced). It is neither morphologically nor genetically distinct from this species.

NJ analysis of the barcode sequences indicated that representatives of each of the 70 species formed a cohesive and reciprocally monophyletic cluster (Fig. 2; see Fig. S1 for details). The mean within-genus divergence (7.13%, sd. = 0.007%) is considerably higher than the mean within-species distance (0.3%, sd. = 0.007%), matching the pattern of variation already reported in other families of Lepidoptera [43]. The lowest minimum interspecific distance was 1.19% between *Macroglossum rectans* and *M. hirundo errans,* but the clusters for these species were still separate, and both taxa possessed a maximum intraspecific divergence of 1.0%. The next closest pair, *Hippotion celerio* and *H. velox,* had 2.7% divergence, while *Coequosa australasiae* and *C. triangularis* had the greatest nearest-Neighbour divergence (7.2%). Intraspecific variation among the Australian samples (Fig. 2 and Table 1) was generally low, even in some of the most heavily sampled species such as *Agrius convolvuli* (Dmax = 0.5%, N = 113) or *Theretra latreillii* (Dmax = 0.5%, N = 56). However, intraspecific divergence did exceed 2.0% in four species (Table 1): *Imber tropicus* (2%), *Eupanacra splendens* (2.2%), *Acosmeryx anceus* (6.4%), and *Synoecha marmorata* (9.7%) with each taxon represented by two clusters (Fig. 2, Fig. S1). The first two cases involve intraspecific distances of approximately 2%, and we treat them as cases of DCL (Fig. 2, highlighted in pale yellow), a conclusion supported by their lack of morphological divergence and the absence in *E. splendens* of congruent variation in 28S rRNA sequences (Fig. S2). In the other two cases, intraspecific divergence was substantially higher (Fig. 2, highlighted in red), and other lines of evidence support their status as CCS. Morphological investigation of the split in *S. marmorata* [44] revealed that one of its lineages represents an undescribed species (formally described as *Coenotes* Moulds & Melichar, [2014] during the review of this study). The original generic and specific identifications were erroneous, and the affinity of these specimens with the genus *Coenotes* is confirmed by morphology and is supported by the DNA barcode data (representatives of the new taxon show just 3.3% minimum genetic distance from *C. eremophilae* (Fig. S1)). The fourth species, *A. anceus,* includes two lineages with 6.4% divergence, one broadly distributed in eastern Australia, the other restricted to northern Queensland. Interestingly, this split matches a tentative separation already proposed in the 1970s by taxonomists Alan Hayes and Jean-Marie Cadiou (I.J. Kitching, personal communication) and materialized through specimens sorted accordingly in collections at the Natural History Museum, London. Re-examination of series of specimens and rearing revealed several morphological characters (differences in size and wing colouration, as well as larval and pupal morphology (J.P. Tuttle & M.S. Moulds, personal communication)) and there is also one diagnostic nucleotide substitution between the taxa at position 550 in the analysed segment of 28S rRNA (Fig. S2). Furthermore, while the two DNA barcode clusters of *A. anceus* appear sympatric in northern Queensland (Fig. S3), they have micro-allopatric distributions; one cluster is restricted to sclerophyll forests along the east coast of Australia, and the other has only been collected in rain forests in Eastern Cape York Peninsula and Papua New Guinea. Lastly, a shallower split (1.4%) among Australian records of *Hippotion brennus* suggests the validity (in agreement with similar suggestions by Darge [45] and Riotte [46]) of the synonymized taxon, *johanna* (Kirby, 1877), because the three sequenced specimens with a typical *johanna-*like morphology form a distinct DNA barcode group (Figs S1 and S4) that is also separated geographically. Overall, considering this last and the two additional new species in the genera *Acosmeryx* and *Coenotes*, these results bring the total of Australian sphingid species to 75.

### A Global Perspective on Australian Sphingids

Of the 75 species of sphingids that occur in Australia, 30 of them (40%) are endemic to the continent (Table S4). Another 45 species then occur in both Australia and another geographic region, of which four are represented by an endemic subspecies in Australia (the nominal subspecies in two cases). This brings to 34 (45%) the number of endemic sphingid taxa in Australia, at species or subspecies level. For all of the 45 species distributed in Australia and outside the continent, we examined the patterns of DNA barcode variation after including specimens from other regions. This involved the combined analysis of sequences from 1054 Australian specimens, 599 conspecifics from outside Australia, and 136 records for closely related species from other regions (Table 1 and Tables S1–S3). The 599 conspecific records include 112 con-subspecific records and 140 hetero-subspecific records for 18 polytypic Australian species, and 347 records for 26 monotypic Australian species or species with no available record for distinct subspecies. The results of the analysis of this combined dataset are given in Fig. 3 (see Fig. S5 for details).

This analysis revealed an additional very deep split in *Eupanacra splendens* and ten more cases of deep (>2%) intraspecific divergences involving *Agrius convolvuli, Cephonodes picus, Daphnis placida, Gnathothlibus eras, Hippotion brennus, Macroglossum corythus, M. tenebrosa, Nephele hespera, Psilogramma menephron* and *Theretra nessus* (Table 1, Fig. 3). The two DNA barcode clusters in *Agrius convolvuli* show deep divergence (Dmax = 5.37%), and clear geographical segregation (Fig. S6). Preliminary comparisons of wing patterns and male genitalia have been inconclusive and further morphological study is required, but the two lineages show three diagnostic substitutions (positions 317, 486 and 505) in sequences for the 28S rDNA gene (Fig. S2), supporting their status as different species. Three other species with deep splits (*E. splendens* (the West New Britain record only), *T. nessus, H. brennus*) showed morphological differences between representatives of the divergent DNA barcode clusters (e.g. Fig. S4), suggesting that they too represent cases of overlooked diversity. Other cases also merit deeper investigation. For example, the observed genetic divergences in *C. picus* challenge the proposed synonymy of *C. cunninghami* (Walker, 1856) [21] described from Australia. In this case, as well as three others (*G. eras, D. placida, N. hespera*), the genetic splits are associated with geographical distances of several thousand kilometres across major biogeographical boundaries. In *M. tenebrosa,* DNA barcodes reveal the genetic distinctness of the representatives from Sulawesi (Fig. S5); morphological comparisons are yet to be conducted, and no variation was observed in the few available sequences of the D2 fragment of the 28S rDNA gene (Fig. S2). Finally, both *M. corythus* and *P. menephron* represent species complexes requiring a comprehensive revision to establish the validity of the many available names; there are nine valid subspecies names [21] for the former, while we found that the sequenced holotypes of 10 species within genus *Psilogramma* fall within our set of DNA barcode clusters forming the *P. menephron* complex (Fig. S5).

In contrast to these cases of deep sequence divergence within species, the extended dataset revealed eight cases where Australian taxa showed less than 2% divergence (Table S5) from non-Australian specimens assigned to a different species (Fig. 3, Fig. S5, green boxes). Further analysis is required to evaluate the significance of these cases of similarity in DNA barcodes despite differing taxonomic assignments. Such cases can have several origins: identification errors, overlooked synonymy, incomplete lineage sorting or mitochondrial introgression through hybridization [47], or mitogenome replacement induced by *Wolbachia* infections [48]. Identification errors could not be ruled out to explain our results in the pair *Hippotion rosetta*/*H. boerhaviae,* two species long treated as one and often indistinguishable without genitalia dissection, and in the triplet *Gnathothlibus eras/saccoi/vanuatuensis*, which are difficult to distinguish from wing morphology. Cases of overlooked synonymy account for many of the taxa in the *Psilogramma menephron* complex, and probably also the triplet *Ambulyx wildei*/*ceramensis*/*rudloffi* (holotype sequenced) and possibly the pair *Coenotes eremophilae*/*jakli* (paratype sequenced). Finally, hybridization and associated introgression, or mitogenome replacement, may account for the barcode similarity between the morphologically very distinctive species pairs *Hippotion brennus/joiceyi* (Fig. S4) and *Theretra oldenlandiae/insignis* (Fig. S7). In certain cases such as the triplet *Theretra radiosa*/*queenslandi*/*muricolour*, barcode similarity may reflect the impact of two or more of these causal agents.

These analyses also indicated that Australian subspecies are reciprocally monophyletic for 16 of the 18 species examined (Table S5 and Fig. S5). In ten of these cases, the Australian subspecies diverges by more than 2% from its closest conspecific subspecies with the most distant pair being *Eupanacra splendens splendens/paradoxa* with a minimum pairwise distance of 5.96%. We found only one pair and one triplet of subspecies that share highly similar DNA barcodes: (1) *Theretra oldenlandiae oldenlandiae/lewini,* for which we found no shared haplotype, despite dense sampling (82 samples of *lewini* and 6 of *oldenlandiae*); and (2) *Theretra indistincta indistincta/manuselensis/papuensis*, a triplet possibly representing a case of synonymy.

## Discussion

### Identification and discrimination of Australian sphingid moths

The barcode reference library developed in this study provides coverage for 70 of the 72 currently valid Australian sphingid species and each of these species possesses a diagnostic array of DNA barcodes on the continent. Our results revealed seven species overlooked by past taxonomic work and five of these have recently been described, raising the species count for *Psilogramma* from five to seven, *Hopliocnema* from one to three, and *Coenotes* from one to two [23], [41], [42], [44]. One species of *Acosmeryx* and *Hippotion johanna* need revalidation. These changes raise the species count for Australian sphingids to 75, a 17% increase from the last checklist for the family [22]. Our work also led to the unexpected discovery that the Convolvulus Hawkmoth, *Agrius convolvuli*, an emerging model species [49] is almost certainly two species. Its division into two species is relevant to all studies on this species (e.g. the identification of its sex pheromones [50]) and may be of practical importance as it is a pest on sweet potatoes, a major crop in Papua New Guinea [51].

### Extent of the Linnean shortfall

The Linnean shortfall [6] refers to the fact that most species on Earth are yet to be formally described, and implies that our current census of species (and other meaningful evolutionary units used by taxonomists, such as subspecies) is incomplete. In the case of Australian sphingids, our acquisition of DNA barcodes revealed seven overlooked species. While this result was perhaps unexpected for a fauna considered as taxonomically mature, its significance is twofold. On one hand, it must be noted that all of the newly revealed species are highly similar morphologically to previously known taxa, and thus the integration of DNA barcodes played a key role in circumscribing these species and revealing their distinctness. On the other hand, these discoveries also reflect insufficient sampling effort as most of the new species originate from areas in the outback, where limited collecting efforts have occurred. In addition, our results question the taxonomic status of 13 other species whose Australian lineages show marked sequence divergence from their counterparts outside Australia. Finally, eight other Australian species show close barcode congruence with lineages in other settings that are assigned to different species (Fig. 3). In total, 18 species (three show both splitting and lumping) – almost a quarter of the continental fauna – possess a taxonomic assignment that requires further study. The resolution of each case will require a thorough and critical revision of species diagnoses using an integrative approach, and will demand consideration of names currently listed as junior synonyms. Tackling the nomenclatural part of this work is a particular challenge because sphingids have attracted so much attention from taxonomists resulting, for example, in 14 synonyms for *A. convolvuli* [21]. Fortunately, recent progress in the recovery of whole or partial DNA barcodes from old type specimens will aid resolution of these nomenclatural conundrums [32], [52].

### Extent of the Wallacean shortfall

**Figure 4:**
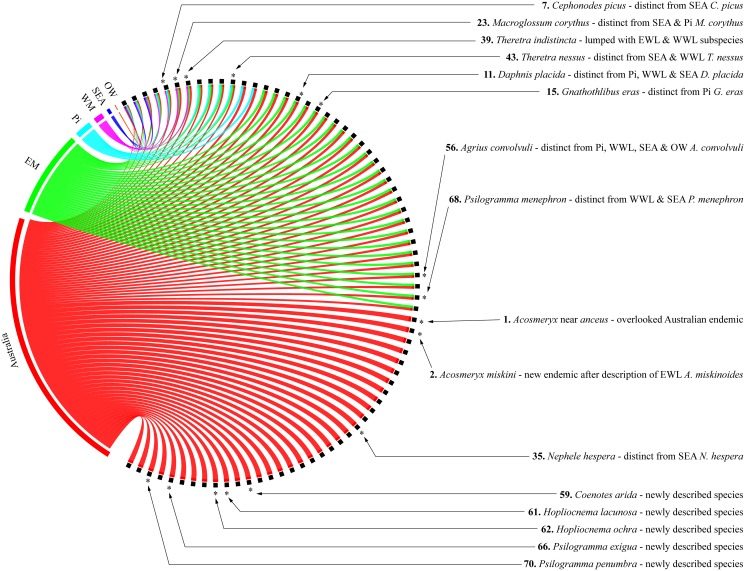
Distribution of Australian sphingid species. All 75 recognized species are linked by one or more coloured ribbons to the boundaries of their distributions (abbreviations as in Table 1). Species are ordered by distribution type, from the Australian endemics at the bottom left toward taxa with broader ranges at the top. Stars mark all species whose distributions were altered by our work, while text provides details about the cause of their distributional shift. The figure was assembled with the online application of Circos [57].

Knowledge of species distributions is fundamental to the design of sound conservation strategies and gaps in such information have been termed the “Wallacean shortfall” by biogeographers and conservation biologists [6]. Besides the scarcity of distributional data for most species, incorrect or incomplete delineation of taxonomic units will, in most cases, lead to incorrect information on species distributions, impeding efforts to understand the historical and contemporaneous processes underlying the occurrence patterns of species or subspecies [6], [53]. This study has revealed a significant mismatch between past understanding of the taxonomic diversity of Australian sphingids and that revealed through molecular analysis, especially when results are placed in a broader biogeographical context. The recent description of two Australian species each of *Psilogramma* and *Hopliocnema* [23], [41], [42], of one new species of *Coenotes* [44], and the separation by Vaglia & Haxaire [54] of the Papuan *A. miskinoides* from its Australian counterpart, *A. miskini*, were all motivated by DNA barcode data coupled subsequently with morphological analysis. These updates raise the number of endemic Australian species and subspecies from 26 to 34 (Fig. 4, Table S4). Further analysis of *N. hespera* may add another endemic species, while the possible synonymy of subspecies of *T. indistincta* may change its status from one polytypic species with an endemic Australian subspecies to a monotypic species distributed over Australia and the Malesian region.

Our results additionally revealed that seven species with broad ranges (namely *Cephonodes picus, Macroglossum corythus, Theretra nessus, Daphnis placida, Nephele hespera, Agrius convolvuli,* and *Psilogramma menephron*; see Fig. 4, and Fig. S5) actually comprise two (or more) genetically differentiated lineages likely to represent distinct species. In fact, in all of these cases, the genetic split is associated to a geographical disjunction. Although our current sampling of specimens with a DNA barcode remains too sparse in the region to precisely delimit the ranges of these newly circumscribed units, it is worth noting that in all cases the genetic lineage including the Australian representatives never extend westward beyond the Wallacean region. This result fits previous observations of the region as representing a major faunal discontinuity for Malesian sphingids, although it remains unclear where precisely the disjunctions occur and whether they match the modified course of Wallace’s Line proposed by Beck *et al.* [55] for sphingids. Overall, our results indicate that the Australian sphingid fauna comprises two large biogeographical subsets (Fig. 4), one endemic to the continent (30 species and 4 subspecies, 45% of the fauna) and one including taxa also occurring on Pacific Islands and/or in the Papuan and Wallacean region (34 species/subspecies, 45%). A third small subset includes just seven Australian species (10%) whose distributions extend westward beyond the Wallacean region (Fig. 4). The situation of those taxa found in Australia and on Pacific Islands deserves further attention. It is, for instance, interesting that the two species found in Australia and in the Pacific, but not in Papua New Guinea, occur in New Caledonia (N. subvaria) or New Caledonia and Fiji (Hippotion scrofa) but not in the Solomon Islands. In contrast, the three Australian species occurring on the Solomon Islands (*D. moorei, T. nessus* and *E. splendens*) are all also present on New Guinea. Overall, among the 11 Australian species or subspecies that also occur on Pacific islands (Table 1; Fig. 4), eight were sampled from these islands and six revealed genetic divergences ranging from 1 to 2% (e.g. 1% for *D. moorei* in Solomon Islands, 1.5% for *H. scrofa* in New Caledonia and Fiji, 1.4% for *A. convolvuli* in New Caledonia), while two (*H. velox* and *T. silhetensis intersecta*) were genetically undifferentiated. These relatively low divergences likely reflect the recent history of colonization of these islands from Australia or Papua New Guinea during the Pleistocene, when periods of lowered sea level facilitated dispersal across sea gaps. Climatic events or human-mediated colonization might also have caused contemporaneous range expansion in Pacific islands; *H. scrofa* was for instance unrecorded from Fiji before 1975 despite sustained collecting efforts before then (G. Robinson, personal communication), and the genetic identity of the specimen sampled here with one from New Caledonia (Fig. S5) supports this hypothesis of a recent colonization event.

The taxonomic implications of these genetic divergences between allopatric populations require further examination, but these island “populations” certainly represent important evolutionary units, yet another layer of the Wallacean shortfall concealed by their current taxonomic treatment. We did examine some subspecies already described from Pacific islands and they show comparable levels of genetic divergence (e.g. 1.9% between *D. p. placida* and *D. p. salomonis* from the Solomon Islands) or even less (e.g. only 0.6% between *T. n. nessus* and *T. n. albata* from New Caledonia and Vanuatu). Judging from genetic evidence alone, the island lineages of some Australian species may deserve at least subspecific status, and confirmation by morphology should be sought.

### Conclusions

The Linnean and Wallacean shortfalls have been stressed as two critical gaps in our knowledge of global biodiversity. Overlooked cryptic diversity and inadequate delineation of species boundaries inevitably create erroneous distribution patterns impeding the development of conservation strategies that focus on the right objects and the right places. In groups that have received little taxonomic attention, such as tropical and small-bodied invertebrates, these shortfalls have been recognized as substantial [9], [56]. However, this study reveals that a taxonomically mature insect fauna can also be strongly impacted by these shortfalls. Here, we have illustrated a solution involving the integration of DNA barcodes with an established taxonomic system based largely on morphology. This approach provides a robust means to identify and correct possible discrepancies in our current understanding of species and their distributions.

## Supporting Information

Figure S1 **NJ phylogram for Australian sphingid records.**
(PDF)

Figure S2 **28S–D2 rDNA sequence alignments.**
(PDF)

Figure S3 **Overlooked diversity within** ***Acosmeryx anceus.***
(PDF)

Figure S4 **Consensus tree (MP) for records of** ***Hippotion brennus*** **and** ***H. joiceyi.***
(PDF)

Figure S5 **NJ phylogram for Australian and non-Australian records.**
(PDF)

Figure S6 **Geographical structure of genetic variation in the Convolvulus Hawkmoth.**
(PDF)

Figure S7 **Consensus tree (MP) for records within the** ***Theretra oldenlandiae/insignis*** **complex.**
(PDF)

Table S1 **List of Australian records.**
(PDF)

Table S2 **List of conspecific records from outside Australia.**
(PDF)

Table S3 **List of heterospecific records from outside Australia.**
(PDF)

Table S4 **List of endemic species and subspecies of Sphingidae in Australia.**
(PDF)

Table S5 **DNA barcode matches at subspecies and species levels.**
(PDF)

Appendix S1 **Sequencing methods.**
(DOCX)
