# Supplementary material for: Australian Sphingidae – DNA Barcodes Challenge Current Species Boundaries and Distributions
Source: PLoS One. 2014 Jul 2;9(7):e101108. doi: 10.1371/journal.pone.0101108 (PMC4079597; doi:10.1371/journal.pone.0101108)
Supplement: Figure S1 — NJ phylogram for Australian sphingid records. (PDF) [file pone.0101108.s001.pdf]

Figure S1: Phylogram derived from the Neighbour Joining analysis of K2P distances by BOLD of 1054 Australian sphingid records. Taxa in red frames are those with intraspecific distances equal or above 2%; records highlighted in red are the sequenced holotypes within genus *Psilogramma*.

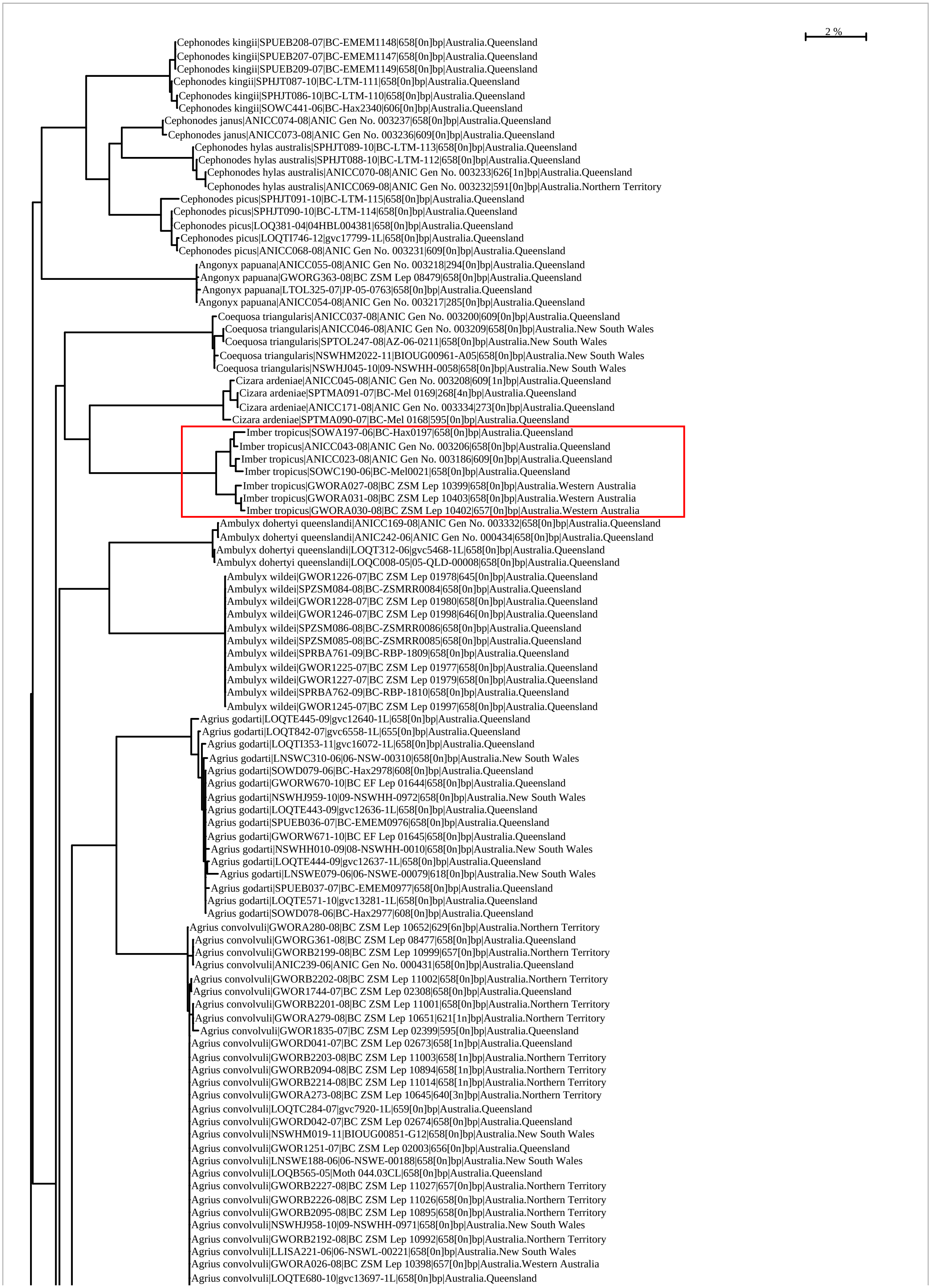
2 %
Cephonodes kingii|SPUEB208-07|BC-EMEM1148|658[0n]bp|Australia.Queensland
Cephonodes kingii|SPUEB207-07|BC-EMEM1147|658[0n]bp|Australia.Queensland
Cephonodes kingii|SPUEB209-07|BC-EMEM1149|658[0n]bp|Australia.Queensland
Cephonodes kingii|SPHJT087-10|BC-LTM-111|658[0n]bp|Australia.Queensland
Cephonodes kingii|SPHJT086-10|BC-LTM-110|658[0n]bp|Australia.Queensland
Cephonodes kingii|SOWC441-06|BC-Hax2340|606[0n]bp|Australia.Queensland
Cephonodes janus|ANICC074-08|ANIC Gen No. 003237|658[0n]bp|Australia.Queensland
Cephonodes janus|ANICC073-08|ANIC Gen No. 003236|609[0n]bp|Australia.Queensland
Cephonodes hylas australis|SPHJT089-10|BC-LTM-113|658[0n]bp|Australia.Queensland
Cephonodes hylas australis|SPHJT088-10|BC-LTM-112|658[0n]bp|Australia.Queensland
Cephonodes hylas australis|ANICC070-08|ANIC Gen No. 003233|626[1n]bp|Australia.Queensland
Cephonodes hylas australis|ANICC069-08|ANIC Gen No. 003232|591[0n]bp|Australia.Northern Territory
Cephonodes picus|SPHJT091-10|BC-LTM-115|658[0n]bp|Australia.Queensland
Cephonodes picus|SPHJT090-10|BC-LTM-114|658[0n]bp|Australia.Queensland
Cephonodes picus|LOQ381-04|04HBL004381|658[0n]bp|Australia.Queensland
Cephonodes picus|LOQTI746-12|gvc17799-1L|658[0n]bp|Australia.Queensland
Cephonodes picus|ANICC068-08|ANIC Gen No. 003231|609[0n]bp|Australia.Queensland
Angonyx papuana|ANICC055-08|ANIC Gen No. 003218|294[0n]bp|Australia.Queensland
Angonyx papuana|GWORG363-08|BC ZSM Lep 08479|658[0n]bp|Australia.Queensland
Angonyx papuana|LTOL325-07|JP-05-0763|658[0n]bp|Australia.Queensland
Angonyx papuana|ANICC054-08|ANIC Gen No. 003217|285[0n]bp|Australia.Queensland
Coequosa triangularis|ANICC037-08|ANIC Gen No. 003200|609[0n]bp|Australia.Queensland
Coequosa triangularis|ANICC046-08|ANIC Gen No. 003209|658[0n]bp|Australia.New South Wales
Coequosa triangularis|SPTOL247-08|AZ-06-0211|658[0n]bp|Australia.New South Wales
Coequosa triangularis|NSWHM2022-11|BIOUG00961-A05|658[0n]bp|Australia.New South Wales
Coequosa triangularis|NSWHJ045-10|09-NSWHH-0058|658[0n]bp|Australia.New South Wales
Cizara ardeniae|ANICC045-08|ANIC Gen No. 003208|609[1n]bp|Australia.Queensland
Cizara ardeniae|SPTMA091-07|BC-Mel 0169|268[4n]bp|Australia.Queensland
Cizara ardeniae|ANICC171-08|ANIC Gen No. 003334|273[0n]bp|Australia.Queensland
Cizara ardeniae|SPTMA090-07|BC-Mel 0168|595[0n]bp|Australia.Queensland
Imber tropicus|SOWA197-06|BC-Hax0197|658[0n]bp|Australia.Queensland
Imber tropicus|ANICC043-08|ANIC Gen No. 003206|658[0n]bp|Australia.Queensland
Imber tropicus|ANICC023-08|ANIC Gen No. 003186|609[0n]bp|Australia.Queensland
Imber tropicus|SOWC190-06|BC-Mel0021|658[0n]bp|Australia.Queensland
Imber tropicus|GWORA027-08|BC ZSM Lep 10399|658[0n]bp|Australia.Western Australia
Imber tropicus|GWORA031-08|BC ZSM Lep 10403|658[0n]bp|Australia.Western Australia
Imber tropicus|GWORA030-08|BC ZSM Lep 10402|657[0n]bp|Australia.Western Australia
Ambulyx dohertyi queenslandi|ANICC169-08|ANIC Gen No. 003332|658[0n]bp|Australia.Queensland
Ambulyx dohertyi queenslandi|ANIC242-06|ANIC Gen No. 000434|658[0n]bp|Australia.Queensland
Ambulyx dohertyi queenslandi|LOQT312-06|gvc5468-1L|658[0n]bp|Australia.Queensland
Ambulyx dohertyi queenslandi|LOQC008-05|05-QLD-00008|658[0n]bp|Australia.Queensland
Ambulyx wildei|GWOR1226-07|BC ZSM Lep 01978|645[0n]bp|Australia.Queensland
Ambulyx wildei|SPZSM084-08|BC-ZSMRR0084|658[0n]bp|Australia.Queensland
Ambulyx wildei|GWOR1228-07|BC ZSM Lep 01980|658[0n]bp|Australia.Queensland
Ambulyx wildei|GWOR1246-07|BC ZSM Lep 01998|646[0n]bp|Australia.Queensland
Ambulyx wildei|SPZSM086-08|BC-ZSMRR0086|658[0n]bp|Australia.Queensland
Ambulyx wildei|SPZSM085-08|BC-ZSMRR0085|658[0n]bp|Australia.Queensland
Ambulyx wildei|SPRBA761-09|BC-RBP-1809|658[0n]bp|Australia.Queensland
Ambulyx wildei|GWOR1225-07|BC ZSM Lep 01977|658[0n]bp|Australia.Queensland
Ambulyx wildei|GWOR1227-07|BC ZSM Lep 01979|658[0n]bp|Australia.Queensland
Ambulyx wildei|SPRBA762-09|BC-RBP-1810|658[0n]bp|Australia.Queensland
Ambulyx wildei|GWOR1245-07|BC ZSM Lep 01997|658[0n]bp|Australia.Queensland
Agrius godarti|LOQTE445-09|gvc12640-1L|658[0n]bp|Australia.Queensland
Agrius godarti|LOQT842-07|gvc6558-1L|655[0n]bp|Australia.Queensland
Agrius godarti|LOQTI353-11|gvc16072-1L|658[0n]bp|Australia.Queensland
Agrius godarti|LNSWC310-06|06-NSW-00310|658[0n]bp|Australia.New South Wales
Agrius godarti|SOWD079-06|BC-Hax2978|608[0n]bp|Australia.Queensland
Agrius godarti|GWORW670-10|BC EF Lep 01644|658[0n]bp|Australia.Queensland
Agrius godarti|NSWHJ959-10|09-NSWHH-0972|658[0n]bp|Australia.New South Wales
Agrius godarti|LOQTE443-09|gvc12636-1L|658[0n]bp|Australia.Queensland
Agrius godarti|SPUEB036-07|BC-EMEM0976|658[0n]bp|Australia.Queensland
Agrius godarti|GWORW671-10|BC EF Lep 01645|658[0n]bp|Australia.Queensland
Agrius godarti|NSWHH010-09|08-NSWHH-0010|658[0n]bp|Australia.New South Wales
Agrius godarti|LOQTE444-09|gvc12637-1L|658[0n]bp|Australia.Queensland
Agrius godarti|LNSWE079-06|06-NSWE-00079|618[0n]bp|Australia.New South Wales
Agrius godarti|SPUEB037-07|BC-EMEM0977|658[0n]bp|Australia.Queensland
Agrius godarti|LOQTE571-10|gvc13281-1L|658[0n]bp|Australia.Queensland
Agrius godarti|SOWD078-06|BC-Hax2977|608[0n]bp|Australia.Queensland
Agrius convolvuli|GWORA280-08|BC ZSM Lep 10652|629[6n]bp|Australia.Northern Territory
Agrius convolvuli|GWORG361-08|BC ZSM Lep 08477|658[0n]bp|Australia.Queensland
Agrius convolvuli|GWORB2199-08|BC ZSM Lep 10999|657[0n]bp|Australia.Northern Territory
Agrius convolvuli|ANIC239-06|ANIC Gen No. 000431|658[0n]bp|Australia.Queensland
Agrius convolvuli|GWORB2202-08|BC ZSM Lep 11002|658[0n]bp|Australia.Northern Territory
Agrius convolvuli|GWOR1744-07|BC ZSM Lep 02308|658[0n]bp|Australia.Queensland
Agrius convolvuli|GWORB2201-08|BC ZSM Lep 11001|658[0n]bp|Australia.Northern Territory
Agrius convolvuli|GWORA279-08|BC ZSM Lep 10651|621[1n]bp|Australia.Northern Territory
Agrius convolvuli|GWOR1835-07|BC ZSM Lep 02399|595[0n]bp|Australia.Queensland
Agrius convolvuli|GWORD041-07|BC ZSM Lep 02673|658[1n]bp|Australia.Queensland
Agrius convolvuli|GWORB2203-08|BC ZSM Lep 11003|658[1n]bp|Australia.Northern Territory
Agrius convolvuli|GWORB2094-08|BC ZSM Lep 10894|658[1n]bp|Australia.Northern Territory
Agrius convolvuli|GWORB2214-08|BC ZSM Lep 11014|658[1n]bp|Australia.Northern Territory
Agrius convolvuli|GWORA273-08|BC ZSM Lep 10645|640[3n]bp|Australia.Northern Territory
Agrius convolvuli|LOQTC284-07|gvc7920-1L|659[0n]bp|Australia.Queensland
Agrius convolvuli|GWORD042-07|BC ZSM Lep 02674|658[0n]bp|Australia.Queensland
Agrius convolvuli|NSWHM019-11|BIOUG00851-G12|658[0n]bp|Australia.New South Wales
Agrius convolvuli|GWOR1251-07|BC ZSM Lep 02003|656[0n]bp|Australia.Queensland
Agrius convolvuli|LNSWE188-06|06-NSWE-00188|658[0n]bp|Australia.New South Wales
Agrius convolvuli|LOQB565-05|Moth 044.03CL|658[0n]bp|Australia.Queensland
Agrius convolvuli|GWORB2227-08|BC ZSM Lep 11027|657[0n]bp|Australia.Northern Territory
Agrius convolvuli|GWORB2226-08|BC ZSM Lep 11026|658[0n]bp|Australia.Northern Territory
Agrius convolvuli|GWORB2095-08|BC ZSM Lep 10895|658[0n]bp|Australia.Northern Territory
Agrius convolvuli|NSWHJ958-10|09-NSWHH-0971|658[0n]bp|Australia.New South Wales
Agrius convolvuli|GWORB2192-08|BC ZSM Lep 10992|658[0n]bp|Australia.Northern Territory
Agrius convolvuli|LLISA221-06|06-NSWL-00221|658[0n]bp|Australia.New South Wales
Agrius convolvuli|GWORA026-08|BC ZSM Lep 10398|657[0n]bp|Australia.Western Australia
Agrius convolvuli|LOQTE680-10|gvc13697-1L|658[0n]bp|Australia.Queensland

Agrius convolvuli|LLISA221-06|06-NSWL-00221|658[0n]bp|Australia.New South Wales
Agrius convolvuli|GWORA026-08|BC ZSM Lep 10398|657[0n]bp|Australia.Western Australia
Agrius convolvuli|LOQTE680-10|gvc13697-1L|658[0n]bp|Australia.Queensland
Agrius convolvuli|GWORA033-08|BC ZSM Lep 10405|657[0n]bp|Australia.Western Australia
Agrius convolvuli|NSWHM010-11|BIOUG00851-G03|658[0n]bp|Australia.New South Wales
Agrius convolvuli|GWORG300-08|BC ZSM Lep 08416|658[0n]bp|Australia.Queensland
Agrius convolvuli|PHLCC1137-11|BIOUG01235-A01|658[0n]bp|Australia.Australian Capital Territory
Agrius convolvuli|LOQB567-05|Moth 046.03CL|658[0n]bp|Australia.Queensland
Agrius convolvuli|LOQT747-06|gvc6449-1L|658[0n]bp|Australia.Queensland
Agrius convolvuli|IMLQ052-07|IM06-0212|658[0n]bp|Australia.Queensland
Agrius convolvuli|GWORA267-08|BC ZSM Lep 10639|658[0n]bp|Australia.Northern Territory
Agrius convolvuli|GWORB2213-08|BC ZSM Lep 11013|657[0n]bp|Australia.Northern Territory
Agrius convolvuli|LNSWE136-06|06-NSWE-00136|658[0n]bp|Australia.New South Wales
Agrius convolvuli|SPUEB033-07|BC-EMEM0973|658[0n]bp|Australia.Western Australia
Agrius convolvuli|LOQB569-05|Moth 048.03CL|658[0n]bp|Australia.Queensland
Agrius convolvuli|LOQB074-05|Moth 074.03LZ|658[0n]bp|Australia.Queensland
Agrius convolvuli|NSWHH011-09|08-NSWHH-0011|658[0n]bp|Australia.New South Wales
Agrius convolvuli|LNSWE081-06|06-NSWE-00081|658[0n]bp|Australia.New South Wales
Agrius convolvuli|NSWHM029-11|BIOUG00851-H10|658[0n]bp|Australia.New South Wales
Agrius convolvuli|LSM256-11|K278668|658[0n]bp|Australia.New South Wales
Agrius convolvuli|GWOR1247-07|BC ZSM Lep 01999|658[0n]bp|Australia.Queensland
Agrius convolvuli|GWORI528-09|BC ZSM Lep 13276|658[0n]bp|Australia.Northern Territory
Agrius convolvuli|NSWBB1144-08|07-NSWBB-1144|657[0n]bp|Australia.New South Wales
Agrius convolvuli|GWORC185-07|BC ZSM Lep 02535|658[0n]bp|Australia.Queensland
Agrius convolvuli|GWORB2195-08|BC ZSM Lep 10995|658[0n]bp|Australia.Northern Territory
Agrius convolvuli|GWORA032-08|BC ZSM Lep 10404|658[0n]bp|Australia.Western Australia
Agrius convolvuli|GWORI485-09|BC ZSM Lep 13233|658[0n]bp|Australia.Northern Territory
Agrius convolvuli|GWORI500-09|BC ZSM Lep 13248|658[0n]bp|Australia.Northern Territory
Agrius convolvuli|IMLQ131-07|IM06-0439|658[0n]bp|Australia.Queensland
Agrius convolvuli|GWORI526-09|BC ZSM Lep 13274|658[0n]bp|Australia.Northern Territory
Agrius convolvuli|GWORB2187-08|BC ZSM Lep 10987|658[0n]bp|Australia.Northern Territory
Agrius convolvuli|NSWHJ960-10|09-NSWHH-0973|658[0n]bp|Australia.New South Wales
Agrius convolvuli|LOQB566-05|Moth 045.03CL|658[0n]bp|Australia.Queensland
Agrius convolvuli|LOQTE740-10|gvc13992-1L|658[0n]bp|Australia.Queensland
Agrius convolvuli|GWORH539-09|BC ZSM Lep 10253|658[0n]bp|Australia.Queensland
Agrius convolvuli|GWORA262-08|BC ZSM Lep 10634|658[0n]bp|Australia.Northern Territory
Agrius convolvuli|GWORB2200-08|BC ZSM Lep 11000|657[0n]bp|Australia.Northern Territory
Agrius convolvuli|GWORA014-08|BC ZSM Lep 10386|658[0n]bp|Australia.Western Australia
Agrius convolvuli|LOQB075-05|Moth 075.03LZ|658[0n]bp|Australia.Queensland
Agrius convolvuli|ANICB366-06|ANIC Gen No. 000167|658[0n]bp|Australia.Queensland
Agrius convolvuli|NSWBB1227-08|07-NSWBB-1227|658[0n]bp|Australia.New South Wales
Agrius convolvuli|GWORB2196-08|BC ZSM Lep 10996|658[0n]bp|Australia.Northern Territory
Agrius convolvuli|NSWHM456-11|BIOUG00912-G05|658[0n]bp|Australia.New South Wales
Agrius convolvuli|GWORC183-07|BC ZSM Lep 02533|658[0n]bp|Australia.Queensland
Agrius convolvuli|GWORB2191-08|BC ZSM Lep 10991|656[0n]bp|Australia.Northern Territory
Agrius convolvuli|GWORC144-07|BC ZSM Lep 02494|655[0n]bp|Australia.Queensland
Agrius convolvuli|GWORB2194-08|BC ZSM Lep 10994|656[0n]bp|Australia.Northern Territory
Agrius convolvuli|GWORA025-08|BC ZSM Lep 10397|655[0n]bp|Australia.Western Australia
Agrius convolvuli|LOQT806-07|gvc6516-1L|656[0n]bp|Australia.Queensland
Agrius convolvuli|GWORB2188-08|BC ZSM Lep 10988|655[0n]bp|Australia.Northern Territory
Agrius convolvuli|GWORA101-08|BC ZSM Lep 10473|646[0n]bp|Australia.Northern Territory
Agrius convolvuli|GWORC186-07|BC ZSM Lep 02536|647[0n]bp|Australia.Queensland
Agrius convolvuli|GWORA189-08|BC ZSM Lep 10561|645[0n]bp|Australia.Northern Territory
Agrius convolvuli|GWORC180-07|BC ZSM Lep 02530|631[0n]bp|Australia.Queensland
Agrius convolvuli|GWORC182-07|BC ZSM Lep 02532|632[0n]bp|Australia.Queensland
Agrius convolvuli|GWORB2198-08|BC ZSM Lep 10998|632[0n]bp|Australia.Northern Territory
Agrius convolvuli|GWORC184-07|BC ZSM Lep 02534|632[0n]bp|Australia.Queensland
Agrius convolvuli|GWORC181-07|BC ZSM Lep 02531|632[0n]bp|Australia.Queensland
Agrius convolvuli|GWORB2193-08|BC ZSM Lep 10993|632[0n]bp|Australia.Northern Territory
Agrius convolvuli|GWORB892-07|BC ZSM Lep 01362|632[0n]bp|Australia.Queensland
Agrius convolvuli|LOQB073-05|Moth 073.03LZ|649[0n]bp|Australia.Queensland
Agrius convolvuli|GWORA142-08|BC ZSM Lep 10514|563[0n]bp|Australia.Northern Territory
Agrius convolvuli|ANICB1117-07|ANIC Gen No. 003135|647[0n]bp|Australia.South Australia
Agrius convolvuli|LOQ341-04|04HBL004341|593[0n]bp|Australia.Queensland
Agrius convolvuli|GWORA274-08|BC ZSM Lep 10646|639[0n]bp|Australia.Northern Territory
Agrius convolvuli|GWORB2092-08|BC ZSM Lep 10892|618[0n]bp|Australia.Northern Territory
Agrius convolvuli|GWORB854-07|BC ZSM Lep 01324|617[0n]bp|Australia.Queensland
Agrius convolvuli|LOQB077-05|Moth 077.03LZ|616[0n]bp|Australia.Queensland
Agrius convolvuli|GWORA266-08|BC ZSM Lep 10638|617[0n]bp|Australia.Northern Territory
Agrius convolvuli|GWORA281-08|BC ZSM Lep 10653|638[1n]bp|Australia.Northern Territory
Agrius convolvuli|GWORA144-08|BC ZSM Lep 10516|618[0n]bp|Australia.Northern Territory
Agrius convolvuli|GWORB2069-08|BC ZSM Lep 10869|609[0n]bp|Australia.Northern Territory
Agrius convolvuli|GWORB2093-08|BC ZSM Lep 10893|624[0n]bp|Australia.Northern Territory
Agrius convolvuli|LOQB072-05|Moth 072.03LZ|620[0n]bp|Australia.Queensland
Agrius convolvuli|GWORA160-08|BC ZSM Lep 10532|602[0n]bp|Australia.Western Australia
Agrius convolvuli|GWORA159-08|BC ZSM Lep 10531|587[0n]bp|Australia.Western Australia
Agrius convolvuli|LNSWE009-06|06-NSWE-00009|600[0n]bp|Australia.New South Wales
Agrius convolvuli|LSM875-11|K287774|628[0n]bp|Australia.New South Wales
Agrius convolvuli|GWORA140-08|BC ZSM Lep 10512|610[1n]bp|Australia.Western Australia
Agrius convolvuli|GWOR1836-07|BC ZSM Lep 02400|586[0n]bp|Australia.Queensland
Agrius convolvuli|LOQB313-05|Moth 313.01LZ|570[0n]bp|Australia.Queensland
Agrius convolvuli|LOQB076-05|Moth 076.03LZ|573[0n]bp|Australia.Queensland
Agrius convolvuli|LOQB078-05|Moth 078.03LZ|572[0n]bp|Australia.Queensland
Agrius convolvuli|LOQB071-05|Moth 071.03LZ|572[0n]bp|Australia.Queensland
Agrius convolvuli|GWORA143-08|BC ZSM Lep 10515|552[1n]bp|Australia.Northern Territory
Agrius convolvuli|LOQB568-05|Moth 047.03CL|528[0n]bp|Australia.Queensland
Agrius convolvuli|GWORI527-09|BC ZSM Lep 13275|658[0n]bp|Australia.Northern Territory
Agrius convolvuli|NSWHJ954-10|09-NSWHH-0967|658[0n]bp|Australia.New South Wales
Tetrachroa edwardsi|SOWD284-06|BC-Hax3183|658[0n]bp|Australia.Queensland
Tetrachroa edwardsi|ANICB284-06|ANIC Gen No. 000135|646[0n]bp|Australia.Queensland
Tetrachroa edwardsi|SOWD285-06|BC-Hax3184|658[0n]bp|Australia.Queensland
Tetrachroa edwardsi|SOWD286-06|BC-Hax3185|658[0n]bp|Australia.Queensland
Hopliocnema brachycera|ANICK404-10|10ANIC-07401|658[0n]bp|Australia.New South Wales
Hopliocnema brachycera|SPHJT157-12|BC-LTM-178|658[0n]bp|Australia.Western Australia
Hopliocnema brachycera|SPHJT158-12|BC-LTM-179|658[0n]bp|Australia.Western Australia
Hopliocnema brachycera|SPTOL205-07|MJM-96-0232|658[0n]bp|Australia.Northern Territory
Hopliocnema brachycera|ANICK405-10|10ANIC-07402|658[0n]bp|Australia.Western Australia
Hopliocnema brachycera|ANICK406-10|10ANIC-07403|658[0n]bp|Australia.South Australia
Hopliocnema brachycera|SPHJT144-11|BC-LTM-166|658[0n]bp|Australia.Northern Territory

Hopliocnema brachycera|ANICK405-10|10ANIC-07402|658[0n]bp|Australia.Western Australia
Hopliocnema brachycera|ANICK406-10|10ANIC-07403|658[0n]bp|Australia.South Australia
Hopliocnema brachycera|SPHJT144-11|BC-LTM-166|658[0n]bp|Australia.Northern Territory
Hopliocnema brachycera|ANICK402-10|10ANIC-07399|658[0n]bp|Australia.Victoria
Hopliocnema brachycera|SOWA731-06|BC-Hax0724|630[7n]bp|Australia.Western Australia
Hopliocnema brachycera|ANICC173-08|ANIC Gen No. 003336|609[0n]bp|Australia.Northern Territory
Hopliocnema brachycera|ANICK403-10|10ANIC-07400|609[0n]bp|Australia.South Australia
Hopliocnema brachycera|SPHJT143-11|BC-LTM-165|603[0n]bp|Australia.New South Wales
Hopliocnema brachycera|SPTOL206-07|MJM-96-0233|609[0n]bp|Australia.Northern Territory
Hopliocnema lacunosa|ANICK407-10|10ANIC-07404|658[0n]bp|Australia.Western Australia
Hopliocnema lacunosa|GWORO558-09|BC ZSM Lep 26354|658[0n]bp|Australia.Western Australia
Hopliocnema lacunosa|SPHJT154-12|BC-LTM-175|658[0n]bp|Australia.Western Australia
Hopliocnema lacunosa|SPHJT153-12|BC-LTM-174|658[0n]bp|Australia.Western Australia
Hopliocnema lacunosa|SPHJT155-12|BC-LTM-176|658[0n]bp|Australia.Western Australia
Hopliocnema ochra|SPHJT156-12|BC-LTM-177|658[0n]bp|Australia.Western Australia
Hopliocnema ochra|SPHJT159-12|BC-LTM-180|658[0n]bp|Australia.Western Australia
Hopliocnema ochra|GWORN199-09|BC ZSM Lep 18227|658[0n]bp|Australia.Western Australia
Hopliocnema ochra|GWORN198-09|BC ZSM Lep 18226|658[0n]bp|Australia.Western Australia
Hopliocnema ochra|GWORO578-09|BC ZSM Lep 26374|658[0n]bp|Australia.Western Australia
Hopliocnema ochra|GWORO577-09|BC ZSM Lep 26373|658[0n]bp|Australia.Western Australia
Synoecha marmorata|LTOL925-08|AZ-06-0210|657[0n]bp|Australia.New South Wales
Synoecha marmorata|SPTMC307-12|BC-Mel2308|658[0n]bp|Australia.Queensland
Synoecha marmorata|SPTMC306-12|BC-Mel2307|658[0n]bp|Australia.Queensland
Synoecha marmorata|ANICB362-06|ANIC Gen No. 000164|658[0n]bp|Australia.Queensland
Synoecha marmorata|ANICB265-06|ANIC Gen No. 001397|658[0n]bp|Australia.Queensland
Synoecha marmorata|SPTMC312-12|BC-Mel2313|658[0n]bp|Australia.Queensland
Synoecha marmorata|SPTMC309-12|BC-Mel2310|621[0n]bp|Australia.Queensland
Synoecha marmorata|SPTMC310-12|BC-Mel2311|639[0n]bp|Australia.Queensland
Synoecha marmorata|ANICC177-08|ANIC Gen No. 003340|609[0n]bp|Australia.Queensland
Synoecha marmorata|ANICB266-06|ANIC Gen No. 001398|637[0n]bp|Australia.Queensland
Synoecha marmorata|SPTMC308-12|BC-Mel2309|658[0n]bp|Australia.Queensland
Synoecha marmorata|SPTMC311-12|BC-Mel2312|633[0n]bp|Australia.Queensland
Leucomonia bethia|ANICC080-08|ANIC Gen No. 003243|658[0n]bp|Australia.Western Australia
Leucomonia bethia|GWORA135-08|BC ZSM Lep 10507|658[0n]bp|Australia.Western Australia
Leucomonia bethia|ANICC078-08|ANIC Gen No. 003241|658[0n]bp|Australia.Western Australia
Leucomonia bethia|ANICC077-08|ANIC Gen No. 003240|609[0n]bp|Australia.Northern Territory
Leucomonia bethia|SOWD283-06|BC-Hax3182|607[0n]bp|Australia.Queensland
Leucomonia bethia|ANICC079-08|ANIC Gen No. 003242|658[0n]bp|Australia.Western Australia
Synoecha marmorata|SPTMC305-12|BC-Mel2306|658[0n]bp|Australia.Northern Territory
Synoecha marmorata|SPTMC304-12|BC-Mel2305|658[0n]bp|Australia.Northern Territory
Coenotes eremophilae|SPTMA053-07|BC-Mel 0131|625[0n]bp|Australia.Western Australia
Coenotes eremophilae|SPHJT083-10|BC-LTM-107|658[0n]bp|Australia.Western Australia
Coenotes eremophilae|SOWF177-12|BC-Hax5035|658[0n]bp|Australia.Northern Territory
Coenotes eremophilae|LOQTC781-08|gvc8426-1L|658[0n]bp|Australia.Queensland
Coenotes eremophilae|SPHJT080-10|BC-LTM-104|658[0n]bp|Australia.Western Australia
Coenotes eremophilae|LOQTC769-08|gvc8414-1L|658[0n]bp|Australia.Queensland
Coenotes eremophilae|LOQTC771-08|gvc8416-1L|658[0n]bp|Australia.Queensland
Coenotes eremophilae|LOQTB023-07|gvc6797-1L|658[0n]bp|Australia.Queensland
Coenotes eremophilae|LOQTC783-08|gvc8428-1L|658[0n]bp|Australia.Queensland
Coenotes eremophilae|LOQT053-06|2006-LOQT-053|617[2n]bp|Australia.Queensland
Coenotes eremophilae|SPTMA056-07|BC-Mel 0134|658[0n]bp|Australia.Western Australia
Coenotes eremophilae|LOQT218-06|2006-LOQT-218|603[0n]bp|Australia.Queensland
Coenotes eremophilae|SPHJT081-10|BC-LTM-105|614[0n]bp|Australia.Western Australia
Coenotes eremophilae|LOQTC770-08|gvc8415-1L|658[0n]bp|Australia.Queensland
Coenotes eremophilae|SPHJT082-10|BC-LTM-106|658[0n]bp|Australia.Western Australia
Coenotes eremophilae|SOWF178-12|BC-Hax5036|658[0n]bp|Australia.Northern Territory
Coenotes eremophilae|LOQTC782-08|gvc8427-1L|632[0n]bp|Australia.Queensland
Psilogramma argos|SPHJT100-10|BC-LTM-124|658[0n]bp|Australia.Northern Territory
Psilogramma argos|ANICC072-08|ANIC Gen No. 003235|658[0n]bp|Australia.Northern Territory
Psilogramma argos|SPTMB093-09|BC-Mel 1105|658[0n]bp|Australia.Northern Territory
Psilogramma exigua|SPTMB091-09|BC-Mel 1103|658[0n]bp|Australia.Northern Territory
Psilogramma exigua|SPHJT118-10|BC-LTM-142|658[0n]bp|Australia.Northern Territory
Psilogramma exigua|SPHJT117-10|BC-LTM-141|658[0n]bp|Australia.Queensland
Psilogramma exigua|LOQTB045-07|gvc6811-1L|643[1n]bp|Australia.Queensland
Psilogramma exigua|SPTMB090-09|BC-Mel 1102|658[0n]bp|Australia.Northern Territory
Psilogramma exigua|SPHJT142-10|BC-LTM-164|658[0n]bp|Australia.Northern Territory
Psilogramma exigua|SPHJT120-10|BC-LTM-144|658[0n]bp|Australia.Northern Territory
Psilogramma exigua|GWORI484-09|BC ZSM Lep 13232|658[0n]bp|Australia.Northern Territory
Psilogramma exigua|SPTMB088-09|BC-Mel 1100|658[0n]bp|Australia.Northern Territory
Psilogramma exigua|SPTMC623-12|BC-Mel2624|658[0n]bp|Australia.Northern Territory
Psilogramma exigua|SPTMC626-12|BC-Mel2627|658[0n]bp|Australia.Northern Territory
Psilogramma exigua|SPHJT119-10|BC-LTM-143|658[0n]bp|Australia.Northern Territory
Psilogramma exigua|SPTMC627-12|BC-Mel2628|658[0n]bp|Australia.Northern Territory
Psilogramma exigua|SPTMB092-09|BC-Mel 1104|651[0n]bp|Australia.Northern Territory
Psilogramma exigua|SPTMB089-09|BC-Mel 1101|658[0n]bp|Australia.Northern Territory
Psilogramma exigua|SPTMC624-12|BC-Mel2625|630[0n]bp|Australia.Northern Territory
Psilogramma hausmanni|SPZSM052-08|BC-ZSMRR0052|658[4n]bp|Australia.New South Wales
Psilogramma casuarinae|SPHJT113-10|BC-LTM-137|658[0n]bp|Australia.Queensland
Psilogramma casuarinae|AMWW009-11|K290749|658[0n]bp|Australia.New South Wales
Psilogramma casuarinae|AMWW027-11|K290767|658[0n]bp|Australia.New South Wales
Psilogramma casuarinae|NSWHM027-11|BIOUG00851-H08|658[0n]bp|Australia.New South Wales
Psilogramma casuarinae|NSWBB1203-08|07-NSWBB-1203|658[0n]bp|Australia.New South Wales
Psilogramma casuarinae|SPTMA300-07|BC-Mel 0378|658[0n]bp|Australia.Queensland
Psilogramma casuarinae|IMLQ239-07|IM07-0193|658[0n]bp|Australia.Queensland
Psilogramma casuarinae|SPTMA296-07|BC-Mel 0374|658[0n]bp|Australia.Queensland
Psilogramma casuarinae|SPHJT092-10|BC-LTM-116|630[0n]bp|Australia.Queensland
Psilogramma casuarinae|LNSWE010-06|06-NSWE-00010|590[2n]bp|Australia.New South Wales
Psilogramma casuarinae|LNSWE110-06|06-NSWE-00110|582[0n]bp|Australia.New South Wales
Psilogramma casuarinae|SPHJT047-10|BC-JT-0028|407[0n]bp|Australia.Victoria
Psilogramma casuarinae|LOQTE795-10|gvc14173-1L|658[0n]bp|Australia.Queensland
Psilogramma casuarinae|NSWHJ076-10|09-NSWHH-0089|658[0n]bp|Australia.New South Wales
Psilogramma casuarinae|LNSWE085-06|06-NSWE-00085|657[0n]bp|Australia.New South Wales
Psilogramma casuarinae|NSWHJ088-10|09-NSWHH-0101|658[0n]bp|Australia.New South Wales
Psilogramma casuarinae|LOQTB025-07|gvc6799-1L|658[0n]bp|Australia.Queensland
Psilogramma casuarinae|LNSWE044-06|06-NSWE-00044|658[0n]bp|Australia.New South Wales
Psilogramma casuarinae|NSWHJ042-10|09-NSWHH-0055|658[0n]bp|Australia.New South Wales
Psilogramma casuarinae|SPHJT095-10|BC-LTM-119|658[0n]bp|Australia.Queensland
Psilogramma casuarinae|SPHJT115-10|BC-LTM-139|658[0n]bp|Australia.New South Wales
Psilogramma casuarinae|SPTMA298-07|BC-Mel 0376|658[0n]bp|Australia.Queensland

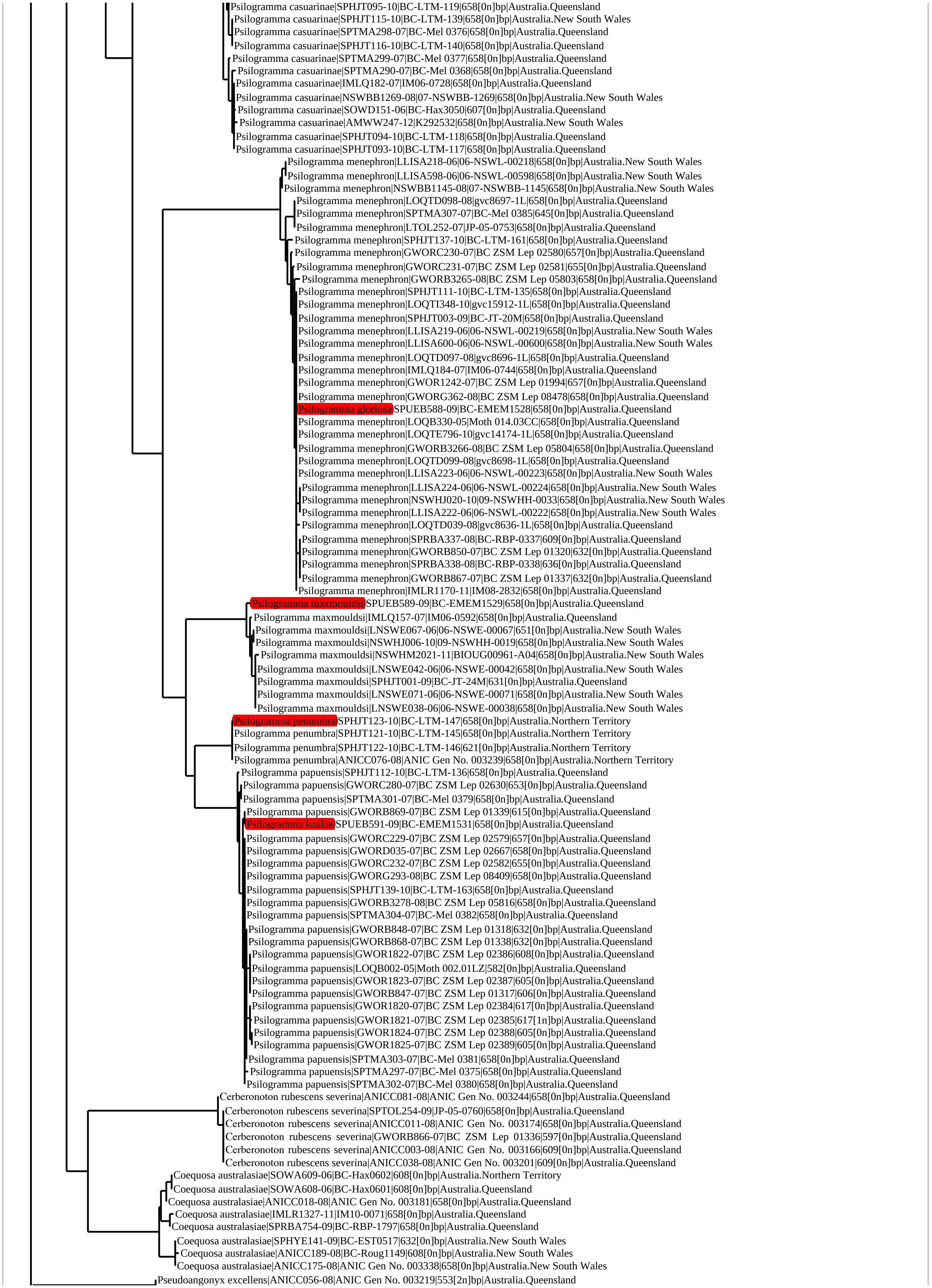
Psilogramma casuarinae|SPHJT095-10|BC-LTM-119|658[0n]bp|Australia.Queensland
Psilogramma casuarinae|SPHJT115-10|BC-LTM-139|658[0n]bp|Australia.New South Wales
Psilogramma casuarinae|SPTMA298-07|BC-Mel 0376|658[0n]bp|Australia.Queensland
Psilogramma casuarinae|SPHJT116-10|BC-LTM-140|658[0n]bp|Australia.Queensland
Psilogramma casuarinae|SPTMA299-07|BC-Mel 0377|658[0n]bp|Australia.Queensland
Psilogramma casuarinae|SPTMA290-07|BC-Mel 0368|658[0n]bp|Australia.Queensland
Psilogramma casuarinae|IMLQ182-07|IM06-0728|658[0n]bp|Australia.Queensland
Psilogramma casuarinae|NSWBB1269-08|07-NSWBB-1269|658[0n]bp|Australia.New South Wales
Psilogramma casuarinae|SOWD151-06|BC-Hax3050|607[0n]bp|Australia.Queensland
Psilogramma casuarinae|AMWW247-12|K292532|658[0n]bp|Australia.New South Wales
Psilogramma casuarinae|SPHJT094-10|BC-LTM-118|658[0n]bp|Australia.Queensland
Psilogramma casuarinae|SPHJT093-10|BC-LTM-117|658[0n]bp|Australia.Queensland
Psilogramma menephron|LLISA218-06|06-NSWL-00218|658[0n]bp|Australia.New South Wales
Psilogramma menephron|LLISA598-06|06-NSWL-00598|658[0n]bp|Australia.New South Wales
Psilogramma menephron|NSWBB1145-08|07-NSWBB-1145|658[0n]bp|Australia.New South Wales
Psilogramma menephron|LOQTD098-08|gvc8697-1L|658[0n]bp|Australia.Queensland
Psilogramma menephron|SPTMA307-07|BC-Mel 0385|645[0n]bp|Australia.Queensland
Psilogramma menephron|LTOL252-07|JP-05-0753|658[0n]bp|Australia.Queensland
Psilogramma menephron|SPHJT137-10|BC-LTM-161|658[0n]bp|Australia.Queensland
Psilogramma menephron|GWORC230-07|BC ZSM Lep 02580|657[0n]bp|Australia.Queensland
Psilogramma menephron|GWORC231-07|BC ZSM Lep 02581|655[0n]bp|Australia.Queensland
Psilogramma menephron|GWORB3265-08|BC ZSM Lep 05803|658[0n]bp|Australia.Queensland
Psilogramma menephron|SPHJT111-10|BC-LTM-135|658[0n]bp|Australia.Queensland
Psilogramma menephron|LOQTI348-10|gvc15912-1L|658[0n]bp|Australia.Queensland
Psilogramma menephron|SPHJT003-09|BC-JT-20M|658[0n]bp|Australia.Queensland
Psilogramma menephron|LLISA219-06|06-NSWL-00219|658[0n]bp|Australia.New South Wales
Psilogramma menephron|LLISA600-06|06-NSWL-00600|658[0n]bp|Australia.New South Wales
Psilogramma menephron|LOQTD097-08|gvc8696-1L|658[0n]bp|Australia.Queensland
Psilogramma menephron|IMLQ184-07|IM06-0744|658[0n]bp|Australia.Queensland
Psilogramma menephron|GWOR1242-07|BC ZSM Lep 01994|657[0n]bp|Australia.Queensland
Psilogramma menephron|GWORG362-08|BC ZSM Lep 08478|658[0n]bp|Australia.Queensland
Psilogramma gloriosa|SPUEB588-09|BC-EMEM1528|658[0n]bp|Australia.Queensland
Psilogramma menephron|LOQB330-05|Moth 014.03CC|658[0n]bp|Australia.Queensland
Psilogramma menephron|LOQTE796-10|gvc14174-1L|658[0n]bp|Australia.Queensland
Psilogramma menephron|GWORB3266-08|BC ZSM Lep 05804|658[0n]bp|Australia.Queensland
Psilogramma menephron|LOQTD099-08|gvc8698-1L|658[0n]bp|Australia.Queensland
Psilogramma menephron|LLISA223-06|06-NSWL-00223|658[0n]bp|Australia.New South Wales
Psilogramma menephron|LLISA224-06|06-NSWL-00224|658[0n]bp|Australia.New South Wales
Psilogramma menephron|NSWHJ020-10|09-NSWHH-0033|658[0n]bp|Australia.New South Wales
Psilogramma menephron|LLISA222-06|06-NSWL-00222|658[0n]bp|Australia.New South Wales
Psilogramma menephron|LOQTD039-08|gvc8636-1L|658[0n]bp|Australia.Queensland
Psilogramma menephron|SPRBA337-08|BC-RBP-0337|609[0n]bp|Australia.Queensland
Psilogramma menephron|GWORB850-07|BC ZSM Lep 01320|632[0n]bp|Australia.Queensland
Psilogramma menephron|SPRBA338-08|BC-RBP-0338|636[0n]bp|Australia.Queensland
Psilogramma menephron|GWORB867-07|BC ZSM Lep 01337|632[0n]bp|Australia.Queensland
Psilogramma menephron|IMLR1170-11|IM08-2832|658[0n]bp|Australia.Queensland
Psilogramma maxmouldsi|SPUEB589-09|BC-EMEM1529|658[0n]bp|Australia.Queensland
Psilogramma maxmouldsi|IMLQ157-07|IM06-0592|658[0n]bp|Australia.Queensland
Psilogramma maxmouldsi|LNSWE067-06|06-NSWE-00067|651[0n]bp|Australia.New South Wales
Psilogramma maxmouldsi|NSWHJ006-10|09-NSWHH-0019|658[0n]bp|Australia.New South Wales
Psilogramma maxmouldsi|NSWHM2021-11|BIOUG00961-A04|658[0n]bp|Australia.New South Wales
Psilogramma maxmouldsi|LNSWE042-06|06-NSWE-00042|658[0n]bp|Australia.New South Wales
Psilogramma maxmouldsi|SPHJT001-09|BC-JT-24M|631[0n]bp|Australia.Queensland
Psilogramma maxmouldsi|LNSWE071-06|06-NSWE-00071|658[0n]bp|Australia.New South Wales
Psilogramma maxmouldsi|LNSWE038-06|06-NSWE-00038|658[0n]bp|Australia.New South Wales
Psilogramma penumbra|SPHJT123-10|BC-LTM-147|658[0n]bp|Australia.Northern Territory
Psilogramma penumbra|SPHJT121-10|BC-LTM-145|658[0n]bp|Australia.Northern Territory
Psilogramma penumbra|SPHJT122-10|BC-LTM-146|621[0n]bp|Australia.Northern Territory
Psilogramma penumbra|ANICC076-08|ANIC Gen No. 003239|658[0n]bp|Australia.Northern Territory
Psilogramma papuensis|SPHJT112-10|BC-LTM-136|658[0n]bp|Australia.Queensland
Psilogramma papuensis|GWORC280-07|BC ZSM Lep 02630|653[0n]bp|Australia.Queensland
Psilogramma papuensis|SPTMA301-07|BC-Mel 0379|658[0n]bp|Australia.Queensland
Psilogramma papuensis|GWORB869-07|BC ZSM Lep 01339|615[0n]bp|Australia.Queensland
Psilogramma koalae|SPUEB591-09|BC-EMEM1531|658[0n]bp|Australia.Queensland
Psilogramma papuensis|GWORC229-07|BC ZSM Lep 02579|657[0n]bp|Australia.Queensland
Psilogramma papuensis|GWORD035-07|BC ZSM Lep 02667|658[0n]bp|Australia.Queensland
Psilogramma papuensis|GWORC232-07|BC ZSM Lep 02582|655[0n]bp|Australia.Queensland
Psilogramma papuensis|GWORG293-08|BC ZSM Lep 08409|658[0n]bp|Australia.Queensland
Psilogramma papuensis|SPHJT139-10|BC-LTM-163|658[0n]bp|Australia.Queensland
Psilogramma papuensis|GWORB3278-08|BC ZSM Lep 05816|658[0n]bp|Australia.Queensland
Psilogramma papuensis|SPTMA304-07|BC-Mel 0382|658[0n]bp|Australia.Queensland
Psilogramma papuensis|GWORB848-07|BC ZSM Lep 01318|632[0n]bp|Australia.Queensland
Psilogramma papuensis|GWORB868-07|BC ZSM Lep 01338|632[0n]bp|Australia.Queensland
Psilogramma papuensis|GWOR1822-07|BC ZSM Lep 02386|608[0n]bp|Australia.Queensland
Psilogramma papuensis|LOQB002-05|Moth 002.01LZ|582[0n]bp|Australia.Queensland
Psilogramma papuensis|GWOR1823-07|BC ZSM Lep 02387|605[0n]bp|Australia.Queensland
Psilogramma papuensis|GWORB847-07|BC ZSM Lep 01317|606[0n]bp|Australia.Queensland
Psilogramma papuensis|GWOR1820-07|BC ZSM Lep 02384|617[0n]bp|Australia.Queensland
Psilogramma papuensis|GWOR1821-07|BC ZSM Lep 02385|617[1n]bp|Australia.Queensland
Psilogramma papuensis|GWOR1824-07|BC ZSM Lep 02388|605[0n]bp|Australia.Queensland
Psilogramma papuensis|GWOR1825-07|BC ZSM Lep 02389|605[0n]bp|Australia.Queensland
Psilogramma papuensis|SPTMA303-07|BC-Mel 0381|658[0n]bp|Australia.Queensland
Psilogramma papuensis|SPTMA297-07|BC-Mel 0375|658[0n]bp|Australia.Queensland
Psilogramma papuensis|SPTMA302-07|BC-Mel 0380|658[0n]bp|Australia.Queensland
Cerberonoton rubescens severina|ANICC081-08|ANIC Gen No. 003244|658[0n]bp|Australia.Queensland
Cerberonoton rubescens severina|SPTOL254-09|JP-05-0760|658[0n]bp|Australia.Queensland
Cerberonoton rubescens severina|ANICC011-08|ANIC Gen No. 003174|658[0n]bp|Australia.Queensland
Cerberonoton rubescens severina|GWORB866-07|BC ZSM Lep 01336|597[0n]bp|Australia.Queensland
Cerberonoton rubescens severina|ANICC003-08|ANIC Gen No. 003166|609[0n]bp|Australia.Queensland
Cerberonoton rubescens severina|ANICC038-08|ANIC Gen No. 003201|609[0n]bp|Australia.Queensland
Coequosa australasiae|SOWA609-06|BC-Hax0602|608[0n]bp|Australia.Northern Territory
Coequosa australasiae|SOWA608-06|BC-Hax0601|608[0n]bp|Australia.Queensland
Coequosa australasiae|ANICC018-08|ANIC Gen No. 003181|658[0n]bp|Australia.Queensland
Coequosa australasiae|IMLR1327-11|IM10-0071|658[0n]bp|Australia.Queensland
Coequosa australasiae|SPRBA754-09|BC-RBP-1797|658[0n]bp|Australia.Queensland
Coequosa australasiae|SPHYE141-09|BC-EST0517|632[0n]bp|Australia.New South Wales
Coequosa australasiae|ANICC189-08|BC-Roug1149|608[0n]bp|Australia.New South Wales
Coequosa australasiae|ANICC175-08|ANIC Gen No. 003338|658[0n]bp|Australia.New South Wales
Pseudoangonyx excellens|ANICC056-08|ANIC Gen No. 003219|553[2n]bp|Australia.Queensland

Coequosa australasiae|ANICC189-08|BC-Roug1149|608[0n]bp|Australia.New South Wales
Coequosa australasiae|ANICC175-08|ANIC Gen No. 003338|658[0n]bp|Australia.New South Wales
Pseudoangonyx excellens|ANICC056-08|ANIC Gen No. 003219|553[2n]bp|Australia.Queensland
Pseudoangonyx excellens|ANICC044-08|ANIC Gen No. 003207|658[3n]bp|Australia.Queensland
Theretra oldenlandiae lewini|GWORI499-09|BC ZSM Lep 13247|658[0n]bp|Australia.Northern Territory
Theretra oldenlandiae lewini|GWORD054-07|BC ZSM Lep 02686|658[0n]bp|Australia.Queensland
Theretra oldenlandiae lewini|GWORB865-07|BC ZSM Lep 01335|658[0n]bp|Australia.Queensland
Theretra oldenlandiae lewini|LOQTC768-08|gvc8413-1L|658[1n]bp|Australia.Queensland
Theretra oldenlandiae lewini|GWORB3268-08|BC ZSM Lep 05806|658[0n]bp|Australia.Queensland
Theretra oldenlandiae lewini|LOQT800-07|gvc6507-1L|658[0n]bp|Australia.Queensland
Theretra oldenlandiae lewini|NSWHJ040-10|09-NSWHH-0053|658[0n]bp|Australia.New South Wales
Theretra oldenlandiae lewini|LNSWE121-06|06-NSWE-00121|584[0n]bp|Australia.New South Wales
Theretra oldenlandiae lewini|SOWE243-07|BC-Hax4142|407[0n]bp|Australia
Theretra oldenlandiae lewini|LNSWE011-06|06-NSWE-00011|613[0n]bp|Australia.New South Wales
Theretra oldenlandiae lewini|GWORD057-07|BC ZSM Lep 02689|658[0n]bp|Australia.Queensland
Theretra oldenlandiae lewini|LNSWE125-06|06-NSWE-00125|608[0n]bp|Australia.New South Wales
Theretra oldenlandiae lewini|LOQ346-04|04HBL004346|658[0n]bp|Australia.Queensland
Theretra oldenlandiae lewini|LNSWE091-06|06-NSWE-00091|658[0n]bp|Australia.New South Wales
Theretra oldenlandiae lewini|GWORD059-07|BC ZSM Lep 02691|658[0n]bp|Australia.Queensland
Theretra oldenlandiae lewini|NSWHJ014-10|09-NSWHH-0027|658[0n]bp|Australia.New South Wales
Theretra oldenlandiae lewini|GWORD066-07|BC ZSM Lep 02698|658[0n]bp|Australia.Queensland
Theretra oldenlandiae lewini|GWORD068-07|BC ZSM Lep 02700|650[2n]bp|Australia.Queensland
Theretra oldenlandiae lewini|LNSWE129-06|06-NSWE-00129|658[0n]bp|Australia.New South Wales
Theretra oldenlandiae lewini|LOQT453-06|gvc6234-1L|658[0n]bp|Australia.Queensland
Theretra oldenlandiae lewini|GWORD056-07|BC ZSM Lep 02688|655[0n]bp|Australia.Queensland
Theretra oldenlandiae lewini|SOWE242-07|BC-Hax4141|647[0n]bp|Australia
Theretra oldenlandiae lewini|LOQTE588-10|gvc13322-1L|658[0n]bp|Australia.Queensland
Theretra oldenlandiae lewini|GWORB3243-08|BC ZSM Lep 05781|658[0n]bp|Australia.Queensland
Theretra oldenlandiae lewini|LOQ345-04|04HBL004345|658[0n]bp|Australia.Queensland
Theretra oldenlandiae lewini|GWORD067-07|BC ZSM Lep 02699|658[0n]bp|Australia.Queensland
Theretra oldenlandiae lewini|LOQ349-04|04HBL004349|658[0n]bp|Australia.Queensland
Theretra oldenlandiae lewini|GWORI548-09|BC ZSM Lep 13296|658[0n]bp|Australia.Northern Territory
Theretra oldenlandiae lewini|GWORG562-08|BC ZSM Lep 15002|658[2n]bp|Australia.Queensland
Theretra oldenlandiae lewini|GWORB2215-08|BC ZSM Lep 11015|640[0n]bp|Australia.Northern Territory
Theretra oldenlandiae lewini|LNSWE082-06|06-NSWE-00082|511[0n]bp|Australia.New South Wales
Theretra oldenlandiae lewini|NSWHM018-11|BIOUG00851-G11|658[0n]bp|Australia.New South Wales
Theretra oldenlandiae lewini|LOQTC417-07|gvc8053-1L|658[0n]bp|Australia.Queensland
Theretra oldenlandiae lewini|GWORD062-07|BC ZSM Lep 02694|658[0n]bp|Australia.Queensland
Theretra oldenlandiae lewini|LNSWE006-06|06-NSWE-00006|609[0n]bp|Australia.New South Wales
Theretra oldenlandiae lewini|GWORI543-09|BC ZSM Lep 13291|658[0n]bp|Australia.Northern Territory
Theretra oldenlandiae lewini|GWORD065-07|BC ZSM Lep 02697|609[0n]bp|Australia.Queensland
Theretra oldenlandiae lewini|LNSWE109-06|06-NSWE-00109|593[0n]bp|Australia.New South Wales
Theretra oldenlandiae lewini|LNSWE107-06|06-NSWE-00107|593[0n]bp|Australia.New South Wales
Theretra oldenlandiae lewini|LNSWE118-06|06-NSWE-00118|583[0n]bp|Australia.New South Wales
Theretra oldenlandiae lewini|LNSWE130-06|06-NSWE-00130|658[0n]bp|Australia.New South Wales
Theretra oldenlandiae lewini|LNSWE032-06|06-NSWE-00032|608[0n]bp|Australia.New South Wales
Theretra oldenlandiae lewini|GWORA139-08|BC ZSM Lep 10511|602[0n]bp|Australia.Western Australia
Theretra oldenlandiae lewini|LNSWE101-06|06-NSWE-00101|599[0n]bp|Australia.New South Wales
Theretra oldenlandiae lewini|LNSWE132-06|06-NSWE-00132|573[0n]bp|Australia.New South Wales
Theretra oldenlandiae lewini|LOQT1052-07|gvc6754-1L|658[0n]bp|Australia.Queensland
Theretra oldenlandiae lewini|LOQB591-05|Moth 070.03CL|574[0n]bp|Australia.Queensland
Theretra oldenlandiae lewini|GWORD064-07|BC ZSM Lep 02696|609[0n]bp|Australia.Queensland
Theretra oldenlandiae lewini|GWORB2190-08|BC ZSM Lep 10990|657[0n]bp|Australia.Northern Territory
Theretra oldenlandiae lewini|GWORD052-07|BC ZSM Lep 02684|609[0n]bp|Australia.Queensland
Theretra oldenlandiae lewini|LOQT626-06|gvc5640-1L|656[0n]bp|Australia.Queensland
Theretra oldenlandiae lewini|NSWHM032-11|BIOUG00851-F05|658[0n]bp|Australia.New South Wales
Theretra oldenlandiae lewini|NSWHM455-11|BIOUG00912-G04|658[0n]bp|Australia.New South Wales
Theretra oldenlandiae lewini|GWORD063-07|BC ZSM Lep 02695|609[0n]bp|Australia.Queensland
Theretra oldenlandiae lewini|LNSWE098-06|06-NSWE-00098|658[1n]bp|Australia.New South Wales
Theretra oldenlandiae lewini|GWORH540-09|BC ZSM Lep 10254|658[0n]bp|Australia.Queensland
Theretra oldenlandiae lewini|GWORI495-09|BC ZSM Lep 13243|658[0n]bp|Australia.Northern Territory
Theretra oldenlandiae lewini|GWORI544-09|BC ZSM Lep 13292|658[0n]bp|Australia.Northern Territory
Theretra oldenlandiae lewini|LOQT023-06|2006-LOQT-023|587[0n]bp|Australia.Queensland
Theretra oldenlandiae lewini|LNSWE075-06|06-NSWE-00075|517[1n]bp|Australia.New South Wales
Theretra oldenlandiae lewini|LOQTE672-10|gvc13682-1L|614[0n]bp|Australia.Queensland
Theretra oldenlandiae lewini|LNSWE089-06|06-NSWE-00089|589[0n]bp|Australia.New South Wales
Theretra oldenlandiae lewini|LNSWE016-06|06-NSWE-00016|604[0n]bp|Australia.New South Wales
Theretra oldenlandiae lewini|LNSWE100-06|06-NSWE-00100|657[0n]bp|Australia.New South Wales
Theretra oldenlandiae lewini|GWORD055-07|BC ZSM Lep 02687|658[0n]bp|Australia.Queensland
Theretra oldenlandiae lewini|LOQTE772-10|gvc14063-1L|658[0n]bp|Australia.Queensland
Theretra oldenlandiae lewini|NSWHH054-09|08-NSWHH-0054|658[0n]bp|Australia.New South Wales
Theretra oldenlandiae lewini|LNSWE056-06|06-NSWE-00056|606[0n]bp|Australia.New South Wales
Theretra oldenlandiae lewini|GWORD058-07|BC ZSM Lep 02690|658[1n]bp|Australia.Queensland
Theretra oldenlandiae lewini|LNSWE007-06|06-NSWE-00007|598[0n]bp|Australia.New South Wales
Theretra oldenlandiae lewini|LNSWE119-06|06-NSWE-00119|510[1n]bp|Australia.New South Wales
Theretra oldenlandiae lewini|LOQTB068-07|gvc6834-1L|597[0n]bp|Australia.Queensland
Theretra oldenlandiae lewini|GWORG563-08|BC ZSM Lep 15003|658[0n]bp|Australia.Queensland
Theretra oldenlandiae lewini|GWORI496-09|BC ZSM Lep 13244|658[0n]bp|Australia.Northern Territory
Theretra oldenlandiae lewini|LNSWE096-06|06-NSWE-00096|658[0n]bp|Australia.New South Wales
Theretra oldenlandiae lewini|LOQTD848-08|gvc10517-1L|658[0n]bp|Australia.Queensland
Theretra oldenlandiae lewini|GWORI506-09|BC ZSM Lep 13254|658[0n]bp|Australia.Northern Territory
Theretra oldenlandiae lewini|LOQB571-05|Moth 050.03CL|658[2n]bp|Australia.Queensland
Theretra oldenlandiae lewini|GWORD053-07|BC ZSM Lep 02685|658[0n]bp|Australia.Queensland
Theretra oldenlandiae lewini|GWORB2197-08|BC ZSM Lep 10997|658[0n]bp|Australia.Northern Territory
Theretra oldenlandiae lewini|NSWHM2156-11|BIOUG00961-F07|658[0n]bp|Australia.New South Wales
Theretra turneri|SOWD644-06|BC-Hax3543|658[0n]bp|Australia
Theretra turneri|GWORD045-07|BC ZSM Lep 02677|658[0n]bp|Australia.Queensland
Theretra turneri|ANICC033-08|ANIC Gen No. 003196|658[0n]bp|Australia.Queensland
Theretra turneri|GWORD044-07|BC ZSM Lep 02676|658[0n]bp|Australia.Queensland
Theretra turneri|LOQT839-07|gvc6555-1L|643[0n]bp|Australia.Queensland
Theretra turneri|GWORC142-07|BC ZSM Lep 02492|655[0n]bp|Australia.Queensland
Theretra turneri|SPTMC489-12|BC-Mel2490|658[0n]bp|Australia.Queensland
Theretra margarita|GWORB2256-08|BC ZSM Lep 11056|609[0n]bp|Australia.Northern Territory
Theretra margarita|GWORD050-07|BC ZSM Lep 02682|572[0n]bp|Australia.Queensland
Theretra margarita|GWORA186-08|BC ZSM Lep 10558|649[1n]bp|Australia.Western Australia
Theretra margarita|LOQTB046-07|gvc6812-1L|643[1n]bp|Australia.Queensland
Theretra margarita|GWORA099-08|BC ZSM Lep 10471|558[1n]bp|Australia.Northern Territory
Theretra margarita|LOQT807-07|gvc6515-1L|658[0n]bp|Australia.Queensland
Theretra margarita|LOQTE720-10|gvc13863-1L|658[6n]bp|Australia.Queensland

Theretra margarita|GWORA099-08|BC ZSM Lep 10471|558[1n]bp|Australia.Northern Territory
Theretra margarita|LOQT807-07|gvc6515-1L|658[0n]bp|Australia.Queensland
Theretra margarita|LOQTE720-10|gvc13863-1L|658[6n]bp|Australia.Queensland
Theretra margarita|LOQTI518-11|gvc17066-1L|658[0n]bp|Australia.Queensland
Theretra margarita|LOQT210-06|2006-LOQT-210|632[0n]bp|Australia.Queensland
Theretra margarita|GWORN236-09|BC ZSM Lep 18264|658[0n]bp|Australia.Western Australia
Theretra margarita|LOQTI516-11|gvc17064-1L|658[0n]bp|Australia.Queensland
Theretra margarita|GWORA162-08|BC ZSM Lep 10534|615[0n]bp|Australia.Western Australia
Theretra margarita|GWORA182-08|BC ZSM Lep 10554|649[0n]bp|Australia.Western Australia
Theretra margarita|GWORA187-08|BC ZSM Lep 10559|646[0n]bp|Australia.Western Australia
Theretra margarita|GWORA124-08|BC ZSM Lep 10496|646[0n]bp|Australia.Western Australia
Theretra margarita|GWORA188-08|BC ZSM Lep 10560|604[4n]bp|Australia.Western Australia
Theretra margarita|GWORC188-07|BC ZSM Lep 02538|630[0n]bp|Australia.Queensland
Theretra margarita|GWORA013-08|BC ZSM Lep 10385|656[0n]bp|Australia.Western Australia
Theretra margarita|LOQTB002-07|gvc6771-1L|658[0n]bp|Australia.Queensland
Theretra margarita|LOQT700-06|gvc6396-1L|658[0n]bp|Australia.Queensland
Theretra margarita|GWORA088-08|BC ZSM Lep 10460|658[0n]bp|Australia.Western Australia
Theretra margarita|GWORD061-07|BC ZSM Lep 02693|658[0n]bp|Australia.Queensland
Theretra margarita|GWORN237-09|BC ZSM Lep 18265|658[0n]bp|Australia.Western Australia
Theretra margarita|GWORN238-09|BC ZSM Lep 18266|646[0n]bp|Australia.Western Australia
Theretra margarita|GWORA137-08|BC ZSM Lep 10509|618[0n]bp|Australia.Western Australia
Theretra margarita|LOQTE688-10|gvc13805-1L|641[0n]bp|Australia.Queensland
Theretra margarita|LOQTI517-11|gvc17065-1L|658[0n]bp|Australia.Queensland
Theretra margarita|GWORD049-07|BC ZSM Lep 02681|658[0n]bp|Australia.Queensland
Theretra margarita|GWORN239-09|BC ZSM Lep 18267|658[0n]bp|Australia.Western Australia
Theretra margarita|GWORA138-08|BC ZSM Lep 10510|649[0n]bp|Australia.Western Australia
Theretra silhetensis intersecta|GWORI542-09|BC ZSM Lep 13290|637[0n]bp|Australia.Northern Territory
Theretra silhetensis intersecta|LOQTE109-09|gvc11552-1L|654[2n]bp|Australia.Queensland
Theretra silhetensis intersecta|LOQTE052-09|gvc11442-1L|658[1n]bp|Australia.Queensland
Theretra silhetensis intersecta|LOQTE824-10|gvc14263-1L|658[0n]bp|Australia.Queensland
Theretra silhetensis intersecta|LOQC038-05|05-QLD-00038|658[0n]bp|Australia.Queensland
Theretra silhetensis intersecta|IMLR228-08|IM07-1133|658[0n]bp|Australia.Queensland
Theretra silhetensis intersecta|ANICC002-08|ANIC Gen No. 003165|658[0n]bp|Australia.Northern Terr...
Theretra silhetensis intersecta|IMLR1334-11|IM10-0094|658[0n]bp|Australia.Queensland
Theretra silhetensis intersecta|ANICC001-08|ANIC Gen No. 003164|658[0n]bp|Australia.Northern Terr...
Theretra silhetensis intersecta|GWORY458-10|BC EF Lep 03412|658[0n]bp|Australia.Queensland
Theretra silhetensis intersecta|LOQTE889-10|gvc14423-1L|658[0n]bp|Australia.Queensland
Theretra silhetensis intersecta|IMLR899-11|IM07-0028|658[0n]bp|Australia.Queensland
Theretra silhetensis intersecta|GWORI502-09|BC ZSM Lep 13250|658[0n]bp|Australia.Northern Territory
Theretra silhetensis intersecta|IMLR1332-11|IM10-0090|658[0n]bp|Australia.Queensland
Theretra silhetensis intersecta|GWORY459-10|BC EF Lep 03413|658[0n]bp|Australia.Northern Territory
Theretra silhetensis intersecta|GWORI551-09|BC ZSM Lep 13299|658[0n]bp|Australia.Northern Territory
Theretra silhetensis intersecta|SOWD629-06|BC-Hax3528|407[0n]bp|Australia.Queensland
Theretra silhetensis intersecta|ANICC009-08|ANIC Gen No. 003172|598[0n]bp|Australia.Western Austr...
Theretra silhetensis intersecta|LOQTE785-10|gvc14162-1L|658[0n]bp|Australia.Queensland
Theretra silhetensis intersecta|IMLQ900-08|IM08-0248|658[0n]bp|Australia.Queensland
Hyles livornicoides|GWORA177-08|BC ZSM Lep 10549|625[0n]bp|Australia.Western Australia
Hyles livornicoides|LNSWC315-06|06-NSW-00315|658[0n]bp|Australia.New South Wales
Hyles livornicoides|AMWW210-12|K292495|658[0n]bp|Australia.New South Wales
Hyles livornicoides|ANICB365-06|ANIC Gen No. 000212|658[0n]bp|Australia.Queensland
Hyles livornicoides|LNSWB104-05|05-NSW-01044|658[0n]bp|Australia.New South Wales
Hyles livornicoides|LOQTC660-08|gvc8302-1L|658[0n]bp|Australia.Queensland
Hyles livornicoides|LNSWB108-05|05-NSW-01048|608[0n]bp|Australia.New South Wales
Hyles livornicoides|LNSWB105-05|05-NSW-01045|658[0n]bp|Australia.New South Wales
Hyles livornicoides|LOQT840-07|gvc6556-1L|621[0n]bp|Australia.Queensland
Hyles livornicoides|LOQT212-06|2006-LOQT-212|658[0n]bp|Australia.Queensland
Hyles livornicoides|SPUEB391-07|BC-EMEM1331|658[0n]bp|Australia.Queensland
Hyles livornicoides|GWORN201-09|BC ZSM Lep 18229|658[0n]bp|Australia.Western Australia
Hyles livornicoides|LOQT005-06|2006-LOQT-005|657[0n]bp|Australia.Queensland
Hyles livornicoides|LOQTB047-07|gvc6813-1L|646[0n]bp|Australia.Queensland
Hyles livornicoides|AMWW212-12|K292497|658[0n]bp|Australia.New South Wales
Hyles livornicoides|AMWW211-12|K292496|658[0n]bp|Australia.New South Wales
Hyles livornicoides|LOQTC659-08|gvc8301-1L|658[0n]bp|Australia.Queensland
Hyles livornicoides|AMWW213-12|K292498|658[0n]bp|Australia.New South Wales
Hyles livornicoides|GWORO526-09|BC ZSM Lep 26322|658[0n]bp|Australia.Western Australia
Hyles livornicoides|LOQT408-06|gvc5635-1L|658[0n]bp|Australia.Queensland
Hyles livornicoides|LNSWB106-05|05-NSW-01046|658[0n]bp|Australia.New South Wales
Hyles livornicoides|AMWW011-11|K290751|658[0n]bp|Australia.New South Wales
Hyles livornicoides|LOQTC628-07|gvc8269-1L|658[0n]bp|Australia.Queensland
Hyles livornicoides|LOQT940-07|gvc6650-1L|658[0n]bp|Australia.Queensland
Hyles livornicoides|LOQTI651-12|gvc2677-1L|658[0n]bp|Australia.Queensland
Hyles livornicoides|AMWW214-12|K292499|658[0n]bp|Australia.New South Wales
Hyles livornicoides|AMWW039-11|K290779|658[0n]bp|Australia.New South Wales
Hyles livornicoides|LOQTB048-07|gvc6814-1L|658[0n]bp|Australia.Queensland
Hyles livornicoides|LNSWB107-05|05-NSW-01047|658[0n]bp|Australia.New South Wales
Hyles livornicoides|AMWW208-12|K292493|658[0n]bp|Australia.New South Wales
Hyles livornicoides|AMWW209-12|K292494|658[0n]bp|Australia.New South Wales
Hyles livornicoides|LOQTB085-07|gvc6846-1L|658[0n]bp|Australia.Queensland
Hyles livornicoides|LOQTC767-08|gvc8412-1L|658[0n]bp|Australia.Queensland
Hyles livornicoides|GWORN195-09|BC ZSM Lep 18223|658[0n]bp|Australia.Western Australia
Hyles livornicoides|LOQT841-07|gvc6557-1L|656[0n]bp|Australia.Queensland
Hyles livornicoides|GWORA176-08|BC ZSM Lep 10548|552[0n]bp|Australia.Western Australia
Hyles livornicoides|LNSWB103-05|05-NSW-01043|614[0n]bp|Australia.New South Wales
Hyles livornicoides|GWORA168-08|BC ZSM Lep 10540|547[0n]bp|Australia.Western Australia
Hyles livornicoides|AMWW170-12|K287974|658[0n]bp|Australia.New South Wales
Theretra nessus|GWORB884-07|BC ZSM Lep 01354|632[0n]bp|Australia.Queensland
Theretra nessus|GWORB882-07|BC ZSM Lep 01352|632[0n]bp|Australia.Queensland
Theretra nessus|GWORB872-07|BC ZSM Lep 01342|632[0n]bp|Australia.Queensland
Theretra nessus|GWORG359-08|BC ZSM Lep 08475|658[0n]bp|Australia.Queensland
Theretra nessus|NSWHM2152-11|BIOUG00961-F03|658[0n]bp|Australia.New South Wales
Theretra nessus|NSWHM001-11|BIOUG00851-F06|658[0n]bp|Australia.New South Wales
Theretra nessus|NSWHM015-11|BIOUG00851-G08|658[0n]bp|Australia.New South Wales
Theretra nessus|NSWHM013-11|BIOUG00851-G06|658[0n]bp|Australia.New South Wales
Theretra nessus|LNSWE003-06|06-NSWE-00003|658[0n]bp|Australia.New South Wales
Theretra nessus|NSWHM2019-11|BIOUG00961-A02|658[1n]bp|Australia.New South Wales
Theretra nessus|LNSWE088-06|06-NSWE-00088|541[0n]bp|Australia.New South Wales
Theretra nessus|LNSWE092-06|06-NSWE-00092|584[0n]bp|Australia.New South Wales
Theretra nessus|LNSWE045-06|06-NSWE-00045|591[0n]bp|Australia.New South Wales
Theretra nessus|NSWHM2159-11|BIOUG00961-F10|658[0n]bp|Australia.New South Wales

Theretra nessus|LNSWE092-06|06-NSWE-00092|584[0n]bp|Australia.New South Wales
Theretra nessus|LNSWE045-06|06-NSWE-00045|591[0n]bp|Australia.New South Wales
Theretra nessus|NSWHM2159-11|BIOUG00961-F10|658[0n]bp|Australia.New South Wales
Theretra nessus|LNSWE069-06|06-NSWE-00069|658[0n]bp|Australia.New South Wales
Theretra nessus|GWORC241-07|BC ZSM Lep 02591|658[0n]bp|Australia.Queensland
Theretra latreillii|SOWC868-06|BC-Hax2767|494[0n]bp|Australia.Queensland
Theretra latreillii|GWORB857-07|BC ZSM Lep 01327|627[0n]bp|Australia.Queensland
Theretra latreillii|GWORB875-07|BC ZSM Lep 01345|627[0n]bp|Australia.Queensland
Theretra latreillii|GWORB898-07|BC ZSM Lep 01368|627[0n]bp|Australia.Queensland
Theretra latreillii|GWORI507-09|BC ZSM Lep 13255|627[0n]bp|Australia.Northern Territory
Theretra latreillii|GWORB876-07|BC ZSM Lep 01346|621[0n]bp|Australia.Queensland
Theretra latreillii|GWORB888-07|BC ZSM Lep 01358|621[0n]bp|Australia.Queensland
Theretra latreillii|GWORB860-07|BC ZSM Lep 01330|621[0n]bp|Australia.Queensland
Theretra latreillii|GWORB891-07|BC ZSM Lep 01361|621[0n]bp|Australia.Queensland
Theretra latreillii|GWORB890-07|BC ZSM Lep 01360|621[0n]bp|Australia.Queensland
Theretra latreillii|LOQTD909-09|gvc11071-1L|658[0n]bp|Australia.Queensland
Theretra latreillii|GWORG484-08|BC ZSM Lep 14924|658[0n]bp|Australia.Queensland
Theretra latreillii|LOQTD889-09|gvc11028-1L|658[0n]bp|Australia.Queensland
Theretra latreillii|GWOR1229-07|BC ZSM Lep 01981|657[0n]bp|Australia.Queensland
Theretra latreillii|IMLR890-11|IM06-0552|658[0n]bp|Australia.Queensland
Theretra latreillii|GWOR1231-07|BC ZSM Lep 01983|658[0n]bp|Australia.Queensland
Theretra latreillii|NSWHJ026-10|09-NSWHH-0039|658[0n]bp|Australia.New South Wales
Theretra latreillii|LOQT712-06|gvc6424-1L|658[0n]bp|Australia.Queensland
Theretra latreillii|LOQTB049-07|gvc6825-1L|658[0n]bp|Australia.Queensland
Theretra latreillii|GWOR1695-07|BC ZSM Lep 02259|658[0n]bp|Australia.Queensland
Theretra latreillii|GWOR1236-07|BC ZSM Lep 01988|658[0n]bp|Australia.Queensland
Theretra latreillii|NSWHJ064-10|09-NSWHH-0077|658[0n]bp|Australia.New South Wales
Theretra latreillii|ANIC347-06|ANIC Gen No. 000539|658[0n]bp|Australia.Queensland
Theretra latreillii|LOQTB024-07|gvc6798-1L|658[0n]bp|Australia.Queensland
Theretra latreillii|GWORG360-08|BC ZSM Lep 08476|658[0n]bp|Australia.Queensland
Theretra latreillii|GWOR1238-07|BC ZSM Lep 01990|658[0n]bp|Australia.Queensland
Theretra latreillii|NSWHJ038-10|09-NSWHH-0051|658[0n]bp|Australia.New South Wales
Theretra latreillii|IMLQ230-07|IM07-0146|658[0n]bp|Australia.Queensland
Theretra latreillii|GWORB3270-08|BC ZSM Lep 05808|658[0n]bp|Australia.Queensland
Theretra latreillii|GWORB3269-08|BC ZSM Lep 05807|658[0n]bp|Australia.Queensland
Theretra latreillii|GWOR1232-07|BC ZSM Lep 01984|658[0n]bp|Australia.Queensland
Theretra latreillii|IMLR1085-11|IM08-2609|658[0n]bp|Australia.Queensland
Theretra latreillii|NSWHJ074-10|09-NSWHH-0087|658[0n]bp|Australia.New South Wales
Theretra latreillii|GWORB3267-08|BC ZSM Lep 05805|658[0n]bp|Australia.Queensland
Theretra latreillii|NSWHJ028-10|09-NSWHH-0041|658[0n]bp|Australia.New South Wales
Theretra latreillii|LOQTB139-07|gvc6902-1L|658[0n]bp|Australia.Queensland
Theretra latreillii|LOQTC919-08|gvc8566-1L|658[0n]bp|Australia.Queensland
Theretra latreillii|GWORG303-08|BC ZSM Lep 08419|658[0n]bp|Australia.Queensland
Theretra latreillii|GWOR1241-07|BC ZSM Lep 01993|658[0n]bp|Australia.Queensland
Theretra latreillii|NSWHJ086-10|09-NSWHH-0099|658[0n]bp|Australia.New South Wales
Theretra latreillii|GWORB856-07|BC ZSM Lep 01326|614[0n]bp|Australia.Queensland
Theretra latreillii|GWORB858-07|BC ZSM Lep 01328|632[0n]bp|Australia.Queensland
Theretra latreillii|GWORB859-07|BC ZSM Lep 01329|632[0n]bp|Australia.Queensland
Theretra latreillii|GWORC234-07|BC ZSM Lep 02584|653[0n]bp|Australia.Queensland
Theretra latreillii|GWORB861-07|BC ZSM Lep 01331|632[0n]bp|Australia.Queensland
Theretra latreillii|GWOR1694-07|BC ZSM Lep 02258|644[0n]bp|Australia.Queensland
Theretra latreillii|GWORD040-07|BC ZSM Lep 02672|658[0n]bp|Australia.Queensland
Theretra latreillii|GWORB874-07|BC ZSM Lep 01344|607[0n]bp|Australia.Queensland
Theretra latreillii|NSWHJ062-10|09-NSWHH-0075|622[0n]bp|Australia.New South Wales
Theretra latreillii|GWOR1838-07|BC ZSM Lep 02402|582[0n]bp|Australia.Queensland
Theretra latreillii|GWORA136-08|BC ZSM Lep 10508|646[1n]bp|Australia.Western Australia
Theretra latreillii|LNSWE001-06|06-NSWE-00001|532[0n]bp|Australia.New South Wales
Theretra latreillii|LOQB003-05|Moth 003.01LZ|575[0n]bp|Australia.Queensland
Theretra latreillii|LOQB004-05|Moth 004.01LZ|575[0n]bp|Australia.Queensland
Theretra latreillii|GWORC233-07|BC ZSM Lep 02583|622[0n]bp|Australia.Queensland
Theretra latreillii|NSWHJ016-10|09-NSWHH-0029|658[0n]bp|Australia.New South Wales
Theretra inornata|GWORI474-09|BC ZSM Lep 13222|658[0n]bp|Australia.Northern Territory
Theretra inornata|GWOR1248-07|BC ZSM Lep 02000|656[0n]bp|Australia.Queensland
Theretra inornata|GWOR1249-07|BC ZSM Lep 02001|658[0n]bp|Australia.Queensland
Theretra inornata|GWORI473-09|BC ZSM Lep 13221|658[0n]bp|Australia.Northern Territory
Theretra inornata|GWORI476-09|BC ZSM Lep 13224|658[0n]bp|Australia.Northern Territory
Theretra inornata|GWORI478-09|BC ZSM Lep 13226|621[0n]bp|Australia.Northern Territory
Theretra inornata|GWORI482-09|BC ZSM Lep 13230|658[0n]bp|Australia.Northern Territory
Theretra inornata|GWORI479-09|BC ZSM Lep 13227|658[0n]bp|Australia.Northern Territory
Theretra inornata|GWORI475-09|BC ZSM Lep 13223|577[0n]bp|Australia.Northern Territory
Theretra inornata|GWORI477-09|BC ZSM Lep 13225|658[0n]bp|Australia.Northern Territory
Theretra inornata|GWORI481-09|BC ZSM Lep 13229|658[0n]bp|Australia.Northern Territory
Theretra inornata|GWORI480-09|BC ZSM Lep 13228|658[0n]bp|Australia.Northern Territory
Theretra celata|SOWC844-06|BC-Hax2743|513[0n]bp|Australia.Queensland
Theretra celata|GWORB855-07|BC ZSM Lep 01325|622[0n]bp|Australia.Queensland
Theretra celata|GWORB3272-08|BC ZSM Lep 05810|658[0n]bp|Australia.Queensland
Theretra celata|GWOR1239-07|BC ZSM Lep 01991|658[0n]bp|Australia.Queensland
Theretra celata|NSWHJ004-10|09-NSWHH-0017|644[0n]bp|Australia.New South Wales
Theretra celata|GWORB3230-08|BC ZSM Lep 05768|658[0n]bp|Australia.Queensland
Theretra celata|ANICC005-08|ANIC Gen No. 003168|609[0n]bp|Australia.Queensland
Theretra celata|ANICC004-08|ANIC Gen No. 003167|658[0n]bp|Australia.Queensland
Theretra celata|GWORD039-07|BC ZSM Lep 02671|658[0n]bp|Australia.Queensland
Theretra celata|GWORB3271-08|BC ZSM Lep 05809|658[0n]bp|Australia.Queensland
Theretra celata|LOQ342-04|04HBL004342|658[0n]bp|Australia.Queensland
Theretra celata|LOQT217-06|2006-LOQT-217|642[0n]bp|Australia.Queensland
Theretra celata|LNSWE015-06|06-NSWE-00015|656[0n]bp|Australia.New South Wales
Theretra celata|ANICC007-08|ANIC Gen No. 003170|636[0n]bp|Australia.Queensland
Theretra celata|LOQ343-04|04HBL004343|584[0n]bp|Australia.Queensland
Theretra celata|GWORD046-07|BC ZSM Lep 02678|658[0n]bp|Australia.Queensland
Theretra celata|ANICC006-08|ANIC Gen No. 003169|598[3n]bp|Australia.Queensland
Theretra indistincta|SOWC850-06|BC-Hax2749|608[0n]bp|Australia.Queensland
Theretra indistincta|GWORB887-07|BC ZSM Lep 01357|632[0n]bp|Australia.Queensland
Theretra indistincta|SPTMB246-10|BC-Mel1248|658[0n]bp|Australia.Queensland
Theretra indistincta|GWORA263-08|BC ZSM Lep 10635|658[0n]bp|Australia.Northern Territory
Theretra indistincta|LNSWE027-06|06-NSWE-00027|553[1n]bp|Australia.New South Wales
Theretra indistincta|GWOR1837-07|BC ZSM Lep 02401|572[0n]bp|Australia.Queensland
Theretra indistincta|LOQ344-04|04HBL004344|603[1n]bp|Australia.Queensland
Theretra indistincta|LNSWE014-06|06-NSWE-00014|575[0n]bp|Australia.New South Wales
Theretra indistincta|NSWHJ050-10|09-NSWHH-0063|658[0n]bp|Australia.New South Wales

Theretra indistincta|LOQ344-04|04HBL004344|603[1n]bp|Australia.Queensland
Theretra indistincta|LNSWE014-06|06-NSWE-00014|575[0n]bp|Australia.New South Wales
Theretra indistincta|NSWHJ050-10|09-NSWHH-0063|658[0n]bp|Australia.New South Wales
Theretra indistincta|SPTMB248-10|BC-Mel1250|658[0n]bp|Australia.Northern Territory
Theretra indistincta|SPTMB247-10|BC-Mel1249|658[0n]bp|Australia.Northern Territory
Theretra indistincta|GWORD043-07|BC ZSM Lep 02675|658[0n]bp|Australia.Queensland
Theretra tryoni|NSWBB1250-08|07-NSWBB-1250|658[0n]bp|Australia.New South Wales
Theretra tryoni|GWOR1237-07|BC ZSM Lep 01989|658[0n]bp|Australia.Queensland
Theretra tryoni|GWOR1235-07|BC ZSM Lep 01987|645[0n]bp|Australia.Queensland
Theretra tryoni|GWOR1233-07|BC ZSM Lep 01985|658[0n]bp|Australia.Queensland
Theretra tryoni|GWORG483-08|BC ZSM Lep 14923|407[0n]bp|Australia.Queensland
Theretra queenslandi|LNSWE013-06|06-NSWE-00013|658[0n]bp|Australia.New South Wales
Theretra queenslandi|ANICH131-10|10ANIC-04128|609[0n]bp|Australia.Queensland
Theretra queenslandi|SPTVA374-07|VAG-2185|609[0n]bp|Australia.New South Wales
Theretra queenslandi|ANICH128-10|10ANIC-04125|609[0n]bp|Australia.Queensland
Theretra queenslandi|ANICH130-10|10ANIC-04127|658[0n]bp|Australia.Queensland
Theretra queenslandi|LOQT207-06|2006-LOQT-207|658[0n]bp|Australia.Queensland
Theretra queenslandi|LNSWE012-06|06-NSWE-00012|658[0n]bp|Australia.New South Wales
Theretra queenslandi|SPTMB239-10|BC-Mel1241|658[0n]bp|Australia.Queensland
Theretra queenslandi|ANICC041-08|ANIC Gen No. 003204|658[0n]bp|Australia.Queensland
Theretra queenslandi|ANICH129-10|10ANIC-04126|658[0n]bp|Australia.Queensland
Theretra queenslandi|ANICC042-08|ANIC Gen No. 003205|625[0n]bp|Australia.Queensland
Theretra queenslandi|LNSWE080-06|06-NSWE-00080|557[1n]bp|Australia.New South Wales
Theretra queenslandi|LNSWE099-06|06-NSWE-00099|515[0n]bp|Australia.New South Wales
Theretra queenslandi|ANICC091-08|ANIC Gen No. 003254|577[0n]bp|Australia.New South Wales
Theretra queenslandi|LNSWE115-06|06-NSWE-00115|658[0n]bp|Australia.New South Wales
Macroglossum troglodytus|ANICC049-08|ANIC Gen No. 003212|294[1n]bp|Australia.Queensland
Macroglossum alcedo|SPHJT008-09|BC-JT-23M|658[0n]bp|Australia.Queensland
Macroglossum alcedo|SPHJT097-10|BC-LTM-121|658[0n]bp|Australia.Queensland
Eupanacra splendens|LOQTI673-12|gvc17580-1L|638[0n]bp|Australia.Queensland
Eupanacra splendens|LOQTC288-07|gvc7924-1L|659[0n]bp|Australia.Queensland
Eupanacra splendens|LTOL253-07|JP-05-0766|658[0n]bp|Australia.Queensland
Eupanacra splendens|LOQTI136-10|gvc15252-1L|658[0n]bp|Australia.Queensland
Eupanacra splendens|LOQTD377-08|gvc8982-1L|658[0n]bp|Australia.Queensland
Eupanacra splendens|GWOR1240-07|BC ZSM Lep 01992|657[0n]bp|Australia.Queensland
Eupanacra splendens|GWORB3275-08|BC ZSM Lep 05813|658[0n]bp|Australia.Queensland
Eupanacra splendens|LOQTE774-10|gvc14103-1L|658[0n]bp|Australia.Queensland
Eupanacra splendens|LOQTD126-08|gvc8725-1L|658[0n]bp|Australia.Queensland
Eupanacra splendens|LOQT004-06|2006-LOQT-004|658[0n]bp|Australia.Queensland
Eupanacra splendens|SOWB487-06|BC-Hax1480|608[0n]bp|Australia.Queensland
Eupanacra splendens|GWORB893-07|BC ZSM Lep 01363|632[0n]bp|Australia.Queensland
Eupanacra splendens|GWOR1244-07|BC ZSM Lep 01996|658[0n]bp|Australia.Queensland
Eupanacra splendens|GWORB3274-08|BC ZSM Lep 05812|658[0n]bp|Australia.Queensland
Eupanacra splendens|GWORB3276-08|BC ZSM Lep 05814|658[0n]bp|Australia.Queensland
Gnathothlibus australiensis|SPHJT103-10|BC-LTM-127|658[0n]bp|Australia.Queensland
Gnathothlibus australiensis|SPHJT102-10|BC-LTM-126|658[0n]bp|Australia.Queensland
Gnathothlibus australiensis|SPHJT104-10|BC-LTM-128|658[0n]bp|Australia.Queensland
Gnathothlibus australiensis|ANICC083-08|ANIC Gen No. 003246|609[0n]bp|Australia.Queensland
Gnathothlibus australiensis|SPHJT101-10|BC-LTM-125|658[0n]bp|Australia.Queensland
Gnathothlibus eras|SPHJT105-10|BC-LTM-129|606[1n]bp|Australia.Queensland
Gnathothlibus eras|LOQB001-05|Moth 001.01LZ|658[0n]bp|Australia.Queensland
Gnathothlibus eras|SPHJT004-09|BC-JT-22M|658[0n]bp|Australia.Queensland
Gnathothlibus eras|SPHJT108-10|BC-LTM-132|658[0n]bp|Australia.Queensland
Gnathothlibus eras|LOQTE814-10|gvc14230-1L|658[0n]bp|Australia.Queensland
Gnathothlibus eras|LOQTB079-07|gvc6840-1L|658[0n]bp|Australia.Queensland
Gnathothlibus eras|SPHJT110-10|BC-LTM-134|658[0n]bp|Australia.Queensland
Gnathothlibus eras|LOQB299-05|Moth 299.01LZ|574[0n]bp|Australia.Queensland
Gnathothlibus eras|GWOR1827-07|BC ZSM Lep 02391|600[0n]bp|Australia.Queensland
Gnathothlibus eras|LOQTD850-09|gvc10628-1L|658[0n]bp|Australia.Queensland
Gnathothlibus eras|SPHJT107-10|BC-LTM-131|658[0n]bp|Australia.Queensland
Gnathothlibus eras|SPHJT009-09|BC-JT-21M|658[0n]bp|Australia.Queensland
Gnathothlibus eras|ANICC090-08|ANIC Gen No. 003253|658[0n]bp|Australia.Northern Territory
Gnathothlibus eras|LOQTB140-07|gvc6904-1L|658[0n]bp|Australia.Queensland
Gnathothlibus eras|LOQB298-05|Moth 298.01LZ|569[0n]bp|Australia.Queensland
Gnathothlibus eras|ANICC088-08|ANIC Gen No. 003251|658[0n]bp|Australia.Queensland
Gnathothlibus eras|SOWE582-07|BC-Hax4481|658[0n]bp|Australia
Gnathothlibus eras|ANICC067-08|ANIC Gen No. 003230|658[0n]bp|Australia.Queensland
Gnathothlibus eras|LTOL251-07|JP-05-0761|658[0n]bp|Australia.Queensland
Gnathothlibus eras|ANICC089-08|ANIC Gen No. 003252|658[0n]bp|Australia.Northern Territory
Gnathothlibus eras|GWORD037-07|BC ZSM Lep 02669|658[0n]bp|Australia.Queensland
Gnathothlibus eras|LOQTB138-07|gvc6901-1L|658[0n]bp|Australia.Queensland
Gnathothlibus eras|GWORD038-07|BC ZSM Lep 02670|658[0n]bp|Australia.Queensland
Gnathothlibus eras|LOQTB141-07|gvc6905-1L|645[0n]bp|Australia.Queensland
Gnathothlibus eras|GWOR1833-07|BC ZSM Lep 02397|626[0n]bp|Australia.Queensland
Gnathothlibus eras|SPHJT106-10|BC-LTM-130|658[0n]bp|Australia.Queensland
Gnathothlibus eras|GWOR1834-07|BC ZSM Lep 02398|600[0n]bp|Australia.Queensland
Gnathothlibus eras|LOQTI636-12|gvc17314-1L|658[0n]bp|Australia.Queensland
Gnathothlibus eras|GWORD036-07|BC ZSM Lep 02668|658[0n]bp|Australia.Queensland
Gnathothlibus eras|SPHJT109-10|BC-LTM-133|658[0n]bp|Australia.Queensland
Hippotion velox|LOQTB799-07|gvc7494-1L|658[0n]bp|Australia.Queensland
Hippotion velox|ANIC315-06|ANIC Gen No. 000507|658[0n]bp|Australia.Queensland
Hippotion velox|LOQTC808-08|gvc8453-1L|658[0n]bp|Australia.Queensland
Hippotion velox|ANIC316-06|ANIC Gen No. 000508|658[0n]bp|Australia.Queensland
Hippotion velox|SPTMA866-09|BC-Mel 0944|649[0n]bp|Australia.Queensland
Hippotion velox|LOQB292-05|Moth 292.01LZ|575[0n]bp|Australia.Queensland
Hippotion velox|LOQB293-05|Moth 293.01LZ|617[0n]bp|Australia.Queensland
Hippotion celerio|PHLCC1153-11|BIOUG01235-B05|658[0n]bp|Australia.Australian Capital Territory
Hippotion celerio|LNSWE126-06|06-NSWE-00126|584[0n]bp|Australia.New South Wales
Hippotion celerio|LNSWE137-06|06-NSWE-00137|533[0n]bp|Australia.New South Wales
Hippotion celerio|LNSWE087-06|06-NSWE-00087|578[0n]bp|Australia.New South Wales
Hippotion celerio|LNSWE090-06|06-NSWE-00090|658[0n]bp|Australia.New South Wales
Hippotion celerio|LNSWE084-06|06-NSWE-00084|658[0n]bp|Australia.New South Wales
Hippotion celerio|LNSWE041-06|06-NSWE-00041|658[0n]bp|Australia.New South Wales
Hippotion celerio|LNSWE093-06|06-NSWE-00093|658[0n]bp|Australia.New South Wales
Hippotion celerio|LNSWB151-05|05-NSW-01091|658[0n]bp|Australia.New South Wales
Hippotion celerio|LNSWE128-06|06-NSWE-00128|658[0n]bp|Australia.New South Wales
Hippotion celerio|NSWHJ052-10|09-NSWHH-0065|658[0n]bp|Australia.New South Wales
Hippotion celerio|LNSWE052-06|06-NSWE-00052|658[0n]bp|Australia.New South Wales
Hippotion celerio|LSM1761-11|am10761|658[0n]bp|Australia.New South Wales

Hippotion celerio|NSWHJ052-10|09-NSWHH-0065|658[0n]bp|Australia.New South Wales
Hippotion celerio|LNSWE052-06|06-NSWE-00052|658[0n]bp|Australia.New South Wales
Hippotion celerio|LSM1761-11|am10761|658[0n]bp|Australia.New South Wales
Hippotion celerio|LNSWC575-08|AM 2296|658[0n]bp|Australia.New South Wales
Hippotion celerio|LNSWB152-05|05-NSW-01092|658[0n]bp|Australia.New South Wales
Hippotion celerio|LNSWC573-08|AM 2294|658[0n]bp|Australia.New South Wales
Hippotion celerio|LNSWE102-06|06-NSWE-00102|658[0n]bp|Australia.New South Wales
Hippotion celerio|LOQTI613-12|gvc17129-1L|658[0n]bp|Australia.Queensland
Hippotion celerio|PHLCC1141-11|BIOUG01235-A05|658[0n]bp|Australia.Australian Capital Territory
Hippotion celerio|LNSWC576-08|AM 2297|658[0n]bp|Australia.New South Wales
Hippotion celerio|LNSWE058-06|06-NSWE-00058|658[0n]bp|Australia.New South Wales
Hippotion celerio|LNSWC574-08|AM 2295|658[0n]bp|Australia.New South Wales
Hippotion celerio|LNSWC312-06|06-NSW-00312|658[0n]bp|Australia.New South Wales
Hippotion celerio|LOQTI045-10|gvc14670-1L|658[0n]bp|Australia.Queensland
Hippotion celerio|LNSWE053-06|06-NSWE-00053|658[0n]bp|Australia.New South Wales
Hippotion celerio|GWORA089-08|BC ZSM Lep 10461|655[0n]bp|Australia.Western Australia
Hippotion celerio|LNSWE077-06|06-NSWE-00077|656[0n]bp|Australia.New South Wales
Hippotion celerio|LNSWE123-06|06-NSWE-00123|613[0n]bp|Australia.New South Wales
Hippotion celerio|LNSWE055-06|06-NSWE-00055|600[0n]bp|Australia.New South Wales
Hippotion celerio|LNSWE072-06|06-NSWE-00072|600[0n]bp|Australia.New South Wales
Hippotion celerio|LNSWE004-06|06-NSWE-00004|600[0n]bp|Australia.New South Wales
Hippotion celerio|LNSWE138-06|06-NSWE-00138|591[0n]bp|Australia.New South Wales
Hippotion celerio|LNSWE106-06|06-NSWE-00106|595[0n]bp|Australia.New South Wales
Hippotion celerio|LNSWE048-06|06-NSWE-00048|597[0n]bp|Australia.New South Wales
Hippotion celerio|LNSWE068-06|06-NSWE-00068|615[0n]bp|Australia.New South Wales
Hippotion celerio|LNSWE054-06|06-NSWE-00054|615[0n]bp|Australia.New South Wales
Hippotion celerio|LNSWE131-06|06-NSWE-00131|561[0n]bp|Australia.New South Wales
Hippotion celerio|LNSWE108-06|06-NSWE-00108|560[0n]bp|Australia.New South Wales
Hippotion celerio|LNSWE127-06|06-NSWE-00127|615[2n]bp|Australia.New South Wales
Hippotion celerio|LNSWB153-05|05-NSW-01093|658[0n]bp|Australia.New South Wales
Hippotion celerio|LNSWE050-06|06-NSWE-00050|658[0n]bp|Australia.New South Wales
Hippotion celerio|GWORN207-09|BC ZSM Lep 18235|658[0n]bp|Australia.Western Australia
Hippotion brennus|ANICC092-08|ANIC Gen No. 003255|658[0n]bp|Australia.Queensland
Hippotion brennus|SPHJT149-11|BC-LTM-171|658[0n]bp|Australia.New South Wales
Hippotion brennus|LOQTE455-09|gvc12757-1L|658[0n]bp|Australia.Queensland
Hippotion brennus|GWORY462-10|BC EF Lep 03416|658[0n]bp|Australia.Queensland
Hippotion brennus|SPTMA922-09|BC-Mel 1000|643[0n]bp|Australia.Queensland
Hippotion brennus|SPHJT147-11|BC-LTM-169|658[0n]bp|Australia.Queensland
Hippotion brennus|ANICC030-08|ANIC Gen No. 003193|613[0n]bp|Australia.Queensland
Hippotion brennus|ANICC031-08|ANIC Gen No. 003194|614[0n]bp|Australia.Queensland
Hippotion scrofa|GWORI552-09|BC ZSM Lep 13300|658[0n]bp|Australia.Northern Territory
Hippotion scrofa|NSWHM2102-11|BIOUG00961-B01|658[0n]bp|Australia.New South Wales
Hippotion scrofa|LNSWE135-06|06-NSWE-00135|658[0n]bp|Australia.New South Wales
Hippotion scrofa|SPRBA756-09|BC-RBP-1800|658[0n]bp|Australia.Queensland
Hippotion scrofa|LNSWE057-06|06-NSWE-00057|658[0n]bp|Australia.New South Wales
Hippotion scrofa|PHLCC1142-11|BIOUG01235-A06|658[0n]bp|Australia.Australian Capital Territory
Hippotion scrofa|LSM1704-11|am10638|658[0n]bp|Australia.New South Wales
Hippotion scrofa|LNSWC638-08|AM 2359|658[0n]bp|Australia.New South Wales
Hippotion scrofa|NSWHJ619-10|09-NSWHH-0632|658[0n]bp|Australia.New South Wales
Hippotion scrofa|LNSWE083-06|06-NSWE-00083|658[0n]bp|Australia.New South Wales
Hippotion scrofa|LNSWE026-06|06-NSWE-00026|658[0n]bp|Australia.New South Wales
Hippotion scrofa|NSWHM2154-11|BIOUG00961-F05|658[0n]bp|Australia.New South Wales
Hippotion scrofa|LNSWE113-06|06-NSWE-00113|658[0n]bp|Australia.New South Wales
Hippotion scrofa|NSWHH058-09|08-NSWHH-0058|658[0n]bp|Australia.New South Wales
Hippotion scrofa|LNSWC306-06|06-NSW-00306|658[0n]bp|Australia.New South Wales
Hippotion scrofa|LNSWE074-06|06-NSWE-00074|658[0n]bp|Australia.New South Wales
Hippotion scrofa|LNSWE046-06|06-NSWE-00046|658[0n]bp|Australia.New South Wales
Hippotion scrofa|GWORN203-09|BC ZSM Lep 18231|658[0n]bp|Australia.Western Australia
Hippotion scrofa|NSWHJ965-10|09-NSWHH-0978|658[0n]bp|Australia.New South Wales
Hippotion scrofa|LOQTI638-12|gvc17316-1L|658[0n]bp|Australia.Queensland
Hippotion scrofa|NSWHH051-09|08-NSWHH-0051|658[0n]bp|Australia.New South Wales
Hippotion scrofa|LOQT486-06|gvc6313-1L|658[0n]bp|Australia.Queensland
Hippotion scrofa|LNSWC637-08|AM 2358|658[0n]bp|Australia.New South Wales
Hippotion scrofa|GWORN205-09|BC ZSM Lep 18233|658[0n]bp|Australia.Western Australia
Hippotion scrofa|AMWW022-11|K290762|658[0n]bp|Australia.New South Wales
Hippotion scrofa|LCANA353-06|05-ACTC-353|658[0n]bp|Australia.Australian Capital Territory
Hippotion scrofa|LNSWC308-06|06-NSW-00308|658[0n]bp|Australia.New South Wales
Hippotion scrofa|NSWHM2119-11|BIOUG00961-C06|658[0n]bp|Australia.New South Wales
Hippotion scrofa|NSWHH050-09|08-NSWHH-0050|658[0n]bp|Australia.New South Wales
Hippotion scrofa|NSWHJ962-10|09-NSWHH-0975|658[0n]bp|Australia.New South Wales
Hippotion scrofa|IMLQ233-07|IM07-0165|658[0n]bp|Australia.Queensland
Hippotion scrofa|LNSWC307-06|06-NSW-00307|658[0n]bp|Australia.New South Wales
Hippotion scrofa|LNSWE025-06|06-NSWE-00025|658[0n]bp|Australia.New South Wales
Hippotion scrofa|NSWHM017-11|BIOUG00851-G10|658[0n]bp|Australia.New South Wales
Hippotion scrofa|SPRBA166-08|BC-RBP-0166|658[0n]bp|Australia.Queensland
Hippotion scrofa|LOQTC419-07|gvc8055-1L|658[0n]bp|Australia.Queensland
Hippotion scrofa|LNSWE059-06|06-NSWE-00059|658[0n]bp|Australia.New South Wales
Hippotion scrofa|LNSWB126-05|05-NSW-01066|658[0n]bp|Australia.New South Wales
Hippotion scrofa|NSWHM451-11|BIOUG00912-F12|658[0n]bp|Australia.New South Wales
Hippotion scrofa|NSWHH053-09|08-NSWHH-0053|658[0n]bp|Australia.New South Wales
Hippotion scrofa|NSWHJ964-10|09-NSWHH-0977|658[0n]bp|Australia.New South Wales
Hippotion scrofa|NSWBB1216-08|07-NSWBB-1216|656[0n]bp|Australia.New South Wales
Hippotion scrofa|IMLQ127-07|IM06-0428|656[0n]bp|Australia.Queensland
Hippotion scrofa|LNSWE086-06|06-NSWE-00086|600[0n]bp|Australia.New South Wales
Hippotion scrofa|NSWHJ191-10|09-NSWHH-0204|638[0n]bp|Australia.New South Wales
Hippotion scrofa|LNSWE104-06|06-NSWE-00104|594[0n]bp|Australia.New South Wales
Hippotion scrofa|LNSWE078-06|06-NSWE-00078|595[0n]bp|Australia.New South Wales
Hippotion scrofa|LNSWE124-06|06-NSWE-00124|603[0n]bp|Australia.New South Wales
Hippotion scrofa|LNSWE076-06|06-NSWE-00076|581[0n]bp|Australia.New South Wales
Hippotion scrofa|LNSWE105-06|06-NSWE-00105|596[0n]bp|Australia.New South Wales
Hippotion scrofa|GWORC178-07|BC ZSM Lep 02528|595[0n]bp|Australia.Queensland
Hippotion scrofa|LNSWE095-06|06-NSWE-00095|595[0n]bp|Australia.New South Wales
Hippotion scrofa|LNSWE111-06|06-NSWE-00111|550[1n]bp|Australia.New South Wales
Hippotion scrofa|LNSWE097-06|06-NSWE-00097|595[0n]bp|Australia.New South Wales
Hippotion scrofa|LNSWE063-06|06-NSWE-00063|577[0n]bp|Australia.New South Wales
Hippotion scrofa|LNSWE120-06|06-NSWE-00120|578[2n]bp|Australia.New South Wales
Hippotion scrofa|LNSWE005-06|06-NSWE-00005|564[0n]bp|Australia.New South Wales
Hippotion scrofa|LNSWE103-06|06-NSWE-00103|538[0n]bp|Australia.New South Wales
Hippotion scrofa|LNSWE112-06|06-NSWE-00112|595[1n]bp|Australia.New South Wales

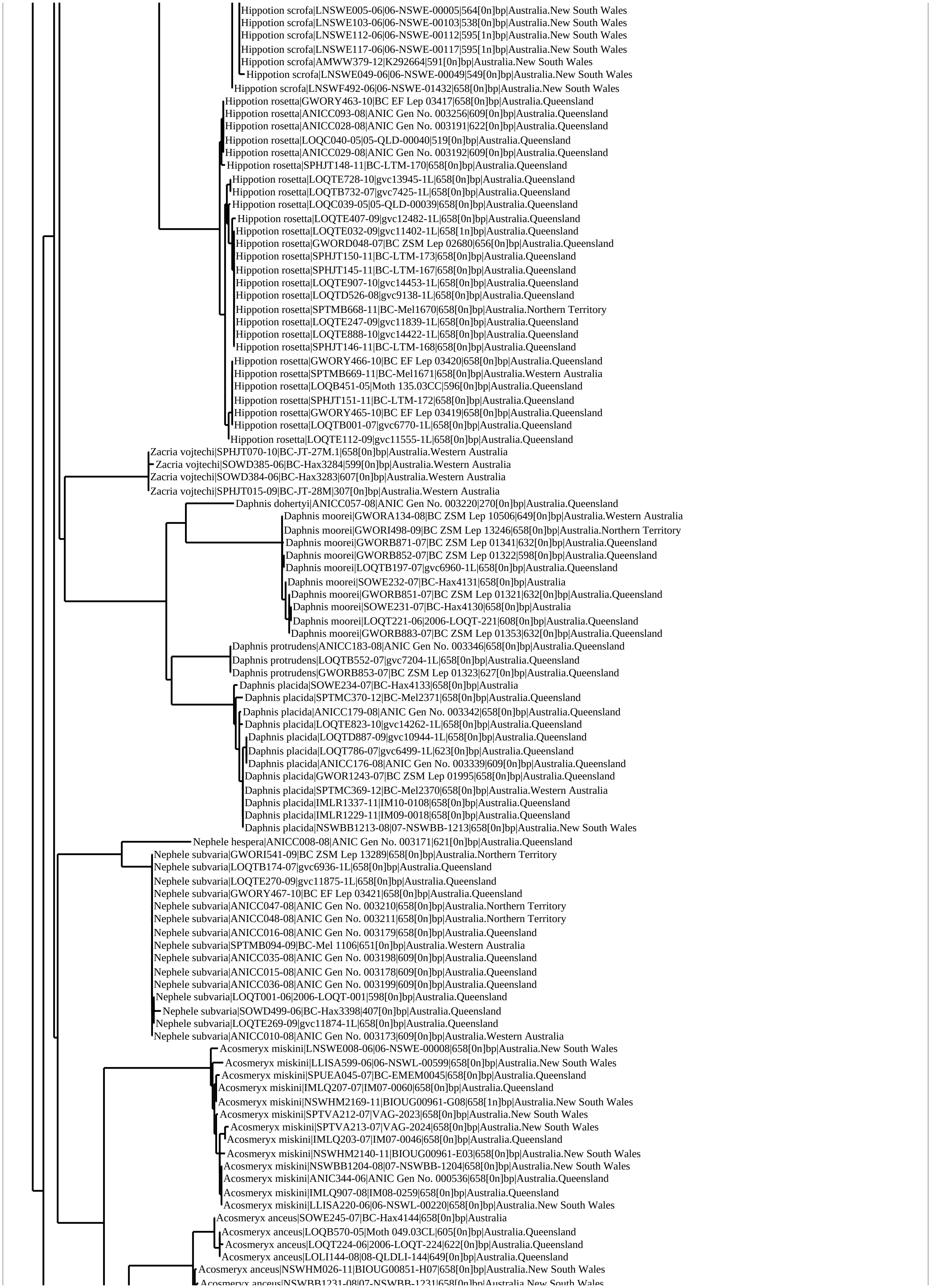
Hippotion scrofa|LNSWE005-06|06-NSWE-00005|564[0n]bp|Australia.New South Wales
Hippotion scrofa|LNSWE103-06|06-NSWE-00103|538[0n]bp|Australia.New South Wales
Hippotion scrofa|LNSWE112-06|06-NSWE-00112|595[1n]bp|Australia.New South Wales
Hippotion scrofa|LNSWE117-06|06-NSWE-00117|595[1n]bp|Australia.New South Wales
Hippotion scrofa|AMWW379-12|K292664|591[0n]bp|Australia.New South Wales
Hippotion scrofa|LNSWE049-06|06-NSWE-00049|549[0n]bp|Australia.New South Wales
Hippotion scrofa|LNSWF492-06|06-NSWE-01432|658[0n]bp|Australia.New South Wales
Hippotion rosetta|GWORY463-10|BC EF Lep 03417|658[0n]bp|Australia.Queensland
Hippotion rosetta|ANICC093-08|ANIC Gen No. 003256|609[0n]bp|Australia.Queensland
Hippotion rosetta|ANICC028-08|ANIC Gen No. 003191|622[0n]bp|Australia.Queensland
Hippotion rosetta|LOQC040-05|05-QLD-00040|519[0n]bp|Australia.Queensland
Hippotion rosetta|ANICC029-08|ANIC Gen No. 003192|609[0n]bp|Australia.Queensland
Hippotion rosetta|SPHJT148-11|BC-LTM-170|658[0n]bp|Australia.Queensland
Hippotion rosetta|LOQTE728-10|gvc13945-1L|658[0n]bp|Australia.Queensland
Hippotion rosetta|LOQTB732-07|gvc7425-1L|658[0n]bp|Australia.Queensland
Hippotion rosetta|LOQC039-05|05-QLD-00039|658[0n]bp|Australia.Queensland
Hippotion rosetta|LOQTE407-09|gvc12482-1L|658[0n]bp|Australia.Queensland
Hippotion rosetta|LOQTE032-09|gvc11402-1L|658[1n]bp|Australia.Queensland
Hippotion rosetta|GWORD048-07|BC ZSM Lep 02680|656[0n]bp|Australia.Queensland
Hippotion rosetta|SPHJT150-11|BC-LTM-173|658[0n]bp|Australia.Queensland
Hippotion rosetta|SPHJT145-11|BC-LTM-167|658[0n]bp|Australia.Queensland
Hippotion rosetta|LOQTE907-10|gvc14453-1L|658[0n]bp|Australia.Queensland
Hippotion rosetta|LOQTD526-08|gvc9138-1L|658[0n]bp|Australia.Queensland
Hippotion rosetta|SPTMB668-11|BC-Mel1670|658[0n]bp|Australia.Northern Territory
Hippotion rosetta|LOQTE247-09|gvc11839-1L|658[0n]bp|Australia.Queensland
Hippotion rosetta|LOQTE888-10|gvc14422-1L|658[0n]bp|Australia.Queensland
Hippotion rosetta|SPHJT146-11|BC-LTM-168|658[0n]bp|Australia.Queensland
Hippotion rosetta|GWORY466-10|BC EF Lep 03420|658[0n]bp|Australia.Queensland
Hippotion rosetta|SPTMB669-11|BC-Mel1671|658[0n]bp|Australia.Western Australia
Hippotion rosetta|LOQB451-05|Moth 135.03CC|596[0n]bp|Australia.Queensland
Hippotion rosetta|SPHJT151-11|BC-LTM-172|658[0n]bp|Australia.Queensland
Hippotion rosetta|GWORY465-10|BC EF Lep 03419|658[0n]bp|Australia.Queensland
Hippotion rosetta|LOQTB001-07|gvc6770-1L|658[0n]bp|Australia.Queensland
Hippotion rosetta|LOQTE112-09|gvc11555-1L|658[0n]bp|Australia.Queensland
Zacria vojtechi|SPHJT070-10|BC-JT-27M.1|658[0n]bp|Australia.Western Australia
Zacria vojtechi|SOWD385-06|BC-Hax3284|599[0n]bp|Australia.Western Australia
Zacria vojtechi|SOWD384-06|BC-Hax3283|607[0n]bp|Australia.Western Australia
Zacria vojtechi|SPHJT015-09|BC-JT-28M|307[0n]bp|Australia.Western Australia
Daphnis dohertyi|ANICC057-08|ANIC Gen No. 003220|270[0n]bp|Australia.Queensland
Daphnis moorei|GWORA134-08|BC ZSM Lep 10506|649[0n]bp|Australia.Western Australia
Daphnis moorei|GWORI498-09|BC ZSM Lep 13246|658[0n]bp|Australia.Northern Territory
Daphnis moorei|GWORB871-07|BC ZSM Lep 01341|632[0n]bp|Australia.Queensland
Daphnis moorei|GWORB852-07|BC ZSM Lep 01322|598[0n]bp|Australia.Queensland
Daphnis moorei|LOQTB197-07|gvc6960-1L|658[0n]bp|Australia.Queensland
Daphnis moorei|SOWE232-07|BC-Hax4131|658[0n]bp|Australia
Daphnis moorei|GWORB851-07|BC ZSM Lep 01321|632[0n]bp|Australia.Queensland
Daphnis moorei|SOWE231-07|BC-Hax4130|658[0n]bp|Australia
Daphnis moorei|LOQT221-06|2006-LOQT-221|608[0n]bp|Australia.Queensland
Daphnis moorei|GWORB883-07|BC ZSM Lep 01353|632[0n]bp|Australia.Queensland
Daphnis protrudens|ANICC183-08|ANIC Gen No. 003346|658[0n]bp|Australia.Queensland
Daphnis protrudens|LOQTB552-07|gvc7204-1L|658[0n]bp|Australia.Queensland
Daphnis protrudens|GWORB853-07|BC ZSM Lep 01323|627[0n]bp|Australia.Queensland
Daphnis placida|SOWE234-07|BC-Hax4133|658[0n]bp|Australia
Daphnis placida|SPTMC370-12|BC-Mel2371|658[0n]bp|Australia.Queensland
Daphnis placida|ANICC179-08|ANIC Gen No. 003342|658[0n]bp|Australia.Queensland
Daphnis placida|LOQTE823-10|gvc14262-1L|658[0n]bp|Australia.Queensland
Daphnis placida|LOQTD887-09|gvc10944-1L|658[0n]bp|Australia.Queensland
Daphnis placida|LOQT786-07|gvc6499-1L|623[0n]bp|Australia.Queensland
Daphnis placida|ANICC176-08|ANIC Gen No. 003339|609[0n]bp|Australia.Queensland
Daphnis placida|GWOR1243-07|BC ZSM Lep 01995|658[0n]bp|Australia.Queensland
Daphnis placida|SPTMC369-12|BC-Mel2370|658[0n]bp|Australia.Western Australia
Daphnis placida|IMLR1337-11|IM10-0108|658[0n]bp|Australia.Queensland
Daphnis placida|IMLR1229-11|IM09-0018|658[0n]bp|Australia.Queensland
Daphnis placida|NSWBB1213-08|07-NSWBB-1213|658[0n]bp|Australia.New South Wales
Nephele hespera|ANICC008-08|ANIC Gen No. 003171|621[0n]bp|Australia.Queensland
Nephele subvaria|GWORI541-09|BC ZSM Lep 13289|658[0n]bp|Australia.Northern Territory
Nephele subvaria|LOQTB174-07|gvc6936-1L|658[0n]bp|Australia.Queensland
Nephele subvaria|LOQTE270-09|gvc11875-1L|658[0n]bp|Australia.Queensland
Nephele subvaria|GWORY467-10|BC EF Lep 03421|658[0n]bp|Australia.Queensland
Nephele subvaria|ANICC047-08|ANIC Gen No. 003210|658[0n]bp|Australia.Northern Territory
Nephele subvaria|ANICC048-08|ANIC Gen No. 003211|658[0n]bp|Australia.Northern Territory
Nephele subvaria|ANICC016-08|ANIC Gen No. 003179|658[0n]bp|Australia.Queensland
Nephele subvaria|SPTMB094-09|BC-Mel 1106|651[0n]bp|Australia.Western Australia
Nephele subvaria|ANICC035-08|ANIC Gen No. 003198|609[0n]bp|Australia.Queensland
Nephele subvaria|ANICC015-08|ANIC Gen No. 003178|609[0n]bp|Australia.Queensland
Nephele subvaria|ANICC036-08|ANIC Gen No. 003199|609[0n]bp|Australia.Queensland
Nephele subvaria|LOQT001-06|2006-LOQT-001|598[0n]bp|Australia.Queensland
Nephele subvaria|SOWD499-06|BC-Hax3398|407[0n]bp|Australia.Queensland
Nephele subvaria|LOQTE269-09|gvc11874-1L|658[0n]bp|Australia.Queensland
Nephele subvaria|ANICC010-08|ANIC Gen No. 003173|609[0n]bp|Australia.Western Australia
Acosmeryx miskini|LNSWE008-06|06-NSWE-00008|658[0n]bp|Australia.New South Wales
Acosmeryx miskini|LLISA599-06|06-NSWL-00599|658[0n]bp|Australia.New South Wales
Acosmeryx miskini|SPUEA045-07|BC-EMEM0045|658[0n]bp|Australia.Queensland
Acosmeryx miskini|IMLQ207-07|IM07-0060|658[0n]bp|Australia.Queensland
Acosmeryx miskini|NSWHM2169-11|BIOUG00961-G08|658[1n]bp|Australia.New South Wales
Acosmeryx miskini|SPTVA212-07|VAG-2023|658[0n]bp|Australia.New South Wales
Acosmeryx miskini|SPTVA213-07|VAG-2024|658[0n]bp|Australia.New South Wales
Acosmeryx miskini|IMLQ203-07|IM07-0046|658[0n]bp|Australia.Queensland
Acosmeryx miskini|NSWHM2140-11|BIOUG00961-E03|658[0n]bp|Australia.New South Wales
Acosmeryx miskini|NSWBB1204-08|07-NSWBB-1204|658[0n]bp|Australia.New South Wales
Acosmeryx miskini|ANIC344-06|ANIC Gen No. 000536|658[0n]bp|Australia.Queensland
Acosmeryx miskini|IMLQ907-08|IM08-0259|658[0n]bp|Australia.Queensland
Acosmeryx miskini|LLISA220-06|06-NSWL-00220|658[0n]bp|Australia.New South Wales
Acosmeryx anceus|SOWE245-07|BC-Hax4144|658[0n]bp|Australia
Acosmeryx anceus|LOQB570-05|Moth 049.03CL|605[0n]bp|Australia.Queensland
Acosmeryx anceus|LOQT224-06|2006-LOQT-224|622[0n]bp|Australia.Queensland
Acosmeryx anceus|LOLI144-08|08-QLDLI-144|649[0n]bp|Australia.Queensland
Acosmeryx anceus|NSWHM026-11|BIOUG00851-H07|658[0n]bp|Australia.New South Wales
Acosmeryx anceus|NSWBB1231-08|07-NSWBB-1231|658[0n]bp|Australia.New South Wales

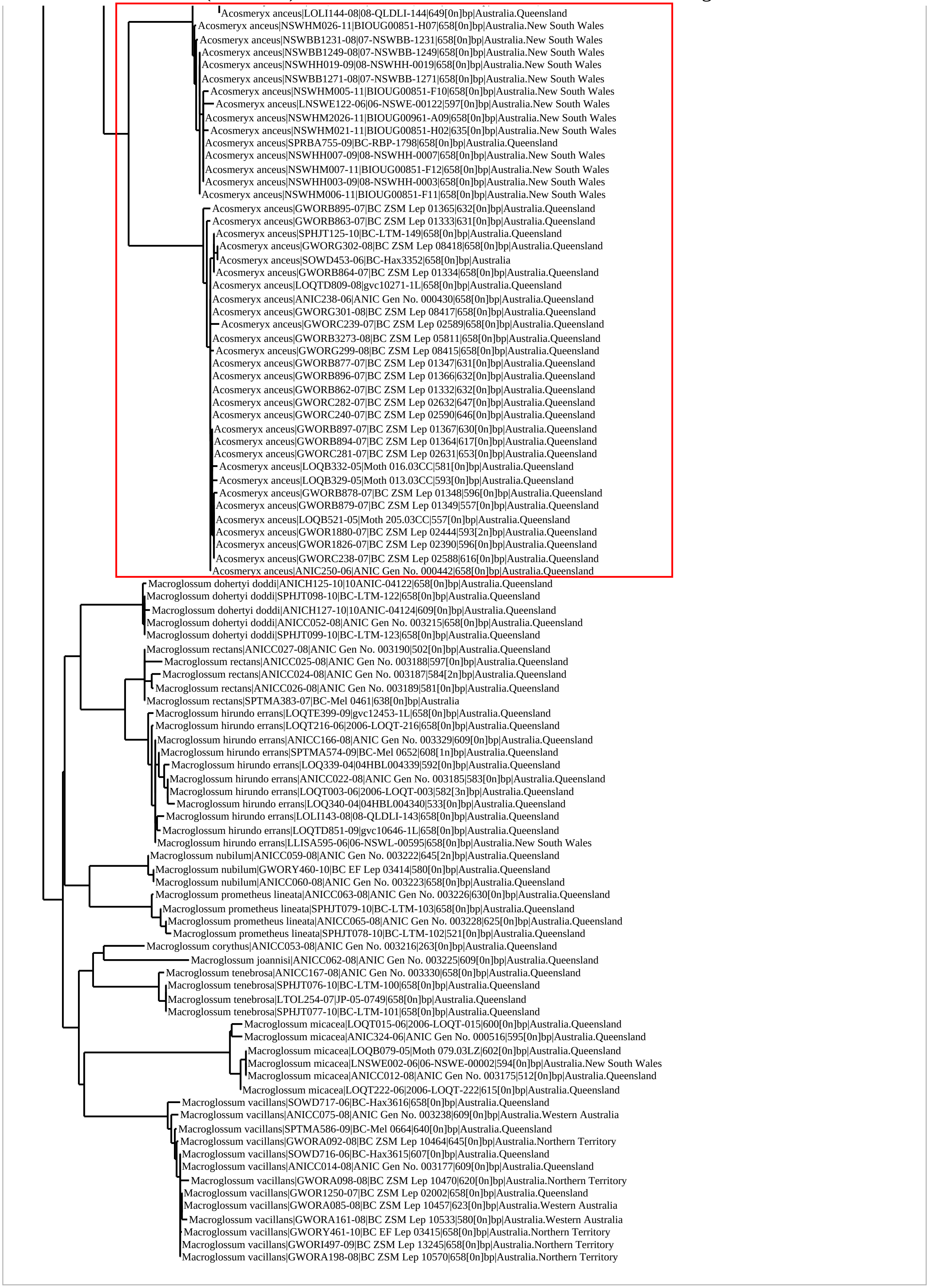
Acosmeryx anceus|LOLI144-08|08-QLDLI-144|649[0n]bp|Australia.Queensland
Acosmeryx anceus|NSWHM026-11|BIOUG00851-H07|658[0n]bp|Australia.New South Wales
Acosmeryx anceus|NSWBB1231-08|07-NSWBB-1231|658[0n]bp|Australia.New South Wales
Acosmeryx anceus|NSWBB1249-08|07-NSWBB-1249|658[0n]bp|Australia.New South Wales
Acosmeryx anceus|NSWHH019-09|08-NSWHH-0019|658[0n]bp|Australia.New South Wales
Acosmeryx anceus|NSWBB1271-08|07-NSWBB-1271|658[0n]bp|Australia.New South Wales
Acosmeryx anceus|NSWHM005-11|BIOUG00851-F10|658[0n]bp|Australia.New South Wales
Acosmeryx anceus|LNSWE122-06|06-NSWE-00122|597[0n]bp|Australia.New South Wales
Acosmeryx anceus|NSWHM2026-11|BIOUG00961-A09|658[0n]bp|Australia.New South Wales
Acosmeryx anceus|NSWHM021-11|BIOUG00851-H02|635[0n]bp|Australia.New South Wales
Acosmeryx anceus|SPRBA755-09|BC-RBP-1798|658[0n]bp|Australia.Queensland
Acosmeryx anceus|NSWHH007-09|08-NSWHH-0007|658[0n]bp|Australia.New South Wales
Acosmeryx anceus|NSWHM007-11|BIOUG00851-F12|658[0n]bp|Australia.New South Wales
Acosmeryx anceus|NSWHH003-09|08-NSWHH-0003|658[0n]bp|Australia.New South Wales
Acosmeryx anceus|NSWHM006-11|BIOUG00851-F11|658[0n]bp|Australia.New South Wales
Acosmeryx anceus|GWORB895-07|BC ZSM Lep 01365|632[0n]bp|Australia.Queensland
Acosmeryx anceus|GWORB863-07|BC ZSM Lep 01333|631[0n]bp|Australia.Queensland
Acosmeryx anceus|SPHJT125-10|BC-LTM-149|658[0n]bp|Australia.Queensland
Acosmeryx anceus|GWORG302-08|BC ZSM Lep 08418|658[0n]bp|Australia.Queensland
Acosmeryx anceus|SOWD453-06|BC-Hax3352|658[0n]bp|Australia
Acosmeryx anceus|GWORB864-07|BC ZSM Lep 01334|658[0n]bp|Australia.Queensland
Acosmeryx anceus|LOQTD809-08|gvc10271-1L|658[0n]bp|Australia.Queensland
Acosmeryx anceus|ANIC238-06|ANIC Gen No. 000430|658[0n]bp|Australia.Queensland
Acosmeryx anceus|GWORG301-08|BC ZSM Lep 08417|658[0n]bp|Australia.Queensland
Acosmeryx anceus|GWORC239-07|BC ZSM Lep 02589|658[0n]bp|Australia.Queensland
Acosmeryx anceus|GWORB3273-08|BC ZSM Lep 05811|658[0n]bp|Australia.Queensland
Acosmeryx anceus|GWORG299-08|BC ZSM Lep 08415|658[0n]bp|Australia.Queensland
Acosmeryx anceus|GWORB877-07|BC ZSM Lep 01347|631[0n]bp|Australia.Queensland
Acosmeryx anceus|GWORB896-07|BC ZSM Lep 01366|632[0n]bp|Australia.Queensland
Acosmeryx anceus|GWORB862-07|BC ZSM Lep 01332|632[0n]bp|Australia.Queensland
Acosmeryx anceus|GWORC282-07|BC ZSM Lep 02632|647[0n]bp|Australia.Queensland
Acosmeryx anceus|GWORC240-07|BC ZSM Lep 02590|646[0n]bp|Australia.Queensland
Acosmeryx anceus|GWORB897-07|BC ZSM Lep 01367|630[0n]bp|Australia.Queensland
Acosmeryx anceus|GWORB894-07|BC ZSM Lep 01364|617[0n]bp|Australia.Queensland
Acosmeryx anceus|GWORC281-07|BC ZSM Lep 02631|653[0n]bp|Australia.Queensland
Acosmeryx anceus|LOQB332-05|Moth 016.03CC|581[0n]bp|Australia.Queensland
Acosmeryx anceus|LOQB329-05|Moth 013.03CC|593[0n]bp|Australia.Queensland
Acosmeryx anceus|GWORB878-07|BC ZSM Lep 01348|596[0n]bp|Australia.Queensland
Acosmeryx anceus|GWORB879-07|BC ZSM Lep 01349|557[0n]bp|Australia.Queensland
Acosmeryx anceus|LOQB521-05|Moth 205.03CC|557[0n]bp|Australia.Queensland
Acosmeryx anceus|GWOR1880-07|BC ZSM Lep 02444|593[2n]bp|Australia.Queensland
Acosmeryx anceus|GWOR1826-07|BC ZSM Lep 02390|596[0n]bp|Australia.Queensland
Acosmeryx anceus|GWORC238-07|BC ZSM Lep 02588|616[0n]bp|Australia.Queensland
Acosmeryx anceus|ANIC250-06|ANIC Gen No. 000442|658[0n]bp|Australia.Queensland
Macroglossum dohertyi doddi|ANICH125-10|10ANIC-04122|658[0n]bp|Australia.Queensland
Macroglossum dohertyi doddi|SPHJT098-10|BC-LTM-122|658[0n]bp|Australia.Queensland
Macroglossum dohertyi doddi|ANICH127-10|10ANIC-04124|609[0n]bp|Australia.Queensland
Macroglossum dohertyi doddi|ANICC052-08|ANIC Gen No. 003215|658[0n]bp|Australia.Queensland
Macroglossum dohertyi doddi|SPHJT099-10|BC-LTM-123|658[0n]bp|Australia.Queensland
Macroglossum rectans|ANICC027-08|ANIC Gen No. 003190|502[0n]bp|Australia.Queensland
Macroglossum rectans|ANICC025-08|ANIC Gen No. 003188|597[0n]bp|Australia.Queensland
Macroglossum rectans|ANICC024-08|ANIC Gen No. 003187|584[2n]bp|Australia.Queensland
Macroglossum rectans|ANICC026-08|ANIC Gen No. 003189|581[0n]bp|Australia.Queensland
Macroglossum rectans|SPTMA383-07|BC-Mel 0461|638[0n]bp|Australia
Macroglossum hirundo errans|LOQTE399-09|gvc12453-1L|658[0n]bp|Australia.Queensland
Macroglossum hirundo errans|LOQT216-06|2006-LOQT-216|658[0n]bp|Australia.Queensland
Macroglossum hirundo errans|ANICC166-08|ANIC Gen No. 003329|609[0n]bp|Australia.Queensland
Macroglossum hirundo errans|SPTMA574-09|BC-Mel 0652|608[1n]bp|Australia.Queensland
Macroglossum hirundo errans|LOQ339-04|04HBL004339|592[0n]bp|Australia.Queensland
Macroglossum hirundo errans|ANICC022-08|ANIC Gen No. 003185|583[0n]bp|Australia.Queensland
Macroglossum hirundo errans|LOQT003-06|2006-LOQT-003|582[3n]bp|Australia.Queensland
Macroglossum hirundo errans|LOQ340-04|04HBL004340|533[0n]bp|Australia.Queensland
Macroglossum hirundo errans|LOLI143-08|08-QLDLI-143|658[0n]bp|Australia.Queensland
Macroglossum hirundo errans|LOQTD851-09|gvc10646-1L|658[0n]bp|Australia.Queensland
Macroglossum hirundo errans|LLISA595-06|06-NSWL-00595|658[0n]bp|Australia.New South Wales
Macroglossum nubilum|ANICC059-08|ANIC Gen No. 003222|645[2n]bp|Australia.Queensland
Macroglossum nubilum|GWORY460-10|BC EF Lep 03414|580[0n]bp|Australia.Queensland
Macroglossum nubilum|ANICC060-08|ANIC Gen No. 003223|658[0n]bp|Australia.Queensland
Macroglossum prometheus lineata|ANICC063-08|ANIC Gen No. 003226|630[0n]bp|Australia.Queensland
Macroglossum prometheus lineata|SPHJT079-10|BC-LTM-103|658[0n]bp|Australia.Queensland
Macroglossum prometheus lineata|ANICC065-08|ANIC Gen No. 003228|625[0n]bp|Australia.Queensland
Macroglossum prometheus lineata|SPHJT078-10|BC-LTM-102|521[0n]bp|Australia.Queensland
Macroglossum corythus|ANICC053-08|ANIC Gen No. 003216|263[0n]bp|Australia.Queensland
Macroglossum joannisi|ANICC062-08|ANIC Gen No. 003225|609[0n]bp|Australia.Queensland
Macroglossum tenebrosa|ANICC167-08|ANIC Gen No. 003330|658[0n]bp|Australia.Queensland
Macroglossum tenebrosa|SPHJT076-10|BC-LTM-100|658[0n]bp|Australia.Queensland
Macroglossum tenebrosa|LTOL254-07|JP-05-0749|658[0n]bp|Australia.Queensland
Macroglossum tenebrosa|SPHJT077-10|BC-LTM-101|658[0n]bp|Australia.Queensland
Macroglossum micacea|LOQT015-06|2006-LOQT-015|600[0n]bp|Australia.Queensland
Macroglossum micacea|ANIC324-06|ANIC Gen No. 000516|595[0n]bp|Australia.Queensland
Macroglossum micacea|LOQB079-05|Moth 079.03LZ|602[0n]bp|Australia.Queensland
Macroglossum micacea|LNSWE002-06|06-NSWE-00002|594[0n]bp|Australia.New South Wales
Macroglossum micacea|ANICC012-08|ANIC Gen No. 003175|512[0n]bp|Australia.Queensland
Macroglossum micacea|LOQT222-06|2006-LOQT-222|615[0n]bp|Australia.Queensland
Macroglossum vacillans|SOWD717-06|BC-Hax3616|658[0n]bp|Australia.Queensland
Macroglossum vacillans|ANICC075-08|ANIC Gen No. 003238|609[0n]bp|Australia.Western Australia
Macroglossum vacillans|SPTMA586-09|BC-Mel 0664|640[0n]bp|Australia.Queensland
Macroglossum vacillans|GWORA092-08|BC ZSM Lep 10464|645[0n]bp|Australia.Northern Territory
Macroglossum vacillans|SOWD716-06|BC-Hax3615|607[0n]bp|Australia.Queensland
Macroglossum vacillans|ANICC014-08|ANIC Gen No. 003177|609[0n]bp|Australia.Queensland
Macroglossum vacillans|GWORA098-08|BC ZSM Lep 10470|620[0n]bp|Australia.Northern Territory
Macroglossum vacillans|GWOR1250-07|BC ZSM Lep 02002|658[0n]bp|Australia.Queensland
Macroglossum vacillans|GWORA085-08|BC ZSM Lep 10457|623[0n]bp|Australia.Western Australia
Macroglossum vacillans|GWORA161-08|BC ZSM Lep 10533|580[0n]bp|Australia.Western Australia
Macroglossum vacillans|GWORY461-10|BC EF Lep 03415|658[0n]bp|Australia.Northern Territory
Macroglossum vacillans|GWORI497-09|BC ZSM Lep 13245|658[0n]bp|Australia.Northern Territory
Macroglossum vacillans|GWORA198-08|BC ZSM Lep 10570|658[0n]bp|Australia.Northern Territory
