# Supplementary material for: Australian Sphingidae – DNA Barcodes Challenge Current Species Boundaries and Distributions
Source: PLoS One. 2014 Jul 2;9(7):e101108. doi: 10.1371/journal.pone.0101108 (PMC4079597; doi:10.1371/journal.pone.0101108)
Supplement: Figure S2 — 28S–D2 rDNA sequence alignments. (PDF) [file pone.0101108.s002.pdf]

Figure S2: Sequence alignments for 28S-D2 rDNA sequences of *Macroglossum tenebrosa, Eupanacra splendens, Acosmeryx anceus* and *Agrius convolvuli.* Sequences are also available from BOLD datasets SPH01AUS and SPH02AUS (www.boldsystems.org) and from GenBank.

28S - *Macroglossum tenebrosa*

```
                              10        20        30        40        50        60        70        80        90       100
                      ....|....|....|....|....|....|....|....|....|....|....|....|....|....|....|....|....|....|....|....|
LTOL254-07 AUSTRALIA AGCCCTAAGTGGGTGGTAAACTCCATCTAAGGCTAAATATTACCGCGAGACCGATAGCGAACAAGTACCGTGAGGGAAAGTTGAAAAGAACTTTGAAGAG

SOWD753-06 SULAWESI  AGCCCTAAGTGGGTGGTAAACTCCATCTAAGGCTAAATATTACCGCGAGACCGATAGCGAACAAGTACCGTGAGGGAAAGTTGAAAAGAACTTTGAAGAG
SOWD754-06 SULAWESI  AGCCCTAAGTGGGTGGTAAACTCCATCTAAGGCTAAATATTACCGCGAGACCGATAGCGAACAAGTACCGTGAGGGAAAGTTGAAAAGAACTTTGAAGAG

                             110       120       130       140       150       160       170       180       190       200
                      ....|....|....|....|....|....|....|....|....|....|....|....|....|....|....|....|....|....|....|....|
LTOL254-07 AUSTRALIA AGAGTTCAAGAGTACGTGAAACCGTTCAGGGGTAAACCTGCGAAACTCGAATGAACGAACGGAGAGATTCATCGTCATTCCGGGGCGTACGTGCGTACTC

SOWD753-06 SULAWESI  AGAGTTCAAGAGTACGTGAAACCGTTCAGGGGTAAACCTGCGAAACTCGAATGAACGAACGGAGAGATTCATCGTCATTCCGGGGCGTACGTGCGTACTC
SOWD754-06 SULAWESI  AGAGTTCAAGAGTACGTGAAACCGTTCAGGGGTAAACCTGCGAAACTCGAATGAACGAACGGAGAGATTCATCGTCATTCCGGGGCGTACGTGCGTACTC

                             210       220       230       240       250       260       270       280       290       300
                      ....|....|....|....|....|....|....|....|....|....|....|....|....|....|....|....|....|....|....|....|
LTOL254-07 AUSTRALIA CACGATGTGAGCGGTGCGTTTACGCGTATCCGTTTGCACGGGACGCGCACGTCTCACGTCCTCGGACGGCGTGCACTTCTCTCTTAGTAATGCATCGCGA

SOWD753-06 SULAWESI  CACGATGTGAGCGGTGCGTTTACGCGTATCCGTTTGCACGGGACGCGCACGTCTCACGTCCTCGGACGGCGTGCACTTCTCTCTTAGTAATGCATCGCGA
SOWD754-06 SULAWESI  CACGATGTGAGCGGTGCGTTTACGCGTATCCGTTTGCACGGGACGCGCACGTCTCACGTCCTCGGACGGCGTGCACTTCTCTCTTAGTAATGCATCGCGA

                             310       320       330       340       350       360       370       380       390       400
                      ....|....|....|....|....|....|....|....|....|....|....|....|....|....|....|....|....|....|....|....|
LTOL254-07 AUSTRALIA CCCGTTCCGTTTATTATCTAAGCGCCTTTCGGGAGTCCCGGATGTATCCTTCACGGGTTACATTCGTGGACCGAACTTGATCGGGTGGCCGATAGTAACG

SOWD753-06 SULAWESI  CCCGTTCCGTTTATTATCTAAGCGCCTTTCGGGAGTCCCGGATGTATCCTTCACGGGTTACATTCGTGGACCGAACTTGATCGGGTGGCCGATAGTAACG
SOWD754-06 SULAWESI  CCCGTTCCGTTTATTATCTAAGCGCCTTTCGGGAGTCCCGGATGTATCCTTCACGGGTTACATTCGTGGACCGAACTTGATCGGGTGGCCGATAGTAACG

                             410       420       430       440       450       460       470       480       490       500
                      ....|....|....|....|....|....|....|....|....|....|....|....|....|....|....|....|....|....|....|....|
LTOL254-07 AUSTRALIA GGACGGTATTTGACAATGACGACACGCACGCGTTTAACGCGTCCGGCCCGACGCAAGTCAACGTCGCCTATCTTATTCCTGCCCGTGTGCGGACTAGGGT

SOWD753-06 SULAWESI  GGACGGTATTTGACAATGACGACACGCACGCGTTTAACGCGTCCGGCCCGACGCAAGTCAACGTCGCCTATCTTATTCCTGCCCGTGTGCGGACTAGGGT
SOWD754-06 SULAWESI  GGACGGTATTTGACAATGACGACACGCACGCGTTTAACGCGTCCGGCCCGACGCAAGTCAACGTCGCCTATCTTATTCCTGCCCGTGTGCGGACTAGGGT

                             510       520       530       540       550       560       570       580       590
                      ....|....|....|....|....|....|....|....|....|....|....|....|....|....|....|....|....|....|..
LTOL254-07 AUSTRALIA GCGGCGCGTCTGTTGTCGCCGCCGTGTCGTCTCGGACTTGTGCGTGTCGTTTCACTTTAGTGTATGTCTGCGATGATTCAGTTTCGGGCACTCGCAG

SOWD753-06 SULAWESI  GCGGCGCGTCTGTTGTCGCCGCCGTGTCGTCTCGGACTTGTGCGTGTCGTTTCACTTTAGTGTATGTCTGCGATGATTCAGTTTCGGGCACTCGCAG
SOWD754-06 SULAWESI  GCGGCGCGTCTGTTGTCGCCGCCGTGTCGTCTCGGACTTGTGCGTGTCGTTTCACTTTAGTGTATGTCTGCGATGATTCAGTTTCGGG
```

28S - *Eupanacra splendens*

```
                                      10        20        30        40        50        60        70        80        90       100
                              ....|....|....|....|....|....|....|....|....|....|....|....|....|....|....|....|....|....|....|....|
LOQTC288-07 Group 1 AUSTRALIA AGCCCTAAGTGGGTGGTAAACTCCATCTAAGGCTAAATATTACCGCGAGACCGATAGCGAACAAGTACCGTGAGGGAAAGTTGAAAAGAACTTTGAAGAG
LOQTD126-08 Group 1 AUSTRALIA AGCCctaagtgGGTGGTAAACTcCAtctAAGGcTAAATATTACCGCGAGACCGATAGCGAACAAGTACCGTGAGGGAAAGTTGAAAAGAACTTTGAAGAG
LOQTD377-08 Group 1 AUSTRALIA AGCCCTAAGTGGGTGGTAAACTCCATCTAAGGCTAAATATTACCGCGAGACCGATAGCGAACAAGTACCGTGAGGGAAAGTTGAAAAGAACTTTGAAGAG

GWOR1244-07 Group 2 AUSTRALIA AGCCCTAAGTGGGTGGTAAACTCCATCTAAGGCTAAATATTACCGCGAGACCGATAGCGAACAAGTACCGTGAGGGAAAGTTGAAAAGAACTTTGAAGAG
SOWC197-06 Group 2 PAPUA      AGCCCTAAGTGGGTGGTAAACTCCATCTAAGGCTAAATATTACCGCGAGACCGATAGCGAACAAGTACCGTGAGGGAAAGTTGAAAAGAACTTTGAAGAG

                                     110       120       130       140       150       160       170       180       190       200
                              ....|....|....|....|....|....|....|....|....|....|....|....|....|....|....|....|....|....|....|....|
LOQTC288-07 Group 1 AUSTRALIA AGAGTTCAAGAGTACGTGAAACCGTTCAGGGGTAAACCTGCGAAACTCGAATGAACGAACGGAGAGATTCATCGTCATTCCGGGGCGTACGTGCGTGCTC
LOQTD126-08 Group 1 AUSTRALIA AGAGTTCAAGAGTACGTGAAACCGTTCAGGGGTAAACCTGCGAAACTCGAATGAACGAACGGAGAGATTCATCGTCATTCCGGGGCGTACGTGCGTGCTC
LOQTD377-08 Group 1 AUSTRALIA AGAGTTCAAGAGTACGTGAAACCGTTCAGGGGTAAACCTGCGAAACTCGAATGAACGAACGGAGAGATTCATCGTCATTCCGGGGCGTACGTGCGTGCTC

GWOR1244-07 Group 2 AUSTRALIA AGAGTTCAAGAGTACGTGAAACCGTTCAGGGGTAAACCTGCGAAACTCGAATGAACGAACGGAGAGATTCATCGTCATTCCGGGGCGTACGTGCGTGCTC
SOWC197-06 Group 2 PAPUA      AGAGTTCAAGAGTACGTGAAACCGTTCAGGGGTAAACCTGCGAAACTCGAATGAACGAACGGAGAGATTCATCGTCATTCCGGGGCGTACGTGCGTGCTC

                                     210       220       230       240       250       260       270       280       290       300
                              ....|....|....|....|....|....|....|....|....|....|....|....|....|....|....|....|....|....|....|....|
LOQTC288-07 Group 1 AUSTRALIA CACGATGCGAGCGGTGCGTTTCGCGCGTGTCCGTTCGCACGGGACGCGCACGTCTCACGTCCTCGGACGGCGTGCACTTCTCTCTTAGTAATGCATCGCG
LOQTD126-08 Group 1 AUSTRALIA CACGATGCGAGCGGTGCGTTTCGCGCGTGTCCGTTCGCACGGGACGCGCACGTCTCACGTCCTCGGACGGCGTGCACTTCTCTCTTAGTAATGCATCGCG
LOQTD377-08 Group 1 AUSTRALIA CACGATGCGAGCGGTGCGTTTCGCGCGTGTCCGTCCGCACGGGACGCGCACGTCTCACGTCCTCGGACGGCGTGCACTTCTCTCTTAGTAATGCATCGCG

GWOR1244-07 Group 2 AUSTRALIA CACGATGCGAGCGGTGCGTTTCGCGCGTGTCCGTCCGCACGGGACGCGCACGTCTCACGTCCTCGGACGGCGTGCACTTCTCTCTTAGTAATGCATCGCG
SOWC197-06 Group 2 PAPUA      CACGATGCGAGCGGTGCGTTTCGCGCGTGTCCGTCCGCACGGGACGCGCACGTCTCACGTCCTCGGACGGCGTGCACTTCTCTCTTAGTAATGCATCGCG

                                     310       320       330       340       350       360       370       380       390       400
                              ....|....|....|....|....|....|....|....|....|....|....|....|....|....|....|....|....|....|....|....|
LOQTC288-07 Group 1 AUSTRALIA ACCCGTTCCGTCTCCAATTATCTAAGCGCCTTTCGGGAGTCCCGGATGTATTCTTTACCGGA-TACATTCGTGGACCGAACTTGATCGGGTGGCCGATAA
LOQTD126-08 Group 1 AUSTRALIA ACCCGTTCCGTCTCCAATTATCTAAGCGCCTTTCGGGAGTCCCGGATGTATTCTT--CGGGA-TACATTCA-GGACCGAACTTGATCGGGTGGCCGATAA
LOQTD377-08 Group 1 AUSTRALIA ACCCGTTCCGTCTCCAATTATCTAAGCGCCTTTCGGGAGTCCCGGATGTATTCTT---CGGATTACATTCA-GGACCGAACTTGATCGGGTGGCCGATAA

GWOR1244-07 Group 2 AUSTRALIA ACCCGTTCCGTCTCCAATTATCTAAGCGCCTTTCGGGAGTCCCGGATGTATTCTT---CGGATTACATTCA-GGACCGAACTTGATCGGGTGGCCGATAA
SOWC197-06 Group 2 PAPUA      ACCCGTTCCGTCTCCAATTATCTAAGCGCCTTTCGGGAGTCCCGGATGTATTCTT---CGGATTACATTCA-GGACCGAACTTGATCGGGTGGCCGATAA

                                     410       420       430       440       450       460       470       480       490       500
                              ....|....|....|....|....|....|....|....|....|....|....|....|....|....|....|....|....|....|....|....|
LOQTC288-07 Group 1 AUSTRALIA TGACGGGACGGTATTTGACAATGACGACGCGCACGCGTTTACAACGCGTCCGGCCCGACGCAAGACAACGTCGCCTATCTTATTCCTGCCTGTGTGCGGA
LOQTD126-08 Group 1 AUSTRALIA TGACGGGACGGTATTTGACAATGACGACGCGCACGCGTTT--AACGCGTCCGGCCCGACGCAAGACAACGTCGCCTATCTTATTCCTGCCTGTGTGCGGA
LOQTD377-08 Group 1 AUSTRALIA TGACGGGACGGTATTTGACAATGACGACGCGCACGCGTTTACAACGCGTCCGGCCCGACGCAAGACAACGTCGCCTATCTTATTCCTGCCTGTGTGCGGA

GWOR1244-07 Group 2 AUSTRALIA TGACGGGACGGTATTTGACAATGACGACGCGCACGCGTTTACAACGCGTCCGGCCCGACGCAAGACAACGTCGCCTATCTTATTCCTGCCTGTGTGCGGA
SOWC197-06 Group 2 PAPUA      TGACGGGACGGTATTTGACAATGACGACGCGCACGCGTTTACAACGCGTCCGGCCCGACGCAAGACAACGTCGCCTATCTTATTCCTGCCTGTGTGCGGA
```

```
                                         510       520       530       540       550       560       570       580       590       600
                                 ....|....|....|....|....|....|....|....|....|....|....|....|....|....|....|....|....|....|....|....|
LOQTC288-07 Group 1 AUSTRALIA    CTAGGGTGCGGCGCGTCTGTTGTCGCCGCCGTGTCGTCTCGGACTTGTGCGCGTCGTTTCACTTTACGTGTATGTCTGCGATGATTCAGTTTCGGGCACT
LOQTD126-08 Group 1 AUSTRALIA    CTAGGGTGCGGCGCGTCTGTTGTCGCTGCCGTGTCGTCTCGGACTTGTGCGCGTCGTTTCACTTTATGTGTATGTCTGCGATGATTCAGTTTCGGgCACT
LOQTD377-08 Group 1 AUSTRALIA    CTAGGGTGCGGCGCGTCTGTTGTCGCCGCCGTGTCGTCTCGGACTTGTGCGCGTCGTTTCACTTTATGTGTATGTCTGCGATGATTCAGTTTCGGGCACT

GWOR1244-07 Group 2 AUSTRALIA    CTAGGGTGCGGCGCGTCTGTTGTCGCCGCCGTGTCGTCTCGGACTTGTGCGCGTCGTTTCACTTTATGTGTATGTCTGCGATGATTCAGTTTCGGGCACT
SOWC197-06 Group 2 PAPUA         CTAGGGTGCGGCGCGTCTGTTGTCGCCGCCGTGTCGTCTCGGACTTGTGCGCGTCGTTTCACTTTATGTGTATGTCTGCGATGATTCAGTTTCGGGCACT


                                 ....|
LOQTC288-07 Group 1 AUSTRALIA    CGCAG
LOQTD126-08 Group 1 AUSTRALIA    CGCAG
LOQTD377-08 Group 1 AUSTRALIA    CGCAg

GWOR1244-07 Group 2 AUSTRALIA    CGCAG
SOWC197-06 Group 2 PAPUA         CGCAG
```

28S - *Acosmeryx anceus*

```
                                    10        20        30        40        50        60        70        80        90       100
                           ....|....|....|....|....|....|....|....|....|....|....|....|....|....|....|....|....|....|....|....|
SML190-06 Group 1 PAPUA    AGCCCTAAGTGGGTGGTAAACTCCATCTAAGGCTAAATATTACCGCGAGACCGATAGCGAACAAGTACCGTGAGGGAAAGTTGAAAAGAACTTTGAAGAG
SOWE245-07 Group 1 AUSTRALIA AGCCCTAAGTGGGTGGTAAACTCCATCTAAGGCTAAATATTACCGCGAGACCGATAGCGAACAAGTACCGTGAGGGAAAGTTGAAAAGAACTTTGAAGAG

ANIC238-06 Group 2 AUSTRALIA AGCCCTAAGTGGGTGGTAAACTCCATCTAAGGCTAAATATTACCGCGAGACCGATAGCGAACAAGTACCGTGAGGGAAAGTTGAAAAGAACTTTGAAGAG
ANIC250-06 Group 2 AUSTRALIA AGCCCTAAGTGGGTGGTaAACTCCATCTAAGGCTAAATATTACCGCGAGACCGATAGCGAACAAGTACCGTGAGGGAAAGTTGAAAAGAACTTTGAAGAG
SML185-06 Group 2 PAPUA    AGCCCTAAGTGGGTGGTAAACTCCATCTAAGGCTAAATATTACCGCGAGACCGATAGCGAACAAGTACCGTGAGGGAAAGTTGAAAAGAACTTTGAAGAG
SML189-06 Group 2 PAPUA    AGCCCTAAGTGGGTGGTAAACTCCATCTaAGGCTAAATATTACCGCGAGACCGATAGCGAACAAGTACCGTGAGGGAAAGTTGAAAAGAACTTTGAAGAG

                                   110       120       130       140       150       160       170       180       190       200
                           ....|....|....|....|....|....|....|....|....|....|....|....|....|....|....|....|....|....|....|....|
SML190-06 Group 1 PAPUA    AGAGTTCAAGAGTACGTGAAACCGTTCAGGGGTAAACCTGCGAAACTCGAATGAACGAACGGAGAGATTCATCGTCATTCCGGGGCGTACGTGCGTGCTC
SOWE245-07 Group 1 AUSTRALIA AGAGTTCAAGAGTACGTGAAACCGTTCAGGGGTAAACCTGCGAAACTCGAATGAACGAACGGAGAGATTCATCGTCATTCCGGGGCGTACGTGCGTGCTC

ANIC238-06 Group 2 AUSTRALIA AGAGTTCAAGAGTACGTGAAACCGTTCAGGGGTAAACCTGCGAAACTCGAATGAACGAACGGAGAGATTCATCGTCATTCCGGGGCGTACGTGCGTGCTC
ANIC250-06 Group 2 AUSTRALIA AGAGTTCAAGAGTACGTGAAACCGTTCAGGGGTAAACCTGCGAAACTCGAATGAACGAACGGAGAGATTCATCGTCATTCCGGGGCGTACGTGCGTGCTC
SML185-06 Group 2 PAPUA    AGAGTTCAAGAGTACGTGAAACCGTTCAGGGGTAAACCTGCGAAACTCGAATGAACGAACGGAGAGATTCATCGTCATTCCGGGGCGTACGTGCGTGCTC
SML189-06 Group 2 PAPUA    AGAGTTCAAGAGTACGTGAAACCGTTCAGGGGTAAACCTGCGAAACTCGAATGAACGAACGGAGAGATTCATCGTCATTCCGGGGCGTACGTGCGTGCTC

                                   210       220       230       240       250       260       270       280       290       300
                           ....|....|....|....|....|....|....|....|....|....|....|....|....|....|....|....|....|....|....|....|
SML190-06 Group 1 PAPUA    CACGATGCGAGCGGTGCGTTTCGCGCGTGTCCGTTCGCACGGGACGCGCACGTCTCACGTCCTCGGACGGCGTGCACTTCTCTCTTAGTAATGCATCGCG
SOWE245-07 Group 1 AUSTRALIA CACGATGCGAGCGGTGCGTTTCGCGCGTGTCCGTTCGCACGGGACGCGCACGTCTCACGTCCTCGGACGGCGTGCACTTCTCTCTTAGTAATGCATCGCG

ANIC238-06 Group 2 AUSTRALIA CACGATGCGAGCGGTGCGTTTCGCGCGTGTCCGTTCGCACGGGACGCGCACGTCTCACGTCCTCGGACGGCGTGCACTTCTCTCTTAGTAATGCATCGCG
ANIC250-06 Group 2 AUSTRALIA CACGATGCGAGCGGTGCGTTTCGCGCGTGTCCGTTCGCACGGGACGCGCACGTCTCACGTCCTCGGACGGCGTGCACTTCTCTCTTAGTAATGCATCGCG
SML185-06 Group 2 PAPUA    CACGATGCGAGCGGTGCGTTTCGCGCGTGTCCGTTCGCACGGGACGCGCACGTCTCACGTCCTCGGACGGCGTGCACTTCTCTCTTAGTAATGCATCGCG
SML189-06 Group 2 PAPUA    CACGATGCGAGCGGTGCGTTTCGCGCGTGTCCGTTCGCACGGGACGCGCACGTCTCACGTCCTCGGACGGCGTGCACTTCTCTCTTAGTAATGCATCGCG

                                   310       320       330       340       350       360       370       380       390       400
                           ....|....|....|....|....|....|....|....|....|....|....|....|....|....|....|....|....|....|....|....|
SML190-06 Group 1 PAPUA    ACCCGTTCCGTCTCCAATTATCTAAGCGCCTTTCGGGAGTCCCGGATGTATTCTTCACGGGGTATATTCTGGACCGAACTTGATCGGGTGGCCGATAATG
SOWE245-07 Group 1 AUSTRALIA ACCCGTTCCGTCTCCAATTATCTAAGCGCCTTTCGGGAGTCCCGGATGTATTCTTCACGGGGTACATTCTGGACCGAACTTGATCGGGTGGCCGATAATG

ANIC238-06 Group 2 AUSTRALIA ACCCGTTCCGTCTCCAATTATCTAAGCGCCTTTCGGGAGTCCCGGATGTATTCTTCACGGGGTACATTCTGGACCGAACTTGATCGGGTGGCCGATAATG
ANIC250-06 Group 2 AUSTRALIA ACCCGTTCCGTCTCCAATTATCTAAGCGCCTTTCGGGAGTCCCGGATGTATTCTTCACGGGGTACATTCTGGACCGAACTTGATCGGGTGGCCGATAATG
SML185-06 Group 2 PAPUA    ACCCGTTCCGTCTCCAATTATCTAAGCGCCTTTCGGGAGTCCCGGATGTATTCTTCACGGGGTACATTCTGGACCGAACTTGATCGGGTGGCCGATAATG
SML189-06 Group 2 PAPUA    ACCCGTTCCGTCTCCAATTATCTAAGCGCCTTTCGGGAGTCCCGGATGTATTCTTCACGGGGTACATTCTGGACCGAACTTGATCGGGTGGCCGATAATG

                                   410       420       430       440       450       460       470       480       490       500
                           ....|....|....|....|....|....|....|....|....|....|....|....|....|....|....|....|....|....|....|....|
SML190-06 Group 1 PAPUA    ACGGGACGGTATTTGACAATGACGACGCGCACGCGTTTACAACGCGTTCGGCCCGACGCAAGACAACGTCGCCTATCTTATTCCTGCCCGTGTGCGGACT
SOWE245-07 Group 1 AUSTRALIA ACGGGACGGTATTTGACAATGACGACGCGCACGCGTTTACAACGCGTTCGGCCCGACGCAAGACAACGTCGCCTATCTTATTCCTGCCCGTGTGCGGACT

ANIC238-06 Group 2 AUSTRALIA ACGGGACGGTATTTGACAATGACGACGCGCACGCGTTTACAACGCGTTCGGCCCGACGCAAGACAACGTCGCCTATCTTATTCCTGCCCGTGTGCGGACT
ANIC250-06 Group 2 AUSTRALIA ACGGGACGGTATTTGACAATGACGACGCGCACGCGTTTACAACGCGTTCGGCCCGACGCAAGACAACGTCGCCTATCTTATTCCTGCCCGTGTGCGGACT
SML185-06 Group 2 PAPUA    ACGGGACGGTATTTGACAATGACGACGCGCACGCGTTTACAACGCGTTCGGCCCGACGCAAGACAACGTCGCCTATCTTATTCCTGCCCGTGTGCGGACT
SML189-06 Group 2 PAPUA    ACGGGACGGTATTTGACAATGACGACGCGCACGCGTTTACAACGCGTTCGGCCCGACGCAAGACAACGTCGCCTATCTTATTCCTGCCCGTGTGCGGACT
```

```
                                      510       520       530       540       550       560       570       580       590       600
                               ....|....|....|....|....|....|....|....|....|....|....|....|....|....|....|....|....|....|....|....|
SML190-06 Group 1 PAPUA        AGGGTGCGGCGCGTCTGTTGTTGCAGCCGTGTCGTCTCGGACTTGTGCGTGTCGTTTCACTTTAGTGTATGTCTGCGATGATTCAGTTTCGGGCACTCGC
SOWE245-07 Group 1 AUSTRALIA   AGGGTGCGGCGCGTCTGTTGTTGCAGCCGTGTCGTCTCGGACTTGTGCGTGTCGTTTCACTTTAGTGTATGTCTGCGATGATTCAGTTTCGGGCACTCGC

ANIC238-06 Group 2 AUSTRALIA  AGGGTGCGGCGCGTCTGTTGTTGCAGCCGTGTCGTCTCGGACTTGTGCGCGTCGTTTCACTTTAGTGTATGTCTGCGATGATTCAGTTTCGGGCACTCGC
ANIC250-06 Group 2 AUSTRALIA  AGGGTGCGGCGCGTCTGTTGTTGCAGCCGTGTCGTCTCGGACTTGTGCGCGTCGTTTCACTTTAGTGTATGTCTGCGATGATTCAGTTTCGGGCACTCGC
SML185-06 Group 2 PAPUA        AGGGTGCGGCGCGTCTGTTGTTGCAGCCGTGTCGTCTCGGACTTGTGCGCGTCGTTTCACTTTAGTGTATGTCTGCGATGATTcAGTTTCGGGCACTCGC
SML189-06 Group 2 PAPUA        AGGGTGCGGCGCGTCTGTTGTTGCAGCCGTGTCGTCTCGGACTTGTGCGCGTCGTTTCACTTTAGTGTATGTCTGCGATGATTCAGTTTCGGGCACTCGC


                               ..
SML190-06 Group 1 PAPUA        AG
SOWE245-07 Group 1 AUSTRALIA   AG

ANIC238-06 Group 2 AUSTRALIA  AG
ANIC250-06 Group 2 AUSTRALIA  AG
SML185-06 Group 2 PAPUA        AG
SML189-06 Group 2 PAPUA        AG
```

28S - *Agrius convolvuli*

```
                                 10        20        30        40        50        60        70        80        90        100
                        ....|....|....|....|....|....|....|....|....|....|....|....|....|....|....|....|....|....|....|....|
SPHYE007-08 Group 1 FRANCE      AGCCCTAAGTGGGTGGTAAACTCCATCTAAGGCTAAATATTACCGCGAGACCGATAGCGAACAAGTACCGTGAGGGAAAGTTGAAAAGAACTTTGAAGAG
SPPBA360-07 Group 1 MADAGASCAR  AGCCCTAAGTGGGTGGTAAACTCCATCTAAGGCTAAATATTACCGCGAGACCGATAGCGAACAAGTACCGTGAGGGAAAGTTGAAAAGAACTTTGAAGAG
SSDA224-06 Group 1 TANZANIA     AGCCCTAAGTGGGTGGTAAACTCCATCTAAGGCTAAATATTACCGCGAGACCGATAGCGAACAAGTACCGTGAGGGAAAGTTGAAAAGAACTTTGAAGAG
SPPDA091-07 Group 1 TANZANIA    AGCCCTAAGTGGGTGGTAAACTCCATCTAAGGCTAAATATTACCGCGAGACCGATAGCGAACAAGTACCGTGAGGGAAAGTTGAAAAGAACTTTGAAGAG
SPPDA001-07 Group 1 TANZANIA    AGCCCTAAGTGGGTGGTAAACTCCATCTAAGGCTAAATATTACCGCGAGACCGATAGCGAACAAGTACCGTGAGGGAAAGTTGAAAAGAACTTTGAAGAG
SPHPA185-07 Group 1 C.AFR.REP.  AGCCCTAAGTGGGTGGTAAACTCCATCTAAGGCTAAATATTACCGCGAGACCGATAGCGAACAAGTACCGTGAGGGAAAGTTGAAAAGAACTTTGAAGAG
GWORC183-07 Group 2 AUSTRALIA   ------------------AACTCCATCTAAGGCTAAATATTACCGCGAGACCGATAGCGAACAAGTACCGTGAGGGAAAGTTGAAAAGAACTTTGAAGAG
GWORD041-07 Group 2 AUSTRALIA   ------------------AACTCCATCTAAGGCTAAATATTACCGCGAGACCGATAGCGAACAAGTACCGTGAGGGAAAGTTGAAAAGAACTTTGAAGAG
GWOR1744-07 Group 2 AUSTRALIA   -------------------------------------TATTACCGCGAGACCGATAGCGAACAAGTACCGTGAGGGAAAGTTGAAAAGAACTTTGAAGAG
SPUEB032-07 Group 2 MALUKU      AGCCCTAAGTGGGTGGTAAACTCCATCTAAGGCTAAATATTACCGCGAGACCGATAGCGAACAAGTACCGTGAGGGAAAGTTGAAAAGAACTTTGAAGAG
SOWD069-06 Group 2 TAHITI      --------------------CTCCATCTAAGGCTAAATATTACCGCGAGACCGATAGCGAACAAGTACCGTGAGGGAAAGTTGAAAAGAACTTTGAAGAG
SOWD070-06 Group 2 TAHITI      -------------------------------------------------------------------------------------------TTTGAAGAG
SPUEB034-07 Group 2 NEW CALED.  ----------GGGTGGTAAACTCCATCTAAGGCTAAATATTACCGCGAGACCGATAGCGAACAAGTACCGTGAGGGAAAGTTGAAAAGAACTTTGAAGAG

                                 110       120       130       140       150       160       170       180       190       200
                        ....|....|....|....|....|....|....|....|....|....|....|....|....|....|....|....|....|....|....|
SPHYE007-08 Group 1 FRANCE      AGAGTTCAAGAGTACGTGAAACCGTTCAGGGGTAAACCTGCGAAACTCGAATGAACGAACGGAGAGATTCATCGTCATTCCGGGGCGTACGTGAGCGCTC
SPPBA360-07 Group 1 MADAGASCAR  AGAGTTCAAGAGTACGTGAAACCGTTCAGGGGTAAACCTGCGAAACTCGAATGAACGAACGGAGAGATTCATCGTCATTCCGGGGCGTACGTGAGCGCTC
SSDA224-06 Group 1 TANZANIA     AGAGTTCAAGAGTACGTGAAACCGTTCAGGGGTAAACCTGCGAAACTCGAATGAACGAACGGAGAGATTCATCGTCATTCCGGGGCGTACGTGAGCGCTC
SPPDA091-07 Group 1 TANZANIA    AGAGTTCAAGAGTACGTGAAACCGTTCAGGGGTAAACCTGCGAAACTCGAATGAACGAACGGAGAGATTCATCGTCATTCCGGGGCGTACGTGAGCGCTC
SPPDA001-07 Group 1 TANZANIA    AGAGTTCAAGAGTACGTGAAACCGTTCAGGGGTAAACCTGCGAAACTCGAATGAACGAACGGAGAGATTCATCGTCATTCCGGGGCGTACGTGAGCGCTC
SPHPA185-07 Group 1 C.AFR.REP.  AGAGTTCAAGAGTACGTGAAACCGTTCAGGGGTAAACCTGCGAAACTCGAATGAACGAACGGAGAGATTCATCGTCATTCCGGGGCGTACGTGAGCGCTC
GWORC183-07 Group 2 AUSTRALIA   AGAGTTCAAGAGTACGTGAAACCGTTCAGGGGTAAACCTGCGAAACTCGAATGAACGAACGGAGAGATTCATCGTCATTCCGGGGCGTACGTGAGCGCTC
GWORD041-07 Group 2 AUSTRALIA   AGAGTTCAAGAGTACGTGAAACCGTTCAGGGGTAAACCTGCGAAACTCGAATGAACGAACGGAGAGATTCATCGTCATTCCGGGGCGTACGTGAGCGCTC
GWOR1744-07 Group 2 AUSTRALIA   AGAGTTCAAGAGTACGTGAAACCGTTCAGGGGTAAACCTGCGAAACTCGAATGAACGAACGGAGAGATTCATCGTCATTCCGGGGCGTACGTGAGCGCTC
SPUEB032-07 Group 2 MALUKU      AGAGTTCAAGAGTACGTGAAACCGTTCAGGGGTAAACCTGCGAAACTCGAATGAACGAACGGAGAGATTCATCGTCATTCCGGGGCGTACGTGAGCGCTC
SOWD069-06 Group 2 TAHITI      AGAGTTCAAGAGTACGTGAAACCGTTCAGGGGTAAACCTGCGAAACTCGAATGAACGAACGGAGAGATTCATCGTCATTCCGGGGCGTACGTGAGCGCTC
SOWD070-06 Group 2 TAHITI      AGAGTTCAAGAGTACGTGAAACCGTTCAGGGGTAAACCTGCGAAACTCGAATGAACGAACGGAGAGATTCATCGTCATTCCGGGGCGTACGTGAGCGCTC
SPUEB034-07 Group 2 NEW CALED.  AGAGTTCAAGAGTACGTGAAACCGTTCAGGGGTAAACCTGCGAAACTCGAATGAACGAACGGAGAGATTCATCGTCATTCCGGGGCGTACGTGAGCGCTC

                                 210       220       230       240       250       260       270       280       290       300
                        ....|....|....|....|....|....|....|....|....|....|....|....|....|....|....|....|....|....|....|
SPHYE007-08 Group 1 FRANCE      CACGATGCGGATGGTACGACTCGTGCGTTTCCGTTCACACGGGACGTTCACGTCTAACGTTCTCGGACGGCGTGCACTTCTCTCTAAGTAATACATCGCG
SPPBA360-07 Group 1 MADAGASCAR  CACGATGCGGATGGTACGACTCGTGCGTTTCCGTTCACACGGGACGTTCACGTCTAACGTTCTCGGACGGCGTGCACTTCTCTCTAAGTAATACATCGCG
SSDA224-06 Group 1 TANZANIA     CACGATGCGGATGGTACGACTCGTGCGTTTCCGTTCACACGGGACGTTCACGTCTAACGTTCTCGGACGGCGTGCACTTCTCTCTAAGTAATACATCGCG
SPPDA091-07 Group 1 TANZANIA    CACGATGCGGATGGTACGACTCGTGCGTTTCCGTTCACACGGGACGTTCACGTCTAACGTTCTCGGACGGCGTGCACTTCTCTCTAAGTAATACATCGCG
SPPDA001-07 Group 1 TANZANIA    CACGATGCGGATGGTACGACTCGTGCGTTTCCGTTCACACGGGACGTTCACGTCTAACGTTCTCGGACGGCGTGCACTTCTCTCTAAGTAATACATCGCG
SPHPA185-07 Group 1 C.AFR.REP.  CACGATGCGGATGGTACGACTCGTGCGTTTCCGTTCACACGGGACGTTCACGTCTAACGTTCTCGGACGGCGTGCACTTCTCTCTAAGTAATACATCGCG
GWORC183-07 Group 2 AUSTRALIA   CACGATGCGGATGGTACGACTCGTGCGTTTCCGTTCACACGGGACGTTCACGTCTAACGTTCTCGGACGGCGTGCACTTCTCTCTAAGTAATACATCGCG
GWORD041-07 Group 2 AUSTRALIA   CACGATGCGGATGGTACGACTCGTGCGTTTCCGTTCACACGGGACGTTCACGTCTAACGTTCTCGGACGGCGTGCACTTCTCTCTAAGTAATACATCGCG
GWOR1744-07 Group 2 AUSTRALIA   CACGATGCGGATGGTACGACTCGTGCGTTTCCGTTCACACGGGACGTTCACGTCTAACGTTCTCGGACGGCGTGCACTTCTCTCTAAGTAATACATCGCG
SPUEB032-07 Group 2 MALUKU      CACGATGCGGATGGTACGACTCGTGCGTTTCCGTTCACACGGGACGTTCACGTCTAACGTTCTCGGACGGCGTGCACTTCTCTCTAAGTAATACATCGCG
SOWD069-06 Group 2 TAHITI      CACGATGCGGATGGTACGACTCGTGCGTTTCCGTTCACACGGGACGTTCACGTCTAACGTTCTCGGACGGCGTGCACTTCTCTCTAAGTAATACATCGCG
SOWD070-06 Group 2 TAHITI      CACGATGCGGATGGTACGACTCGTGCGTTTCCGTTCACACGGGACGTTCACGTCTAACGTTCTCGGACGGCGTGCACTTCTCTCTAAGTAATACATCGCG
SPUEB034-07 Group 2 NEW CALED.  CACGATGCGGATGGTACGACTCGTGCGTTTCCGTTCACACGGGACGTTCACGTCTAACGTTCTCGGACGGCGTGCACTTCTCTCTAAGTAATACATCGCG
```

```
                                       310       320       330       340       350       360       370       380       390       400
                                  ....|....|....|....|....|....|....|....|....|....|....|....|....|....|....|....|....|....|....|....|
SPHYE007-08 Group 1 FRANCE        ACCCGTTCCGTGTTCTGAAATTATCTAAGCGCCTTACGGGAGCCCAGGACGTGCACTTTAACCGGTACACGTTCGCGGACCGTATTTGATCGGGTAAGCT
SPPBA360-07 Group 1 MADAGASCAR    ACCCGTTCCGTGTTCTGAAATTATCTAAGCGCCTTACGGGAGCCCAGGACGTGCACTTTAACCGGTACACGTTCGCGGACCGTATTTGATCGGGTAAGCT
SSDA224-06 Group 1 TANZANIA      ACCCGTTCCGTGTTCTGAAATTATCTAAGCGCCTTACGGGAGCCCAGGACGTGCACTTTAACCGGTACACGTTCGCGGACCGTATTTGATCGGGTAAGCT
SPPDA091-07 Group 1 TANZANIA     ACCCGTTCCGTGTTCTGAAATTATCTAAGCGCCTTACGGGAGCCCAGGACGTGCACTTTAACCGGTACACGTTCGCGGACCGTATTTGATCGGGTAAGCT
SPPDA001-07 Group 1 TANZANIA     ACCCGTTCCGTGTTCTGAAATTATCTAAGCGCCTTACGGGAGCCCAGGACGTGCNCTTTAACCGGTACACGTTCGCGGACCGTATTTGATCGGGTAAGCT
SPHPA185-07 Group 1 C.AFR.REP.   ACCCGTTCCGTGTTCTGAAATTATCTAAGCGCCTTACGGGAGCCCAGGACGTGCACTTTAACCGGTACACGTTCGCGGACCGTATTTGATCGGGTAAGCT
GWORC183-07 Group 2 AUSTRALIA    ACCCGTTCCGTGTTCTCAAATTATCTAAGCGCCTTACGGGAGCCCAGGACGTGCACTTTAACCGGTACACGTTCGCGGACCGTATTTGATCGGGTAAGCT
GWORD041-07 Group 2 AUSTRALIA    ACCCGTTCCGTGTTCTCAAATTATCTAAGCGCCTTACGGGAGCCCAGGACGTGCACTTTAACCGGTACACGTTCGCGGACCGTATTTGATCGGGTAAGCT
GWOR1744-07 Group 2 AUSTRALIA    ACCCGTTCCGTGTTCTCAAATTATCTAAGCGCCTTACGGGAGCCCAGGACGTGCACTTTAACCGGTACACGTTCGCGGACCGTATTTGATCGGGTAAGCT
SPUEB032-07 Group 2 MALUKU       ACCCGTTCCGTGTTCTCAAATTATCTAAGCGCCTTACGGGAGCCCAGGACGTGCACTTTAACCGGTACACGTTCGCGGACCGTATTTGATCGGGTAAGCT
SOWD069-06 Group 2 TAHITI       ACCCGTTCCGTGTTCTCAAATTATCTAAGCGCCTTACGGGAGCCCAGGACGTGCACTTTAACCGGTACACGTTCGCGGACCGTATTTGATCGGGTAAGCT
SOWD070-06 Group 2 TAHITI       ACCCGTTCCGTGTTCTCAAATTATCTAAGCGCCTTACGGGAGCCCAGGACGTGCACTTTAACCGGTACACGTTCGCGGACCGTATTTGATCGGGTAAGCT
SPUEB034-07 Group 2 NEW CALED.   ACCCGTTCCGTGTTCTCAAATTATCTAAGCGCCTTACGGGAGCCCAGGACGTGCACTTTAACCGGTACACGTTCGCGGACCGTATTTGATCGGGTAAGCT
```

```
                                       410       420       430       440       450       460       470       480       490       500
                                  ....|....|....|....|....|....|....|....|....|....|....|....|....|....|....|....|....|....|....|....|
SPHYE007-08 Group 1 FRANCE        GCCGATAATTGCGGGACGGTATTTAAAATATGACGACGCGCACGCGTATTGAACGCGTCCGGCCCGACGCAAGACAACGTCGTCTCACGATTCCCGTCCT
SPPBA360-07 Group 1 MADAGASCAR    GCCGATAATTGCGGGACGGTATTTAAAATATGACGACGCGCACGCGTATTGAACGCGTCCGGCCCGACGCAAGACAACGTCGTCTCACGATTCCCGTCCT
SSDA224-06 Group 1 TANZANIA      GCCGATAATTGCGGGACGGTATTTAAAATATGACGACGCGCACGCGTATTGAACGCGTCCGGCCCGACGCAAGACAACGTCGTCTCACGATTCCCGTCCT
SPPDA091-07 Group 1 TANZANIA     GCCGATAATTGCGGGACGGTATTTAAAATATGACGACGCGCACGCGTATTGAACGCGTCCGGCCCGACGCAAGACAACGTCGTCTCACGATTCCCGTCCT
SPPDA001-07 Group 1 TANZANIA     GCCGATAATTGCGGGACGGTATTTAAAATATGACGACGCGCACGCGTATTGAACGCGTCCGGCCCGACGCAAGACAACGTCGTCTCACGATTCCCGTCCT
SPHPA185-07 Group 1 C.AFR.REP.   GCCGATAATTGCGGGACGGTATTTAAAATATGACGACGCGCACGCGTATTGAACGCGTCCGGCCCGACGCAAGACAACGTCGTCTCACGATTCCCGTCCT
GWORC183-07 Group 2 AUSTRALIA    GCCGATAATTGCGGGACGGTATTTAAAATATGACGACGCGCACGCGTATTGAACGCGTCCGGCCCGACGCAAGACAACGTCGTCTTACGATTCCCGTCCT
GWORD041-07 Group 2 AUSTRALIA    GCCGATAATTGCGGGACGGTATTTAAAATATGACGACGCGCACGCGTATTGAACGCGTCCGGCCCGACGCAAGACAACGTCGTCTTACGATTCCCGTCCT
GWOR1744-07 Group 2 AUSTRALIA    GCCGATAATTGCGGGACGGTATTTAAAATATGACGACGCGCACGCGTATTGAACGCGTCCGGCCCGACGCAAGACAACGTCGTCTTACGATTCCCGTCCT
SPUEB032-07 Group 2 MALUKU       GCCGATAATTGCGGGACGGTATTTAAAATATGACGACGCGCACGCGTATTGAACGCGTCCGGCCCGACGCAAGACAACGTCGTCTTACGATTCCCGTCCT
SOWD069-06 Group 2 TAHITI       GCCGATAATTGCGGGACGGTATTTAAAATATGACGACGCGCACGCGTATTGAACGCGTCCGGCCCGACGCAAGACAACGTCGTCTTACGATTCCCGTCCT
SOWD070-06 Group 2 TAHITI       GCCGATAATTGCGGGACGGTATTTAAAATATGACGACGCGCACGCGTATTGAACGCGTCCGGCCCGACGCAAGACAACGTCGTCTTACGATTCCCGTCCT
SPUEB034-07 Group 2 NEW CALED.   GCCGATAATTGCGGGACGGTATTTAAAATATGACGACGCGCACGCGTATTGAACGCGTCCGGCCCGACGCAAGACAACGTCGTCTTACGATTCCCGTCCT
```

```
                                       510       520       530       540       550       560       570       580       590       600
                                  ....|....|....|....|....|....|....|....|....|....|....|....|....|....|....|....|....|....|....|....|
SPHYE007-08 Group 1 FRANCE        GCCCGAGTGCGAGCGGGGATCCGGCGCGTCTGTTGTCGCCGCCGTGCCGTCTCGGACTTGTGCGCGTCGTTTTGAAATTTGTATGTCTGCGATGATTCAG
SPPBA360-07 Group 1 MADAGASCAR    GCCCGAGTGCGAGCGGGGATCCGGCGCGTCTGTTGTCGCCGCCGTGCCGTCTCGGACTTGTGCGCGTCGTTTTGAAATTTGTATGTCTGCGATGATTCAG
SSDA224-06 Group 1 TANZANIA      GCCCGAGTGCGAGCGGGGATCCGGCGCGTCTGTTGTCGCCGCCGTGCCGTCTCGGACTTGTGCGCGTCGTTTTGAAATTTGTATGTCTGCGATGATTCAG
SPPDA091-07 Group 1 TANZANIA     GCCCGAGTGCGAGCGGGGATCCGGCGCGTCTGTTGTCGCCGCCGTGCCGTCTCGGACTTGTGCGCGTCGTTTTGAAATTTGTATGTCTGCGATGATTCAG
SPPDA001-07 Group 1 TANZANIA     GCCCGAGTGCGAGCGGGGATCCGGCGCGTCTGTTGTCGCCGCCGTGCCGTCTCGGACTTGTGCGCGTCGTTTTGAAATTTGTATGT--------------
SPHPA185-07 Group 1 C.AFR.REP.   GCCCGAGTGCGAGCGGGGATCCGGCGCGTCTGTTGTCGCCGCCGTGCCGTCTCGGACTTGTGCGCGTCGTTTTGAAATTTGTATGTCTGCGATGATTCAG
GWORC183-07 Group 2 AUSTRALIA    GCCCAAGTGCGAGCGGGGATCCGGCGCGTCTGTTGTCGCCGCCGTGCCGTCTCGGACTTGTGCGCGTCGTTTTGAAATTTGTATGTCTGCGATGAT----
GWORD041-07 Group 2 AUSTRALIA    GCCCAAGTGCGAGCGGGGATCCGGCGCGTCTGTTGTCGCCGCCGTGCCGTCTCGGACTTGTGCGCGTCGTTTTGAAATTTGTATGTCTGCGATGAT----
GWOR1744-07 Group 2 AUSTRALIA    GCCCAAGTGCGAGCGGGGATCCGGCGCGTCTGTTGTCGCCGCCGTGCCGTCTCGGACTTGTGCGCGTCGTTTTGAAATTTGTATGTCTGCGATGAT----
SPUEB032-07 Group 2 MALUKU       GCCCAAGTGCGAGCGGGGATCCGGCGCGTCTGTTGTCGCCGCCGTGCCGTCTCGGACTTGTGCGCGTCGTTTTGAAATTTGTATGTCTGCGATGATTCAG
SOWD069-06 Group 2 TAHITI       GCCCAAGTGCGAGCGGGGATCCGGCGCGTCTGTTGTCGCCGCCGTGCCGTCTCGGACTTGTGCGCGTCGTTTTGAAATTTGTATGTCTGCGATGAT----
SOWD070-06 Group 2 TAHITI       GCCCAAGTGCGAGCGGGGATCCGGCGCGTCTGTTGTCGCCGCCGTGCCGTCTCGGACTTGTGCGCGTCGTTTTGAAATTTGTATGTC-------------
SPUEB034-07 Group 2 NEW CALED.   GCCCAAGTGCGAGCGGGGATCCGGCGCGTCTGTTGTCGCCGCCGTGCCGTCTCGGACTTGTGCGCGTCGTTTTGAAATTTGTATGTCTGCGATGATTCAG
```

```
                                             610
                                   ....|....|....|
SPHYE007-08 Group 1 FRANCE         TTTCGGGCACTCGCA
SPPBA360-07 Group 1 MADAGASCAR     TTTCGGGCACTCGCA
SSDA224-06 Group 1 TANZANIA        TTTCGGGCACTCGCA
SPPDA091-07 Group 1 TANZANIA       TTTCGGGCACTCGCA
SPPDA001-07 Group 1 TANZANIA       ---------------
SPHPA185-07 Group 1 C.AFR.REP.     TTTCGGGCACTCGCA
GWORC183-07 Group 2 AUSTRALIA      ---------------
GWORD041-07 Group 2 AUSTRALIA      ---------------
GWOR1744-07 Group 2 AUSTRALIA      ---------------
SPUEB032-07 Group 2 MALUKU         TTTCGGGCACTCGCA
SOWD069-06 Group 2 TAHITI          ---------------
SOWD070-06 Group 2 TAHITI          ---------------
SPUEB034-07 Group 2 NEW CALED.     TTTCGGGCACTCGCA
```
