# Supplementary material for: Australian Sphingidae – DNA Barcodes Challenge Current Species Boundaries and Distributions
Source: PLoS One. 2014 Jul 2;9(7):e101108. doi: 10.1371/journal.pone.0101108 (PMC4079597; doi:10.1371/journal.pone.0101108)

Fig. S3: Overlooked diversity within *Acosmeryx anceus.* Geographical distribution of the two DNA barcode clusters revealed after analysis of genetic distances (Neighbour Joining tree based on K2P-distances) between representatives of *Acosmeryx anceus*. This split is further confirmed by a nuclear marker (28S rDNA) and by morphological differences in adult and immature stages. The two species are found in the same area in Queensland, but inhabit different habitats: rainforest (green cluster) and eucalyptus woodland (red cluster).

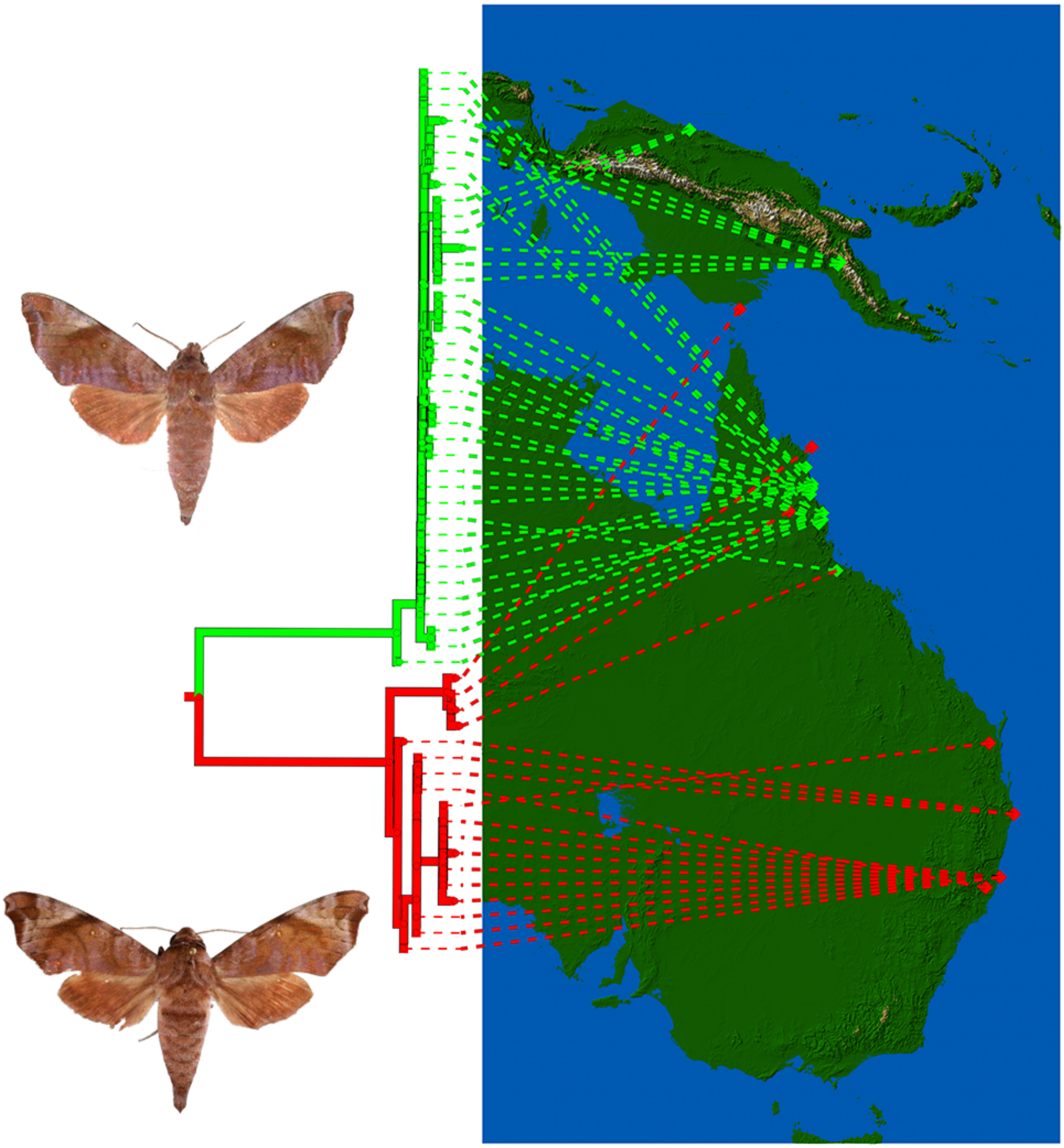

Supplement: Figure S3 — Overlooked diversity within Acosmeryx anceus. (PDF) [file pone.0101108.s003.pdf]
