# Supplementary material for: Australian Sphingidae – DNA Barcodes Challenge Current Species Boundaries and Distributions
Source: PLoS One. 2014 Jul 2;9(7):e101108. doi: 10.1371/journal.pone.0101108 (PMC4079597; doi:10.1371/journal.pone.0101108)

Figure S4: Maximum Parsimony tree (strict consensus of 5 trees) for 38 records of *Hippotion brennus* and 3 records of *H. joiceyi* (568 bp analyzed, 37 informative characters, CI=0.77, RI=0.97). Terminals are identified by their ProcessID code on BOLD (www.boldsystems.org), and scale is expressed in units of the number of changes over the whole sequence, with branch lengths calculated using the average pathway method (Nei & Kumar, 2000). Red dots point Australian records. Bootstrap values (500 replicates) above 75 are given for relevant nodes. While the two species are barely separated by their DNA barcodes, they can be readily distinguished upon habitus with *H. joiceyi* specimens lacking the patches of large white scales on the dorsal surface of the abdomen. This tree and the moth images also illustrate the possible validity of the currently synonymized Australian *H. johanna*, as well as the possible existence of two overlooked species in West Papua (Indonesia) and in the Moluccas.

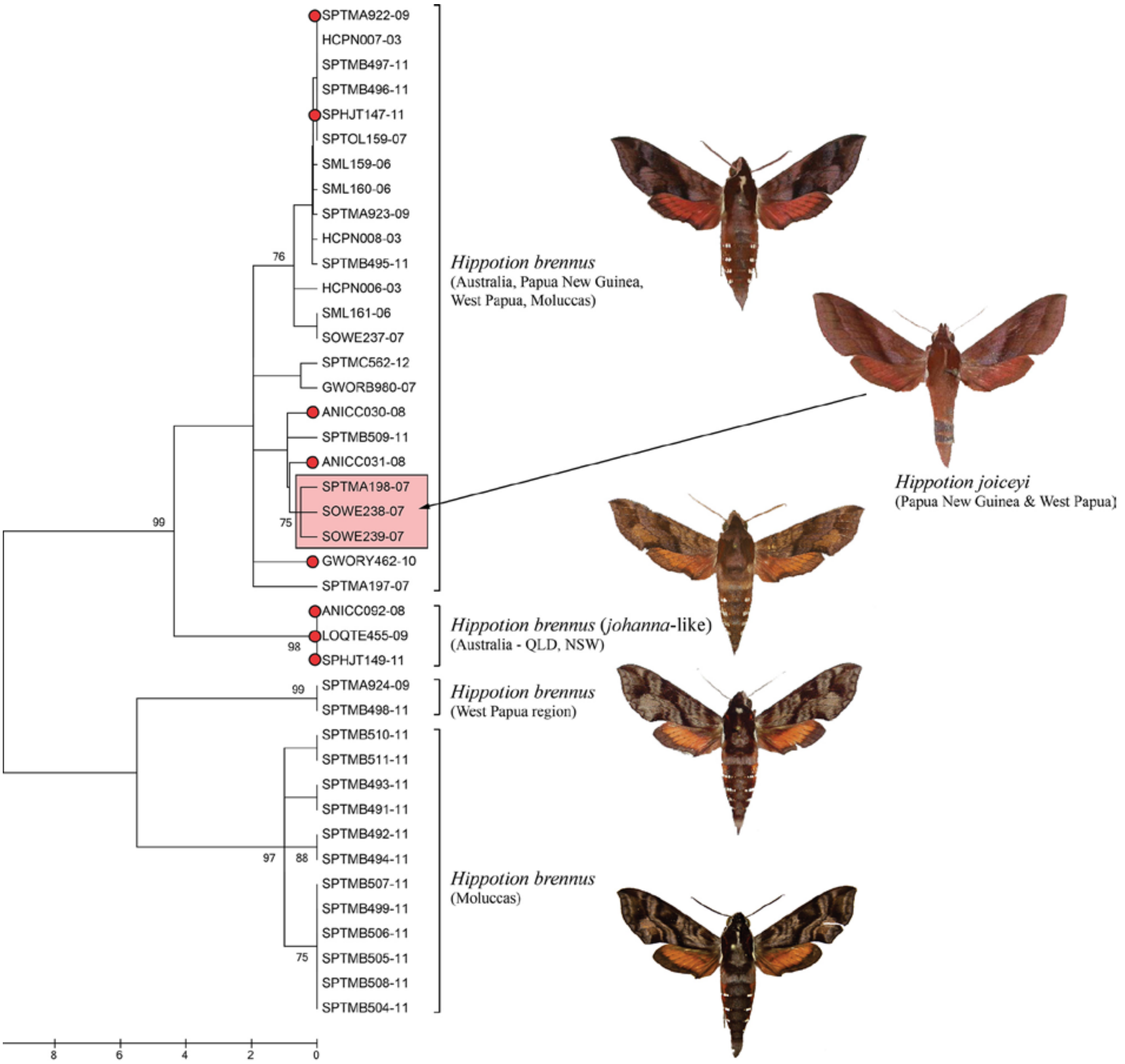

Supplement: Figure S4 — Consensus tree (MP) for records of Hippotion brennus and H. joiceyi. (PDF) [file pone.0101108.s004.pdf]
