# Supplementary material for: Australian Sphingidae – DNA Barcodes Challenge Current Species Boundaries and Distributions
Source: PLoS One. 2014 Jul 2;9(7):e101108. doi: 10.1371/journal.pone.0101108 (PMC4079597; doi:10.1371/journal.pone.0101108)
Supplement: Figure S5 — NJ phylogram for Australian and non-Australian records. (PDF) [file pone.0101108.s005.pdf]

Figure S5: Phylogram derived from the Neighbour Joining analysis of K2P distances by BOLD of 1789 sphingid records including 1054 Australian samples and 735 samples of conspecifics, co-subspecifics and closely related species. Red boxes highlight those cases where records from outside Australia are more than 2% distant from Australian specimens considered conspecific (for monotypic species) or co-subspecific (for polytypic species). Green boxes highlight these cases where heterospecific records from Australia and from outside the continent are lumped together. Records for holotypes in the *Psilogramma menephron* complex are in red characters.

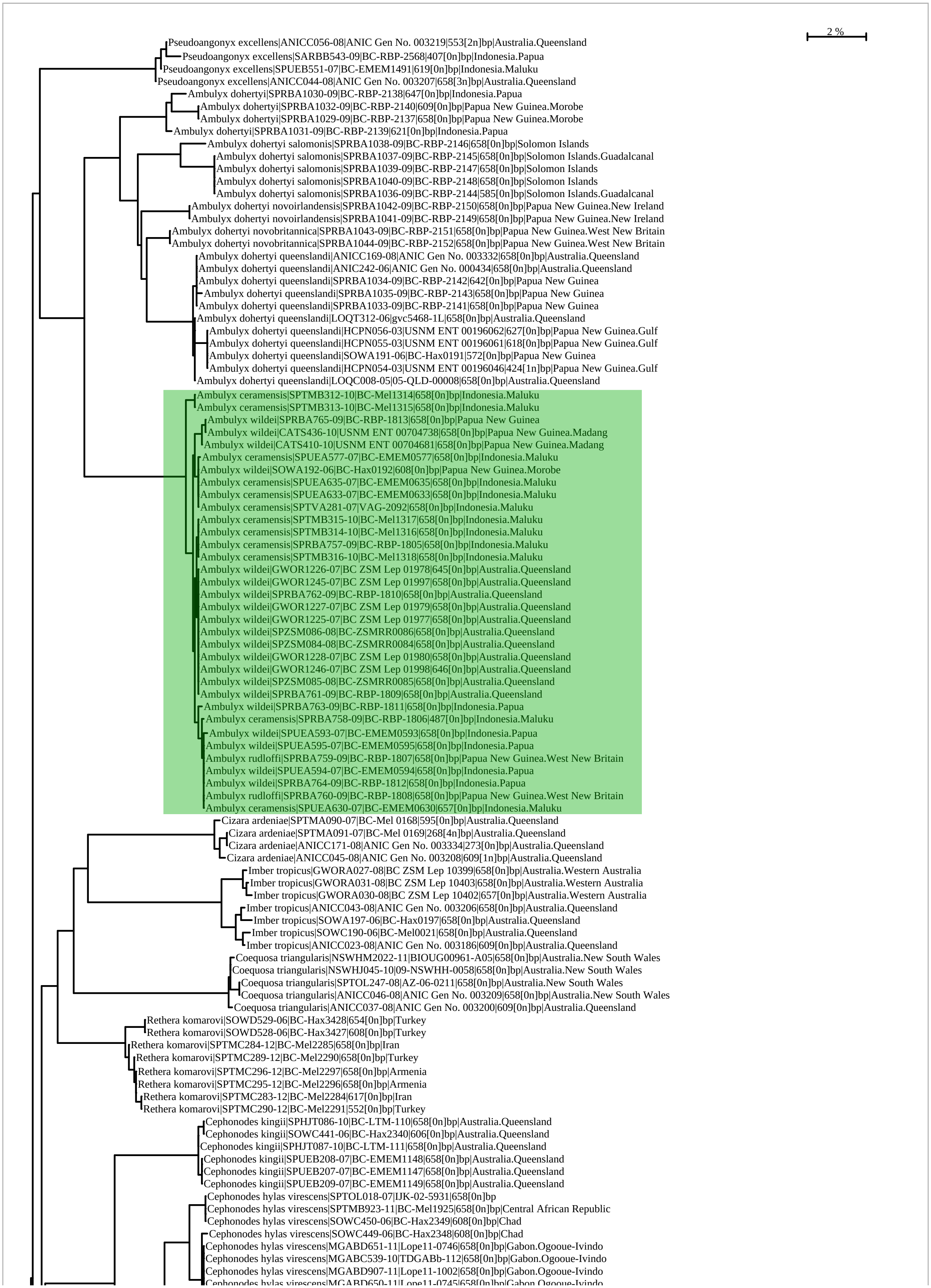
2 %
Pseudoangonyx excellens|ANICC056-08|ANIC Gen No. 003219|553[2n]bp|Australia.Queensland
Pseudoangonyx excellens|SARBB543-09|BC-RBP-2568|407[0n]bp|Indonesia.Papua
Pseudoangonyx excellens|SPUEB551-07|BC-EMEM1491|619[0n]bp|Indonesia.Maluku
Pseudoangonyx excellens|ANICC044-08|ANIC Gen No. 003207|658[3n]bp|Australia.Queensland
Ambulyx dohertyi|SPRBA1030-09|BC-RBP-2138|647[0n]bp|Indonesia.Papua
Ambulyx dohertyi|SPRBA1032-09|BC-RBP-2140|609[0n]bp|Papua New Guinea.Morobe
Ambulyx dohertyi|SPRBA1029-09|BC-RBP-2137|658[0n]bp|Papua New Guinea.Morobe
Ambulyx dohertyi|SPRBA1031-09|BC-RBP-2139|621[0n]bp|Indonesia.Papua
Ambulyx dohertyi salomonis|SPRBA1038-09|BC-RBP-2146|658[0n]bp|Solomon Islands
Ambulyx dohertyi salomonis|SPRBA1037-09|BC-RBP-2145|658[0n]bp|Solomon Islands.Guadalcanal
Ambulyx dohertyi salomonis|SPRBA1039-09|BC-RBP-2147|658[0n]bp|Solomon Islands
Ambulyx dohertyi salomonis|SPRBA1040-09|BC-RBP-2148|658[0n]bp|Solomon Islands
Ambulyx dohertyi salomonis|SPRBA1036-09|BC-RBP-2144|585[0n]bp|Solomon Islands.Guadalcanal
Ambulyx dohertyi novoirlandensis|SPRBA1042-09|BC-RBP-2150|658[0n]bp|Papua New Guinea.New Ireland
Ambulyx dohertyi novoirlandensis|SPRBA1041-09|BC-RBP-2149|658[0n]bp|Papua New Guinea.New Ireland
Ambulyx dohertyi novobritannica|SPRBA1043-09|BC-RBP-2151|658[0n]bp|Papua New Guinea.West New Britain
Ambulyx dohertyi novobritannica|SPRBA1044-09|BC-RBP-2152|658[0n]bp|Papua New Guinea.West New Britain
Ambulyx dohertyi queenslandi|ANICC169-08|ANIC Gen No. 003332|658[0n]bp|Australia.Queensland
Ambulyx dohertyi queenslandi|ANIC242-06|ANIC Gen No. 000434|658[0n]bp|Australia.Queensland
Ambulyx dohertyi queenslandi|SPRBA1034-09|BC-RBP-2142|642[0n]bp|Papua New Guinea
Ambulyx dohertyi queenslandi|SPRBA1035-09|BC-RBP-2143|658[0n]bp|Papua New Guinea
Ambulyx dohertyi queenslandi|SPRBA1033-09|BC-RBP-2141|658[0n]bp|Papua New Guinea
Ambulyx dohertyi queenslandi|LOQT312-06|gvc5468-1L|658[0n]bp|Australia.Queensland
Ambulyx dohertyi queenslandi|HCPN056-03|USNM ENT 00196062|627[0n]bp|Papua New Guinea.Gulf
Ambulyx dohertyi queenslandi|HCPN055-03|USNM ENT 00196061|618[0n]bp|Papua New Guinea.Gulf
Ambulyx dohertyi queenslandi|SOWA191-06|BC-Hax0191|572[0n]bp|Papua New Guinea
Ambulyx dohertyi queenslandi|HCPN054-03|USNM ENT 00196046|424[1n]bp|Papua New Guinea.Gulf
Ambulyx dohertyi queenslandi|LOQC008-05|05-QLD-00008|658[0n]bp|Australia.Queensland
Ambulyx ceramensis|SPTMB312-10|BC-Mel1314|658[0n]bp|Indonesia.Maluku
Ambulyx ceramensis|SPTMB313-10|BC-Mel1315|658[0n]bp|Indonesia.Maluku
Ambulyx wildei|SPRBA765-09|BC-RBP-1813|658[0n]bp|Papua New Guinea
Ambulyx wildei|CATS436-10|USNM ENT 00704738|658[0n]bp|Papua New Guinea.Madang
Ambulyx wildei|CATS410-10|USNM ENT 00704681|658[0n]bp|Papua New Guinea.Madang
Ambulyx ceramensis|SPUEA577-07|BC-EMEM0577|658[0n]bp|Indonesia.Maluku
Ambulyx wildei|SOWA192-06|BC-Hax0192|608[0n]bp|Papua New Guinea.Morobe
Ambulyx ceramensis|SPUEA635-07|BC-EMEM0635|658[0n]bp|Indonesia.Maluku
Ambulyx ceramensis|SPUEA633-07|BC-EMEM0633|658[0n]bp|Indonesia.Maluku
Ambulyx ceramensis|SPTVA281-07|VAG-2092|658[0n]bp|Indonesia.Maluku
Ambulyx ceramensis|SPTMB315-10|BC-Mel1317|658[0n]bp|Indonesia.Maluku
Ambulyx ceramensis|SPTMB314-10|BC-Mel1316|658[0n]bp|Indonesia.Maluku
Ambulyx ceramensis|SPRBA757-09|BC-RBP-1805|658[0n]bp|Indonesia.Maluku
Ambulyx ceramensis|SPTMB316-10|BC-Mel1318|658[0n]bp|Indonesia.Maluku
Ambulyx wildei|GWOR1226-07|BC ZSM Lep 01978|645[0n]bp|Australia.Queensland
Ambulyx wildei|GWOR1245-07|BC ZSM Lep 01997|658[0n]bp|Australia.Queensland
Ambulyx wildei|SPRBA762-09|BC-RBP-1810|658[0n]bp|Australia.Queensland
Ambulyx wildei|GWOR1227-07|BC ZSM Lep 01979|658[0n]bp|Australia.Queensland
Ambulyx wildei|GWOR1225-07|BC ZSM Lep 01977|658[0n]bp|Australia.Queensland
Ambulyx wildei|SPZSM086-08|BC-ZSMRR0086|658[0n]bp|Australia.Queensland
Ambulyx wildei|SPZSM084-08|BC-ZSMRR0084|658[0n]bp|Australia.Queensland
Ambulyx wildei|GWOR1228-07|BC ZSM Lep 01980|658[0n]bp|Australia.Queensland
Ambulyx wildei|GWOR1246-07|BC ZSM Lep 01998|646[0n]bp|Australia.Queensland
Ambulyx wildei|SPZSM085-08|BC-ZSMRR0085|658[0n]bp|Australia.Queensland
Ambulyx wildei|SPRBA761-09|BC-RBP-1809|658[0n]bp|Australia.Queensland
Ambulyx wildei|SPRBA763-09|BC-RBP-1811|658[0n]bp|Indonesia.Papua
Ambulyx ceramensis|SPRBA758-09|BC-RBP-1806|487[0n]bp|Indonesia.Maluku
Ambulyx wildei|SPUEA593-07|BC-EMEM0593|658[0n]bp|Indonesia.Papua
Ambulyx wildei|SPUEA595-07|BC-EMEM0595|658[0n]bp|Indonesia.Papua
Ambulyx rudloffi|SPRBA759-09|BC-RBP-1807|658[0n]bp|Papua New Guinea.West New Britain
Ambulyx wildei|SPUEA594-07|BC-EMEM0594|658[0n]bp|Indonesia.Papua
Ambulyx wildei|SPRBA764-09|BC-RBP-1812|658[0n]bp|Indonesia.Papua
Ambulyx rudloffi|SPRBA760-09|BC-RBP-1808|658[0n]bp|Papua New Guinea.West New Britain
Ambulyx ceramensis|SPUEA630-07|BC-EMEM0630|657[0n]bp|Indonesia.Maluku
Cizara ardeniae|SPTMA090-07|BC-Mel 0168|595[0n]bp|Australia.Queensland
Cizara ardeniae|SPTMA091-07|BC-Mel 0169|268[4n]bp|Australia.Queensland
Cizara ardeniae|ANICC171-08|ANIC Gen No. 003334|273[0n]bp|Australia.Queensland
Cizara ardeniae|ANICC045-08|ANIC Gen No. 003208|609[1n]bp|Australia.Queensland
Imber tropicus|GWORA027-08|BC ZSM Lep 10399|658[0n]bp|Australia.Western Australia
Imber tropicus|GWORA031-08|BC ZSM Lep 10403|658[0n]bp|Australia.Western Australia
Imber tropicus|GWORA030-08|BC ZSM Lep 10402|657[0n]bp|Australia.Western Australia
Imber tropicus|ANICC043-08|ANIC Gen No. 003206|658[0n]bp|Australia.Queensland
Imber tropicus|SOWA197-06|BC-Hax0197|658[0n]bp|Australia.Queensland
Imber tropicus|SOWC190-06|BC-Mel0021|658[0n]bp|Australia.Queensland
Imber tropicus|ANICC023-08|ANIC Gen No. 003186|609[0n]bp|Australia.Queensland
Coequosa triangularis|NSWHM2022-11|BIOUG00961-A05|658[0n]bp|Australia.New South Wales
Coequosa triangularis|NSWHJ045-10|09-NSWHH-0058|658[0n]bp|Australia.New South Wales
Coequosa triangularis|SPTOL247-08|AZ-06-0211|658[0n]bp|Australia.New South Wales
Coequosa triangularis|ANICC046-08|ANIC Gen No. 003209|658[0n]bp|Australia.New South Wales
Coequosa triangularis|ANICC037-08|ANIC Gen No. 003200|609[0n]bp|Australia.Queensland
Rethera komarovi|SOWD529-06|BC-Hax3428|654[0n]bp|Turkey
Rethera komarovi|SOWD528-06|BC-Hax3427|608[0n]bp|Turkey
Rethera komarovi|SPTMC284-12|BC-Mel2285|658[0n]bp|Iran
Rethera komarovi|SPTMC289-12|BC-Mel2290|658[0n]bp|Turkey
Rethera komarovi|SPTMC296-12|BC-Mel2297|658[0n]bp|Armenia
Rethera komarovi|SPTMC295-12|BC-Mel2296|658[0n]bp|Armenia
Rethera komarovi|SPTMC283-12|BC-Mel2284|617[0n]bp|Iran
Rethera komarovi|SPTMC290-12|BC-Mel2291|552[0n]bp|Turkey
Cephonodes kingii|SPHJT086-10|BC-LTM-110|658[0n]bp|Australia.Queensland
Cephonodes kingii|SOWC441-06|BC-Hax2340|606[0n]bp|Australia.Queensland
Cephonodes kingii|SPHJT087-10|BC-LTM-111|658[0n]bp|Australia.Queensland
Cephonodes kingii|SPUEB208-07|BC-EMEM1148|658[0n]bp|Australia.Queensland
Cephonodes kingii|SPUEB207-07|BC-EMEM1147|658[0n]bp|Australia.Queensland
Cephonodes kingii|SPUEB209-07|BC-EMEM1149|658[0n]bp|Australia.Queensland
Cephonodes hylas virescens|SPTOL018-07|IJK-02-5931|658[0n]bp
Cephonodes hylas virescens|SPTMB923-11|BC-Mel1925|658[0n]bp|Central African Republic
Cephonodes hylas virescens|SOWC450-06|BC-Hax2349|608[0n]bp|Chad
Cephonodes hylas virescens|SOWC449-06|BC-Hax2348|608[0n]bp|Chad
Cephonodes hylas virescens|MGABD651-11|Lope11-0746|658[0n]bp|Gabon.Ogooue-Ivindo
Cephonodes hylas virescens|MGABC539-10|TDGABb-112|658[0n]bp|Gabon.Ogooue-Ivindo
Cephonodes hylas virescens|MGABD907-11|Lope11-1002|658[0n]bp|Gabon.Ogooue-Ivindo
Cephonodes hylas virescens|MGABD650-11|Lope11-0745|658[0n]bp|Gabon.Ogooue-Ivindo

Cephonodes hylas virescens|[illegible]
Cephonodes hylas virescens|MGABC539-10|TDGABb-112|658[0n]bp|Gabon.Ogooue-Ivindo
Cephonodes hylas virescens|MGABD907-11|Lope11-1002|658[0n]bp|Gabon.Ogooue-Ivindo
Cephonodes hylas virescens|MGABD650-11|Lope11-0745|658[0n]bp|Gabon.Ogooue-Ivindo
Cephonodes hylas virescens|SPTMB922-11|BC-Mel1924|658[0n]bp|Central African Republic
Cephonodes hylas virescens|PMANL1483-11|USNM ENT 00719779|658[0n]bp|Kenya
Cephonodes hylas australis|SPHJT089-10|BC-LTM-113|658[0n]bp|Australia.Queensland
Cephonodes hylas australis|SPHJT088-10|BC-LTM-112|658[0n]bp|Australia.Queensland
Cephonodes hylas australis|ANICC070-08|ANIC Gen No. 003233|626[1n]bp|Australia.Queensland
Cephonodes hylas australis|ANICC069-08|ANIC Gen No. 003232|591[0n]bp|Australia.Northern Territory
Cephonodes hylas melanogaster|SPUEB196-07|BC-EMEM1136|609[0n]bp|Indonesia.Sulawesi Selatan
Cephonodes hylas melanogaster|SPUEB197-07|BC-EMEM1137|658[0n]bp|Indonesia.Sulawesi Barat
Cephonodes hylas melanogaster|SPUEB198-07|BC-EMEM1138|658[0n]bp|Indonesia.Sulawesi Selatan
Cephonodes hylas|SPTOL113-07|AYK-04-0171|658[0n]bp|Taiwan.Nantou County
Cephonodes hylas|SPUEB202-07|BC-EMEM1142|658[0n]bp|Thailand.Chiang Mai
Cephonodes hylas|SPUEB200-07|BC-EMEM1140|658[0n]bp|Vietnam
Cephonodes hylas|SPUEB201-07|BC-EMEM1141|658[0n]bp|Japan
Cephonodes hylas|SPUEB203-07|BC-EMEM1143|658[0n]bp|Thailand.Chiang Mai
Cephonodes janus|ANICC074-08|ANIC Gen No. 003237|658[0n]bp|Australia.Queensland
Cephonodes janus|ANICC073-08|ANIC Gen No. 003236|609[0n]bp|Australia.Queensland
Cephonodes xanthus|SOWC442-06|BC-Hax2341|608[0n]bp|Japan
Cephonodes xanthus|SPTMB627-11|BC-Mel1629|658[1n]bp|Japan.Okinawa
Cephonodes picus|SOWC443-06|BC-Hax2342|605[0n]bp|India
Cephonodes picus|SOWC444-06|BC-Hax2343|608[0n]bp|India
Cephonodes picus|SPHJT091-10|BC-LTM-115|658[0n]bp|Australia.Queensland
Cephonodes picus|LTOL262-07|MF-05-0013|658[0n]bp|Palau
Cephonodes picus|LOQTI746-12|gvc17799-1L|658[0n]bp|Australia.Queensland
Cephonodes picus|ANICC068-08|ANIC Gen No. 003231|609[0n]bp|Australia.Queensland
Cephonodes picus|SPUEB204-07|BC-EMEM1144|658[0n]bp|Philippines
Cephonodes picus|SPUEB205-07|BC-EMEM1145|658[0n]bp|Philippines
Cephonodes picus|SPHJT090-10|BC-LTM-114|658[0n]bp|Australia.Queensland
Cephonodes picus|LOQ381-04|04HBL004381|658[0n]bp|Australia.Queensland
Cephonodes picus|SPUEB206-07|BC-EMEM1146|658[0n]bp|Philippines
Angonyx testacea|SARBB547-09|BC-RBP-2572|574[0n]bp|China.Yunnan
Angonyx testacea|SOWE189-07|BC-Hax4088|658[0n]bp|Indonesia
Angonyx testacea|SARBB546-09|BC-RBP-2571|658[0n]bp|Laos
Angonyx testacea|SARBB1031-09|BC-RBP 3056|658[0n]bp|Philippines.Palawan
Angonyx testacea|SARBB1029-09|BC-RBP 3054|658[0n]bp|China.Hainan
Angonyx testacea|SARBB1026-09|BC-RBP 3051|658[0n]bp|India.Assam
Angonyx testacea|SARBB1439-10|BC-RBP-2574.1|658[0n]bp|Indonesia
Angonyx testacea|LTOL334-07|AYK-04-0304|658[0n]bp|Taiwan.Pingtung County
Angonyx testacea|SARBB1017-09|BC-RBP 3042|658[0n]bp|Philippines.Leyte
Angonyx testacea|SARBB1030-09|BC-RBP 3055|658[0n]bp|Philippines.Palawan
Angonyx testacea|SARBB1037-09|BC-RBP 3062|658[0n]bp|Philippines.Negros Occidental
Angonyx testacea|SARBB1032-09|BC-RBP 3057|658[0n]bp|Philippines.Palawan
Angonyx testacea|SARBB1022-09|BC-RBP 3047|658[0n]bp|India.Andaman and Nicobar
Angonyx testacea|SARBB1034-09|BC-RBP 3059|658[0n]bp|Philippines.Negros Occidental
Angonyx testacea|SARBB1027-09|BC-RBP 3052|658[0n]bp|Vietnam.South Vietnam
Angonyx testacea|SARBB1446-10|BC-RBP-2572.1|658[0n]bp|China.Yunnan
Angonyx testacea|SARBB1035-09|BC-RBP 3060|658[0n]bp|Philippines.Leyte
Angonyx testacea|SARBB1028-09|BC-RBP 3053|658[0n]bp|China.Yunnan
Angonyx testacea|SOWE190-07|BC-Hax4089|658[0n]bp|Vietnam
Angonyx testacea|SARBB1036-09|BC-RBP 3061|658[0n]bp|Philippines.Leyte
Angonyx testacea|SOWE188-07|BC-Hax4087|630[0n]bp|Malaysia.Sabah
Angonyx testacea|SPTOL048-07|AYK-04-0243|658[0n]bp|Taiwan.Pingtung County
Angonyx testacea|SARBB1025-09|BC-RBP 3050|658[0n]bp|India.Assam
Angonyx testacea|SARBB1039-09|BC-RBP 3064|658[0n]bp|Indonesia.Sulawesi Selatan
Angonyx testacea|SARBB1021-09|BC-RBP 3046|658[0n]bp|Indonesia.Nusa Tenggara Timur
Angonyx testacea|SOWE186-07|BC-Hax4085|658[0n]bp|Indonesia.Sulawesi Utara
Angonyx testacea|SARBB1038-09|BC-RBP 3063|658[0n]bp|Indonesia.Sulawesi Selatan
Angonyx testacea|SARBB1020-09|BC-RBP 3045|658[0n]bp|Indonesia
Angonyx testacea|SARBB545-09|BC-RBP-2570|658[0n]bp|Bhutan
Angonyx testacea|SARBB1044-09|BC-RBP 3069|658[0n]bp|Indonesia.Papua
Angonyx testacea|SARBB1043-09|BC-RBP 3068|658[0n]bp|Indonesia.Papua
Angonyx testacea|SPTMB620-11|BC-Mel1622|658[0n]bp|Indonesia
Angonyx testacea|SPTMC055-11|BC-Mel2056|658[0n]bp|Indonesia
Angonyx meeki|SARBB1430-10|BC-RBP-2562.1|658[0n]bp|Solomon Islands
Angonyx meeki|SARBB1431-10|BC-RBP-2563.1|658[0n]bp|Solomon Islands
Angonyx meeki|SPTMA041-07|BC-Mel 0119b|635[0n]bp|Solomon Islands.Guadalcanal
Angonyx meeki|SARBB532-09|BC-RBP-2557|658[0n]bp|Solomon Islands.Guadalcanal
Angonyx meeki|SARBB1428-10|BC-RBP-2558.1|658[0n]bp|Solomon Islands.Guadalcanal
Angonyx meeki|SARBB1429-10|BC-RBP-2559.1|658[0n]bp|Solomon Islands
Angonyx meeki|SARBB1442-10|BC-RBP-2561.1|658[0n]bp|Solomon Islands.Makira
Angonyx meeki|SARBB535-09|BC-RBP-2560|658[0n]bp|Solomon Islands
Angonyx papuana|ANICC055-08|ANIC Gen No. 003218|294[0n]bp|Australia.Queensland
Angonyx papuana|SARBB1435-10|BC-RBP-2567.1|658[0n]bp|Papua New Guinea
Angonyx papuana|SARBB1432-10|BC-RBP-2564.1|658[0n]bp|Papua New Guinea.West New Britain
Angonyx papuana|SARBB1434-10|BC-RBP-2566.1|658[0n]bp|Papua New Guinea
Angonyx papuana|SARBB1433-10|BC-RBP-2565.1|658[0n]bp|Papua New Guinea.West New Britain
Angonyx papuana|SPTMC521-12|BC-Mel2522|658[0n]bp|Indonesia
Angonyx papuana|GWORG363-08|BC ZSM Lep 08479|658[0n]bp|Australia.Queensland
Angonyx papuana|SPTMC520-12|BC-Mel2521|633[0n]bp|Indonesia
Angonyx papuana|LTOL325-07|JP-05-0763|658[0n]bp|Australia.Queensland
Angonyx papuana|ANICC054-08|ANIC Gen No. 003217|285[0n]bp|Australia.Queensland
Angonyx papuana|SPTMA043-07|BC-Mel 0121b|657[3n]bp|Indonesia.Papua
Cizara sculpta|SOWD383-06|BC-Hax3282|658[0n]bp|Laos
Cizara sculpta|SOWD382-06|BC-Hax3281|625[0n]bp|Laos
Cizara sculpta|SOWD381-06|BC-Hax3280|625[0n]bp|Laos
Coequosa australasiae|SPHYE141-09|BC-EST0517|632[0n]bp|Australia.New South Wales
Coequosa australasiae|ANICC189-08|BC-Roug1149|608[0n]bp|Australia.New South Wales
Coequosa australasiae|ANICC175-08|ANIC Gen No. 003338|658[0n]bp|Australia.New South Wales
Coequosa australasiae|IMLR1327-11|IM10-0071|658[0n]bp|Australia.Queensland
Coequosa australasiae|SPRBA754-09|BC-RBP-1797|658[0n]bp|Australia.Queensland
Coequosa australasiae|SOWA609-06|BC-Hax0602|608[0n]bp|Australia.Northern Territory
Coequosa australasiae|SOWA608-06|BC-Hax0601|608[0n]bp|Australia.Queensland
Coequosa australasiae|ANICC018-08|ANIC Gen No. 003181|658[0n]bp|Australia.Queensland
Psilogramma argos|SPHJT100-10|BC-LTM-124|658[0n]bp|Australia.Northern Territory
Psilogramma argos|ANICC072-08|ANIC Gen No. 003235|658[0n]bp|Australia.Northern Territory
Psilogramma argos|SPTMB093-09|BC-Mel 1105|658[0n]bp|Australia.Northern Territory
Leucomonia bethia|ANICC080-08|ANIC Gen No. 003243|658[0n]bp|Australia.Western Australia
Leucomonia bethia|ANICC079-08|ANIC Gen No. 003242|658[0n]bp|Australia.Western Australia
Leucomonia bethia|ANICC077-08|ANIC Gen No. 003240|609[0n]bp|Australia.Northern Territory
Leucomonia bethia|SOWD283-06|BC-Hax3182|607[0n]bp|Australia.Queensland

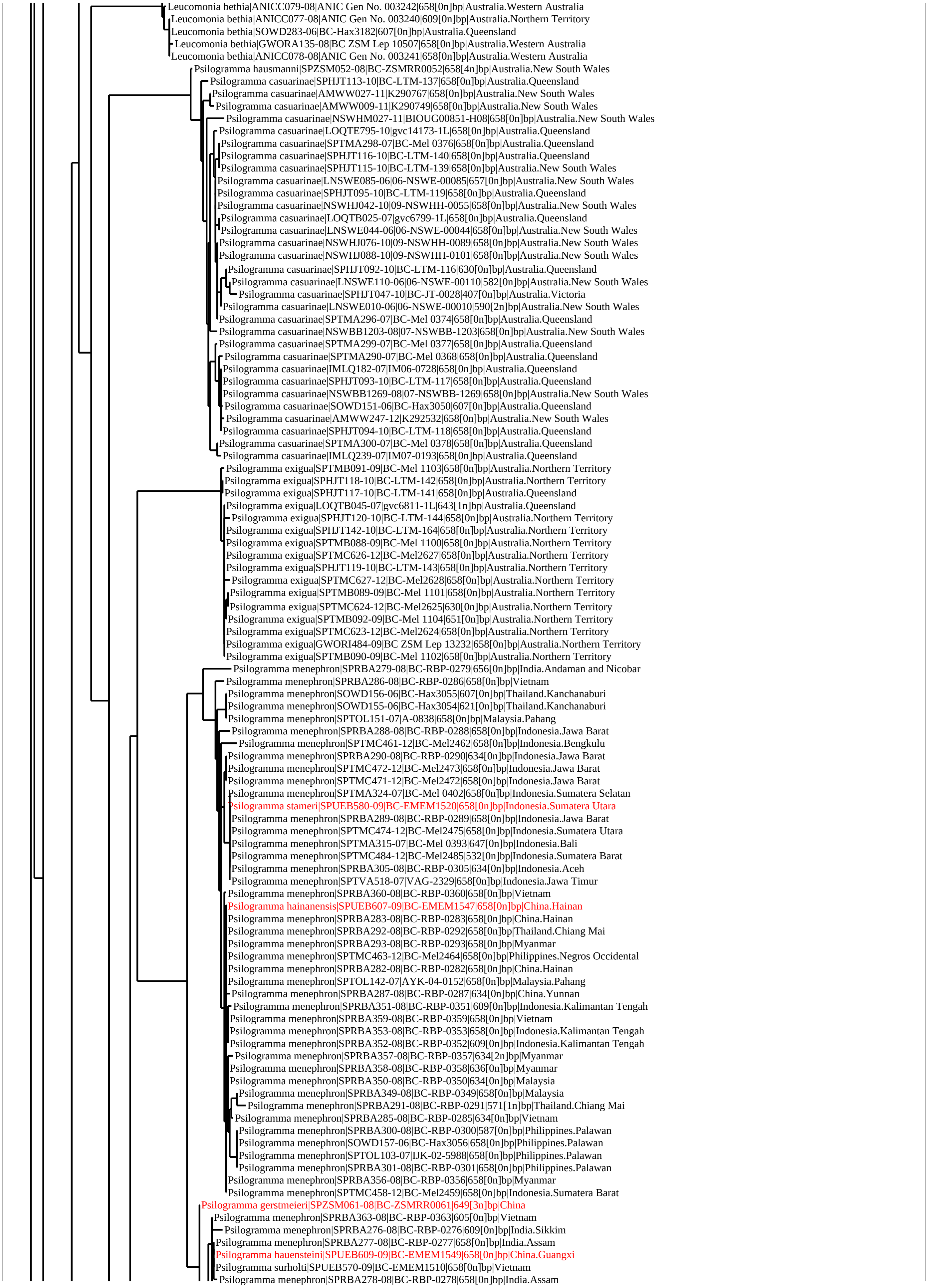
Leucomonia bethia|ANICC079-08|ANIC Gen No. 003242|658[0n]bp|Australia.Western Australia
Leucomonia bethia|ANICC077-08|ANIC Gen No. 003240|609[0n]bp|Australia.Northern Territory
Leucomonia bethia|SOWD283-06|BC-Hax3182|607[0n]bp|Australia.Queensland
Leucomonia bethia|GWORA135-08|BC ZSM Lep 10507|658[0n]bp|Australia.Western Australia
Leucomonia bethia|ANICC078-08|ANIC Gen No. 003241|658[0n]bp|Australia.Western Australia
Psilogramma hausmanni|SPZSM052-08|BC-ZSMRR0052|658[4n]bp|Australia.New South Wales
Psilogramma casuarinae|SPHJT113-10|BC-LTM-137|658[0n]bp|Australia.Queensland
Psilogramma casuarinae|AMWW027-11|K290767|658[0n]bp|Australia.New South Wales
Psilogramma casuarinae|AMWW009-11|K290749|658[0n]bp|Australia.New South Wales
Psilogramma casuarinae|NSWHM027-11|BIOUG00851-H08|658[0n]bp|Australia.New South Wales
Psilogramma casuarinae|LOQTE795-10|gvc14173-1L|658[0n]bp|Australia.Queensland
Psilogramma casuarinae|SPTMA298-07|BC-Mel 0376|658[0n]bp|Australia.Queensland
Psilogramma casuarinae|SPHJT116-10|BC-LTM-140|658[0n]bp|Australia.Queensland
Psilogramma casuarinae|SPHJT115-10|BC-LTM-139|658[0n]bp|Australia.New South Wales
Psilogramma casuarinae|LNSWE085-06|06-NSWE-00085|657[0n]bp|Australia.New South Wales
Psilogramma casuarinae|SPHJT095-10|BC-LTM-119|658[0n]bp|Australia.Queensland
Psilogramma casuarinae|NSWHJ042-10|09-NSWHH-0055|658[0n]bp|Australia.New South Wales
Psilogramma casuarinae|LOQTB025-07|gvc6799-1L|658[0n]bp|Australia.Queensland
Psilogramma casuarinae|LNSWE044-06|06-NSWE-00044|658[0n]bp|Australia.New South Wales
Psilogramma casuarinae|NSWHJ076-10|09-NSWHH-0089|658[0n]bp|Australia.New South Wales
Psilogramma casuarinae|NSWHJ088-10|09-NSWHH-0101|658[0n]bp|Australia.New South Wales
Psilogramma casuarinae|SPHJT092-10|BC-LTM-116|630[0n]bp|Australia.Queensland
Psilogramma casuarinae|LNSWE110-06|06-NSWE-00110|582[0n]bp|Australia.New South Wales
Psilogramma casuarinae|SPHJT047-10|BC-JT-0028|407[0n]bp|Australia.Victoria
Psilogramma casuarinae|LNSWE010-06|06-NSWE-00010|590[2n]bp|Australia.New South Wales
Psilogramma casuarinae|SPTMA296-07|BC-Mel 0374|658[0n]bp|Australia.Queensland
Psilogramma casuarinae|NSWBB1203-08|07-NSWBB-1203|658[0n]bp|Australia.New South Wales
Psilogramma casuarinae|SPTMA299-07|BC-Mel 0377|658[0n]bp|Australia.Queensland
Psilogramma casuarinae|SPTMA290-07|BC-Mel 0368|658[0n]bp|Australia.Queensland
Psilogramma casuarinae|IMLQ182-07|IM06-0728|658[0n]bp|Australia.Queensland
Psilogramma casuarinae|SPHJT093-10|BC-LTM-117|658[0n]bp|Australia.Queensland
Psilogramma casuarinae|NSWBB1269-08|07-NSWBB-1269|658[0n]bp|Australia.New South Wales
Psilogramma casuarinae|SOWD151-06|BC-Hax3050|607[0n]bp|Australia.Queensland
Psilogramma casuarinae|AMWW247-12|K292532|658[0n]bp|Australia.New South Wales
Psilogramma casuarinae|SPHJT094-10|BC-LTM-118|658[0n]bp|Australia.Queensland
Psilogramma casuarinae|SPTMA300-07|BC-Mel 0378|658[0n]bp|Australia.Queensland
Psilogramma casuarinae|IMLQ239-07|IM07-0193|658[0n]bp|Australia.Queensland
Psilogramma exigua|SPTMB091-09|BC-Mel 1103|658[0n]bp|Australia.Northern Territory
Psilogramma exigua|SPHJT118-10|BC-LTM-142|658[0n]bp|Australia.Northern Territory
Psilogramma exigua|SPHJT117-10|BC-LTM-141|658[0n]bp|Australia.Queensland
Psilogramma exigua|LOQTB045-07|gvc6811-1L|643[1n]bp|Australia.Queensland
Psilogramma exigua|SPHJT120-10|BC-LTM-144|658[0n]bp|Australia.Northern Territory
Psilogramma exigua|SPHJT142-10|BC-LTM-164|658[0n]bp|Australia.Northern Territory
Psilogramma exigua|SPTMB088-09|BC-Mel 1100|658[0n]bp|Australia.Northern Territory
Psilogramma exigua|SPTMC626-12|BC-Mel2627|658[0n]bp|Australia.Northern Territory
Psilogramma exigua|SPHJT119-10|BC-LTM-143|658[0n]bp|Australia.Northern Territory
Psilogramma exigua|SPTMC627-12|BC-Mel2628|658[0n]bp|Australia.Northern Territory
Psilogramma exigua|SPTMB089-09|BC-Mel 1101|658[0n]bp|Australia.Northern Territory
Psilogramma exigua|SPTMC624-12|BC-Mel2625|630[0n]bp|Australia.Northern Territory
Psilogramma exigua|SPTMB092-09|BC-Mel 1104|651[0n]bp|Australia.Northern Territory
Psilogramma exigua|SPTMC623-12|BC-Mel2624|658[0n]bp|Australia.Northern Territory
Psilogramma exigua|GWORI484-09|BC ZSM Lep 13232|658[0n]bp|Australia.Northern Territory
Psilogramma exigua|SPTMB090-09|BC-Mel 1102|658[0n]bp|Australia.Northern Territory
Psilogramma menephron|SPRBA279-08|BC-RBP-0279|656[0n]bp|India.Andaman and Nicobar
Psilogramma menephron|SPRBA286-08|BC-RBP-0286|658[0n]bp|Vietnam
Psilogramma menephron|SOWD156-06|BC-Hax3055|607[0n]bp|Thailand.Kanchanaburi
Psilogramma menephron|SOWD155-06|BC-Hax3054|621[0n]bp|Thailand.Kanchanaburi
Psilogramma menephron|SPTOL151-07|A-0838|658[0n]bp|Malaysia.Pahang
Psilogramma menephron|SPRBA288-08|BC-RBP-0288|658[0n]bp|Indonesia.Jawa Barat
Psilogramma menephron|SPTMC461-12|BC-Mel2462|658[0n]bp|Indonesia.Bengkulu
Psilogramma menephron|SPRBA290-08|BC-RBP-0290|634[0n]bp|Indonesia.Jawa Barat
Psilogramma menephron|SPTMC472-12|BC-Mel2473|658[0n]bp|Indonesia.Jawa Barat
Psilogramma menephron|SPTMC471-12|BC-Mel2472|658[0n]bp|Indonesia.Jawa Barat
Psilogramma menephron|SPTMA324-07|BC-Mel 0402|658[0n]bp|Indonesia.Sumatera Selatan
Psilogramma stameri|SPUEB580-09|BC-EMEM1520|658[0n]bp|Indonesia.Sumatera Utara
Psilogramma menephron|SPRBA289-08|BC-RBP-0289|658[0n]bp|Indonesia.Jawa Barat
Psilogramma menephron|SPTMC474-12|BC-Mel2475|658[0n]bp|Indonesia.Sumatera Utara
Psilogramma menephron|SPTMA315-07|BC-Mel 0393|647[0n]bp|Indonesia.Bali
Psilogramma menephron|SPTMC484-12|BC-Mel2485|532[0n]bp|Indonesia.Sumatera Barat
Psilogramma menephron|SPRBA305-08|BC-RBP-0305|634[0n]bp|Indonesia.Aceh
Psilogramma menephron|SPTVA518-07|VAG-2329|658[0n]bp|Indonesia.Jawa Timur
Psilogramma menephron|SPRBA360-08|BC-RBP-0360|658[0n]bp|Vietnam
Psilogramma hainanensis|SPUEB607-09|BC-EMEM1547|658[0n]bp|China.Hainan
Psilogramma menephron|SPRBA283-08|BC-RBP-0283|658[0n]bp|China.Hainan
Psilogramma menephron|SPRBA292-08|BC-RBP-0292|658[0n]bp|Thailand.Chiang Mai
Psilogramma menephron|SPRBA293-08|BC-RBP-0293|658[0n]bp|Myanmar
Psilogramma menephron|SPTMC463-12|BC-Mel2464|658[0n]bp|Philippines.Negros Occidental
Psilogramma menephron|SPRBA282-08|BC-RBP-0282|658[0n]bp|China.Hainan
Psilogramma menephron|SPTOL142-07|AYK-04-0152|658[0n]bp|Malaysia.Pahang
Psilogramma menephron|SPRBA287-08|BC-RBP-0287|634[0n]bp|China.Yunnan
Psilogramma menephron|SPRBA351-08|BC-RBP-0351|609[0n]bp|Indonesia.Kalimantan Tengah
Psilogramma menephron|SPRBA359-08|BC-RBP-0359|658[0n]bp|Vietnam
Psilogramma menephron|SPRBA353-08|BC-RBP-0353|658[0n]bp|Indonesia.Kalimantan Tengah
Psilogramma menephron|SPRBA352-08|BC-RBP-0352|609[0n]bp|Indonesia.Kalimantan Tengah
Psilogramma menephron|SPRBA357-08|BC-RBP-0357|634[2n]bp|Myanmar
Psilogramma menephron|SPRBA358-08|BC-RBP-0358|636[0n]bp|Myanmar
Psilogramma menephron|SPRBA350-08|BC-RBP-0350|634[0n]bp|Malaysia
Psilogramma menephron|SPRBA349-08|BC-RBP-0349|658[0n]bp|Malaysia
Psilogramma menephron|SPRBA291-08|BC-RBP-0291|571[1n]bp|Thailand.Chiang Mai
Psilogramma menephron|SPRBA285-08|BC-RBP-0285|634[0n]bp|Vietnam
Psilogramma menephron|SPRBA300-08|BC-RBP-0300|587[0n]bp|Philippines.Palawan
Psilogramma menephron|SOWD157-06|BC-Hax3056|658[0n]bp|Philippines.Palawan
Psilogramma menephron|SPTOL103-07|IJK-02-5988|658[0n]bp|Philippines.Palawan
Psilogramma menephron|SPRBA301-08|BC-RBP-0301|658[0n]bp|Philippines.Palawan
Psilogramma menephron|SPRBA356-08|BC-RBP-0356|658[0n]bp|Myanmar
Psilogramma menephron|SPTMC458-12|BC-Mel2459|658[0n]bp|Indonesia.Sumatera Barat
Psilogramma gerstmeieri|SPZSM061-08|BC-ZSMRR0061|649[3n]bp|China
Psilogramma menephron|SPRBA363-08|BC-RBP-0363|605[0n]bp|Vietnam
Psilogramma menephron|SPRBA276-08|BC-RBP-0276|609[0n]bp|India.Sikkim
Psilogramma menephron|SPRBA277-08|BC-RBP-0277|658[0n]bp|India.Assam
Psilogramma hauensteini|SPUEB609-09|BC-EMEM1549|658[0n]bp|China.Guangxi
Psilogramma surholti|SPUEB570-09|BC-EMEM1510|658[0n]bp|Vietnam
Psilogramma menephron|SPRBA278-08|BC-RBP-0278|658[0n]bp|India.Assam

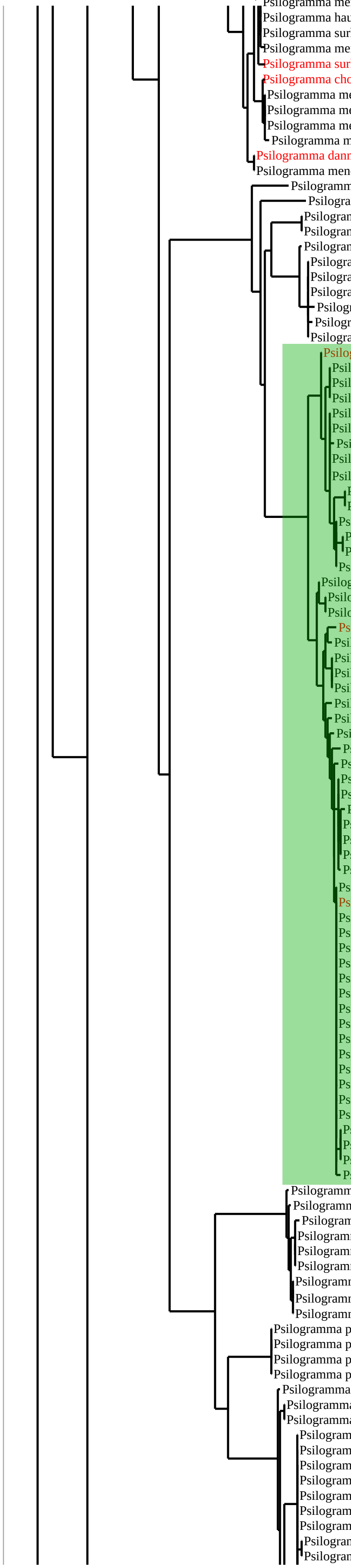

Psilogramma menephron|SPRBA277-08|BC-RBP-0277|658[0n]bp|India.Assam
Psilogramma hauensteini|SPUEB609-09|BC-EMEM1549|658[0n]bp|China.Guangxi
Psilogramma surholti|SPUEB570-09|BC-EMEM1510|658[0n]bp|Vietnam
Psilogramma menephron|SPRBA278-08|BC-RBP-0278|658[0n]bp|India.Assam
Psilogramma surholti|SPUEB569-09|BC-EMEM1509|658[0n]bp|Vietnam
Psilogramma choui|SPUEB605-09|BC-EMEM1545|658[0n]bp|China.Zhejiang
Psilogramma menephron|SPTMC476-12|BC-Mel2477|658[0n]bp|Vietnam
Psilogramma menephron|SPTVA521-07|VAG-2332|597[0n]bp|Thailand
Psilogramma menephron|SPTMA322-07|BC-Mel 0400|647[0n]bp|China.Yunnan
Psilogramma menephron|SPMNP346-07|BC-MNHNJP0236|658[0n]bp|China.Yunnan
Psilogramma danneri|SPZSM067-08|BC-ZSMRR0067|658[0n]bp|India
Psilogramma menephron|SPRBA367-08|BC-RBP-0367|609[0n]bp|Taiwan.Yilan County
Psilogramma menephron|SPUEB581-09|BC-EMEM1521|658[0n]bp|Indonesia.Maluku
Psilogramma menephron|SPUEB587-09|BC-EMEM1527|658[0n]bp|Indonesia.Maluku
Psilogramma menephron|SPUEB597-09|BC-EMEM1537|658[0n]bp|Indonesia.Maluku
Psilogramma menephron|SPUEB596-09|BC-EMEM1536|658[0n]bp|Indonesia.Maluku
Psilogramma menephron|SPTMA489-07|BC-Mel 0567|658[0n]bp|Indonesia.Maluku
Psilogramma menephron|SPRBA323-08|BC-RBP-0323|658[0n]bp|Indonesia.Maluku
Psilogramma menephron|SPRBA322-08|BC-RBP-0322|658[0n]bp|Indonesia.Sulawesi Tengah
Psilogramma menephron|SPTMA490-07|BC-Mel 0568|658[0n]bp|Indonesia.Sulawesi Tengah
Psilogramma menephron|SPTMC457-12|BC-Mel2458|573[0n]bp|Indonesia.Maluku
Psilogramma menephron|SPRBA324-08|BC-RBP-0324|636[0n]bp|Indonesia.Maluku
Psilogramma menephron|SPRBA321-08|BC-RBP-0321|658[0n]bp|Indonesia.Sulawesi Tengah
Psilogramma bartschereri|SPZSM066-08|BC-ZSMRR0066|658[2n]bp|Sri Lanka
Psilogramma menephron|SML153-06|USNM ENT 00209792|658[0n]bp|Papua New Guinea.Madang
Psilogramma menephron|SML157-06|USNM ENT 00209799|658[0n]bp|Papua New Guinea.Madang
Psilogramma menephron|SML150-06|USNM ENT 00209797|658[0n]bp|Papua New Guinea.Madang
Psilogramma menephron|SML158-06|USNM ENT 00209796|658[0n]bp|Papua New Guinea.Madang
Psilogramma menephron|SML156-06|USNM ENT 00210163|658[0n]bp|Papua New Guinea.Madang
Psilogramma menephron|SML154-06|USNM ENT 00209798|658[0n]bp|Papua New Guinea.Madang
Psilogramma menephron|SPRBA334-08|BC-RBP-0334|658[0n]bp|Papua New Guinea
Psilogramma menephron|SML155-06|USNM ENT 00209794|658[0n]bp|Papua New Guinea.Madang
Psilogramma menephron|SARBA157-08|BC-RBP-0627|614[0n]bp|Papua New Guinea
Psilogramma menephron|SARBA156-08|BC-RBP-0626|614[0n]bp|Papua New Guinea
Psilogramma menephron|SML152-06|USNM ENT 00209808|567[0n]bp|Papua New Guinea.Madang
Psilogramma menephron|GWORB987-07|BC ZSM Lep 02209|568[1n]bp|Papua New Guinea.Morobe
Psilogramma menephron|SML151-06|USNM ENT 00209811|567[0n]bp|Papua New Guinea.Madang
Psilogramma menephron|SPRBA336-08|BC-RBP-0336|609[0n]bp|Papua New Guinea
Psilogramma menephron|NSWBB1145-08|07-NSWBB-1145|658[0n]bp|Australia.New South Wales
Psilogramma menephron|LLISA218-06|06-NSWL-00218|658[0n]bp|Australia.New South Wales
Psilogramma menephron|LLISA598-06|06-NSWL-00598|658[0n]bp|Australia.New South Wales
Psilogramma anne|SPUEB594-09|BC-EMEM1534|649[0n]bp|Indonesia.Papua
Psilogramma menephron|SPRBA333-08|BC-RBP-0333|658[0n]bp|Indonesia.Papua
Psilogramma menephron|LTOL252-07|JP-05-0753|658[0n]bp|Australia.Queensland
Psilogramma menephron|LOQTD098-08|gvc8697-1L|658[0n]bp|Australia.Queensland
Psilogramma menephron|SPTMA307-07|BC-Mel 0385|645[0n]bp|Australia.Queensland
Psilogramma menephron|SPHJT137-10|BC-LTM-161|658[0n]bp|Australia.Queensland
Psilogramma menephron|GWORC230-07|BC ZSM Lep 02580|657[0n]bp|Australia.Queensland
Psilogramma menephron|GWORC231-07|BC ZSM Lep 02581|655[0n]bp|Australia.Queensland
Psilogramma menephron|HCPN070-03|USNM ENT 00196053|658[0n]bp|Papua New Guinea.Gulf
Psilogramma menephron|GWORB3265-08|BC ZSM Lep 05803|658[0n]bp|Australia.Queensland
Psilogramma menephron|SPRBA332-08|BC-RBP-0332|658[0n]bp|Indonesia.Papua
Psilogramma menephron|SPRBA337-08|BC-RBP-0337|609[0n]bp|Australia.Queensland
Psilogramma menephron|HCPN074-03|USNM ENT 00196077|639[0n]bp|Papua New Guinea.Gulf
Psilogramma menephron|GWORB850-07|BC ZSM Lep 01320|632[0n]bp|Australia.Queensland
Psilogramma menephron|SPRBA338-08|BC-RBP-0338|636[0n]bp|Australia.Queensland
Psilogramma menephron|GWORB867-07|BC ZSM Lep 01337|632[0n]bp|Australia.Queensland
Psilogramma menephron|SPTMA321-07|BC-Mel 0399|648[0n]bp|Indonesia.Papua
Psilogramma menephron|GWOR1242-07|BC ZSM Lep 01994|657[0n]bp|Australia.Queensland
Psilogramma gloriosa|SPUEB588-09|BC-EMEM1528|658[0n]bp|Australia.Queensland
Psilogramma menephron|LOQB330-05|Moth 014.03CC|658[0n]bp|Australia.Queensland
Psilogramma menephron|GWORB3266-08|BC ZSM Lep 05804|658[0n]bp|Australia.Queensland
Psilogramma menephron|IMLR1170-11|IM08-2832|658[0n]bp|Australia.Queensland
Psilogramma menephron|SPHJT111-10|BC-LTM-135|658[0n]bp|Australia.Queensland
Psilogramma menephron|LOQTI348-10|gvc15912-1L|658[0n]bp|Australia.Queensland
Psilogramma menephron|SPHJT003-09|BC-JT-20M|658[0n]bp|Australia.Queensland
Psilogramma menephron|LLISA219-06|06-NSWL-00219|658[0n]bp|Australia.New South Wales
Psilogramma menephron|LLISA600-06|06-NSWL-00600|658[0n]bp|Australia.New South Wales
Psilogramma menephron|LOQTD097-08|gvc8696-1L|658[0n]bp|Australia.Queensland
Psilogramma menephron|IMLQ184-07|IM06-0744|658[0n]bp|Australia.Queensland
Psilogramma menephron|GWORG362-08|BC ZSM Lep 08478|658[0n]bp|Australia.Queensland
Psilogramma menephron|LOQTE796-10|gvc14174-1L|658[0n]bp|Australia.Queensland
Psilogramma menephron|LOQTD099-08|gvc8698-1L|658[0n]bp|Australia.Queensland
Psilogramma menephron|LLISA223-06|06-NSWL-00223|658[0n]bp|Australia.New South Wales
Psilogramma menephron|LLISA224-06|06-NSWL-00224|658[0n]bp|Australia.New South Wales
Psilogramma menephron|NSWHJ020-10|09-NSWHH-0033|658[0n]bp|Australia.New South Wales
Psilogramma menephron|LLISA222-06|06-NSWL-00222|658[0n]bp|Australia.New South Wales
Psilogramma menephron|LOQTD039-08|gvc8636-1L|658[0n]bp|Australia.Queensland
Psilogramma maxmouldsi|SPUEB589-09|BC-EMEM1529|658[0n]bp|Australia.Queensland
Psilogramma maxmouldsi|IMLQ157-07|IM06-0592|658[0n]bp|Australia.Queensland
Psilogramma maxmouldsi|NSWHM2021-11|BIOUG00961-A04|658[0n]bp|Australia.New South Wales
Psilogramma maxmouldsi|LNSWE042-06|06-NSWE-00042|658[0n]bp|Australia.New South Wales
Psilogramma maxmouldsi|LNSWE071-06|06-NSWE-00071|658[0n]bp|Australia.New South Wales
Psilogramma maxmouldsi|LNSWE038-06|06-NSWE-00038|658[0n]bp|Australia.New South Wales
Psilogramma maxmouldsi|SPHJT001-09|BC-JT-24M|631[0n]bp|Australia.Queensland
Psilogramma maxmouldsi|LNSWE067-06|06-NSWE-00067|651[0n]bp|Australia.New South Wales
Psilogramma maxmouldsi|NSWHJ006-10|09-NSWHH-0019|658[0n]bp|Australia.New South Wales
Psilogramma penumbra|SPHJT123-10|BC-LTM-147|658[0n]bp|Australia.Northern Territory
Psilogramma penumbra|SPHJT121-10|BC-LTM-145|658[0n]bp|Australia.Northern Territory
Psilogramma penumbra|SPHJT122-10|BC-LTM-146|621[0n]bp|Australia.Northern Territory
Psilogramma penumbra|ANICC076-08|ANIC Gen No. 003239|658[0n]bp|Australia.Northern Territory
Psilogramma papuensis|SPHJT112-10|BC-LTM-136|658[0n]bp|Australia.Queensland
Psilogramma papuensis|GWORC280-07|BC ZSM Lep 02630|653[0n]bp|Australia.Queensland
Psilogramma papuensis|SPTMA301-07|BC-Mel 0379|658[0n]bp|Australia.Queensland
Psilogramma papuensis|SPTMA320-07|BC-Mel 0398|630[1n]bp|Indonesia.Papua
Psilogramma mastrigti|SPUEB602-09|BC-EMEM1542|658[0n]bp|Indonesia.Papua
Psilogramma papuensis|SPRBA258-08|BC-RBP-0258|658[0n]bp|Indonesia.Papua
Psilogramma papuensis|SPRBA257-08|BC-RBP-0257|658[0n]bp|Indonesia.Papua
Psilogramma papuensis|SPRBA254-08|BC-RBP-0254|639[1n]bp|Papua New Guinea
Psilogramma papuensis|SPRBA256-08|BC-RBP-0256|632[0n]bp|Papua New Guinea
Psilogramma papuensis|GWORB943-07|BC ZSM Lep 02165|503[0n]bp|Indonesia.Papua
Psilogramma papuensis|HCPN071-03|USNM ENT 00196054|574[3n]bp|Papua New Guinea.Gulf
Psilogramma papuensis|HCPN077-03|USNM ENT 00196080|639[6n]bp|Papua New Guinea.Gulf

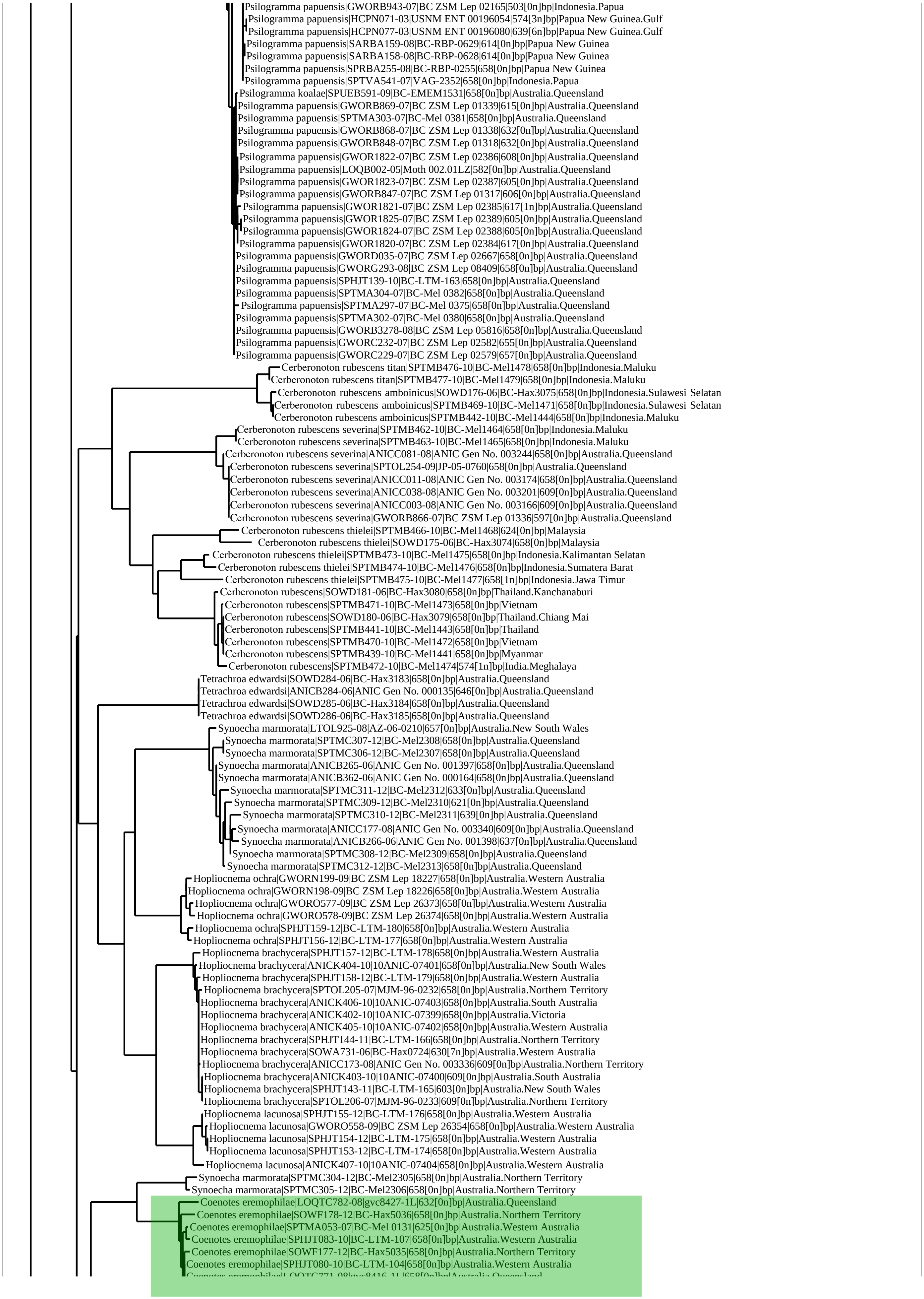
Psilogramma papuensis|GWORB943-07|BC ZSM Lep 02165|503[0n]bp|Indonesia.Papua
Psilogramma papuensis|HCPN071-03|USNM ENT 00196054|574[3n]bp|Papua New Guinea.Gulf
Psilogramma papuensis|HCPN077-03|USNM ENT 00196080|639[6n]bp|Papua New Guinea.Gulf
Psilogramma papuensis|SARBA159-08|BC-RBP-0629|614[0n]bp|Papua New Guinea
Psilogramma papuensis|SARBA158-08|BC-RBP-0628|614[0n]bp|Papua New Guinea
Psilogramma papuensis|SPRBA255-08|BC-RBP-0255|658[0n]bp|Papua New Guinea
Psilogramma papuensis|SPTVA541-07|VAG-2352|658[0n]bp|Indonesia.Papua
Psilogramma koalae|SPUEB591-09|BC-EMEM1531|658[0n]bp|Australia.Queensland
Psilogramma papuensis|GWORB869-07|BC ZSM Lep 01339|615[0n]bp|Australia.Queensland
Psilogramma papuensis|SPTMA303-07|BC-Mel 0381|658[0n]bp|Australia.Queensland
Psilogramma papuensis|GWORB868-07|BC ZSM Lep 01338|632[0n]bp|Australia.Queensland
Psilogramma papuensis|GWORB848-07|BC ZSM Lep 01318|632[0n]bp|Australia.Queensland
Psilogramma papuensis|GWOR1822-07|BC ZSM Lep 02386|608[0n]bp|Australia.Queensland
Psilogramma papuensis|LOQB002-05|Moth 002.01LZ|582[0n]bp|Australia.Queensland
Psilogramma papuensis|GWOR1823-07|BC ZSM Lep 02387|605[0n]bp|Australia.Queensland
Psilogramma papuensis|GWORB847-07|BC ZSM Lep 01317|606[0n]bp|Australia.Queensland
Psilogramma papuensis|GWOR1821-07|BC ZSM Lep 02385|617[1n]bp|Australia.Queensland
Psilogramma papuensis|GWOR1825-07|BC ZSM Lep 02389|605[0n]bp|Australia.Queensland
Psilogramma papuensis|GWOR1824-07|BC ZSM Lep 02388|605[0n]bp|Australia.Queensland
Psilogramma papuensis|GWOR1820-07|BC ZSM Lep 02384|617[0n]bp|Australia.Queensland
Psilogramma papuensis|GWORD035-07|BC ZSM Lep 02667|658[0n]bp|Australia.Queensland
Psilogramma papuensis|GWORG293-08|BC ZSM Lep 08409|658[0n]bp|Australia.Queensland
Psilogramma papuensis|SPHJT139-10|BC-LTM-163|658[0n]bp|Australia.Queensland
Psilogramma papuensis|SPTMA304-07|BC-Mel 0382|658[0n]bp|Australia.Queensland
Psilogramma papuensis|SPTMA297-07|BC-Mel 0375|658[0n]bp|Australia.Queensland
Psilogramma papuensis|SPTMA302-07|BC-Mel 0380|658[0n]bp|Australia.Queensland
Psilogramma papuensis|GWORB3278-08|BC ZSM Lep 05816|658[0n]bp|Australia.Queensland
Psilogramma papuensis|GWORC232-07|BC ZSM Lep 02582|655[0n]bp|Australia.Queensland
Psilogramma papuensis|GWORC229-07|BC ZSM Lep 02579|657[0n]bp|Australia.Queensland
Cerberonoton rubescens titan|SPTMB476-10|BC-Mel1478|658[0n]bp|Indonesia.Maluku
Cerberonoton rubescens titan|SPTMB477-10|BC-Mel1479|658[0n]bp|Indonesia.Maluku
Cerberonoton rubescens amboinicus|SOWD176-06|BC-Hax3075|658[0n]bp|Indonesia.Sulawesi Selatan
Cerberonoton rubescens amboinicus|SPTMB469-10|BC-Mel1471|658[0n]bp|Indonesia.Sulawesi Selatan
Cerberonoton rubescens amboinicus|SPTMB442-10|BC-Mel1444|658[0n]bp|Indonesia.Maluku
Cerberonoton rubescens severina|SPTMB462-10|BC-Mel1464|658[0n]bp|Indonesia.Maluku
Cerberonoton rubescens severina|SPTMB463-10|BC-Mel1465|658[0n]bp|Indonesia.Maluku
Cerberonoton rubescens severina|ANICC081-08|ANIC Gen No. 003244|658[0n]bp|Australia.Queensland
Cerberonoton rubescens severina|SPTOL254-09|JP-05-0760|658[0n]bp|Australia.Queensland
Cerberonoton rubescens severina|ANICC011-08|ANIC Gen No. 003174|658[0n]bp|Australia.Queensland
Cerberonoton rubescens severina|ANICC038-08|ANIC Gen No. 003201|609[0n]bp|Australia.Queensland
Cerberonoton rubescens severina|ANICC003-08|ANIC Gen No. 003166|609[0n]bp|Australia.Queensland
Cerberonoton rubescens severina|GWORB866-07|BC ZSM Lep 01336|597[0n]bp|Australia.Queensland
Cerberonoton rubescens thielei|SPTMB466-10|BC-Mel1468|624[0n]bp|Malaysia
Cerberonoton rubescens thielei|SOWD175-06|BC-Hax3074|658[0n]bp|Malaysia
Cerberonoton rubescens thielei|SPTMB473-10|BC-Mel1475|658[0n]bp|Indonesia.Kalimantan Selatan
Cerberonoton rubescens thielei|SPTMB474-10|BC-Mel1476|658[0n]bp|Indonesia.Sumatera Barat
Cerberonoton rubescens thielei|SPTMB475-10|BC-Mel1477|658[1n]bp|Indonesia.Jawa Timur
Cerberonoton rubescens|SOWD181-06|BC-Hax3080|658[0n]bp|Thailand.Kanchanaburi
Cerberonoton rubescens|SPTMB471-10|BC-Mel1473|658[0n]bp|Vietnam
Cerberonoton rubescens|SOWD180-06|BC-Hax3079|658[0n]bp|Thailand.Chiang Mai
Cerberonoton rubescens|SPTMB441-10|BC-Mel1443|658[0n]bp|Thailand
Cerberonoton rubescens|SPTMB470-10|BC-Mel1472|658[0n]bp|Vietnam
Cerberonoton rubescens|SPTMB439-10|BC-Mel1441|658[0n]bp|Myanmar
Cerberonoton rubescens|SPTMB472-10|BC-Mel1474|574[1n]bp|India.Meghalaya
Tetrachroa edwardsi|SOWD284-06|BC-Hax3183|658[0n]bp|Australia.Queensland
Tetrachroa edwardsi|ANICB284-06|ANIC Gen No. 000135|646[0n]bp|Australia.Queensland
Tetrachroa edwardsi|SOWD285-06|BC-Hax3184|658[0n]bp|Australia.Queensland
Tetrachroa edwardsi|SOWD286-06|BC-Hax3185|658[0n]bp|Australia.Queensland
Synoecha marmorata|LTOL925-08|AZ-06-0210|657[0n]bp|Australia.New South Wales
Synoecha marmorata|SPTMC307-12|BC-Mel2308|658[0n]bp|Australia.Queensland
Synoecha marmorata|SPTMC306-12|BC-Mel2307|658[0n]bp|Australia.Queensland
Synoecha marmorata|ANICB265-06|ANIC Gen No. 001397|658[0n]bp|Australia.Queensland
Synoecha marmorata|ANICB362-06|ANIC Gen No. 000164|658[0n]bp|Australia.Queensland
Synoecha marmorata|SPTMC311-12|BC-Mel2312|633[0n]bp|Australia.Queensland
Synoecha marmorata|SPTMC309-12|BC-Mel2310|621[0n]bp|Australia.Queensland
Synoecha marmorata|SPTMC310-12|BC-Mel2311|639[0n]bp|Australia.Queensland
Synoecha marmorata|ANICC177-08|ANIC Gen No. 003340|609[0n]bp|Australia.Queensland
Synoecha marmorata|ANICB266-06|ANIC Gen No. 001398|637[0n]bp|Australia.Queensland
Synoecha marmorata|SPTMC308-12|BC-Mel2309|658[0n]bp|Australia.Queensland
Synoecha marmorata|SPTMC312-12|BC-Mel2313|658[0n]bp|Australia.Queensland
Hopliocnema ochra|GWORN199-09|BC ZSM Lep 18227|658[0n]bp|Australia.Western Australia
Hopliocnema ochra|GWORN198-09|BC ZSM Lep 18226|658[0n]bp|Australia.Western Australia
Hopliocnema ochra|GWORO577-09|BC ZSM Lep 26373|658[0n]bp|Australia.Western Australia
Hopliocnema ochra|GWORO578-09|BC ZSM Lep 26374|658[0n]bp|Australia.Western Australia
Hopliocnema ochra|SPHJT159-12|BC-LTM-180|658[0n]bp|Australia.Western Australia
Hopliocnema ochra|SPHJT156-12|BC-LTM-177|658[0n]bp|Australia.Western Australia
Hopliocnema brachycera|SPHJT157-12|BC-LTM-178|658[0n]bp|Australia.Western Australia
Hopliocnema brachycera|ANICK404-10|10ANIC-07401|658[0n]bp|Australia.New South Wales
Hopliocnema brachycera|SPHJT158-12|BC-LTM-179|658[0n]bp|Australia.Western Australia
Hopliocnema brachycera|SPTOL205-07|MJM-96-0232|658[0n]bp|Australia.Northern Territory
Hopliocnema brachycera|ANICK406-10|10ANIC-07403|658[0n]bp|Australia.South Australia
Hopliocnema brachycera|ANICK402-10|10ANIC-07399|658[0n]bp|Australia.Victoria
Hopliocnema brachycera|ANICK405-10|10ANIC-07402|658[0n]bp|Australia.Western Australia
Hopliocnema brachycera|SPHJT144-11|BC-LTM-166|658[0n]bp|Australia.Northern Territory
Hopliocnema brachycera|SOWA731-06|BC-Hax0724|630[7n]bp|Australia.Western Australia
Hopliocnema brachycera|ANICC173-08|ANIC Gen No. 003336|609[0n]bp|Australia.Northern Territory
Hopliocnema brachycera|ANICK403-10|10ANIC-07400|609[0n]bp|Australia.South Australia
Hopliocnema brachycera|SPHJT143-11|BC-LTM-165|603[0n]bp|Australia.New South Wales
Hopliocnema brachycera|SPTOL206-07|MJM-96-0233|609[0n]bp|Australia.Northern Territory
Hopliocnema lacunosa|SPHJT155-12|BC-LTM-176|658[0n]bp|Australia.Western Australia
Hopliocnema lacunosa|GWORO558-09|BC ZSM Lep 26354|658[0n]bp|Australia.Western Australia
Hopliocnema lacunosa|SPHJT154-12|BC-LTM-175|658[0n]bp|Australia.Western Australia
Hopliocnema lacunosa|SPHJT153-12|BC-LTM-174|658[0n]bp|Australia.Western Australia
Hopliocnema lacunosa|ANICK407-10|10ANIC-07404|658[0n]bp|Australia.Western Australia
Synoecha marmorata|SPTMC304-12|BC-Mel2305|658[0n]bp|Australia.Northern Territory
Synoecha marmorata|SPTMC305-12|BC-Mel2306|658[0n]bp|Australia.Northern Territory
Coenotes eremophilae|LOQTC782-08|gvc8427-1L|632[0n]bp|Australia.Queensland
Coenotes eremophilae|SOWF178-12|BC-Hax5036|658[0n]bp|Australia.Northern Territory
Coenotes eremophilae|SPTMA053-07|BC-Mel 0131|625[0n]bp|Australia.Western Australia
Coenotes eremophilae|SPHJT083-10|BC-LTM-107|658[0n]bp|Australia.Western Australia
Coenotes eremophilae|SOWF177-12|BC-Hax5035|658[0n]bp|Australia.Northern Territory
Coenotes eremophilae|SPHJT080-10|BC-LTM-104|658[0n]bp|Australia.Western Australia
Coenotes eremophilae|LOQTC771-08|gvc8416-1L|658[0n]bp|Australia.Queensland

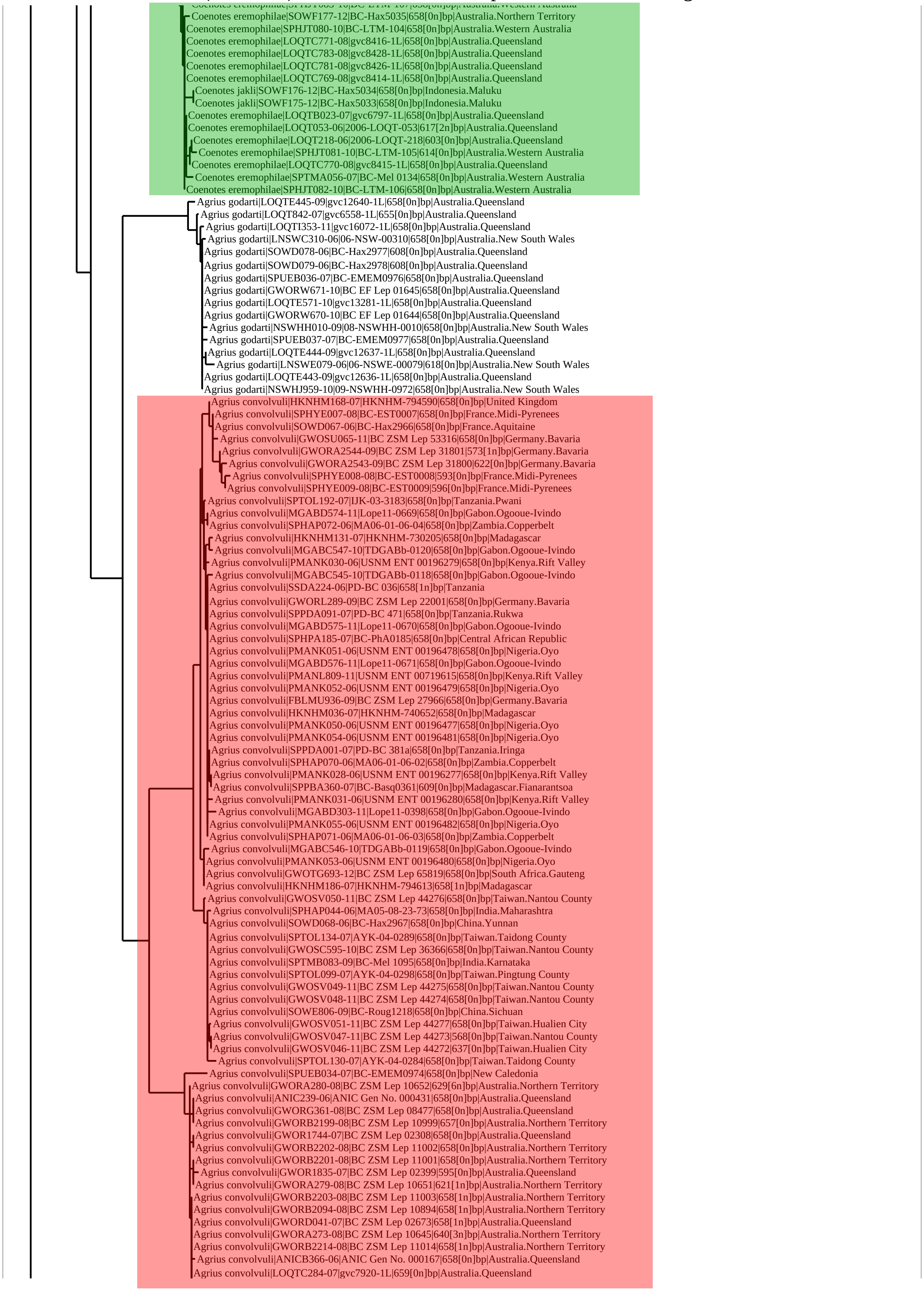
Coenotes eremophilae|SOWF177-12|BC-Hax5035|658[0n]bp|Australia.Northern Territory
Coenotes eremophilae|SPHJT080-10|BC-LTM-104|658[0n]bp|Australia.Western Australia
Coenotes eremophilae|LOQTC771-08|gvc8416-1L|658[0n]bp|Australia.Queensland
Coenotes eremophilae|LOQTC783-08|gvc8428-1L|658[0n]bp|Australia.Queensland
Coenotes eremophilae|LOQTC781-08|gvc8426-1L|658[0n]bp|Australia.Queensland
Coenotes eremophilae|LOQTC769-08|gvc8414-1L|658[0n]bp|Australia.Queensland
Coenotes jakli|SOWF176-12|BC-Hax5034|658[0n]bp|Indonesia.Maluku
Coenotes jakli|SOWF175-12|BC-Hax5033|658[0n]bp|Indonesia.Maluku
Coenotes eremophilae|LOQTB023-07|gvc6797-1L|658[0n]bp|Australia.Queensland
Coenotes eremophilae|LOQT053-06|2006-LOQT-053|617[2n]bp|Australia.Queensland
Coenotes eremophilae|LOQT218-06|2006-LOQT-218|603[0n]bp|Australia.Queensland
Coenotes eremophilae|SPHJT081-10|BC-LTM-105|614[0n]bp|Australia.Western Australia
Coenotes eremophilae|LOQTC770-08|gvc8415-1L|658[0n]bp|Australia.Queensland
Coenotes eremophilae|SPTMA056-07|BC-Mel 0134|658[0n]bp|Australia.Western Australia
Coenotes eremophilae|SPHJT082-10|BC-LTM-106|658[0n]bp|Australia.Western Australia
Agrius godarti|LOQTE445-09|gvc12640-1L|658[0n]bp|Australia.Queensland
Agrius godarti|LOQT842-07|gvc6558-1L|655[0n]bp|Australia.Queensland
Agrius godarti|LOQTI353-11|gvc16072-1L|658[0n]bp|Australia.Queensland
Agrius godarti|LNSWC310-06|06-NSW-00310|658[0n]bp|Australia.New South Wales
Agrius godarti|SOWD078-06|BC-Hax2977|608[0n]bp|Australia.Queensland
Agrius godarti|SOWD079-06|BC-Hax2978|608[0n]bp|Australia.Queensland
Agrius godarti|SPUEB036-07|BC-EMEM0976|658[0n]bp|Australia.Queensland
Agrius godarti|GWORW671-10|BC EF Lep 01645|658[0n]bp|Australia.Queensland
Agrius godarti|LOQTE571-10|gvc13281-1L|658[0n]bp|Australia.Queensland
Agrius godarti|GWORW670-10|BC EF Lep 01644|658[0n]bp|Australia.Queensland
Agrius godarti|NSWHH010-09|08-NSWHH-0010|658[0n]bp|Australia.New South Wales
Agrius godarti|SPUEB037-07|BC-EMEM0977|658[0n]bp|Australia.Queensland
Agrius godarti|LOQTE444-09|gvc12637-1L|658[0n]bp|Australia.Queensland
Agrius godarti|LNSWE079-06|06-NSWE-00079|618[0n]bp|Australia.New South Wales
Agrius godarti|LOQTE443-09|gvc12636-1L|658[0n]bp|Australia.Queensland
Agrius godarti|NSWHJ959-10|09-NSWHH-0972|658[0n]bp|Australia.New South Wales
Agrius convolvuli|HKNHM168-07|HKNHM-794590|658[0n]bp|United Kingdom
Agrius convolvuli|SPHYE007-08|BC-EST0007|658[0n]bp|France.Midi-Pyrenees
Agrius convolvuli|SOWD067-06|BC-Hax2966|658[0n]bp|France.Aquitaine
Agrius convolvuli|GWOSU065-11|BC ZSM Lep 53316|658[0n]bp|Germany.Bavaria
Agrius convolvuli|GWORA2544-09|BC ZSM Lep 31801|573[1n]bp|Germany.Bavaria
Agrius convolvuli|GWORA2543-09|BC ZSM Lep 31800|622[0n]bp|Germany.Bavaria
Agrius convolvuli|SPHYE008-08|BC-EST0008|593[0n]bp|France.Midi-Pyrenees
Agrius convolvuli|SPHYE009-08|BC-EST0009|596[0n]bp|France.Midi-Pyrenees
Agrius convolvuli|SPTOL192-07|IJK-03-3183|658[0n]bp|Tanzania.Pwani
Agrius convolvuli|MGABD574-11|Lope11-0669|658[0n]bp|Gabon.Ogooue-Ivindo
Agrius convolvuli|SPHAP072-06|MA06-01-06-04|658[0n]bp|Zambia.Copperbelt
Agrius convolvuli|HKNHM131-07|HKNHM-730205|658[0n]bp|Madagascar
Agrius convolvuli|MGABC547-10|TDGABb-0120|658[0n]bp|Gabon.Ogooue-Ivindo
Agrius convolvuli|PMANK030-06|USNM ENT 00196279|658[0n]bp|Kenya.Rift Valley
Agrius convolvuli|MGABC545-10|TDGABb-0118|658[0n]bp|Gabon.Ogooue-Ivindo
Agrius convolvuli|SSDA224-06|PD-BC 036|658[1n]bp|Tanzania
Agrius convolvuli|GWORL289-09|BC ZSM Lep 22001|658[0n]bp|Germany.Bavaria
Agrius convolvuli|SPPDA091-07|PD-BC 471|658[0n]bp|Tanzania.Rukwa
Agrius convolvuli|MGABD575-11|Lope11-0670|658[0n]bp|Gabon.Ogooue-Ivindo
Agrius convolvuli|SPHPA185-07|BC-PhA0185|658[0n]bp|Central African Republic
Agrius convolvuli|PMANK051-06|USNM ENT 00196478|658[0n]bp|Nigeria.Oyo
Agrius convolvuli|MGABD576-11|Lope11-0671|658[0n]bp|Gabon.Ogooue-Ivindo
Agrius convolvuli|PMANL809-11|USNM ENT 00719615|658[0n]bp|Kenya.Rift Valley
Agrius convolvuli|PMANK052-06|USNM ENT 00196479|658[0n]bp|Nigeria.Oyo
Agrius convolvuli|FBLMU936-09|BC ZSM Lep 27966|658[0n]bp|Germany.Bavaria
Agrius convolvuli|HKNHM036-07|HKNHM-740652|658[0n]bp|Madagascar
Agrius convolvuli|PMANK050-06|USNM ENT 00196477|658[0n]bp|Nigeria.Oyo
Agrius convolvuli|PMANK054-06|USNM ENT 00196481|658[0n]bp|Nigeria.Oyo
Agrius convolvuli|SPPDA001-07|PD-BC 381a|658[0n]bp|Tanzania.Iringa
Agrius convolvuli|SPHAP070-06|MA06-01-06-02|658[0n]bp|Zambia.Copperbelt
Agrius convolvuli|PMANK028-06|USNM ENT 00196277|658[0n]bp|Kenya.Rift Valley
Agrius convolvuli|SPPBA360-07|BC-Basq0361|609[0n]bp|Madagascar.Fianarantsoa
Agrius convolvuli|PMANK031-06|USNM ENT 00196280|658[0n]bp|Kenya.Rift Valley
Agrius convolvuli|MGABD303-11|Lope11-0398|658[0n]bp|Gabon.Ogooue-Ivindo
Agrius convolvuli|PMANK055-06|USNM ENT 00196482|658[0n]bp|Nigeria.Oyo
Agrius convolvuli|SPHAP071-06|MA06-01-06-03|658[0n]bp|Zambia.Copperbelt
Agrius convolvuli|MGABC546-10|TDGABb-0119|658[0n]bp|Gabon.Ogooue-Ivindo
Agrius convolvuli|PMANK053-06|USNM ENT 00196480|658[0n]bp|Nigeria.Oyo
Agrius convolvuli|GWOTG693-12|BC ZSM Lep 65819|658[0n]bp|South Africa.Gauteng
Agrius convolvuli|HKNHM186-07|HKNHM-794613|658[1n]bp|Madagascar
Agrius convolvuli|GWOSV050-11|BC ZSM Lep 44276|658[0n]bp|Taiwan.Nantou County
Agrius convolvuli|SPHAP044-06|MA05-08-23-73|658[0n]bp|India.Maharashtra
Agrius convolvuli|SOWD068-06|BC-Hax2967|658[0n]bp|China.Yunnan
Agrius convolvuli|SPTOL134-07|AYK-04-0289|658[0n]bp|Taiwan.Taidong County
Agrius convolvuli|GWOSC595-10|BC ZSM Lep 36366|658[0n]bp|Taiwan.Nantou County
Agrius convolvuli|SPTMB083-09|BC-Mel 1095|658[0n]bp|India.Karnataka
Agrius convolvuli|SPTOL099-07|AYK-04-0298|658[0n]bp|Taiwan.Pingtung County
Agrius convolvuli|GWOSV049-11|BC ZSM Lep 44275|658[0n]bp|Taiwan.Nantou County
Agrius convolvuli|GWOSV048-11|BC ZSM Lep 44274|658[0n]bp|Taiwan.Nantou County
Agrius convolvuli|SOWE806-09|BC-Roug1218|658[0n]bp|China.Sichuan
Agrius convolvuli|GWOSV051-11|BC ZSM Lep 44277|658[0n]bp|Taiwan.Hualien City
Agrius convolvuli|GWOSV047-11|BC ZSM Lep 44273|568[0n]bp|Taiwan.Nantou County
Agrius convolvuli|GWOSV046-11|BC ZSM Lep 44272|637[0n]bp|Taiwan.Hualien City
Agrius convolvuli|SPTOL130-07|AYK-04-0284|658[0n]bp|Taiwan.Taidong County
Agrius convolvuli|SPUEB034-07|BC-EMEM0974|658[0n]bp|New Caledonia
Agrius convolvuli|GWORA280-08|BC ZSM Lep 10652|629[6n]bp|Australia.Northern Territory
Agrius convolvuli|ANIC239-06|ANIC Gen No. 000431|658[0n]bp|Australia.Queensland
Agrius convolvuli|GWORG361-08|BC ZSM Lep 08477|658[0n]bp|Australia.Queensland
Agrius convolvuli|GWORB2199-08|BC ZSM Lep 10999|657[0n]bp|Australia.Northern Territory
Agrius convolvuli|GWOR1744-07|BC ZSM Lep 02308|658[0n]bp|Australia.Queensland
Agrius convolvuli|GWORB2202-08|BC ZSM Lep 11002|658[0n]bp|Australia.Northern Territory
Agrius convolvuli|GWORB2201-08|BC ZSM Lep 11001|658[0n]bp|Australia.Northern Territory
Agrius convolvuli|GWOR1835-07|BC ZSM Lep 02399|595[0n]bp|Australia.Queensland
Agrius convolvuli|GWORA279-08|BC ZSM Lep 10651|621[1n]bp|Australia.Northern Territory
Agrius convolvuli|GWORB2203-08|BC ZSM Lep 11003|658[1n]bp|Australia.Northern Territory
Agrius convolvuli|GWORB2094-08|BC ZSM Lep 10894|658[1n]bp|Australia.Northern Territory
Agrius convolvuli|GWORD041-07|BC ZSM Lep 02673|658[1n]bp|Australia.Queensland
Agrius convolvuli|GWORA273-08|BC ZSM Lep 10645|640[3n]bp|Australia.Northern Territory
Agrius convolvuli|GWORB2214-08|BC ZSM Lep 11014|658[1n]bp|Australia.Northern Territory
Agrius convolvuli|ANICB366-06|ANIC Gen No. 000167|658[0n]bp|Australia.Queensland
Agrius convolvuli|LOQTC284-07|gvc7920-1L|659[0n]bp|Australia.Queensland

Agrius convolvuli|GWORB2214-08|BC ZSM Lep 11014|658[1n]bp|Australia.Northern Territory
Agrius convolvuli|ANICB366-06|ANIC Gen No. 000167|658[0n]bp|Australia.Queensland
Agrius convolvuli|LOQTC284-07|gvc7920-1L|659[0n]bp|Australia.Queensland
Agrius convolvuli|GWORA267-08|BC ZSM Lep 10639|658[0n]bp|Australia.Northern Territory
Agrius convolvuli|LNSWE136-06|06-NSWE-00136|658[0n]bp|Australia.New South Wales
Agrius convolvuli|SPUEB033-07|BC-EMEM0973|658[0n]bp|Australia.Western Australia
Agrius convolvuli|LOQB569-05|Moth 048.03CL|658[0n]bp|Australia.Queensland
Agrius convolvuli|LSM256-11|K278668|658[0n]bp|Australia.New South Wales
Agrius convolvuli|GWOR1247-07|BC ZSM Lep 01999|658[0n]bp|Australia.Queensland
Agrius convolvuli|SPTOL196-07|MF-05-0017|658[0n]bp|Palau
Agrius convolvuli|GWORI528-09|BC ZSM Lep 13276|658[0n]bp|Australia.Northern Territory
Agrius convolvuli|NSWBB1144-08|07-NSWBB-1144|657[0n]bp|Australia.New South Wales
Agrius convolvuli|GWORA032-08|BC ZSM Lep 10404|658[0n]bp|Australia.Western Australia
Agrius convolvuli|GWORI500-09|BC ZSM Lep 13248|658[0n]bp|Australia.Northern Territory
Agrius convolvuli|IMLQ131-07|IM06-0439|658[0n]bp|Australia.Queensland
Agrius convolvuli|GWORI526-09|BC ZSM Lep 13274|658[0n]bp|Australia.Northern Territory
Agrius convolvuli|GWORA262-08|BC ZSM Lep 10634|658[0n]bp|Australia.Northern Territory
Agrius convolvuli|SPUEB032-07|BC-EMEM0972|658[0n]bp|Indonesia.Maluku
Agrius convolvuli|GWORD042-07|BC ZSM Lep 02674|658[0n]bp|Australia.Queensland
Agrius convolvuli|NSWHM019-11|BIOUG00851-G12|658[0n]bp|Australia.New South Wales
Agrius convolvuli|GWOR1251-07|BC ZSM Lep 02003|656[0n]bp|Australia.Queensland
Agrius convolvuli|LNSWE188-06|06-NSWE-00188|658[0n]bp|Australia.New South Wales
Agrius convolvuli|LOQB565-05|Moth 044.03CL|658[0n]bp|Australia.Queensland
Agrius convolvuli|GWORB2227-08|BC ZSM Lep 11027|657[0n]bp|Australia.Northern Territory
Agrius convolvuli|GWORB2226-08|BC ZSM Lep 11026|658[0n]bp|Australia.Northern Territory
Agrius convolvuli|GWORB2095-08|BC ZSM Lep 10895|658[0n]bp|Australia.Northern Territory
Agrius convolvuli|NSWHJ958-10|09-NSWHH-0971|658[0n]bp|Australia.New South Wales
Agrius convolvuli|GWORB2192-08|BC ZSM Lep 10992|658[0n]bp|Australia.Northern Territory
Agrius convolvuli|GWORA026-08|BC ZSM Lep 10398|657[0n]bp|Australia.Western Australia
Agrius convolvuli|NSWHM010-11|BIOUG00851-G03|658[0n]bp|Australia.New South Wales
Agrius convolvuli|GWORG300-08|BC ZSM Lep 08416|658[0n]bp|Australia.Queensland
Agrius convolvuli|PHLCC1137-11|BIOUG01235-A01|658[0n]bp|Australia.Australian Capital Territory
Agrius convolvuli|LOQB567-05|Moth 046.03CL|658[0n]bp|Australia.Queensland
Agrius convolvuli|LOQT747-06|gvc6449-1L|658[0n]bp|Australia.Queensland
Agrius convolvuli|IMLQ052-07|IM06-0212|658[0n]bp|Australia.Queensland
Agrius convolvuli|GWORB2213-08|BC ZSM Lep 11013|657[0n]bp|Australia.Northern Territory
Agrius convolvuli|LOQB074-05|Moth 074.03LZ|658[0n]bp|Australia.Queensland
Agrius convolvuli|NSWHH011-09|08-NSWHH-0011|658[0n]bp|Australia.New South Wales
Agrius convolvuli|LNSWE081-06|06-NSWE-00081|658[0n]bp|Australia.New South Wales
Agrius convolvuli|NSWHM029-11|BIOUG00851-H10|658[0n]bp|Australia.New South Wales
Agrius convolvuli|GWORC185-07|BC ZSM Lep 02535|658[0n]bp|Australia.Queensland
Agrius convolvuli|GWORB2195-08|BC ZSM Lep 10995|658[0n]bp|Australia.Northern Territory
Agrius convolvuli|GWORI485-09|BC ZSM Lep 13233|658[0n]bp|Australia.Northern Territory
Agrius convolvuli|GWORB2187-08|BC ZSM Lep 10987|658[0n]bp|Australia.Northern Territory
Agrius convolvuli|NSWHJ960-10|09-NSWHH-0973|658[0n]bp|Australia.New South Wales
Agrius convolvuli|LOQB566-05|Moth 045.03CL|658[0n]bp|Australia.Queensland
Agrius convolvuli|LOQTE740-10|gvc13992-1L|658[0n]bp|Australia.Queensland
Agrius convolvuli|GWORH539-09|BC ZSM Lep 10253|658[0n]bp|Australia.Queensland
Agrius convolvuli|GWORB2200-08|BC ZSM Lep 11000|657[0n]bp|Australia.Northern Territory
Agrius convolvuli|GWORA014-08|BC ZSM Lep 10386|658[0n]bp|Australia.Western Australia
Agrius convolvuli|LOQB075-05|Moth 075.03LZ|658[0n]bp|Australia.Queensland
Agrius convolvuli|NSWBB1227-08|07-NSWBB-1227|658[0n]bp|Australia.New South Wales
Agrius convolvuli|NSWHJ954-10|09-NSWHH-0967|658[0n]bp|Australia.New South Wales
Agrius convolvuli|GWORI527-09|BC ZSM Lep 13275|658[0n]bp|Australia.Northern Territory
Agrius convolvuli|GWORB2196-08|BC ZSM Lep 10996|658[0n]bp|Australia.Northern Territory
Agrius convolvuli|GWORC183-07|BC ZSM Lep 02533|658[0n]bp|Australia.Queensland
Agrius convolvuli|NSWHM456-11|BIOUG00912-G05|658[0n]bp|Australia.New South Wales
Agrius convolvuli|GWORB2191-08|BC ZSM Lep 10991|656[0n]bp|Australia.Northern Territory
Agrius convolvuli|GWORC144-07|BC ZSM Lep 02494|655[0n]bp|Australia.Queensland
Agrius convolvuli|GWORB2194-08|BC ZSM Lep 10994|656[0n]bp|Australia.Northern Territory
Agrius convolvuli|GWORA025-08|BC ZSM Lep 10397|655[0n]bp|Australia.Western Australia
Agrius convolvuli|LOQT806-07|gvc6516-1L|656[0n]bp|Australia.Queensland
Agrius convolvuli|GWORB2188-08|BC ZSM Lep 10988|655[0n]bp|Australia.Northern Territory
Agrius convolvuli|GWORA101-08|BC ZSM Lep 10473|646[0n]bp|Australia.Northern Territory
Agrius convolvuli|GWORC186-07|BC ZSM Lep 02536|647[0n]bp|Australia.Queensland
Agrius convolvuli|GWORA189-08|BC ZSM Lep 10561|645[0n]bp|Australia.Northern Territory
Agrius convolvuli|GWORB2198-08|BC ZSM Lep 10998|632[0n]bp|Australia.Northern Territory
Agrius convolvuli|GWORB2193-08|BC ZSM Lep 10993|632[0n]bp|Australia.Northern Territory
Agrius convolvuli|GWORB892-07|BC ZSM Lep 01362|632[0n]bp|Australia.Queensland
Agrius convolvuli|GWORC180-07|BC ZSM Lep 02530|631[0n]bp|Australia.Queensland
Agrius convolvuli|GWORC182-07|BC ZSM Lep 02532|632[0n]bp|Australia.Queensland
Agrius convolvuli|GWORC184-07|BC ZSM Lep 02534|632[0n]bp|Australia.Queensland
Agrius convolvuli|GWORC181-07|BC ZSM Lep 02531|632[0n]bp|Australia.Queensland
Agrius convolvuli|LOQB073-05|Moth 073.03LZ|649[0n]bp|Australia.Queensland
Agrius convolvuli|LOQ341-04|04HBL004341|593[0n]bp|Australia.Queensland
Agrius convolvuli|GWORA142-08|BC ZSM Lep 10514|563[0n]bp|Australia.Northern Territory
Agrius convolvuli|ANICB1117-07|ANIC Gen No. 003135|647[0n]bp|Australia.South Australia
Agrius convolvuli|LOQB077-05|Moth 077.03LZ|616[0n]bp|Australia.Queensland
Agrius convolvuli|GWORB2092-08|BC ZSM Lep 10892|618[0n]bp|Australia.Northern Territory
Agrius convolvuli|GWORB854-07|BC ZSM Lep 01324|617[0n]bp|Australia.Queensland
Agrius convolvuli|GWORA266-08|BC ZSM Lep 10638|617[0n]bp|Australia.Northern Territory
Agrius convolvuli|GWORA274-08|BC ZSM Lep 10646|639[0n]bp|Australia.Northern Territory
Agrius convolvuli|GWORA144-08|BC ZSM Lep 10516|618[0n]bp|Australia.Northern Territory
Agrius convolvuli|GWORA281-08|BC ZSM Lep 10653|638[1n]bp|Australia.Northern Territory
Agrius convolvuli|SOWD070-06|BC-Hax2969|552[0n]bp|French Polynesia.Society Islands
Agrius convolvuli|SOWD069-06|BC-Hax2968|608[0n]bp|French Polynesia.Society Islands
Agrius convolvuli|GWORB2069-08|BC ZSM Lep 10869|609[0n]bp|Australia.Northern Territory
Agrius convolvuli|GWORB2093-08|BC ZSM Lep 10893|624[0n]bp|Australia.Northern Territory
Agrius convolvuli|LOQB072-05|Moth 072.03LZ|620[0n]bp|Australia.Queensland
Agrius convolvuli|GWORA159-08|BC ZSM Lep 10531|587[0n]bp|Australia.Western Australia
Agrius convolvuli|GWORA160-08|BC ZSM Lep 10532|602[0n]bp|Australia.Western Australia
Agrius convolvuli|LSM875-11|K287774|628[0n]bp|Australia.New South Wales
Agrius convolvuli|LNSWE009-06|06-NSWE-00009|600[0n]bp|Australia.New South Wales
Agrius convolvuli|GWORA140-08|BC ZSM Lep 10512|610[1n]bp|Australia.Western Australia
Agrius convolvuli|GWORB988-07|BC ZSM Lep 02210|589[1n]bp|Papua New Guinea.Central
Agrius convolvuli|GWOR1836-07|BC ZSM Lep 02400|586[0n]bp|Australia.Queensland
Agrius convolvuli|HCPN009-03|USNM ENT 00678977|601[0n]bp|Papua New Guinea.Madang
Agrius convolvuli|LOQB078-05|Moth 078.03LZ|572[0n]bp|Australia.Queensland
Agrius convolvuli|LOQB076-05|Moth 076.03LZ|573[0n]bp|Australia.Queensland
Agrius convolvuli|LOQB071-05|Moth 071.03LZ|572[0n]bp|Australia.Queensland
Agrius convolvuli|LOQB568-05|Moth 047.03CL|528[0n]bp|Australia.Queensland
Agrius convolvuli|GWORA143-08|BC ZSM Lep 10515|552[1n]bp|Australia.Northern Territory
Agrius convolvuli|LOQB313-05|Moth 313.01LZ|570[0n]bp|Australia.Queensland

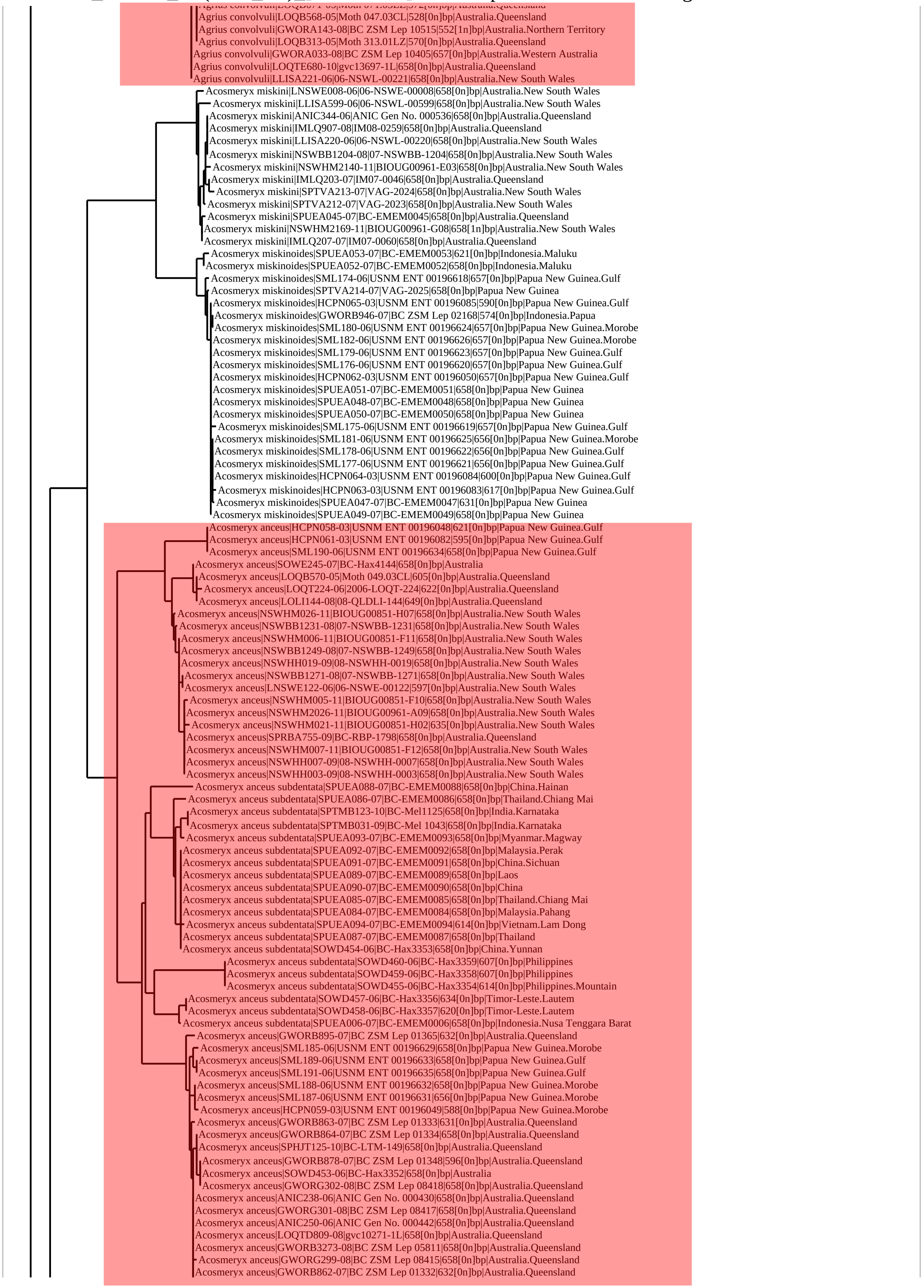
Agrius convolvuli|LOQB568-05|Moth 047.03CL|528[0n]bp|Australia.Queensland
Agrius convolvuli|GWORA143-08|BC ZSM Lep 10515|552[1n]bp|Australia.Northern Territory
Agrius convolvuli|LOQB313-05|Moth 313.01LZ|570[0n]bp|Australia.Queensland
Agrius convolvuli|GWORA033-08|BC ZSM Lep 10405|657[0n]bp|Australia.Western Australia
Agrius convolvuli|LOQTE680-10|gvc13697-1L|658[0n]bp|Australia.Queensland
Agrius convolvuli|LLISA221-06|06-NSWL-00221|658[0n]bp|Australia.New South Wales
Acosmeryx miskini|LNSWE008-06|06-NSWE-00008|658[0n]bp|Australia.New South Wales
Acosmeryx miskini|LLISA599-06|06-NSWL-00599|658[0n]bp|Australia.New South Wales
Acosmeryx miskini|ANIC344-06|ANIC Gen No. 000536|658[0n]bp|Australia.Queensland
Acosmeryx miskini|IMLQ907-08|IM08-0259|658[0n]bp|Australia.Queensland
Acosmeryx miskini|LLISA220-06|06-NSWL-00220|658[0n]bp|Australia.New South Wales
Acosmeryx miskini|NSWBB1204-08|07-NSWBB-1204|658[0n]bp|Australia.New South Wales
Acosmeryx miskini|NSWHM2140-11|BIOUG00961-E03|658[0n]bp|Australia.New South Wales
Acosmeryx miskini|IMLQ203-07|IM07-0046|658[0n]bp|Australia.Queensland
Acosmeryx miskini|SPTVA213-07|VAG-2024|658[0n]bp|Australia.New South Wales
Acosmeryx miskini|SPTVA212-07|VAG-2023|658[0n]bp|Australia.New South Wales
Acosmeryx miskini|SPUEA045-07|BC-EMEM0045|658[0n]bp|Australia.Queensland
Acosmeryx miskini|NSWHM2169-11|BIOUG00961-G08|658[1n]bp|Australia.New South Wales
Acosmeryx miskini|IMLQ207-07|IM07-0060|658[0n]bp|Australia.Queensland
Acosmeryx miskinoides|SPUEA053-07|BC-EMEM0053|621[0n]bp|Indonesia.Maluku
Acosmeryx miskinoides|SPUEA052-07|BC-EMEM0052|658[0n]bp|Indonesia.Maluku
Acosmeryx miskinoides|SML174-06|USNM ENT 00196618|657[0n]bp|Papua New Guinea.Gulf
Acosmeryx miskinoides|SPTVA214-07|VAG-2025|658[0n]bp|Papua New Guinea
Acosmeryx miskinoides|HCPN065-03|USNM ENT 00196085|590[0n]bp|Papua New Guinea.Gulf
Acosmeryx miskinoides|GWORB946-07|BC ZSM Lep 02168|574[0n]bp|Indonesia.Papua
Acosmeryx miskinoides|SML180-06|USNM ENT 00196624|657[0n]bp|Papua New Guinea.Morobe
Acosmeryx miskinoides|SML182-06|USNM ENT 00196626|657[0n]bp|Papua New Guinea.Morobe
Acosmeryx miskinoides|SML179-06|USNM ENT 00196623|657[0n]bp|Papua New Guinea.Gulf
Acosmeryx miskinoides|SML176-06|USNM ENT 00196620|657[0n]bp|Papua New Guinea.Gulf
Acosmeryx miskinoides|HCPN062-03|USNM ENT 00196050|657[0n]bp|Papua New Guinea.Gulf
Acosmeryx miskinoides|SPUEA051-07|BC-EMEM0051|658[0n]bp|Papua New Guinea
Acosmeryx miskinoides|SPUEA048-07|BC-EMEM0048|658[0n]bp|Papua New Guinea
Acosmeryx miskinoides|SPUEA050-07|BC-EMEM0050|658[0n]bp|Papua New Guinea
Acosmeryx miskinoides|SML175-06|USNM ENT 00196619|657[0n]bp|Papua New Guinea.Gulf
Acosmeryx miskinoides|SML181-06|USNM ENT 00196625|656[0n]bp|Papua New Guinea.Morobe
Acosmeryx miskinoides|SML178-06|USNM ENT 00196622|656[0n]bp|Papua New Guinea.Gulf
Acosmeryx miskinoides|SML177-06|USNM ENT 00196621|656[0n]bp|Papua New Guinea.Gulf
Acosmeryx miskinoides|HCPN064-03|USNM ENT 00196084|600[0n]bp|Papua New Guinea.Gulf
Acosmeryx miskinoides|HCPN063-03|USNM ENT 00196083|617[0n]bp|Papua New Guinea.Gulf
Acosmeryx miskinoides|SPUEA047-07|BC-EMEM0047|631[0n]bp|Papua New Guinea
Acosmeryx miskinoides|SPUEA049-07|BC-EMEM0049|658[0n]bp|Papua New Guinea
Acosmeryx anceus|HCPN058-03|USNM ENT 00196048|621[0n]bp|Papua New Guinea.Gulf
Acosmeryx anceus|HCPN061-03|USNM ENT 00196082|595[0n]bp|Papua New Guinea.Gulf
Acosmeryx anceus|SML190-06|USNM ENT 00196634|658[0n]bp|Papua New Guinea.Gulf
Acosmeryx anceus|SOWE245-07|BC-Hax4144|658[0n]bp|Australia
Acosmeryx anceus|LOQB570-05|Moth 049.03CL|605[0n]bp|Australia.Queensland
Acosmeryx anceus|LOQT224-06|2006-LOQT-224|622[0n]bp|Australia.Queensland
Acosmeryx anceus|LOLI144-08|08-QLDLI-144|649[0n]bp|Australia.Queensland
Acosmeryx anceus|NSWHM026-11|BIOUG00851-H07|658[0n]bp|Australia.New South Wales
Acosmeryx anceus|NSWBB1231-08|07-NSWBB-1231|658[0n]bp|Australia.New South Wales
Acosmeryx anceus|NSWHM006-11|BIOUG00851-F11|658[0n]bp|Australia.New South Wales
Acosmeryx anceus|NSWBB1249-08|07-NSWBB-1249|658[0n]bp|Australia.New South Wales
Acosmeryx anceus|NSWHH019-09|08-NSWHH-0019|658[0n]bp|Australia.New South Wales
Acosmeryx anceus|NSWBB1271-08|07-NSWBB-1271|658[0n]bp|Australia.New South Wales
Acosmeryx anceus|LNSWE122-06|06-NSWE-00122|597[0n]bp|Australia.New South Wales
Acosmeryx anceus|NSWHM005-11|BIOUG00851-F10|658[0n]bp|Australia.New South Wales
Acosmeryx anceus|NSWHM2026-11|BIOUG00961-A09|658[0n]bp|Australia.New South Wales
Acosmeryx anceus|NSWHM021-11|BIOUG00851-H02|635[0n]bp|Australia.New South Wales
Acosmeryx anceus|SPRBA755-09|BC-RBP-1798|658[0n]bp|Australia.Queensland
Acosmeryx anceus|NSWHM007-11|BIOUG00851-F12|658[0n]bp|Australia.New South Wales
Acosmeryx anceus|NSWHH007-09|08-NSWHH-0007|658[0n]bp|Australia.New South Wales
Acosmeryx anceus|NSWHH003-09|08-NSWHH-0003|658[0n]bp|Australia.New South Wales
Acosmeryx anceus subdentata|SPUEA088-07|BC-EMEM0088|658[0n]bp|China.Hainan
Acosmeryx anceus subdentata|SPUEA086-07|BC-EMEM0086|658[0n]bp|Thailand.Chiang Mai
Acosmeryx anceus subdentata|SPTMB123-10|BC-Mel1125|658[0n]bp|India.Karnataka
Acosmeryx anceus subdentata|SPTMB031-09|BC-Mel 1043|658[0n]bp|India.Karnataka
Acosmeryx anceus subdentata|SPUEA093-07|BC-EMEM0093|658[0n]bp|Myanmar.Magway
Acosmeryx anceus subdentata|SPUEA092-07|BC-EMEM0092|658[0n]bp|Malaysia.Perak
Acosmeryx anceus subdentata|SPUEA091-07|BC-EMEM0091|658[0n]bp|China.Sichuan
Acosmeryx anceus subdentata|SPUEA089-07|BC-EMEM0089|658[0n]bp|Laos
Acosmeryx anceus subdentata|SPUEA090-07|BC-EMEM0090|658[0n]bp|China
Acosmeryx anceus subdentata|SPUEA085-07|BC-EMEM0085|658[0n]bp|Thailand.Chiang Mai
Acosmeryx anceus subdentata|SPUEA084-07|BC-EMEM0084|658[0n]bp|Malaysia.Pahang
Acosmeryx anceus subdentata|SPUEA094-07|BC-EMEM0094|614[0n]bp|Vietnam.Lam Dong
Acosmeryx anceus subdentata|SPUEA087-07|BC-EMEM0087|658[0n]bp|Thailand
Acosmeryx anceus subdentata|SOWD454-06|BC-Hax3353|658[0n]bp|China.Yunnan
Acosmeryx anceus subdentata|SOWD460-06|BC-Hax3359|607[0n]bp|Philippines
Acosmeryx anceus subdentata|SOWD459-06|BC-Hax3358|607[0n]bp|Philippines
Acosmeryx anceus subdentata|SOWD455-06|BC-Hax3354|614[0n]bp|Philippines.Mountain
Acosmeryx anceus subdentata|SOWD457-06|BC-Hax3356|634[0n]bp|Timor-Leste.Lautem
Acosmeryx anceus subdentata|SOWD458-06|BC-Hax3357|620[0n]bp|Timor-Leste.Lautem
Acosmeryx anceus subdentata|SPUEA006-07|BC-EMEM0006|658[0n]bp|Indonesia.Nusa Tenggara Barat
Acosmeryx anceus|GWORB895-07|BC ZSM Lep 01365|632[0n]bp|Australia.Queensland
Acosmeryx anceus|SML185-06|USNM ENT 00196629|658[0n]bp|Papua New Guinea.Morobe
Acosmeryx anceus|SML189-06|USNM ENT 00196633|658[0n]bp|Papua New Guinea.Gulf
Acosmeryx anceus|SML191-06|USNM ENT 00196635|658[0n]bp|Papua New Guinea.Gulf
Acosmeryx anceus|SML188-06|USNM ENT 00196632|658[0n]bp|Papua New Guinea.Morobe
Acosmeryx anceus|SML187-06|USNM ENT 00196631|656[0n]bp|Papua New Guinea.Morobe
Acosmeryx anceus|HCPN059-03|USNM ENT 00196049|588[0n]bp|Papua New Guinea.Morobe
Acosmeryx anceus|GWORB863-07|BC ZSM Lep 01333|631[0n]bp|Australia.Queensland
Acosmeryx anceus|GWORB864-07|BC ZSM Lep 01334|658[0n]bp|Australia.Queensland
Acosmeryx anceus|SPHJT125-10|BC-LTM-149|658[0n]bp|Australia.Queensland
Acosmeryx anceus|GWORB878-07|BC ZSM Lep 01348|596[0n]bp|Australia.Queensland
Acosmeryx anceus|SOWD453-06|BC-Hax3352|658[0n]bp|Australia
Acosmeryx anceus|GWORG302-08|BC ZSM Lep 08418|658[0n]bp|Australia.Queensland
Acosmeryx anceus|ANIC238-06|ANIC Gen No. 000430|658[0n]bp|Australia.Queensland
Acosmeryx anceus|GWORG301-08|BC ZSM Lep 08417|658[0n]bp|Australia.Queensland
Acosmeryx anceus|ANIC250-06|ANIC Gen No. 000442|658[0n]bp|Australia.Queensland
Acosmeryx anceus|LOQTD809-08|gvc10271-1L|658[0n]bp|Australia.Queensland
Acosmeryx anceus|GWORB3273-08|BC ZSM Lep 05811|658[0n]bp|Australia.Queensland
Acosmeryx anceus|GWORG299-08|BC ZSM Lep 08415|658[0n]bp|Australia.Queensland
Acosmeryx anceus|GWORB862-07|BC ZSM Lep 01332|632[0n]bp|Australia.Queensland

Acosmeryx anceus|GWORB3273-08|BC ZSM Lep 05811|658[0n]bp|Australia.Queensland
Acosmeryx anceus|GWORG299-08|BC ZSM Lep 08415|658[0n]bp|Australia.Queensland
Acosmeryx anceus|GWORB862-07|BC ZSM Lep 01332|632[0n]bp|Australia.Queensland
Acosmeryx anceus|GWORB877-07|BC ZSM Lep 01347|631[0n]bp|Australia.Queensland
Acosmeryx anceus|GWORB896-07|BC ZSM Lep 01366|632[0n]bp|Australia.Queensland
Acosmeryx anceus|GWORC240-07|BC ZSM Lep 02590|646[0n]bp|Australia.Queensland
Acosmeryx anceus|GWORC282-07|BC ZSM Lep 02632|647[0n]bp|Australia.Queensland
Acosmeryx anceus|GWORB897-07|BC ZSM Lep 01367|630[0n]bp|Australia.Queensland
Acosmeryx anceus|GWORB894-07|BC ZSM Lep 01364|617[0n]bp|Australia.Queensland
Acosmeryx anceus|LOQB332-05|Moth 016.03CC|581[0n]bp|Australia.Queensland
Acosmeryx anceus|GWORC239-07|BC ZSM Lep 02589|658[0n]bp|Australia.Queensland
Acosmeryx anceus|LOQB521-05|Moth 205.03CC|557[0n]bp|Australia.Queensland
Acosmeryx anceus|GWORC238-07|BC ZSM Lep 02588|616[0n]bp|Australia.Queensland
Acosmeryx anceus|LOQB329-05|Moth 013.03CC|593[0n]bp|Australia.Queensland
Acosmeryx anceus|GWORB978-07|BC ZSM Lep 02200|594[2n]bp|Indonesia.Papua
Acosmeryx anceus|GWORB948-07|BC ZSM Lep 02170|524[2n]bp|Indonesia.Papua
Acosmeryx anceus|GWORB979-07|BC ZSM Lep 02201|594[2n]bp|Indonesia.Papua
Acosmeryx anceus|GWORB879-07|BC ZSM Lep 01349|557[0n]bp|Australia.Queensland
Acosmeryx anceus|GWOR1880-07|BC ZSM Lep 02444|593[2n]bp|Australia.Queensland
Acosmeryx anceus|GWOR1826-07|BC ZSM Lep 02390|596[0n]bp|Australia.Queensland
Acosmeryx anceus|GWORC281-07|BC ZSM Lep 02631|653[0n]bp|Australia.Queensland
Hippotion celerio|LNSWE077-06|06-NSWE-00077|656[0n]bp|Australia.New South Wales
Hippotion celerio|GWORA089-08|BC ZSM Lep 10461|655[0n]bp|Australia.Western Australia
Hippotion celerio|LNSWE004-06|06-NSWE-00004|600[0n]bp|Australia.New South Wales
Hippotion celerio|LNSWE055-06|06-NSWE-00055|600[0n]bp|Australia.New South Wales
Hippotion celerio|LNSWE072-06|06-NSWE-00072|600[0n]bp|Australia.New South Wales
Hippotion celerio|HCPN012-03|USNM ENT 00678971|600[0n]bp|Papua New Guinea.Madang
Hippotion celerio|LNSWE138-06|06-NSWE-00138|591[0n]bp|Australia.New South Wales
Hippotion celerio|SPTMB552-11|BC-Mel1554|658[0n]bp|Indonesia.Sumatera Barat
Hippotion celerio|LNSWB151-05|05-NSW-01091|658[0n]bp|Australia.New South Wales
Hippotion celerio|NSWHJ052-10|09-NSWHH-0065|658[0n]bp|Australia.New South Wales
Hippotion celerio|LNSWE052-06|06-NSWE-00052|658[0n]bp|Australia.New South Wales
Hippotion celerio|LNSWC575-08|AM 2296|658[0n]bp|Australia.New South Wales
Hippotion celerio|LNSWB152-05|05-NSW-01092|658[0n]bp|Australia.New South Wales
Hippotion celerio|PHLCC1141-11|BIOUG01235-A05|658[0n]bp|Australia.Australian Capital Territory
Hippotion celerio|LNSWE058-06|06-NSWE-00058|658[0n]bp|Australia.New South Wales
Hippotion celerio|LNSWC312-06|06-NSW-00312|658[0n]bp|Australia.New South Wales
Hippotion celerio|LOQTI045-10|gvc14670-1L|658[0n]bp|Australia.Queensland
Hippotion celerio|LNSWE050-06|06-NSWE-00050|658[0n]bp|Australia.New South Wales
Hippotion celerio|LNSWB153-05|05-NSW-01093|658[0n]bp|Australia.New South Wales
Hippotion celerio|SPTMB559-11|BC-Mel1561|658[0n]bp|Indonesia.Papua Barat
Hippotion celerio|LNSWE090-06|06-NSWE-00090|658[0n]bp|Australia.New South Wales
Hippotion celerio|SOWF097-12|BC-Hax4955|658[0n]bp|New Caledonia.South
Hippotion celerio|LNSWE084-06|06-NSWE-00084|658[0n]bp|Australia.New South Wales
Hippotion celerio|LNSWE041-06|06-NSWE-00041|658[0n]bp|Australia.New South Wales
Hippotion celerio|LNSWE093-06|06-NSWE-00093|658[0n]bp|Australia.New South Wales
Hippotion celerio|SPTMB558-11|BC-Mel1560|658[0n]bp|Indonesia.Papua Barat
Hippotion celerio|LNSWE128-06|06-NSWE-00128|658[0n]bp|Australia.New South Wales
Hippotion celerio|GWORB989-07|BC ZSM Lep 02211|606[1n]bp|Papua New Guinea.Central
Hippotion celerio|LNSWE123-06|06-NSWE-00123|613[0n]bp|Australia.New South Wales
Hippotion celerio|LNSWE048-06|06-NSWE-00048|597[0n]bp|Australia.New South Wales
Hippotion celerio|LNSWE106-06|06-NSWE-00106|595[0n]bp|Australia.New South Wales
Hippotion celerio|SPTMB554-11|BC-Mel1556|658[0n]bp|Indonesia
Hippotion celerio|LNSWC573-08|AM 2294|658[0n]bp|Australia.New South Wales
Hippotion celerio|LOQTI613-12|gvc17129-1L|658[0n]bp|Australia.Queensland
Hippotion celerio|LNSWE131-06|06-NSWE-00131|561[0n]bp|Australia.New South Wales
Hippotion celerio|LNSWE137-06|06-NSWE-00137|533[0n]bp|Australia.New South Wales
Hippotion celerio|LNSWE087-06|06-NSWE-00087|578[0n]bp|Australia.New South Wales
Hippotion celerio|LNSWE108-06|06-NSWE-00108|560[0n]bp|Australia.New South Wales
Hippotion celerio|GWORN207-09|BC ZSM Lep 18235|658[0n]bp|Australia.Western Australia
Hippotion celerio|LNSWE126-06|06-NSWE-00126|584[0n]bp|Australia.New South Wales
Hippotion celerio|LSM1761-11|am10761|658[0n]bp|Australia.New South Wales
Hippotion celerio|LNSWE102-06|06-NSWE-00102|658[0n]bp|Australia.New South Wales
Hippotion celerio|LNSWC574-08|AM 2295|658[0n]bp|Australia.New South Wales
Hippotion celerio|LNSWE127-06|06-NSWE-00127|615[2n]bp|Australia.New South Wales
Hippotion celerio|LNSWE054-06|06-NSWE-00054|615[0n]bp|Australia.New South Wales
Hippotion celerio|LNSWE068-06|06-NSWE-00068|615[0n]bp|Australia.New South Wales
Hippotion celerio|LNSWC576-08|AM 2297|658[0n]bp|Australia.New South Wales
Hippotion celerio|SPTMB553-11|BC-Mel1555|658[0n]bp|Indonesia.Sumatera Selatan
Hippotion celerio|LNSWE053-06|06-NSWE-00053|658[0n]bp|Australia.New South Wales
Hippotion celerio|PHLCC1153-11|BIOUG01235-B05|658[0n]bp|Australia.Australian Capital Territory
Hippotion celerio|SPTMB557-11|BC-Mel1559|658[0n]bp|Indonesia.Bali
Hippotion celerio|SPTMB555-11|BC-Mel1557|658[0n]bp|Indonesia.Nusa Tenggara Timur
Hippotion celerio|SPTMB551-11|BC-Mel1553|658[0n]bp|Indonesia.Nusa Tenggara Timur
Hippotion celerio|SPTMB556-11|BC-Mel1558|658[0n]bp|Indonesia.Bali
Hippotion celerio|SPTMB550-11|BC-Mel1552|658[0n]bp|Indonesia.Nusa Tenggara Timur
Hippotion celerio|PMANK047-06|USNM ENT 00196449|658[0n]bp|Kenya.Rift Valley
Hippotion celerio|LOSA203-08|05-SA-211|658[0n]bp|South Africa
Hippotion celerio|PMANK014-06|USNM ENT 00196006|658[0n]bp|Kenya.Rift Valley
Hippotion celerio|PMANK015-06|USNM ENT 00196010|658[0n]bp|Kenya.Rift Valley
Hippotion celerio|SPHYE219-09|BC-EST0595|658[0n]bp|Tanzania
Hippotion celerio|SSDA208-06|PD-BC 020|658[0n]bp|Tanzania
Hippotion celerio|PMANK017-06|USNM ENT 00196012|658[0n]bp|Kenya.Rift Valley
Hippotion celerio|SPPDA093-07|PD-BC 473|658[0n]bp|Tanzania.Rukwa
Hippotion celerio|PMANK021-06|USNM ENT 00196270|658[0n]bp|Kenya.Rift Valley
Hippotion celerio|SPTMB904-11|BC-Mel1906|658[0n]bp|Central African Republic
Hippotion celerio|PMANK016-06|USNM ENT 00196011|658[0n]bp|Kenya.Rift Valley
Hippotion celerio|LOSA200-08|05-SA-208|658[0n]bp|South Africa
Hippotion celerio|SPTMB905-11|BC-Mel1907|658[0n]bp|Central African Republic
Hippotion celerio|HKNHM150-07|HKNHM-794572|658[0n]bp|Madagascar
Hippotion celerio|SPMNP290-07|BC-MNHNJP0180|658[0n]bp|Malawi
Hippotion celerio|PMANK023-06|USNM ENT 00196272|658[0n]bp|Kenya.Rift Valley
Hippotion celerio|PMANK101-06|USNM ENT 00196505|658[0n]bp|Nigeria.Oyo
Hippotion celerio|PMANK019-06|USNM ENT 00196027|658[0n]bp|Kenya.Rift Valley
Hippotion celerio|SPHYE116-09|BC-EST0492|618[0n]bp|Tanzania
Hippotion celerio|PMANK102-06|USNM ENT 00196506|658[0n]bp|Nigeria.Oyo
Hippotion celerio|SPHAP036-06|MA05-06-11-10|658[0n]bp|Zambia.Copperbelt
Hippotion celerio|HKNHM043-07|HKNHM-740660|658[0n]bp|Madagascar
Hippotion celerio|PMANK103-06|USNM ENT 00196507|658[0n]bp|Nigeria.Oyo
Hippotion celerio|PMANK104-06|USNM ENT 00196508|658[0n]bp|Nigeria.Oyo
Hippotion celerio|PMANK020-06|USNM ENT 00196028|658[0n]bp|Kenya.Rift Valley
Hippotion celerio|HKNHM153-07|HKNHM-794575|658[0n]bp|Madagascar
[illegible]

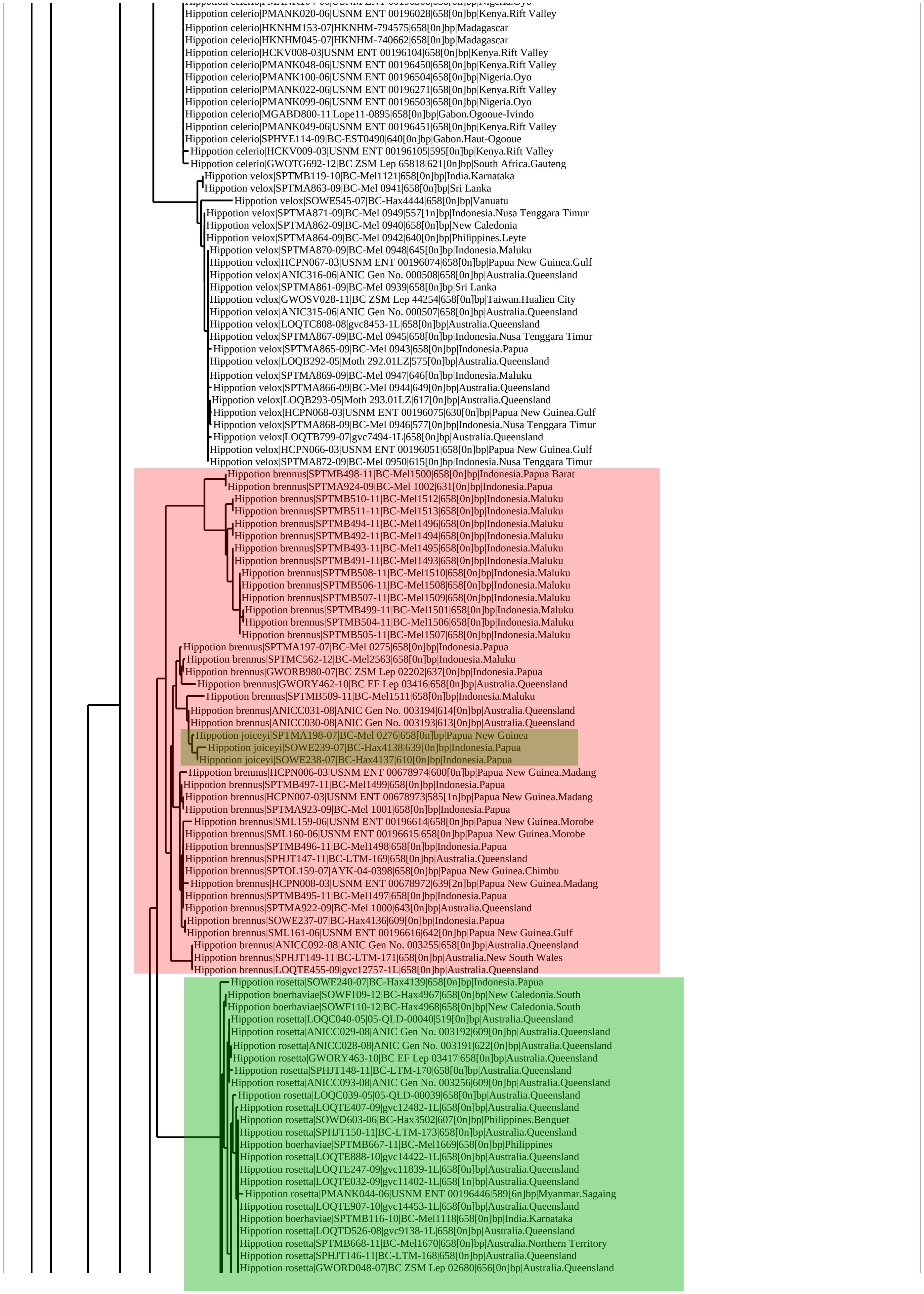
Hippotion celerio|PMANK020-06|USNM ENT 00196028|658[0n]bp|Kenya.Rift Valley
Hippotion celerio|HKNHM153-07|HKNHM-794575|658[0n]bp|Madagascar
Hippotion celerio|HKNHM045-07|HKNHM-740662|658[0n]bp|Madagascar
Hippotion celerio|HCKV008-03|USNM ENT 00196104|658[0n]bp|Kenya.Rift Valley
Hippotion celerio|PMANK048-06|USNM ENT 00196450|658[0n]bp|Kenya.Rift Valley
Hippotion celerio|PMANK100-06|USNM ENT 00196504|658[0n]bp|Nigeria.Oyo
Hippotion celerio|PMANK022-06|USNM ENT 00196271|658[0n]bp|Kenya.Rift Valley
Hippotion celerio|PMANK099-06|USNM ENT 00196503|658[0n]bp|Nigeria.Oyo
Hippotion celerio|MGABD800-11|Lope11-0895|658[0n]bp|Gabon.Ogooue-Ivindo
Hippotion celerio|PMANK049-06|USNM ENT 00196451|658[0n]bp|Kenya.Rift Valley
Hippotion celerio|SPHYE114-09|BC-EST0490|640[0n]bp|Gabon.Haut-Ogooue
Hippotion celerio|HCKV009-03|USNM ENT 00196105|595[0n]bp|Kenya.Rift Valley
Hippotion celerio|GWOTG692-12|BC ZSM Lep 65818|621[0n]bp|South Africa.Gauteng
Hippotion velox|SPTMB119-10|BC-Mel1121|658[0n]bp|India.Karnataka
Hippotion velox|SPTMA863-09|BC-Mel 0941|658[0n]bp|Sri Lanka
Hippotion velox|SOWE545-07|BC-Hax4444|658[0n]bp|Vanuatu
Hippotion velox|SPTMA871-09|BC-Mel 0949|557[1n]bp|Indonesia.Nusa Tenggara Timur
Hippotion velox|SPTMA862-09|BC-Mel 0940|658[0n]bp|New Caledonia
Hippotion velox|SPTMA864-09|BC-Mel 0942|640[0n]bp|Philippines.Leyte
Hippotion velox|SPTMA870-09|BC-Mel 0948|645[0n]bp|Indonesia.Maluku
Hippotion velox|HCPN067-03|USNM ENT 00196074|658[0n]bp|Papua New Guinea.Gulf
Hippotion velox|ANIC316-06|ANIC Gen No. 000508|658[0n]bp|Australia.Queensland
Hippotion velox|SPTMA861-09|BC-Mel 0939|658[0n]bp|Sri Lanka
Hippotion velox|GWOSV028-11|BC ZSM Lep 44254|658[0n]bp|Taiwan.Hualien City
Hippotion velox|ANIC315-06|ANIC Gen No. 000507|658[0n]bp|Australia.Queensland
Hippotion velox|LOQTC808-08|gvc8453-1L|658[0n]bp|Australia.Queensland
Hippotion velox|SPTMA867-09|BC-Mel 0945|658[0n]bp|Indonesia.Nusa Tenggara Timur
Hippotion velox|SPTMA865-09|BC-Mel 0943|658[0n]bp|Indonesia.Papua
Hippotion velox|LOQB292-05|Moth 292.01LZ|575[0n]bp|Australia.Queensland
Hippotion velox|SPTMA869-09|BC-Mel 0947|646[0n]bp|Indonesia.Maluku
Hippotion velox|SPTMA866-09|BC-Mel 0944|649[0n]bp|Australia.Queensland
Hippotion velox|LOQB293-05|Moth 293.01LZ|617[0n]bp|Australia.Queensland
Hippotion velox|HCPN068-03|USNM ENT 00196075|630[0n]bp|Papua New Guinea.Gulf
Hippotion velox|SPTMA868-09|BC-Mel 0946|577[0n]bp|Indonesia.Nusa Tenggara Timur
Hippotion velox|LOQTB799-07|gvc7494-1L|658[0n]bp|Australia.Queensland
Hippotion velox|HCPN066-03|USNM ENT 00196051|658[0n]bp|Papua New Guinea.Gulf
Hippotion velox|SPTMA872-09|BC-Mel 0950|615[0n]bp|Indonesia.Nusa Tenggara Timur
Hippotion brennus|SPTMB498-11|BC-Mel1500|658[0n]bp|Indonesia.Papua Barat
Hippotion brennus|SPTMA924-09|BC-Mel 1002|631[0n]bp|Indonesia.Papua
Hippotion brennus|SPTMB510-11|BC-Mel1512|658[0n]bp|Indonesia.Maluku
Hippotion brennus|SPTMB511-11|BC-Mel1513|658[0n]bp|Indonesia.Maluku
Hippotion brennus|SPTMB494-11|BC-Mel1496|658[0n]bp|Indonesia.Maluku
Hippotion brennus|SPTMB492-11|BC-Mel1494|658[0n]bp|Indonesia.Maluku
Hippotion brennus|SPTMB493-11|BC-Mel1495|658[0n]bp|Indonesia.Maluku
Hippotion brennus|SPTMB491-11|BC-Mel1493|658[0n]bp|Indonesia.Maluku
Hippotion brennus|SPTMB508-11|BC-Mel1510|658[0n]bp|Indonesia.Maluku
Hippotion brennus|SPTMB506-11|BC-Mel1508|658[0n]bp|Indonesia.Maluku
Hippotion brennus|SPTMB507-11|BC-Mel1509|658[0n]bp|Indonesia.Maluku
Hippotion brennus|SPTMB499-11|BC-Mel1501|658[0n]bp|Indonesia.Maluku
Hippotion brennus|SPTMB504-11|BC-Mel1506|658[0n]bp|Indonesia.Maluku
Hippotion brennus|SPTMB505-11|BC-Mel1507|658[0n]bp|Indonesia.Maluku
Hippotion brennus|SPTMA197-07|BC-Mel 0275|658[0n]bp|Indonesia.Papua
Hippotion brennus|SPTMC562-12|BC-Mel2563|658[0n]bp|Indonesia.Maluku
Hippotion brennus|GWORB980-07|BC ZSM Lep 02202|637[0n]bp|Indonesia.Papua
Hippotion brennus|GWORY462-10|BC EF Lep 03416|658[0n]bp|Australia.Queensland
Hippotion brennus|SPTMB509-11|BC-Mel1511|658[0n]bp|Indonesia.Maluku
Hippotion brennus|ANICC031-08|ANIC Gen No. 003194|614[0n]bp|Australia.Queensland
Hippotion brennus|ANICC030-08|ANIC Gen No. 003193|613[0n]bp|Australia.Queensland
Hippotion joiceyi|SPTMA198-07|BC-Mel 0276|658[0n]bp|Papua New Guinea
Hippotion joiceyi|SOWE239-07|BC-Hax4138|639[0n]bp|Indonesia.Papua
Hippotion joiceyi|SOWE238-07|BC-Hax4137|610[0n]bp|Indonesia.Papua
Hippotion brennus|HCPN006-03|USNM ENT 00678974|600[0n]bp|Papua New Guinea.Madang
Hippotion brennus|SPTMB497-11|BC-Mel1499|658[0n]bp|Indonesia.Papua
Hippotion brennus|HCPN007-03|USNM ENT 00678973|585[1n]bp|Papua New Guinea.Madang
Hippotion brennus|SPTMA923-09|BC-Mel 1001|658[0n]bp|Indonesia.Papua
Hippotion brennus|SML159-06|USNM ENT 00196614|658[0n]bp|Papua New Guinea.Morobe
Hippotion brennus|SML160-06|USNM ENT 00196615|658[0n]bp|Papua New Guinea.Morobe
Hippotion brennus|SPTMB496-11|BC-Mel1498|658[0n]bp|Indonesia.Papua
Hippotion brennus|SPHJT147-11|BC-LTM-169|658[0n]bp|Australia.Queensland
Hippotion brennus|SPTOL159-07|AYK-04-0398|658[0n]bp|Papua New Guinea.Chimbu
Hippotion brennus|HCPN008-03|USNM ENT 00678972|639[2n]bp|Papua New Guinea.Madang
Hippotion brennus|SPTMB495-11|BC-Mel1497|658[0n]bp|Indonesia.Papua
Hippotion brennus|SPTMA922-09|BC-Mel 1000|643[0n]bp|Australia.Queensland
Hippotion brennus|SOWE237-07|BC-Hax4136|609[0n]bp|Indonesia.Papua
Hippotion brennus|SML161-06|USNM ENT 00196616|642[0n]bp|Papua New Guinea.Gulf
Hippotion brennus|ANICC092-08|ANIC Gen No. 003255|658[0n]bp|Australia.Queensland
Hippotion brennus|SPHJT149-11|BC-LTM-171|658[0n]bp|Australia.New South Wales
Hippotion brennus|LOQTE455-09|gvc12757-1L|658[0n]bp|Australia.Queensland
Hippotion rosetta|SOWE240-07|BC-Hax4139|658[0n]bp|Indonesia.Papua
Hippotion boerhaviae|SOWF109-12|BC-Hax4967|658[0n]bp|New Caledonia.South
Hippotion boerhaviae|SOWF110-12|BC-Hax4968|658[0n]bp|New Caledonia.South
Hippotion rosetta|LOQC040-05|05-QLD-00040|519[0n]bp|Australia.Queensland
Hippotion rosetta|ANICC029-08|ANIC Gen No. 003192|609[0n]bp|Australia.Queensland
Hippotion rosetta|ANICC028-08|ANIC Gen No. 003191|622[0n]bp|Australia.Queensland
Hippotion rosetta|GWORY463-10|BC EF Lep 03417|658[0n]bp|Australia.Queensland
Hippotion rosetta|SPHJT148-11|BC-LTM-170|658[0n]bp|Australia.Queensland
Hippotion rosetta|ANICC093-08|ANIC Gen No. 003256|609[0n]bp|Australia.Queensland
Hippotion rosetta|LOQC039-05|05-QLD-00039|658[0n]bp|Australia.Queensland
Hippotion rosetta|LOQTE407-09|gvc12482-1L|658[0n]bp|Australia.Queensland
Hippotion rosetta|SOWD603-06|BC-Hax3502|607[0n]bp|Philippines.Benguet
Hippotion rosetta|SPHJT150-11|BC-LTM-173|658[0n]bp|Australia.Queensland
Hippotion boerhaviae|SPTMB667-11|BC-Mel1669|658[0n]bp|Philippines
Hippotion rosetta|LOQTE888-10|gvc14422-1L|658[0n]bp|Australia.Queensland
Hippotion rosetta|LOQTE247-09|gvc11839-1L|658[0n]bp|Australia.Queensland
Hippotion rosetta|LOQTE032-09|gvc11402-1L|658[1n]bp|Australia.Queensland
Hippotion rosetta|PMANK044-06|USNM ENT 00196446|589[6n]bp|Myanmar.Sagaing
Hippotion rosetta|LOQTE907-10|gvc14453-1L|658[0n]bp|Australia.Queensland
Hippotion boerhaviae|SPTMB116-10|BC-Mel1118|658[0n]bp|India.Karnataka
Hippotion rosetta|LOQTD526-08|gvc9138-1L|658[0n]bp|Australia.Queensland
Hippotion rosetta|SPTMB668-11|BC-Mel1670|658[0n]bp|Australia.Northern Territory
Hippotion rosetta|SPHJT146-11|BC-LTM-168|658[0n]bp|Australia.Queensland
Hippotion rosetta|GWORD048-07|BC ZSM Lep 02680|656[0n]bp|Australia.Queensland

Hippotion rosetta|SPTMB668-11|BC-Mel1670|658[0n]bp|Australia.Northern Territory
Hippotion rosetta|SPHJT146-11|BC-LTM-168|658[0n]bp|Australia.Queensland
Hippotion rosetta|GWORD048-07|BC ZSM Lep 02680|656[0n]bp|Australia.Queensland
Hippotion boerhaviae|SPTMB662-11|BC-Mel1664|658[0n]bp|Indonesia.Bali
Hippotion boerhaviae|SPTMB666-11|BC-Mel1668|658[0n]bp|Philippines
Hippotion rosetta|SPHJT145-11|BC-LTM-167|658[0n]bp|Australia.Queensland
Hippotion rosetta|LOQTE112-09|gvc11555-1L|658[0n]bp|Australia.Queensland
Hippotion rosetta|SOWE241-07|BC-Hax4140|658[0n]bp|Indonesia.Papua
Hippotion rosetta|SPTMB669-11|BC-Mel1671|658[0n]bp|Australia.Western Australia
Hippotion rosetta|LOQB451-05|Moth 135.03CC|596[0n]bp|Australia.Queensland
Hippotion rosetta|SPHJT151-11|BC-LTM-172|658[0n]bp|Australia.Queensland
Hippotion rosetta|GWORY465-10|BC EF Lep 03419|658[0n]bp|Australia.Queensland
Hippotion rosetta|LOQTB001-07|gvc6770-1L|658[0n]bp|Australia.Queensland
Hippotion rosetta|GWORY466-10|BC EF Lep 03420|658[0n]bp|Australia.Queensland
Hippotion rosetta|LOQTB732-07|gvc7425-1L|658[0n]bp|Australia.Queensland
Hippotion rosetta|LOQTE728-10|gvc13945-1L|658[0n]bp|Australia.Queensland
Hippotion boerhaviae|SPTMB660-11|BC-Mel1662|658[0n]bp|Indonesia.Bali
Hippotion boerhaviae|SPTMB661-11|BC-Mel1663|658[0n]bp|Indonesia.Bali
Hippotion boerhaviae|SPHAP045-06|MA05-08-23-74|603[0n]bp|India.Maharashtra
Hippotion boerhaviae|SPTMB042-09|BC-Mel 1054|658[0n]bp|India.Karnataka
Hippotion boerhaviae|SPTMB058-09|BC-Mel 1070|658[0n]bp|India.Karnataka
Hippotion boerhaviae|SPTMB115-10|BC-Mel1117|658[0n]bp|India.Karnataka
Hippotion boerhaviae|SPTMB114-10|BC-Mel1116|658[0n]bp|India.Karnataka
Hippotion rosetta|SOWD602-06|BC-Hax3501|607[0n]bp|Malaysia.Sabah
Hippotion boerhaviae|SPTMB060-09|BC-Mel 1072|658[0n]bp|India.Karnataka
Hippotion boerhaviae|SOWF232-12|BC-Hax5090|658[0n]bp|Laos.Khammouan
Hippotion boerhaviae|SPTMB665-11|BC-Mel1667|658[0n]bp|India.Karnataka
Hippotion scrofa|LNSWE135-06|06-NSWE-00135|658[0n]bp|Australia.New South Wales
Hippotion scrofa|GWORI552-09|BC ZSM Lep 13300|658[0n]bp|Australia.Northern Territory
Hippotion scrofa|NSWHM2102-11|BIOUG00961-B01|658[0n]bp|Australia.New South Wales
Hippotion scrofa|LNSWE086-06|06-NSWE-00086|600[0n]bp|Australia.New South Wales
Hippotion scrofa|LNSWE026-06|06-NSWE-00026|658[0n]bp|Australia.New South Wales
Hippotion scrofa|LNSWE083-06|06-NSWE-00083|658[0n]bp|Australia.New South Wales
Hippotion scrofa|NSWHJ619-10|09-NSWHH-0632|658[0n]bp|Australia.New South Wales
Hippotion scrofa|LOQT486-06|gvc6313-1L|658[0n]bp|Australia.Queensland
Hippotion scrofa|AMWW022-11|K290762|658[0n]bp|Australia.New South Wales
Hippotion scrofa|LNSWC308-06|06-NSW-00308|658[0n]bp|Australia.New South Wales
Hippotion scrofa|NSWHH050-09|08-NSWHH-0050|658[0n]bp|Australia.New South Wales
Hippotion scrofa|NSWHJ962-10|09-NSWHH-0975|658[0n]bp|Australia.New South Wales
Hippotion scrofa|SPRBA166-08|BC-RBP-0166|658[0n]bp|Australia.Queensland
Hippotion scrofa|LOQTC419-07|gvc8055-1L|658[0n]bp|Australia.Queensland
Hippotion scrofa|LNSWB126-05|05-NSW-01066|658[0n]bp|Australia.New South Wales
Hippotion scrofa|LNSWF492-06|06-NSWE-01432|658[0n]bp|Australia.New South Wales
Hippotion scrofa|LOQTI638-12|gvc17316-1L|658[0n]bp|Australia.Queensland
Hippotion scrofa|NSWHJ965-10|09-NSWHH-0978|658[0n]bp|Australia.New South Wales
Hippotion scrofa|GWORN203-09|BC ZSM Lep 18231|658[0n]bp|Australia.Western Australia
Hippotion scrofa|LNSWE046-06|06-NSWE-00046|658[0n]bp|Australia.New South Wales
Hippotion scrofa|LNSWE074-06|06-NSWE-00074|658[0n]bp|Australia.New South Wales
Hippotion scrofa|LNSWC306-06|06-NSW-00306|658[0n]bp|Australia.New South Wales
Hippotion scrofa|NSWHH058-09|08-NSWHH-0058|658[0n]bp|Australia.New South Wales
Hippotion scrofa|NSWHM2154-11|BIOUG00961-F05|658[0n]bp|Australia.New South Wales
Hippotion scrofa|GWORN205-09|BC ZSM Lep 18233|658[0n]bp|Australia.Western Australia
Hippotion scrofa|LNSWC637-08|AM 2358|658[0n]bp|Australia.New South Wales
Hippotion scrofa|LNSWC638-08|AM 2359|658[0n]bp|Australia.New South Wales
Hippotion scrofa|NSWHH051-09|08-NSWHH-0051|658[0n]bp|Australia.New South Wales
Hippotion scrofa|NSWHM2119-11|BIOUG00961-C06|658[0n]bp|Australia.New South Wales
Hippotion scrofa|PHLCC1142-11|BIOUG01235-A06|658[0n]bp|Australia.Australian Capital Territory
Hippotion scrofa|LSM1704-11|am10638|658[0n]bp|Australia.New South Wales
Hippotion scrofa|LCANA353-06|05-ACTC-353|658[0n]bp|Australia.Australian Capital Territory
Hippotion scrofa|IMLQ233-07|IM07-0165|658[0n]bp|Australia.Queensland
Hippotion scrofa|SPRBA756-09|BC-RBP-1800|658[0n]bp|Australia.Queensland
Hippotion scrofa|LNSWE025-06|06-NSWE-00025|658[0n]bp|Australia.New South Wales
Hippotion scrofa|LNSWC307-06|06-NSW-00307|658[0n]bp|Australia.New South Wales
Hippotion scrofa|NSWHM017-11|BIOUG00851-G10|658[0n]bp|Australia.New South Wales
Hippotion scrofa|LNSWE059-06|06-NSWE-00059|658[0n]bp|Australia.New South Wales
Hippotion scrofa|LNSWE057-06|06-NSWE-00057|658[0n]bp|Australia.New South Wales
Hippotion scrofa|NSWHM451-11|BIOUG00912-F12|658[0n]bp|Australia.New South Wales
Hippotion scrofa|NSWHJ964-10|09-NSWHH-0977|658[0n]bp|Australia.New South Wales
Hippotion scrofa|NSWHH053-09|08-NSWHH-0053|658[0n]bp|Australia.New South Wales
Hippotion scrofa|NSWBB1216-08|07-NSWBB-1216|656[0n]bp|Australia.New South Wales
Hippotion scrofa|IMLQ127-07|IM06-0428|656[0n]bp|Australia.Queensland
Hippotion scrofa|LNSWE078-06|06-NSWE-00078|595[0n]bp|Australia.New South Wales
Hippotion scrofa|NSWHJ191-10|09-NSWHH-0204|638[0n]bp|Australia.New South Wales
Hippotion scrofa|LNSWE111-06|06-NSWE-00111|550[1n]bp|Australia.New South Wales
Hippotion scrofa|AMWW379-12|K292664|591[0n]bp|Australia.New South Wales
Hippotion scrofa|LNSWE005-06|06-NSWE-00005|564[0n]bp|Australia.New South Wales
Hippotion scrofa|LNSWE124-06|06-NSWE-00124|603[0n]bp|Australia.New South Wales
Hippotion scrofa|GWORC178-07|BC ZSM Lep 02528|595[0n]bp|Australia.Queensland
Hippotion scrofa|LNSWE105-06|06-NSWE-00105|596[0n]bp|Australia.New South Wales
Hippotion scrofa|LNSWE095-06|06-NSWE-00095|595[0n]bp|Australia.New South Wales
Hippotion scrofa|LNSWE097-06|06-NSWE-00097|595[0n]bp|Australia.New South Wales
Hippotion scrofa|LNSWE112-06|06-NSWE-00112|595[1n]bp|Australia.New South Wales
Hippotion scrofa|LNSWE117-06|06-NSWE-00117|595[1n]bp|Australia.New South Wales
Hippotion scrofa|LNSWE076-06|06-NSWE-00076|581[0n]bp|Australia.New South Wales
Hippotion scrofa|LNSWE063-06|06-NSWE-00063|577[0n]bp|Australia.New South Wales
Hippotion scrofa|LNSWE120-06|06-NSWE-00120|578[2n]bp|Australia.New South Wales
Hippotion scrofa|LNSWE049-06|06-NSWE-00049|549[0n]bp|Australia.New South Wales
Hippotion scrofa|LNSWE104-06|06-NSWE-00104|594[0n]bp|Australia.New South Wales
Hippotion scrofa|LNSWE103-06|06-NSWE-00103|538[0n]bp|Australia.New South Wales
Hippotion scrofa|LNSWE113-06|06-NSWE-00113|658[0n]bp|Australia.New South Wales
Hippotion scrofa|SOWD679-06|BC-Hax3578|607[0n]bp|New Caledonia
Hippotion scrofa|SPRBA167-08|BC-RBP-0167|635[0n]bp|New Caledonia
Hippotion scrofa|SPRBA168-08|BC-RBP-0168|601[1n]bp|Fiji.Viti Levu Island
Macroglossum corythus|ANICC053-08|ANIC Gen No. 003216|263[0n]bp|Australia.Queensland
Eupanacra splendens paradoxa|SPTMB579-11|BC-Mel1581|658[0n]bp|Indonesia.Maluku
Eupanacra splendens paradoxa|SPTMB047-09|BC-Mel 1059|658[0n]bp|Indonesia.Maluku
Eupanacra splendens|SPRBA559-09|BC-RBP-1217|658[0n]bp|Indonesia.Maluku
Eupanacra splendens|SPRBA561-09|BC-RBP-1219|658[0n]bp|Papua New Guinea.West New Britain
Eupanacra splendens|SPRBA560-09|BC-RBP-1218|658[0n]bp|Papua New Guinea
Eupanacra splendens|PMANL1970-12|USNM ENT 00510594|658[0n]bp|Papua New Guinea.Madang
Eupanacra splendens|EPNG3394-11|USNM ENT 00697250|614[0n]bp|Papua New Guinea.Madang
Eupanacra splendens|LOQTI673-12|gvc17580-1L|638[0n]bp|Australia.Queensland
[illegible]

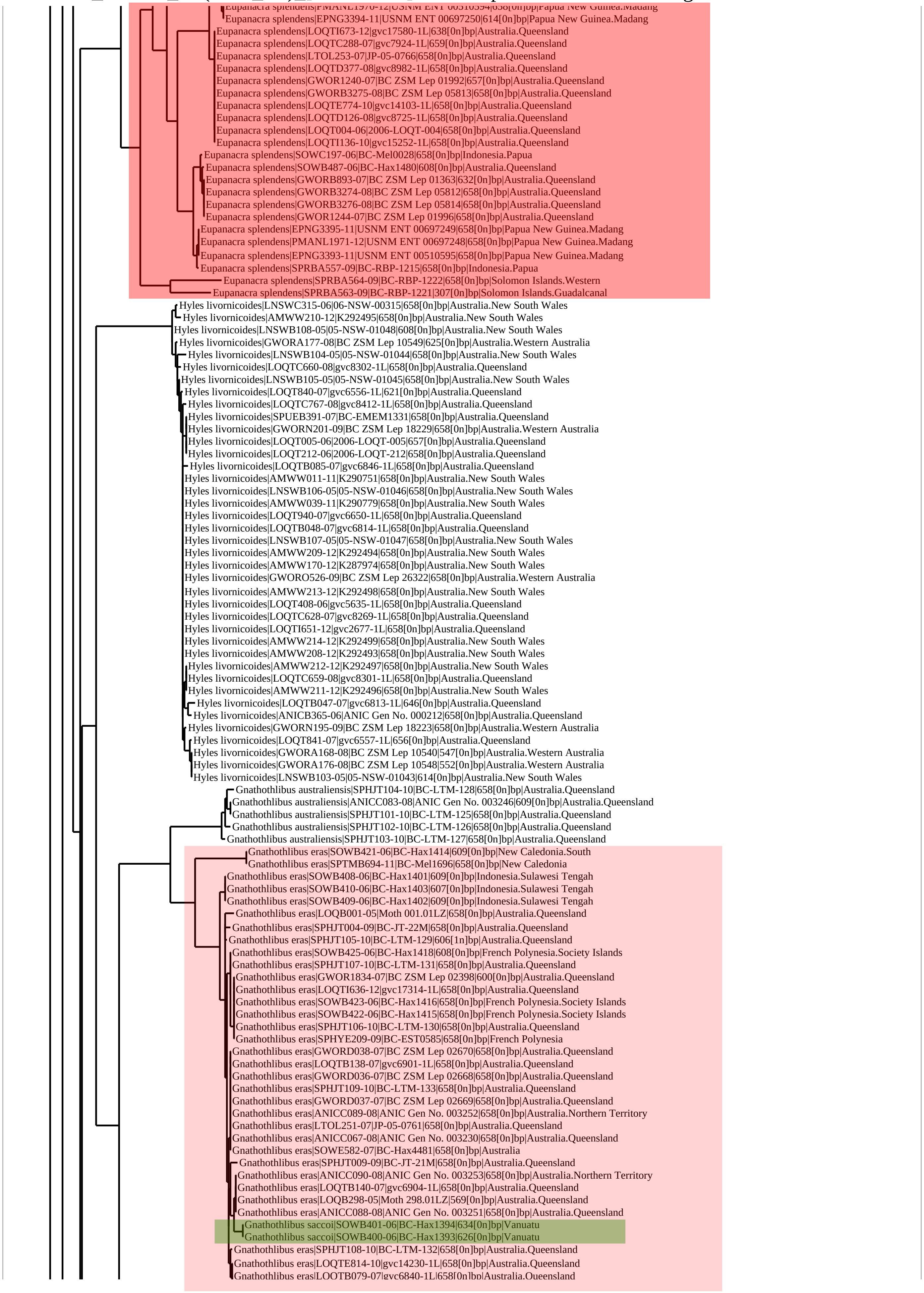
Eupanacra splendens|PMANL1970-12|USNM ENT 00510594|658[0n]bp|Papua New Guinea.Madang
Eupanacra splendens|EPNG3394-11|USNM ENT 00697250|614[0n]bp|Papua New Guinea.Madang
Eupanacra splendens|LOQTI673-12|gvc17580-1L|638[0n]bp|Australia.Queensland
Eupanacra splendens|LOQTC288-07|gvc7924-1L|659[0n]bp|Australia.Queensland
Eupanacra splendens|LTOL253-07|JP-05-0766|658[0n]bp|Australia.Queensland
Eupanacra splendens|LOQTD377-08|gvc8982-1L|658[0n]bp|Australia.Queensland
Eupanacra splendens|GWOR1240-07|BC ZSM Lep 01992|657[0n]bp|Australia.Queensland
Eupanacra splendens|GWORB3275-08|BC ZSM Lep 05813|658[0n]bp|Australia.Queensland
Eupanacra splendens|LOQTE774-10|gvc14103-1L|658[0n]bp|Australia.Queensland
Eupanacra splendens|LOQTD126-08|gvc8725-1L|658[0n]bp|Australia.Queensland
Eupanacra splendens|LOQT004-06|2006-LOQT-004|658[0n]bp|Australia.Queensland
Eupanacra splendens|LOQTI136-10|gvc15252-1L|658[0n]bp|Australia.Queensland
Eupanacra splendens|SOWC197-06|BC-Mel0028|658[0n]bp|Indonesia.Papua
Eupanacra splendens|SOWB487-06|BC-Hax1480|608[0n]bp|Australia.Queensland
Eupanacra splendens|GWORB893-07|BC ZSM Lep 01363|632[0n]bp|Australia.Queensland
Eupanacra splendens|GWORB3274-08|BC ZSM Lep 05812|658[0n]bp|Australia.Queensland
Eupanacra splendens|GWORB3276-08|BC ZSM Lep 05814|658[0n]bp|Australia.Queensland
Eupanacra splendens|GWOR1244-07|BC ZSM Lep 01996|658[0n]bp|Australia.Queensland
Eupanacra splendens|EPNG3395-11|USNM ENT 00697249|658[0n]bp|Papua New Guinea.Madang
Eupanacra splendens|PMANL1971-12|USNM ENT 00697248|658[0n]bp|Papua New Guinea.Madang
Eupanacra splendens|EPNG3393-11|USNM ENT 00510595|658[0n]bp|Papua New Guinea.Madang
Eupanacra splendens|SPRBA557-09|BC-RBP-1215|658[0n]bp|Indonesia.Papua
Eupanacra splendens|SPRBA564-09|BC-RBP-1222|658[0n]bp|Solomon Islands.Western
Eupanacra splendens|SPRBA563-09|BC-RBP-1221|307[0n]bp|Solomon Islands.Guadalcanal
Hyles livornicoides|LNSWC315-06|06-NSW-00315|658[0n]bp|Australia.New South Wales
Hyles livornicoides|AMWW210-12|K292495|658[0n]bp|Australia.New South Wales
Hyles livornicoides|LNSWB108-05|05-NSW-01048|608[0n]bp|Australia.New South Wales
Hyles livornicoides|GWORA177-08|BC ZSM Lep 10549|625[0n]bp|Australia.Western Australia
Hyles livornicoides|LNSWB104-05|05-NSW-01044|658[0n]bp|Australia.New South Wales
Hyles livornicoides|LOQTC660-08|gvc8302-1L|658[0n]bp|Australia.Queensland
Hyles livornicoides|LNSWB105-05|05-NSW-01045|658[0n]bp|Australia.New South Wales
Hyles livornicoides|LOQT840-07|gvc6556-1L|621[0n]bp|Australia.Queensland
Hyles livornicoides|LOQTC767-08|gvc8412-1L|658[0n]bp|Australia.Queensland
Hyles livornicoides|SPUEB391-07|BC-EMEM1331|658[0n]bp|Australia.Queensland
Hyles livornicoides|GWORN201-09|BC ZSM Lep 18229|658[0n]bp|Australia.Western Australia
Hyles livornicoides|LOQT005-06|2006-LOQT-005|657[0n]bp|Australia.Queensland
Hyles livornicoides|LOQT212-06|2006-LOQT-212|658[0n]bp|Australia.Queensland
Hyles livornicoides|LOQTB085-07|gvc6846-1L|658[0n]bp|Australia.Queensland
Hyles livornicoides|AMWW011-11|K290751|658[0n]bp|Australia.New South Wales
Hyles livornicoides|LNSWB106-05|05-NSW-01046|658[0n]bp|Australia.New South Wales
Hyles livornicoides|AMWW039-11|K290779|658[0n]bp|Australia.New South Wales
Hyles livornicoides|LOQT940-07|gvc6650-1L|658[0n]bp|Australia.Queensland
Hyles livornicoides|LOQTB048-07|gvc6814-1L|658[0n]bp|Australia.Queensland
Hyles livornicoides|LNSWB107-05|05-NSW-01047|658[0n]bp|Australia.New South Wales
Hyles livornicoides|AMWW209-12|K292494|658[0n]bp|Australia.New South Wales
Hyles livornicoides|AMWW170-12|K287974|658[0n]bp|Australia.New South Wales
Hyles livornicoides|GWORO526-09|BC ZSM Lep 26322|658[0n]bp|Australia.Western Australia
Hyles livornicoides|AMWW213-12|K292498|658[0n]bp|Australia.New South Wales
Hyles livornicoides|LOQT408-06|gvc5635-1L|658[0n]bp|Australia.Queensland
Hyles livornicoides|LOQTC628-07|gvc8269-1L|658[0n]bp|Australia.Queensland
Hyles livornicoides|LOQTI651-12|gvc2677-1L|658[0n]bp|Australia.Queensland
Hyles livornicoides|AMWW214-12|K292499|658[0n]bp|Australia.New South Wales
Hyles livornicoides|AMWW208-12|K292493|658[0n]bp|Australia.New South Wales
Hyles livornicoides|AMWW212-12|K292497|658[0n]bp|Australia.New South Wales
Hyles livornicoides|LOQTC659-08|gvc8301-1L|658[0n]bp|Australia.Queensland
Hyles livornicoides|AMWW211-12|K292496|658[0n]bp|Australia.New South Wales
Hyles livornicoides|LOQTB047-07|gvc6813-1L|646[0n]bp|Australia.Queensland
Hyles livornicoides|ANICB365-06|ANIC Gen No. 000212|658[0n]bp|Australia.Queensland
Hyles livornicoides|GWORN195-09|BC ZSM Lep 18223|658[0n]bp|Australia.Western Australia
Hyles livornicoides|LOQT841-07|gvc6557-1L|656[0n]bp|Australia.Queensland
Hyles livornicoides|GWORA168-08|BC ZSM Lep 10540|547[0n]bp|Australia.Western Australia
Hyles livornicoides|GWORA176-08|BC ZSM Lep 10548|552[0n]bp|Australia.Western Australia
Hyles livornicoides|LNSWB103-05|05-NSW-01043|614[0n]bp|Australia.New South Wales
Gnathothlibus australiensis|SPHJT104-10|BC-LTM-128|658[0n]bp|Australia.Queensland
Gnathothlibus australiensis|ANICC083-08|ANIC Gen No. 003246|609[0n]bp|Australia.Queensland
Gnathothlibus australiensis|SPHJT101-10|BC-LTM-125|658[0n]bp|Australia.Queensland
Gnathothlibus australiensis|SPHJT102-10|BC-LTM-126|658[0n]bp|Australia.Queensland
Gnathothlibus australiensis|SPHJT103-10|BC-LTM-127|658[0n]bp|Australia.Queensland
Gnathothlibus eras|SOWB421-06|BC-Hax1414|609[0n]bp|New Caledonia.South
Gnathothlibus eras|SPTMB694-11|BC-Mel1696|658[0n]bp|New Caledonia
Gnathothlibus eras|SOWB408-06|BC-Hax1401|609[0n]bp|Indonesia.Sulawesi Tengah
Gnathothlibus eras|SOWB410-06|BC-Hax1403|607[0n]bp|Indonesia.Sulawesi Tengah
Gnathothlibus eras|SOWB409-06|BC-Hax1402|609[0n]bp|Indonesia.Sulawesi Tengah
Gnathothlibus eras|LOQB001-05|Moth 001.01LZ|658[0n]bp|Australia.Queensland
Gnathothlibus eras|SPHJT004-09|BC-JT-22M|658[0n]bp|Australia.Queensland
Gnathothlibus eras|SPHJT105-10|BC-LTM-129|606[1n]bp|Australia.Queensland
Gnathothlibus eras|SOWB425-06|BC-Hax1418|608[0n]bp|French Polynesia.Society Islands
Gnathothlibus eras|SPHJT107-10|BC-LTM-131|658[0n]bp|Australia.Queensland
Gnathothlibus eras|GWOR1834-07|BC ZSM Lep 02398|600[0n]bp|Australia.Queensland
Gnathothlibus eras|LOQTI636-12|gvc17314-1L|658[0n]bp|Australia.Queensland
Gnathothlibus eras|SOWB423-06|BC-Hax1416|658[0n]bp|French Polynesia.Society Islands
Gnathothlibus eras|SOWB422-06|BC-Hax1415|658[0n]bp|French Polynesia.Society Islands
Gnathothlibus eras|SPHJT106-10|BC-LTM-130|658[0n]bp|Australia.Queensland
Gnathothlibus eras|SPHYE209-09|BC-EST0585|658[0n]bp|French Polynesia
Gnathothlibus eras|GWORD038-07|BC ZSM Lep 02670|658[0n]bp|Australia.Queensland
Gnathothlibus eras|LOQTB138-07|gvc6901-1L|658[0n]bp|Australia.Queensland
Gnathothlibus eras|GWORD036-07|BC ZSM Lep 02668|658[0n]bp|Australia.Queensland
Gnathothlibus eras|SPHJT109-10|BC-LTM-133|658[0n]bp|Australia.Queensland
Gnathothlibus eras|GWORD037-07|BC ZSM Lep 02669|658[0n]bp|Australia.Queensland
Gnathothlibus eras|ANICC089-08|ANIC Gen No. 003252|658[0n]bp|Australia.Northern Territory
Gnathothlibus eras|LTOL251-07|JP-05-0761|658[0n]bp|Australia.Queensland
Gnathothlibus eras|ANICC067-08|ANIC Gen No. 003230|658[0n]bp|Australia.Queensland
Gnathothlibus eras|SOWE582-07|BC-Hax4481|658[0n]bp|Australia
Gnathothlibus eras|SPHJT009-09|BC-JT-21M|658[0n]bp|Australia.Queensland
Gnathothlibus eras|ANICC090-08|ANIC Gen No. 003253|658[0n]bp|Australia.Northern Territory
Gnathothlibus eras|LOQTB140-07|gvc6904-1L|658[0n]bp|Australia.Queensland
Gnathothlibus eras|LOQB298-05|Moth 298.01LZ|569[0n]bp|Australia.Queensland
Gnathothlibus eras|ANICC088-08|ANIC Gen No. 003251|658[0n]bp|Australia.Queensland
Gnathothlibus saccoi|SOWB401-06|BC-Hax1394|634[0n]bp|Vanuatu
Gnathothlibus saccoi|SOWB400-06|BC-Hax1393|626[0n]bp|Vanuatu
Gnathothlibus eras|SPHJT108-10|BC-LTM-132|658[0n]bp|Australia.Queensland
Gnathothlibus eras|LOQTE814-10|gvc14230-1L|658[0n]bp|Australia.Queensland
Gnathothlibus eras|LOQTB079-07|gvc6840-1L|658[0n]bp|Australia.Queensland

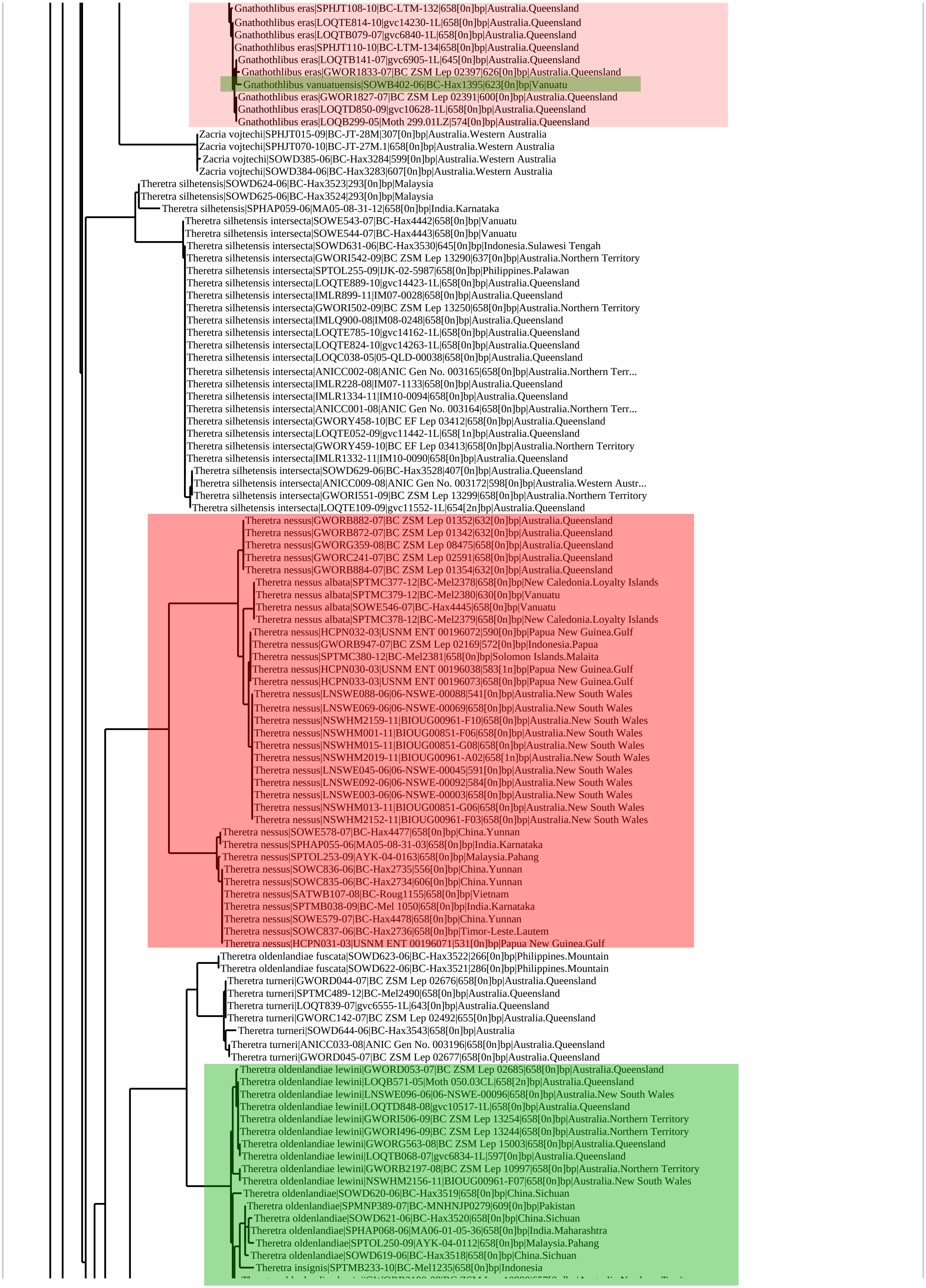
Gnathothlibus eras|SPHJT108-10|BC-LTM-132|658[0n]bp|Australia.Queensland
Gnathothlibus eras|LOQTE814-10|gvc14230-1L|658[0n]bp|Australia.Queensland
Gnathothlibus eras|LOQTB079-07|gvc6840-1L|658[0n]bp|Australia.Queensland
Gnathothlibus eras|SPHJT110-10|BC-LTM-134|658[0n]bp|Australia.Queensland
Gnathothlibus eras|LOQTB141-07|gvc6905-1L|645[0n]bp|Australia.Queensland
Gnathothlibus eras|GWOR1833-07|BC ZSM Lep 02397|626[0n]bp|Australia.Queensland
Gnathothlibus vanuatuensis|SOWB402-06|BC-Hax1395|623[0n]bp|Vanuatu
Gnathothlibus eras|GWOR1827-07|BC ZSM Lep 02391|600[0n]bp|Australia.Queensland
Gnathothlibus eras|LOQTD850-09|gvc10628-1L|658[0n]bp|Australia.Queensland
Gnathothlibus eras|LOQB299-05|Moth 299.01LZ|574[0n]bp|Australia.Queensland
Zacria vojtechi|SPHJT015-09|BC-JT-28M|307[0n]bp|Australia.Western Australia
Zacria vojtechi|SPHJT070-10|BC-JT-27M.1|658[0n]bp|Australia.Western Australia
Zacria vojtechi|SOWD385-06|BC-Hax3284|599[0n]bp|Australia.Western Australia
Zacria vojtechi|SOWD384-06|BC-Hax3283|607[0n]bp|Australia.Western Australia
Theretra silhetensis|SOWD624-06|BC-Hax3523|293[0n]bp|Malaysia
Theretra silhetensis|SOWD625-06|BC-Hax3524|293[0n]bp|Malaysia
Theretra silhetensis|SPHAP059-06|MA05-08-31-12|658[0n]bp|India.Karnataka
Theretra silhetensis intersecta|SOWE543-07|BC-Hax4442|658[0n]bp|Vanuatu
Theretra silhetensis intersecta|SOWE544-07|BC-Hax4443|658[0n]bp|Vanuatu
Theretra silhetensis intersecta|SOWD631-06|BC-Hax3530|645[0n]bp|Indonesia.Sulawesi Tengah
Theretra silhetensis intersecta|GWORI542-09|BC ZSM Lep 13290|637[0n]bp|Australia.Northern Territory
Theretra silhetensis intersecta|SPTOL255-09|IJK-02-5987|658[0n]bp|Philippines.Palawan
Theretra silhetensis intersecta|LOQTE889-10|gvc14423-1L|658[0n]bp|Australia.Queensland
Theretra silhetensis intersecta|IMLR899-11|IM07-0028|658[0n]bp|Australia.Queensland
Theretra silhetensis intersecta|GWORI502-09|BC ZSM Lep 13250|658[0n]bp|Australia.Northern Territory
Theretra silhetensis intersecta|IMLQ900-08|IM08-0248|658[0n]bp|Australia.Queensland
Theretra silhetensis intersecta|LOQTE785-10|gvc14162-1L|658[0n]bp|Australia.Queensland
Theretra silhetensis intersecta|LOQTE824-10|gvc14263-1L|658[0n]bp|Australia.Queensland
Theretra silhetensis intersecta|LOQC038-05|05-QLD-00038|658[0n]bp|Australia.Queensland
Theretra silhetensis intersecta|ANICC002-08|ANIC Gen No. 003165|658[0n]bp|Australia.Northern Terr...
Theretra silhetensis intersecta|IMLR228-08|IM07-1133|658[0n]bp|Australia.Queensland
Theretra silhetensis intersecta|IMLR1334-11|IM10-0094|658[0n]bp|Australia.Queensland
Theretra silhetensis intersecta|ANICC001-08|ANIC Gen No. 003164|658[0n]bp|Australia.Northern Terr...
Theretra silhetensis intersecta|GWORY458-10|BC EF Lep 03412|658[0n]bp|Australia.Queensland
Theretra silhetensis intersecta|LOQTE052-09|gvc11442-1L|658[1n]bp|Australia.Queensland
Theretra silhetensis intersecta|GWORY459-10|BC EF Lep 03413|658[0n]bp|Australia.Northern Territory
Theretra silhetensis intersecta|IMLR1332-11|IM10-0090|658[0n]bp|Australia.Queensland
Theretra silhetensis intersecta|SOWD629-06|BC-Hax3528|407[0n]bp|Australia.Queensland
Theretra silhetensis intersecta|ANICC009-08|ANIC Gen No. 003172|598[0n]bp|Australia.Western Austr...
Theretra silhetensis intersecta|GWORI551-09|BC ZSM Lep 13299|658[0n]bp|Australia.Northern Territory
Theretra silhetensis intersecta|LOQTE109-09|gvc11552-1L|654[2n]bp|Australia.Queensland
Theretra nessus|GWORB882-07|BC ZSM Lep 01352|632[0n]bp|Australia.Queensland
Theretra nessus|GWORB872-07|BC ZSM Lep 01342|632[0n]bp|Australia.Queensland
Theretra nessus|GWORG359-08|BC ZSM Lep 08475|658[0n]bp|Australia.Queensland
Theretra nessus|GWORC241-07|BC ZSM Lep 02591|658[0n]bp|Australia.Queensland
Theretra nessus|GWORB884-07|BC ZSM Lep 01354|632[0n]bp|Australia.Queensland
Theretra nessus albata|SPTMC377-12|BC-Mel2378|658[0n]bp|New Caledonia.Loyalty Islands
Theretra nessus albata|SPTMC379-12|BC-Mel2380|630[0n]bp|Vanuatu
Theretra nessus albata|SOWE546-07|BC-Hax4445|658[0n]bp|Vanuatu
Theretra nessus albata|SPTMC378-12|BC-Mel2379|658[0n]bp|New Caledonia.Loyalty Islands
Theretra nessus|HCPN032-03|USNM ENT 00196072|590[0n]bp|Papua New Guinea.Gulf
Theretra nessus|GWORB947-07|BC ZSM Lep 02169|572[0n]bp|Indonesia.Papua
Theretra nessus|SPTMC380-12|BC-Mel2381|658[0n]bp|Solomon Islands.Malaita
Theretra nessus|HCPN030-03|USNM ENT 00196038|583[1n]bp|Papua New Guinea.Gulf
Theretra nessus|HCPN033-03|USNM ENT 00196073|658[0n]bp|Papua New Guinea.Gulf
Theretra nessus|LNSWE088-06|06-NSWE-00088|541[0n]bp|Australia.New South Wales
Theretra nessus|LNSWE069-06|06-NSWE-00069|658[0n]bp|Australia.New South Wales
Theretra nessus|NSWHM2159-11|BIOUG00961-F10|658[0n]bp|Australia.New South Wales
Theretra nessus|NSWHM001-11|BIOUG00851-F06|658[0n]bp|Australia.New South Wales
Theretra nessus|NSWHM015-11|BIOUG00851-G08|658[0n]bp|Australia.New South Wales
Theretra nessus|NSWHM2019-11|BIOUG00961-A02|658[1n]bp|Australia.New South Wales
Theretra nessus|LNSWE045-06|06-NSWE-00045|591[0n]bp|Australia.New South Wales
Theretra nessus|LNSWE092-06|06-NSWE-00092|584[0n]bp|Australia.New South Wales
Theretra nessus|LNSWE003-06|06-NSWE-00003|658[0n]bp|Australia.New South Wales
Theretra nessus|NSWHM013-11|BIOUG00851-G06|658[0n]bp|Australia.New South Wales
Theretra nessus|NSWHM2152-11|BIOUG00961-F03|658[0n]bp|Australia.New South Wales
Theretra nessus|SOWE578-07|BC-Hax4477|658[0n]bp|China.Yunnan
Theretra nessus|SPHAP055-06|MA05-08-31-03|658[0n]bp|India.Karnataka
Theretra nessus|SPTOL253-09|AYK-04-0163|658[0n]bp|Malaysia.Pahang
Theretra nessus|SOWC836-06|BC-Hax2735|556[0n]bp|China.Yunnan
Theretra nessus|SOWC835-06|BC-Hax2734|606[0n]bp|China.Yunnan
Theretra nessus|SATWB107-08|BC-Roug1155|658[0n]bp|Vietnam
Theretra nessus|SPTMB038-09|BC-Mel 1050|658[0n]bp|India.Karnataka
Theretra nessus|SOWE579-07|BC-Hax4478|658[0n]bp|China.Yunnan
Theretra nessus|SOWC837-06|BC-Hax2736|658[0n]bp|Timor-Leste.Lautem
Theretra nessus|HCPN031-03|USNM ENT 00196071|531[0n]bp|Papua New Guinea.Gulf
Theretra oldenlandiae fuscata|SOWD623-06|BC-Hax3522|266[0n]bp|Philippines.Mountain
Theretra oldenlandiae fuscata|SOWD622-06|BC-Hax3521|286[0n]bp|Philippines.Mountain
Theretra turneri|GWORD044-07|BC ZSM Lep 02676|658[0n]bp|Australia.Queensland
Theretra turneri|SPTMC489-12|BC-Mel2490|658[0n]bp|Australia.Queensland
Theretra turneri|LOQT839-07|gvc6555-1L|643[0n]bp|Australia.Queensland
Theretra turneri|GWORC142-07|BC ZSM Lep 02492|655[0n]bp|Australia.Queensland
Theretra turneri|SOWD644-06|BC-Hax3543|658[0n]bp|Australia
Theretra turneri|ANICC033-08|ANIC Gen No. 003196|658[0n]bp|Australia.Queensland
Theretra turneri|GWORD045-07|BC ZSM Lep 02677|658[0n]bp|Australia.Queensland
Theretra oldenlandiae lewini|GWORD053-07|BC ZSM Lep 02685|658[0n]bp|Australia.Queensland
Theretra oldenlandiae lewini|LOQB571-05|Moth 050.03CL|658[2n]bp|Australia.Queensland
Theretra oldenlandiae lewini|LNSWE096-06|06-NSWE-00096|658[0n]bp|Australia.New South Wales
Theretra oldenlandiae lewini|LOQTD848-08|gvc10517-1L|658[0n]bp|Australia.Queensland
Theretra oldenlandiae lewini|GWORI506-09|BC ZSM Lep 13254|658[0n]bp|Australia.Northern Territory
Theretra oldenlandiae lewini|GWORI496-09|BC ZSM Lep 13244|658[0n]bp|Australia.Northern Territory
Theretra oldenlandiae lewini|GWORG563-08|BC ZSM Lep 15003|658[0n]bp|Australia.Queensland
Theretra oldenlandiae lewini|LOQTB068-07|gvc6834-1L|597[0n]bp|Australia.Queensland
Theretra oldenlandiae lewini|GWORB2197-08|BC ZSM Lep 10997|658[0n]bp|Australia.Northern Territory
Theretra oldenlandiae lewini|NSWHM2156-11|BIOUG00961-F07|658[0n]bp|Australia.New South Wales
Theretra oldenlandiae|SOWD620-06|BC-Hax3519|658[0n]bp|China.Sichuan
Theretra oldenlandiae|SPMNP389-07|BC-MNHNJP0279|609[0n]bp|Pakistan
Theretra oldenlandiae|SOWD621-06|BC-Hax3520|658[0n]bp|China.Sichuan
Theretra oldenlandiae|SPHAP068-06|MA06-01-05-36|658[0n]bp|India.Maharashtra
Theretra oldenlandiae|SPTOL250-09|AYK-04-0112|658[0n]bp|Malaysia.Pahang
Theretra oldenlandiae|SOWD619-06|BC-Hax3518|658[0n]bp|China.Sichuan
Theretra insignis|SPTMB233-10|BC-Mel1235|658[0n]bp|Indonesia

Theretra oldenlandiae|SPTOL250-09|ATK-04-0112|658[0n]bp|Malaysia.Pahang
Theretra oldenlandiae|SOWD619-06|BC-Hax3518|658[0n]bp|China.Sichuan
Theretra insignis|SPTMB233-10|BC-Mel1235|658[0n]bp|Indonesia
Theretra oldenlandiae lewini|GWORB2190-08|BC ZSM Lep 10990|657[0n]bp|Australia.Northern Territory
Theretra oldenlandiae lewini|GWORD052-07|BC ZSM Lep 02684|609[0n]bp|Australia.Queensland
Theretra oldenlandiae lewini|LOQT626-06|gvc5640-1L|656[0n]bp|Australia.Queensland
Theretra oldenlandiae lewini|NSWHM455-11|BIOUG00912-G04|658[0n]bp|Australia.New South Wales
Theretra oldenlandiae lewini|NSWHM032-11|BIOUG00851-F05|658[0n]bp|Australia.New South Wales
Theretra oldenlandiae lewini|NSWHM018-11|BIOUG00851-G11|658[0n]bp|Australia.New South Wales
Theretra oldenlandiae lewini|LNSWE082-06|06-NSWE-00082|511[0n]bp|Australia.New South Wales
Theretra insignis|SPTMB232-10|BC-Mel1234|658[0n]bp|Indonesia
Theretra insignis|SPTMB234-10|BC-Mel1236|658[0n]bp|Indonesia
Theretra insignis|SPTMB235-10|BC-Mel1237|658[0n]bp|Indonesia
Theretra insignis|SPTMB238-10|BC-Mel1240|658[0n]bp|Indonesia
Theretra oldenlandiae lewini|LNSWE119-06|06-NSWE-00119|510[1n]bp|Australia.New South Wales
Theretra insignis|SPTMB236-10|BC-Mel1238|658[0n]bp|Indonesia
Theretra oldenlandiae lewini|LNSWE007-06|06-NSWE-00007|598[0n]bp|Australia.New South Wales
Theretra oldenlandiae lewini|GWORD055-07|BC ZSM Lep 02687|658[0n]bp|Australia.Queensland
Theretra oldenlandiae lewini|GWORB865-07|BC ZSM Lep 01335|658[0n]bp|Australia.Queensland
Theretra oldenlandiae lewini|LOQTC768-08|gvc8413-1L|658[1n]bp|Australia.Queensland
Theretra oldenlandiae lewini|GWORB3268-08|BC ZSM Lep 05806|658[0n]bp|Australia.Queensland
Theretra oldenlandiae lewini|SPTMB536-11|BC-Mel1538|658[0n]bp|Indonesia.Sulawesi Utara
Theretra insignis|SPTMB237-10|BC-Mel1239|658[0n]bp|Indonesia
Theretra oldenlandiae lewini|LOQTE672-10|gvc13682-1L|614[0n]bp|Australia.Queensland
Theretra oldenlandiae lewini|LNSWE032-06|06-NSWE-00032|608[0n]bp|Australia.New South Wales
Theretra oldenlandiae lewini|LNSWE130-06|06-NSWE-00130|658[0n]bp|Australia.New South Wales
Theretra oldenlandiae lewini|LNSWE016-06|06-NSWE-00016|604[0n]bp|Australia.New South Wales
Theretra oldenlandiae lewini|LOQ346-04|04HBL004346|658[0n]bp|Australia.Queensland
Theretra oldenlandiae lewini|LNSWE091-06|06-NSWE-00091|658[0n]bp|Australia.New South Wales
Theretra oldenlandiae lewini|GWORD059-07|BC ZSM Lep 02691|658[0n]bp|Australia.Queensland
Theretra oldenlandiae lewini|LNSWE125-06|06-NSWE-00125|608[0n]bp|Australia.New South Wales
Theretra oldenlandiae lewini|GWORD056-07|BC ZSM Lep 02688|655[0n]bp|Australia.Queensland
Theretra oldenlandiae lewini|SOWE242-07|BC-Hax4141|647[0n]bp|Australia
Theretra oldenlandiae lewini|GWORI499-09|BC ZSM Lep 13247|658[0n]bp|Australia.Northern Territory
Theretra oldenlandiae lewini|GWORI548-09|BC ZSM Lep 13296|658[0n]bp|Australia.Northern Territory
Theretra oldenlandiae lewini|NSWHJ014-10|09-NSWHH-0027|658[0n]bp|Australia.New South Wales
Theretra oldenlandiae lewini|GWORD067-07|BC ZSM Lep 02699|658[0n]bp|Australia.Queensland
Theretra oldenlandiae lewini|LOQ345-04|04HBL004345|658[0n]bp|Australia.Queensland
Theretra oldenlandiae lewini|GWORB3243-08|BC ZSM Lep 05781|658[0n]bp|Australia.Queensland
Theretra oldenlandiae lewini|LOQTE588-10|gvc13322-1L|658[0n]bp|Australia.Queensland
Theretra oldenlandiae lewini|LOQTC417-07|gvc8053-1L|658[0n]bp|Australia.Queensland
Theretra oldenlandiae lewini|GWORD066-07|BC ZSM Lep 02698|658[0n]bp|Australia.Queensland
Theretra oldenlandiae lewini|LOQT453-06|gvc6234-1L|658[0n]bp|Australia.Queensland
Theretra oldenlandiae lewini|GWORD057-07|BC ZSM Lep 02689|658[0n]bp|Australia.Queensland
Theretra oldenlandiae lewini|GWORD062-07|BC ZSM Lep 02694|658[0n]bp|Australia.Queensland
Theretra oldenlandiae lewini|GWORD054-07|BC ZSM Lep 02686|658[0n]bp|Australia.Queensland
Theretra oldenlandiae lewini|GWORI543-09|BC ZSM Lep 13291|658[0n]bp|Australia.Northern Territory
Theretra oldenlandiae lewini|GWORG562-08|BC ZSM Lep 15002|658[2n]bp|Australia.Queensland
Theretra oldenlandiae lewini|NSWHJ040-10|09-NSWHH-0053|658[0n]bp|Australia.New South Wales
Theretra oldenlandiae lewini|LOQT800-07|gvc6507-1L|658[0n]bp|Australia.Queensland
Theretra oldenlandiae lewini|GWORD068-07|BC ZSM Lep 02700|650[2n]bp|Australia.Queensland
Theretra oldenlandiae lewini|LNSWE129-06|06-NSWE-00129|658[0n]bp|Australia.New South Wales
Theretra oldenlandiae lewini|GWORB2215-08|BC ZSM Lep 11015|640[0n]bp|Australia.Northern Territory
Theretra oldenlandiae lewini|LNSWE006-06|06-NSWE-00006|609[0n]bp|Australia.New South Wales
Theretra oldenlandiae lewini|LNSWE107-06|06-NSWE-00107|593[0n]bp|Australia.New South Wales
Theretra oldenlandiae lewini|LNSWE132-06|06-NSWE-00132|573[0n]bp|Australia.New South Wales
Theretra oldenlandiae lewini|LNSWE118-06|06-NSWE-00118|583[0n]bp|Australia.New South Wales
Theretra oldenlandiae lewini|LOQB591-05|Moth 070.03CL|574[0n]bp|Australia.Queensland
Theretra oldenlandiae lewini|LNSWE011-06|06-NSWE-00011|613[0n]bp|Australia.New South Wales
Theretra oldenlandiae lewini|SOWE243-07|BC-Hax4142|407[0n]bp|Australia
Theretra oldenlandiae lewini|LNSWE121-06|06-NSWE-00121|584[0n]bp|Australia.New South Wales
Theretra oldenlandiae lewini|LNSWE101-06|06-NSWE-00101|599[0n]bp|Australia.New South Wales
Theretra oldenlandiae lewini|LOQT1052-07|gvc6754-1L|658[0n]bp|Australia.Queensland
Theretra oldenlandiae lewini|LNSWE109-06|06-NSWE-00109|593[0n]bp|Australia.New South Wales
Theretra oldenlandiae lewini|LOQ349-04|04HBL004349|658[0n]bp|Australia.Queensland
Theretra oldenlandiae lewini|LNSWE098-06|06-NSWE-00098|658[1n]bp|Australia.New South Wales
Theretra oldenlandiae lewini|GWORD063-07|BC ZSM Lep 02695|609[0n]bp|Australia.Queensland
Theretra oldenlandiae lewini|GWORI544-09|BC ZSM Lep 13292|658[0n]bp|Australia.Northern Territory
Theretra oldenlandiae lewini|GWORH540-09|BC ZSM Lep 10254|658[0n]bp|Australia.Queensland
Theretra oldenlandiae lewini|GWORI495-09|BC ZSM Lep 13243|658[0n]bp|Australia.Northern Territory
Theretra oldenlandiae lewini|LNSWE075-06|06-NSWE-00075|517[1n]bp|Australia.New South Wales
Theretra oldenlandiae lewini|LOQT023-06|2006-LOQT-023|587[0n]bp|Australia.Queensland
Theretra oldenlandiae lewini|GWORD064-07|BC ZSM Lep 02696|609[0n]bp|Australia.Queensland
Theretra oldenlandiae lewini|GWORD065-07|BC ZSM Lep 02697|609[0n]bp|Australia.Queensland
Theretra oldenlandiae lewini|GWORA139-08|BC ZSM Lep 10511|602[0n]bp|Australia.Western Australia
Theretra oldenlandiae lewini|LNSWE089-06|06-NSWE-00089|589[0n]bp|Australia.New South Wales
Theretra oldenlandiae lewini|LNSWE056-06|06-NSWE-00056|606[0n]bp|Australia.New South Wales
Theretra oldenlandiae lewini|LNSWE100-06|06-NSWE-00100|657[0n]bp|Australia.New South Wales
Theretra oldenlandiae lewini|NSWHH054-09|08-NSWHH-0054|658[0n]bp|Australia.New South Wales
Theretra oldenlandiae lewini|LOQTE772-10|gvc14063-1L|658[0n]bp|Australia.Queensland
Theretra oldenlandiae lewini|GWORD058-07|BC ZSM Lep 02690|658[1n]bp|Australia.Queensland
Theretra margarita|GWORB2256-08|BC ZSM Lep 11056|609[0n]bp|Australia.Northern Territory
Theretra margarita|GWORA099-08|BC ZSM Lep 10471|558[1n]bp|Australia.Northern Territory
Theretra margarita|GWORD050-07|BC ZSM Lep 02682|572[0n]bp|Australia.Queensland
Theretra margarita|GWORA186-08|BC ZSM Lep 10558|649[1n]bp|Australia.Western Australia
Theretra margarita|LOQTI518-11|gvc17066-1L|658[0n]bp|Australia.Queensland
Theretra margarita|LOQTB046-07|gvc6812-1L|643[1n]bp|Australia.Queensland
Theretra margarita|LOQTE720-10|gvc13863-1L|658[6n]bp|Australia.Queensland
Theretra margarita|LOQT807-07|gvc6515-1L|658[0n]bp|Australia.Queensland
Theretra margarita|GWORA162-08|BC ZSM Lep 10534|615[0n]bp|Australia.Western Australia
Theretra margarita|LOQTI516-11|gvc17064-1L|658[0n]bp|Australia.Queensland
Theretra margarita|GWORN236-09|BC ZSM Lep 18264|658[0n]bp|Australia.Western Australia
Theretra margarita|GWORA182-08|BC ZSM Lep 10554|649[0n]bp|Australia.Western Australia
Theretra margarita|GWORA187-08|BC ZSM Lep 10559|646[0n]bp|Australia.Western Australia
Theretra margarita|GWORA188-08|BC ZSM Lep 10560|604[4n]bp|Australia.Western Australia
Theretra margarita|LOQTE688-10|gvc13805-1L|641[0n]bp|Australia.Queensland
Theretra margarita|GWORA124-08|BC ZSM Lep 10496|646[0n]bp|Australia.Western Australia
Theretra margarita|GWORN237-09|BC ZSM Lep 18265|658[0n]bp|Australia.Western Australia
Theretra margarita|GWORA013-08|BC ZSM Lep 10385|656[0n]bp|Australia.Western Australia
Theretra margarita|LOQT700-06|gvc6396-1L|658[0n]bp|Australia.Queensland
Theretra margarita|GWORA088-08|BC ZSM Lep 10460|658[0n]bp|Australia.Western Australia
Theretra margarita|GWORD061-07|BC ZSM Lep 02693|658[0n]bp|Australia.Queensland
Theretra margarita|GWORC188-07|BC ZSM Lep 02538|630[0n]bp|Australia.Queensland

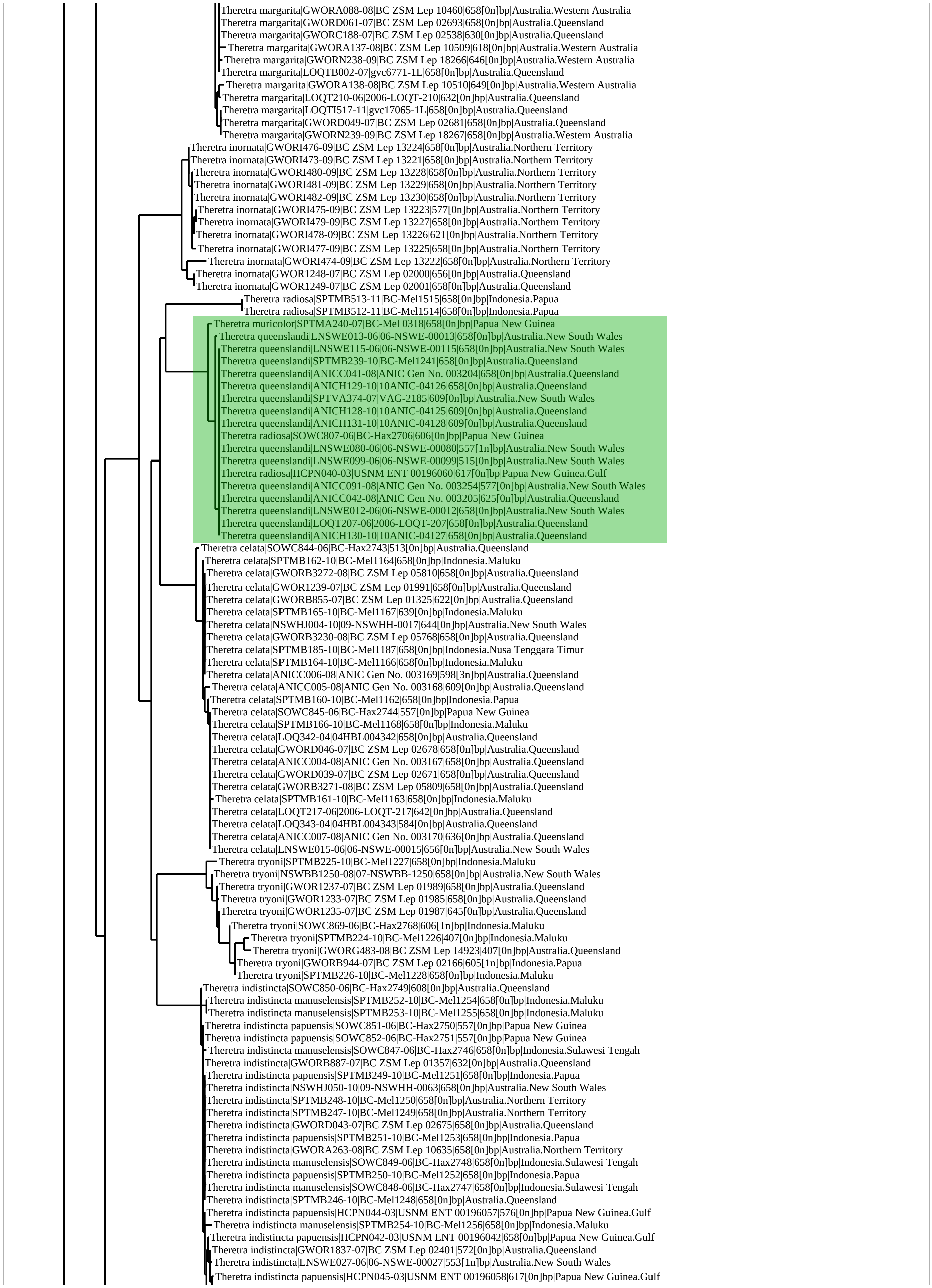
Theretra margarita|GWORA088-08|BC ZSM Lep 10460|658[0n]bp|Australia.Western Australia
Theretra margarita|GWORD061-07|BC ZSM Lep 02693|658[0n]bp|Australia.Queensland
Theretra margarita|GWORC188-07|BC ZSM Lep 02538|630[0n]bp|Australia.Queensland
Theretra margarita|GWORA137-08|BC ZSM Lep 10509|618[0n]bp|Australia.Western Australia
Theretra margarita|GWORN238-09|BC ZSM Lep 18266|646[0n]bp|Australia.Western Australia
Theretra margarita|LOQTB002-07|gvc6771-1L|658[0n]bp|Australia.Queensland
Theretra margarita|GWORA138-08|BC ZSM Lep 10510|649[0n]bp|Australia.Western Australia
Theretra margarita|LOQT210-06|2006-LOQT-210|632[0n]bp|Australia.Queensland
Theretra margarita|LOQTI517-11|gvc17065-1L|658[0n]bp|Australia.Queensland
Theretra margarita|GWORD049-07|BC ZSM Lep 02681|658[0n]bp|Australia.Queensland
Theretra margarita|GWORN239-09|BC ZSM Lep 18267|658[0n]bp|Australia.Western Australia
Theretra inornata|GWORI476-09|BC ZSM Lep 13224|658[0n]bp|Australia.Northern Territory
Theretra inornata|GWORI473-09|BC ZSM Lep 13221|658[0n]bp|Australia.Northern Territory
Theretra inornata|GWORI480-09|BC ZSM Lep 13228|658[0n]bp|Australia.Northern Territory
Theretra inornata|GWORI481-09|BC ZSM Lep 13229|658[0n]bp|Australia.Northern Territory
Theretra inornata|GWORI482-09|BC ZSM Lep 13230|658[0n]bp|Australia.Northern Territory
Theretra inornata|GWORI475-09|BC ZSM Lep 13223|577[0n]bp|Australia.Northern Territory
Theretra inornata|GWORI479-09|BC ZSM Lep 13227|658[0n]bp|Australia.Northern Territory
Theretra inornata|GWORI478-09|BC ZSM Lep 13226|621[0n]bp|Australia.Northern Territory
Theretra inornata|GWORI477-09|BC ZSM Lep 13225|658[0n]bp|Australia.Northern Territory
Theretra inornata|GWORI474-09|BC ZSM Lep 13222|658[0n]bp|Australia.Northern Territory
Theretra inornata|GWOR1248-07|BC ZSM Lep 02000|656[0n]bp|Australia.Queensland
Theretra inornata|GWOR1249-07|BC ZSM Lep 02001|658[0n]bp|Australia.Queensland
Theretra radiosa|SPTMB513-11|BC-Mel1515|658[0n]bp|Indonesia.Papua
Theretra radiosa|SPTMB512-11|BC-Mel1514|658[0n]bp|Indonesia.Papua
Theretra muricolor|SPTMA240-07|BC-Mel 0318|658[0n]bp|Papua New Guinea
Theretra queenslandi|LNSWE013-06|06-NSWE-00013|658[0n]bp|Australia.New South Wales
Theretra queenslandi|LNSWE115-06|06-NSWE-00115|658[0n]bp|Australia.New South Wales
Theretra queenslandi|SPTMB239-10|BC-Mel1241|658[0n]bp|Australia.Queensland
Theretra queenslandi|ANICC041-08|ANIC Gen No. 003204|658[0n]bp|Australia.Queensland
Theretra queenslandi|ANICH129-10|10ANIC-04126|658[0n]bp|Australia.Queensland
Theretra queenslandi|SPTVA374-07|VAG-2185|609[0n]bp|Australia.New South Wales
Theretra queenslandi|ANICH128-10|10ANIC-04125|609[0n]bp|Australia.Queensland
Theretra queenslandi|ANICH131-10|10ANIC-04128|609[0n]bp|Australia.Queensland
Theretra radiosa|SOWC807-06|BC-Hax2706|606[0n]bp|Papua New Guinea
Theretra queenslandi|LNSWE080-06|06-NSWE-00080|557[1n]bp|Australia.New South Wales
Theretra queenslandi|LNSWE099-06|06-NSWE-00099|515[0n]bp|Australia.New South Wales
Theretra radiosa|HCPN040-03|USNM ENT 00196060|617[0n]bp|Papua New Guinea.Gulf
Theretra queenslandi|ANICC091-08|ANIC Gen No. 003254|577[0n]bp|Australia.New South Wales
Theretra queenslandi|ANICC042-08|ANIC Gen No. 003205|625[0n]bp|Australia.Queensland
Theretra queenslandi|LNSWE012-06|06-NSWE-00012|658[0n]bp|Australia.New South Wales
Theretra queenslandi|LOQT207-06|2006-LOQT-207|658[0n]bp|Australia.Queensland
Theretra queenslandi|ANICH130-10|10ANIC-04127|658[0n]bp|Australia.Queensland
Theretra celata|SOWC844-06|BC-Hax2743|513[0n]bp|Australia.Queensland
Theretra celata|SPTMB162-10|BC-Mel1164|658[0n]bp|Indonesia.Maluku
Theretra celata|GWORB3272-08|BC ZSM Lep 05810|658[0n]bp|Australia.Queensland
Theretra celata|GWOR1239-07|BC ZSM Lep 01991|658[0n]bp|Australia.Queensland
Theretra celata|GWORB855-07|BC ZSM Lep 01325|622[0n]bp|Australia.Queensland
Theretra celata|SPTMB165-10|BC-Mel1167|639[0n]bp|Indonesia.Maluku
Theretra celata|NSWHJ004-10|09-NSWHH-0017|644[0n]bp|Australia.New South Wales
Theretra celata|GWORB3230-08|BC ZSM Lep 05768|658[0n]bp|Australia.Queensland
Theretra celata|SPTMB185-10|BC-Mel1187|658[0n]bp|Indonesia.Nusa Tenggara Timur
Theretra celata|SPTMB164-10|BC-Mel1166|658[0n]bp|Indonesia.Maluku
Theretra celata|ANICC006-08|ANIC Gen No. 003169|598[3n]bp|Australia.Queensland
Theretra celata|ANICC005-08|ANIC Gen No. 003168|609[0n]bp|Australia.Queensland
Theretra celata|SPTMB160-10|BC-Mel1162|658[0n]bp|Indonesia.Papua
Theretra celata|SOWC845-06|BC-Hax2744|557[0n]bp|Papua New Guinea
Theretra celata|SPTMB166-10|BC-Mel1168|658[0n]bp|Indonesia.Maluku
Theretra celata|LOQ342-04|04HBL004342|658[0n]bp|Australia.Queensland
Theretra celata|GWORD046-07|BC ZSM Lep 02678|658[0n]bp|Australia.Queensland
Theretra celata|ANICC004-08|ANIC Gen No. 003167|658[0n]bp|Australia.Queensland
Theretra celata|GWORD039-07|BC ZSM Lep 02671|658[0n]bp|Australia.Queensland
Theretra celata|GWORB3271-08|BC ZSM Lep 05809|658[0n]bp|Australia.Queensland
Theretra celata|SPTMB161-10|BC-Mel1163|658[0n]bp|Indonesia.Maluku
Theretra celata|LOQT217-06|2006-LOQT-217|642[0n]bp|Australia.Queensland
Theretra celata|LOQ343-04|04HBL004343|584[0n]bp|Australia.Queensland
Theretra celata|ANICC007-08|ANIC Gen No. 003170|636[0n]bp|Australia.Queensland
Theretra celata|LNSWE015-06|06-NSWE-00015|656[0n]bp|Australia.New South Wales
Theretra tryoni|SPTMB225-10|BC-Mel1227|658[0n]bp|Indonesia.Maluku
Theretra tryoni|NSWBB1250-08|07-NSWBB-1250|658[0n]bp|Australia.New South Wales
Theretra tryoni|GWOR1237-07|BC ZSM Lep 01989|658[0n]bp|Australia.Queensland
Theretra tryoni|GWOR1233-07|BC ZSM Lep 01985|658[0n]bp|Australia.Queensland
Theretra tryoni|GWOR1235-07|BC ZSM Lep 01987|645[0n]bp|Australia.Queensland
Theretra tryoni|SOWC869-06|BC-Hax2768|606[1n]bp|Indonesia.Maluku
Theretra tryoni|SPTMB224-10|BC-Mel1226|407[0n]bp|Indonesia.Maluku
Theretra tryoni|GWORG483-08|BC ZSM Lep 14923|407[0n]bp|Australia.Queensland
Theretra tryoni|GWORB944-07|BC ZSM Lep 02166|605[1n]bp|Indonesia.Papua
Theretra tryoni|SPTMB226-10|BC-Mel1228|658[0n]bp|Indonesia.Maluku
Theretra indistincta|SOWC850-06|BC-Hax2749|608[0n]bp|Australia.Queensland
Theretra indistincta manuselensis|SPTMB252-10|BC-Mel1254|658[0n]bp|Indonesia.Maluku
Theretra indistincta manuselensis|SPTMB253-10|BC-Mel1255|658[0n]bp|Indonesia.Maluku
Theretra indistincta papuensis|SOWC851-06|BC-Hax2750|557[0n]bp|Papua New Guinea
Theretra indistincta papuensis|SOWC852-06|BC-Hax2751|557[0n]bp|Papua New Guinea
Theretra indistincta manuselensis|SOWC847-06|BC-Hax2746|658[0n]bp|Indonesia.Sulawesi Tengah
Theretra indistincta|GWORB887-07|BC ZSM Lep 01357|632[0n]bp|Australia.Queensland
Theretra indistincta papuensis|SPTMB249-10|BC-Mel1251|658[0n]bp|Indonesia.Papua
Theretra indistincta|NSWHJ050-10|09-NSWHH-0063|658[0n]bp|Australia.New South Wales
Theretra indistincta|SPTMB248-10|BC-Mel1250|658[0n]bp|Australia.Northern Territory
Theretra indistincta|SPTMB247-10|BC-Mel1249|658[0n]bp|Australia.Northern Territory
Theretra indistincta|GWORD043-07|BC ZSM Lep 02675|658[0n]bp|Australia.Queensland
Theretra indistincta papuensis|SPTMB251-10|BC-Mel1253|658[0n]bp|Indonesia.Papua
Theretra indistincta|GWORA263-08|BC ZSM Lep 10635|658[0n]bp|Australia.Northern Territory
Theretra indistincta manuselensis|SOWC849-06|BC-Hax2748|658[0n]bp|Indonesia.Sulawesi Tengah
Theretra indistincta papuensis|SPTMB250-10|BC-Mel1252|658[0n]bp|Indonesia.Papua
Theretra indistincta manuselensis|SOWC848-06|BC-Hax2747|658[0n]bp|Indonesia.Sulawesi Tengah
Theretra indistincta|SPTMB246-10|BC-Mel1248|658[0n]bp|Australia.Queensland
Theretra indistincta papuensis|HCPN044-03|USNM ENT 00196057|576[0n]bp|Papua New Guinea.Gulf
Theretra indistincta manuselensis|SPTMB254-10|BC-Mel1256|658[0n]bp|Indonesia.Maluku
Theretra indistincta papuensis|HCPN042-03|USNM ENT 00196042|658[0n]bp|Papua New Guinea.Gulf
Theretra indistincta|GWOR1837-07|BC ZSM Lep 02401|572[0n]bp|Australia.Queensland
Theretra indistincta|LNSWE027-06|06-NSWE-00027|553[1n]bp|Australia.New South Wales
Theretra indistincta papuensis|HCPN045-03|USNM ENT 00196058|617[0n]bp|Papua New Guinea.Gulf

Theretra indistincta|GWOR1837-07|BC ZSM Lep 02401|572[0n]bp|Australia.Queensland
Theretra indistincta|LNSWE027-06|06-NSWE-00027|553[1n]bp|Australia.New South Wales
Theretra indistincta papuensis|HCPN045-03|USNM ENT 00196058|617[0n]bp|Papua New Guinea.Gulf
Theretra indistincta|LOQ344-04|04HBL004344|603[1n]bp|Australia.Queensland
Theretra indistincta|LNSWE014-06|06-NSWE-00014|575[0n]bp|Australia.New South Wales
Theretra latreillii lucasii|SOWC866-06|BC-Hax2765|497[0n]bp|Malaysia
Theretra latreillii lucasii|SOWC867-06|BC-Hax2766|557[0n]bp|Philippines.Mountain
Theretra latreillii prattorum|SPTMC385-12|BC-Mel2386|658[0n]bp|Indonesia.Maluku
Theretra latreillii prattorum|SPTMC383-12|BC-Mel2384|658[0n]bp|Indonesia.Maluku
Theretra latreillii prattorum|SPTMC386-12|BC-Mel2387|658[0n]bp|Indonesia.Maluku
Theretra latreillii lucasii|SPTOL106-07|AYK-04-0111|658[0n]bp|Malaysia.Pahang
Theretra latreillii prattorum|SPTMB227-10|BC-Mel1229|658[0n]bp|Indonesia.Maluku
Theretra latreillii prattorum|SPTMC384-12|BC-Mel2385|658[0n]bp|Indonesia.Maluku
Theretra latreillii prattorum|SPTMB228-10|BC-Mel1230|658[0n]bp|Indonesia.Maluku
Theretra latreillii lucasii|SOWC862-06|BC-Hax2761|606[0n]bp|Indonesia.Nusa Tenggara Barat
Theretra latreillii lucasii|SOWC864-06|BC-Hax2763|606[0n]bp|Indonesia.Nusa Tenggara Barat
Theretra latreillii lucasii|SOWC863-06|BC-Hax2762|606[0n]bp|Indonesia.Nusa Tenggara Barat
Theretra latreillii|SOWC868-06|BC-Hax2767|494[0n]bp|Australia.Queensland
Theretra latreillii|SPTOL158-07|AYK-04-0378-1|658[0n]bp|Papua New Guinea.Chimbu
Theretra latreillii|SPTOL157-07|AYK-04-0377|658[0n]bp|Papua New Guinea.Chimbu
Theretra latreillii|GWORD040-07|BC ZSM Lep 02672|658[0n]bp|Australia.Queensland
Theretra latreillii|GWORB875-07|BC ZSM Lep 01345|627[0n]bp|Australia.Queensland
Theretra latreillii|GWORB857-07|BC ZSM Lep 01327|627[0n]bp|Australia.Queensland
Theretra latreillii|GWORB898-07|BC ZSM Lep 01368|627[0n]bp|Australia.Queensland
Theretra latreillii|GWOR1229-07|BC ZSM Lep 01981|657[0n]bp|Australia.Queensland
Theretra latreillii|GWOR1695-07|BC ZSM Lep 02259|658[0n]bp|Australia.Queensland
Theretra latreillii|GWOR1236-07|BC ZSM Lep 01988|658[0n]bp|Australia.Queensland
Theretra latreillii|NSWHJ064-10|09-NSWHH-0077|658[0n]bp|Australia.New South Wales
Theretra latreillii|LOQT712-06|gvc6424-1L|658[0n]bp|Australia.Queensland
Theretra latreillii|NSWHJ016-10|09-NSWHH-0029|658[0n]bp|Australia.New South Wales
Theretra latreillii|NSWHJ086-10|09-NSWHH-0099|658[0n]bp|Australia.New South Wales
Theretra latreillii|NSWHJ028-10|09-NSWHH-0041|658[0n]bp|Australia.New South Wales
Theretra latreillii|LOQTB139-07|gvc6902-1L|658[0n]bp|Australia.Queensland
Theretra latreillii|NSWHJ074-10|09-NSWHH-0087|658[0n]bp|Australia.New South Wales
Theretra latreillii|LOQTB024-07|gvc6798-1L|658[0n]bp|Australia.Queensland
Theretra latreillii|NSWHJ026-10|09-NSWHH-0039|658[0n]bp|Australia.New South Wales
Theretra latreillii|NSWHJ038-10|09-NSWHH-0051|658[0n]bp|Australia.New South Wales
Theretra latreillii|LOQTD909-09|gvc11071-1L|658[0n]bp|Australia.Queensland
Theretra latreillii|IMLR1085-11|IM08-2609|658[0n]bp|Australia.Queensland
Theretra latreillii|GWORB3269-08|BC ZSM Lep 05807|658[0n]bp|Australia.Queensland
Theretra latreillii|GWORG484-08|BC ZSM Lep 14924|658[0n]bp|Australia.Queensland
Theretra latreillii|LOQTD889-09|gvc11028-1L|658[0n]bp|Australia.Queensland
Theretra latreillii|GWOR1231-07|BC ZSM Lep 01983|658[0n]bp|Australia.Queensland
Theretra latreillii|ANIC347-06|ANIC Gen No. 000539|658[0n]bp|Australia.Queensland
Theretra latreillii|GWORG360-08|BC ZSM Lep 08476|658[0n]bp|Australia.Queensland
Theretra latreillii|LOQTB049-07|gvc6825-1L|658[0n]bp|Australia.Queensland
Theretra latreillii|GWOR1238-07|BC ZSM Lep 01990|658[0n]bp|Australia.Queensland
Theretra latreillii|IMLQ230-07|IM07-0146|658[0n]bp|Australia.Queensland
Theretra latreillii|GWORB3270-08|BC ZSM Lep 05808|658[0n]bp|Australia.Queensland
Theretra latreillii|GWOR1232-07|BC ZSM Lep 01984|658[0n]bp|Australia.Queensland
Theretra latreillii|GWORB3267-08|BC ZSM Lep 05805|658[0n]bp|Australia.Queensland
Theretra latreillii|LOQTC919-08|gvc8566-1L|658[0n]bp|Australia.Queensland
Theretra latreillii|GWORG303-08|BC ZSM Lep 08419|658[0n]bp|Australia.Queensland
Theretra latreillii|GWOR1241-07|BC ZSM Lep 01993|658[0n]bp|Australia.Queensland
Theretra latreillii|GWORB856-07|BC ZSM Lep 01326|614[0n]bp|Australia.Queensland
Theretra latreillii|GWORB891-07|BC ZSM Lep 01361|621[0n]bp|Australia.Queensland
Theretra latreillii|GWORB876-07|BC ZSM Lep 01346|621[0n]bp|Australia.Queensland
Theretra latreillii|GWORB888-07|BC ZSM Lep 01358|621[0n]bp|Australia.Queensland
Theretra latreillii|GWORB860-07|BC ZSM Lep 01330|621[0n]bp|Australia.Queensland
Theretra latreillii|GWORB890-07|BC ZSM Lep 01360|621[0n]bp|Australia.Queensland
Theretra latreillii|GWORB859-07|BC ZSM Lep 01329|632[0n]bp|Australia.Queensland
Theretra latreillii|GWORB861-07|BC ZSM Lep 01331|632[0n]bp|Australia.Queensland
Theretra latreillii|GWORB858-07|BC ZSM Lep 01328|632[0n]bp|Australia.Queensland
Theretra latreillii|GWORC234-07|BC ZSM Lep 02584|653[0n]bp|Australia.Queensland
Theretra latreillii|GWOR1694-07|BC ZSM Lep 02258|644[0n]bp|Australia.Queensland
Theretra latreillii|GWORI507-09|BC ZSM Lep 13255|627[0n]bp|Australia.Northern Territory
Theretra latreillii|GWORA136-08|BC ZSM Lep 10508|646[1n]bp|Australia.Western Australia
Theretra latreillii|LNSWE001-06|06-NSWE-00001|532[0n]bp|Australia.New South Wales
Theretra latreillii|GWOR1838-07|BC ZSM Lep 02402|582[0n]bp|Australia.Queensland
Theretra latreillii|GWORB874-07|BC ZSM Lep 01344|607[0n]bp|Australia.Queensland
Theretra latreillii|NSWHJ062-10|09-NSWHH-0075|622[0n]bp|Australia.New South Wales
Theretra latreillii|LOQB003-05|Moth 003.01LZ|575[0n]bp|Australia.Queensland
Theretra latreillii|LOQB004-05|Moth 004.01LZ|575[0n]bp|Australia.Queensland
Theretra latreillii|GWORC233-07|BC ZSM Lep 02583|622[0n]bp|Australia.Queensland
Theretra latreillii|IMLR890-11|IM06-0552|658[0n]bp|Australia.Queensland
Daphnis dohertyi|ANICC057-08|ANIC Gen No. 003220|270[0n]bp|Australia.Queensland
Daphnis dohertyi|SOWB340-06|BC-Hax1333|658[0n]bp|Papua New Guinea.Morobe
Daphnis dohertyi|SOWB341-06|BC-Hax1334|658[0n]bp|Indonesia.Papua
Daphnis dohertyi|SOWB339-06|BC-Hax1332|605[7n]bp|Papua New Guinea.Morobe
Daphnis hypothous crameri|SPTMB409-10|BC-Mel1411|658[0n]bp|India.Karnataka
Daphnis hypothous crameri|SPTMB408-10|BC-Mel1410|658[0n]bp|India.Karnataka
Daphnis hypothous crameri|SPTMB086-09|BC-Mel 1098|658[0n]bp|India.Karnataka
Daphnis hypothous crameri|SPTMB407-10|BC-Mel1409|639[0n]bp|India.Karnataka
Daphnis hypothous crameri|SPTMB406-10|BC-Mel1408|658[0n]bp|India.Karnataka
Daphnis hypothous crameri|SATWB106-08|BC-Roug1154|658[0n]bp|Vietnam
Daphnis moorei|SPTMA488-07|BC-Mel 0566|658[0n]bp|Solomon Islands.Guadalcanal
Daphnis moorei|GWORA134-08|BC ZSM Lep 10506|649[0n]bp|Australia.Western Australia
Daphnis moorei|GWORI498-09|BC ZSM Lep 13246|658[0n]bp|Australia.Northern Territory
Daphnis moorei|LOQTB197-07|gvc6960-1L|658[0n]bp|Australia.Queensland
Daphnis moorei|GWORB871-07|BC ZSM Lep 01341|632[0n]bp|Australia.Queensland
Daphnis moorei|HCPN003-03|USNM ENT 00678975|639[0n]bp|Papua New Guinea.Madang
Daphnis moorei|GWORB852-07|BC ZSM Lep 01322|598[0n]bp|Australia.Queensland
Daphnis moorei|GWORB977-07|BC ZSM Lep 02199|594[1n]bp|Indonesia.Papua
Daphnis moorei|SOWE232-07|BC-Hax4131|658[0n]bp|Australia
Daphnis moorei|GWORB883-07|BC ZSM Lep 01353|632[0n]bp|Australia.Queensland
Daphnis moorei|GWORB851-07|BC ZSM Lep 01321|632[0n]bp|Australia.Queensland
Daphnis moorei|HCPN002-03|USNM ENT 00678979|590[0n]bp|Papua New Guinea.Madang
Daphnis moorei|HCPN004-03|USNM ENT 00678970|605[6n]bp|Papua New Guinea.Madang
Daphnis moorei|LOQT221-06|2006-LOQT-221|608[0n]bp|Australia.Queensland
Daphnis moorei|GWORB976-07|BC ZSM Lep 02198|573[0n]bp|Indonesia.Papua
Daphnis moorei|SOWE231-07|BC-Hax4130|658[0n]bp|Australia
Daphnis protrudens lecourti|SOWB390-06|BC-Hax1383|631[0n]bp|Indonesia.Sulawesi Selatan
Daphnis protrudens lecourti|SOWB389-06|BC-Hax1382|657[0n]bp|Indonesia.Sulawesi Barat

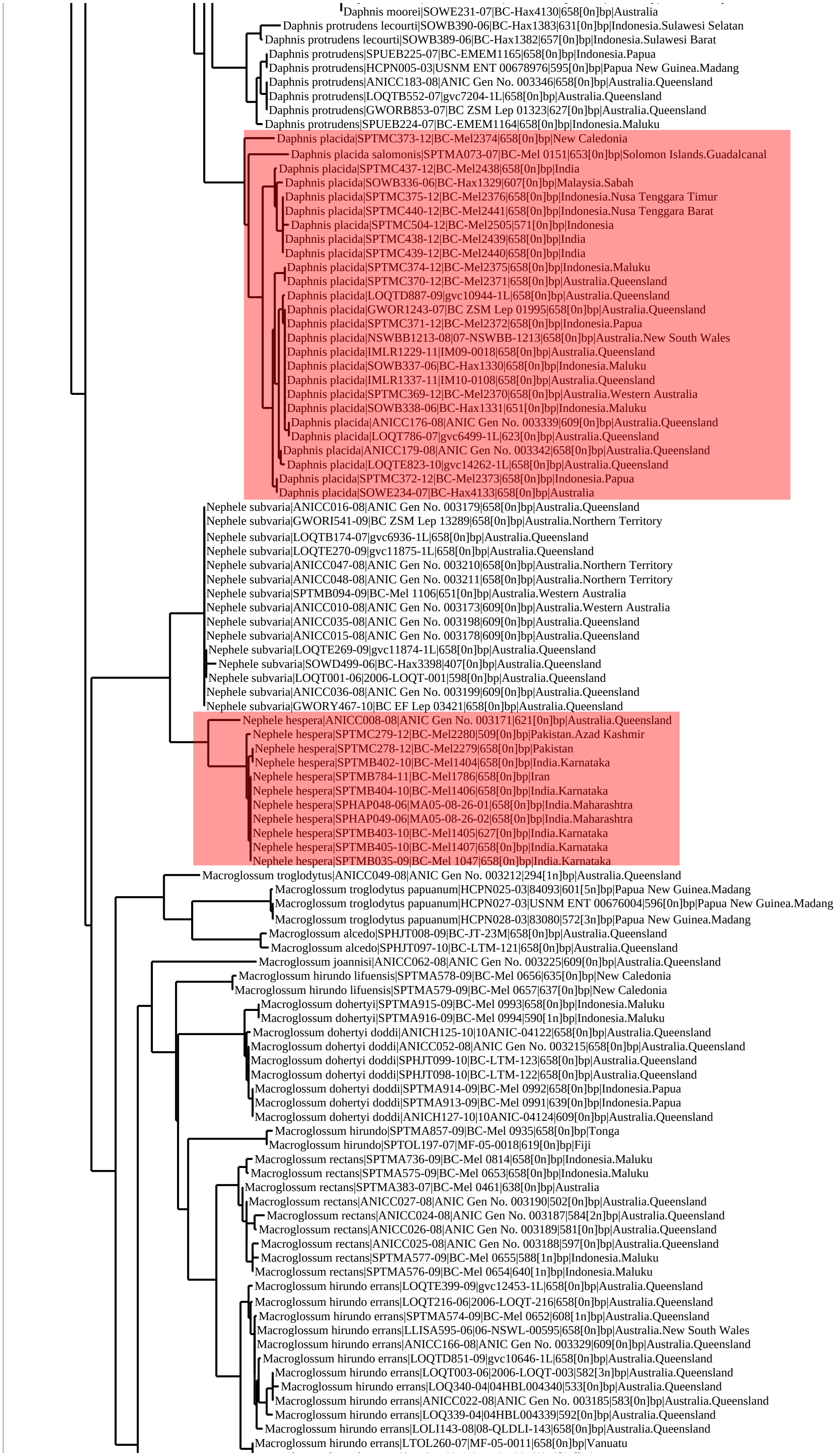
Daphnis moorei|SOWE231-07|BC-Hax4130|658[0n]bp|Australia
Daphnis protrudens lecourti|SOWB390-06|BC-Hax1383|631[0n]bp|Indonesia.Sulawesi Selatan
Daphnis protrudens lecourti|SOWB389-06|BC-Hax1382|657[0n]bp|Indonesia.Sulawesi Barat
Daphnis protrudens|SPUEB225-07|BC-EMEM1165|658[0n]bp|Indonesia.Papua
Daphnis protrudens|HCPN005-03|USNM ENT 00678976|595[0n]bp|Papua New Guinea.Madang
Daphnis protrudens|ANICC183-08|ANIC Gen No. 003346|658[0n]bp|Australia.Queensland
Daphnis protrudens|LOQTB552-07|gvc7204-1L|658[0n]bp|Australia.Queensland
Daphnis protrudens|GWORB853-07|BC ZSM Lep 01323|627[0n]bp|Australia.Queensland
Daphnis protrudens|SPUEB224-07|BC-EMEM1164|658[0n]bp|Indonesia.Maluku
Daphnis placida|SPTMC373-12|BC-Mel2374|658[0n]bp|New Caledonia
Daphnis placida salomonis|SPTMA073-07|BC-Mel 0151|653[0n]bp|Solomon Islands.Guadalcanal
Daphnis placida|SPTMC437-12|BC-Mel2438|658[0n]bp|India
Daphnis placida|SOWB336-06|BC-Hax1329|607[0n]bp|Malaysia.Sabah
Daphnis placida|SPTMC375-12|BC-Mel2376|658[0n]bp|Indonesia.Nusa Tenggara Timur
Daphnis placida|SPTMC440-12|BC-Mel2441|658[0n]bp|Indonesia.Nusa Tenggara Barat
Daphnis placida|SPTMC504-12|BC-Mel2505|571[0n]bp|Indonesia
Daphnis placida|SPTMC438-12|BC-Mel2439|658[0n]bp|India
Daphnis placida|SPTMC439-12|BC-Mel2440|658[0n]bp|India
Daphnis placida|SPTMC374-12|BC-Mel2375|658[0n]bp|Indonesia.Maluku
Daphnis placida|SPTMC370-12|BC-Mel2371|658[0n]bp|Australia.Queensland
Daphnis placida|LOQTD887-09|gvc10944-1L|658[0n]bp|Australia.Queensland
Daphnis placida|GWOR1243-07|BC ZSM Lep 01995|658[0n]bp|Australia.Queensland
Daphnis placida|SPTMC371-12|BC-Mel2372|658[0n]bp|Indonesia.Papua
Daphnis placida|NSWBB1213-08|07-NSWBB-1213|658[0n]bp|Australia.New South Wales
Daphnis placida|IMLR1229-11|IM09-0018|658[0n]bp|Australia.Queensland
Daphnis placida|SOWB337-06|BC-Hax1330|658[0n]bp|Indonesia.Maluku
Daphnis placida|IMLR1337-11|IM10-0108|658[0n]bp|Australia.Queensland
Daphnis placida|SPTMC369-12|BC-Mel2370|658[0n]bp|Australia.Western Australia
Daphnis placida|SOWB338-06|BC-Hax1331|651[0n]bp|Indonesia.Maluku
Daphnis placida|ANICC176-08|ANIC Gen No. 003339|609[0n]bp|Australia.Queensland
Daphnis placida|LOQT786-07|gvc6499-1L|623[0n]bp|Australia.Queensland
Daphnis placida|ANICC179-08|ANIC Gen No. 003342|658[0n]bp|Australia.Queensland
Daphnis placida|LOQTE823-10|gvc14262-1L|658[0n]bp|Australia.Queensland
Daphnis placida|SPTMC372-12|BC-Mel2373|658[0n]bp|Indonesia.Papua
Daphnis placida|SOWE234-07|BC-Hax4133|658[0n]bp|Australia
Nephele subvaria|ANICC016-08|ANIC Gen No. 003179|658[0n]bp|Australia.Queensland
Nephele subvaria|GWORI541-09|BC ZSM Lep 13289|658[0n]bp|Australia.Northern Territory
Nephele subvaria|LOQTB174-07|gvc6936-1L|658[0n]bp|Australia.Queensland
Nephele subvaria|LOQTE270-09|gvc11875-1L|658[0n]bp|Australia.Queensland
Nephele subvaria|ANICC047-08|ANIC Gen No. 003210|658[0n]bp|Australia.Northern Territory
Nephele subvaria|ANICC048-08|ANIC Gen No. 003211|658[0n]bp|Australia.Northern Territory
Nephele subvaria|SPTMB094-09|BC-Mel 1106|651[0n]bp|Australia.Western Australia
Nephele subvaria|ANICC010-08|ANIC Gen No. 003173|609[0n]bp|Australia.Western Australia
Nephele subvaria|ANICC035-08|ANIC Gen No. 003198|609[0n]bp|Australia.Queensland
Nephele subvaria|ANICC015-08|ANIC Gen No. 003178|609[0n]bp|Australia.Queensland
Nephele subvaria|LOQTE269-09|gvc11874-1L|658[0n]bp|Australia.Queensland
Nephele subvaria|SOWD499-06|BC-Hax3398|407[0n]bp|Australia.Queensland
Nephele subvaria|LOQT001-06|2006-LOQT-001|598[0n]bp|Australia.Queensland
Nephele subvaria|ANICC036-08|ANIC Gen No. 003199|609[0n]bp|Australia.Queensland
Nephele subvaria|GWORY467-10|BC EF Lep 03421|658[0n]bp|Australia.Queensland
Nephele hespera|ANICC008-08|ANIC Gen No. 003171|621[0n]bp|Australia.Queensland
Nephele hespera|SPTMC279-12|BC-Mel2280|509[0n]bp|Pakistan.Azad Kashmir
Nephele hespera|SPTMC278-12|BC-Mel2279|658[0n]bp|Pakistan
Nephele hespera|SPTMB402-10|BC-Mel1404|658[0n]bp|India.Karnataka
Nephele hespera|SPTMB784-11|BC-Mel1786|658[0n]bp|Iran
Nephele hespera|SPTMB404-10|BC-Mel1406|658[0n]bp|India.Karnataka
Nephele hespera|SPHAP048-06|MA05-08-26-01|658[0n]bp|India.Maharashtra
Nephele hespera|SPHAP049-06|MA05-08-26-02|658[0n]bp|India.Maharashtra
Nephele hespera|SPTMB403-10|BC-Mel1405|627[0n]bp|India.Karnataka
Nephele hespera|SPTMB405-10|BC-Mel1407|658[0n]bp|India.Karnataka
Nephele hespera|SPTMB035-09|BC-Mel 1047|658[0n]bp|India.Karnataka
Macroglossum troglodytus|ANICC049-08|ANIC Gen No. 003212|294[1n]bp|Australia.Queensland
Macroglossum troglodytus papuanum|HCPN025-03|84093|601[5n]bp|Papua New Guinea.Madang
Macroglossum troglodytus papuanum|HCPN027-03|USNM ENT 00676004|596[0n]bp|Papua New Guinea.Madang
Macroglossum troglodytus papuanum|HCPN028-03|83080|572[3n]bp|Papua New Guinea.Madang
Macroglossum alcedo|SPHJT008-09|BC-JT-23M|658[0n]bp|Australia.Queensland
Macroglossum alcedo|SPHJT097-10|BC-LTM-121|658[0n]bp|Australia.Queensland
Macroglossum joannisi|ANICC062-08|ANIC Gen No. 003225|609[0n]bp|Australia.Queensland
Macroglossum hirundo lifuensis|SPTMA578-09|BC-Mel 0656|635[0n]bp|New Caledonia
Macroglossum hirundo lifuensis|SPTMA579-09|BC-Mel 0657|637[0n]bp|New Caledonia
Macroglossum dohertyi|SPTMA915-09|BC-Mel 0993|658[0n]bp|Indonesia.Maluku
Macroglossum dohertyi|SPTMA916-09|BC-Mel 0994|590[1n]bp|Indonesia.Maluku
Macroglossum dohertyi doddi|ANICH125-10|10ANIC-04122|658[0n]bp|Australia.Queensland
Macroglossum dohertyi doddi|ANICC052-08|ANIC Gen No. 003215|658[0n]bp|Australia.Queensland
Macroglossum dohertyi doddi|SPHJT099-10|BC-LTM-123|658[0n]bp|Australia.Queensland
Macroglossum dohertyi doddi|SPHJT098-10|BC-LTM-122|658[0n]bp|Australia.Queensland
Macroglossum dohertyi doddi|SPTMA914-09|BC-Mel 0992|658[0n]bp|Indonesia.Papua
Macroglossum dohertyi doddi|SPTMA913-09|BC-Mel 0991|639[0n]bp|Indonesia.Papua
Macroglossum dohertyi doddi|ANICH127-10|10ANIC-04124|609[0n]bp|Australia.Queensland
Macroglossum hirundo|SPTMA857-09|BC-Mel 0935|658[0n]bp|Tonga
Macroglossum hirundo|SPTOL197-07|MF-05-0018|619[0n]bp|Fiji
Macroglossum rectans|SPTMA736-09|BC-Mel 0814|658[0n]bp|Indonesia.Maluku
Macroglossum rectans|SPTMA575-09|BC-Mel 0653|658[0n]bp|Indonesia.Maluku
Macroglossum rectans|SPTMA383-07|BC-Mel 0461|638[0n]bp|Australia
Macroglossum rectans|ANICC027-08|ANIC Gen No. 003190|502[0n]bp|Australia.Queensland
Macroglossum rectans|ANICC024-08|ANIC Gen No. 003187|584[2n]bp|Australia.Queensland
Macroglossum rectans|ANICC026-08|ANIC Gen No. 003189|581[0n]bp|Australia.Queensland
Macroglossum rectans|ANICC025-08|ANIC Gen No. 003188|597[0n]bp|Australia.Queensland
Macroglossum rectans|SPTMA577-09|BC-Mel 0655|588[1n]bp|Indonesia.Maluku
Macroglossum rectans|SPTMA576-09|BC-Mel 0654|640[1n]bp|Indonesia.Maluku
Macroglossum hirundo errans|LOQTE399-09|gvc12453-1L|658[0n]bp|Australia.Queensland
Macroglossum hirundo errans|LOQT216-06|2006-LOQT-216|658[0n]bp|Australia.Queensland
Macroglossum hirundo errans|SPTMA574-09|BC-Mel 0652|608[1n]bp|Australia.Queensland
Macroglossum hirundo errans|LLISA595-06|06-NSWL-00595|658[0n]bp|Australia.New South Wales
Macroglossum hirundo errans|ANICC166-08|ANIC Gen No. 003329|609[0n]bp|Australia.Queensland
Macroglossum hirundo errans|LOQTD851-09|gvc10646-1L|658[0n]bp|Australia.Queensland
Macroglossum hirundo errans|LOQT003-06|2006-LOQT-003|582[3n]bp|Australia.Queensland
Macroglossum hirundo errans|LOQ340-04|04HBL004340|533[0n]bp|Australia.Queensland
Macroglossum hirundo errans|ANICC022-08|ANIC Gen No. 003185|583[0n]bp|Australia.Queensland
Macroglossum hirundo errans|LOQ339-04|04HBL004339|592[0n]bp|Australia.Queensland
Macroglossum hirundo errans|LOLI143-08|08-QLDLI-143|658[0n]bp|Australia.Queensland
Macroglossum hirundo errans|LTOL260-07|MF-05-0011|658[0n]bp|Vanuatu

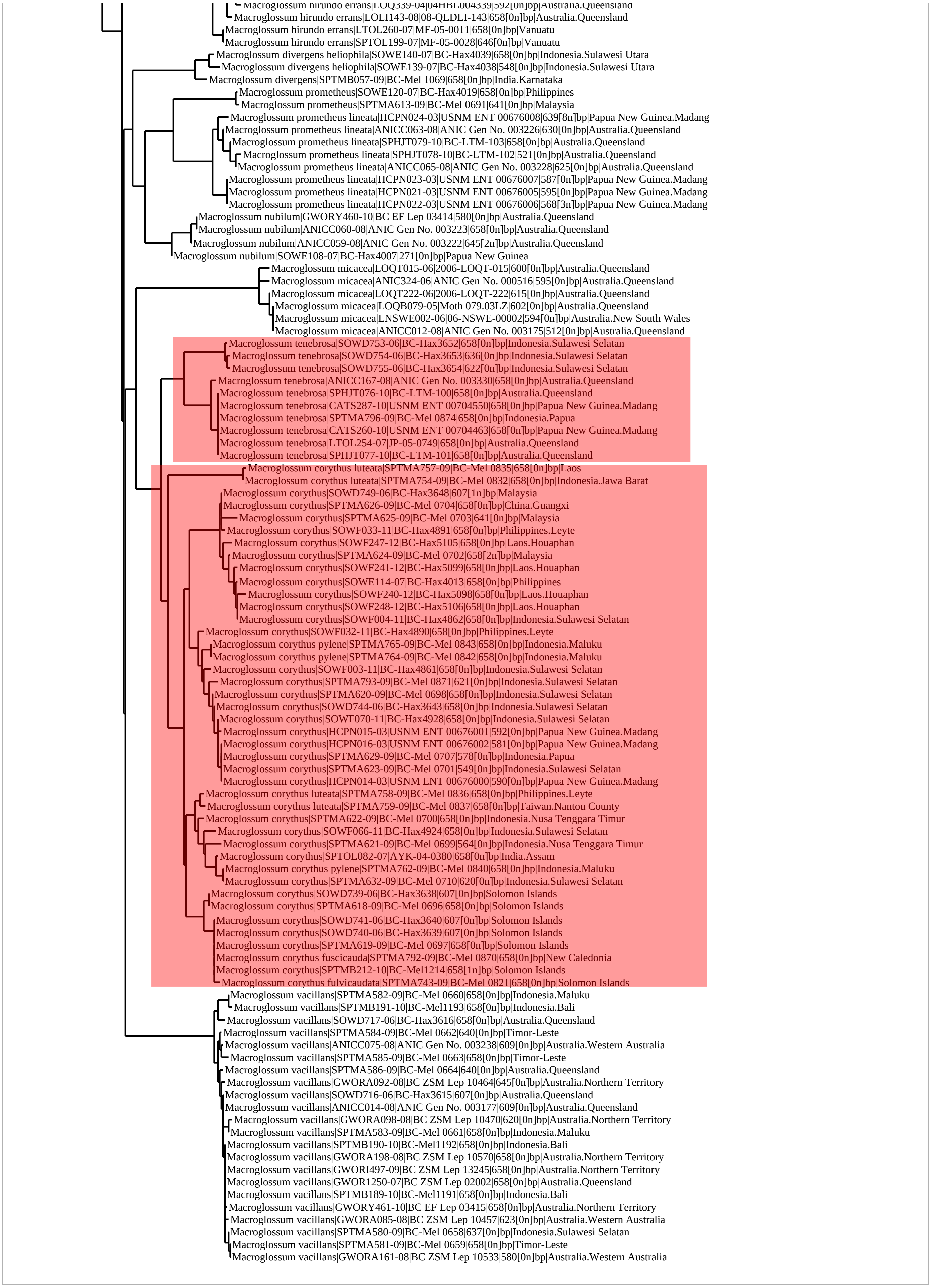
Macroglossum hirundo errans|LOQ339-04|04HBL004339|592[0n]bp|Australia.Queensland
Macroglossum hirundo errans|LOLI143-08|08-QLDLI-143|658[0n]bp|Australia.Queensland
Macroglossum hirundo errans|LTOL260-07|MF-05-0011|658[0n]bp|Vanuatu
Macroglossum hirundo errans|SPTOL199-07|MF-05-0028|646[0n]bp|Vanuatu
Macroglossum divergens heliophila|SOWE140-07|BC-Hax4039|658[0n]bp|Indonesia.Sulawesi Utara
Macroglossum divergens heliophila|SOWE139-07|BC-Hax4038|548[0n]bp|Indonesia.Sulawesi Utara
Macroglossum divergens|SPTMB057-09|BC-Mel 1069|658[0n]bp|India.Karnataka
Macroglossum prometheus|SOWE120-07|BC-Hax4019|658[0n]bp|Philippines
Macroglossum prometheus|SPTMA613-09|BC-Mel 0691|641[0n]bp|Malaysia
Macroglossum prometheus lineata|HCPN024-03|USNM ENT 00676008|639[8n]bp|Papua New Guinea.Madang
Macroglossum prometheus lineata|ANICC063-08|ANIC Gen No. 003226|630[0n]bp|Australia.Queensland
Macroglossum prometheus lineata|SPHJT079-10|BC-LTM-103|658[0n]bp|Australia.Queensland
Macroglossum prometheus lineata|SPHJT078-10|BC-LTM-102|521[0n]bp|Australia.Queensland
Macroglossum prometheus lineata|ANICC065-08|ANIC Gen No. 003228|625[0n]bp|Australia.Queensland
Macroglossum prometheus lineata|HCPN023-03|USNM ENT 00676007|587[0n]bp|Papua New Guinea.Madang
Macroglossum prometheus lineata|HCPN021-03|USNM ENT 00676005|595[0n]bp|Papua New Guinea.Madang
Macroglossum prometheus lineata|HCPN022-03|USNM ENT 00676006|568[3n]bp|Papua New Guinea.Madang
Macroglossum nubilum|GWORY460-10|BC EF Lep 03414|580[0n]bp|Australia.Queensland
Macroglossum nubilum|ANICC060-08|ANIC Gen No. 003223|658[0n]bp|Australia.Queensland
Macroglossum nubilum|ANICC059-08|ANIC Gen No. 003222|645[2n]bp|Australia.Queensland
Macroglossum nubilum|SOWE108-07|BC-Hax4007|271[0n]bp|Papua New Guinea
Macroglossum micacea|LOQT015-06|2006-LOQT-015|600[0n]bp|Australia.Queensland
Macroglossum micacea|ANIC324-06|ANIC Gen No. 000516|595[0n]bp|Australia.Queensland
Macroglossum micacea|LOQT222-06|2006-LOQT-222|615[0n]bp|Australia.Queensland
Macroglossum micacea|LOQB079-05|Moth 079.03LZ|602[0n]bp|Australia.Queensland
Macroglossum micacea|LNSWE002-06|06-NSWE-00002|594[0n]bp|Australia.New South Wales
Macroglossum micacea|ANICC012-08|ANIC Gen No. 003175|512[0n]bp|Australia.Queensland
Macroglossum tenebrosa|SOWD753-06|BC-Hax3652|658[0n]bp|Indonesia.Sulawesi Selatan
Macroglossum tenebrosa|SOWD754-06|BC-Hax3653|636[0n]bp|Indonesia.Sulawesi Selatan
Macroglossum tenebrosa|SOWD755-06|BC-Hax3654|622[0n]bp|Indonesia.Sulawesi Selatan
Macroglossum tenebrosa|ANICC167-08|ANIC Gen No. 003330|658[0n]bp|Australia.Queensland
Macroglossum tenebrosa|SPHJT076-10|BC-LTM-100|658[0n]bp|Australia.Queensland
Macroglossum tenebrosa|CATS287-10|USNM ENT 00704550|658[0n]bp|Papua New Guinea.Madang
Macroglossum tenebrosa|SPTMA796-09|BC-Mel 0874|658[0n]bp|Indonesia.Papua
Macroglossum tenebrosa|CATS260-10|USNM ENT 00704463|658[0n]bp|Papua New Guinea.Madang
Macroglossum tenebrosa|LTOL254-07|JP-05-0749|658[0n]bp|Australia.Queensland
Macroglossum tenebrosa|SPHJT077-10|BC-LTM-101|658[0n]bp|Australia.Queensland
Macroglossum corythus luteata|SPTMA757-09|BC-Mel 0835|658[0n]bp|Laos
Macroglossum corythus luteata|SPTMA754-09|BC-Mel 0832|658[0n]bp|Indonesia.Jawa Barat
Macroglossum corythus|SOWD749-06|BC-Hax3648|607[1n]bp|Malaysia
Macroglossum corythus|SPTMA626-09|BC-Mel 0704|658[0n]bp|China.Guangxi
Macroglossum corythus|SPTMA625-09|BC-Mel 0703|641[0n]bp|Malaysia
Macroglossum corythus|SOWF033-11|BC-Hax4891|658[0n]bp|Philippines.Leyte
Macroglossum corythus|SOWF247-12|BC-Hax5105|658[0n]bp|Laos.Houaphan
Macroglossum corythus|SPTMA624-09|BC-Mel 0702|658[2n]bp|Malaysia
Macroglossum corythus|SOWF241-12|BC-Hax5099|658[0n]bp|Laos.Houaphan
Macroglossum corythus|SOWE114-07|BC-Hax4013|658[0n]bp|Philippines
Macroglossum corythus|SOWF240-12|BC-Hax5098|658[0n]bp|Laos.Houaphan
Macroglossum corythus|SOWF248-12|BC-Hax5106|658[0n]bp|Laos.Houaphan
Macroglossum corythus|SOWF004-11|BC-Hax4862|658[0n]bp|Indonesia.Sulawesi Selatan
Macroglossum corythus|SOWF032-11|BC-Hax4890|658[0n]bp|Philippines.Leyte
Macroglossum corythus pylene|SPTMA765-09|BC-Mel 0843|658[0n]bp|Indonesia.Maluku
Macroglossum corythus pylene|SPTMA764-09|BC-Mel 0842|658[0n]bp|Indonesia.Maluku
Macroglossum corythus|SOWF003-11|BC-Hax4861|658[0n]bp|Indonesia.Sulawesi Selatan
Macroglossum corythus|SPTMA793-09|BC-Mel 0871|621[0n]bp|Indonesia.Sulawesi Selatan
Macroglossum corythus|SPTMA620-09|BC-Mel 0698|658[0n]bp|Indonesia.Sulawesi Selatan
Macroglossum corythus|SOWD744-06|BC-Hax3643|658[0n]bp|Indonesia.Sulawesi Selatan
Macroglossum corythus|SOWF070-11|BC-Hax4928|658[0n]bp|Indonesia.Sulawesi Selatan
Macroglossum corythus|HCPN015-03|USNM ENT 00676001|592[0n]bp|Papua New Guinea.Madang
Macroglossum corythus|HCPN016-03|USNM ENT 00676002|581[0n]bp|Papua New Guinea.Madang
Macroglossum corythus|SPTMA629-09|BC-Mel 0707|578[0n]bp|Indonesia.Papua
Macroglossum corythus|SPTMA623-09|BC-Mel 0701|549[0n]bp|Indonesia.Sulawesi Selatan
Macroglossum corythus|HCPN014-03|USNM ENT 00676000|590[0n]bp|Papua New Guinea.Madang
Macroglossum corythus luteata|SPTMA758-09|BC-Mel 0836|658[0n]bp|Philippines.Leyte
Macroglossum corythus luteata|SPTMA759-09|BC-Mel 0837|658[0n]bp|Taiwan.Nantou County
Macroglossum corythus|SPTMA622-09|BC-Mel 0700|658[0n]bp|Indonesia.Nusa Tenggara Timur
Macroglossum corythus|SOWF066-11|BC-Hax4924|658[0n]bp|Indonesia.Sulawesi Selatan
Macroglossum corythus|SPTMA621-09|BC-Mel 0699|564[0n]bp|Indonesia.Nusa Tenggara Timur
Macroglossum corythus|SPTOL082-07|AYK-04-0380|658[0n]bp|India.Assam
Macroglossum corythus pylene|SPTMA762-09|BC-Mel 0840|658[0n]bp|Indonesia.Maluku
Macroglossum corythus|SPTMA632-09|BC-Mel 0710|620[0n]bp|Indonesia.Sulawesi Selatan
Macroglossum corythus|SOWD739-06|BC-Hax3638|607[0n]bp|Solomon Islands
Macroglossum corythus|SPTMA618-09|BC-Mel 0696|658[0n]bp|Solomon Islands
Macroglossum corythus|SOWD741-06|BC-Hax3640|607[0n]bp|Solomon Islands
Macroglossum corythus|SOWD740-06|BC-Hax3639|607[0n]bp|Solomon Islands
Macroglossum corythus|SPTMA619-09|BC-Mel 0697|658[0n]bp|Solomon Islands
Macroglossum corythus fuscicauda|SPTMA792-09|BC-Mel 0870|658[0n]bp|New Caledonia
Macroglossum corythus|SPTMB212-10|BC-Mel1214|658[1n]bp|Solomon Islands
Macroglossum corythus fulvicaudata|SPTMA743-09|BC-Mel 0821|658[0n]bp|Solomon Islands
Macroglossum vacillans|SPTMA582-09|BC-Mel 0660|658[0n]bp|Indonesia.Maluku
Macroglossum vacillans|SPTMB191-10|BC-Mel1193|658[0n]bp|Indonesia.Bali
Macroglossum vacillans|SOWD717-06|BC-Hax3616|658[0n]bp|Australia.Queensland
Macroglossum vacillans|SPTMA584-09|BC-Mel 0662|640[0n]bp|Timor-Leste
Macroglossum vacillans|ANICC075-08|ANIC Gen No. 003238|609[0n]bp|Australia.Western Australia
Macroglossum vacillans|SPTMA585-09|BC-Mel 0663|658[0n]bp|Timor-Leste
Macroglossum vacillans|SPTMA586-09|BC-Mel 0664|640[0n]bp|Australia.Queensland
Macroglossum vacillans|GWORA092-08|BC ZSM Lep 10464|645[0n]bp|Australia.Northern Territory
Macroglossum vacillans|SOWD716-06|BC-Hax3615|607[0n]bp|Australia.Queensland
Macroglossum vacillans|ANICC014-08|ANIC Gen No. 003177|609[0n]bp|Australia.Queensland
Macroglossum vacillans|GWORA098-08|BC ZSM Lep 10470|620[0n]bp|Australia.Northern Territory
Macroglossum vacillans|SPTMA583-09|BC-Mel 0661|658[0n]bp|Indonesia.Maluku
Macroglossum vacillans|SPTMB190-10|BC-Mel1192|658[0n]bp|Indonesia.Bali
Macroglossum vacillans|GWORA198-08|BC ZSM Lep 10570|658[0n]bp|Australia.Northern Territory
Macroglossum vacillans|GWORI497-09|BC ZSM Lep 13245|658[0n]bp|Australia.Northern Territory
Macroglossum vacillans|GWOR1250-07|BC ZSM Lep 02002|658[0n]bp|Australia.Queensland
Macroglossum vacillans|SPTMB189-10|BC-Mel1191|658[0n]bp|Indonesia.Bali
Macroglossum vacillans|GWORY461-10|BC EF Lep 03415|658[0n]bp|Australia.Northern Territory
Macroglossum vacillans|GWORA085-08|BC ZSM Lep 10457|623[0n]bp|Australia.Western Australia
Macroglossum vacillans|SPTMA580-09|BC-Mel 0658|637[0n]bp|Indonesia.Sulawesi Selatan
Macroglossum vacillans|SPTMA581-09|BC-Mel 0659|658[0n]bp|Timor-Leste
Macroglossum vacillans|GWORA161-08|BC ZSM Lep 10533|580[0n]bp|Australia.Western Australia
