# Supplementary material for: Australian Sphingidae – DNA Barcodes Challenge Current Species Boundaries and Distributions
Source: PLoS One. 2014 Jul 2;9(7):e101108. doi: 10.1371/journal.pone.0101108 (PMC4079597; doi:10.1371/journal.pone.0101108)

Fig S6. Geographical structure of DNA barcode variation in the Convolvulus Hawkmoth. Geographical distribution of the two DNA barcode clusters in *Agrius convolvuli* (Neighbour Joining tree based on K2P-distances). A congruent split was found after analysing sequences of the nuclear marker 28S rDNA, suggesting that this broadly distributed emerging model-species is in fact two distinct species.

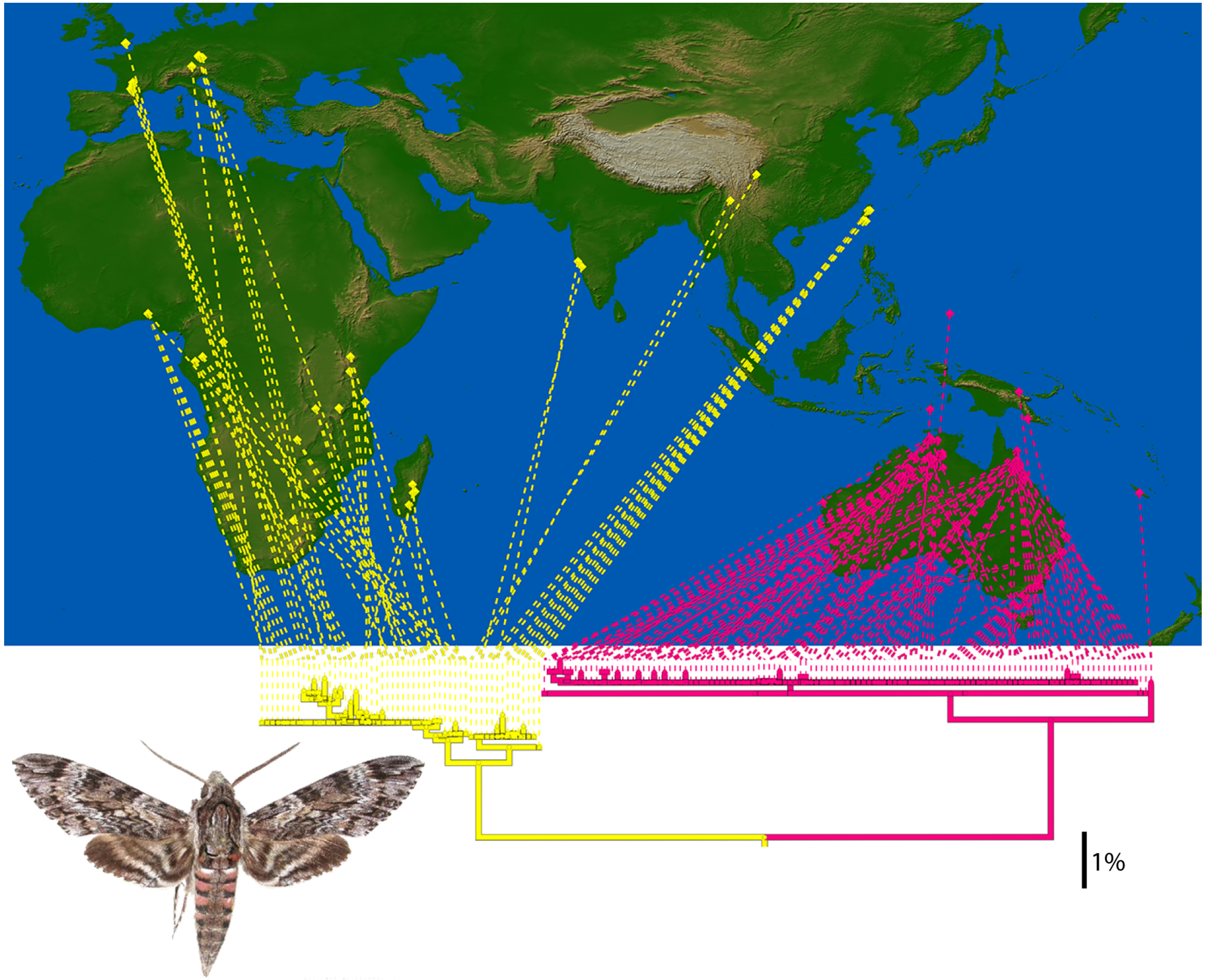

Supplement: Figure S6 — Geographical structure of genetic variation in the Convolvulus Hawkmoth. (PDF) [file pone.0101108.s006.pdf]
