# Supplementary material for: Australian Sphingidae – DNA Barcodes Challenge Current Species Boundaries and Distributions
Source: PLoS One. 2014 Jul 2;9(7):e101108. doi: 10.1371/journal.pone.0101108 (PMC4079597; doi:10.1371/journal.pone.0101108)
Supplement: Figure S7 — Consensus tree (MP) for records within the Theretra oldenlandiae/insignis complex. (PDF) [file pone.0101108.s007.pdf]

Figure S7: Maximum Parsimony tree (strict consensus of 3 trees, CI=0.78, RI=0.82) and geographical distribution for 98 DNA barcode records in the *Theretra oldenlandiae / Theretra insignis* complex. While subspecies *fuscata* (blue) of *T. oldenlandiae* and *kuhnei* (red) of *T. insignis* are both well differentiated genetically from all other taxa, subspecies *lewini* (yellow) is barely differentiated from the nominal subspecies of *T. oldenlandiae* (gray/white). Surprisingly, specimens of *T. insignis* are not genetically different either from *T. oldenlandiae lewini*, despite a very distinctive habitus. The genetic divergence between the latter and *T. insignis kuhnei* suggest however that we may be facing a case of current or past genetic introgression with specimens of *T. insignis* being of hybrid origin.

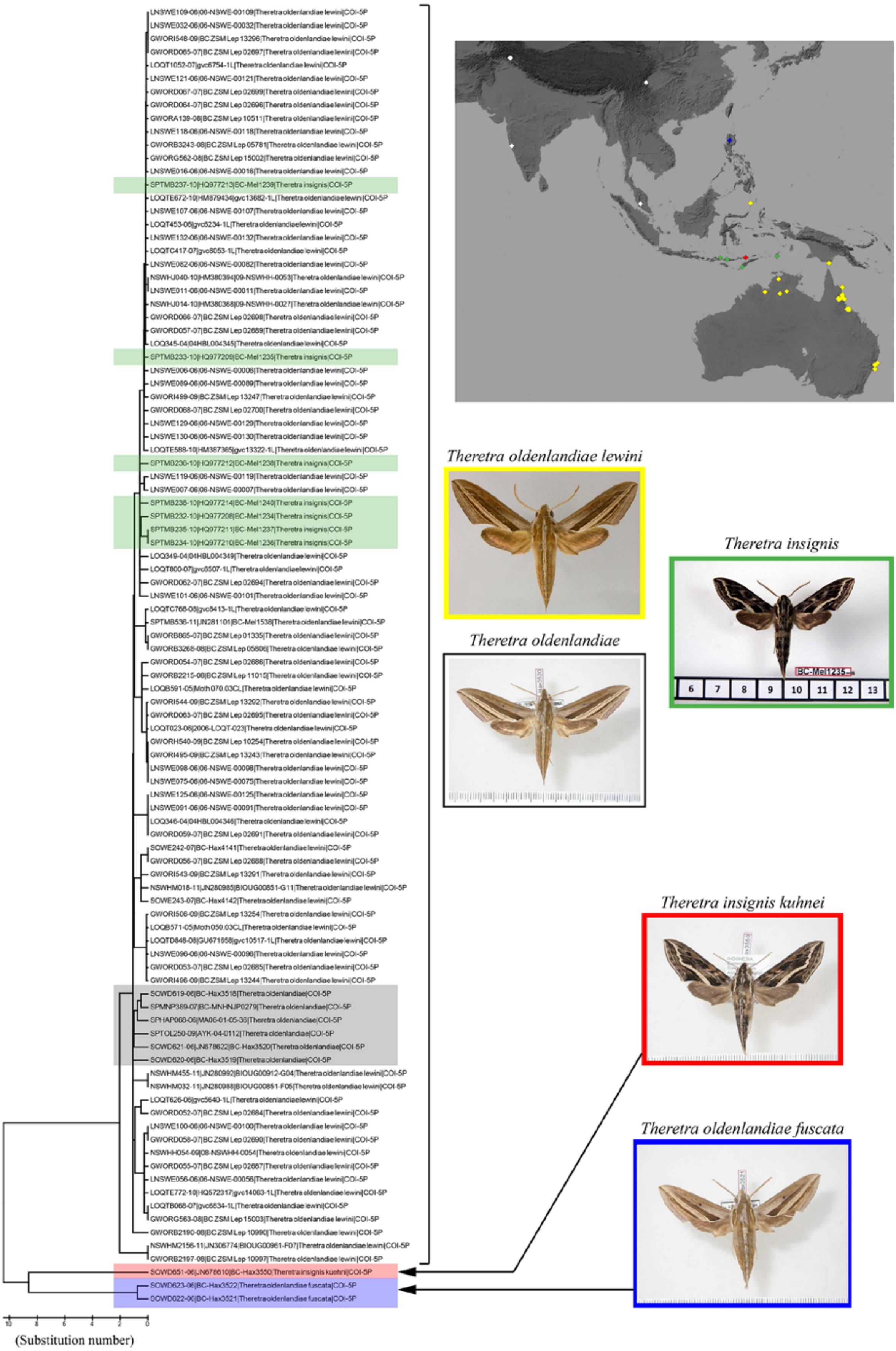
Theretra oldenlandiae lewini
Theretra insignis
Theretra oldenlandiae
Theretra insignis kuhnei
Theretra oldenlandiae fuscata
(Substitution number)
