# Supplementary material for: Australian Sphingidae – DNA Barcodes Challenge Current Species Boundaries and Distributions
Source: PLoS One. 2014 Jul 2;9(7):e101108. doi: 10.1371/journal.pone.0101108 (PMC4079597; doi:10.1371/journal.pone.0101108)
Supplement: Table S1 — List of Australian records. (PDF) [file pone.0101108.s008.pdf]

Table S1: List of Australian records included in the analysis, ordered by *Genus + species* name. GB Acc. = GenBank accession numbers.

Process ID	Sample ID	COI-5P	GB Acc. COI	28S-D2	GB Acc. 28S	Institution Storing	Species	Types	Origin
NSWHM007-11	BIOUG00851-F12	658[0n]	JN280981	-		Biodiversity Institute of Ontario	*Acosmeryx anceus*		New South Wales
NSWBB1231-08	07-NSWBB-1231	658[0n]	KJ168801	-		Biodiversity Institute of Ontario	*Acosmeryx anceus*		New South Wales
GWORB879-07	BC ZSM Lep 01349	557[0n]	KJ168965	-		Bavarian State Collection of Zoology	*Acosmeryx anceus*		Queensland
GWORB862-07	BC ZSM Lep 01332	632[0n]	KJ169142	-		Bavarian State Collection of Zoology	*Acosmeryx anceus*		Queensland
ANIC250-06	ANIC Gen No. 000442	658[0n]	KJ168796	602[0n]	KJ169410	Australian National Insect Collection	*Acosmeryx anceus*		Queensland
GWORC239-07	BC ZSM Lep 02589	658[0n]	KJ169359	-		Bavarian State Collection of Zoology	*Acosmeryx anceus*		Queensland
GWORB3273-08	BC ZSM Lep 05811	658[0n]	KJ169097	-		Bavarian State Collection of Zoology	*Acosmeryx anceus*		Queensland
LOQT224-06	2006-LOQT-224	622[0n]	KJ168970	-		Biodiversity Institute of Ontario	*Acosmeryx anceus*		Queensland
NSWHH007-09	08-NSWHH-0007	658[0n]	KJ169026	-		Biodiversity Institute of Ontario	*Acosmeryx anceus*		New South Wales
LOLI144-08	08-QLDLI-144	649[0n]	KJ168736	-		Biodiversity Institute of Ontario	*Acosmeryx anceus*		Queensland
GWORC238-07	BC ZSM Lep 02588	616[0n]	KJ169093	-		Bavarian State Collection of Zoology	*Acosmeryx anceus*		Queensland
GWORG299-08	BC ZSM Lep 08415	658[0n]	KJ168886	-		Bavarian State Collection of Zoology	*Acosmeryx anceus*		Queensland
LNSWE122-06	06-NSWE-00122	597[0n]	KJ168772	-		Biodiversity Institute of Ontario	*Acosmeryx anceus*		New South Wales
LOQB332-05	Moth 016.03CC	581[0n]	KJ169383	-		Biodiversity Institute of Ontario	*Acosmeryx anceus*		Queensland
NSWHM2026-11	BIOUG00961-A09	658[0n]	JN280990	-		Biodiversity Institute of Ontario	*Acosmeryx anceus*		New South Wales
SPHJT125-10	BC-LTM-149	658[0n]	HQ580427	-		Research Collection of David A. Lane	*Acosmeryx anceus*		Queensland
NSWHM006-11	BIOUG00851-F11	658[0n]	JN280980	-		Biodiversity Institute of Ontario	*Acosmeryx anceus*		New South Wales
LOQB329-05	Moth 013.03CC	593[0n]	KJ168997	-		Biodiversity Institute of Ontario	*Acosmeryx anceus*		Queensland
ANIC238-06	ANIC Gen No. 000430	658[0n]	KJ169358	602[0n]	KJ169424	Australian National Insect Collection	*Acosmeryx anceus*		Queensland
GWOR1826-07	BC ZSM Lep 02390	596[0n]	KJ168961	-		Bavarian State Collection of Zoology	*Acosmeryx anceus*		Queensland
GWOR1880-07	BC ZSM Lep 02444	591[2n]	KJ168815	-		Bavarian State Collection of Zoology	*Acosmeryx anceus*		Queensland
NSWBB1271-08	07-NSWBB-1271	658[0n]	KJ168671	-		Biodiversity Institute of Ontario	*Acosmeryx anceus*		New South Wales
GWORG302-08	BC ZSM Lep 08418	658[0n]	KJ168877	-		Bavarian State Collection of Zoology	*Acosmeryx anceus*		Queensland
GWORB878-07	BC ZSM Lep 01348	596[0n]	KJ168927	-		Bavarian State Collection of Zoology	*Acosmeryx anceus*		Queensland
LOQB570-05	Moth 049.03CL	605[0n]	KJ169015	-		Biodiversity Institute of Ontario	*Acosmeryx anceus*		Queensland
GWORB895-07	BC ZSM Lep 01365	632[0n]	KJ168943	-		Bavarian State Collection of Zoology	*Acosmeryx anceus*		Queensland
NSWHH003-09	08-NSWHH-0003	658[0n]	KJ169018	-		Biodiversity Institute of Ontario	*Acosmeryx anceus*		New South Wales
GWORB877-07	BC ZSM Lep 01347	631[0n]	KJ169073	-		Bavarian State Collection of Zoology	*Acosmeryx anceus*		Queensland
GWORB863-07	BC ZSM Lep 01333	631[0n]	KJ168953	-		Bavarian State Collection of Zoology	*Acosmeryx anceus*		Queensland
GWORB897-07	BC ZSM Lep 01367	630[0n]	KJ169178	-		Bavarian State Collection of Zoology	*Acosmeryx anceus*		Queensland
NSWHH019-09	08-NSWHH-0019	658[0n]	KJ168986	-		Biodiversity Institute of Ontario	*Acosmeryx anceus*		New South Wales
GWORG301-08	BC ZSM Lep 08417	658[0n]	KJ169281	-		Bavarian State Collection of Zoology	*Acosmeryx anceus*		Queensland
NSWHM005-11	BIOUG00851-F10	658[0n]	JN280979	-		Biodiversity Institute of Ontario	*Acosmeryx anceus*		New South Wales

Process ID	Sample ID	COI-5P	GB Acc. COI	28S-D2	GB Acc. 28S	Institution Storing	Species	Types	Origin
GWORC282-07	BC ZSM Lep 02632	647[0n]	KJ168950	-		Bavarian State Collection of Zoology	*Acosmeryx anceus*		Queensland
LOQB521-05	Moth 205.03CC	557[0n]	KJ169075	-		Biodiversity Institute of Ontario	*Acosmeryx anceus*		Queensland
NSWHM021-11	BIOUG00851-H02	635[0n]	JN280986	-		Biodiversity Institute of Ontario	*Acosmeryx anceus*		New South Wales
SPRBA755-09	BC-RBP-1798	658[0n]	GU704157	-		Research Collection of Ron Brechlin	*Acosmeryx anceus*		Queensland
SOWD453-06	BC-Hax3352	658[0n]	HM384128	-		Research Collection of Jean Haxaire	*Acosmeryx anceus*		
GWORB894-07	BC ZSM Lep 01364	617[0n]	KJ168763	-		Bavarian State Collection of Zoology	*Acosmeryx anceus*		Queensland
GWORC281-07	BC ZSM Lep 02631	653[0n]	KJ168977	-		Bavarian State Collection of Zoology	*Acosmeryx anceus*		Queensland
GWORC240-07	BC ZSM Lep 02590	646[0n]	KJ169077	-		Bavarian State Collection of Zoology	*Acosmeryx anceus*		Queensland
NSWBB1249-08	07-NSWBB-1249	658[0n]	KJ169229	-		Biodiversity Institute of Ontario	*Acosmeryx anceus*		New South Wales
GWORB896-07	BC ZSM Lep 01366	632[0n]	KJ169039	-		Bavarian State Collection of Zoology	*Acosmeryx anceus*		Queensland
NSWHM026-11	BIOUG00851-H07	658[0n]	JN280987	-		Biodiversity Institute of Ontario	*Acosmeryx anceus*		New South Wales
SOWE245-07	BC-Hax4144	658[0n]	JN677651	602[0n]	KJ169417	Research Collection of Jean Haxaire	*Acosmeryx anceus*		
GWORB864-07	BC ZSM Lep 01334	658[0n]	KJ168797	-		Bavarian State Collection of Zoology	*Acosmeryx anceus*		Queensland
LOQTD809-08	gvc10271-1L	658[0n]	GU671694	-		Research Collection of Graeme V. Cocks	*Acosmeryx anceus*		Queensland
NSWHM2140-11	BIOUG00961-E03	658[0n]	JN306757	-		Biodiversity Institute of Ontario	*Acosmeryx miskini*		New South Wales
IMLQ207-07	IM07-0060	658[0n]	KJ168880	-		Biodiversity Institute of Ontario	*Acosmeryx miskini*		Queensland
SPUEA045-07	BC-EMEM0045	658[0n]	KJ169027	-		Entomologisches Museum Eitschberger	*Acosmeryx miskini*		Queensland
NSWBB1204-08	07-NSWBB-1204	658[0n]	KJ169132	-		Biodiversity Institute of Ontario	*Acosmeryx miskini*		New South Wales
LLISA599-06	06-NSWL-00599	658[0n]	KJ169372	-		Biodiversity Institute of Ontario	*Acosmeryx miskini*		New South Wales
IMLQ907-08	IM08-0259	658[0n]	KJ168940	-		Biodiversity Institute of Ontario	*Acosmeryx miskini*		Queensland
ANIC344-06	ANIC Gen No. 000536	658[0n]	KJ169005	-		Australian National Insect Collection	*Acosmeryx miskini*		Queensland
NSWHM2169-11	BIOUG00961-G08	657[1n]	JN306786	-		Biodiversity Institute of Ontario	*Acosmeryx miskini*		New South Wales
LNSWE008-06	06-NSWE-00008	658[0n]	KJ169327	-		Biodiversity Institute of Ontario	*Acosmeryx miskini*		New South Wales
SPTVA213-07	VAG-2024	658[0n]	KJ169096	-		Research Collection of T. Vaglia	*Acosmeryx miskini*		New South Wales
IMLQ203-07	IM07-0046	658[0n]	JN677654	-		Biodiversity Institute of Ontario	*Acosmeryx miskini*		Queensland
SPTVA212-07	VAG-2023	658[0n]	KJ168893	-		Research Collection of T. Vaglia	*Acosmeryx miskini*		New South Wales
LLISA220-06	06-NSWL-00220	658[0n]	KJ169215	-		Biodiversity Institute of Ontario	*Acosmeryx miskini*		New South Wales
GWORC185-07	BC ZSM Lep 02535	658[0n]	KJ168915	-		Bavarian State Collection of Zoology	*Agrius convolvuli*		Queensland
LOQTE680-10	gvc13697-1L	658[0n]	HM879438	-		Biodiversity Institute of Ontario	*Agrius convolvuli*		Queensland
LNSWE009-06	06-NSWE-00009	600[0n]	KJ168876	-		Biodiversity Institute of Ontario	*Agrius convolvuli*		New South Wales
GWORH539-09	BC ZSM Lep 10253	658[0n]	KJ168992	-		Bavarian State Collection of Zoology	*Agrius convolvuli*		Queensland
NSWBB1144-08	07-NSWBB-1144	657[0n]	KJ169321	-		Biodiversity Institute of Ontario	*Agrius convolvuli*		New South Wales
GWORA144-08	BC ZSM Lep 10516	618[0n]	KJ168951	-		Bavarian State Collection of Zoology	*Agrius convolvuli*		Northern Territory
LSM875-11	K287774	628[0n]	KJ168996	-		Australian Museum, Sydney	*Agrius convolvuli*		New South Wales
LOQT806-07	gvc6516-1L	656[0n]	KJ168844	-		Biodiversity Institute of Ontario	*Agrius convolvuli*		Queensland
ANICB366-06	ANIC Gen No. 000167	658[0n]	KJ168873	-		Australian National Insect Collection	*Agrius convolvuli*		Queensland

Process ID	Sample ID	COI-5P	GB Acc. COI	28S-D2	GB Acc. 28S	Institution Storing	Species	Types	Origin
GWORB892-07	BC ZSM Lep 01362	632[0n]	KJ169342	-		Bavarian State Collection of Zoology	*Agrius convolvuli*		Queensland
GWORA033-08	BC ZSM Lep 10405	657[0n]	KJ168930	-		Bavarian State Collection of Zoology	*Agrius convolvuli*		Western Australia
LOQB313-05	Moth 313.01LZ	570[0n]	KJ168670	-		Biodiversity Institute of Ontario	*Agrius convolvuli*		Queensland
GWORB2191-08	BC ZSM Lep 10991	656[0n]	KJ169348	-		Bavarian State Collection of Zoology	*Agrius convolvuli*		Northern Territory
GWORA143-08	BC ZSM Lep 10515	551[1n]	KJ169207	-		Bavarian State Collection of Zoology	*Agrius convolvuli*		Northern Territory
GWORG300-08	BC ZSM Lep 08416	658[0n]	KJ168942	-		Bavarian State Collection of Zoology	*Agrius convolvuli*		Queensland
GWORB2193-08	BC ZSM Lep 10993	632[0n]	KJ169004	-		Bavarian State Collection of Zoology	*Agrius convolvuli*		Northern Territory
NSWHM010-11	BIOUG00851-G03	658[0n]	JN284549	-		Biodiversity Institute of Ontario	*Agrius convolvuli*		New South Wales
GWORG361-08	BC ZSM Lep 08477	658[0n]	KJ169328	-		Bavarian State Collection of Zoology	*Agrius convolvuli*		Queensland
NSWHJ960-10	09-NSWHH-0973	658[0n]	HM381045	-		Biodiversity Institute of Ontario	*Agrius convolvuli*		New South Wales
GWOR1247-07	BC ZSM Lep 01999	658[0n]	KJ168669	-		Bavarian State Collection of Zoology	*Agrius convolvuli*		Queensland
ANICB1117-07	ANIC Gen No. 003135	647[0n]	KJ168660	-		Australian National Insect Collection	*Agrius convolvuli*		South Australia
IMLQ052-07	IM06-0212	658[0n]	KJ169218	-		Biodiversity Institute of Ontario	*Agrius convolvuli*		Queensland
LOQB075-05	Moth 075.03LZ	658[0n]	KJ169257	-		Biodiversity Institute of Ontario	*Agrius convolvuli*		Queensland
NSWBB1227-08	07-NSWBB-1227	658[0n]	KJ169063	-		Biodiversity Institute of Ontario	*Agrius convolvuli*		New South Wales
GWORB2094-08	BC ZSM Lep 10894	657[1n]	KJ169326	-		Bavarian State Collection of Zoology	*Agrius convolvuli*		Northern Territory
SPUEB033-07	BC-EMEM0973	658[0n]	KJ168708	-		Entomologisches Museum Eitschberger	*Agrius convolvuli*		Western Australia
GWORB2188-08	BC ZSM Lep 10988	655[0n]	KJ168956	-		Bavarian State Collection of Zoology	*Agrius convolvuli*		Northern Territory
NSWHJ954-10	09-NSWHH-0967	658[0n]	HM381039	-		Biodiversity Institute of Ontario	*Agrius convolvuli*		New South Wales
GWORB2200-08	BC ZSM Lep 11000	657[0n]	KJ168821	-		Bavarian State Collection of Zoology	*Agrius convolvuli*		Northern Territory
GWORD042-07	BC ZSM Lep 02674	658[0n]	KJ168755	-		Bavarian State Collection of Zoology	*Agrius convolvuli*		Queensland
LOQB568-05	Moth 047.03CL	528[0n]	KJ169336	-		Biodiversity Institute of Ontario	*Agrius convolvuli*		Queensland
GWORA142-08	BC ZSM Lep 10514	563[0n]	KJ169044	-		Bavarian State Collection of Zoology	*Agrius convolvuli*		Northern Territory
GWORB2093-08	BC ZSM Lep 10893	624[0n]	KJ169367	-		Bavarian State Collection of Zoology	*Agrius convolvuli*		Northern Territory
GWORI527-09	BC ZSM Lep 13275	658[0n]	KJ169365	-		Bavarian State Collection of Zoology	*Agrius convolvuli*		Northern Territory
GWORC180-07	BC ZSM Lep 02530	631[0n]	KJ168910	-		Bavarian State Collection of Zoology	*Agrius convolvuli*		Queensland
GWORA280-08	BC ZSM Lep 10652	623[6n]	KJ169168	-		Bavarian State Collection of Zoology	*Agrius convolvuli*		Northern Territory
GWORA262-08	BC ZSM Lep 10634	658[0n]	KJ168700	-		Bavarian State Collection of Zoology	*Agrius convolvuli*		Northern Territory
GWORC182-07	BC ZSM Lep 02532	632[0n]	KJ169329	-		Bavarian State Collection of Zoology	*Agrius convolvuli*		Queensland
LOQTC284-07	gvc7920-1L	659[0n]	KJ168617	-		Biodiversity Institute of Ontario	*Agrius convolvuli*		Queensland
NSWHM019-11	BIOUG00851-G12	658[0n]	JN284550	-		Biodiversity Institute of Ontario	*Agrius convolvuli*		New South Wales
GWORA274-08	BC ZSM Lep 10646	639[0n]	KJ169199	-		Bavarian State Collection of Zoology	*Agrius convolvuli*		Northern Territory
GWORA025-08	BC ZSM Lep 10397	655[0n]	KJ169252	-		Bavarian State Collection of Zoology	*Agrius convolvuli*		Western Australia
GWOR1836-07	BC ZSM Lep 02400	586[0n]	KJ169165	-		Bavarian State Collection of Zoology	*Agrius convolvuli*		Queensland
GWORA014-08	BC ZSM Lep 10386	658[0n]	KJ169388	-		Bavarian State Collection of Zoology	*Agrius convolvuli*		Western Australia
GWORA160-08	BC ZSM Lep 10532	602[0n]	KJ168990	-		Bavarian State Collection of Zoology	*Agrius convolvuli*		Western Australia

Process ID	Sample ID	COI-5P	GB Acc. COI	28S-D2	GB Acc. 28S	Institution Storing	Species	Types	Origin
GWOR1251-07	BC ZSM Lep 02003	656[0n]	KJ169118	-		Bavarian State Collection of Zoology	*Agrius convolvuli*		Queensland
GWORB2201-08	BC ZSM Lep 11001	658[0n]	KJ169068	-		Bavarian State Collection of Zoology	*Agrius convolvuli*		Northern Territory
GWORD041-07	BC ZSM Lep 02673	657[1n]	KJ169202	578[0n]	KJ169420	Bavarian State Collection of Zoology	*Agrius convolvuli*		Queensland
LOQB072-05	Moth 072.03LZ	620[0n]	KJ169313	-		Biodiversity Institute of Ontario	*Agrius convolvuli*		Queensland
LNSWE188-06	06-NSWE-00188	658[0n]	KJ168734	-		Biodiversity Institute of Ontario	*Agrius convolvuli*		New South Wales
LOQTE740-10	gvc13992-1L	658[0n]	HM879474	-		Biodiversity Institute of Ontario	*Agrius convolvuli*		Queensland
LOQB566-05	Moth 045.03CL	658[0n]	KJ169181	-		Biodiversity Institute of Ontario	*Agrius convolvuli*		Queensland
GWORA266-08	BC ZSM Lep 10638	617[0n]	KJ168935	-		Bavarian State Collection of Zoology	*Agrius convolvuli*		Northern Territory
GWORA281-08	BC ZSM Lep 10653	637[1n]	KJ168860	-		Bavarian State Collection of Zoology	*Agrius convolvuli*		Northern Territory
GWORB2214-08	BC ZSM Lep 11014	657[1n]	KJ168834	-		Bavarian State Collection of Zoology	*Agrius convolvuli*		Northern Territory
LOQB565-05	Moth 044.03CL	658[0n]	KJ168750	-		Biodiversity Institute of Ontario	*Agrius convolvuli*		Queensland
GWORB2092-08	BC ZSM Lep 10892	618[0n]	KJ169017	-		Bavarian State Collection of Zoology	*Agrius convolvuli*		Northern Territory
GWORA189-08	BC ZSM Lep 10561	645[0n]	KJ169405	-		Bavarian State Collection of Zoology	*Agrius convolvuli*		Northern Territory
GWORB2227-08	BC ZSM Lep 11027	657[0n]	KJ168836	-		Bavarian State Collection of Zoology	*Agrius convolvuli*		Northern Territory
GWORB2187-08	BC ZSM Lep 10987	658[0n]	KJ169312	-		Bavarian State Collection of Zoology	*Agrius convolvuli*		Northern Territory
LOQB071-05	Moth 071.03LZ	572[0n]	KJ168831	-		Biodiversity Institute of Ontario	*Agrius convolvuli*		Queensland
GWORB2226-08	BC ZSM Lep 11026	658[0n]	KJ169006	-		Bavarian State Collection of Zoology	*Agrius convolvuli*		Northern Territory
LOQB076-05	Moth 076.03LZ	573[0n]	KJ169110	-		Biodiversity Institute of Ontario	*Agrius convolvuli*		Queensland
GWORB2069-08	BC ZSM Lep 10869	609[0n]	KJ168988	-		Bavarian State Collection of Zoology	*Agrius convolvuli*		Northern Territory
IMLQ131-07	IM06-0439	658[0n]	KJ169028	-		Biodiversity Institute of Ontario	*Agrius convolvuli*		Queensland
GWORB2196-08	BC ZSM Lep 10996	658[0n]	KJ169059	-		Bavarian State Collection of Zoology	*Agrius convolvuli*		Northern Territory
GWORB854-07	BC ZSM Lep 01324	617[0n]	KJ168677	-		Bavarian State Collection of Zoology	*Agrius convolvuli*		Queensland
GWORB2095-08	BC ZSM Lep 10895	658[0n]	KJ168858	-		Bavarian State Collection of Zoology	*Agrius convolvuli*		Northern Territory
GWORA032-08	BC ZSM Lep 10404	658[0n]	KJ168974	-		Bavarian State Collection of Zoology	*Agrius convolvuli*		Western Australia
GWORB2192-08	BC ZSM Lep 10992	658[0n]	KJ169284	-		Bavarian State Collection of Zoology	*Agrius convolvuli*		Northern Territory
GWORA159-08	BC ZSM Lep 10531	587[0n]	KJ169231	-		Bavarian State Collection of Zoology	*Agrius convolvuli*		Western Australia
GWOR1744-07	BC ZSM Lep 02308	658[0n]	KJ168759	559[0n]	KJ169407	Bavarian State Collection of Zoology	*Agrius convolvuli*		Queensland
GWORB2194-08	BC ZSM Lep 10994	656[0n]	KJ168630	-		Bavarian State Collection of Zoology	*Agrius convolvuli*		Northern Territory
GWOR1835-07	BC ZSM Lep 02399	595[0n]	KJ169169	-		Bavarian State Collection of Zoology	*Agrius convolvuli*		Queensland
GWORC186-07	BC ZSM Lep 02536	647[0n]	KJ168916	-		Bavarian State Collection of Zoology	*Agrius convolvuli*		Queensland
GWORB2195-08	BC ZSM Lep 10995	658[0n]	KJ169376	-		Bavarian State Collection of Zoology	*Agrius convolvuli*		Northern Territory
ANIC239-06	ANIC Gen No. 000431	658[0n]	KJ168817	-		Australian National Insect Collection	*Agrius convolvuli*		Queensland
GWORA140-08	BC ZSM Lep 10512	609[1n]	KJ168846	-		Bavarian State Collection of Zoology	*Agrius convolvuli*		Western Australia
LLISA221-06	06-NSWL-00221	658[0n]	KJ169241	-		Biodiversity Institute of Ontario	*Agrius convolvuli*		New South Wales
GWORA026-08	BC ZSM Lep 10398	657[0n]	KJ168788	-		Bavarian State Collection of Zoology	*Agrius convolvuli*		Western Australia
NSWHM029-11	BIOUG00851-H10	658[0n]	JN284552	-		Biodiversity Institute of Ontario	*Agrius convolvuli*		New South Wales

Process ID	Sample ID	COI-5P	GB Acc. COI	28S-D2	GB Acc. 28S	Institution Storing	Species	Types	Origin
GWORI526-09	BC ZSM Lep 13274	658[0n]	KJ169188	-		Bavarian State Collection of Zoology	*Agrius convolvuli*		Northern Territory
LNSWE081-06	06-NSWE-00081	658[0n]	KJ168735	-		Biodiversity Institute of Ontario	*Agrius convolvuli*		New South Wales
LOQB073-05	Moth 073.03LZ	649[0n]	KJ169370	-		Biodiversity Institute of Ontario	*Agrius convolvuli*		Queensland
GWORB2203-08	BC ZSM Lep 11003	657[1n]	KJ168911	-		Bavarian State Collection of Zoology	*Agrius convolvuli*		Northern Territory
NSWHM456-11	BIOUG00912-G05	658[0n]	JN284554	-		Biodiversity Institute of Ontario	*Agrius convolvuli*		New South Wales
GWORA101-08	BC ZSM Lep 10473	646[0n]	KJ168620	-		Bavarian State Collection of Zoology	*Agrius convolvuli*		Northern Territory
GWORB2199-08	BC ZSM Lep 10999	657[0n]	KJ169253	-		Bavarian State Collection of Zoology	*Agrius convolvuli*		Northern Territory
GWORC144-07	BC ZSM Lep 02494	655[0n]	KJ168678	-		Bavarian State Collection of Zoology	*Agrius convolvuli*		Queensland
NSWHH011-09	08-NSWHH-0011	658[0n]	KJ168983	-		Biodiversity Institute of Ontario	*Agrius convolvuli*		New South Wales
PHLCC1137-11	BIOUG01235-A01	658[0n]	KJ168892	-		Biodiversity Institute of Ontario	*Agrius convolvuli*		Australian Capital Territory
GWORA273-08	BC ZSM Lep 10645	637[3n]	KJ169360	-		Bavarian State Collection of Zoology	*Agrius convolvuli*		Northern Territory
LOQB074-05	Moth 074.03LZ	658[0n]	KJ169054	-		Biodiversity Institute of Ontario	*Agrius convolvuli*		Queensland
LOQB567-05	Moth 046.03CL	658[0n]	KJ168850	-		Biodiversity Institute of Ontario	*Agrius convolvuli*		Queensland
LOQB569-05	Moth 048.03CL	658[0n]	KJ168964	-		Biodiversity Institute of Ontario	*Agrius convolvuli*		Queensland
GWORI500-09	BC ZSM Lep 13248	658[0n]	KJ168833	-		Bavarian State Collection of Zoology	*Agrius convolvuli*		Northern Territory
GWORI485-09	BC ZSM Lep 13233	658[0n]	KJ168721	-		Bavarian State Collection of Zoology	*Agrius convolvuli*		Northern Territory
NSWHJ958-10	09-NSWHH-0971	658[0n]	HM381043	-		Biodiversity Institute of Ontario	*Agrius convolvuli*		New South Wales
GWORA279-08	BC ZSM Lep 10651	620[1n]	KJ168692	-		Bavarian State Collection of Zoology	*Agrius convolvuli*		Northern Territory
GWORC183-07	BC ZSM Lep 02533	658[0n]	KJ168963	578[0n]	KJ169412	Bavarian State Collection of Zoology	*Agrius convolvuli*		Queensland
GWORB2198-08	BC ZSM Lep 10998	632[0n]	KJ169316	-		Bavarian State Collection of Zoology	*Agrius convolvuli*		Northern Territory
LOQT747-06	gvc6449-1L	658[0n]	KJ169167	-		Biodiversity Institute of Ontario	*Agrius convolvuli*		Queensland
LNSWE136-06	06-NSWE-00136	658[0n]	KJ168824	-		Biodiversity Institute of Ontario	*Agrius convolvuli*		New South Wales
LOQB078-05	Moth 078.03LZ	572[0n]	KJ168908	-		Biodiversity Institute of Ontario	*Agrius convolvuli*		Queensland
LOQ341-04	04HBL004341	593[0n]	KJ169066	-		Biodiversity Institute of Ontario	*Agrius convolvuli*		Queensland
GWORB2213-08	BC ZSM Lep 11013	657[0n]	KJ169082	-		Bavarian State Collection of Zoology	*Agrius convolvuli*		Northern Territory
GWORC181-07	BC ZSM Lep 02531	632[0n]	KJ168727	-		Bavarian State Collection of Zoology	*Agrius convolvuli*		Queensland
GWORC184-07	BC ZSM Lep 02534	632[0n]	KJ169033	-		Bavarian State Collection of Zoology	*Agrius convolvuli*		Queensland
LOQB077-05	Moth 077.03LZ	616[0n]	KJ169121	-		Biodiversity Institute of Ontario	*Agrius convolvuli*		Queensland
GWORA267-08	BC ZSM Lep 10639	658[0n]	KJ168839	-		Bavarian State Collection of Zoology	*Agrius convolvuli*		Northern Territory
GWORB2202-08	BC ZSM Lep 11002	658[0n]	KJ169217	-		Bavarian State Collection of Zoology	*Agrius convolvuli*		Northern Territory
LSM256-11	K278668	658[0n]	KJ169101	-		Australian Museum, Sydney	*Agrius convolvuli*		New South Wales
GWORI528-09	BC ZSM Lep 13276	658[0n]	KJ169209	-		Bavarian State Collection of Zoology	*Agrius convolvuli*		Northern Territory
SPUEB036-07	BC-EMEM0976	658[0n]	KJ169371	-		Entomologisches Museum Eitschberger	*Agrius godarti*		Queensland
SPUEB037-07	BC-EMEM0977	658[0n]	KJ168929	-		Entomologisches Museum Eitschberger	*Agrius godarti*		Queensland
LOQTE444-09	gvc12637-1L	658[0n]	HM387295	-		Research Collection of Graeme V. Cocks	*Agrius godarti*		Queensland

Process ID	Sample ID	COI-5P	GB Acc. COI	28S-D2	GB Acc. 28S	Institution Storing	Species	Types	Origin
LOQTE445-09	gvc12640-1L	658[0n]	HQ989569	-		Research Collection of Graeme V. Cocks	*Agrius godarti*		Queensland
NSWHJ959-10	09-NSWHH-0972	658[0n]	HM381044	-		Biodiversity Institute of Ontario	*Agrius godarti*		New South Wales
LOQTE571-10	gvc13281-1L	658[0n]	HM387351	-		Research Collection of Graeme V. Cocks	*Agrius godarti*		Queensland
LNSWE079-06	06-NSWE-00079	618[0n]	KJ168787	-		Biodiversity Institute of Ontario	*Agrius godarti*		New South Wales
LOQTI353-11	gvc16072-1L	658[0n]	KJ169315	-		Biodiversity Institute of Ontario	*Agrius godarti*		Queensland
SOWD078-06	BC-Hax2977	608[0n]	KJ169363	-		Research Collection of Jean Haxaire	*Agrius godarti*		Queensland
LOQT842-07	gvc6558-1L	655[0n]	KJ168658	-		Biodiversity Institute of Ontario	*Agrius godarti*		Queensland
NSWHH010-09	08-NSWHH-0010	658[0n]	KJ168624	-		Biodiversity Institute of Ontario	*Agrius godarti*		New South Wales
GWORW671-10	BC EF Lep 01645	658[0n]	HM912260	-		Research Collection of Egbert Friedrich	*Agrius godarti*		Queensland
GWORW670-10	BC EF Lep 01644	658[0n]	HM912259	-		Research Collection of Egbert Friedrich	*Agrius godarti*		Queensland
LNSWC310-06	06-NSW-00310	658[0n]	JN677684	-		Agricultural Scientific Collections Unit, New South Wales	*Agrius godarti*		New South Wales
LOQTE443-09	gvc12636-1L	658[0n]	HM387294	-		Research Collection of Graeme V. Cocks	*Agrius godarti*		Queensland
SOWD079-06	BC-Hax2978	608[0n]	KJ169042	-		Research Collection of Jean Haxaire	*Agrius godarti*		Queensland
ANIC242-06	ANIC Gen No. 000434	658[0n]	KJ168784	-		Australian National Insect Collection	*Ambulyx dohertyi queenslandi*		Queensland
LOQC008-05	05-QLD-00008	658[0n]	JN677703	-		Biodiversity Institute of Ontario	*Ambulyx dohertyi queenslandi*		Queensland
ANICC169-08	ANIC Gen No. 003332	658[0n]	KJ168832	-		Australian National Insect Collection	*Ambulyx dohertyi queenslandi*		Queensland
LOQT312-06	gvc5468-1L	658[0n]	KJ169146	-		Biodiversity Institute of Ontario	*Ambulyx dohertyi queenslandi*		Queensland
SPZSM085-08	BC-ZSMRR0085	658[0n]	KJ168652	-		Bavarian State Collection of Zoology	*Ambulyx wildei*		Queensland
GWOR1246-07	BC ZSM Lep 01998	646[0n]	KJ168762	-		Bavarian State Collection of Zoology	*Ambulyx wildei*		Queensland
SPRBA762-09	BC-RBP-1810	658[0n]	GU704169	-		Research Collection of Ron Brechlin	*Ambulyx wildei*		Queensland
SPZSM084-08	BC-ZSMRR0084	658[0n]	JN677734	-		Bavarian State Collection of Zoology	*Ambulyx wildei*		Queensland
GWOR1227-07	BC ZSM Lep 01979	658[0n]	KJ168662	-		Bavarian State Collection of Zoology	*Ambulyx wildei*		Queensland
GWOR1225-07	BC ZSM Lep 01977	658[0n]	KJ168600	-		Bavarian State Collection of Zoology	*Ambulyx wildei*		Queensland
GWOR1228-07	BC ZSM Lep 01980	658[0n]	KJ169173	-		Bavarian State Collection of Zoology	*Ambulyx wildei*		Queensland
GWOR1226-07	BC ZSM Lep 01978	645[0n]	KJ168816	-		Bavarian State Collection of Zoology	*Ambulyx wildei*		Queensland
SPRBA761-09	BC-RBP-1809	658[0n]	GU704170	-		Research Collection of Ron Brechlin	*Ambulyx wildei*		Queensland
GWOR1245-07	BC ZSM Lep 01997	658[0n]	KJ168998	-		Bavarian State Collection of Zoology	*Ambulyx wildei*		Queensland
SPZSM086-08	BC-ZSMRR0086	658[0n]	KJ168628	-		Bavarian State Collection of Zoology	*Ambulyx wildei*		Queensland
GWORG363-08	BC ZSM Lep 08479	658[0n]	KJ168621	-		Bavarian State Collection of Zoology	*Angonyx papuana*		Queensland
ANICC055-08	ANIC Gen No. 003218	294[0n]	KJ169256	-		Australian National Insect Collection	*Angonyx papuana*		Queensland
LTOL325-07	JP-05-0763	658[0n]	KJ168808	-		University of Maryland	*Angonyx papuana*		Queensland
ANICC054-08	ANIC Gen No. 003217	285[0n]	KJ169153	-		Australian National Insect Collection	*Angonyx papuana*		Queensland
SPHJT088-10	BC-LTM-112	658[0n]	HQ975009	-		Research Collection of Max S. Moulds	*Cephonodes hylas australis*		Queensland
ANICC069-08	ANIC Gen No. 003232	591[0n]	KJ169079	-		Australian National Insect Collection	*Cephonodes hylas australis*		Northern Territory
SPHJT089-10	BC-LTM-113	658[0n]	HQ975010	-		Research Collection of Max S. Moulds	*Cephonodes hylas australis*		Queensland

Process ID	Sample ID	COI-5P	GB Acc. COI	28S-D2	GB Acc. 28S	Institution Storing	Species	Types	Origin
ANICC070-08	ANIC Gen No. 003233	625[1n]	KJ169254	-		Australian National Insect Collection	*Cephonodes hylas australis*		Queensland
ANICC073-08	ANIC Gen No. 003236	609[0n]	KJ169228	-		Australian National Insect Collection	*Cephonodes janus*		Queensland
ANICC074-08	ANIC Gen No. 003237	658[0n]	JN677806	-		Australian National Insect Collection	*Cephonodes janus*		Queensland
SPHJT087-10	BC-LTM-111	658[0n]	HQ975008	-		Research Collection of Max S. Moulds	*Cephonodes kingii*		Queensland
SOWC441-06	BC-Hax2340	606[0n]	KJ168771	-		Research Collection of Jean Haxaire	*Cephonodes kingii*		Queensland
SPUEB208-07	BC-EMEM1148	658[0n]	KJ169081	-		Entomologisches Museum Eitschberger	*Cephonodes kingii*		Queensland
SPUEB207-07	BC-EMEM1147	658[0n]	KJ168780	-		Entomologisches Museum Eitschberger	*Cephonodes kingii*		Queensland
SPUEB209-07	BC-EMEM1149	658[0n]	JN677807	-		Entomologisches Museum Eitschberger	*Cephonodes kingii*		Queensland
SPHJT086-10	BC-LTM-110	658[0n]	HQ975007	-		Research Collection of Max S. Moulds	*Cephonodes kingii*		Queensland
LOQTI746-12	gvc17799-1L	658[0n]	KJ169166	-		Biodiversity Institute of Ontario	*Cephonodes picus*		Queensland
SPHJT090-10	BC-LTM-114	658[0n]	HQ975011	-		Research Collection of Max S. Moulds	*Cephonodes picus*		Queensland
LOQ381-04	04HBL004381	658[0n]	KJ168924	-		Biodiversity Institute of Ontario	*Cephonodes picus*		Queensland
ANICC068-08	ANIC Gen No. 003231	609[0n]	KJ168984	-		Australian National Insect Collection	*Cephonodes picus*		Queensland
SPHJT091-10	BC-LTM-115	658[0n]	HQ975012	-		Research Collection of Max S. Moulds	*Cephonodes picus*		Queensland
GWORB866-07	BC ZSM Lep 01336	597[0n]	KJ169041	-		Bavarian State Collection of Zoology	*Cerberonoton rubescens severina*		Queensland
ANICC003-08	ANIC Gen No. 003166	609[0n]	KJ169011	-		Australian National Insect Collection	*Cerberonoton rubescens severina*		Queensland
SPTOL254-09	JP-05-0760	658[0n]	KJ169137	-		University of Maryland	*Cerberonoton rubescens severina*		Queensland
ANICC038-08	ANIC Gen No. 003201	609[0n]	KJ168809	-		Australian National Insect Collection	*Cerberonoton rubescens severina*		Queensland
ANICC011-08	ANIC Gen No. 003174	658[0n]	KJ168613	-		Australian National Insect Collection	*Cerberonoton rubescens severina*		Queensland
ANICC081-08	ANIC Gen No. 003244	658[0n]	KJ169086	-		Australian National Insect Collection	*Cerberonoton rubescens severina*		Queensland
SPTMA090-07	BC-Mel 0168	595[0n]	KJ169356	-		Research Collection of Tomas Melichar	*Cizara ardeniae*		Queensland
ANICC171-08	ANIC Gen No. 003334	273[0n]	KJ169337	-		Australian National Insect Collection	*Cizara ardeniae*		Queensland
ANICC045-08	ANIC Gen No. 003208	608[1n]	JN677823	-		Australian National Insect Collection	*Cizara ardeniae*		Queensland
SPTMA091-07	BC-Mel 0169	264[4n]	KJ169299	-		Research Collection of Tomas Melichar	*Cizara ardeniae*		Queensland
SPHJT080-10	BC-LTM-104	658[0n]	HQ975003	-		Research Collection of Max S. Moulds	*Coenotes arida*		Western Australia
SPTMC304-12	BC-Mel2305	658[0n]	KJ169382	-		Research Collection of Tomas Melichar	*Coenotes arida*		Northern Territory
SPTMC305-12	BC-Mel2306	658[0n]	KJ168835	-		Research Collection of Tomas Melichar	*Coenotes arida*		Northern Territory
SPHJT083-10	BC-LTM-107	658[0n]	HQ975006	-		Research Collection of Max S. Moulds	*Coenotes eremophilae*		Western Australia
LOQTC782-08	gvc8427-1L	632[0n]	KJ169288	-		Biodiversity Institute of Ontario	*Coenotes eremophilae*		Queensland
LOQT218-06	2006-LOQT-218	603[0n]	KJ169141	-		Biodiversity Institute of Ontario	*Coenotes eremophilae*		Queensland
LOQTC769-08	gvc8414-1L	658[0n]	KJ169227	-		Biodiversity Institute of Ontario	*Coenotes eremophilae*		Queensland
SPTMA053-07	BC-Mel 0131	625[0n]	KJ169219	-		Research Collection of Tomas Melichar	*Coenotes eremophilae*		Western Australia
LOQTC771-08	gvc8416-1L	658[0n]	KJ169037	-		Biodiversity Institute of Ontario	*Coenotes eremophilae*		Queensland
SOWF178-12	BC-Hax5036	658[0n]	KJ169260	-		Research Collection of Jean Haxaire	*Coenotes eremophilae*		Northern Territory
LOQTB023-07	gvc6797-1L	658[0n]	JN677846	-		Biodiversity Institute of Ontario	*Coenotes eremophilae*		Queensland
SPHJT082-10	BC-LTM-106	658[0n]	HQ975005	-		Research Collection of Max S. Moulds	*Coenotes eremophilae*		Western Australia

Process ID	Sample ID	COI-5P	GB Acc. COI	28S-D2	GB Acc. 28S	Institution Storing	Species	Types	Origin
LOQT053-06	2006-LOQT-053	615[2n]	KJ169232	-		Biodiversity Institute of Ontario	*Coenotes eremophilae*		Queensland
SPTMA056-07	BC-Mel 0134	658[0n]	KJ168740	-		Research Collection of Tomas Melichar	*Coenotes eremophilae*		Western Australia
LOQTC781-08	gvc8426-1L	658[0n]	KJ168698	-		Biodiversity Institute of Ontario	*Coenotes eremophilae*		Queensland
LOQTC770-08	gvc8415-1L	658[0n]	KJ169154	-		Research Collection of Graeme V. Cocks	*Coenotes eremophilae*		Queensland
SOWF177-12	BC-Hax5035	658[0n]	KJ168890	-		Research Collection of Jean Haxaire	*Coenotes eremophilae*		Northern Territory
LOQTC783-08	gvc8428-1L	658[0n]	KJ168830	-		Research Collection of Graeme V. Cocks	*Coenotes eremophilae*		Queensland
SPHJT081-10	BC-LTM-105	614[0n]	HQ975004	-		Research Collection of Max S. Moulds	*Coenotes eremophilae*		Western Australia
ANICC189-08	BC-Roug1149	608[0n]	KJ169286	-		Biodiversity Institute of Ontario	*Coequosa australasiae*		New South Wales
ANICC018-08	ANIC Gen No. 003181	658[0n]	KJ169403	-		Australian National Insect Collection	*Coequosa australasiae*		Queensland
SOWA608-06	BC-Hax0601	608[0n]	KJ168682	-		Research Collection of Jean Haxaire	*Coequosa australasiae*		Queensland
IMLR1327-11	IM10-0071	658[0n]	KJ169385	-		Biodiversity Institute of Ontario	*Coequosa australasiae*		Queensland
SPRBA754-09	BC-RBP-1797	658[0n]	GU704153	-		Research Collection of Ron Brechlin	*Coequosa australasiae*		Queensland
ANICC175-08	ANIC Gen No. 003338	658[0n]	JN677847	-		Australian National Insect Collection	*Coequosa australasiae*		New South Wales
SPHYE141-09	BC-EST0517	632[0n]	GU703909	-		Research Collection of Yves Estradel	*Coequosa australasiae*		New South Wales
SOWA609-06	BC-Hax0602	608[0n]	KJ168643	-		Research Collection of Jean Haxaire	*Coequosa australasiae*		Northern Territory
NSWHJ045-10	09-NSWHH-0058	658[0n]	HM380399	-		Biodiversity Institute of Ontario	*Coequosa triangularis*		New South Wales
SPTOL247-08	AZ-06-0211	658[0n]	KJ169255	-		University of Maryland	*Coequosa triangularis*		New South Wales
ANICC037-08	ANIC Gen No. 003200	609[0n]	KJ169183	-		Australian National Insect Collection	*Coequosa triangularis*		Queensland
NSWHM2022-11	BIOUG00961-A05	658[0n]	JN279360	-		Biodiversity Institute of Ontario	*Coequosa triangularis*		New South Wales
ANICC046-08	ANIC Gen No. 003209	658[0n]	JN677848	-		Australian National Insect Collection	*Coequosa triangularis*		New South Wales
ANICC057-08	ANIC Gen No. 003220	270[0n]	KJ168960	-		Australian National Insect Collection	*Daphnis dohertyi*		Queensland
SOWE232-07	BC-Hax4131	658[0n]	KJ168637	-		Research Collection of Jean Haxaire	*Daphnis moorei*		
GWORI498-09	BC ZSM Lep 13246	658[0n]	KJ169164	-		Bavarian State Collection of Zoology	*Daphnis moorei*		Northern Territory
SOWE231-07	BC-Hax4130	658[0n]	KJ169078	-		Research Collection of Jean Haxaire	*Daphnis moorei*		
GWORB871-07	BC ZSM Lep 01341	632[0n]	KJ169324	-		Bavarian State Collection of Zoology	*Daphnis moorei*		Queensland
GWORA134-08	BC ZSM Lep 10506	649[0n]	KJ168889	-		Bavarian State Collection of Zoology	*Daphnis moorei*		Western Australia
LOQTB197-07	gvc6960-1L	658[0n]	KJ169222	-		Biodiversity Institute of Ontario	*Daphnis moorei*		Queensland
GWORB852-07	BC ZSM Lep 01322	598[0n]	KJ168694	-		Bavarian State Collection of Zoology	*Daphnis moorei*		Queensland
LOQT221-06	2006-LOQT-221	608[0n]	KJ169267	-		Biodiversity Institute of Ontario	*Daphnis moorei*		Queensland
GWORB851-07	BC ZSM Lep 01321	632[0n]	KJ169149	-		Bavarian State Collection of Zoology	*Daphnis moorei*		Queensland
GWORB883-07	BC ZSM Lep 01353	632[0n]	KJ169355	-		Bavarian State Collection of Zoology	*Daphnis moorei*		Queensland
IMLR1337-11	IM10-0108	658[0n]	KJ169322	-		Biodiversity Institute of Ontario	*Daphnis placida*		Queensland
IMLR1229-11	IM09-0018	658[0n]	KJ169306	-		Biodiversity Institute of Ontario	*Daphnis placida*		Queensland
LOQTE823-10	gvc14262-1L	658[0n]	HQ572365	-		Biodiversity Institute of Ontario	*Daphnis placida*		Queensland
SPTMC370-12	BC-Mel2371	658[0n]	KJ168869	-		Research Collection of Tomas Melichar	*Daphnis placida*		Queensland
SPTMC369-12	BC-Mel2370	658[0n]	KJ169280	-		Research Collection of Tomas Melichar	*Daphnis placida*		Western Australia

Process ID	Sample ID	COI-5P	GB Acc. COI	28S-D2	GB Acc. 28S	Institution Storing	Species	Types	Origin
NSWBB1213-08	07-NSWBB-1213	658[0n]	KJ168810	-		Biodiversity Institute of Ontario	*Daphnis placida*		New South Wales
ANICC179-08	ANIC Gen No. 003342	658[0n]	KJ169301	-		Australian National Insect Collection	*Daphnis placida*		Queensland
SOWE234-07	BC-Hax4133	658[0n]	KJ168900	-		Research Collection of Jean Haxaire	*Daphnis placida*		
LOQTD887-09	gvc10944-1L	658[0n]	KJ169113	-		Research Collection of Graeme V. Cocks	*Daphnis placida*		Queensland
LOQT786-07	gvc6499-1L	623[0n]	KJ168712	-		Biodiversity Institute of Ontario	*Daphnis placida*		Queensland
ANICC176-08	ANIC Gen No. 003339	609[0n]	KJ169243	-		Australian National Insect Collection	*Daphnis placida*		Queensland
GWOR1243-07	BC ZSM Lep 01995	658[0n]	KJ168730	-		Bavarian State Collection of Zoology	*Daphnis placida*		Queensland
LOQTB552-07	gvc7204-1L	658[0n]	KJ168710	-		Biodiversity Institute of Ontario	*Daphnis protrudens*		Queensland
GWORB853-07	BC ZSM Lep 01323	627[0n]	KJ168874	-		Bavarian State Collection of Zoology	*Daphnis protrudens*		Queensland
ANICC183-08	ANIC Gen No. 003346	658[0n]	JN677873	-		Australian National Insect Collection	*Daphnis protrudens*		Queensland
LOQTI136-10	gvc15252-1L	658[0n]	JF857277	-		Research Collection of Graeme V. Cocks	*Eupanacra splendens*		Queensland
GWORB3274-08	BC ZSM Lep 05812	658[0n]	KJ169346	-		Bavarian State Collection of Zoology	*Eupanacra splendens*		Queensland
GWORB3275-08	BC ZSM Lep 05813	658[0n]	KJ169380	-		Bavarian State Collection of Zoology	*Eupanacra splendens*		Queensland
LTOL253-07	JP-05-0766	658[0n]	KJ168814	-		University of Maryland	*Eupanacra splendens*		Queensland
GWOR1240-07	BC ZSM Lep 01992	657[0n]	KJ168738	-		Bavarian State Collection of Zoology	*Eupanacra splendens*		Queensland
LOQTC288-07	gvc7924-1L	659[0n]	KJ168867	604[0n]	KJ169411	Biodiversity Institute of Ontario	*Eupanacra splendens*		Queensland
LOQTD377-08	gvc8982-1L	658[0n]	KJ169072	601[0n]	KJ169415	Research Collection of Graeme V. Cocks	*Eupanacra splendens*		Queensland
GWORB3276-08	BC ZSM Lep 05814	658[0n]	KJ168934	-		Bavarian State Collection of Zoology	*Eupanacra splendens*		Queensland
SOWB487-06	BC-Hax1480	608[0n]	KJ168655	-		Research Collection of Jean Haxaire	*Eupanacra splendens*		Queensland
LOQTI673-12	gvc17580-1L	638[0n]	KJ168884	-		Biodiversity Institute of Ontario	*Eupanacra splendens*		Queensland
LOQTE774-10	gvc14103-1L	658[0n]	HQ572319	-		Research Collection of Graeme V. Cocks	*Eupanacra splendens*		Queensland
GWORB893-07	BC ZSM Lep 01363	632[0n]	KJ168696	-		Bavarian State Collection of Zoology	*Eupanacra splendens*		Queensland
GWOR1244-07	BC ZSM Lep 01996	658[0n]	JN677958	601[0n]	KJ169413	Bavarian State Collection of Zoology	*Eupanacra splendens*		Queensland
LOQTD126-08	gvc8725-1L	658[0n]	KJ169189	599[0n]	KJ169418	Research Collection of Graeme V. Cocks	*Eupanacra splendens*		Queensland
LOQT004-06	2006-LOQT-004	658[0n]	KJ169318	-		Biodiversity Institute of Ontario	*Eupanacra splendens*		Queensland
SPHJT103-10	BC-LTM-127	658[0n]	HQ975023	-		Research Collection of Max S. Moulds	*Gnathothlibus australiensis*		Queensland
SPHJT101-10	BC-LTM-125	658[0n]	HQ975021	-		Research Collection of Max S. Moulds	*Gnathothlibus australiensis*		Queensland
SPHJT102-10	BC-LTM-126	658[0n]	HQ975022	-		Research Collection of Max S. Moulds	*Gnathothlibus australiensis*		Queensland
ANICC083-08	ANIC Gen No. 003246	609[0n]	KJ169062	-		Australian National Insect Collection	*Gnathothlibus australiensis*		Queensland
SPHJT104-10	BC-LTM-128	658[0n]	HQ975024	-		Research Collection of Max S. Moulds	*Gnathothlibus australiensis*		Queensland
LOQTB138-07	gvc6901-1L	658[0n]	JX438282	-		Biodiversity Institute of Ontario	*Gnathothlibus eras*		Queensland
GWORD037-07	BC ZSM Lep 02669	658[0n]	KJ169025	-		Bavarian State Collection of Zoology	*Gnathothlibus eras*		Queensland
LOQTD850-09	gvc10628-1L	658[0n]	KJ169235	-		Research Collection of Graeme V. Cocks	*Gnathothlibus eras*		Queensland
ANICC067-08	ANIC Gen No. 003230	658[0n]	JX438280	-		Australian National Insect Collection	*Gnathothlibus eras*		Queensland
LOQTB079-07	gvc6840-1L	658[0n]	JX438290	-		Biodiversity Institute of Ontario	*Gnathothlibus eras*		Queensland
LOQTB141-07	gvc6905-1L	645[0n]	KJ168895	-		Biodiversity Institute of Ontario	*Gnathothlibus eras*		Queensland

Process ID	Sample ID	COI-5P	GB Acc. COI	28S-D2	GB Acc. 28S	Institution Storing	Species	Types	Origin
LOQB001-05	Moth 001.01LZ	658[0n]	KJ169386	-		Biodiversity Institute of Ontario	*Gnathothlibus eras*		Queensland
SPHJT009-09	BC-JT-21M	658[0n]	HM384235	-		Research Collection of James A. Tuttle	*Gnathothlibus eras*		Queensland
SPHJT004-09	BC-JT-22M	658[0n]	HM384231	-		Research Collection of James A. Tuttle	*Gnathothlibus eras*		Queensland
GWORD038-07	BC ZSM Lep 02670	658[0n]	KJ169198	-		Bavarian State Collection of Zoology	*Gnathothlibus eras*		Queensland
LOQTE814-10	gvc14230-1L	658[0n]	HQ572356	-		Biodiversity Institute of Ontario	*Gnathothlibus eras*		Queensland
ANICC089-08	ANIC Gen No. 003252	658[0n]	JX438292	-		Australian National Insect Collection	*Gnathothlibus eras*		Northern Territory
GWOR1827-07	BC ZSM Lep 02391	600[0n]	KJ169055	-		Bavarian State Collection of Zoology	*Gnathothlibus eras*		Queensland
SPHJT105-10	BC-LTM-129	605[1n]	HQ975025	-		Research Collection of Max S. Moulds	*Gnathothlibus eras*		Queensland
SPHJT108-10	BC-LTM-132	658[0n]	HQ975028	-		Research Collection of Max S. Moulds	*Gnathothlibus eras*		Queensland
LOQTB140-07	gvc6904-1L	658[0n]	JX438293	-		Biodiversity Institute of Ontario	*Gnathothlibus eras*		Queensland
LOQB298-05	Moth 298.01LZ	569[0n]	KJ169357	-		Biodiversity Institute of Ontario	*Gnathothlibus eras*		Queensland
SPHJT110-10	BC-LTM-134	658[0n]	HQ975030	-		Research Collection of Max S. Moulds	*Gnathothlibus eras*		Queensland
ANICC088-08	ANIC Gen No. 003251	658[0n]	JX438277	-		Australian National Insect Collection	*Gnathothlibus eras*		Queensland
LTOL251-07	JP-05-0761	658[0n]	KJ168973	-		University of Maryland	*Gnathothlibus eras*		Queensland
LOQTI636-12	gvc17314-1L	658[0n]	KJ168622	-		Biodiversity Institute of Ontario	*Gnathothlibus eras*		Queensland
GWOR1834-07	BC ZSM Lep 02398	600[0n]	KJ168636	-		Bavarian State Collection of Zoology	*Gnathothlibus eras*		Queensland
SPHJT106-10	BC-LTM-130	658[0n]	HQ975026	-		Research Collection of Max S. Moulds	*Gnathothlibus eras*		Queensland
GWOR1833-07	BC ZSM Lep 02397	626[0n]	KJ168752	-		Bavarian State Collection of Zoology	*Gnathothlibus eras*		Queensland
SPHJT109-10	BC-LTM-133	658[0n]	HQ975029	-		Research Collection of Max S. Moulds	*Gnathothlibus eras*		Queensland
SOWE582-07	BC-Hax4481	658[0n]	HM384195	-		Research Collection of Jean Haxaire	*Gnathothlibus eras*		
SPHJT107-10	BC-LTM-131	658[0n]	HQ975027	-		Research Collection of Max S. Moulds	*Gnathothlibus eras*		Queensland
GWORD036-07	BC ZSM Lep 02668	658[0n]	KJ169276	-		Bavarian State Collection of Zoology	*Gnathothlibus eras*		Queensland
LOQB299-05	Moth 299.01LZ	574[0n]	KJ168879	-		Biodiversity Institute of Ontario	*Gnathothlibus eras*		Queensland
ANICC090-08	ANIC Gen No. 003253	658[0n]	JX438279	-		Australian National Insect Collection	*Gnathothlibus eras*		Northern Territory
SPHJT147-11	BC-LTM-169	658[0n]	KJ169014	-		Research Collection of Max S. Moulds	*Hippotion brennus*		Queensland
SPTMA922-09	BC-Mel 1000	643[0n]	KJ168920	-		Research Collection of Tomas Melichar	*Hippotion brennus*		Queensland
ANICC031-08	ANIC Gen No. 003194	614[0n]	KJ169212	-		Australian National Insect Collection	*Hippotion brennus*		Queensland
LOQTE455-09	gvc12757-1L	658[0n]	HM387305	-		Biodiversity Institute of Ontario	*Hippotion brennus*		Queensland
ANICC092-08	ANIC Gen No. 003255	658[0n]	JN678015	-		Australian National Insect Collection	*Hippotion brennus*		Queensland
GWORY462-10	BC EF Lep 03416	658[0n]	HM913603	-		Research Collection of Egbert Friedrich	*Hippotion brennus*		Queensland
SPHJT149-11	BC-LTM-171	658[0n]	KJ168907	-		Research Collection of B. M. Fjellstad	*Hippotion brennus*		New South Wales
ANICC030-08	ANIC Gen No. 003193	613[0n]	KJ169157	-		Australian National Insect Collection	*Hippotion brennus*		Queensland
LNSWC574-08	AM 2295	658[0n]	KJ169300	-		Orange Agricultural Institute	*Hippotion celerio*		New South Wales
NSWHJ052-10	09-NSWHH-0065	658[0n]	HM380405	-		Biodiversity Institute of Ontario	*Hippotion celerio*		New South Wales
LNSWE058-06	06-NSWE-00058	658[0n]	KJ168905	-		Biodiversity Institute of Ontario	*Hippotion celerio*		New South Wales
LNSWE090-06	06-NSWE-00090	658[0n]	KJ168714	-		Biodiversity Institute of Ontario	*Hippotion celerio*		New South Wales

Process ID	Sample ID	COI-5P	GB Acc. COI	28S-D2	GB Acc. 28S	Institution Storing	Species	Types	Origin
LNSWE050-06	06-NSWE-00050	658[0n]	KJ169298	-		Biodiversity Institute of Ontario	*Hippotion celerio*		New South Wales
LNSWE084-06	06-NSWE-00084	658[0n]	KJ169038	-		Biodiversity Institute of Ontario	*Hippotion celerio*		New South Wales
LNSWC576-08	AM 2297	658[0n]	KJ168601	-		Orange Agricultural Institute	*Hippotion celerio*		New South Wales
LNSWE054-06	06-NSWE-00054	615[0n]	KJ168822	-		Biodiversity Institute of Ontario	*Hippotion celerio*		New South Wales
LNSWE041-06	06-NSWE-00041	658[0n]	KJ169341	-		Biodiversity Institute of Ontario	*Hippotion celerio*		New South Wales
PHLCC1141-11	BIOUG01235-A05	658[0n]	KJ168975	-		Biodiversity Institute of Ontario	*Hippotion celerio*		Australian Capital Territory
LNSWE052-06	06-NSWE-00052	658[0n]	KJ169220	-		Biodiversity Institute of Ontario	*Hippotion celerio*		New South Wales
LOQTI613-12	gvc17129-1L	658[0n]	KJ169200	-		Biodiversity Institute of Ontario	*Hippotion celerio*		Queensland
LNSWE004-06	06-NSWE-00004	600[0n]	KJ169195	-		Biodiversity Institute of Ontario	*Hippotion celerio*		New South Wales
PHLCC1153-11	BIOUG01235-B05	658[0n]	KJ168728	-		Biodiversity Institute of Ontario	*Hippotion celerio*		Australian Capital Territory
LNSWE093-06	06-NSWE-00093	658[0n]	KJ168675	-		Biodiversity Institute of Ontario	*Hippotion celerio*		New South Wales
LNSWE127-06	06-NSWE-00127	612[3n]	KJ168994	-		Biodiversity Institute of Ontario	*Hippotion celerio*		New South Wales
LNSWE123-06	06-NSWE-00123	613[0n]	KJ169216	-		Biodiversity Institute of Ontario	*Hippotion celerio*		New South Wales
LNSWE108-06	06-NSWE-00108	560[0n]	KJ169009	-		Biodiversity Institute of Ontario	*Hippotion celerio*		New South Wales
LNSWE055-06	06-NSWE-00055	600[0n]	KJ169221	-		Biodiversity Institute of Ontario	*Hippotion celerio*		New South Wales
LNSWE128-06	06-NSWE-00128	658[0n]	KJ169060	-		Biodiversity Institute of Ontario	*Hippotion celerio*		New South Wales
LNSWB153-05	05-NSW-01093	658[0n]	KJ169152	-		New South Wales Department of Primary Industries	*Hippotion celerio*		New South Wales
LNSWE131-06	06-NSWE-00131	561[0n]	KJ169402	-		Biodiversity Institute of Ontario	*Hippotion celerio*		New South Wales
LNSWE138-06	06-NSWE-00138	591[0n]	KJ169084	-		Biodiversity Institute of Ontario	*Hippotion celerio*		New South Wales
LNSWE102-06	06-NSWE-00102	658[0n]	KJ169273	-		Biodiversity Institute of Ontario	*Hippotion celerio*		New South Wales
LOQTI045-10	gvc14670-1L	658[0n]	JF857214	-		Biodiversity Institute of Ontario	*Hippotion celerio*		Queensland
LNSWE126-06	06-NSWE-00126	583[1n]	KJ169330	-		Biodiversity Institute of Ontario	*Hippotion celerio*		New South Wales
LNSWC573-08	AM 2294	658[0n]	KJ168883	-		Orange Agricultural Institute	*Hippotion celerio*		New South Wales
LNSWE072-06	06-NSWE-00072	600[0n]	KJ169384	-		Biodiversity Institute of Ontario	*Hippotion celerio*		New South Wales
LNSWE137-06	06-NSWE-00137	533[0n]	KJ168813	-		Biodiversity Institute of Ontario	*Hippotion celerio*		New South Wales
LNSWB151-05	05-NSW-01091	658[0n]	KJ168843	-		New South Wales Department of Primary Industries	*Hippotion celerio*		New South Wales
LNSWE068-06	06-NSWE-00068	615[0n]	KJ168639	-		Biodiversity Institute of Ontario	*Hippotion celerio*		New South Wales
LNSWB152-05	05-NSW-01092	658[0n]	KJ169393	-		New South Wales Department of Primary Industries	*Hippotion celerio*		New South Wales
LNSWE106-06	06-NSWE-00106	595[0n]	KJ168987	-		Biodiversity Institute of Ontario	*Hippotion celerio*		New South Wales
LNSWE048-06	06-NSWE-00048	597[0n]	KJ169350	-		Biodiversity Institute of Ontario	*Hippotion celerio*		New South Wales
LNSWC575-08	AM 2296	658[0n]	KJ168849	-		Orange Agricultural Institute	*Hippotion celerio*		New South Wales
LNSWE087-06	06-NSWE-00087	578[0n]	KJ169208	-		Biodiversity Institute of Ontario	*Hippotion celerio*		New South Wales
GWORA089-08	BC ZSM Lep 10461	655[0n]	KJ168803	-		Bavarian State Collection of Zoology	*Hippotion celerio*		Western Australia
LSM1761-11	am10761	658[0n]	KJ169197	-		Australian National Insect Collection	*Hippotion celerio*		New South Wales
LNSWE077-06	06-NSWE-00077	656[0n]	KJ168928	-		Biodiversity Institute of Ontario	*Hippotion celerio*		New South Wales

Process ID	Sample ID	COI-5P	GB Acc. COI	28S-D2	GB Acc. 28S	Institution Storing	Species	Types	Origin
LNSWE053-06	06-NSWE-00053	658[0n]	KJ168701	-		Biodiversity Institute of Ontario	*Hippotion celerio*		New South Wales
LNSWC312-06	06-NSW-00312	658[0n]	KJ169107	-		Agricultural Scientific Collections Unit, New South Wales	*Hippotion celerio*		New South Wales
GWORN207-09	BC ZSM Lep 18235	658[0n]	GU704659	-		Bavarian State Collection of Zoology	*Hippotion celerio*		Western Australia
LOQTE112-09	gvc11555-1L	658[0n]	KJ168649	-		Research Collection of Graeme V. Cocks	*Hippotion rosetta*		Queensland
SPTMB669-11	BC-Mel1671	658[0n]	JN281158	-		Research Collection of Tomas Melichar	*Hippotion rosetta*		Western Australia
SPHJT148-11	BC-LTM-170	658[0n]	KJ169012	-		Research Collection of Max S. Moulds	*Hippotion rosetta*		Queensland
SPHJT150-11	BC-LTM-173	658[0n]	KJ168931	-		Research Collection of David Lane	*Hippotion rosetta*		Queensland
SPHJT151-11	BC-LTM-172	658[0n]	KJ168603	-		Research Collection of David Lane	*Hippotion rosetta*		Queensland
SPHJT145-11	BC-LTM-167	658[0n]	KJ168799	-		Research Collection of Max S. Moulds	*Hippotion rosetta*		Queensland
ANICC028-08	ANIC Gen No. 003191	622[0n]	KJ168878	-		Australian National Insect Collection	*Hippotion rosetta*		Queensland
ANICC093-08	ANIC Gen No. 003256	609[0n]	KJ168952	-		Australian National Insect Collection	*Hippotion rosetta*		Queensland
LOQTB001-07	gvc6770-1L	658[0n]	KJ169128	599[0n]	KJ169416	Biodiversity Institute of Ontario	*Hippotion rosetta*		Queensland
SPTMB668-11	BC-Mel1670	658[0n]	JN281157	-		Research Collection of Tomas Melichar	*Hippotion rosetta*		Northern Territory
SPHJT146-11	BC-LTM-168	658[0n]	KJ168793	-		Research Collection of Max S. Moulds	*Hippotion rosetta*		Queensland
LOQTB732-07	gvc7425-1L	658[0n]	KJ169266	599[0n]	KJ169422	Biodiversity Institute of Ontario	*Hippotion rosetta*		Queensland
ANICC029-08	ANIC Gen No. 003192	609[0n]	KJ169251	-		Australian National Insect Collection	*Hippotion rosetta*		Queensland
LOQTE032-09	gvc11402-1L	657[1n]	KJ169381	-		Research Collection of Graeme V. Cocks	*Hippotion rosetta*		Queensland
LOQTE728-10	gvc13945-1L	658[0n]	KJ168718	-		Research Collection of Graeme V. Cocks	*Hippotion rosetta*		Queensland
LOQTE247-09	gvc11839-1L	658[0n]	GU669940	-		Research Collection of Graeme V. Cocks	*Hippotion rosetta*		Queensland
LOQTE888-10	gvc14422-1L	658[0n]	HQ572424	-		Research Collection of Graeme V. Cocks	*Hippotion rosetta*		Queensland
GWORD048-07	BC ZSM Lep 02680	656[0n]	KJ169282	599[0n]	KJ169423	Bavarian State Collection of Zoology	*Hippotion rosetta*		Queensland
GWORY466-10	BC EF Lep 03420	658[0n]	HM913606	-		Research Collection of Egbert Friedrich	*Hippotion rosetta*		Queensland
LOQTE407-09	gvc12482-1L	658[0n]	GU670093	-		Research Collection of Graeme V. Cocks	*Hippotion rosetta*		Queensland
LOQC039-05	05-QLD-00039	658[0n]	KJ169194	599[0n]	KJ169419	Biodiversity Institute of Ontario	*Hippotion rosetta*		Queensland
LOQB451-05	Moth 135.03CC	596[0n]	KJ168828	-		Biodiversity Institute of Ontario	*Hippotion rosetta*		Queensland
LOQTE907-10	gvc14453-1L	658[0n]	HQ572439	-		Research Collection of Graeme V. Cocks	*Hippotion rosetta*		Queensland
LOQTD526-08	gvc9138-1L	658[0n]	KJ169023	599[0n]	KJ169414	Research Collection of Graeme V. Cocks	*Hippotion rosetta*		Queensland
GWORY465-10	BC EF Lep 03419	658[0n]	HM913605	-		Research Collection of Egbert Friedrich	*Hippotion rosetta*		Queensland
LOQC040-05	05-QLD-00040	519[0n]	KJ168769	599[0n]	KJ169409	Biodiversity Institute of Ontario	*Hippotion rosetta*		Queensland
GWORY463-10	BC EF Lep 03417	658[0n]	HM913604	-		Research Collection of Egbert Friedrich	*Hippotion rosetta*		Queensland
GWORN205-09	BC ZSM Lep 18233	658[0n]	GU704660	-		Bavarian State Collection of Zoology	*Hippotion scrofa*		Western Australia
LNSWE086-06	06-NSWE-00086	600[0n]	KJ169204	-		Biodiversity Institute of Ontario	*Hippotion scrofa*		New South Wales
LNSWE112-06	06-NSWE-00112	594[1n]	KJ168716	-		Biodiversity Institute of Ontario	*Hippotion scrofa*		New South Wales
NSWHM017-11	BIOUG00851-G10	658[0n]	JN280984	-		Biodiversity Institute of Ontario	*Hippotion scrofa*		New South Wales
NSWHJ964-10	09-NSWHH-0977	658[0n]	HM381049	-		Biodiversity Institute of Ontario	*Hippotion scrofa*		New South Wales

Process ID	Sample ID	COI-5P	GB Acc. COI	28S-D2	GB Acc. 28S	Institution Storing	Species	Types	Origin
NSWHM451-11	BIOUG00912-F12	658[0n]	JN280991	-		Biodiversity Institute of Ontario	*Hippotion scrofa*		New South Wales
NSWHH051-09	08-NSWHH-0051	658[0n]	KJ169210	-		Biodiversity Institute of Ontario	*Hippotion scrofa*		New South Wales
LNSWE124-06	06-NSWE-00124	603[0n]	KJ168872	-		Biodiversity Institute of Ontario	*Hippotion scrofa*		New South Wales
LNSWB126-05	05-NSW-01066	658[0n]	KJ168679	-		New South Wales Department of Primary Industries	*Hippotion scrofa*		New South Wales
LNSWE057-06	06-NSWE-00057	658[0n]	KJ169265	-		Biodiversity Institute of Ontario	*Hippotion scrofa*		New South Wales
LOQT486-06	gvc6313-1L	658[0n]	KJ169126	-		Biodiversity Institute of Ontario	*Hippotion scrofa*		Queensland
LNSWE097-06	06-NSWE-00097	595[0n]	KJ169185	-		Biodiversity Institute of Ontario	*Hippotion scrofa*		New South Wales
NSWHH053-09	08-NSWHH-0053	658[0n]	KJ169361	-		Biodiversity Institute of Ontario	*Hippotion scrofa*		New South Wales
NSWHM2119-11	BIOUG00961-C06	658[0n]	JN306736	-		Biodiversity Institute of Ontario	*Hippotion scrofa*		New South Wales
GWORC178-07	BC ZSM Lep 02528	595[0n]	KJ168614	-		Bavarian State Collection of Zoology	*Hippotion scrofa*		Queensland
LNSWE105-06	06-NSWE-00105	596[0n]	KJ169304	-		Biodiversity Institute of Ontario	*Hippotion scrofa*		New South Wales
LNSWE005-06	06-NSWE-00005	564[0n]	KJ169205	-		Biodiversity Institute of Ontario	*Hippotion scrofa*		New South Wales
IMLQ127-07	IM06-0428	656[0n]	KJ168786	-		Biodiversity Institute of Ontario	*Hippotion scrofa*		Queensland
NSWHM2154-11	BIOUG00961-F05	658[0n]	JN306772	-		Biodiversity Institute of Ontario	*Hippotion scrofa*		New South Wales
NSWHH050-09	08-NSWHH-0050	658[0n]	KJ169123	-		Biodiversity Institute of Ontario	*Hippotion scrofa*		New South Wales
NSWHJ965-10	09-NSWHH-0978	658[0n]	HM381050	-		Biodiversity Institute of Ontario	*Hippotion scrofa*		New South Wales
LNSWE103-06	06-NSWE-00103	538[0n]	KJ169057	-		Biodiversity Institute of Ontario	*Hippotion scrofa*		New South Wales
GWORI552-09	BC ZSM Lep 13300	658[0n]	KJ168776	-		Bavarian State Collection of Zoology	*Hippotion scrofa*		Northern Territory
NSWBB1216-08	07-NSWBB-1216	656[0n]	KJ169230	-		Biodiversity Institute of Ontario	*Hippotion scrofa*		New South Wales
LNSWE026-06	06-NSWE-00026	658[0n]	KJ168686	-		Biodiversity Institute of Ontario	*Hippotion scrofa*		New South Wales
LNSWE049-06	06-NSWE-00049	549[0n]	KJ169127	-		Biodiversity Institute of Ontario	*Hippotion scrofa*		New South Wales
LNSWC308-06	06-NSW-00308	658[0n]	KJ169332	-		Agricultural Scientific Collections Unit, New South Wales	*Hippotion scrofa*		New South Wales
AMWW379-12	K292664	591[0n]	KJ169050	-		Australian Museum, Sydney	*Hippotion scrofa*		New South Wales
NSWHJ191-10	09-NSWHH-0204	638[0n]	HM888482	-		Biodiversity Institute of Ontario	*Hippotion scrofa*		New South Wales
LNSWE078-06	06-NSWE-00078	595[0n]	KJ168857	-		Biodiversity Institute of Ontario	*Hippotion scrofa*		New South Wales
NSWHH058-09	08-NSWHH-0058	658[0n]	KJ168949	-		Biodiversity Institute of Ontario	*Hippotion scrofa*		New South Wales
LNSWE059-06	06-NSWE-00059	658[0n]	KJ168629	-		Biodiversity Institute of Ontario	*Hippotion scrofa*		New South Wales
LNSWC637-08	AM 2358	658[0n]	KJ168732	-		Orange Agricultural Institute	*Hippotion scrofa*		New South Wales
LNSWC638-08	AM 2359	658[0n]	KJ168746	-		Orange Agricultural Institute	*Hippotion scrofa*		New South Wales
LNSWE063-06	06-NSWE-00063	577[0n]	KJ169325	-		Biodiversity Institute of Ontario	*Hippotion scrofa*		New South Wales
PHLCC1142-11	BIOUG01235-A06	658[0n]	KJ168634	-		Biodiversity Institute of Ontario	*Hippotion scrofa*		Australian Capital Territory
LNSWE113-06	06-NSWE-00113	658[0n]	JN678037	-		Biodiversity Institute of Ontario	*Hippotion scrofa*		New South Wales
LNSWE120-06	06-NSWE-00120	576[2n]	KJ169258	-		Biodiversity Institute of Ontario	*Hippotion scrofa*		New South Wales
LNSWC306-06	06-NSW-00306	658[0n]	KJ169192	-		Agricultural Scientific Collections Unit, New South Wales	*Hippotion scrofa*		New South Wales
GWORN203-09	BC ZSM Lep 18231	658[0n]	GU704661	-		Bavarian State Collection of Zoology	*Hippotion scrofa*		Western Australia

Process ID	Sample ID	COI-5P	GB Acc. COI	28S-D2	GB Acc. 28S	Institution Storing	Species	Types	Origin
LNSWE095-06	06-NSWE-00095	595[0n]	KJ168865	-		Biodiversity Institute of Ontario	*Hippotion scrofa*		New South Wales
LSM1704-11	am10638	658[0n]	KJ169016	-		Australian National Insect Collection	*Hippotion scrofa*		New South Wales
NSWHJ962-10	09-NSWHH-0975	658[0n]	HM381047	-		Biodiversity Institute of Ontario	*Hippotion scrofa*		New South Wales
LCANA353-06	05-ACTC-353	658[0n]	KJ168702	-		Biodiversity Institute of Ontario	*Hippotion scrofa*		Australian Capital Territory
LNSWF492-06	06-NSWE-01432	658[0n]	KJ168917	-		Biodiversity Institute of Ontario	*Hippotion scrofa*		New South Wales
NSWHJ619-10	09-NSWHH-0632	658[0n]	HM380810	-		Biodiversity Institute of Ontario	*Hippotion scrofa*		New South Wales
SPRBA166-08	BC-RBP-0166	658[0n]	KJ169130	-		Research Collection of Ron Brechlin	*Hippotion scrofa*		Queensland
LOQTI638-12	gvc17316-1L	658[0n]	KJ168819	-		Biodiversity Institute of Ontario	*Hippotion scrofa*		Queensland
LNSWE083-06	06-NSWE-00083	658[0n]	KJ169296	-		Biodiversity Institute of Ontario	*Hippotion scrofa*		New South Wales
LNSWE074-06	06-NSWE-00074	658[0n]	KJ168638	-		Biodiversity Institute of Ontario	*Hippotion scrofa*		New South Wales
LNSWE076-06	06-NSWE-00076	581[0n]	KJ169046	-		Biodiversity Institute of Ontario	*Hippotion scrofa*		New South Wales
LNSWE104-06	06-NSWE-00104	594[0n]	KJ169145	-		Biodiversity Institute of Ontario	*Hippotion scrofa*		New South Wales
IMLQ233-07	IM07-0165	658[0n]	KJ168968	-		Biodiversity Institute of Ontario	*Hippotion scrofa*		Queensland
LNSWE111-06	06-NSWE-00111	549[1n]	KJ169391	-		Biodiversity Institute of Ontario	*Hippotion scrofa*		New South Wales
LNSWE046-06	06-NSWE-00046	658[0n]	KJ168856	-		Biodiversity Institute of Ontario	*Hippotion scrofa*		New South Wales
SPRBA756-09	BC-RBP-1800	658[0n]	GU704163	-		Research Collection of Ron Brechlin	*Hippotion scrofa*		Queensland
LNSWC307-06	06-NSW-00307	658[0n]	KJ168659	-		Agricultural Scientific Collections Unit, New South Wales	*Hippotion scrofa*		New South Wales
LOQTC419-07	gvc8055-1L	658[0n]	KJ169283	-		Biodiversity Institute of Ontario	*Hippotion scrofa*		Queensland
NSWHM2102-11	BIOUG00961-B01	658[0n]	JN306721	-		Biodiversity Institute of Ontario	*Hippotion scrofa*		New South Wales
LNSWE025-06	06-NSWE-00025	658[0n]	KJ169293	-		Biodiversity Institute of Ontario	*Hippotion scrofa*		New South Wales
LNSWE135-06	06-NSWE-00135	658[0n]	KJ168978	-		Biodiversity Institute of Ontario	*Hippotion scrofa*		New South Wales
LNSWE117-06	06-NSWE-00117	594[1n]	KJ169323	-		Biodiversity Institute of Ontario	*Hippotion scrofa*		New South Wales
AMWW022-11	K290762	658[0n]	KJ169089	-		Australian Museum, Sydney	*Hippotion scrofa*		New South Wales
ANIC316-06	ANIC Gen No. 000508	658[0n]	KJ168875	-		Australian National Insect Collection	*Hippotion velox*		Queensland
ANIC315-06	ANIC Gen No. 000507	658[0n]	KJ169087	-		Australian National Insect Collection	*Hippotion velox*		Queensland
SPTMA866-09	BC-Mel 0944	649[0n]	KJ169398	-		Research Collection of Tomas Melichar	*Hippotion velox*		Queensland
LOQTB799-07	gvc7494-1L	658[0n]	KJ169115	-		Biodiversity Institute of Ontario	*Hippotion velox*		Queensland
LOQB293-05	Moth 293.01LZ	617[0n]	KJ169277	-		Biodiversity Institute of Ontario	*Hippotion velox*		Queensland
LOQB292-05	Moth 292.01LZ	575[0n]	KJ168757	-		Biodiversity Institute of Ontario	*Hippotion velox*		Queensland
LOQTC808-08	gvc8453-1L	658[0n]	KJ168713	-		Research Collection of Graeme V. Cocks	*Hippotion velox*		Queensland
SPHJT158-12	BC-LTM-179	658[0n]	KJ168773	-		Research Collection of Max S. Moulds	*Hopliocnema brachycera*		Western Australia
ANICC173-08	ANIC Gen No. 003336	609[0n]	KJ169261	-		Australian National Insect Collection	*Hopliocnema brachycera*		Northern Territory
SPHJT157-12	BC-LTM-178	658[0n]	KJ169237	-		Research Collection of Max S. Moulds	*Hopliocnema brachycera*		Western Australia
SPHJT144-11	BC-LTM-166	658[0n]	KJ169007	-		Research Collection of Max S. Moulds	*Hopliocnema brachycera*		Northern Territory
SOWA731-06	BC-Hax0724	623[7n]	KJ169111	-		Research Collection of Jean Haxaire	*Hopliocnema brachycera*		Western Australia

Process ID	Sample ID	COI-5P	GB Acc. COI	28S-D2	GB Acc. 28S	Institution Storing	Species	Types	Origin
ANICK403-10	10ANIC-07400	609[0n]	HQ950494	-		Australian National Insect Collection	*Hopliocnema brachycera*		South Australia
ANICK405-10	10ANIC-07402	658[0n]	HQ950496	-		Australian National Insect Collection	*Hopliocnema brachycera*		Western Australia
ANICK404-10	10ANIC-07401	658[0n]	HQ950495	-		Australian National Insect Collection	*Hopliocnema brachycera*		New South Wales
SPTOL205-07	MJM-96-0232	658[0n]	JN678042	-		University of Maryland	*Hopliocnema brachycera*		Northern Territory
ANICK402-10	10ANIC-07399	658[0n]	HQ950493	-		Australian National Insect Collection	*Hopliocnema brachycera*		Victoria
SPHJT143-11	BC-LTM-165	603[0n]	KJ169131	-		Research Collection of Max S. Moulds	*Hopliocnema brachycera*		New South Wales
SPTOL206-07	MJM-96-0233	609[0n]	KJ168704	-		University of Maryland	*Hopliocnema brachycera*		Northern Territory
ANICK406-10	10ANIC-07403	658[0n]	HQ950497	-		Australian National Insect Collection	*Hopliocnema brachycera*		South Australia
SPHJT155-12	BC-LTM-176	658[0n]	KJ168845	-		Research Collection of Max S. Moulds	*Hopliocnema lacunosa*	Paratype	Western Australia
SPHJT153-12	BC-LTM-174	658[0n]	KJ169002	-		Research Collection of Max S. Moulds	*Hopliocnema lacunosa*	Holotype	Western Australia
ANICK407-10	10ANIC-07404	658[0n]	HQ950498	-		Australian National Insect Collection	*Hopliocnema lacunosa*	Paratype	Western Australia
SPHJT154-12	BC-LTM-175	658[0n]	KJ169151	-		Research Collection of Max S. Moulds	*Hopliocnema lacunosa*	Paratype	Western Australia
GWORO558-09	BC ZSM Lep 26354	658[0n]	HM422804	-		Bavarian State Collection of Zoology	*Hopliocnema lacunosa*	Paratype	Western Australia
GWORN199-09	BC ZSM Lep 18227	658[0n]	HQ957320	-		Bavarian State Collection of Zoology	*Hopliocnema ochra*	Paratype	Western Australia
SPHJT156-12	BC-LTM-177	658[0n]	KJ169335	-		Research Collection of Max S. Moulds	*Hopliocnema ochra*	Holotype	Western Australia
GWORO577-09	BC ZSM Lep 26373	658[0n]	GU688168	-		Bavarian State Collection of Zoology	*Hopliocnema ochra*	Paratype	Western Australia
GWORO578-09	BC ZSM Lep 26374	658[0n]	GU688165	-		Bavarian State Collection of Zoology	*Hopliocnema ochra*		Western Australia
GWORN198-09	BC ZSM Lep 18226	658[0n]	HQ957319	-		Bavarian State Collection of Zoology	*Hopliocnema ochra*	Paratype	Western Australia
SPHJT159-12	BC-LTM-180	658[0n]	KJ169224	-		Research Collection of Max S. Moulds	*Hopliocnema ochra*	Paratype	Western Australia
LOQTB047-07	gvc6813-1L	646[0n]	KJ169047	-		Biodiversity Institute of Ontario	*Hyles livornicoides*		Queensland
LOQTB085-07	gvc6846-1L	658[0n]	KJ168626	-		Biodiversity Institute of Ontario	*Hyles livornicoides*		Queensland
LNSWB105-05	05-NSW-01045	658[0n]	JN678060	-		New South Wales Department of Primary Industries	*Hyles livornicoides*		New South Wales
SPUEB391-07	BC-EMEM1331	658[0n]	KJ168722	-		Entomologisches Museum Eitschberger	*Hyles livornicoides*		Queensland
GWORO526-09	BC ZSM Lep 26322	658[0n]	GU688604	-		Bavarian State Collection of Zoology	*Hyles livornicoides*		Western Australia
AMWW213-12	K292498	658[0n]	KJ168972	-		Australian Museum, Sydney	*Hyles livornicoides*		New South Wales
LNSWC315-06	06-NSW-00315	658[0n]	KJ168711	-		Biodiversity Institute of Ontario	*Hyles livornicoides*		New South Wales
LOQT940-07	gvc6650-1L	658[0n]	KJ169248	-		Biodiversity Institute of Ontario	*Hyles livornicoides*		Queensland
GWORN195-09	BC ZSM Lep 18223	658[0n]	GU704663	-		Bavarian State Collection of Zoology	*Hyles livornicoides*		Western Australia
LOQT005-06	2006-LOQT-005	657[0n]	KJ168688	-		Biodiversity Institute of Ontario	*Hyles livornicoides*		Queensland
AMWW210-12	K292495	658[0n]	KJ168937	-		Australian Museum, Sydney	*Hyles livornicoides*		New South Wales
LNSWB107-05	05-NSW-01047	658[0n]	KJ169333	-		New South Wales Department of Primary Industries	*Hyles livornicoides*		New South Wales
AMWW170-12	K287974	658[0n]	KJ169143	-		Australian Museum, Sydney	*Hyles livornicoides*		New South Wales
LOQT840-07	gvc6556-1L	621[0n]	KJ168958	-		Biodiversity Institute of Ontario	*Hyles livornicoides*		Queensland
LNSWB108-05	05-NSW-01048	608[0n]	KJ168667	-		New South Wales Department of Primary Industries	*Hyles livornicoides*		New South Wales
AMWW211-12	K292496	658[0n]	KJ169193	-		Australian Museum, Sydney	*Hyles livornicoides*		New South Wales
LOQTC660-08	gvc8302-1L	658[0n]	KJ168699	-		Research Collection of Graeme V. Cocks	*Hyles livornicoides*		Queensland

Process ID	Sample ID	COI-5P	GB Acc. COI	28S-D2	GB Acc. 28S	Institution Storing	Species	Types	Origin
LOQTC628-07	gvc8269-1L	658[0n]	KJ168926	-		Biodiversity Institute of Ontario	*Hyles livornicoides*		Queensland
AMWW039-11	K290779	658[0n]	KJ168646	-		Australian Museum, Sydney	*Hyles livornicoides*		New South Wales
GWORA168-08	BC ZSM Lep 10540	547[0n]	KJ169048	-		Bavarian State Collection of Zoology	*Hyles livornicoides*		Western Australia
AMWW011-11	K290751	658[0n]	KJ168705	-		Australian Museum, Sydney	*Hyles livornicoides*		New South Wales
LOQTC767-08	gvc8412-1L	658[0n]	KJ169187	-		Biodiversity Institute of Ontario	*Hyles livornicoides*		Queensland
GWORA177-08	BC ZSM Lep 10549	625[0n]	KJ168863	-		Bavarian State Collection of Zoology	*Hyles livornicoides*		Western Australia
AMWW212-12	K292497	658[0n]	KJ169203	-		Australian Museum, Sydney	*Hyles livornicoides*		New South Wales
GWORN201-09	BC ZSM Lep 18229	658[0n]	GU704662	-		Bavarian State Collection of Zoology	*Hyles livornicoides*		Western Australia
LOQT212-06	2006-LOQT-212	658[0n]	KJ168635	-		Biodiversity Institute of Ontario	*Hyles livornicoides*		Queensland
ANICB365-06	ANIC Gen No. 000212	658[0n]	KJ169076	-		Australian National Insect Collection	*Hyles livornicoides*		Queensland
GWORA176-08	BC ZSM Lep 10548	551[1n]	KJ169103	-		Bavarian State Collection of Zoology	*Hyles livornicoides*		Western Australia
AMWW209-12	K292494	658[0n]	KJ168896	-		Australian Museum, Sydney	*Hyles livornicoides*		New South Wales
LOQTB048-07	gvc6814-1L	658[0n]	KJ168619	-		Biodiversity Institute of Ontario	*Hyles livornicoides*		Queensland
LOQT841-07	gvc6557-1L	656[0n]	KJ169344	-		Biodiversity Institute of Ontario	*Hyles livornicoides*		Queensland
LOQT408-06	gvc5635-1L	658[0n]	KJ169368	-		Biodiversity Institute of Ontario	*Hyles livornicoides*		Queensland
LOQTI651-12	gvc2677-1L	658[0n]	KJ169345	-		Biodiversity Institute of Ontario	*Hyles livornicoides*		Queensland
AMWW208-12	K292493	658[0n]	KJ168852	-		Australian Museum, Sydney	*Hyles livornicoides*		New South Wales
LNSWB104-05	05-NSW-01044	658[0n]	KJ168976	-		New South Wales Department of Primary Industries	*Hyles livornicoides*		New South Wales
LNSWB103-05	05-NSW-01043	614[0n]	KJ169331	-		New South Wales Department of Primary Industries	*Hyles livornicoides*		New South Wales
LOQTC659-08	gvc8301-1L	658[0n]	KJ168781	-		Research Collection of Graeme V. Cocks	*Hyles livornicoides*		Queensland
LNSWB106-05	05-NSW-01046	658[0n]	KJ168885	-		New South Wales Department of Primary Industries	*Hyles livornicoides*		New South Wales
AMWW214-12	K292499	658[0n]	KJ169247	-		Australian Museum, Sydney	*Hyles livornicoides*		New South Wales
ANICC043-08	ANIC Gen No. 003206	658[0n]	KJ168774	-		Australian National Insect Collection	*Imber tropicus*		Queensland
GWORA030-08	BC ZSM Lep 10402	657[0n]	KJ169343	-		Bavarian State Collection of Zoology	*Imber tropicus*		Western Australia
ANICC023-08	ANIC Gen No. 003186	609[0n]	KJ169311	-		Australian National Insect Collection	*Imber tropicus*		Queensland
SOWC190-06	BC-Mel0021	658[0n]	KJ168825	-		Research Collection of Tomas Melichar	*Imber tropicus*		Queensland
GWORA031-08	BC ZSM Lep 10403	658[0n]	KJ169334	-		Bavarian State Collection of Zoology	*Imber tropicus*		Western Australia
GWORA027-08	BC ZSM Lep 10399	658[0n]	KJ168891	-		Bavarian State Collection of Zoology	*Imber tropicus*		Western Australia
SOWA197-06	BC-Hax0197	658[0n]	JN678074	-		Research Collection of Jean Haxaire	*Imber tropicus*		Queensland
ANICC077-08	ANIC Gen No. 003240	609[0n]	KJ169279	-		Australian National Insect Collection	*Leucomonia bethia*		Northern Territory
ANICC078-08	ANIC Gen No. 003241	658[0n]	JN678099	-		Australian National Insect Collection	*Leucomonia bethia*		Western Australia
ANICC080-08	ANIC Gen No. 003243	658[0n]	KJ168623	-		Australian National Insect Collection	*Leucomonia bethia*		Western Australia
SOWD283-06	BC-Hax3182	607[0n]	KJ168647	-		Research Collection of Jean Haxaire	*Leucomonia bethia*		Queensland
GWORA135-08	BC ZSM Lep 10507	658[0n]	KJ168790	-		Bavarian State Collection of Zoology	*Leucomonia bethia*		Western Australia
ANICC079-08	ANIC Gen No. 003242	658[0n]	KJ169196	-		Australian National Insect Collection	*Leucomonia bethia*		Western Australia
SPHJT097-10	BC-LTM-121	658[0n]	HQ975017	-		Research Collection of David A. Lane	*Macroglossum alcedo*		Queensland

Process ID	Sample ID	COI-5P	GB Acc. COI	28S-D2	GB Acc. 28S	Institution Storing	Species	Types	Origin
SPHJT008-09	BC-JT-23M	658[0n]	HM384234	-		Research Collection of James A. Tuttle	*Macroglossum alcedo*		Queensland
ANICC053-08	ANIC Gen No. 003216	263[0n]	KJ169088	-		Australian National Insect Collection	*Macroglossum corythus*		Queensland
SPHJT099-10	BC-LTM-123	658[0n]	HQ975019	-		Research Collection of David A. Lane	*Macroglossum dohertyi doddi*		Queensland
ANICH125-10	10ANIC-04122	658[0n]	HQ923135	-		Australian National Insect Collection	*Macroglossum dohertyi doddi*		Queensland
SPHJT098-10	BC-LTM-122	658[0n]	HQ975018	-		Research Collection of David A. Lane	*Macroglossum dohertyi doddi*		Queensland
ANICH127-10	10ANIC-04124	609[0n]	HQ923136	-		Australian National Insect Collection	*Macroglossum dohertyi doddi*		Queensland
ANICC052-08	ANIC Gen No. 003215	658[0n]	JN678136	-		Australian National Insect Collection	*Macroglossum dohertyi doddi*		Queensland
LOQT216-06	2006-LOQT-216	658[0n]	KJ169010	-		Biodiversity Institute of Ontario	*Macroglossum hirundo errans*		Queensland
SPTMA574-09	BC-Mel 0652	607[1n]	KJ168604	-		Research Collection of Tomas Melichar	*Macroglossum hirundo errans*		Queensland
LOQ340-04	04HBL004340	533[0n]	KJ169056	-		Biodiversity Institute of Ontario	*Macroglossum hirundo errans*		Queensland
LOQTE399-09	gvc12453-1L	658[0n]	KJ168778	-		Research Collection of Graeme V. Cocks	*Macroglossum hirundo errans*		Queensland
ANICC166-08	ANIC Gen No. 003329	609[0n]	KJ168789	-		Australian National Insect Collection	*Macroglossum hirundo errans*		Queensland
LOQTD851-09	gvc10646-1L	658[0n]	KJ168806	-		Research Collection of Graeme V. Cocks	*Macroglossum hirundo errans*		Queensland
LOQT003-06	2006-LOQT-003	579[3n]	KJ168665	-		Biodiversity Institute of Ontario	*Macroglossum hirundo errans*		Queensland
LOQ339-04	04HBL004339	592[0n]	KJ169238	-		Biodiversity Institute of Ontario	*Macroglossum hirundo errans*		Queensland
LOLI143-08	08-QLDLI-143	658[0n]	KJ169213	-		Biodiversity Institute of Ontario	*Macroglossum hirundo errans*		Queensland
LLISA595-06	06-NSWL-00595	658[0n]	JN678144	-		Biodiversity Institute of Ontario	*Macroglossum hirundo errans*		New South Wales
ANICC022-08	ANIC Gen No. 003185	583[0n]	KJ168807	-		Australian National Insect Collection	*Macroglossum hirundo errans*		Queensland
ANICC062-08	ANIC Gen No. 003225	609[0n]	JN678146	-		Australian National Insect Collection	*Macroglossum joannisi*		Queensland
LNSWE002-06	06-NSWE-00002	594[0n]	KJ169148	-		Biodiversity Institute of Ontario	*Macroglossum micacea*		New South Wales
LOQT015-06	2006-LOQT-015	600[0n]	KJ169091	-		Biodiversity Institute of Ontario	*Macroglossum micacea*		Queensland
ANICC012-08	ANIC Gen No. 003175	512[0n]	KJ169236	-		Australian National Insect Collection	*Macroglossum micacea*		Queensland
LOQB079-05	Moth 079.03LZ	602[0n]	KJ168765	-		Biodiversity Institute of Ontario	*Macroglossum micacea*		Queensland
ANIC324-06	ANIC Gen No. 000516	595[0n]	KJ168676	-		Australian National Insect Collection	*Macroglossum micacea*		Queensland
LOQT222-06	2006-LOQT-222	615[0n]	JN678150	-		Biodiversity Institute of Ontario	*Macroglossum micacea*		Queensland
GWORY460-10	BC EF Lep 03414	580[0n]	HM913601	-		Research Collection of Egbert Friedrich	*Macroglossum nubilum*		Queensland
ANICC059-08	ANIC Gen No. 003222	553[92n]	KJ169377	-		Australian National Insect Collection	*Macroglossum nubilum*		Queensland
ANICC060-08	ANIC Gen No. 003223	649[9n]	JN678157	-		Australian National Insect Collection	*Macroglossum nubilum*		Queensland
ANICC063-08	ANIC Gen No. 003226	630[0n]	KJ168791	-		Australian National Insect Collection	*Macroglossum prometheus lineata*		Queensland
ANICC065-08	ANIC Gen No. 003228	625[0n]	KJ169090	-		Australian National Insect Collection	*Macroglossum prometheus lineata*		Queensland
SPHJT079-10	BC-LTM-103	658[0n]	HQ975002	-		Research Collection of Max S. Moulds	*Macroglossum prometheus lineata*		Queensland
SPHJT078-10	BC-LTM-102	521[0n]	HQ975001	-		Research Collection of Max S. Moulds	*Macroglossum prometheus lineata*		Queensland
ANICC027-08	ANIC Gen No. 003190	502[0n]	KJ168887	-		Australian National Insect Collection	*Macroglossum rectans*		Queensland
ANICC026-08	ANIC Gen No. 003189	581[0n]	KJ168719	-		Australian National Insect Collection	*Macroglossum rectans*		Queensland
ANICC025-08	ANIC Gen No. 003188	597[0n]	JN678161	-		Australian National Insect Collection	*Macroglossum rectans*		Queensland
ANICC024-08	ANIC Gen No. 003187	582[2n]	KJ169150	-		Australian National Insect Collection	*Macroglossum rectans*		Queensland

Process ID	Sample ID	COI-5P	GB Acc. COI	28S-D2	GB Acc. 28S	Institution Storing	Species	Types	Origin
SPTMA383-07	BC-Mel 0461	638[0n]	KJ168697	-		Research Collection of Tomas Melichar	*Macroglossum rectans*		
SPHJT077-10	BC-LTM-101	658[0n]	HQ975000	-		Research Collection of Max S. Moulds	*Macroglossum tenebrosa*		Queensland
LTOL254-07	JP-05-0749	658[0n]	KJ169263	597[0n]	KJ169421	University of Maryland	*Macroglossum tenebrosa*		Queensland
SPHJT076-10	BC-LTM-100	658[0n]	HQ974999	-		Research Collection of Max S. Moulds	*Macroglossum tenebrosa*		Queensland
ANICC167-08	ANIC Gen No. 003330	658[0n]	KJ169353	-		Australian National Insect Collection	*Macroglossum tenebrosa*		Queensland
ANICC049-08	ANIC Gen No. 003212	293[1n]	KJ168954	-		Australian National Insect Collection	*Macroglossum troglodytus*		Queensland
GWORY461-10	BC EF Lep 03415	658[0n]	HM913602	-		Research Collection of Egbert Friedrich	*Macroglossum vacillans*		Northern Territory
GWORA085-08	BC ZSM Lep 10457	623[0n]	KJ169106	-		Bavarian State Collection of Zoology	*Macroglossum vacillans*		Western Australia
SOWD716-06	BC-Hax3615	607[0n]	KJ169347	-		Research Collection of Jean Haxaire	*Macroglossum vacillans*		Queensland
SPTMA586-09	BC-Mel 0664	640[0n]	KJ169000	-		Research Collection of Tomas Melichar	*Macroglossum vacillans*		Queensland
ANICC075-08	ANIC Gen No. 003238	609[0n]	KJ168888	-		Australian National Insect Collection	*Macroglossum vacillans*		Western Australia
GWORI497-09	BC ZSM Lep 13245	658[0n]	KJ169008	-		Bavarian State Collection of Zoology	*Macroglossum vacillans*		Northern Territory
GWORA098-08	BC ZSM Lep 10470	619[1n]	KJ168914	-		Bavarian State Collection of Zoology	*Macroglossum vacillans*		Northern Territory
ANICC014-08	ANIC Gen No. 003177	609[0n]	KJ169297	-		Australian National Insect Collection	*Macroglossum vacillans*		Queensland
SOWD717-06	BC-Hax3616	658[0n]	JN678173	-		Research Collection of Jean Haxaire	*Macroglossum vacillans*		Queensland
GWORA198-08	BC ZSM Lep 10570	658[0n]	KJ168818	-		Bavarian State Collection of Zoology	*Macroglossum vacillans*		Northern Territory
GWOR1250-07	BC ZSM Lep 02002	658[0n]	KJ168868	-		Bavarian State Collection of Zoology	*Macroglossum vacillans*		Queensland
GWORA092-08	BC ZSM Lep 10464	645[0n]	KJ168744	-		Bavarian State Collection of Zoology	*Macroglossum vacillans*		Northern Territory
GWORA161-08	BC ZSM Lep 10533	580[0n]	KJ168687	-		Bavarian State Collection of Zoology	*Macroglossum vacillans*		Western Australia
ANICC008-08	ANIC Gen No. 003171	621[0n]	KJ168663	-		Australian National Insect Collection	*Nephele hespera*		Queensland
LOQT001-06	2006-LOQT-001	598[0n]	KJ169340	-		Biodiversity Institute of Ontario	*Nephele subvaria*		Queensland
LOQTE270-09	gvc11875-1L	658[0n]	GU669915	-		Research Collection of Graeme V. Cocks	*Nephele subvaria*		Queensland
ANICC035-08	ANIC Gen No. 003198	609[0n]	KJ168785	-		Australian National Insect Collection	*Nephele subvaria*		Queensland
GWORI541-09	BC ZSM Lep 13289	658[0n]	KJ168664	-		Bavarian State Collection of Zoology	*Nephele subvaria*		Northern Territory
ANICC047-08	ANIC Gen No. 003210	658[0n]	KJ168685	-		Australian National Insect Collection	*Nephele subvaria*		Northern Territory
SPTMB094-09	BC-Mel 1106	651[0n]	GU704485	-		Research Collection of Tomas Melichar	*Nephele subvaria*		Western Australia
ANICC015-08	ANIC Gen No. 003178	609[0n]	KJ168897	-		Australian National Insect Collection	*Nephele subvaria*		Queensland
GWORY467-10	BC EF Lep 03421	658[0n]	HM913607	-		Research Collection of Egbert Friedrich	*Nephele subvaria*		Queensland
SOWD499-06	BC-Hax3398	407[0n]	KJ169225	-		Research Collection of Jean Haxaire	*Nephele subvaria*		Queensland
ANICC048-08	ANIC Gen No. 003211	658[0n]	KJ169400	-		Australian National Insect Collection	*Nephele subvaria*		Northern Territory
ANICC036-08	ANIC Gen No. 003199	609[0n]	KJ169374	-		Australian National Insect Collection	*Nephele subvaria*		Queensland
LOQTE269-09	gvc11874-1L	658[0n]	GU669914	-		Research Collection of Graeme V. Cocks	*Nephele subvaria*		Queensland
ANICC010-08	ANIC Gen No. 003173	609[0n]	KJ169242	-		Australian National Insect Collection	*Nephele subvaria*		Western Australia
ANICC016-08	ANIC Gen No. 003179	658[0n]	JN678305	-		Australian National Insect Collection	*Nephele subvaria*		Queensland
LOQTB174-07	gvc6936-1L	658[0n]	KJ168941	-		Biodiversity Institute of Ontario	*Nephele subvaria*		Queensland
ANICC056-08	ANIC Gen No. 003219	551[2n]	KJ168725	-		Australian National Insect Collection	*Pseudoangonyx excellens*		Queensland

Process ID	Sample ID	COI-5P	GB Acc. COI	28S-D2	GB Acc. 28S	Institution Storing	Species	Types	Origin
ANICC044-08	ANIC Gen No. 003207	655[3n]	JN677748	-		Australian National Insect Collection	*Pseudoangonyx excellens*		Queensland
ANICC072-08	ANIC Gen No. 003235	658[0n]	KJ169295	-		Australian National Insect Collection	*Psilogramma argos*		Northern Territory
SPHJT100-10	BC-LTM-124	658[0n]	HQ975020	-		Research Collection of David A. Lane	*Psilogramma argos*		Northern Territory
SPTMB093-09	BC-Mel 1105	658[0n]	GU704487	-		Research Collection of Tomas Melichar	*Psilogramma argos*		Northern Territory
NSWBB1269-08	07-NSWBB-1269	658[0n]	KJ169021	-		Biodiversity Institute of Ontario	*Psilogramma casuarinae*		New South Wales
NSWHJ076-10	09-NSWHH-0089	658[0n]	HM380429	-		Biodiversity Institute of Ontario	*Psilogramma casuarinae*		New South Wales
LNSWE010-06	06-NSWE-00010	588[2n]	KJ169172	-		Biodiversity Institute of Ontario	*Psilogramma casuarinae*		New South Wales
SPTMA298-07	BC-Mel 0376	658[0n]	KJ168599	-		Research Collection of Tomas Melichar	*Psilogramma casuarinae*		Queensland
AMWW009-11	K290749	658[0n]	KJ168792	-		Australian Museum, Sydney	*Psilogramma casuarinae*		New South Wales
SPHJT093-10	BC-LTM-117	658[0n]	HQ975014	-		Research Collection of Max S. Moulds	*Psilogramma casuarinae*		Queensland
IMLQ182-07	IM06-0728	658[0n]	KJ168939	-		Biodiversity Institute of Ontario	*Psilogramma casuarinae*		Queensland
LNSWE085-06	06-NSWE-00085	657[0n]	KJ169395	-		Biodiversity Institute of Ontario	*Psilogramma casuarinae*		New South Wales
LOQTE795-10	gvc14173-1L	658[0n]	HQ572338	-		Biodiversity Institute of Ontario	*Psilogramma casuarinae*		Queensland
SPTMA296-07	BC-Mel 0374	658[0n]	KJ169102	-		Research Collection of Tomas Melichar	*Psilogramma casuarinae*		Queensland
SPTMA300-07	BC-Mel 0378	658[0n]	KJ168615	-		Research Collection of Tomas Melichar	*Psilogramma casuarinae*		Queensland
AMWW247-12	K292532	658[0n]	KJ168745	-		Australian Museum, Sydney	*Psilogramma casuarinae*		New South Wales
SPHJT047-10	BC-JT-0028	407[0n]	KJ168800	-		Biodiversity Institute of Ontario	*Psilogramma casuarinae*		Victoria
NSWHJ088-10	09-NSWHH-0101	658[0n]	HM380441	-		Biodiversity Institute of Ontario	*Psilogramma casuarinae*		New South Wales
NSWHM027-11	BIOUG00851-H08	658[0n]	JN284551	-		Biodiversity Institute of Ontario	*Psilogramma casuarinae*		New South Wales
SPHJT116-10	BC-LTM-140	658[0n]	HQ580420	-		Research Collection of David A. Lane	*Psilogramma casuarinae*		Queensland
LNSWE110-06	06-NSWE-00110	582[0n]	KJ169163	-		Biodiversity Institute of Ontario	*Psilogramma casuarinae*		New South Wales
SPTMA299-07	BC-Mel 0377	658[0n]	KJ169116	-		Research Collection of Tomas Melichar	*Psilogramma casuarinae*		Queensland
LNSWE044-06	06-NSWE-00044	658[0n]	KJ168654	-		Biodiversity Institute of Ontario	*Psilogramma casuarinae*		New South Wales
SPTMA290-07	BC-Mel 0368	658[0n]	KJ169339	-		Research Collection of Tomas Melichar	*Psilogramma casuarinae*		Queensland
LOQTB025-07	gvc6799-1L	658[0n]	KJ168693	-		Biodiversity Institute of Ontario	*Psilogramma casuarinae*		Queensland
NSWHJ042-10	09-NSWHH-0055	658[0n]	HM380396	-		Biodiversity Institute of Ontario	*Psilogramma casuarinae*		New South Wales
SPHJT095-10	BC-LTM-119	658[0n]	HQ975016	-		Research Collection of Max S. Moulds	*Psilogramma casuarinae*		Queensland
SPHJT113-10	BC-LTM-137	658[0n]	HQ580419	-		Research Collection of Tom Sleep	*Psilogramma casuarinae*		Queensland
SPHJT094-10	BC-LTM-118	658[0n]	HQ975015	-		Research Collection of Max S. Moulds	*Psilogramma casuarinae*		Queensland
AMWW027-11	K290767	658[0n]	KJ168902	-		Australian Museum, Sydney	*Psilogramma casuarinae*		New South Wales
SPHJT092-10	BC-LTM-116	630[0n]	HQ975013	-		Research Collection of Max S. Moulds	*Psilogramma casuarinae*		Queensland
IMLQ239-07	IM07-0193	658[0n]	KJ169109	-		Biodiversity Institute of Ontario	*Psilogramma casuarinae*		Queensland
SOWD151-06	BC-Hax3050	607[0n]	KJ168847	-		Research Collection of Jean Haxaire	*Psilogramma casuarinae*		Queensland
SPHJT115-10	BC-LTM-139	658[0n]	KJ169303	-		Research Collection of Max S. Moulds	*Psilogramma casuarinae*		New South Wales
NSWBB1203-08	07-NSWBB-1203	658[0n]	KJ169098	-		Biodiversity Institute of Ontario	*Psilogramma casuarinae*		New South Wales
SPHJT117-10	BC-LTM-141	658[0n]	KJ169158	-		Research Collection of David A. Lane	*Psilogramma exigua*		Queensland

Process ID	Sample ID	COI-5P	GB Acc. COI	28S-D2	GB Acc. 28S	Institution Storing	Species	Types	Origin
SPHJT119-10	BC-LTM-143	658[0n]	HQ580422	-		Research Collection of David A. Lane	*Psilogramma exigua*	Paratype	Northern Territory
SPTMB089-09	BC-Mel 1101	658[0n]	GU704491	-		Research Collection of Tomas Melichar	*Psilogramma exigua*		Northern Territory
SPHJT142-10	BC-LTM-164	658[0n]	KJ168993	-		Research Collection of Max S. Moulds	*Psilogramma exigua*		Northern Territory
GWORI484-09	BC ZSM Lep 13232	658[0n]	KJ168812	-		Bavarian State Collection of Zoology	*Psilogramma exigua*		Northern Territory
SPHJT118-10	BC-LTM-142	658[0n]	HQ580421	-		Research Collection of David A. Lane	*Psilogramma exigua*	Paratype	Northern Territory
LOQTB045-07	gvc6811-1L	642[1n]	KJ169308	-		Biodiversity Institute of Ontario	*Psilogramma exigua*		Queensland
SPTMC624-12	BC-Mel2625	630[0n]	KJ169052	-		Research Collection of Tomas Melichar	*Psilogramma exigua*		Northern Territory
SPTMB088-09	BC-Mel 1100	658[0n]	GU704490	-		Research Collection of Tomas Melichar	*Psilogramma exigua*		Northern Territory
SPTMC627-12	BC-Mel2628	658[0n]	KJ168608	-		Research Collection of Tomas Melichar	*Psilogramma exigua*		Northern Territory
SPHJT120-10	BC-LTM-144	658[0n]	HQ580423	-		Research Collection of David A. Lane	*Psilogramma exigua*		Northern Territory
SPTMC623-12	BC-Mel2624	658[0n]	KJ168947	-		Research Collection of Tomas Melichar	*Psilogramma exigua*		Northern Territory
SPTMB092-09	BC-Mel 1104	651[0n]	GU704486	-		Research Collection of Tomas Melichar	*Psilogramma exigua*		Northern Territory
SPTMB090-09	BC-Mel 1102	658[0n]	GU704488	-		Research Collection of Tomas Melichar	*Psilogramma exigua*		Northern Territory
SPTMB091-09	BC-Mel 1103	658[0n]	GU704489	-		Research Collection of Tomas Melichar	*Psilogramma exigua*		Northern Territory
SPTMC626-12	BC-Mel2627	658[0n]	KJ168668	-		Research Collection of Tomas Melichar	*Psilogramma exigua*		Northern Territory
SPUEB588-09	BC-EMEM1528	658[0n]	GU704630	-		Entomologisches Museum Eitschberger	*Psilogramma gloriosa*	Holotype	Queensland
SPZSM052-08	BC-ZSMRR0052	462[196n]	KJ168651	-		Bavarian State Collection of Zoology	*Psilogramma hausmanni*	Holotype	New South Wales
SPUEB591-09	BC-EMEM1531	658[0n]	GU704627	-		Entomologisches Museum Eitschberger	*Psilogramma koalae*	Holotype	Queensland
LNSWE042-06	06-NSWE-00042	658[0n]	KJ168795	-		Biodiversity Institute of Ontario	*Psilogramma maxmouldsi*		New South Wales
SPUEB589-09	BC-EMEM1529	658[0n]	GU704629	-		Entomologisches Museum Eitschberger	*Psilogramma maxmouldsi*	Holotype	Queensland
LNSWE071-06	06-NSWE-00071	658[0n]	KJ169104	-		Biodiversity Institute of Ontario	*Psilogramma maxmouldsi*		New South Wales
LNSWE067-06	06-NSWE-00067	651[0n]	KJ169320	-		Biodiversity Institute of Ontario	*Psilogramma maxmouldsi*		New South Wales
LNSWE038-06	06-NSWE-00038	658[0n]	KJ168760	-		Biodiversity Institute of Ontario	*Psilogramma maxmouldsi*		New South Wales
IMLQ157-07	IM06-0592	658[0n]	KJ169307	-		Biodiversity Institute of Ontario	*Psilogramma maxmouldsi*		Queensland
NSWHM2021-11	BIOUG00961-A04	658[0n]	JN284553	-		Biodiversity Institute of Ontario	*Psilogramma maxmouldsi*		New South Wales
NSWHJ006-10	09-NSWHH-0019	658[0n]	HM380360	-		Biodiversity Institute of Ontario	*Psilogramma maxmouldsi*		New South Wales
SPHJT001-09	BC-JT-24M	631[0n]	HM384228	-		Research Collection of James A. Tuttle	*Psilogramma maxmouldsi*		Queensland
LOQTD098-08	gvc8697-1L	658[0n]	KJ169290	-		Research Collection of Graeme V. Cocks	*Psilogramma menephron*		Queensland
GWORC230-07	BC ZSM Lep 02580	657[0n]	KJ169244	-		Bavarian State Collection of Zoology	*Psilogramma menephron*		Queensland
SPHJT137-10	BC-LTM-161	658[0n]	HQ580433	-		Research Collection of David A. Lane	*Psilogramma menephron*		Queensland
LLISA224-06	06-NSWL-00224	658[0n]	JN678461	-		Biodiversity Institute of Ontario	*Psilogramma menephron*		New South Wales
LLISA598-06	06-NSWL-00598	658[0n]	KJ168742	-		Biodiversity Institute of Ontario	*Psilogramma menephron*		New South Wales
NSWBB1145-08	07-NSWBB-1145	658[0n]	KJ169317	-		Biodiversity Institute of Ontario	*Psilogramma menephron*		New South Wales
LOQB330-05	Moth 014.03CC	658[0n]	KJ168899	-		Biodiversity Institute of Ontario	*Psilogramma menephron*		Queensland
LOQTD099-08	gvc8698-1L	658[0n]	KJ169246	-		Biodiversity Institute of Ontario	*Psilogramma menephron*		Queensland
SPHJT003-09	BC-JT-20M	658[0n]	HM384230	-		Research Collection of James A. Tuttle	*Psilogramma menephron*		Queensland

Process ID	Sample ID	COI-5P	GB Acc. COI	28S-D2	GB Acc. 28S	Institution Storing	Species	Types	Origin
GWOR1242-07	BC ZSM Lep 01994	657[0n]	KJ169074	-		Bavarian State Collection of Zoology	*Psilogramma menephron*		Queensland
GWORB850-07	BC ZSM Lep 01320	632[0n]	KJ168720	-		Bavarian State Collection of Zoology	*Psilogramma menephron*		Queensland
GWORC231-07	BC ZSM Lep 02581	655[0n]	KJ169129	-		Bavarian State Collection of Zoology	*Psilogramma menephron*		Queensland
LLISA222-06	06-NSWL-00222	658[0n]	KJ169139	-		Biodiversity Institute of Ontario	*Psilogramma menephron*		New South Wales
LLISA219-06	06-NSWL-00219	658[0n]	KJ168684	-		Biodiversity Institute of Ontario	*Psilogramma menephron*		New South Wales
GWORB867-07	BC ZSM Lep 01337	632[0n]	KJ168695	-		Bavarian State Collection of Zoology	*Psilogramma menephron*		Queensland
LOQTI348-10	gvc15912-1L	658[0n]	KJ168709	-		Biodiversity Institute of Ontario	*Psilogramma menephron*		Queensland
NSWHJ020-10	09-NSWHH-0033	658[0n]	HM380374	-		Biodiversity Institute of Ontario	*Psilogramma menephron*		New South Wales
LLISA600-06	06-NSWL-00600	658[0n]	KJ169285	-		Biodiversity Institute of Ontario	*Psilogramma menephron*		New South Wales
LOQTE796-10	gvc14174-1L	658[0n]	HQ572339	-		Biodiversity Institute of Ontario	*Psilogramma menephron*		Queensland
IMLQ184-07	IM06-0744	658[0n]	KJ168672	-		Biodiversity Institute of Ontario	*Psilogramma menephron*		Queensland
GWORB3265-08	BC ZSM Lep 05803	658[0n]	KJ168842	-		Bavarian State Collection of Zoology	*Psilogramma menephron*		Queensland
LTOL252-07	JP-05-0753	658[0n]	KJ168851	-		University of Maryland	*Psilogramma menephron*		Queensland
LLISA218-06	06-NSWL-00218	658[0n]	KJ168925	-		Biodiversity Institute of Ontario	*Psilogramma menephron*		New South Wales
GWORB3266-08	BC ZSM Lep 05804	658[0n]	KJ169373	-		Bavarian State Collection of Zoology	*Psilogramma menephron*		Queensland
LOQTD039-08	gvc8636-1L	658[0n]	KJ169390	-		Research Collection of Graeme V. Cocks	*Psilogramma menephron*		Queensland
LOQTD097-08	gvc8696-1L	658[0n]	KJ169125	-		Research Collection of Graeme V. Cocks	*Psilogramma menephron*		Queensland
SPTMA307-07	BC-Mel 0385	645[0n]	KJ169095	-		Research Collection of Tomas Melichar	*Psilogramma menephron*		Queensland
SPRBA338-08	BC-RBP-0338	636[0n]	KJ169080	-		Research Collection of Ron Brechlin	*Psilogramma menephron*		Queensland
SPRBA337-08	BC-RBP-0337	609[0n]	KJ169272	-		Research Collection of Ron Brechlin	*Psilogramma menephron*		Queensland
IMLR1170-11	IM08-2832	658[0n]	KJ169245	-		Biodiversity Institute of Ontario	*Psilogramma menephron*		Queensland
SPHJT111-10	BC-LTM-135	658[0n]	HQ975031	-		Research Collection of David A. Lane	*Psilogramma menephron*		Queensland
GWORG362-08	BC ZSM Lep 08478	658[0n]	KJ169375	-		Bavarian State Collection of Zoology	*Psilogramma menephron*		Queensland
LLISA223-06	06-NSWL-00223	658[0n]	KJ168966	-		Biodiversity Institute of Ontario	*Psilogramma menephron*		New South Wales
GWOR1824-07	BC ZSM Lep 02388	605[0n]	KJ169278	-		Bavarian State Collection of Zoology	*Psilogramma papuensis*		Queensland
SPHJT112-10	BC-LTM-136	658[0n]	HQ975032	-		Research Collection of David A. Lane	*Psilogramma papuensis*		Queensland
GWORB868-07	BC ZSM Lep 01338	632[0n]	KJ168985	-		Bavarian State Collection of Zoology	*Psilogramma papuensis*		Queensland
GWOR1823-07	BC ZSM Lep 02387	605[0n]	KJ168945	-		Bavarian State Collection of Zoology	*Psilogramma papuensis*		Queensland
SPTMA297-07	BC-Mel 0375	658[0n]	KJ168981	-		Research Collection of Tomas Melichar	*Psilogramma papuensis*		Queensland
GWORB847-07	BC ZSM Lep 01317	606[0n]	KJ169211	-		Bavarian State Collection of Zoology	*Psilogramma papuensis*		Queensland
SPTMA304-07	BC-Mel 0382	658[0n]	KJ168653	-		Research Collection of Tomas Melichar	*Psilogramma papuensis*		Queensland
GWOR1822-07	BC ZSM Lep 02386	608[0n]	KJ169176	-		Bavarian State Collection of Zoology	*Psilogramma papuensis*		Queensland
LOQB002-05	Moth 002.01LZ	582[0n]	KJ169294	-		Biodiversity Institute of Ontario	*Psilogramma papuensis*		Queensland
GWORC280-07	BC ZSM Lep 02630	653[0n]	KJ168840	-		Bavarian State Collection of Zoology	*Psilogramma papuensis*		Queensland
SPHJT139-10	BC-LTM-163	658[0n]	HQ580434	-		Research Collection of David A. Lane	*Psilogramma papuensis*		Queensland
SPTMA303-07	BC-Mel 0381	658[0n]	KJ169134	-		Research Collection of Tomas Melichar	*Psilogramma papuensis*		Queensland

Process ID	Sample ID	COI-5P	GB Acc. COI	28S-D2	GB Acc. 28S	Institution Storing	Species	Types	Origin
SPTMA301-07	BC-Mel 0379	658[0n]	KJ169351	-		Research Collection of Tomas Melichar	*Psilogramma papuensis*		Queensland
GWORB869-07	BC ZSM Lep 01339	615[0n]	KJ169369	-		Bavarian State Collection of Zoology	*Psilogramma papuensis*		Queensland
GWOR1825-07	BC ZSM Lep 02389	605[0n]	KJ168853	-		Bavarian State Collection of Zoology	*Psilogramma papuensis*		Queensland
GWORC232-07	BC ZSM Lep 02582	655[0n]	KJ168777	-		Bavarian State Collection of Zoology	*Psilogramma papuensis*		Queensland
GWOR1820-07	BC ZSM Lep 02384	617[0n]	KJ168753	-		Bavarian State Collection of Zoology	*Psilogramma papuensis*		Queensland
GWORB3278-08	BC ZSM Lep 05816	658[0n]	KJ168782	-		Bavarian State Collection of Zoology	*Psilogramma papuensis*		Queensland
GWORG293-08	BC ZSM Lep 08409	658[0n]	KJ168768	-		Bavarian State Collection of Zoology	*Psilogramma papuensis*		Queensland
GWORD035-07	BC ZSM Lep 02667	658[0n]	KJ169271	-		Bavarian State Collection of Zoology	*Psilogramma papuensis*		Queensland
GWOR1821-07	BC ZSM Lep 02385	616[1n]	KJ168841	-		Bavarian State Collection of Zoology	*Psilogramma papuensis*		Queensland
GWORC229-07	BC ZSM Lep 02579	657[0n]	KJ168650	-		Bavarian State Collection of Zoology	*Psilogramma papuensis*		Queensland
SPTMA302-07	BC-Mel 0380	658[0n]	KJ168903	-		Research Collection of Tomas Melichar	*Psilogramma papuensis*		Queensland
GWORB848-07	BC ZSM Lep 01318	632[0n]	KJ169013	-		Bavarian State Collection of Zoology	*Psilogramma papuensis*		Queensland
SPHJT123-10	BC-LTM-147	658[0n]	HQ580425	-		Research Collection of David A. Lane	*Psilogramma penumbra*	Holotype	Northern Territory
SPHJT121-10	BC-LTM-145	658[0n]	HQ580424	-		Research Collection of David A. Lane	*Psilogramma penumbra*	Paratype	Northern Territory
ANICC076-08	ANIC Gen No. 003239	658[0n]	KJ168607	-		Australian National Insect Collection	*Psilogramma penumbra*		Northern Territory
SPHJT122-10	BC-LTM-146	621[0n]	KJ169170	-		Research Collection of David A. Lane	*Psilogramma penumbra*	Paratype	Northern Territory
SPTMC308-12	BC-Mel2309	658[0n]	KJ168969	-		Research Collection of Tomas Melichar	*Synoecha marmorata*		Queensland
LTOL925-08	AZ-06-0210	657[0n]	KJ168989	-		University of Maryland	*Synoecha marmorata*		New South Wales
ANICB362-06	ANIC Gen No. 000164	658[0n]	KJ169029	-		Australian National Insect Collection	*Synoecha marmorata*		Queensland
SPTMC309-12	BC-Mel2310	621[0n]	KJ168627	-		Research Collection of Tomas Melichar	*Synoecha marmorata*		Queensland
ANICB266-06	ANIC Gen No. 001398	637[0n]	KJ169036	-		Australian National Insect Collection	*Synoecha marmorata*		Queensland
SPTMC312-12	BC-Mel2313	658[0n]	KJ168724	-		Research Collection of Tomas Melichar	*Synoecha marmorata*		Queensland
SPTMC307-12	BC-Mel2308	658[0n]	KJ168631	-		Research Collection of Tomas Melichar	*Synoecha marmorata*		Queensland
SPTMC310-12	BC-Mel2311	639[0n]	KJ168901	-		Research Collection of Tomas Melichar	*Synoecha marmorata*		Queensland
SPTMC306-12	BC-Mel2307	658[0n]	KJ169024	-		Research Collection of Tomas Melichar	*Synoecha marmorata*		Queensland
ANICC177-08	ANIC Gen No. 003340	609[0n]	KJ169349	-		Australian National Insect Collection	*Synoecha marmorata*		Queensland
ANICB265-06	ANIC Gen No. 001397	658[0n]	JN678544	-		Australian National Insect Collection	*Synoecha marmorata*		Queensland
SPTMC311-12	BC-Mel2312	633[0n]	KJ169250	-		Research Collection of Tomas Melichar	*Synoecha marmorata*		Queensland
SOWD285-06	BC-Hax3184	658[0n]	KJ169338	-		Research Collection of Jean Haxaire	*Tetrachroa edwardsi*		Queensland
SOWD284-06	BC-Hax3183	658[0n]	KJ168919	-		Research Collection of Jean Haxaire	*Tetrachroa edwardsi*		Queensland
SOWD286-06	BC-Hax3185	658[0n]	JN678597	-		Research Collection of Jean Haxaire	*Tetrachroa edwardsi*		Queensland
ANICB284-06	ANIC Gen No. 000135	646[0n]	KJ168864	-		Australian National Insect Collection	*Tetrachroa edwardsi*		Queensland
GWORD039-07	BC ZSM Lep 02671	658[0n]	KJ168717	-		Bavarian State Collection of Zoology	*Theretra celata*		Queensland
GWORB3230-08	BC ZSM Lep 05768	658[0n]	KJ168611	-		Bavarian State Collection of Zoology	*Theretra celata*		Queensland
GWORD046-07	BC ZSM Lep 02678	658[0n]	KJ169291	-		Bavarian State Collection of Zoology	*Theretra celata*		Queensland
LOQ343-04	04HBL004343	584[0n]	KJ168683	-		Biodiversity Institute of Ontario	*Theretra celata*		Queensland

Process ID	Sample ID	COI-5P	GB Acc. COI	28S-D2	GB Acc. 28S	Institution Storing	Species	Types	Origin
GWOR1239-07	BC ZSM Lep 01991	658[0n]	KJ169177	-		Bavarian State Collection of Zoology	*Theretra celata*		Queensland
NSWHJ004-10	09-NSWHH-0017	644[0n]	HM380358	-		Biodiversity Institute of Ontario	*Theretra celata*		New South Wales
GWORB3271-08	BC ZSM Lep 05809	658[0n]	KJ168882	-		Bavarian State Collection of Zoology	*Theretra celata*		Queensland
ANICC007-08	ANIC Gen No. 003170	636[0n]	KJ169064	-		Australian National Insect Collection	*Theretra celata*		Queensland
LOQ342-04	04HBL004342	658[0n]	KJ168633	-		Biodiversity Institute of Ontario	*Theretra celata*		Queensland
SOWC844-06	BC-Hax2743	513[0n]	KJ169394	-		Research Collection of Jean Haxaire	*Theretra celata*		Queensland
ANICC006-08	ANIC Gen No. 003169	595[3n]	KJ169401	-		Australian National Insect Collection	*Theretra celata*		Queensland
ANICC004-08	ANIC Gen No. 003167	658[0n]	KJ169226	-		Australian National Insect Collection	*Theretra celata*		Queensland
GWORB855-07	BC ZSM Lep 01325	622[0n]	KJ169161	-		Bavarian State Collection of Zoology	*Theretra celata*		Queensland
GWORB3272-08	BC ZSM Lep 05810	658[0n]	KJ168982	-		Bavarian State Collection of Zoology	*Theretra celata*		Queensland
LNSWE015-06	06-NSWE-00015	656[0n]	KJ169190	-		Biodiversity Institute of Ontario	*Theretra celata*		New South Wales
LOQT217-06	2006-LOQT-217	642[0n]	KJ169070	-		Biodiversity Institute of Ontario	*Theretra celata*		Queensland
ANICC005-08	ANIC Gen No. 003168	609[0n]	KJ169262	-		Australian National Insect Collection	*Theretra celata*		Queensland
GWORD043-07	BC ZSM Lep 02675	658[0n]	KJ168870	-		Bavarian State Collection of Zoology	*Theretra indistincta*		Queensland
SPTMB248-10	BC-Mel1250	658[0n]	HQ977224	-		Research Collection of Tomas Melichar	*Theretra indistincta*		Northern Territory
SOWC850-06	BC-Hax2749	608[0n]	KJ169020	-		Research Collection of Jean Haxaire	*Theretra indistincta*		Queensland
LOQ344-04	04HBL004344	602[1n]	KJ169071	-		Biodiversity Institute of Ontario	*Theretra indistincta*		Queensland
NSWHJ050-10	09-NSWHH-0063	658[0n]	HM380403	-		Biodiversity Institute of Ontario	*Theretra indistincta*		New South Wales
SPTMB247-10	BC-Mel1249	658[0n]	HQ977223	-		Research Collection of Tomas Melichar	*Theretra indistincta*		Northern Territory
LNSWE027-06	06-NSWE-00027	552[1n]	KJ168933	-		Biodiversity Institute of Ontario	*Theretra indistincta*		New South Wales
LNSWE014-06	06-NSWE-00014	575[0n]	KJ168661	-		Biodiversity Institute of Ontario	*Theretra indistincta*		New South Wales
GWOR1837-07	BC ZSM Lep 02401	572[0n]	KJ169117	-		Bavarian State Collection of Zoology	*Theretra indistincta*		Queensland
SPTMB246-10	BC-Mel1248	658[0n]	HQ977222	-		Research Collection of Tomas Melichar	*Theretra indistincta*		Queensland
GWORA263-08	BC ZSM Lep 10635	658[0n]	KJ169399	-		Bavarian State Collection of Zoology	*Theretra indistincta*		Northern Territory
GWORB887-07	BC ZSM Lep 01357	632[0n]	KJ169259	-		Bavarian State Collection of Zoology	*Theretra indistincta*		Queensland
GWORI474-09	BC ZSM Lep 13222	658[0n]	KJ168944	-		Bavarian State Collection of Zoology	*Theretra inornata*		Northern Territory
GWORI477-09	BC ZSM Lep 13225	658[0n]	KJ169314	-		Bavarian State Collection of Zoology	*Theretra inornata*		Northern Territory
GWORI478-09	BC ZSM Lep 13226	621[0n]	KJ168656	-		Bavarian State Collection of Zoology	*Theretra inornata*		Northern Territory
GWORI482-09	BC ZSM Lep 13230	658[0n]	KJ168737	-		Bavarian State Collection of Zoology	*Theretra inornata*		Northern Territory
GWOR1248-07	BC ZSM Lep 02000	656[0n]	KJ169239	-		Bavarian State Collection of Zoology	*Theretra inornata*		Queensland
GWORI476-09	BC ZSM Lep 13224	658[0n]	KJ169240	-		Bavarian State Collection of Zoology	*Theretra inornata*		Northern Territory
GWORI481-09	BC ZSM Lep 13229	658[0n]	KJ168980	-		Bavarian State Collection of Zoology	*Theretra inornata*		Northern Territory
GWOR1249-07	BC ZSM Lep 02001	658[0n]	JN678609	-		Bavarian State Collection of Zoology	*Theretra inornata*		Queensland
GWORI479-09	BC ZSM Lep 13227	658[0n]	KJ169387	-		Bavarian State Collection of Zoology	*Theretra inornata*		Northern Territory
GWORI473-09	BC ZSM Lep 13221	658[0n]	KJ169268	-		Bavarian State Collection of Zoology	*Theretra inornata*		Northern Territory
GWORI475-09	BC ZSM Lep 13223	577[0n]	KJ168715	-		Bavarian State Collection of Zoology	*Theretra inornata*		Northern Territory

Process ID	Sample ID	COI-5P	GB Acc. COI	28S-D2	GB Acc. 28S	Institution Storing	Species	Types	Origin
GWORI480-09	BC ZSM Lep 13228	658[0n]	KJ168749	-		Bavarian State Collection of Zoology	*Theretra inornata*		Northern Territory
GWOR1231-07	BC ZSM Lep 01983	658[0n]	KJ169396	-		Bavarian State Collection of Zoology	*Theretra latreillii*		Queensland
LOQTB049-07	gvc6825-1L	658[0n]	KJ169124	-		Biodiversity Institute of Ontario	*Theretra latreillii*		Queensland
GWOR1241-07	BC ZSM Lep 01993	658[0n]	KJ168906	-		Bavarian State Collection of Zoology	*Theretra latreillii*		Queensland
GWORB859-07	BC ZSM Lep 01329	632[0n]	KJ168605	-		Bavarian State Collection of Zoology	*Theretra latreillii*		Queensland
GWOR1838-07	BC ZSM Lep 02402	582[0n]	KJ169175	-		Bavarian State Collection of Zoology	*Theretra latreillii*		Queensland
LOQTC919-08	gvc8566-1L	658[0n]	KJ169201	-		Research Collection of Graeme V. Cocks	*Theretra latreillii*		Queensland
GWORG484-08	BC ZSM Lep 14924	658[0n]	KJ168971	-		Bavarian State Collection of Zoology	*Theretra latreillii*		Queensland
NSWHJ026-10	09-NSWHH-0039	658[0n]	HM380380	-		Biodiversity Institute of Ontario	*Theretra latreillii*		New South Wales
SOWC868-06	BC-Hax2767	494[0n]	KJ168606	-		Research Collection of Jean Haxaire	*Theretra latreillii*		Queensland
GWORB858-07	BC ZSM Lep 01328	632[0n]	KJ168758	-		Bavarian State Collection of Zoology	*Theretra latreillii*		Queensland
LOQTB024-07	gvc6798-1L	658[0n]	KJ169352	-		Biodiversity Institute of Ontario	*Theretra latreillii*		Queensland
IMLR1085-11	IM08-2609	658[0n]	KJ169182	-		Biodiversity Institute of Ontario	*Theretra latreillii*		Queensland
GWOR1694-07	BC ZSM Lep 02258	644[0n]	KJ168691	-		Bavarian State Collection of Zoology	*Theretra latreillii*		Queensland
GWOR1232-07	BC ZSM Lep 01984	658[0n]	KJ169058	-		Bavarian State Collection of Zoology	*Theretra latreillii*		Queensland
GWORB898-07	BC ZSM Lep 01368	627[0n]	KJ168640	-		Bavarian State Collection of Zoology	*Theretra latreillii*		Queensland
NSWHJ038-10	09-NSWHH-0051	658[0n]	HM380392	-		Biodiversity Institute of Ontario	*Theretra latreillii*		New South Wales
GWORA136-08	BC ZSM Lep 10508	645[1n]	KJ168733	-		Bavarian State Collection of Zoology	*Theretra latreillii*		Western Australia
LNSWE001-06	06-NSWE-00001	532[0n]	KJ168838	-		Biodiversity Institute of Ontario	*Theretra latreillii*		New South Wales
GWORB890-07	BC ZSM Lep 01360	621[0n]	KJ169174	-		Bavarian State Collection of Zoology	*Theretra latreillii*		Queensland
IMLQ230-07	IM07-0146	658[0n]	KJ168767	-		Biodiversity Institute of Ontario	*Theretra latreillii*		Queensland
GWORB891-07	BC ZSM Lep 01361	621[0n]	KJ169206	-		Bavarian State Collection of Zoology	*Theretra latreillii*		Queensland
GWORI507-09	BC ZSM Lep 13255	627[0n]	KJ168932	-		Bavarian State Collection of Zoology	*Theretra latreillii*		Northern Territory
GWOR1238-07	BC ZSM Lep 01990	658[0n]	KJ169397	-		Bavarian State Collection of Zoology	*Theretra latreillii*		Queensland
GWORD040-07	BC ZSM Lep 02672	658[0n]	KJ169179	-		Bavarian State Collection of Zoology	*Theretra latreillii*		Queensland
LOQTD909-09	gvc11071-1L	658[0n]	KJ169135	-		Research Collection of Graeme V. Cocks	*Theretra latreillii*		Queensland
LOQTD889-09	gvc11028-1L	658[0n]	KJ168848	-		Research Collection of Graeme V. Cocks	*Theretra latreillii*		Queensland
GWORB861-07	BC ZSM Lep 01331	632[0n]	KJ168866	-		Bavarian State Collection of Zoology	*Theretra latreillii*		Queensland
GWOR1229-07	BC ZSM Lep 01981	657[0n]	KJ168913	-		Bavarian State Collection of Zoology	*Theretra latreillii*		Queensland
GWORB875-07	BC ZSM Lep 01345	627[0n]	KJ169003	-		Bavarian State Collection of Zoology	*Theretra latreillii*		Queensland
GWORB3270-08	BC ZSM Lep 05808	658[0n]	KJ169112	-		Bavarian State Collection of Zoology	*Theretra latreillii*		Queensland
GWORC234-07	BC ZSM Lep 02584	653[0n]	KJ168854	-		Bavarian State Collection of Zoology	*Theretra latreillii*		Queensland
GWOR1236-07	BC ZSM Lep 01988	658[0n]	KJ168751	-		Bavarian State Collection of Zoology	*Theretra latreillii*		Queensland
GWORB857-07	BC ZSM Lep 01327	627[0n]	KJ168894	-		Bavarian State Collection of Zoology	*Theretra latreillii*		Queensland
GWORB860-07	BC ZSM Lep 01330	621[0n]	KJ168602	-		Bavarian State Collection of Zoology	*Theretra latreillii*		Queensland
LOQB003-05	Moth 003.01LZ	575[0n]	KJ168959	-		Biodiversity Institute of Ontario	*Theretra latreillii*		Queensland

Process ID	Sample ID	COI-5P	GB Acc. COI	28S-D2	GB Acc. 28S	Institution Storing	Species	Types	Origin
GWORB888-07	BC ZSM Lep 01358	621[0n]	KJ168922	-		Bavarian State Collection of Zoology	*Theretra latreillii*		Queensland
GWORB3269-08	BC ZSM Lep 05807	658[0n]	KJ169045	-		Bavarian State Collection of Zoology	*Theretra latreillii*		Queensland
GWORB874-07	BC ZSM Lep 01344	607[0n]	KJ169099	-		Bavarian State Collection of Zoology	*Theretra latreillii*		Queensland
GWORG360-08	BC ZSM Lep 08476	658[0n]	KJ168779	-		Bavarian State Collection of Zoology	*Theretra latreillii*		Queensland
LOQB004-05	Moth 004.01LZ	575[0n]	KJ168946	-		Biodiversity Institute of Ontario	*Theretra latreillii*		Queensland
GWORB856-07	BC ZSM Lep 01326	614[0n]	KJ169035	-		Bavarian State Collection of Zoology	*Theretra latreillii*		Queensland
GWORC233-07	BC ZSM Lep 02583	622[0n]	KJ168912	-		Bavarian State Collection of Zoology	*Theretra latreillii*		Queensland
IMLR890-11	IM06-0552	658[0n]	KJ168625	-		Biodiversity Institute of Ontario	*Theretra latreillii*		Queensland
NSWHJ074-10	09-NSWHH-0087	658[0n]	HM380427	-		Biodiversity Institute of Ontario	*Theretra latreillii*		New South Wales
GWORB3267-08	BC ZSM Lep 05805	658[0n]	KJ168918	-		Bavarian State Collection of Zoology	*Theretra latreillii*		Queensland
ANIC347-06	ANIC Gen No. 000539	658[0n]	KJ168923	-		Australian National Insect Collection	*Theretra latreillii*		Queensland
NSWHJ028-10	09-NSWHH-0041	658[0n]	HM380382	-		Biodiversity Institute of Ontario	*Theretra latreillii*		New South Wales
LOQTB139-07	gvc6902-1L	658[0n]	KJ168871	-		Biodiversity Institute of Ontario	*Theretra latreillii*		Queensland
NSWHJ062-10	09-NSWHH-0075	622[0n]	HM380415	-		Biodiversity Institute of Ontario	*Theretra latreillii*		New South Wales
GWORG303-08	BC ZSM Lep 08419	658[0n]	KJ168802	-		Bavarian State Collection of Zoology	*Theretra latreillii*		Queensland
NSWHJ086-10	09-NSWHH-0099	658[0n]	HM380439	-		Biodiversity Institute of Ontario	*Theretra latreillii*		New South Wales
NSWHJ064-10	09-NSWHH-0077	658[0n]	HM380417	-		Biodiversity Institute of Ontario	*Theretra latreillii*		New South Wales
GWORB876-07	BC ZSM Lep 01346	621[0n]	KJ168770	-		Bavarian State Collection of Zoology	*Theretra latreillii*		Queensland
LOQT712-06	gvc6424-1L	658[0n]	KJ169364	-		Biodiversity Institute of Ontario	*Theretra latreillii*		Queensland
NSWHJ016-10	09-NSWHH-0029	658[0n]	HM380370	-		Biodiversity Institute of Ontario	*Theretra latreillii*		New South Wales
GWOR1695-07	BC ZSM Lep 02259	658[0n]	JN678614	-		Bavarian State Collection of Zoology	*Theretra latreillii*		Queensland
GWORA186-08	BC ZSM Lep 10558	648[1n]	KJ168829	-		Bavarian State Collection of Zoology	*Theretra margarita*		Western Australia
GWORA013-08	BC ZSM Lep 10385	656[0n]	KJ169092	-		Bavarian State Collection of Zoology	*Theretra margarita*		Western Australia
GWORA137-08	BC ZSM Lep 10509	618[0n]	KJ168805	-		Bavarian State Collection of Zoology	*Theretra margarita*		Western Australia
LOQT807-07	gvc6515-1L	658[0n]	KJ169061	-		Biodiversity Institute of Ontario	*Theretra margarita*		Queensland
GWORN236-09	BC ZSM Lep 18264	658[0n]	GU704658	-		Bavarian State Collection of Zoology	*Theretra margarita*		Western Australia
GWORA182-08	BC ZSM Lep 10554	649[0n]	KJ168967	-		Bavarian State Collection of Zoology	*Theretra margarita*		Western Australia
LOQTB002-07	gvc6771-1L	658[0n]	KJ169274	-		Biodiversity Institute of Ontario	*Theretra margarita*		Queensland
GWORC188-07	BC ZSM Lep 02538	630[0n]	KJ169223	-		Bavarian State Collection of Zoology	*Theretra margarita*		Queensland
GWORA188-08	BC ZSM Lep 10560	600[4n]	KJ169392	-		Bavarian State Collection of Zoology	*Theretra margarita*		Western Australia
GWORN237-09	BC ZSM Lep 18265	658[0n]	GU704656	-		Bavarian State Collection of Zoology	*Theretra margarita*		Western Australia
LOQTI518-11	gvc17066-1L	658[0n]	KJ169302	-		Biodiversity Institute of Ontario	*Theretra margarita*		Queensland
GWORA088-08	BC ZSM Lep 10460	658[0n]	KJ168957	-		Bavarian State Collection of Zoology	*Theretra margarita*		Western Australia
LOQTI517-11	gvc17065-1L	658[0n]	KJ168904	-		Biodiversity Institute of Ontario	*Theretra margarita*		Queensland
LOQTE720-10	gvc13863-1L	652[6n]	KJ169379	-		Research Collection of Graeme V. Cocks	*Theretra margarita*		Queensland
GWORN239-09	BC ZSM Lep 18267	658[0n]	GU704655	-		Bavarian State Collection of Zoology	*Theretra margarita*		Western Australia

Process ID	Sample ID	COI-5P	GB Acc. COI	28S-D2	GB Acc. 28S	Institution Storing	Species	Types	Origin
GWORD049-07	BC ZSM Lep 02681	658[0n]	KJ168909	-		Bavarian State Collection of Zoology	*Theretra margarita*		Queensland
GWORA138-08	BC ZSM Lep 10510	649[0n]	KJ168748	-		Bavarian State Collection of Zoology	*Theretra margarita*		Western Australia
GWORN238-09	BC ZSM Lep 18266	646[0n]	GU704657	-		Bavarian State Collection of Zoology	*Theretra margarita*		Western Australia
GWORA187-08	BC ZSM Lep 10559	646[0n]	KJ169404	-		Bavarian State Collection of Zoology	*Theretra margarita*		Western Australia
GWORD061-07	BC ZSM Lep 02693	658[0n]	JN678617	-		Bavarian State Collection of Zoology	*Theretra margarita*		Queensland
LOQTI516-11	gvc17064-1L	658[0n]	KJ169114	-		Biodiversity Institute of Ontario	*Theretra margarita*		Queensland
GWORD050-07	BC ZSM Lep 02682	572[0n]	KJ169378	-		Bavarian State Collection of Zoology	*Theretra margarita*		Queensland
LOQT210-06	2006-LOQT-210	632[0n]	KJ169030	-		Biodiversity Institute of Ontario	*Theretra margarita*		Queensland
GWORA099-08	BC ZSM Lep 10471	557[1n]	KJ168862	-		Bavarian State Collection of Zoology	*Theretra margarita*		Northern Territory
LOQTE688-10	gvc13805-1L	641[0n]	HM879445	-		Research Collection of Graeme V. Cocks	*Theretra margarita*		Queensland
GWORB2256-08	BC ZSM Lep 11056	609[0n]	KJ168999	-		Bavarian State Collection of Zoology	*Theretra margarita*		Northern Territory
LOQT700-06	gvc6396-1L	658[0n]	KJ168743	-		Biodiversity Institute of Ontario	*Theretra margarita*		Queensland
LOQTB046-07	gvc6812-1L	642[1n]	KJ169264	-		Biodiversity Institute of Ontario	*Theretra margarita*		Queensland
GWORA162-08	BC ZSM Lep 10534	615[0n]	KJ168798	-		Bavarian State Collection of Zoology	*Theretra margarita*		Western Australia
GWORA124-08	BC ZSM Lep 10496	646[0n]	KJ169147	-		Bavarian State Collection of Zoology	*Theretra margarita*		Western Australia
NSWHM001-11	BIOUG00851-F06	658[0n]	JN280978	-		Biodiversity Institute of Ontario	*Theretra nessus*		New South Wales
LNSWE003-06	06-NSWE-00003	658[0n]	KJ169156	-		Biodiversity Institute of Ontario	*Theretra nessus*		New South Wales
NSWHM013-11	BIOUG00851-G06	658[0n]	JN280982	-		Biodiversity Institute of Ontario	*Theretra nessus*		New South Wales
LNSWE092-06	06-NSWE-00092	584[0n]	KJ169389	-		Biodiversity Institute of Ontario	*Theretra nessus*		New South Wales
NSWHM015-11	BIOUG00851-G08	658[0n]	JN280983	-		Biodiversity Institute of Ontario	*Theretra nessus*		New South Wales
GWORB882-07	BC ZSM Lep 01352	632[0n]	KJ169269	-		Bavarian State Collection of Zoology	*Theretra nessus*		Queensland
GWORB884-07	BC ZSM Lep 01354	632[0n]	KJ168938	-		Bavarian State Collection of Zoology	*Theretra nessus*		Queensland
NSWHM2159-11	BIOUG00961-F10	658[0n]	JN306777	-		Biodiversity Institute of Ontario	*Theretra nessus*		New South Wales
LNSWE045-06	06-NSWE-00045	591[0n]	KJ168707	-		Biodiversity Institute of Ontario	*Theretra nessus*		New South Wales
LNSWE069-06	06-NSWE-00069	658[0n]	KJ168855	-		Biodiversity Institute of Ontario	*Theretra nessus*		New South Wales
LNSWE088-06	06-NSWE-00088	541[0n]	KJ168898	-		Biodiversity Institute of Ontario	*Theretra nessus*		New South Wales
NSWHM2152-11	BIOUG00961-F03	658[0n]	JN306770	-		Biodiversity Institute of Ontario	*Theretra nessus*		New South Wales
GWORB872-07	BC ZSM Lep 01342	632[0n]	KJ168775	-		Bavarian State Collection of Zoology	*Theretra nessus*		Queensland
GWORC241-07	BC ZSM Lep 02591	658[0n]	KJ168794	-		Bavarian State Collection of Zoology	*Theretra nessus*		Queensland
GWORG359-08	BC ZSM Lep 08475	658[0n]	KJ169292	-		Bavarian State Collection of Zoology	*Theretra nessus*		Queensland
NSWHM2019-11	BIOUG00961-A02	657[1n]	JN280989	-		Biodiversity Institute of Ontario	*Theretra nessus*		New South Wales
GWORB865-07	BC ZSM Lep 01335	658[0n]	KJ169120	-		Bavarian State Collection of Zoology	*Theretra oldenlandiae lewini*		Queensland
GWORD055-07	BC ZSM Lep 02687	658[0n]	KJ169051	-		Bavarian State Collection of Zoology	*Theretra oldenlandiae lewini*		Queensland
GWORD064-07	BC ZSM Lep 02696	609[0n]	KJ169105	-		Bavarian State Collection of Zoology	*Theretra oldenlandiae lewini*		Queensland
LOQB571-05	Moth 050.03CL	656[2n]	KJ169214	-		Biodiversity Institute of Ontario	*Theretra oldenlandiae lewini*		Queensland
NSWHJ014-10	09-NSWHH-0027	658[0n]	HM380368	-		Biodiversity Institute of Ontario	*Theretra oldenlandiae lewini*		New South Wales

Process ID	Sample ID	COI-5P	GB Acc. COI	28S-D2	GB Acc. 28S	Institution Storing	Species	Types	Origin
NSWHH054-09	08-NSWHH-0054	658[0n]	KJ168756	-		Biodiversity Institute of Ontario	*Theretra oldenlandiae lewini*		New South Wales
GWORD054-07	BC ZSM Lep 02686	658[0n]	KJ168921	-		Bavarian State Collection of Zoology	*Theretra oldenlandiae lewini*		Queensland
NSWHJ040-10	09-NSWHH-0053	658[0n]	HM380394	-		Biodiversity Institute of Ontario	*Theretra oldenlandiae lewini*		New South Wales
GWORD059-07	BC ZSM Lep 02691	658[0n]	KJ169366	-		Bavarian State Collection of Zoology	*Theretra oldenlandiae lewini*		Queensland
GWORI496-09	BC ZSM Lep 13244	658[0n]	KJ169162	-		Bavarian State Collection of Zoology	*Theretra oldenlandiae lewini*		Northern Territory
GWORI548-09	BC ZSM Lep 13296	658[0n]	KJ169094	-		Bavarian State Collection of Zoology	*Theretra oldenlandiae lewini*		Northern Territory
GWORD065-07	BC ZSM Lep 02697	609[0n]	KJ169122	-		Bavarian State Collection of Zoology	*Theretra oldenlandiae lewini*		Queensland
LNSWE109-06	06-NSWE-00109	593[0n]	KJ168674	-		Biodiversity Institute of Ontario	*Theretra oldenlandiae lewini*		New South Wales
GWORB2215-08	BC ZSM Lep 11015	640[0n]	KJ169049	-		Bavarian State Collection of Zoology	*Theretra oldenlandiae lewini*		Northern Territory
GWORG563-08	BC ZSM Lep 15003	658[0n]	KJ168995	-		Bavarian State Collection of Zoology	*Theretra oldenlandiae lewini*		Queensland
LNSWE056-06	06-NSWE-00056	606[0n]	KJ168936	-		Biodiversity Institute of Ontario	*Theretra oldenlandiae lewini*		New South Wales
GWORD066-07	BC ZSM Lep 02698	658[0n]	KJ168766	-		Bavarian State Collection of Zoology	*Theretra oldenlandiae lewini*		Queensland
LOQT453-06	gvc6234-1L	658[0n]	KJ169287	-		Biodiversity Institute of Ontario	*Theretra oldenlandiae lewini*		Queensland
NSWHM2156-11	BIOUG00961-F07	658[0n]	JN306774	-		Biodiversity Institute of Ontario	*Theretra oldenlandiae lewini*		New South Wales
LNSWE107-06	06-NSWE-00107	593[0n]	KJ169234	-		Biodiversity Institute of Ontario	*Theretra oldenlandiae lewini*		New South Wales
GWORD053-07	BC ZSM Lep 02685	658[0n]	KJ168859	-		Bavarian State Collection of Zoology	*Theretra oldenlandiae lewini*		Queensland
LOQTC768-08	gvc8413-1L	657[1n]	KJ168861	-		Biodiversity Institute of Ontario	*Theretra oldenlandiae lewini*		Queensland
LNSWE096-06	06-NSWE-00096	658[0n]	KJ169160	-		Biodiversity Institute of Ontario	*Theretra oldenlandiae lewini*		New South Wales
GWORD063-07	BC ZSM Lep 02695	609[0n]	KJ169067	-		Bavarian State Collection of Zoology	*Theretra oldenlandiae lewini*		Queensland
GWORI543-09	BC ZSM Lep 13291	658[0n]	KJ168703	-		Bavarian State Collection of Zoology	*Theretra oldenlandiae lewini*		Northern Territory
GWORD057-07	BC ZSM Lep 02689	658[0n]	KJ168689	-		Bavarian State Collection of Zoology	*Theretra oldenlandiae lewini*		Queensland
LNSWE091-06	06-NSWE-00091	658[0n]	KJ168648	-		Biodiversity Institute of Ontario	*Theretra oldenlandiae lewini*		New South Wales
GWORI495-09	BC ZSM Lep 13243	658[0n]	KJ168827	-		Bavarian State Collection of Zoology	*Theretra oldenlandiae lewini*		Northern Territory
NSWHM032-11	BIOUG00851-F05	658[0n]	JN280988	-		Biodiversity Institute of Ontario	*Theretra oldenlandiae lewini*		New South Wales
LOQTE772-10	gvc14063-1L	658[0n]	HQ572317	-		Research Collection of Graeme V. Cocks	*Theretra oldenlandiae lewini*		Queensland
GWORH540-09	BC ZSM Lep 10254	658[0n]	KJ169310	-		Bavarian State Collection of Zoology	*Theretra oldenlandiae lewini*		Queensland
GWORA139-08	BC ZSM Lep 10511	602[0n]	KJ168644	-		Bavarian State Collection of Zoology	*Theretra oldenlandiae lewini*		Western Australia
LNSWE089-06	06-NSWE-00089	589[0n]	KJ169119	-		Biodiversity Institute of Ontario	*Theretra oldenlandiae lewini*		New South Wales
LOQTC417-07	gvc8053-1L	658[0n]	KJ169140	-		Biodiversity Institute of Ontario	*Theretra oldenlandiae lewini*		Queensland
GWORB2190-08	BC ZSM Lep 10990	657[0n]	KJ169155	-		Bavarian State Collection of Zoology	*Theretra oldenlandiae lewini*		Northern Territory
LOQT800-07	gvc6507-1L	658[0n]	KJ168666	-		Biodiversity Institute of Ontario	*Theretra oldenlandiae lewini*		Queensland
LOQTB068-07	gvc6834-1L	597[0n]	KJ168690	-		Biodiversity Institute of Ontario	*Theretra oldenlandiae lewini*		Queensland
LOQT626-06	gvc5640-1L	656[0n]	KJ169406	-		Research Collection of Graeme V. Cocks	*Theretra oldenlandiae lewini*		Queensland
SOWE243-07	BC-Hax4142	407[0n]	KJ169289	-		Research Collection of Jean Haxaire	*Theretra oldenlandiae lewini*		
GWORD062-07	BC ZSM Lep 02694	658[0n]	KJ169069	-		Bavarian State Collection of Zoology	*Theretra oldenlandiae lewini*		Queensland
LNSWE007-06	06-NSWE-00007	598[0n]	KJ168680	-		Biodiversity Institute of Ontario	*Theretra oldenlandiae lewini*		New South Wales

Process ID	Sample ID	COI-5P	GB Acc. COI	28S-D2	GB Acc. 28S	Institution Storing	Species	Types	Origin
LOQTE588-10	gvc13322-1L	658[0n]	HM387365	-		Research Collection of Graeme V. Cocks	*Theretra oldenlandiae lewini*		Queensland
GWORB3243-08	BC ZSM Lep 05781	658[0n]	KJ168764	-		Bavarian State Collection of Zoology	*Theretra oldenlandiae lewini*		Queensland
GWORI544-09	BC ZSM Lep 13292	658[0n]	KJ169191	-		Bavarian State Collection of Zoology	*Theretra oldenlandiae lewini*		Northern Territory
GWORB2197-08	BC ZSM Lep 10997	658[0n]	KJ168723	-		Bavarian State Collection of Zoology	*Theretra oldenlandiae lewini*		Northern Territory
LOQ345-04	04HBL004345	658[0n]	KJ169065	-		Biodiversity Institute of Ontario	*Theretra oldenlandiae lewini*		Queensland
GWORD056-07	BC ZSM Lep 02688	655[0n]	KJ168706	-		Bavarian State Collection of Zoology	*Theretra oldenlandiae lewini*		Queensland
LNSWE011-06	06-NSWE-00011	612[1n]	KJ168726	-		Biodiversity Institute of Ontario	*Theretra oldenlandiae lewini*		New South Wales
GWORD067-07	BC ZSM Lep 02699	658[0n]	KJ168823	-		Bavarian State Collection of Zoology	*Theretra oldenlandiae lewini*		Queensland
LNSWE098-06	06-NSWE-00098	657[1n]	KJ169034	-		Biodiversity Institute of Ontario	*Theretra oldenlandiae lewini*		New South Wales
LNSWE032-06	06-NSWE-00032	608[0n]	KJ168739	-		Biodiversity Institute of Ontario	*Theretra oldenlandiae lewini*		New South Wales
SOWE242-07	BC-Hax4141	647[0n]	KJ169108	-		Research Collection of Jean Haxaire	*Theretra oldenlandiae lewini*		
GWORB3268-08	BC ZSM Lep 05806	658[0n]	KJ169186	-		Bavarian State Collection of Zoology	*Theretra oldenlandiae lewini*		Queensland
LNSWE082-06	06-NSWE-00082	511[0n]	KJ168811	-		Biodiversity Institute of Ontario	*Theretra oldenlandiae lewini*		New South Wales
LNSWE132-06	06-NSWE-00132	573[0n]	KJ169083	-		Biodiversity Institute of Ontario	*Theretra oldenlandiae lewini*		New South Wales
LNSWE016-06	06-NSWE-00016	604[0n]	KJ169362	-		Biodiversity Institute of Ontario	*Theretra oldenlandiae lewini*		New South Wales
GWORD052-07	BC ZSM Lep 02684	609[0n]	KJ168673	-		Bavarian State Collection of Zoology	*Theretra oldenlandiae lewini*		Queensland
LOQTE672-10	gvc13682-1L	614[0n]	HM879434	-		Research Collection of Graeme V. Cocks	*Theretra oldenlandiae lewini*		Queensland
LOQTD848-08	gvc10517-1L	658[0n]	GU671658	-		Research Collection of Graeme V. Cocks	*Theretra oldenlandiae lewini*		Queensland
LNSWE101-06	06-NSWE-00101	599[0n]	KJ169085	-		Biodiversity Institute of Ontario	*Theretra oldenlandiae lewini*		New South Wales
LNSWE100-06	06-NSWE-00100	657[0n]	KJ168618	-		Biodiversity Institute of Ontario	*Theretra oldenlandiae lewini*		New South Wales
GWORG562-08	BC ZSM Lep 15002	656[2n]	KJ169249	-		Bavarian State Collection of Zoology	*Theretra oldenlandiae lewini*		Queensland
LOQB591-05	Moth 070.03CL	574[0n]	KJ169270	-		Biodiversity Institute of Ontario	*Theretra oldenlandiae lewini*		Queensland
LNSWE121-06	06-NSWE-00121	584[0n]	KJ168881	-		Biodiversity Institute of Ontario	*Theretra oldenlandiae lewini*		New South Wales
NSWHM455-11	BIOUG00912-G04	658[0n]	JN280992	-		Biodiversity Institute of Ontario	*Theretra oldenlandiae lewini*		New South Wales
LNSWE119-06	06-NSWE-00119	509[1n]	KJ168837	-		Biodiversity Institute of Ontario	*Theretra oldenlandiae lewini*		New South Wales
LNSWE118-06	06-NSWE-00118	583[0n]	KJ169053	-		Biodiversity Institute of Ontario	*Theretra oldenlandiae lewini*		New South Wales
LNSWE075-06	06-NSWE-00075	516[1n]	KJ169032	-		Biodiversity Institute of Ontario	*Theretra oldenlandiae lewini*		New South Wales
LOQ346-04	04HBL004346	658[0n]	KJ168616	-		Biodiversity Institute of Ontario	*Theretra oldenlandiae lewini*		Queensland
GWORI506-09	BC ZSM Lep 13254	658[0n]	KJ169100	-		Bavarian State Collection of Zoology	*Theretra oldenlandiae lewini*		Northern Territory
LNSWE125-06	06-NSWE-00125	608[0n]	KJ169001	-		Biodiversity Institute of Ontario	*Theretra oldenlandiae lewini*		New South Wales
LNSWE006-06	06-NSWE-00006	609[0n]	KJ169319	-		Biodiversity Institute of Ontario	*Theretra oldenlandiae lewini*		New South Wales
GWORD058-07	BC ZSM Lep 02690	657[1n]	KJ168729	-		Bavarian State Collection of Zoology	*Theretra oldenlandiae lewini*		Queensland
GWORD068-07	BC ZSM Lep 02700	648[2n]	KJ168820	-		Bavarian State Collection of Zoology	*Theretra oldenlandiae lewini*		Queensland
LNSWE130-06	06-NSWE-00130	658[0n]	KJ168609	-		Biodiversity Institute of Ontario	*Theretra oldenlandiae lewini*		New South Wales
LNSWE129-06	06-NSWE-00129	658[0n]	KJ168962	-		Biodiversity Institute of Ontario	*Theretra oldenlandiae lewini*		New South Wales
GWORI499-09	BC ZSM Lep 13247	658[0n]	KJ168747	-		Bavarian State Collection of Zoology	*Theretra oldenlandiae lewini*		Northern Territory

Process ID	Sample ID	COI-5P	GB Acc. COI	28S-D2	GB Acc. 28S	Institution Storing	Species	Types	Origin
NSWHM018-11	BIOUG00851-G11	658[0n]	JN280985	-		Biodiversity Institute of Ontario	*Theretra oldenlandiae lewini*		New South Wales
LOQT023-06	2006-LOQT-023	587[0n]	KJ169136	-		Biodiversity Institute of Ontario	*Theretra oldenlandiae lewini*		Queensland
LOQT1052-07	gvc6754-1L	658[0n]	KJ168681	-		Biodiversity Institute of Ontario	*Theretra oldenlandiae lewini*		Queensland
LOQ349-04	04HBL004349	658[0n]	KJ169133	-		Biodiversity Institute of Ontario	*Theretra oldenlandiae lewini*		Queensland
ANICH128-10	10ANIC-04125	609[0n]	HQ923137	-		Australian National Insect Collection	*Theretra queenslandi*		Queensland
LNSWE012-06	06-NSWE-00012	658[0n]	KJ168761	601[0n]	KJ169408	Biodiversity Institute of Ontario	*Theretra queenslandi*		New South Wales
LNSWE115-06	06-NSWE-00115	658[0n]	KJ168645	-		Biodiversity Institute of Ontario	*Theretra queenslandi*		New South Wales
ANICH130-10	10ANIC-04127	658[0n]	HQ923139	-		Australian National Insect Collection	*Theretra queenslandi*		Queensland
ANICC091-08	ANIC Gen No. 003254	577[0n]	KJ168804	-		Australian National Insect Collection	*Theretra queenslandi*		New South Wales
ANICC041-08	ANIC Gen No. 003204	658[0n]	JN678627	-		Australian National Insect Collection	*Theretra queenslandi*		Queensland
LNSWE099-06	06-NSWE-00099	515[0n]	KJ169040	-		Biodiversity Institute of Ontario	*Theretra queenslandi*		New South Wales
ANICH131-10	10ANIC-04128	609[0n]	HQ923140	-		Australian National Insect Collection	*Theretra queenslandi*		Queensland
ANICH129-10	10ANIC-04126	658[0n]	HQ923138	-		Australian National Insect Collection	*Theretra queenslandi*		Queensland
SPTMB239-10	BC-Mel1241	658[0n]	HQ977215	-		Research Collection of Tomas Melichar	*Theretra queenslandi*		Queensland
ANICC042-08	ANIC Gen No. 003205	625[0n]	KJ169138	-		Australian National Insect Collection	*Theretra queenslandi*		Queensland
SPTVA374-07	VAG-2185	609[0n]	KJ168783	-		Research Collection of T. Vaglia	*Theretra queenslandi*		New South Wales
LOQT207-06	2006-LOQT-207	658[0n]	KJ169354	-		Biodiversity Institute of Ontario	*Theretra queenslandi*		Queensland
LNSWE080-06	06-NSWE-00080	556[1n]	KJ169233	-		Biodiversity Institute of Ontario	*Theretra queenslandi*		New South Wales
LNSWE013-06	06-NSWE-00013	658[0n]	KJ169159	-		Biodiversity Institute of Ontario	*Theretra queenslandi*		New South Wales
ANICC001-08	ANIC Gen No. 003164	658[0n]	KJ169275	-		Australian National Insect Collection	*Theretra silhetensis intersecta*		Northern Territory
GWORI502-09	BC ZSM Lep 13250	658[0n]	KJ168948	-		Bavarian State Collection of Zoology	*Theretra silhetensis intersecta*		Northern Territory
ANICC002-08	ANIC Gen No. 003165	658[0n]	KJ168657	-		Australian National Insect Collection	*Theretra silhetensis intersecta*		Northern Territory
GWORI551-09	BC ZSM Lep 13299	658[0n]	KJ168991	-		Bavarian State Collection of Zoology	*Theretra silhetensis intersecta*		Northern Territory
ANICC009-08	ANIC Gen No. 003172	598[0n]	KJ169043	-		Australian National Insect Collection	*Theretra silhetensis intersecta*		Western Australia
LOQC038-05	05-QLD-00038	658[0n]	JN678630	-		Biodiversity Institute of Ontario	*Theretra silhetensis intersecta*		Queensland
LOQTE785-10	gvc14162-1L	658[0n]	HQ572330	-		Research Collection of Graeme V. Cocks	*Theretra silhetensis intersecta*		Queensland
IMLR228-08	IM07-1133	658[0n]	KJ169022	-		Biodiversity Institute of Ontario	*Theretra silhetensis intersecta*		Queensland
IMLR1334-11	IM10-0094	658[0n]	KJ168754	-		Biodiversity Institute of Ontario	*Theretra silhetensis intersecta*		Queensland
GWORY459-10	BC EF Lep 03413	658[0n]	HM913600	-		Research Collection of Egbert Friedrich	*Theretra silhetensis intersecta*		Northern Territory
IMLR1332-11	IM10-0090	658[0n]	KJ169144	-		Biodiversity Institute of Ontario	*Theretra silhetensis intersecta*		Queensland
LOQTE109-09	gvc11552-1L	652[2n]	KJ168741	-		Research Collection of Graeme V. Cocks	*Theretra silhetensis intersecta*		Queensland
IMLQ900-08	IM08-0248	658[0n]	KJ168632	-		Biodiversity Institute of Ontario	*Theretra silhetensis intersecta*		Queensland
LOQTE889-10	gvc14423-1L	658[0n]	HQ572425	-		Research Collection of Graeme V. Cocks	*Theretra silhetensis intersecta*		Queensland
LOQTE052-09	gvc11442-1L	657[1n]	KJ169305	-		Research Collection of Graeme V. Cocks	*Theretra silhetensis intersecta*		Queensland
IMLR899-11	IM07-0028	658[0n]	KJ169019	-		Biodiversity Institute of Ontario	*Theretra silhetensis intersecta*		Queensland
LOQTE824-10	gvc14263-1L	658[0n]	HQ572366	-		Biodiversity Institute of Ontario	*Theretra silhetensis intersecta*		Queensland

Process ID	Sample ID	COI-5P	GB Acc. COI	28S-D2	GB Acc. 28S	Institution Storing	Species	Types	Origin
GWORI542-09	BC ZSM Lep 13290	637[0n]	KJ168641	-		Bavarian State Collection of Zoology	*Theretra silhetensis intersecta*		Northern Territory
GWORY458-10	BC EF Lep 03412	658[0n]	HM913599	-		Research Collection of Egbert Friedrich	*Theretra silhetensis intersecta*		Queensland
SOWD629-06	BC-Hax3528	407[0n]	KJ168642	-		Research Collection of Jean Haxaire	*Theretra silhetensis intersecta*		Queensland
NSWBB1250-08	07-NSWBB-1250	658[0n]	KJ168826	-		Biodiversity Institute of Ontario	*Theretra tryoni*		New South Wales
GWOR1235-07	BC ZSM Lep 01987	645[0n]	KJ168610	-		Bavarian State Collection of Zoology	*Theretra tryoni*		Queensland
GWORG483-08	BC ZSM Lep 14923	407[0n]	KJ169031	-		Bavarian State Collection of Zoology	*Theretra tryoni*		Queensland
GWOR1237-07	BC ZSM Lep 01989	658[0n]	JN678634	-		Bavarian State Collection of Zoology	*Theretra tryoni*		Queensland
GWOR1233-07	BC ZSM Lep 01985	658[0n]	KJ168979	-		Bavarian State Collection of Zoology	*Theretra tryoni*		Queensland
ANICC033-08	ANIC Gen No. 003196	658[0n]	KJ169309	-		Australian National Insect Collection	*Theretra turneri*		Queensland
SOWD644-06	BC-Hax3543	658[0n]	HM384133	-		Research Collection of Jean Haxaire	*Theretra turneri*		
GWORD044-07	BC ZSM Lep 02676	658[0n]	JN678635	-		Bavarian State Collection of Zoology	*Theretra turneri*		Queensland
GWORD045-07	BC ZSM Lep 02677	658[0n]	KJ168612	-		Bavarian State Collection of Zoology	*Theretra turneri*		Queensland
GWORC142-07	BC ZSM Lep 02492	655[0n]	KJ169180	-		Bavarian State Collection of Zoology	*Theretra turneri*		Queensland
SPTMC489-12	BC-Mel2490	658[0n]	KJ168731	-		Research Collection of Tomas Melichar	*Theretra turneri*		Queensland
LOQT839-07	gvc6555-1L	643[0n]	KJ169171	-		Biodiversity Institute of Ontario	*Theretra turneri*		Queensland
SPHJT070-10	BC-JT-27M.1	658[0n]	HQ974998	-		Research Collection of James A. Tuttle	*Zacria vojtechi*		Western Australia
SOWD384-06	BC-Hax3283	607[0n]	JN678714	-		Research Collection of Jean Haxaire	*Zacria vojtechi*	Paratype	Western Australia
SPHJT015-09	BC-JT-28M	307[0n]	KJ169184	-		Research Collection of James A. Tuttle	*Zacria vojtechi*		Western Australia
SOWD385-06	BC-Hax3284	599[0n]	KJ168955	-		Research Collection of Jean Haxaire	*Zacria vojtechi*	Paratype	Western Australia
