# Supplementary material for: Australian Sphingidae – DNA Barcodes Challenge Current Species Boundaries and Distributions
Source: PLoS One. 2014 Jul 2;9(7):e101108. doi: 10.1371/journal.pone.0101108 (PMC4079597; doi:10.1371/journal.pone.0101108)
Supplement: Table S2 — List of conspecific records from outside Australia. (PDF) [file pone.0101108.s009.pdf]

Table S2: List of conspecific records from outside Australia included in the analyses. GB Acc. = GenBank accession numbers.

Process ID	Sample ID	COI-5P	GB Acc. COI	28S-D2	GB Acc. 28S	Institution Storing	Species	Types	Origin
GWORB978-07	BC ZSM Lep 02200	592[2n]	KJ168475	-		Bavarian State Collection of Zoology	*Acosmeryx anceus*		Indonesia, Papua
GWORB948-07	BC ZSM Lep 02170	521[3n]	KJ168320	-		Bavarian State Collection of Zoology	*Acosmeryx anceus*		Indonesia, Papua
GWORB979-07	BC ZSM Lep 02201	592[2n]	KJ168226	-		Bavarian State Collection of Zoology	*Acosmeryx anceus*		Indonesia, Papua
SML189-06	USNM ENT 00196633	658[0n]	KJ168464	602[0n]	KJ168591	Smithsonian Institution	*Acosmeryx anceus*		Papua New Guinea, Gulf
HCPN058-03	USNM ENT 00196048	621[0n]	KJ168191	-		Smithsonian Institution	*Acosmeryx anceus*		Papua New Guinea, Gulf
HCPN061-03	USNM ENT 00196082	595[0n]	KJ168078	-		Smithsonian Institution	*Acosmeryx anceus*		Papua New Guinea, Gulf
SML191-06	USNM ENT 00196635	658[0n]	KJ168162	-		Smithsonian Institution	*Acosmeryx anceus*		Papua New Guinea, Gulf
SML190-06	USNM ENT 00196634	658[0n]	KJ168350	602[0n]	KJ168584	Smithsonian Institution	*Acosmeryx anceus*		Papua New Guinea, Gulf
HCPN059-03	USNM ENT 00196049	588[0n]	KJ168210	-		Smithsonian Institution	*Acosmeryx anceus*		Papua New Guinea, Morobe
SML188-06	USNM ENT 00196632	658[0n]	KJ168151	-		Smithsonian Institution	*Acosmeryx anceus*		Papua New Guinea, Morobe
SML185-06	USNM ENT 00196629	658[0n]	KJ168510	602[0n]	KJ168593	Smithsonian Institution	*Acosmeryx anceus*		Papua New Guinea, Morobe
SML187-06	USNM ENT 00196631	656[0n]	KJ168296	-		Smithsonian Institution	*Acosmeryx anceus*		Papua New Guinea, Morobe
SPUEA090-07	BC-EMEM0090	658[0n]	KJ168071	-		Entomologisches Museum Eitschberger	*Acosmeryx anceus subdentata*		China, Guangxi
SPUEA088-07	BC-EMEM0088	658[0n]	KJ168214	-		Entomologisches Museum Eitschberger	*Acosmeryx anceus subdentata*		China, Hainan
SPUEA091-07	BC-EMEM0091	658[0n]	KJ168565	-		Entomologisches Museum Eitschberger	*Acosmeryx anceus subdentata*		China, Sichuan
SOWD454-06	BC-Hax3353	658[0n]	KJ168212	-		Research Collection of Jean Haxaire	*Acosmeryx anceus subdentata*		China, Yunnan
SPTMB123-10	BC-Mel1125	658[0n]	HQ580938	-		Research Collection of Tomas Melichar	*Acosmeryx anceus subdentata*		India, Karnataka
SPTMB031-09	BC-Mel 1043	658[0n]	GU704548	-		Research Collection of Tomas Melichar	*Acosmeryx anceus subdentata*		India, Karnataka
SPUEA006-07	BC-EMEM0006	658[0n]	KJ168080	-		Entomologisches Museum Eitschberger	*Acosmeryx anceus subdentata*		Indonesia, Nusa Tenggara Barat
SPUEA089-07	BC-EMEM0089	658[0n]	KJ168399	-		Entomologisches Museum Eitschberger	*Acosmeryx anceus subdentata*		Laos
SPUEA084-07	BC-EMEM0084	658[0n]	KJ168113	-		Entomologisches Museum Eitschberger	*Acosmeryx anceus subdentata*		Malaysia, Pahang
SPUEA092-07	BC-EMEM0092	658[0n]	KJ168111	-		Entomologisches Museum Eitschberger	*Acosmeryx anceus subdentata*		Malaysia, Perak
SPUEA093-07	BC-EMEM0093	658[0n]	KJ168175	-		Entomologisches Museum Eitschberger	*Acosmeryx anceus subdentata*		Myanmar, Magway
SOWD459-06	BC-Hax3358	607[0n]	KJ168089	-		Research Collection of Jean Haxaire	*Acosmeryx anceus subdentata*		Philippines, Leyte
SOWD460-06	BC-Hax3359	607[0n]	KJ168245	-		Research Collection of Jean Haxaire	*Acosmeryx anceus subdentata*		Philippines, Leyte
SOWD455-06	BC-Hax3354	614[0n]	KJ168231	-		Research Collection of Jean Haxaire	*Acosmeryx anceus subdentata*		Philippines, Mountain
SPUEA087-07	BC-EMEM0087	658[0n]	KJ168109	-		Entomologisches Museum Eitschberger	*Acosmeryx anceus subdentata*		Thailand
SPUEA085-07	BC-EMEM0085	658[0n]	KJ168422	-		Entomologisches Museum Eitschberger	*Acosmeryx anceus subdentata*		Thailand, Chiang Mai
SPUEA086-07	BC-EMEM0086	658[0n]	KJ168107	-		Entomologisches Museum Eitschberger	*Acosmeryx anceus subdentata*		Thailand, Chiang Mai
SOWD457-06	BC-Hax3356	634[0n]	KJ168156	-		Research Collection of Jean Haxaire	*Acosmeryx anceus subdentata*		Timor-Leste, Lautem
SOWD458-06	BC-Hax3357	620[0n]	KJ168469	-		Research Collection of Jean Haxaire	*Acosmeryx anceus subdentata*		Timor-Leste, Lautem
SPUEA094-07	BC-EMEM0094	614[0n]	KJ168265	-		Entomologisches Museum Eitschberger	*Acosmeryx anceus subdentata*		Vietnam, Lam Dong
SPHPA185-07	BC-PhA0185	658[0n]	KJ168285	615[0n]	KJ168581	Research Collection of Philippe Annoyer	*Agrius convolvuli*		Central African Republic
SOWE806-09	BC-Roug1218	658[0n]	GU703833	-		Research Collection of Jean Haxaire	*Agrius convolvuli*		China, Sichuan

Process ID	Sample ID	COI-5P	GB Acc. COI	28S-D2	GB Acc. 28S	Institution Storing	Species	Types	Origin
SOWD068-06	BC-Hax2967	658[0n]	HM384103	-		Research Collection of Jean Haxaire	*Agrius convolvuli*		China, Yunnan
SOWD067-06	BC-Hax2966	658[0n]	HM384102	-		Research Collection of Jean Haxaire	*Agrius convolvuli*		France, Aquitaine
SPHYE007-08	BC-EST0007	658[0n]	JN677683	616[0n]	KJ168589	Research Collection of Yves Estradel	*Agrius convolvuli*		France, Midi-Pyrenees
SPHYE008-08	BC-EST0008	593[0n]	KJ168183	-		Research Collection of Yves Estradel	*Agrius convolvuli*		France, Midi-Pyrenees
SPHYE009-08	BC-EST0009	596[0n]	KJ168543	-		Research Collection of Yves Estradel	*Agrius convolvuli*		France, Midi-Pyrenees
SOWD070-06	BC-Hax2969	552[0n]	KJ168378	496[0n]	KJ168585	Research Collection of Jean Haxaire	*Agrius convolvuli*		French Polynesia, Society Islands
SOWD069-06	BC-Hax2968	608[0n]	KJ168088	576[0n]	KJ168570	Research Collection of Jean Haxaire	*Agrius convolvuli*		French Polynesia, Society Islands
MGABD574-11	Lope11-0669	658[0n]	KJ168459	-		University of Rouen, ECODIV Laboratory	*Agrius convolvuli*		Gabon, Ogooue-Ivindo
MGABD576-11	Lope11-0671	658[0n]	KJ168426	-		University of Rouen, ECODIV Laboratory	*Agrius convolvuli*		Gabon, Ogooue-Ivindo
MGABD575-11	Lope11-0670	658[0n]	KJ168438	-		University of Rouen, ECODIV Laboratory	*Agrius convolvuli*		Gabon, Ogooue-Ivindo
MGABD303-11	Lope11-0398	658[0n]	KJ168341	-		University of Rouen, ECODIV Laboratory	*Agrius convolvuli*		Gabon, Ogooue-Ivindo
MGABC547-10	TDGABb-0120	658[0n]	KJ168499	-		University of Rouen, ECODIV Laboratory	*Agrius convolvuli*		Gabon, Ogooue-Ivindo
MGABC545-10	TDGABb-0118	658[0n]	HQ992037	-		University of Rouen, ECODIV Laboratory	*Agrius convolvuli*		Gabon, Ogooue-Ivindo
MGABC546-10	TDGABb-0119	658[0n]	HQ992038	-		University of Rouen, ECODIV Laboratory	*Agrius convolvuli*		Gabon, Ogooue-Ivindo
GWOSU065-11	BC ZSM Lep 53316	658[0n]	KJ168122	-		Bavarian State Collection of Zoology	*Agrius convolvuli*		Germany, Bavaria
GWORA2543-09	BC ZSM Lep 31800	622[0n]	HM393240	-		Research Collection of Ralph Sturm	*Agrius convolvuli*		Germany, Bavaria
GWORA2544-09	BC ZSM Lep 31801	572[1n]	HM393241	-		Research Collection of Ralph Sturm	*Agrius convolvuli*		Germany, Bavaria
FBLMU936-09	BC ZSM Lep 27966	658[0n]	HQ955338	-		Bavarian State Collection of Zoology	*Agrius convolvuli*		Germany, Bavaria
GWORL289-09	BC ZSM Lep 22001	658[0n]	HM393627	-		Bavarian State Collection of Zoology	*Agrius convolvuli*		Germany, Bavaria
SPTMB083-09	BC-Mel 1095	658[0n]	GU704497	-		Research Collection of Tomas Melichar	*Agrius convolvuli*		India, Karnataka
SPHAP044-06	MA05-08-23-73	658[0n]	KJ168416	-		Research Collection of Morton Sam Adams	*Agrius convolvuli*		India, Maharashtra
SPUEB032-07	BC-EMEM0972	658[0n]	KJ168165	616[0n]	KJ168575	Entomologisches Museum Eitschberger	*Agrius convolvuli*		Indonesia, Maluku
PMANK028-06	USNM ENT 00196277	658[0n]	KJ168515	-		Smithsonian Institution	*Agrius convolvuli*		Kenya, Rift Valley
PMANK030-06	USNM ENT 00196279	658[0n]	KJ168366	-		Smithsonian Institution	*Agrius convolvuli*		Kenya, Rift Valley
PMANL809-11	USNM ENT 00719615	658[0n]	JN284555	-		Smithsonian Institution	*Agrius convolvuli*		Kenya, Rift Valley
PMANK031-06	USNM ENT 00196280	658[0n]	KJ168227	-		Smithsonian Institution	*Agrius convolvuli*		Kenya, Rift Valley
HKNHM131-07	HKNHM-730205	658[0n]	KJ168527	-		Natural History Museum, London	*Agrius convolvuli*		Madagascar
HKNHM186-07	HKNHM-794613	657[1n]	KJ168364	-		Natural History Museum, London	*Agrius convolvuli*		Madagascar
HKNHM036-07	HKNHM-740652	658[0n]	KJ168530	-		Natural History Museum, London	*Agrius convolvuli*		Madagascar
SPPBA360-07	BC-Basq0361	609[0n]	KJ168264	616[0n]	KJ168580	Research Collection of Patrick Basquin	*Agrius convolvuli*		Madagascar, Fianarantsoa
SPUEB034-07	BC-EMEM0974	658[0n]	KJ168539	606[0n]	KJ168595	Entomologisches Museum Eitschberger	*Agrius convolvuli*		New Caledonia
PMANK053-06	USNM ENT 00196480	658[0n]	KJ168335	-		Smithsonian Institution	*Agrius convolvuli*		Nigeria, Oyo
PMANK054-06	USNM ENT 00196481	658[0n]	KJ168300	-		Smithsonian Institution	*Agrius convolvuli*		Nigeria, Oyo
PMANK050-06	USNM ENT 00196477	658[0n]	KJ168271	-		Smithsonian Institution	*Agrius convolvuli*		Nigeria, Oyo
PMANK052-06	USNM ENT 00196479	658[0n]	KJ168363	-		Smithsonian Institution	*Agrius convolvuli*		Nigeria, Oyo
PMANK051-06	USNM ENT 00196478	658[0n]	KJ168340	-		Smithsonian Institution	*Agrius convolvuli*		Nigeria, Oyo

Process ID	Sample ID	COI-5P	GB Acc. COI	28S-D2	GB Acc. 28S	Institution Storing	Species	Types	Origin
PMANK055-06	USNM ENT 00196482	658[0n]	KJ168169	-		Smithsonian Institution	*Agrius convolvuli*		Nigeria, Oyo
SPTOL196-07	MF-05-0017	658[0n]	KJ168310	-		University of Maryland	*Agrius convolvuli*		Palau
GWORB988-07	BC ZSM Lep 02210	588[1n]	KJ168514	-		Bavarian State Collection of Zoology	*Agrius convolvuli*		Papua New Guinea, Central
HCPN009-03	USNM ENT 00678977	601[0n]	KJ168217	-		Smithsonian Institution	*Agrius convolvuli*		Papua New Guinea, Madang
GWOTG693-12	BC ZSM Lep 65819	658[0n]	KJ168380	-		Bavarian State Collection of Zoology	*Agrius convolvuli*		South Africa, Gauteng
GWOSV051-11	BC ZSM Lep 44277	658[0n]	KJ168357	-		Bavarian State Collection of Zoology	*Agrius convolvuli*		Taiwan, Hualien City
GWOSV046-11	BC ZSM Lep 44272	637[0n]	KJ168247	-		Bavarian State Collection of Zoology	*Agrius convolvuli*		Taiwan, Hualien City
GWOSV050-11	BC ZSM Lep 44276	658[0n]	KJ168185	-		Bavarian State Collection of Zoology	*Agrius convolvuli*		Taiwan, Nantou County
GWOSV048-11	BC ZSM Lep 44274	658[0n]	KJ168266	-		Bavarian State Collection of Zoology	*Agrius convolvuli*		Taiwan, Nantou County
GWOSV049-11	BC ZSM Lep 44275	658[0n]	KJ168505	-		Bavarian State Collection of Zoology	*Agrius convolvuli*		Taiwan, Nantou County
GWOSV047-11	BC ZSM Lep 44273	568[0n]	KJ168225	-		Bavarian State Collection of Zoology	*Agrius convolvuli*		Taiwan, Nantou County
GWOSC595-10	BC ZSM Lep 36366	658[0n]	KJ168115	-		Bavarian State Collection of Zoology	*Agrius convolvuli*		Taiwan, Nantou County
SPTOL099-07	AYK-04-0298	658[0n]	KJ168382	-		University of Maryland	*Agrius convolvuli*		Taiwan, Pingtung County
SPTOL134-07	AYK-04-0289	658[0n]	KJ168259	-		University of Maryland	*Agrius convolvuli*		Taiwan, Taidong County
SPTOL130-07	AYK-04-0284	658[0n]	KJ168470	-		University of Maryland	*Agrius convolvuli*		Taiwan, Taidong County
SSDA224-06	PD-BC 036	657[1n]	KJ168425	615[0n]	KJ168587	Research Collection of Philippe Darge	*Agrius convolvuli*		Tanzania
SPPDA001-07	PD-BC 381a	658[0n]	KJ168328	585[1n]	KJ168582	Research Collection of Philippe Darge	*Agrius convolvuli*		Tanzania, Iringa
SPTOL192-07	IJK-03-3183	658[0n]	KJ168256	-		University of Maryland	*Agrius convolvuli*		Tanzania, Pwani
SPPDA091-07	PD-BC 471	658[0n]	KJ168157	615[0n]	KJ168572	Research Collection of Philippe Darge	*Agrius convolvuli*		Tanzania, Rukwa
HKNHM168-07	HKNHM-794590	658[0n]	KJ168119	-		Natural History Museum, London	*Agrius convolvuli*		United Kingdom,
SPHAP070-06	MA06-01-06-02	658[0n]	KJ168067	-		Research Collection of Morton Sam Adams	*Agrius convolvuli*		Zambia, Copperbelt
SPHAP071-06	MA06-01-06-03	658[0n]	KJ168497	-		Research Collection of Morton Sam Adams	*Agrius convolvuli*		Zambia, Copperbelt
SPHAP072-06	MA06-01-06-04	658[0n]	KJ168387	-		Research Collection of Morton Sam Adams	*Agrius convolvuli*		Zambia, Copperbelt
SPRBA1030-09	BC-RBP-2138	647[0n]	KJ168501	-		Research Collection of Ron Brechlin	*Ambulyx dohertyi*		Indonesia, Papua
SPRBA1031-09	BC-RBP-2139	621[0n]	KJ168441	-		Research Collection of Ron Brechlin	*Ambulyx dohertyi*		Indonesia, Papua
SPRBA1032-09	BC-RBP-2140	609[0n]	KJ168270	-		Research Collection of Ron Brechlin	*Ambulyx dohertyi*		Papua New Guinea, Morobe
SPRBA1029-09	BC-RBP-2137	658[0n]	KJ168493	-		Research Collection of Ron Brechlin	*Ambulyx dohertyi*		Papua New Guinea, Morobe
SPRBA1043-09	BC-RBP-2151	658[0n]	KJ168532	-		Research Collection of Ron Brechlin	*Ambulyx dohertyi novobritannica*	Holotype	Papua New Guinea, West New Britain
SPRBA1044-09	BC-RBP-2152	658[0n]	KJ168148	-		Research Collection of Ron Brechlin	*Ambulyx dohertyi novobritannica*	Paratype	Papua New Guinea, West New Britain
SPRBA1041-09	BC-RBP-2149	658[0n]	GU704371	-		Research Collection of Ron Brechlin	*Ambulyx dohertyi novoirlandensis*	Holotype	Papua New Guinea, New Ireland
SPRBA1042-09	BC-RBP-2150	658[0n]	KJ168075	-		Research Collection of Ron Brechlin	*Ambulyx dohertyi novoirlandensis*	Paratype	Papua New Guinea, New Ireland
SOWA191-06	BC-Hax0191	572[0n]	KJ168379	-		Research Collection of Jean Haxaire	*Ambulyx dohertyi queenslandi*		Papua New Guinea
SPRBA1034-09	BC-RBP-2142	642[0n]	KJ168312	-		Research Collection of Ron Brechlin	*Ambulyx dohertyi queenslandi*		Papua New Guinea, Ferguson island
SPRBA1033-09	BC-RBP-2141	658[0n]	KJ168279	-		Research Collection of Ron Brechlin	*Ambulyx dohertyi queenslandi*		Papua New Guinea, Ferguson island

Process ID	Sample ID	COI-5P	GB Acc. COI	28S-D2	GB Acc. 28S	Institution Storing	Species	Types	Origin
SPRBA1035-09	BC-RBP-2143	658[0n]	KJ168254	-		Research Collection of Ron Brechlin	*Ambulyx dohertyi queenslandi*		Papua New Guinea, Goodenough island
HCPN055-03	USNM ENT 00196061	618[0n]	KJ168339	-		Smithsonian Institution	*Ambulyx dohertyi queenslandi*		Papua New Guinea, Gulf
HCPN054-03	USNM ENT 00196046	423[1n]	KJ168308	-		Smithsonian Institution	*Ambulyx dohertyi queenslandi*		Papua New Guinea, Gulf
HCPN056-03	USNM ENT 00196062	627[0n]	KJ168251	-		Smithsonian Institution	*Ambulyx dohertyi queenslandi*		Papua New Guinea, Gulf
SPRBA1037-09	BC-RBP-2145	658[0n]	KJ168388	-		Research Collection of Ron Brechlin	*Ambulyx dohertyi salomonis*		Solomon Islands, Guadalcanal
SPRBA1036-09	BC-RBP-2144	585[0n]	KJ168260	-		Research Collection of Ron Brechlin	*Ambulyx dohertyi salomonis*		Solomon Islands, Guadalcanal
SPRBA1040-09	BC-RBP-2148	658[0n]	KJ168216	-		Research Collection of Ron Brechlin	*Ambulyx dohertyi salomonis*		Solomon Islands, San Cristobal
SPRBA1039-09	BC-RBP-2147	658[0n]	KJ168547	-		Research Collection of Ron Brechlin	*Ambulyx dohertyi salomonis*		Solomon Islands, San Cristobal
SPRBA1038-09	BC-RBP-2146	658[0n]	KJ168108	-		Research Collection of Ron Brechlin	*Ambulyx dohertyi salomonis*		Solomon Islands, Santa Isabel
SPRBA763-09	BC-RBP-1811	658[0n]	GU704172	-		Research Collection of Ron Brechlin	*Ambulyx wildei*		Indonesia, Papua
SPRBA764-09	BC-RBP-1812	658[0n]	GU704171	-		Research Collection of Ron Brechlin	*Ambulyx wildei*		Indonesia, Papua
SPUEA595-07	BC-EMEM0595	658[0n]	KJ168128	-		Entomologisches Museum Eitschberger	*Ambulyx wildei*		Indonesia, Papua
SPUEA594-07	BC-EMEM0594	658[0n]	KJ168242	-		Entomologisches Museum Eitschberger	*Ambulyx wildei*		Indonesia, Papua
SPUEA593-07	BC-EMEM0593	658[0n]	KJ168496	-		Entomologisches Museum Eitschberger	*Ambulyx wildei*		Indonesia, Papua
SPRBA765-09	BC-RBP-1813	658[0n]	GU704173	-		Research Collection of Ron Brechlin	*Ambulyx wildei*		Papua New Guinea, Ferguson island
CATS436-10	USNM ENT 00704738	658[0n]	HM906492	-		Smithsonian Institution	*Ambulyx wildei*		Papua New Guinea, Madang
CATS410-10	USNM ENT 00704681	658[0n]	HM906468	-		Smithsonian Institution	*Ambulyx wildei*		Papua New Guinea, Madang
SOWA192-06	BC-Hax0192	608[0n]	KJ168086	-		Research Collection of Jean Haxaire	*Ambulyx wildei*		Papua New Guinea, Morobe
SPTMA043-07	BC-Mel 0121b	654[3n]	KJ168315	-		Research Collection of Tomas Melichar	*Angonyx papuana*		Indonesia, Papua
SPTMC521-12	BC-Mel2522	658[0n]	KJ168512	-		Research Collection of Tomas Melichar	*Angonyx papuana*		Indonesia, West Papua
SPTMC520-12	BC-Mel2521	633[0n]	KJ168230	-		Research Collection of Tomas Melichar	*Angonyx papuana*		Indonesia, West Papua
SARBB1435-10	BC-RBP-2567.1	658[0n]	HQ973206	-		Research Collection of Ron Brechlin	*Angonyx papuana*		Papua New Guinea, New Ireland
SARBB1434-10	BC-RBP-2566.1	658[0n]	HQ973205	-		Research Collection of Ron Brechlin	*Angonyx papuana*		Papua New Guinea, New Ireland
SARBB1433-10	BC-RBP-2565.1	658[0n]	HQ973204	-		Research Collection of Ron Brechlin	*Angonyx papuana*		Papua New Guinea, West New Britain
SARBB1432-10	BC-RBP-2564.1	658[0n]	HQ973203	-		Research Collection of Ron Brechlin	*Angonyx papuana*		Papua New Guinea, West New Britain
SPUEB201-07	BC-EMEM1141	658[0n]	KJ168398	-		Entomologisches Museum Eitschberger	*Cephonodes hylas*		Japan
SPTOL113-07	AYK-04-0171	658[0n]	KJ168407	-		University of Maryland	*Cephonodes hylas*		Taiwan, Nantou County
SPUEB202-07	BC-EMEM1142	658[0n]	KJ168237	-		Entomologisches Museum Eitschberger	*Cephonodes hylas*		Thailand, Chiang Mai
SPUEB203-07	BC-EMEM1143	658[0n]	KJ168419	-		Entomologisches Museum Eitschberger	*Cephonodes hylas*		Thailand, Chiang Mai
SPUEB200-07	BC-EMEM1140	658[0n]	JN677805	-		Entomologisches Museum Eitschberger	*Cephonodes hylas*		Vietnam
SPUEB197-07	BC-EMEM1137	658[0n]	KJ168307	-		Entomologisches Museum Eitschberger	*Cephonodes hylas melanogaster*		Indonesia, Sulawesi Barat
SPUEB196-07	BC-EMEM1136	609[0n]	KJ168135	-		Entomologisches Museum Eitschberger	*Cephonodes hylas melanogaster*	Paratype	Indonesia, Sulawesi Selatan
SPUEB198-07	BC-EMEM1138	658[0n]	KJ168531	-		Entomologisches Museum Eitschberger	*Cephonodes hylas melanogaster*		Indonesia, Sulawesi Selatan

Process ID	Sample ID	COI-5P	GB Acc. COI	28S-D2	GB Acc. 28S	Institution Storing	Species	Types	Origin
SPTMB923-11	BC-Mel1925	658[0n]	JN281271	-		Research Collection of Tomas Melichar	*Cephonodes hylas virescens*		Central African Republic
SPTMB922-11	BC-Mel1924	658[0n]	JN281270	-		Research Collection of Tomas Melichar	*Cephonodes hylas virescens*		Central African Republic
SOWC449-06	BC-Hax2348	608[0n]	KJ168369	-		Research Collection of Jean Haxaire	*Cephonodes hylas virescens*		Chad
SOWC450-06	BC-Hax2349	608[0n]	KJ168267	-		Research Collection of Jean Haxaire	*Cephonodes hylas virescens*		Chad
MGABD907-11	Lope11-1002	658[0n]	KJ168201	-		University of Rouen, ECODIV Laboratory	*Cephonodes hylas virescens*		Gabon, Ogooue-Ivindo
MGABD650-11	Lope11-0745	658[0n]	KJ168540	-		University of Rouen, ECODIV Laboratory	*Cephonodes hylas virescens*		Gabon, Ogooue-Ivindo
MGABD651-11	Lope11-0746	658[0n]	KJ168534	-		University of Rouen, ECODIV Laboratory	*Cephonodes hylas virescens*		Gabon, Ogooue-Ivindo
MGABC539-10	TDGABb-112	658[0n]	HQ573985	-		University of Rouen, ECODIV Laboratory	*Cephonodes hylas virescens*		Gabon, Ogooue-Ivindo
PMANL1483-11	USNM ENT 00719779	658[0n]	KJ168563	-		Smithsonian Institution	*Cephonodes hylas virescens*		Kenya
SPTOL018-07	IJK-02-5931	658[0n]	KJ168471	-		University of Maryland	*Cephonodes hylas virescens*		
SOWC443-06	BC-Hax2342	605[0n]	KJ168085	-		Research Collection of Jean Haxaire	*Cephonodes picus*		India, Pondicherry
SOWC444-06	BC-Hax2343	608[0n]	KJ168199	-		Research Collection of Jean Haxaire	*Cephonodes picus*		India, Pondicherry
LTOL262-07	MF-05-0013	658[0n]	KJ168490	-		University of Maryland	*Cephonodes picus*		Palau
SPUEB206-07	BC-EMEM1146	658[0n]	JN677808	-		Entomologisches Museum Eitschberger	*Cephonodes picus*		Philippines
SPUEB204-07	BC-EMEM1144	658[0n]	KJ168129	-		Entomologisches Museum Eitschberger	*Cephonodes picus*		Philippines
SPUEB205-07	BC-EMEM1145	658[0n]	KJ168424	-		Entomologisches Museum Eitschberger	*Cephonodes picus*		Philippines
SOWB341-06	BC-Hax1334	658[0n]	JN677867	-		Research Collection of Jean Haxaire	*Daphnis dohertyi*		Indonesia, Papua
SOWB340-06	BC-Hax1333	658[0n]	KJ168393	-		Research Collection of Jean Haxaire	*Daphnis dohertyi*		Papua New Guinea, Morobe
SOWB339-06	BC-Hax1332	598[7n]	KJ168556	-		Research Collection of Jean Haxaire	*Daphnis dohertyi*		Papua New Guinea, Morobe
SPTMB409-10	BC-Mel1411	658[0n]	HQ581114	-		Research Collection of Tomas Melichar	*Daphnis hypothous crameri*		India, Karnataka
SPTMB086-09	BC-Mel 1098	658[0n]	GU704492	-		Research Collection of Tomas Melichar	*Daphnis hypothous crameri*		India, Karnataka
SPTMB408-10	BC-Mel1410	658[0n]	HQ581113	-		Research Collection of Tomas Melichar	*Daphnis hypothous crameri*		India, Karnataka
SPTMB407-10	BC-Mel1409	639[0n]	KJ168189	-		Research Collection of Tomas Melichar	*Daphnis hypothous crameri*		India, Karnataka
SPTMB406-10	BC-Mel1408	658[0n]	HQ581112	-		Research Collection of Tomas Melichar	*Daphnis hypothous crameri*		India, Karnataka
SATWB106-08	BC-Roug1154	658[0n]	JN677869	-		Research Collection of Jean Haxaire	*Daphnis hypothous crameri*		Vietnam
GWORB977-07	BC ZSM Lep 02199	593[1n]	KJ168334	-		Bavarian State Collection of Zoology	*Daphnis moorei*		Indonesia, Papua
GWORB976-07	BC ZSM Lep 02198	573[0n]	KJ168081	-		Bavarian State Collection of Zoology	*Daphnis moorei*		Indonesia, Papua
HCPN003-03	USNM ENT 00678975	639[0n]	KJ168408	-		Smithsonian Institution	*Daphnis moorei*		Papua New Guinea, Madang
HCPN004-03	USNM ENT 00678970	597[8n]	KJ168168	-		Smithsonian Institution	*Daphnis moorei*		Papua New Guinea, Madang
HCPN002-03	USNM ENT 00678979	590[0n]	KJ168145	-		Smithsonian Institution	*Daphnis moorei*		Papua New Guinea, Madang
SPTMA488-07	BC-Mel 0566	658[0n]	KJ168457	-		Research Collection of Tomas Melichar	*Daphnis moorei*		Solomon Islands, Guadalcanal
SPTMC437-12	BC-Mel2438	658[0n]	KJ168337	-		Research Collection of Tomas Melichar	*Daphnis placida*		India, Diglipur
SPTMC438-12	BC-Mel2439	658[0n]	KJ168421	-		Research Collection of Tomas Melichar	*Daphnis placida*		India, Diglipur
SPTMC439-12	BC-Mel2440	658[0n]	KJ168450	-		Research Collection of Tomas Melichar	*Daphnis placida*		India, Diglipur
SPTMC374-12	BC-Mel2375	658[0n]	KJ168211	-		Research Collection of Tomas Melichar	*Daphnis placida*		Indonesia, Maluku
SOWB338-06	BC-Hax1331	651[0n]	KJ168463	-		Research Collection of Jean Haxaire	*Daphnis placida*		Indonesia, Maluku

Process ID	Sample ID	COI-5P	GB Acc. COI	28S-D2	GB Acc. 28S	Institution Storing	Species	Types	Origin
SOWB337-06	BC-Hax1330	658[0n]	JN677872	-		Research Collection of Jean Haxaire	*Daphnis placida*		Indonesia, Maluku
SPTMC440-12	BC-Mel2441	658[0n]	KJ168153	-		Research Collection of Tomas Melichar	*Daphnis placida*		Indonesia, Nusa Tenggara Barat
SPTMC504-12	BC-Mel2505	571[0n]	KJ168181	-		Research Collection of Tomas Melichar	*Daphnis placida*		Indonesia, Nusa Tenggara Barat
SPTMC375-12	BC-Mel2376	658[0n]	KJ168545	-		Research Collection of Tomas Melichar	*Daphnis placida*		Indonesia, Nusa Tenggara Timur
SPTMC372-12	BC-Mel2373	658[0n]	KJ168178	-		Research Collection of Tomas Melichar	*Daphnis placida*		Indonesia, Papua
SPTMC371-12	BC-Mel2372	658[0n]	KJ168523	-		Research Collection of Tomas Melichar	*Daphnis placida*		Indonesia, Papua
SOWB336-06	BC-Hax1329	607[0n]	KJ168091	-		Research Collection of Jean Haxaire	*Daphnis placida*		Malaysia, Sabah
SPTMC373-12	BC-Mel2374	658[0n]	KJ168427	-		Research Collection of Tomas Melichar	*Daphnis placida*		New Caledonia
SPTMA073-07	BC-Mel 0151	653[0n]	KJ168482	-		Research Collection of Tomas Melichar	*Daphnis placida salomonis*		Solomon Islands, Guadalcanal
SPUEB224-07	BC-EMEM1164	658[0n]	KJ168468	-		Entomologisches Museum Eitschberger	*Daphnis protrudens*		Indonesia, Maluku
SPUEB225-07	BC-EMEM1165	658[0n]	KJ168522	-		Entomologisches Museum Eitschberger	*Daphnis protrudens*		Indonesia, Papua
HCPN005-03	USNM ENT 00678976	595[0n]	KJ168568	-		Smithsonian Institution	*Daphnis protrudens*		Papua New Guinea, Madang
SOWB389-06	BC-Hax1382	657[0n]	KJ168215	-		Research Collection of Jean Haxaire	*Daphnis protrudens lecourti*		Indonesia, Sulawesi Barat
SOWB390-06	BC-Hax1383	631[0n]	KJ168346	-		Research Collection of Jean Haxaire	*Daphnis protrudens lecourti*		Indonesia, Sulawesi Selatan
SPRBA559-09	BC-RBP-1217	658[0n]	HM432640	-		Research Collection of Ron Brechlin	*Eupanacra splendens*		Indonesia, Maluku
SOWC197-06	BC-Mel0028	658[0n]	KJ168161	601[0n]	KJ168574	Research Collection of Tomas Melichar	*Eupanacra splendens*		Indonesia, Papua
SPRBA557-09	BC-RBP-1215	658[0n]	GU704380	-		Research Collection of Ron Brechlin	*Eupanacra splendens*		Indonesia, Papua
SPRBA560-09	BC-RBP-1218	658[0n]	GU704381	-		Research Collection of Ron Brechlin	*Eupanacra splendens*		Papua New Guinea, Ferguson island
EPNG3395-11	USNM ENT 00697249	658[0n]	JN280899	-		Smithsonian Institution	*Eupanacra splendens*		Papua New Guinea, Madang
PMANL1971-12	USNM ENT 00697248	658[0n]	KJ168368	-		Smithsonian Institution	*Eupanacra splendens*		Papua New Guinea, Madang
PMANL1970-12	USNM ENT 00510594	658[0n]	KJ168150	-		Smithsonian Institution	*Eupanacra splendens*		Papua New Guinea, Madang
EPNG3394-11	USNM ENT 00697250	614[0n]	JN280898	-		Smithsonian Institution	*Eupanacra splendens*		Papua New Guinea, Madang
EPNG3393-11	USNM ENT 00510595	658[0n]	JN280897	-		Smithsonian Institution	*Eupanacra splendens*		Papua New Guinea, Madang
SPRBA561-09	BC-RBP-1219	658[0n]	HM432641	-		Research Collection of Ron Brechlin	*Eupanacra splendens*		Papua New Guinea, West New Britain
SPRBA563-09	BC-RBP-1221	307[0n]	KJ168343	-		Research Collection of Ron Brechlin	*Eupanacra splendens*		Solomon Islands, Guadalcanal
SPRBA564-09	BC-RBP-1222	658[0n]	HM432642	-		Research Collection of Ron Brechlin	*Eupanacra splendens*		Solomon Islands, Western
SPTMB579-11	BC-Mel1581	658[0n]	JN281130	-		Research Collection of Tomas Melichar	*Eupanacra splendens paradoxa*		Indonesia, Maluku
SPTMB047-09	BC-Mel 1059	658[0n]	HM432643	-		Research Collection of Tomas Melichar	*Eupanacra splendens paradoxa*		Indonesia, Maluku
SPHYE209-09	BC-EST0585	658[0n]	GU703969	-		Research Collection of Yves Estradel	*Gnathothlibus eras*		French Polynesia, Bora Bora
SOWB425-06	BC-Hax1418	608[0n]	JX438284	602[0n]	JX438297	Research Collection of Jean Haxaire	*Gnathothlibus eras*		French Polynesia, Society Islands
SOWB423-06	BC-Hax1416	658[0n]	HM384048	-		Research Collection of Jean Haxaire	*Gnathothlibus eras*		French Polynesia, Society Islands
SOWB422-06	BC-Hax1415	658[0n]	JX438289	602[0n]	JX438300	Research Collection of Jean Haxaire	*Gnathothlibus eras*		French Polynesia, Society Islands
SOWB408-06	BC-Hax1401	609[0n]	JX438287	-		Research Collection of Jean Haxaire	*Gnathothlibus eras*		Indonesia, Sulawesi Tengah
SOWB410-06	BC-Hax1403	607[0n]	JX438291	-		Research Collection of Jean Haxaire	*Gnathothlibus eras*		Indonesia, Sulawesi Tengah
SOWB409-06	BC-Hax1402	609[0n]	JX438285	-		Research Collection of Jean Haxaire	*Gnathothlibus eras*		Indonesia, Sulawesi Tengah

Process ID	Sample ID	COI-5P	GB Acc. COI	28S-D2	GB Acc. 28S	Institution Storing	Species	Types	Origin
SPTMB694-11	BC-Mel1696	658[0n]	KJ168352	-		Research Collection of Tomas Melichar	*Gnathothlibus eras*		New Caledonia
SOWB421-06	BC-Hax1414	609[0n]	KJ168491	602[0n]	KJ168592	Research Collection of Jean Haxaire	*Gnathothlibus eras*		New Caledonia, South
SPTMC562-12	BC-Mel2563	658[0n]	KJ168402	-		Research Collection of Tomas Melichar	*Hippotion brennus*		Indonesia, Maluku
SPTMB494-11	BC-Mel1496	658[0n]	JN281071	-		Research Collection of Tomas Melichar	*Hippotion brennus*		Indonesia, Maluku
SPTMB507-11	BC-Mel1509	658[0n]	JN281080	-		Research Collection of Tomas Melichar	*Hippotion brennus*		Indonesia, Maluku
SPTMB506-11	BC-Mel1508	658[0n]	JN281079	-		Research Collection of Tomas Melichar	*Hippotion brennus*		Indonesia, Maluku
SPTMB510-11	BC-Mel1512	658[0n]	JN281083	-		Research Collection of Tomas Melichar	*Hippotion brennus*		Indonesia, Maluku
SPTMB508-11	BC-Mel1510	658[0n]	JN281081	-		Research Collection of Tomas Melichar	*Hippotion brennus*		Indonesia, Maluku
SPTMB511-11	BC-Mel1513	658[0n]	JN281084	-		Research Collection of Tomas Melichar	*Hippotion brennus*		Indonesia, Maluku
SPTMB499-11	BC-Mel1501	658[0n]	JN281076	-		Research Collection of Tomas Melichar	*Hippotion brennus*		Indonesia, Maluku
SPTMB493-11	BC-Mel1495	658[0n]	JN281070	-		Research Collection of Tomas Melichar	*Hippotion brennus*		Indonesia, Maluku
SPTMB504-11	BC-Mel1506	658[0n]	JN281077	-		Research Collection of Tomas Melichar	*Hippotion brennus*		Indonesia, Maluku
SPTMB491-11	BC-Mel1493	658[0n]	JN281068	-		Research Collection of Tomas Melichar	*Hippotion brennus*		Indonesia, Maluku
SPTMB492-11	BC-Mel1494	658[0n]	JN281069	-		Research Collection of Tomas Melichar	*Hippotion brennus*		Indonesia, Maluku
SPTMB505-11	BC-Mel1507	658[0n]	JN281078	-		Research Collection of Tomas Melichar	*Hippotion brennus*		Indonesia, Maluku
SPTMB509-11	BC-Mel1511	658[0n]	JN281082	-		Research Collection of Tomas Melichar	*Hippotion brennus*		Indonesia, Maluku
SPTMB497-11	BC-Mel1499	658[0n]	JN281074	-		Research Collection of Tomas Melichar	*Hippotion brennus*		Indonesia, Papua
SPTMB496-11	BC-Mel1498	658[0n]	JN281073	-		Research Collection of Tomas Melichar	*Hippotion brennus*		Indonesia, Papua
SPTMB495-11	BC-Mel1497	658[0n]	JN281072	-		Research Collection of Tomas Melichar	*Hippotion brennus*		Indonesia, Papua
SPTMA924-09	BC-Mel 1002	631[0n]	KJ168121	-		Research Collection of Tomas Melichar	*Hippotion brennus*		Indonesia, Papua
SPTMA197-07	BC-Mel 0275	658[0n]	KJ168083	599[0n]	KJ168569	Research Collection of Tomas Melichar	*Hippotion brennus*		Indonesia, Papua
SPTMA923-09	BC-Mel 1001	658[0n]	KJ168437	-		Research Collection of Tomas Melichar	*Hippotion brennus*		Indonesia, Papua
SOWE237-07	BC-Hax4136	609[0n]	KJ168277	-		Research Collection of Jean Haxaire	*Hippotion brennus*		Indonesia, Papua
GWORB980-07	BC ZSM Lep 02202	637[0n]	KJ168304	-		Bavarian State Collection of Zoology	*Hippotion brennus*		Indonesia, Papua
SPTMB498-11	BC-Mel1500	658[0n]	JN281075	-		Research Collection of Tomas Melichar	*Hippotion brennus*		Indonesia, Papua Barat
SPTOL159-07	AYK-04-0398	658[0n]	KJ168126	-		University of Maryland	*Hippotion brennus*		Papua New Guinea, Chimbu
SML161-06	USNM ENT 00196616	642[0n]	KJ168133	599[0n]	KJ168571	Smithsonian Institution	*Hippotion brennus*		Papua New Guinea, Gulf
HCPN007-03	USNM ENT 00678973	584[1n]	KJ168160	599[0n]	KJ168573	Smithsonian Institution	*Hippotion brennus*		Papua New Guinea, Madang
HCPN008-03	USNM ENT 00678972	637[2n]	KJ168373	-		Smithsonian Institution	*Hippotion brennus*		Papua New Guinea, Madang
HCPN006-03	USNM ENT 00678974	600[0n]	KJ168233	599[0n]	KJ168578	Smithsonian Institution	*Hippotion brennus*		Papua New Guinea, Madang
SML160-06	USNM ENT 00196615	658[0n]	KJ168403	599[0n]	KJ168586	Smithsonian Institution	*Hippotion brennus*		Papua New Guinea, Morobe
SML159-06	USNM ENT 00196614	658[0n]	KJ168229	563[34n]	KJ168577	Smithsonian Institution	*Hippotion brennus*		Papua New Guinea, Morobe
SPTMB905-11	BC-Mel1907	658[0n]	JN281257	-		Research Collection of Tomas Melichar	*Hippotion celerio*		Central African Republic
SPTMB904-11	BC-Mel1906	658[0n]	JN281256	-		Research Collection of Tomas Melichar	*Hippotion celerio*		Central African Republic
SPHYE114-09	BC-EST0490	640[0n]	GU703888	-		Research Collection of Yves Estradel	*Hippotion celerio*		Gabon, Haut-Ogooue
MGABD800-11	Lope11-0895	658[0n]	KJ168564	-		University of Rouen, ECODIV Laboratory	*Hippotion celerio*		Gabon, Ogooue-Ivindo

Process ID	Sample ID	COI-5P	GB Acc. COI	28S-D2	GB Acc. 28S	Institution Storing	Species	Types	Origin
SPTMB557-11	BC-Mel1559	658[0n]	JN281119	-		Research Collection of Tomas Melichar	*Hippotion celerio*		Indonesia, Bali
SPTMB556-11	BC-Mel1558	658[0n]	JN281118	-		Research Collection of Tomas Melichar	*Hippotion celerio*		Indonesia, Bali
SPTMB554-11	BC-Mel1556	658[0n]	JN281116	-		Research Collection of Tomas Melichar	*Hippotion celerio*		Indonesia, Bangka-Belitung Islands
SPTMB555-11	BC-Mel1557	658[0n]	JN281117	-		Research Collection of Tomas Melichar	*Hippotion celerio*		Indonesia, Nusa Tenggara Timur
SPTMB551-11	BC-Mel1553	658[0n]	JN281113	-		Research Collection of Tomas Melichar	*Hippotion celerio*		Indonesia, Nusa Tenggara Timur
SPTMB550-11	BC-Mel1552	658[0n]	JN281112	-		Research Collection of Tomas Melichar	*Hippotion celerio*		Indonesia, Nusa Tenggara Timur
SPTMB558-11	BC-Mel1560	658[0n]	JN281120	-		Research Collection of Tomas Melichar	*Hippotion celerio*		Indonesia, Papua Barat
SPTMB559-11	BC-Mel1561	658[0n]	JN281121	-		Research Collection of Tomas Melichar	*Hippotion celerio*		Indonesia, Papua Barat
SPTMB552-11	BC-Mel1554	658[0n]	JN281114	-		Research Collection of Tomas Melichar	*Hippotion celerio*		Indonesia, Sumatera Barat
SPTMB553-11	BC-Mel1555	658[0n]	JN281115	-		Research Collection of Tomas Melichar	*Hippotion celerio*		Indonesia, Sumatera Selatan
PMANK049-06	USNM ENT 00196451	658[0n]	KJ168445	-		Smithsonian Institution	*Hippotion celerio*		Kenya, Rift Valley
PMANK019-06	USNM ENT 00196027	658[0n]	KJ168176	-		Smithsonian Institution	*Hippotion celerio*		Kenya, Rift Valley
PMANK022-06	USNM ENT 00196271	658[0n]	KJ168385	-		Smithsonian Institution	*Hippotion celerio*		Kenya, Rift Valley
PMANK023-06	USNM ENT 00196272	658[0n]	KJ168208	-		Smithsonian Institution	*Hippotion celerio*		Kenya, Rift Valley
PMANK048-06	USNM ENT 00196450	658[0n]	KJ168342	-		Smithsonian Institution	*Hippotion celerio*		Kenya, Rift Valley
HCKV008-03	USNM ENT 00196104	658[0n]	KJ168123	-		Smithsonian Institution	*Hippotion celerio*		Kenya, Rift Valley
PMANK016-06	USNM ENT 00196011	658[0n]	KJ168131	-		Smithsonian Institution	*Hippotion celerio*		Kenya, Rift Valley
PMANK021-06	USNM ENT 00196270	658[0n]	KJ168423	-		Smithsonian Institution	*Hippotion celerio*		Kenya, Rift Valley
HCKV009-03	USNM ENT 00196105	595[0n]	KJ168198	-		Smithsonian Institution	*Hippotion celerio*		Kenya, Rift Valley
PMANK017-06	USNM ENT 00196012	658[0n]	KJ168516	-		Smithsonian Institution	*Hippotion celerio*		Kenya, Rift Valley
PMANK014-06	USNM ENT 00196006	658[0n]	KJ168209	-		Smithsonian Institution	*Hippotion celerio*		Kenya, Rift Valley
PMANK015-06	USNM ENT 00196010	658[0n]	KJ168294	-		Smithsonian Institution	*Hippotion celerio*		Kenya, Rift Valley
PMANK020-06	USNM ENT 00196028	658[0n]	KJ168549	-		Smithsonian Institution	*Hippotion celerio*		Kenya, Rift Valley
PMANK047-06	USNM ENT 00196449	658[0n]	KJ168273	-		Smithsonian Institution	*Hippotion celerio*		Kenya, Rift Valley
HKNHM045-07	HKNHM-740662	658[0n]	KJ168218	-		Natural History Museum, London	*Hippotion celerio*		Madagascar
HKNHM150-07	HKNHM-794572	658[0n]	KJ168291	-		Natural History Museum, London	*Hippotion celerio*		Madagascar
HKNHM153-07	HKNHM-794575	658[0n]	KJ168284	-		Natural History Museum, London	*Hippotion celerio*		Madagascar
HKNHM043-07	HKNHM-740660	658[0n]	KJ168452	-		Natural History Museum, London	*Hippotion celerio*		Madagascar
SPMNP290-07	BC-MNHNJP0180	658[0n]	JN678018	-		Museum National d'Histoire Naturelle, Paris	*Hippotion celerio*		Malawi
SOWF097-12	BC-Hax4955	658[0n]	KJ168485	-		Research Collection of Jean Haxaire	*Hippotion celerio*		New Caledonia, South
PMANK099-06	USNM ENT 00196503	658[0n]	KJ168384	-		Smithsonian Institution	*Hippotion celerio*		Nigeria, Oyo
PMANK101-06	USNM ENT 00196505	658[0n]	KJ168155	-		Smithsonian Institution	*Hippotion celerio*		Nigeria, Oyo
PMANK100-06	USNM ENT 00196504	658[0n]	KJ168105	-		Smithsonian Institution	*Hippotion celerio*		Nigeria, Oyo
PMANK103-06	USNM ENT 00196507	658[0n]	KJ168495	-		Smithsonian Institution	*Hippotion celerio*		Nigeria, Oyo
PMANK104-06	USNM ENT 00196508	658[0n]	KJ168286	-		Smithsonian Institution	*Hippotion celerio*		Nigeria, Oyo

Process ID	Sample ID	COI-5P	GB Acc. COI	28S-D2	GB Acc. 28S	Institution Storing	Species	Types	Origin
PMANK102-06	USNM ENT 00196506	658[0n]	KJ168430	-		Smithsonian Institution	*Hippotion celerio*		Nigeria, Oyo
GWORB989-07	BC ZSM Lep 02211	605[1n]	KJ168077	-		Bavarian State Collection of Zoology	*Hippotion celerio*		Papua New Guinea, Central
HCPN012-03	USNM ENT 00678971	600[0n]	KJ168535	-		Smithsonian Institution	*Hippotion celerio*		Papua New Guinea, Madang
LOSA200-08	05-SA-208	658[0n]	KJ168461	-		Biodiversity Institute of Ontario	*Hippotion celerio*		South Africa
LOSA203-08	05-SA-211	658[0n]	KJ168263	-		Biodiversity Institute of Ontario	*Hippotion celerio*		South Africa
GWOTG692-12	BC ZSM Lep 65818	621[0n]	KJ168092	-		Bavarian State Collection of Zoology	*Hippotion celerio*		South Africa, Gauteng
SSDA208-06	PD-BC 020	658[0n]	KJ168389	-		Research Collection of Philippe Darge	*Hippotion celerio*		Tanzania
SPHYE116-09	BC-EST0492	618[0n]	KJ168244	-		Research Collection of Yves Estradel	*Hippotion celerio*		Tanzania
SPHYE219-09	BC-EST0595	658[0n]	GU703983	-		Research Collection of Yves Estradel	*Hippotion celerio*		Tanzania,
SPPDA093-07	PD-BC 473	658[0n]	KJ168280	-		Research Collection of Philippe Darge	*Hippotion celerio*		Tanzania, Rukwa
SPHAP036-06	MA05-06-11-10	658[0n]	KJ168371	-		Research Collection of Morton Sam Adams	*Hippotion celerio*		Zambia, Copperbelt
SOWE241-07	BC-Hax4140	658[0n]	KJ168474	-		Research Collection of Jean Haxaire	*Hippotion rosetta*		Indonesia, Papua
SOWE240-07	BC-Hax4139	658[0n]	JN678035	-		Research Collection of Jean Haxaire	*Hippotion rosetta*		Indonesia, Papua
SOWD602-06	BC-Hax3501	607[0n]	KJ168413	-		Research Collection of Jean Haxaire	*Hippotion rosetta*		Malaysia, Sabah
PMANK044-06	USNM ENT 00196446	583[6n]	KJ168550	-		Smithsonian Institution	*Hippotion rosetta*		Myanmar, Sagaing
SOWD603-06	BC-Hax3502	607[0n]	KJ168466	-		Research Collection of Jean Haxaire	*Hippotion rosetta*		Philippines, Benguet
SPRBA168-08	BC-RBP-0168	600[1n]	KJ168238	-		Research Collection of Ron Brechlin	*Hippotion scrofa*		Fiji, Viti Levu Island
SPRBA167-08	BC-RBP-0167	635[0n]	KJ168154	-		Research Collection of Ron Brechlin	*Hippotion scrofa*		New Caledonia
SOWD679-06	BC-Hax3578	607[0n]	KJ168411	-		Research Collection of Jean Haxaire	*Hippotion scrofa*		New Caledonia
SPTMB119-10	BC-Mel1121	658[0n]	HQ580934	-		Research Collection of Tomas Melichar	*Hippotion velox*		India, Karnataka
SPTMA869-09	BC-Mel 0947	646[0n]	KJ168313	-		Research Collection of Tomas Melichar	*Hippotion velox*		Indonesia, Maluku
SPTMA870-09	BC-Mel 0948	645[0n]	KJ168110	-		Research Collection of Tomas Melichar	*Hippotion velox*		Indonesia, Maluku
SPTMA867-09	BC-Mel 0945	658[0n]	KJ168541	-		Research Collection of Tomas Melichar	*Hippotion velox*		Indonesia, Nusa Tenggara Timur
SPTMA871-09	BC-Mel 0949	556[1n]	KJ168292	-		Research Collection of Tomas Melichar	*Hippotion velox*		Indonesia, Nusa Tenggara Timur
SPTMA868-09	BC-Mel 0946	577[0n]	KJ168354	-		Research Collection of Tomas Melichar	*Hippotion velox*		Indonesia, Nusa Tenggara Timur
SPTMA872-09	BC-Mel 0950	615[0n]	KJ168297	-		Research Collection of Tomas Melichar	*Hippotion velox*		Indonesia, Nusa Tenggara Timur
SPTMA865-09	BC-Mel 0943	658[0n]	KJ168489	-		Research Collection of Tomas Melichar	*Hippotion velox*		Indonesia, Papua
SPTMA862-09	BC-Mel 0940	658[0n]	KJ168219	-		Research Collection of Tomas Melichar	*Hippotion velox*		New Caledonia
HCPN068-03	USNM ENT 00196075	630[0n]	KJ168444	-		Smithsonian Institution	*Hippotion velox*		Papua New Guinea, Gulf
HCPN067-03	USNM ENT 00196074	658[0n]	KJ168431	-		Smithsonian Institution	*Hippotion velox*		Papua New Guinea, Gulf
HCPN066-03	USNM ENT 00196051	658[0n]	KJ168526	-		Smithsonian Institution	*Hippotion velox*		Papua New Guinea, Gulf
SPTMA864-09	BC-Mel 0942	640[0n]	KJ168186	-		Research Collection of Tomas Melichar	*Hippotion velox*		Philippines, Leyte
SPTMA863-09	BC-Mel 0941	658[0n]	KJ168234	-		Research Collection of Tomas Melichar	*Hippotion velox*		Sri Lanka, Marawilla
SPTMA861-09	BC-Mel 0939	658[0n]	KJ168204	-		Research Collection of Tomas Melichar	*Hippotion velox*		Sri Lanka, Marawilla
GWOSV028-11	BC ZSM Lep 44254	658[0n]	KJ168381	-		Bavarian State Collection of Zoology	*Hippotion velox*		Taiwan, Hualien City
SOWE545-07	BC-Hax4444	658[0n]	HM384193	-		Research Collection of Jean Haxaire	*Hippotion velox*		Vanuatu

Process ID	Sample ID	COI-5P	GB Acc. COI	28S-D2	GB Acc. 28S	Institution Storing	Species	Types	Origin
SPTMA626-09	BC-Mel 0704	658[0n]	KJ168124	-		Research Collection of Tomas Melichar	*Macroglossum corythus*		China, Guangxi
SPTOL082-07	AYK-04-0380	658[0n]	KJ168480	-		University of Maryland	*Macroglossum corythus*		India, Assam
SPTMA622-09	BC-Mel 0700	658[0n]	KJ168177	-		Research Collection of Tomas Melichar	*Macroglossum corythus*		Indonesia, Nusa Tenggara Timur
SPTMA621-09	BC-Mel 0699	564[0n]	KJ168305	-		Research Collection of Tomas Melichar	*Macroglossum corythus*		Indonesia, Nusa Tenggara Timur
SPTMA629-09	BC-Mel 0707	578[0n]	KJ168268	-		Research Collection of Tomas Melichar	*Macroglossum corythus*		Indonesia, Papua
SPTMA793-09	BC-Mel 0871	621[0n]	KJ168309	-		Research Collection of Tomas Melichar	*Macroglossum corythus*		Indonesia, Sulawesi Selatan
SPTMA620-09	BC-Mel 0698	658[0n]	KJ168236	-		Research Collection of Tomas Melichar	*Macroglossum corythus*		Indonesia, Sulawesi Selatan
SPTMA623-09	BC-Mel 0701	549[0n]	KJ168090	-		Research Collection of Tomas Melichar	*Macroglossum corythus*		Indonesia, Sulawesi Selatan
SPTMA632-09	BC-Mel 0710	620[0n]	KJ168555	-		Research Collection of Tomas Melichar	*Macroglossum corythus*		Indonesia, Sulawesi Selatan
SOWF003-11	BC-Hax4861	658[0n]	JN280999	-		Research Collection of Jean Haxaire	*Macroglossum corythus*		Indonesia, Sulawesi Selatan
SOWF070-11	BC-Hax4928	658[0n]	JN281047	-		Research Collection of Jean Haxaire	*Macroglossum corythus*		Indonesia, Sulawesi Selatan
SOWF066-11	BC-Hax4924	658[0n]	JN281044	-		Research Collection of Jean Haxaire	*Macroglossum corythus*		Indonesia, Sulawesi Selatan
SOWF004-11	BC-Hax4862	658[0n]	JN281000	-		Research Collection of Jean Haxaire	*Macroglossum corythus*		Indonesia, Sulawesi Selatan
SOWD744-06	BC-Hax3643	658[0n]	JN678135	-		Research Collection of Jean Haxaire	*Macroglossum corythus*		Indonesia, Sulawesi Selatan
SOWF241-12	BC-Hax5099	658[0n]	KJ168174	-		Research Collection of Jean Haxaire	*Macroglossum corythus*		Laos, Houaphan
SOWF240-12	BC-Hax5098	658[0n]	KJ168205	-		Research Collection of Jean Haxaire	*Macroglossum corythus*		Laos, Houaphan
SOWF248-12	BC-Hax5106	658[0n]	KJ168097	-		Research Collection of Jean Haxaire	*Macroglossum corythus*		Laos, Houaphan
SOWF247-12	BC-Hax5105	658[0n]	KJ168134	-		Research Collection of Jean Haxaire	*Macroglossum corythus*		Laos, Houaphan
SPTMA624-09	BC-Mel 0702	656[2n]	KJ168102	-		Research Collection of Tomas Melichar	*Macroglossum corythus*		Malaysia, Pahang
SPTMA625-09	BC-Mel 0703	641[0n]	KJ168386	-		Research Collection of Tomas Melichar	*Macroglossum corythus*		Malaysia, Pahang
SOWD749-06	BC-Hax3648	606[1n]	KJ168096	-		Research Collection of Jean Haxaire	*Macroglossum corythus*		Malaysia, Pahang
HCPN016-03	USNM ENT 00676002	581[0n]	KJ168293	-		Smithsonian Institution	*Macroglossum corythus*		Papua New Guinea, Madang
HCPN015-03	USNM ENT 00676001	592[0n]	KJ168383	-		Smithsonian Institution	*Macroglossum corythus*		Papua New Guinea, Madang
HCPN014-03	USNM ENT 00676000	590[0n]	KJ168356	-		Smithsonian Institution	*Macroglossum corythus*		Papua New Guinea, Madang
SOWE114-07	BC-Hax4013	658[0n]	KJ168397	-		Research Collection of Jean Haxaire	*Macroglossum corythus*		Philippines, Leyte
SOWF033-11	BC-Hax4891	658[0n]	JN281020	-		Research Collection of Jean Haxaire	*Macroglossum corythus*		Philippines, Leyte
SOWF032-11	BC-Hax4890	658[0n]	JN281019	-		Research Collection of Jean Haxaire	*Macroglossum corythus*		Philippines, Leyte
SPTMB212-10	BC-Mel1214	657[1n]	HQ977191	-		Research Collection of Tomas Melichar	*Macroglossum corythus*		Solomon Islands, Santa Cruz
SPTMA619-09	BC-Mel 0697	658[0n]	KJ168458	-		Research Collection of Tomas Melichar	*Macroglossum corythus*		Solomon Islands, Santa Cruz
SPTMA618-09	BC-Mel 0696	658[0n]	KJ168220	-		Research Collection of Tomas Melichar	*Macroglossum corythus*		Solomon Islands, Santa Cruz
SOWD740-06	BC-Hax3639	607[0n]	KJ168117	-		Research Collection of Jean Haxaire	*Macroglossum corythus*		Solomon Islands, Santa Cruz
SOWD741-06	BC-Hax3640	607[0n]	KJ168193	-		Research Collection of Jean Haxaire	*Macroglossum corythus*		Solomon Islands, Santa Cruz
SOWD739-06	BC-Hax3638	607[0n]	KJ168188	-		Research Collection of Jean Haxaire	*Macroglossum corythus*		Solomon Islands, Santa Cruz
SPTMA743-09	BC-Mel 0821	658[0n]	KJ168147	-		Research Collection of Tomas Melichar	*Macroglossum corythus fulvicaudata*		Solomon Islands, Central Malaita
SPTMA792-09	BC-Mel 0870	658[0n]	KJ168376	-		Research Collection of Tomas Melichar	*Macroglossum corythus fuscicauda*		New Caledonia

Process ID	Sample ID	COI-5P	GB Acc. COI	28S-D2	GB Acc. 28S	Institution Storing	Species	Types	Origin
SPTMA754-09	BC-Mel 0832	658[0n]	KJ168519	-		Research Collection of Tomas Melichar	*Macroglossum corythus luteata*		Indonesia, Jawa Barat
SPTMA757-09	BC-Mel 0835	658[0n]	KJ168518	-		Research Collection of Tomas Melichar	*Macroglossum corythus luteata*		Laos
SPTMA758-09	BC-Mel 0836	658[0n]	KJ168281	-		Research Collection of Tomas Melichar	*Macroglossum corythus luteata*		Philippines, Leyte
SPTMA759-09	BC-Mel 0837	658[0n]	KJ168324	-		Research Collection of Tomas Melichar	*Macroglossum corythus luteata*		Taiwan, Nantou County
SPTMA765-09	BC-Mel 0843	658[0n]	KJ168417	-		Research Collection of Tomas Melichar	*Macroglossum corythus pylene*		Indonesia, Maluku
SPTMA764-09	BC-Mel 0842	658[0n]	KJ168269	-		Research Collection of Tomas Melichar	*Macroglossum corythus pylene*		Indonesia, Maluku
SPTMA762-09	BC-Mel 0840	658[0n]	KJ168306	-		Research Collection of Tomas Melichar	*Macroglossum corythus pylene*		Indonesia, Maluku
SPTMB057-09	BC-Mel 1069	658[0n]	GU704523	-		Research Collection of Tomas Melichar	*Macroglossum divergens*		India, Karnataka
SOWE140-07	BC-Hax4039	658[0n]	KJ168142	-		Research Collection of Jean Haxaire	*Macroglossum divergens heliophila*		Indonesia, Sulawesi Utara
SOWE139-07	BC-Hax4038	548[0n]	KJ168276	-		Research Collection of Jean Haxaire	*Macroglossum divergens heliophila*		Indonesia, Sulawesi Utara
SPTMA915-09	BC-Mel 0993	658[0n]	KJ168498	-		Research Collection of Tomas Melichar	*Macroglossum dohertyi*		Indonesia, Maluku
SPTMA916-09	BC-Mel 0994	589[1n]	KJ168370	-		Research Collection of Tomas Melichar	*Macroglossum dohertyi*		Indonesia, Maluku
SPTMA914-09	BC-Mel 0992	658[0n]	KJ168224	-		Research Collection of Tomas Melichar	*Macroglossum dohertyi doddi*		Indonesia, Papua
SPTMA913-09	BC-Mel 0991	639[0n]	KJ168483	-		Research Collection of Tomas Melichar	*Macroglossum dohertyi doddi*		Indonesia, Papua
SPTOL197-07	MF-05-0018	619[0n]	KJ168327	-		University of Maryland	*Macroglossum hirundo*		Fiji
SPTMA857-09	BC-Mel 0935	658[0n]	KJ168333	-		Research Collection of Tomas Melichar	*Macroglossum hirundo*		Tonga
LTOL260-07	MF-05-0011	658[0n]	KJ168481	-		University of Maryland	*Macroglossum hirundo errans*		Vanuatu
SPTOL199-07	MF-05-0028	646[0n]	KJ168323	-		University of Maryland	*Macroglossum hirundo errans*		Vanuatu
SPTMA578-09	BC-Mel 0656	635[0n]	KJ168069	-		Research Collection of Tomas Melichar	*Macroglossum hirundo lifuensis*		New Caledonia
SPTMA579-09	BC-Mel 0657	637[0n]	KJ168170	-		Research Collection of Tomas Melichar	*Macroglossum hirundo lifuensis*		New Caledonia
SOWE108-07	BC-Hax4007	271[0n]	KJ168180	-		Research Collection of Jean Haxaire	*Macroglossum nubilum*		Papua New Guinea
SPTMA613-09	BC-Mel 0691	641[0n]	KJ168355	-		Research Collection of Tomas Melichar	*Macroglossum prometheus*		Malaysia, Pahang
SOWE120-07	BC-Hax4019	658[0n]	KJ168249	-		Research Collection of Jean Haxaire	*Macroglossum prometheus*		Philippines, Leyte
HCPN023-03	USNM ENT 00676007	587[0n]	KJ168303	-		Smithsonian Institution	*Macroglossum prometheus lineata*		Papua New Guinea, Madang
HCPN021-03	USNM ENT 00676005	595[0n]	KJ168487	-		Smithsonian Institution	*Macroglossum prometheus lineata*		Papua New Guinea, Madang
HCPN024-03	USNM ENT 00676008	604[35n]	KJ168511	-		Smithsonian Institution	*Macroglossum prometheus lineata*		Papua New Guinea, Madang
HCPN022-03	USNM ENT 00676006	565[3n]	KJ168239	-		Smithsonian Institution	*Macroglossum prometheus lineata*		Papua New Guinea, Madang
SPTMA736-09	BC-Mel 0814	658[0n]	KJ168138	-		Research Collection of Tomas Melichar	*Macroglossum rectans*		Indonesia, Maluku
SPTMA577-09	BC-Mel 0655	587[1n]	KJ168283	-		Research Collection of Tomas Melichar	*Macroglossum rectans*		Indonesia, Maluku
SPTMA575-09	BC-Mel 0653	658[0n]	KJ168348	-		Research Collection of Tomas Melichar	*Macroglossum rectans*		Indonesia, Maluku
SPTMA576-09	BC-Mel 0654	639[1n]	KJ168125	-		Research Collection of Tomas Melichar	*Macroglossum rectans*		Indonesia, Maluku
SPTMA796-09	BC-Mel 0874	658[0n]	KJ168392	-		Research Collection of Tomas Melichar	*Macroglossum tenebrosa*		Indonesia, Papua
SOWD754-06	BC-Hax3653	636[0n]	KJ168449	588[0n]	KJ168590	Research Collection of Jean Haxaire	*Macroglossum tenebrosa*		Indonesia, Sulawesi Selatan
SOWD755-06	BC-Hax3654	622[0n]	KJ168347	-		Research Collection of Jean Haxaire	*Macroglossum tenebrosa*		Indonesia, Sulawesi Selatan

Process ID	Sample ID	COI-5P	GB Acc. COI	28S-D2	GB Acc. 28S	Institution Storing	Species	Types	Origin
SOWD753-06	BC-Hax3652	658[0n]	JN678170	597[0n]	KJ168576	Research Collection of Jean Haxaire	*Macroglossum tenebrosa*		Indonesia, Sulawesi Selatan
CATS287-10	USNM ENT 00704550	658[0n]	HM906354	-		Smithsonian Institution	*Macroglossum tenebrosa*		Papua New Guinea, Madang
CATS260-10	USNM ENT 00704463	658[0n]	HM906330	-		Smithsonian Institution	*Macroglossum tenebrosa*		Papua New Guinea, Madang
SPTMB189-10	BC-Mel1191	658[0n]	HQ580998	-		Research Collection of Tomas Melichar	*Macroglossum vacillans*		Indonesia, Bali
SPTMB190-10	BC-Mel1192	658[0n]	HQ580999	-		Research Collection of Tomas Melichar	*Macroglossum vacillans*		Indonesia, Bali
SPTMB191-10	BC-Mel1193	658[0n]	HQ581000	-		Research Collection of Tomas Melichar	*Macroglossum vacillans*		Indonesia, Bali
SPTMA582-09	BC-Mel 0660	658[0n]	KJ168235	-		Research Collection of Tomas Melichar	*Macroglossum vacillans*		Indonesia, Maluku
SPTMA583-09	BC-Mel 0661	658[0n]	KJ168190	-		Research Collection of Tomas Melichar	*Macroglossum vacillans*		Indonesia, Maluku
SPTMA580-09	BC-Mel 0658	637[0n]	KJ168073	-		Research Collection of Tomas Melichar	*Macroglossum vacillans*		Indonesia, Sulawesi Selatan
SPTMA584-09	BC-Mel 0662	640[0n]	KJ168103	-		Research Collection of Tomas Melichar	*Macroglossum vacillans*		Timor-Leste
SPTMA581-09	BC-Mel 0659	658[0n]	KJ168321	-		Research Collection of Tomas Melichar	*Macroglossum vacillans*		Timor-Leste
SPTMA585-09	BC-Mel 0663	658[0n]	KJ168508	-		Research Collection of Tomas Melichar	*Macroglossum vacillans*		Timor-Leste
SPTMB472-10	BC-Mel1474	573[1n]	KJ168548	-		Research Collection of Tomas Melichar	*Meganoton rubescens*		India, Meghalaya
SPTMB439-10	BC-Mel1441	658[0n]	HQ581142	-		Research Collection of Tomas Melichar	*Meganoton rubescens*		Myanmar, Kun Yuam
SPTMB441-10	BC-Mel1443	658[0n]	HQ581143	-		Research Collection of Tomas Melichar	*Meganoton rubescens*		Thailand
SOWD180-06	BC-Hax3079	658[0n]	KJ168137	-		Research Collection of Jean Haxaire	*Meganoton rubescens*		Thailand, Chiang Mai
SOWD181-06	BC-Hax3080	658[0n]	JN678263	-		Research Collection of Jean Haxaire	*Meganoton rubescens*		Thailand, Kanchanaburi
SPTMB471-10	BC-Mel1473	658[0n]	HQ581165	-		Research Collection of Tomas Melichar	*Meganoton rubescens*		Vietnam
SPTMB470-10	BC-Mel1472	658[0n]	HQ581164	-		Research Collection of Tomas Melichar	*Meganoton rubescens*		Vietnam
SPTMB442-10	BC-Mel1444	658[0n]	HQ581144	-		Research Collection of Tomas Melichar	*Meganoton rubescens amboinicus*		Indonesia, Maluku
SPTMB469-10	BC-Mel1471	658[0n]	HQ581163	-		Research Collection of Tomas Melichar	*Meganoton rubescens amboinicus*		Indonesia, Sulawesi Selatan
SOWD176-06	BC-Hax3075	658[0n]	KJ168194	-		Research Collection of Jean Haxaire	*Meganoton rubescens amboinicus*		Indonesia, Sulawesi Selatan
SPTMB462-10	BC-Mel1464	658[0n]	HQ581161	-		Research Collection of Tomas Melichar	*Meganoton rubescens severina*		Indonesia, Maluku
SPTMB463-10	BC-Mel1465	658[0n]	HQ581162	-		Research Collection of Tomas Melichar	*Meganoton rubescens severina*		Indonesia, Maluku
SPTMB475-10	BC-Mel1477	657[1n]	HQ581168	-		Research Collection of Tomas Melichar	*Meganoton rubescens thielei*		Indonesia, Jawa Timur
SPTMB473-10	BC-Mel1475	658[0n]	HQ581166	-		Research Collection of Tomas Melichar	*Meganoton rubescens thielei*		Indonesia, Kalimantan Selatan
SPTMB474-10	BC-Mel1476	658[0n]	HQ581167	-		Research Collection of Tomas Melichar	*Meganoton rubescens thielei*		Indonesia, Sumatera Barat
SPTMB466-10	BC-Mel1468	624[0n]	KJ168068	-		Research Collection of Tomas Melichar	*Meganoton rubescens thielei*		Malaysia, Pahang
SOWD175-06	BC-Hax3074	658[0n]	KJ168095	-		Research Collection of Jean Haxaire	*Meganoton rubescens thielei*		Malaysia, Sabah
SPTMB476-10	BC-Mel1478	658[0n]	HQ581169	-		Research Collection of Tomas Melichar	*Meganoton rubescens titan*		Indonesia, Maluku
SPTMB477-10	BC-Mel1479	658[0n]	HQ581170	-		Research Collection of Tomas Melichar	*Meganoton rubescens titan*		Indonesia, Maluku
SPTMB404-10	BC-Mel1406	658[0n]	HQ581110	-		Research Collection of Tomas Melichar	*Nephele hespera*		India, Karnataka
SPTMB403-10	BC-Mel1405	627[0n]	KJ168197	-		Research Collection of Tomas Melichar	*Nephele hespera*		India, Karnataka
SPTMB402-10	BC-Mel1404	658[0n]	HQ581109	-		Research Collection of Tomas Melichar	*Nephele hespera*		India, Karnataka
SPTMB405-10	BC-Mel1407	658[0n]	HQ581111	-		Research Collection of Tomas Melichar	*Nephele hespera*		India, Karnataka

Process ID	Sample ID	COI-5P	GB Acc. COI	28S-D2	GB Acc. 28S	Institution Storing	Species	Types	Origin
SPTMB035-09	BC-Mel 1047	658[0n]	GU704544	-		Research Collection of Tomas Melichar	*Nephele hespera*		India, Karnataka
SPHAP048-06	MA05-08-26-01	658[0n]	JN678296	-		Research Collection of Morton Sam Adams	*Nephele hespera*		India, Maharashtra
SPHAP049-06	MA05-08-26-02	658[0n]	KJ168311	-		Research Collection of Morton Sam Adams	*Nephele hespera*		India, Maharashtra
SPTMB784-11	BC-Mel1786	658[0n]	JN281200	-		Research Collection of Tomas Melichar	*Nephele hespera*		Iran
SPTMC278-12	BC-Mel2279	658[0n]	KJ168171	-		Research Collection of Tomas Melichar	*Nephele hespera*		Pakistan
SPTMC279-12	BC-Mel2280	509[0n]	KJ168561	-		Research Collection of Tomas Melichar	*Nephele hespera*		Pakistan, Azad Kashmir
SPUEB551-07	BC-EMEM1491	619[0n]	KJ168344	-		Entomologisches Museum Eitschberger	*Pseudoangonyx excellens*		Indonesia, Maluku
SARBB543-09	BC-RBP-2568	407[0n]	KJ168152	-		Research Collection of Ron Brechlin	*Pseudoangonyx excellens*		Indonesia, Papua
SPRBA282-08	BC-RBP-0282	658[0n]	KJ168494	-		Research Collection of Ron Brechlin	*Psilogramma menephron*		China, Hainan
SPRBA283-08	BC-RBP-0283	658[0n]	KJ168484	-		Research Collection of Ron Brechlin	*Psilogramma menephron*		China, Hainan
SPTMA322-07	BC-Mel 0400	647[0n]	KJ168528	-		Research Collection of Tomas Melichar	*Psilogramma menephron*		China, Yunnan
SPRBA287-08	BC-RBP-0287	634[0n]	KJ168375	-		Research Collection of Ron Brechlin	*Psilogramma menephron*		China, Yunnan
SPMNP346-07	BC-MNHNJP0236	658[0n]	KJ168329	-		Museum National d'Histoire Naturelle, Paris	*Psilogramma menephron*		China, Yunnan
SPRBA279-08	BC-RBP-0279	656[0n]	KJ168127	-		Research Collection of Ron Brechlin	*Psilogramma menephron*		India, Andaman and Nicobar
SPRBA277-08	BC-RBP-0277	658[0n]	KJ168098	-		Research Collection of Ron Brechlin	*Psilogramma menephron*		India, Assam
SPRBA278-08	BC-RBP-0278	658[0n]	KJ168492	-		Research Collection of Ron Brechlin	*Psilogramma menephron*		India, Assam
SPRBA276-08	BC-RBP-0276	609[0n]	KJ168414	-		Research Collection of Ron Brechlin	*Psilogramma menephron*		India, Sikkim
SPRBA305-08	BC-RBP-0305	634[0n]	KJ168536	-		Research Collection of Ron Brechlin	*Psilogramma menephron*		Indonesia, Aceh
SPTMA315-07	BC-Mel 0393	647[0n]	KJ168542	-		Research Collection of Tomas Melichar	*Psilogramma menephron*		Indonesia, Bali
SPTMC461-12	BC-Mel2462	658[0n]	KJ168252	-		Research Collection of Tomas Melichar	*Psilogramma menephron*		Indonesia, Bengkulu
SPRBA289-08	BC-RBP-0289	658[0n]	KJ168331	-		Research Collection of Ron Brechlin	*Psilogramma menephron*		Indonesia, Jawa Barat
SPRBA288-08	BC-RBP-0288	658[0n]	KJ168367	-		Research Collection of Ron Brechlin	*Psilogramma menephron*		Indonesia, Jawa Barat
SPTMC472-12	BC-Mel2473	658[0n]	KJ168272	-		Research Collection of Tomas Melichar	*Psilogramma menephron*		Indonesia, Jawa Barat
SPTMC471-12	BC-Mel2472	658[0n]	KJ168473	-		Research Collection of Tomas Melichar	*Psilogramma menephron*		Indonesia, Jawa Barat
SPRBA290-08	BC-RBP-0290	634[0n]	KJ168446	-		Research Collection of Ron Brechlin	*Psilogramma menephron*		Indonesia, Jawa Barat
SPTVA518-07	VAG-2329	658[0n]	KJ168456	-		Research Collection of T. Vaglia	*Psilogramma menephron*		Indonesia, Jawa Timur
SPRBA351-08	BC-RBP-0351	609[0n]	KJ168410	-		Research Collection of Ron Brechlin	*Psilogramma menephron*		Indonesia, Kalimantan Tengah
SPRBA353-08	BC-RBP-0353	658[0n]	KJ168206	-		Research Collection of Ron Brechlin	*Psilogramma menephron*		Indonesia, Kalimantan Tengah
SPRBA352-08	BC-RBP-0352	609[0n]	KJ168434	-		Research Collection of Ron Brechlin	*Psilogramma menephron*		Indonesia, Kalimantan Tengah
SPUEB597-09	BC-EMEM1537	658[0n]	GU704637	-		Entomologisches Museum Eitschberger	*Psilogramma menephron*		Indonesia, Maluku
SPUEB596-09	BC-EMEM1536	658[0n]	GU704638	-		Entomologisches Museum Eitschberger	*Psilogramma menephron*		Indonesia, Maluku
SPUEB587-09	BC-EMEM1527	658[0n]	GU704625	-		Entomologisches Museum Eitschberger	*Psilogramma menephron*		Indonesia, Maluku
SPUEB581-09	BC-EMEM1521	658[0n]	HM432647	-		Entomologisches Museum Eitschberger	*Psilogramma menephron*	Neotype	Indonesia, Maluku
SPTMC457-12	BC-Mel2458	573[0n]	KJ168559	-		Research Collection of Tomas Melichar	*Psilogramma menephron*		Indonesia, Maluku
SPTMA489-07	BC-Mel 0567	658[0n]	KJ168325	-		Research Collection of Tomas Melichar	*Psilogramma menephron*		Indonesia, Maluku
SPRBA323-08	BC-RBP-0323	658[0n]	KJ168288	-		Research Collection of Ron Brechlin	*Psilogramma menephron*		Indonesia, Maluku

Process ID	Sample ID	COI-5P	GB Acc. COI	28S-D2	GB Acc. 28S	Institution Storing	Species	Types	Origin
SPRBA324-08	BC-RBP-0324	636[0n]	KJ168074	-		Research Collection of Ron Brechlin	*Psilogramma menephron*		Indonesia, Maluku
SPTMA321-07	BC-Mel 0399	648[0n]	KJ168336	-		Research Collection of Tomas Melichar	*Psilogramma menephron*		Indonesia, Papua
SPRBA332-08	BC-RBP-0332	658[0n]	KJ168361	-		Research Collection of Ron Brechlin	*Psilogramma menephron*		Indonesia, Papua
SPRBA333-08	BC-RBP-0333	658[0n]	KJ168093	-		Research Collection of Ron Brechlin	*Psilogramma menephron*		Indonesia, Papua
SPTMA490-07	BC-Mel 0568	658[0n]	KJ168187	-		Research Collection of Tomas Melichar	*Psilogramma menephron*		Indonesia, Sulawesi Tengah
SPRBA322-08	BC-RBP-0322	658[0n]	KJ168221	-		Research Collection of Ron Brechlin	*Psilogramma menephron*		Indonesia, Sulawesi Tengah
SPRBA321-08	BC-RBP-0321	658[0n]	KJ168146	-		Research Collection of Ron Brechlin	*Psilogramma menephron*		Indonesia, Sulawesi Tengah
SPTMC484-12	BC-Mel2485	532[0n]	KJ168537	-		Research Collection of Tomas Melichar	*Psilogramma menephron*		Indonesia, Sumatera Barat
SPTMC458-12	BC-Mel2459	658[0n]	KJ168428	-		Research Collection of Tomas Melichar	*Psilogramma menephron*		Indonesia, Sumatera Barat
SPTMA324-07	BC-Mel 0402	658[0n]	KJ168546	-		Research Collection of Tomas Melichar	*Psilogramma menephron*		Indonesia, Sumatera Selatan
SPTMC474-12	BC-Mel2475	658[0n]	KJ168504	-		Research Collection of Tomas Melichar	*Psilogramma menephron*		Indonesia, Sumatera Utara
SPRBA350-08	BC-RBP-0350	634[0n]	KJ168301	-		Research Collection of Ron Brechlin	*Psilogramma menephron*		Malaysia, Pahang
SPRBA349-08	BC-RBP-0349	658[0n]	KJ168278	-		Research Collection of Ron Brechlin	*Psilogramma menephron*		Malaysia, Pahang
SPTOL142-07	AYK-04-0152	658[0n]	KJ168248	-		University of Maryland	*Psilogramma menephron*		Malaysia, Pahang
SPTOL151-07	A-0838	658[0n]	KJ168330	-		University of Maryland	*Psilogramma menephron*		Malaysia, Pahang
SPRBA358-08	BC-RBP-0358	636[0n]	KJ168358	-		Research Collection of Ron Brechlin	*Psilogramma menephron*		Myanmar, Tenasserim
SPRBA357-08	BC-RBP-0357	632[2n]	KJ168274	-		Research Collection of Ron Brechlin	*Psilogramma menephron*		Myanmar, Tenasserim
SPRBA293-08	BC-RBP-0293	658[0n]	KJ168316	-		Research Collection of Ron Brechlin	*Psilogramma menephron*		Myanmar, Tenasserim
SPRBA356-08	BC-RBP-0356	658[0n]	KJ168179	-		Research Collection of Ron Brechlin	*Psilogramma menephron*		Myanmar, Tenasserim
SARBA157-08	BC-RBP-0627	614[0n]	KJ168213	-		Research Collection of Ron Brechlin	*Psilogramma menephron*		Papua New Guinea, Ferguson island
SARBA156-08	BC-RBP-0626	614[0n]	KJ168521	-		Research Collection of Ron Brechlin	*Psilogramma menephron*		Papua New Guinea, Ferguson island
HCPN074-03	USNM ENT 00196077	639[0n]	KJ168396	-		Smithsonian Institution	*Psilogramma menephron*		Papua New Guinea, Gulf
HCPN070-03	USNM ENT 00196053	658[0n]	KJ168184	-		Smithsonian Institution	*Psilogramma menephron*		Papua New Guinea, Gulf
SML152-06	USNM ENT 00209808	567[0n]	KJ168262	-		Smithsonian Institution	*Psilogramma menephron*		Papua New Guinea, Madang
SML156-06	USNM ENT 00210163	658[0n]	KJ168295	-		Smithsonian Institution	*Psilogramma menephron*		Papua New Guinea, Madang
SML153-06	USNM ENT 00209792	658[0n]	KJ168290	-		Smithsonian Institution	*Psilogramma menephron*		Papua New Guinea, Madang
SML157-06	USNM ENT 00209799	658[0n]	KJ168374	-		Smithsonian Institution	*Psilogramma menephron*		Papua New Guinea, Madang
SML158-06	USNM ENT 00209796	658[0n]	KJ168554	-		Smithsonian Institution	*Psilogramma menephron*		Papua New Guinea, Madang
SML151-06	USNM ENT 00209811	567[0n]	KJ168486	-		Smithsonian Institution	*Psilogramma menephron*		Papua New Guinea, Madang
SML154-06	USNM ENT 00209798	658[0n]	KJ168172	-		Smithsonian Institution	*Psilogramma menephron*		Papua New Guinea, Madang
SML155-06	USNM ENT 00209794	658[0n]	KJ168443	-		Smithsonian Institution	*Psilogramma menephron*		Papua New Guinea, Madang
SML150-06	USNM ENT 00209797	658[0n]	KJ168118	-		Smithsonian Institution	*Psilogramma menephron*		Papua New Guinea, Madang
SPRBA334-08	BC-RBP-0334	658[0n]	KJ168462	-		Research Collection of Ron Brechlin	*Psilogramma menephron*		Papua New Guinea, Morobe
SPRBA336-08	BC-RBP-0336	609[0n]	KJ168228	-		Research Collection of Ron Brechlin	*Psilogramma menephron*		Papua New Guinea, Morobe
GWORB987-07	BC ZSM Lep 02209	567[1n]	KJ168400	-		Bavarian State Collection of Zoology	*Psilogramma menephron*		Papua New Guinea, Morobe

Process ID	Sample ID	COI-5P	GB Acc. COI	28S-D2	GB Acc. 28S	Institution Storing	Species	Types	Origin
SPTMC463-12	BC-Mel2464	658[0n]	KJ168287	-		Research Collection of Tomas Melichar	*Psilogramma menephron*		Philippines, Negros Occidental
SPTOL103-07	IJK-02-5988	658[0n]	KJ168362	-		University of Maryland	*Psilogramma menephron*		Philippines, Palawan
SPRBA300-08	BC-RBP-0300	587[0n]	KJ168104	-		Research Collection of Ron Brechlin	*Psilogramma menephron*		Philippines, Palawan
SPRBA301-08	BC-RBP-0301	658[0n]	KJ168509	-		Research Collection of Ron Brechlin	*Psilogramma menephron*		Philippines, Palawan
SOWD157-06	BC-Hax3056	658[0n]	KJ168257	-		Research Collection of Jean Haxaire	*Psilogramma menephron*		Philippines, Palawan
SPRBA367-08	BC-RBP-0367	609[0n]	KJ168087	-		Research Collection of Ron Brechlin	*Psilogramma menephron*		Taiwan, Yilan County
SPTVA521-07	VAG-2332	597[0n]	KJ168319	-		Research Collection of T. Vaglia	*Psilogramma menephron*		Thailand
SPRBA292-08	BC-RBP-0292	658[0n]	KJ168141	-		Research Collection of Ron Brechlin	*Psilogramma menephron*		Thailand, Chiang Mai
SPRBA291-08	BC-RBP-0291	570[1n]	KJ168100	-		Research Collection of Ron Brechlin	*Psilogramma menephron*		Thailand, Chiang Mai
SOWD156-06	BC-Hax3055	607[0n]	KJ168326	-		Research Collection of Jean Haxaire	*Psilogramma menephron*		Thailand, Kanchanaburi
SOWD155-06	BC-Hax3054	621[0n]	KJ168222	-		Research Collection of Jean Haxaire	*Psilogramma menephron*		Thailand, Kanchanaburi
SPTMC476-12	BC-Mel2477	658[0n]	KJ168454	-		Research Collection of Tomas Melichar	*Psilogramma menephron*		Vietnam
SPRBA359-08	BC-RBP-0359	658[0n]	KJ168261	-		Research Collection of Ron Brechlin	*Psilogramma menephron*		Vietnam
SPRBA363-08	BC-RBP-0363	605[0n]	KJ168275	-		Research Collection of Ron Brechlin	*Psilogramma menephron*		Vietnam
SPRBA360-08	BC-RBP-0360	658[0n]	KJ168433	-		Research Collection of Ron Brechlin	*Psilogramma menephron*		Vietnam
SPRBA286-08	BC-RBP-0286	658[0n]	KJ168143	-		Research Collection of Ron Brechlin	*Psilogramma menephron*		Vietnam
SPRBA285-08	BC-RBP-0285	634[0n]	KJ168429	-		Research Collection of Ron Brechlin	*Psilogramma menephron*		Vietnam
SPTVA541-07	VAG-2352	658[0n]	KJ168243	-		Research Collection of T. Vaglia	*Psilogramma papuensis*		Indonesia, Papua
SPTMA320-07	BC-Mel 0398	629[1n]	KJ168478	-		Research Collection of Tomas Melichar	*Psilogramma papuensis*		Indonesia, Papua
GWORB943-07	BC ZSM Lep 02165	503[0n]	KJ168164	-		Bavarian State Collection of Zoology	*Psilogramma papuensis*		Indonesia, Papua
SPRBA257-08	BC-RBP-0257	658[0n]	KJ168173	-		Research Collection of Ron Brechlin	*Psilogramma papuensis*	Paratype	Indonesia, Papua
SPRBA258-08	BC-RBP-0258	658[0n]	KJ168240	-		Research Collection of Ron Brechlin	*Psilogramma papuensis*		Indonesia, Papua
SARBA159-08	BC-RBP-0629	614[0n]	KJ168338	-		Research Collection of Ron Brechlin	*Psilogramma papuensis*		Papua New Guinea, Ferguson island
SARBA158-08	BC-RBP-0628	614[0n]	KJ168114	-		Research Collection of Ron Brechlin	*Psilogramma papuensis*		Papua New Guinea, Ferguson island
HCPN071-03	USNM ENT 00196054	571[3n]	KJ168232	-		Smithsonian Institution	*Psilogramma papuensis*		Papua New Guinea, Gulf
HCPN077-03	USNM ENT 00196080	633[6n]	KJ168132	-		Smithsonian Institution	*Psilogramma papuensis*		Papua New Guinea, Gulf
SPRBA254-08	BC-RBP-0254	638[1n]	KJ168453	-		Research Collection of Ron Brechlin	*Psilogramma papuensis*	Holotype	Papua New Guinea, Western Highland
SPRBA256-08	BC-RBP-0256	632[0n]	KJ168070	-		Research Collection of Ron Brechlin	*Psilogramma papuensis*	Paratype	Papua New Guinea, Western Highland
SPRBA255-08	BC-RBP-0255	658[0n]	KJ168401	-		Research Collection of Ron Brechlin	*Psilogramma papuensis*	Allotype	Papua New Guinea, Western Highland
SPTMB165-10	BC-Mel1167	639[0n]	KJ168120	-		Research Collection of Tomas Melichar	*Theretra celata*		Indonesia, Maluku
SPTMB164-10	BC-Mel1166	658[0n]	HQ580974	-		Research Collection of Tomas Melichar	*Theretra celata*		Indonesia, Maluku
SPTMB162-10	BC-Mel1164	658[0n]	HQ580973	-		Research Collection of Tomas Melichar	*Theretra celata*		Indonesia, Maluku
SPTMB161-10	BC-Mel1163	658[0n]	HQ580972	-		Research Collection of Tomas Melichar	*Theretra celata*		Indonesia, Maluku
SPTMB166-10	BC-Mel1168	658[0n]	HQ580975	-		Research Collection of Tomas Melichar	*Theretra celata*		Indonesia, Maluku

Process ID	Sample ID	COI-5P	GB Acc. COI	28S-D2	GB Acc. 28S	Institution Storing	Species	Types	Origin
SPTMB185-10	BC-Mel1187	658[0n]	HQ580994	-		Research Collection of Tomas Melichar	*Theretra celata*		Indonesia, Nusa Tenggara Timur
SPTMB160-10	BC-Mel1162	658[0n]	HQ580971	-		Research Collection of Tomas Melichar	*Theretra celata*		Indonesia, Papua
SOWC845-06	BC-Hax2744	557[0n]	KJ168332	-		Research Collection of Jean Haxaire	*Theretra celata*		Papua New Guinea, Morobe
SPTMB253-10	BC-Mel1255	658[0n]	HQ977228	-		Research Collection of Tomas Melichar	*Theretra indistincta manuselensis*		Indonesia, Maluku
SPTMB252-10	BC-Mel1254	658[0n]	HQ977227	-		Research Collection of Tomas Melichar	*Theretra indistincta manuselensis*		Indonesia, Maluku
SPTMB254-10	BC-Mel1256	658[0n]	HQ977229	-		Research Collection of Tomas Melichar	*Theretra indistincta manuselensis*		Indonesia, Maluku
SOWC848-06	BC-Hax2747	658[0n]	KJ168079	-		Research Collection of Jean Haxaire	*Theretra indistincta manuselensis*		Indonesia, Sulawesi Tengah
SOWC847-06	BC-Hax2746	658[0n]	KJ168488	-		Research Collection of Jean Haxaire	*Theretra indistincta manuselensis*		Indonesia, Sulawesi Tengah
SOWC849-06	BC-Hax2748	658[0n]	KJ168365	-		Research Collection of Jean Haxaire	*Theretra indistincta manuselensis*		Indonesia, Sulawesi Tengah
SPTMB250-10	BC-Mel1252	658[0n]	HQ977225	-		Research Collection of Tomas Melichar	*Theretra indistincta papuensis*		Indonesia, Papua
SPTMB249-10	BC-Mel1251	658[0n]	HQ581009	-		Research Collection of Tomas Melichar	*Theretra indistincta papuensis*		Indonesia, Papua
SPTMB251-10	BC-Mel1253	658[0n]	HQ977226	-		Research Collection of Tomas Melichar	*Theretra indistincta papuensis*		Indonesia, Papua
HCPN042-03	USNM ENT 00196042	658[0n]	JN678608	-		Smithsonian Institution	*Theretra indistincta papuensis*		Papua New Guinea, Gulf
HCPN044-03	USNM ENT 00196057	576[0n]	KJ168502	-		Smithsonian Institution	*Theretra indistincta papuensis*		Papua New Guinea, Gulf
HCPN045-03	USNM ENT 00196058	617[0n]	KJ168351	-		Smithsonian Institution	*Theretra indistincta papuensis*		Papua New Guinea, Gulf
SOWC852-06	BC-Hax2751	557[0n]	KJ168391	-		Research Collection of Jean Haxaire	*Theretra indistincta papuensis*		Papua New Guinea, Morobe
SOWC851-06	BC-Hax2750	557[0n]	KJ168246	-		Research Collection of Jean Haxaire	*Theretra indistincta papuensis*		Papua New Guinea, Morobe
SPTOL158-07	AYK-04-0378-1	658[0n]	KJ168322	-		University of Maryland	*Theretra latreillii*		Papua New Guinea, Chimbu
SPTOL157-07	AYK-04-0377	658[0n]	KJ168253	-		University of Maryland	*Theretra latreillii*		Papua New Guinea, Chimbu
SOWC862-06	BC-Hax2761	606[0n]	KJ168560	-		Research Collection of Jean Haxaire	*Theretra latreillii lucasii*		Indonesia, Nusa Tenggara Barat
SOWC864-06	BC-Hax2763	606[0n]	KJ168390	-		Research Collection of Jean Haxaire	*Theretra latreillii lucasii*		Indonesia, Nusa Tenggara Barat
SOWC863-06	BC-Hax2762	606[0n]	KJ168435	-		Research Collection of Jean Haxaire	*Theretra latreillii lucasii*		Indonesia, Nusa Tenggara Barat
SPTOL106-07	AYK-04-0111	658[0n]	KJ168163	-		University of Maryland	*Theretra latreillii lucasii*		Malaysia, Pahang
SOWC866-06	BC-Hax2765	495[2n]	KJ168442	-		Research Collection of Jean Haxaire	*Theretra latreillii lucasii*		Malaysia, Sabah
SOWC867-06	BC-Hax2766	557[0n]	KJ168136	-		Research Collection of Jean Haxaire	*Theretra latreillii lucasii*		Philippines, Mountain
SPTMC385-12	BC-Mel2386	658[0n]	KJ168302	-		Research Collection of Tomas Melichar	*Theretra latreillii prattorum*		Indonesia, Maluku
SPTMC383-12	BC-Mel2384	658[0n]	KJ168072	-		Research Collection of Tomas Melichar	*Theretra latreillii prattorum*		Indonesia, Maluku
SPTMC386-12	BC-Mel2387	658[0n]	KJ168255	-		Research Collection of Tomas Melichar	*Theretra latreillii prattorum*		Indonesia, Maluku
SPTMC384-12	BC-Mel2385	658[0n]	KJ168525	-		Research Collection of Tomas Melichar	*Theretra latreillii prattorum*		Indonesia, Maluku
SPTMB227-10	BC-Mel1229	658[0n]	HQ977203	-		Research Collection of Tomas Melichar	*Theretra latreillii prattorum*		Indonesia, Maluku
SPTMB228-10	BC-Mel1230	658[0n]	HQ977204	-		Research Collection of Tomas Melichar	*Theretra latreillii prattorum*		Indonesia, Maluku
SOWE579-07	BC-Hax4478	658[0n]	KJ168076	-		Research Collection of Jean Haxaire	*Theretra nessus*		China, Yunnan
SOWC836-06	BC-Hax2735	556[0n]	KJ168166	-		Research Collection of Jean Haxaire	*Theretra nessus*		China, Yunnan
SOWC835-06	BC-Hax2734	606[0n]	KJ168506	-		Research Collection of Jean Haxaire	*Theretra nessus*		China, Yunnan

Process ID	Sample ID	COI-5P	GB Acc. COI	28S-D2	GB Acc. 28S	Institution Storing	Species	Types	Origin
SOWE578-07	BC-Hax4477	658[0n]	JN678621	-		Research Collection of Jean Haxaire	*Theretra nessus*		China, Yunnan
SPTMB038-09	BC-Mel 1050	658[0n]	GU704539	-		Research Collection of Tomas Melichar	*Theretra nessus*		India, Karnataka
SPHAP055-06	MA05-08-31-03	658[0n]	KJ168203	-		Research Collection of Morton Sam Adams	*Theretra nessus*		India, Karnataka
GWORB947-07	BC ZSM Lep 02169	572[0n]	KJ168202	-		Bavarian State Collection of Zoology	*Theretra nessus*		Indonesia, Papua
SPTOL253-09	AYK-04-0163	658[0n]	KJ168467	-		University of Maryland	*Theretra nessus*		Malaysia, Pahang
HCPN032-03	USNM ENT 00196072	590[0n]	KJ168116	-		Smithsonian Institution	*Theretra nessus*		Papua New Guinea, Gulf
HCPN030-03	USNM ENT 00196038	582[1n]	KJ168404	-		Smithsonian Institution	*Theretra nessus*		Papua New Guinea, Gulf
HCPN033-03	USNM ENT 00196073	658[0n]	KJ168101	-		Smithsonian Institution	*Theretra nessus*		Papua New Guinea, Gulf
HCPN031-03	USNM ENT 00196071	531[0n]	KJ168250	-		Smithsonian Institution	*Theretra nessus*		Papua New Guinea, Gulf
SPTMC380-12	BC-Mel2381	658[0n]	KJ168106	-		Research Collection of Tomas Melichar	*Theretra nessus*		Solomon Islands, Malaita
SOWC837-06	BC-Hax2736	658[0n]	KJ168282	-		Research Collection of Jean Haxaire	*Theretra nessus*		Timor-Leste, Lautem
SATWB107-08	BC-Roug1155	658[0n]	KJ168084	-		Research Collection of Jean Haxaire	*Theretra nessus*		Vietnam
SPTMC377-12	BC-Mel2378	658[0n]	KJ168094	-		Research Collection of Tomas Melichar	*Theretra nessus albata*		New Caledonia, Loyalty Islands
SPTMC378-12	BC-Mel2379	658[0n]	KJ168159	-		Research Collection of Tomas Melichar	*Theretra nessus albata*		New Caledonia, Loyalty Islands
SPTMC379-12	BC-Mel2380	630[0n]	KJ168524	-		Research Collection of Tomas Melichar	*Theretra nessus albata*		Vanuatu
SOWE546-07	BC-Hax4445	658[0n]	HM384194	-		Research Collection of Jean Haxaire	*Theretra nessus albata*		Vanuatu
SOWD620-06	BC-Hax3519	658[0n]	KJ168395	-		Research Collection of Jean Haxaire	*Theretra oldenlandiae*		China, Sichuan
SOWD619-06	BC-Hax3518	658[0n]	KJ168314	-		Research Collection of Jean Haxaire	*Theretra oldenlandiae*		China, Sichuan
SOWD621-06	BC-Hax3520	658[0n]	JN678622	-		Research Collection of Jean Haxaire	*Theretra oldenlandiae*		China, Sichuan
SPHAP068-06	MA06-01-05-36	658[0n]	KJ168195	-		Research Collection of Morton Sam Adams	*Theretra oldenlandiae*		India, Maharashtra
SPTOL250-09	AYK-04-0112	658[0n]	KJ168299	-		University of Maryland	*Theretra oldenlandiae*		Malaysia, Pahang
SPMNP389-07	BC-MNHNJP0279	609[0n]	KJ168353	-		Museum National d'Histoire Naturelle, Paris	*Theretra oldenlandiae*		Pakistan
SOWD623-06	BC-Hax3522	266[0n]	KJ168200	-		Research Collection of Jean Haxaire	*Theretra oldenlandiae fuscata*		Philippines, Mountain
SOWD622-06	BC-Hax3521	286[0n]	KJ168520	-		Research Collection of Jean Haxaire	*Theretra oldenlandiae fuscata*		Philippines, Mountain
SPTMB536-11	BC-Mel1538	658[0n]	JN281101	-		Research Collection of Tomas Melichar	*Theretra oldenlandiae lewini*		Indonesia, Sulawesi Utara
SPTMB513-11	BC-Mel1515	658[0n]	JN281085	-		Research Collection of Tomas Melichar	*Theretra radiosa*		Indonesia, Papua
SPTMB512-11	BC-Mel1514	658[0n]	KJ168500	-		Research Collection of Tomas Melichar	*Theretra radiosa*		Indonesia, Papua
HCPN040-03	USNM ENT 00196060	617[0n]	KJ168318	-		Smithsonian Institution	*Theretra radiosa*		Papua New Guinea, Gulf
SOWC807-06	BC-Hax2706	606[0n]	JN678628	255[0n]	KJ168594	Research Collection of Jean Haxaire	*Theretra radiosa*		Papua New Guinea, Morobe
SPHAP059-06	MA05-08-31-12	658[0n]	KJ168405	-		Research Collection of Morton Sam Adams	*Theretra silhetensis*		India, Karnataka
SOWD625-06	BC-Hax3524	293[0n]	KJ168558	-		Research Collection of Jean Haxaire	*Theretra silhetensis*		Malaysia, Sabah
SOWD624-06	BC-Hax3523	293[0n]	KJ168477	-		Research Collection of Jean Haxaire	*Theretra silhetensis*		Malaysia, Sabah
SOWD631-06	BC-Hax3530	645[0n]	KJ168196	-		Research Collection of Jean Haxaire	*Theretra silhetensis intersecta*		Indonesia, Sulawesi Tengah
SPTOL255-09	IJK-02-5987	658[0n]	KJ168289	-		University of Maryland	*Theretra silhetensis intersecta*		Philippines, Palawan
SOWE543-07	BC-Hax4442	658[0n]	HM384191	-		Research Collection of Jean Haxaire	*Theretra silhetensis intersecta*		Vanuatu
SOWE544-07	BC-Hax4443	658[0n]	HM384192	-		Research Collection of Jean Haxaire	*Theretra silhetensis intersecta*		Vanuatu

Process ID	Sample ID	COI-5P	GB Acc. COI	28S-D2	GB Acc. 28S	Institution Storing	Species	Types	Origin
SPTMB224-10	BC-Mel1226	407[0n]	KJ168557	-		Research Collection of Tomas Melichar	*Theretra tryoni*		Indonesia, Maluku
SPTMB225-10	BC-Mel1227	658[0n]	HQ977201	-		Research Collection of Tomas Melichar	*Theretra tryoni*		Indonesia, Maluku
SPTMB226-10	BC-Mel1228	658[0n]	HQ977202	-		Research Collection of Tomas Melichar	*Theretra tryoni*		Indonesia, Maluku
SOWC869-06	BC-Hax2768	605[1n]	KJ168436	-		Research Collection of Jean Haxaire	*Theretra tryoni*		Indonesia, Maluku
GWORB944-07	BC ZSM Lep 02166	604[1n]	KJ168345	-		Bavarian State Collection of Zoology	*Theretra tryoni*		Indonesia, Papua
