# Supplementary material for: Australian Sphingidae – DNA Barcodes Challenge Current Species Boundaries and Distributions
Source: PLoS One. 2014 Jul 2;9(7):e101108. doi: 10.1371/journal.pone.0101108 (PMC4079597; doi:10.1371/journal.pone.0101108)
Supplement: Table S3 — List of heterospecific records from outside Australia. (PDF) [file pone.0101108.s010.pdf]

Table S3: List of heterospecific records from outside Australia. GB Acc. = GenBank accession numbers.

Process ID	Sample ID	COI-5P	GB Acc. COI	28S-D2	GB Acc. 28S	Institution Storing	Species	Types	Origin
SPUEA052-07	BC-EMEM0052	658[0n]	KJ168562	-		Entomologisches Museum Eitschberger	*Acosmeryx miskinoides*		Indonesia, Maluku
SPUEA053-07	BC-EMEM0053	621[0n]	KJ168420	-		Entomologisches Museum Eitschberger	*Acosmeryx miskinoides*		Indonesia, Maluku
GWORB946-07	BC ZSM Lep 02168	574[0n]	KJ168223	-		Bavarian State Collection of Zoology	*Acosmeryx miskinoides*		Indonesia, Papua
SPUEA047-07	BC-EMEM0047	631[0n]	KJ168406	-		Entomologisches Museum Eitschberger	*Acosmeryx miskinoides*		Indonesia, Papua
SPUEA050-07	BC-EMEM0050	658[0n]	KJ168149	-		Entomologisches Museum Eitschberger	*Acosmeryx miskinoides*		Indonesia, Papua
SPUEA049-07	BC-EMEM0049	658[0n]	KJ168567	-		Entomologisches Museum Eitschberger	*Acosmeryx miskinoides*		Indonesia, Papua
SPUEA048-07	BC-EMEM0048	658[0n]	KJ168415	-		Entomologisches Museum Eitschberger	*Acosmeryx miskinoides*		Indonesia, Papua
SPUEA051-07	BC-EMEM0051	658[0n]	KJ168167	-		Entomologisches Museum Eitschberger	*Acosmeryx miskinoides*		Indonesia, Papua
SPTVA214-07	VAG-2025	658[0n]	KJ168144	-		Research Collection of T. Vaglia	*Acosmeryx miskinoides*	Paratype	Papua New Guinea
HCPN062-03	USNM ENT 00196050	657[0n]	KJ168507	-		Smithsonian Institution	*Acosmeryx miskinoides*		Papua New Guinea, Gulf
SML179-06	USNM ENT 00196623	657[0n]	KJ168409	-		Smithsonian Institution	*Acosmeryx miskinoides*		Papua New Guinea, Gulf
SML178-06	USNM ENT 00196622	656[0n]	KJ168182	-		Smithsonian Institution	*Acosmeryx miskinoides*		Papua New Guinea, Gulf
SML174-06	USNM ENT 00196618	657[0n]	KJ168455	-		Smithsonian Institution	*Acosmeryx miskinoides*		Papua New Guinea, Gulf
SML177-06	USNM ENT 00196621	656[0n]	KJ168317	-		Smithsonian Institution	*Acosmeryx miskinoides*		Papua New Guinea, Gulf
HCPN064-03	USNM ENT 00196084	600[0n]	KJ168140	-		Smithsonian Institution	*Acosmeryx miskinoides*		Papua New Guinea, Gulf
SML176-06	USNM ENT 00196620	657[0n]	KJ168082	-		Smithsonian Institution	*Acosmeryx miskinoides*		Papua New Guinea, Gulf
HCPN063-03	USNM ENT 00196083	617[0n]	KJ168360	-		Smithsonian Institution	*Acosmeryx miskinoides*		Papua New Guinea, Gulf
HCPN065-03	USNM ENT 00196085	590[0n]	KJ168112	-		Smithsonian Institution	*Acosmeryx miskinoides*		Papua New Guinea, Gulf
SML175-06	USNM ENT 00196619	657[0n]	KJ168460	-		Smithsonian Institution	*Acosmeryx miskinoides*		Papua New Guinea, Gulf
SML180-06	USNM ENT 00196624	657[0n]	KJ168192	-		Smithsonian Institution	*Acosmeryx miskinoides*		Papua New Guinea, Morobe
SML181-06	USNM ENT 00196625	656[0n]	KJ168448	-		Smithsonian Institution	*Acosmeryx miskinoides*		Papua New Guinea, Morobe
SML182-06	USNM ENT 00196626	657[0n]	KJ168566	-		Smithsonian Institution	*Acosmeryx miskinoides*		Papua New Guinea, Morobe
SPTMB314-10	BC-Mel1316	658[0n]	HQ581029	-		Research Collection of Tomas Melichar	*Ambulyx ceramensis*		Indonesia, Maluku
SPTMB312-10	BC-Mel1314	658[0n]	HQ581027	-		Research Collection of Tomas Melichar	*Ambulyx ceramensis*		Indonesia, Maluku
SPUEA577-07	BC-EMEM0577	658[0n]	KJ168447	-		Entomologisches Museum Eitschberger	*Ambulyx ceramensis*		Indonesia, Maluku
SPTVA281-07	VAG-2092	658[0n]	JN677699	-		Research Collection of T. Vaglia	*Ambulyx ceramensis*		Indonesia, Maluku
SPRBA757-09	BC-RBP-1805	658[0n]	GU704160	-		Research Collection of Ron Brechlin	*Ambulyx ceramensis*		Indonesia, Maluku
SPUEA633-07	BC-EMEM0633	658[0n]	KJ168472	-		Entomologisches Museum Eitschberger	*Ambulyx ceramensis*		Indonesia, Maluku
SPTMB313-10	BC-Mel1315	658[0n]	HQ581028	-		Research Collection of Tomas Melichar	*Ambulyx ceramensis*		Indonesia, Maluku
SPTMB315-10	BC-Mel1317	658[0n]	HQ581030	-		Research Collection of Tomas Melichar	*Ambulyx ceramensis*		Indonesia, Maluku
SPUEA630-07	BC-EMEM0630	657[0n]	KJ168432	-		Entomologisches Museum Eitschberger	*Ambulyx ceramensis*		Indonesia, Maluku
SPRBA758-09	BC-RBP-1806	487[0n]	KJ168503	-		Research Collection of Ron Brechlin	*Ambulyx ceramensis*		Indonesia, Maluku
SPTMB316-10	BC-Mel1318	658[0n]	HQ581031	-		Research Collection of Tomas Melichar	*Ambulyx ceramensis*		Indonesia, Maluku
SPUEA635-07	BC-EMEM0635	658[0n]	KJ168538	-		Entomologisches Museum Eitschberger	*Ambulyx ceramensis*		Indonesia, Maluku

SPRBA760-09	BC-RBP-1808	658[0n]	GU704167	-	Research Collection of Ron Brechlin	*Ambulyx rudloffi*	Paratype	Papua New Guinea, West New Britain
SPRBA759-09	BC-RBP-1807	658[0n]	GU704168	-	Research Collection of Ron Brechlin	*Ambulyx rudloffi*	Holotype	Papua New Guinea, West New Britain
SARBB532-09	BC-RBP-2557	658[0n]	GU704296	-	Research Collection of Ron Brechlin	*Angonyx meeki*		Solomon Islands, Guadalcanal
SPTMA041-07	BC-Mel 0119b	635[0n]	JN677749	-	Research Collection of Tomas Melichar	*Angonyx meeki*		Solomon Islands, Guadalcanal
SARBB1428-10	BC-RBP-2558.1	658[0n]	HQ973199	-	Research Collection of Ron Brechlin	*Angonyx meeki*		Solomon Islands, Guadalcanal
SARBB1442-10	BC-RBP-2561.1	658[0n]	HQ973210	-	Research Collection of Ron Brechlin	*Angonyx meeki*		Solomon Islands, Makira
SARBB1430-10	BC-RBP-2562.1	658[0n]	HQ973201	-	Research Collection of Ron Brechlin	*Angonyx meeki*		Solomon Islands, Rennell Island
SARBB1431-10	BC-RBP-2563.1	658[0n]	HQ973202	-	Research Collection of Ron Brechlin	*Angonyx meeki*		Solomon Islands, Rennell Island
SARBB535-09	BC-RBP-2560	658[0n]	GU704299	-	Research Collection of Ron Brechlin	*Angonyx meeki*		Solomon Islands, San Cristobal
SARBB1429-10	BC-RBP-2559.1	658[0n]	HQ973200	-	Research Collection of Ron Brechlin	*Angonyx meeki*		Solomon Islands, Santa Isabel
SARBB545-09	BC-RBP-2570	658[0n]	GU704305	-	Research Collection of Ron Brechlin	*Angonyx testacea*		Bhutan
SARBB1029-09	BC-RBP 3054	658[0n]	GU704114	-	Research Collection of Ron Brechlin	*Angonyx testacea*		China, Hainan
SARBB1028-09	BC-RBP 3053	658[0n]	GU704113	-	Research Collection of Ron Brechlin	*Angonyx testacea*		China, Yunnan
SARBB547-09	BC-RBP-2572	574[0n]	GU704303	-	Research Collection of Ron Brechlin	*Angonyx testacea*		China, Yunnan
SARBB1446-10	BC-RBP-2572.1	658[0n]	HQ973212	-	Research Collection of Ron Brechlin	*Angonyx testacea*		China, Yunnan
SARBB1022-09	BC-RBP 3047	658[0n]	GU704135	-	Research Collection of Ron Brechlin	*Angonyx testacea*		India, Andaman and Nicobar
SARBB1026-09	BC-RBP 3051	658[0n]	GU704115	-	Research Collection of Ron Brechlin	*Angonyx testacea*		India, Assam
SARBB1025-09	BC-RBP 3050	658[0n]	GU704117	-	Research Collection of Ron Brechlin	*Angonyx testacea*		India, Assam
SARBB1439-10	BC-RBP-2574.1	658[0n]	HQ973207	-	Research Collection of Ron Brechlin	*Angonyx testacea*		Indonesia, Maluku
SPTMC055-11	BC-Mel2056	658[0n]	JN281289	-	Research Collection of Tomas Melichar	*Angonyx testacea*		Indonesia, Maluku
SARBB1020-09	BC-RBP 3045	658[0n]	GU704136	-	Research Collection of Ron Brechlin	*Angonyx testacea*		Indonesia, Maluku
SARBB1021-09	BC-RBP 3046	658[0n]	GU704134	-	Research Collection of Ron Brechlin	*Angonyx testacea*		Indonesia, Nusa Tenggara Timur
SPTMB620-11	BC-Mel1622	658[0n]	JN281144	-	Research Collection of Tomas Melichar	*Angonyx testacea*		Indonesia, Papua
SARBB1044-09	BC-RBP 3069	658[0n]	GU704101	-	Research Collection of Ron Brechlin	*Angonyx testacea*		Indonesia, Papua
SARBB1043-09	BC-RBP 3068	658[0n]	GU704103	-	Research Collection of Ron Brechlin	*Angonyx testacea*		Indonesia, Papua
SOWE189-07	BC-Hax4088	658[0n]	GU703812	-	Research Collection of Jean Haxaire	*Angonyx testacea*		Indonesia, Sabah
SARBB1039-09	BC-RBP 3064	658[0n]	GU704143	-	Research Collection of Ron Brechlin	*Angonyx testacea*		Indonesia, Sulawesi Selatan
SARBB1038-09	BC-RBP 3063	658[0n]	GU704107	-	Research Collection of Ron Brechlin	*Angonyx testacea*		Indonesia, Sulawesi Selatan
SOWE186-07	BC-Hax4085	658[0n]	JN677751	-	Research Collection of Jean Haxaire	*Angonyx testacea*		Indonesia, Sulawesi Utara
SARBB546-09	BC-RBP-2571	658[0n]	GU704304	-	Research Collection of Ron Brechlin	*Angonyx testacea*		Laos
SOWE188-07	BC-Hax4087	630[0n]	KJ168553	-	Research Collection of Jean Haxaire	*Angonyx testacea*		Malaysia, Sabah
SARBB1017-09	BC-RBP 3042	658[0n]	GU704468	-	Research Collection of Ron Brechlin	*Angonyx testacea*		Philippines, Leyte
SARBB1035-09	BC-RBP 3060	658[0n]	GU704470	-	Research Collection of Ron Brechlin	*Angonyx testacea*		Philippines, Leyte
SARBB1036-09	BC-RBP 3061	658[0n]	GU704109	-	Research Collection of Ron Brechlin	*Angonyx testacea*		Philippines, Leyte
SARBB1037-09	BC-RBP 3062	658[0n]	GU704106	-	Research Collection of Ron Brechlin	*Angonyx testacea*		Philippines, Negros Occidental
SARBB1034-09	BC-RBP 3059	658[0n]	GU704108	-	Research Collection of Ron Brechlin	*Angonyx testacea*		Philippines, Negros Occidental
SARBB1031-09	BC-RBP 3056	658[0n]	GU704112	-	Research Collection of Ron Brechlin	*Angonyx testacea*		Philippines, Palawan

SARBB1030-09	BC-RBP 3055	658[0n]	GU704111	-		Research Collection of Ron Brechlin	*Angonyx testacea*		Philippines, Palawan
SARBB1032-09	BC-RBP 3057	658[0n]	GU704110	-		Research Collection of Ron Brechlin	*Angonyx testacea*		Philippines, Palawan
SPTOL048-07	AYK-04-0243	658[0n]	KJ168394	-		University of Maryland	*Angonyx testacea*		Taiwan, Pingtung County
LTOL334-07	AYK-04-0304	658[0n]	KJ168476	-		University of Maryland	*Angonyx testacea*		Taiwan, Pingtung County
SOWE190-07	BC-Hax4089	658[0n]	GU703814	-		Research Collection of Jean Haxaire	*Angonyx testacea*		Vietnam
SARBB1027-09	BC-RBP 3052	658[0n]	GU704116	-		Research Collection of Ron Brechlin	*Angonyx testacea*		Vietnam, South Vietnam
SOWC442-06	BC-Hax2341	608[0n]	JN677810	-		Research Collection of Jean Haxaire	*Cephonodes xanthus*		Japan
SPTMB627-11	BC-Mel1629	657[1n]	JN281145	-		Research Collection of Tomas Melichar	*Cephonodes xanthus*		Japan, Okinawa
SOWD382-06	BC-Hax3281	625[0n]	JN677824	-		Research Collection of Jean Haxaire	*Cizara sculpta*		Laos
SOWD383-06	BC-Hax3282	658[0n]	HM384123	-		Research Collection of Jean Haxaire	*Cizara sculpta*		Laos
SOWD381-06	BC-Hax3280	625[0n]	KJ168139	-		Research Collection of Jean Haxaire	*Cizara sculpta*		Laos
SOWF176-12	BC-Hax5034	658[0n]	KJ168130	-		Research Collection of Jean Haxaire	*Coenotes jakli*	Paratype	Indonesia, Maluku
SOWF175-12	BC-Hax5033	658[0n]	KJ168207	-		Research Collection of Jean Haxaire	*Coenotes jakli*	Paratype	Indonesia, Maluku
SOWB401-06	BC-Hax1394	634[0n]	KJ168099	-		Research Collection of Jean Haxaire	*Gnathothlibus saccoi*		Vanuatu
SOWB400-06	BC-Hax1393	626[0n]	KJ168479	-		Research Collection of Jean Haxaire	*Gnathothlibus saccoi*		Vanuatu
SOWB402-06	BC-Hax1395	623[0n]	KJ168372	-		Research Collection of Jean Haxaire	*Gnathothlibus vanuatuensis*		Vanuatu
SPTMB116-10	BC-Mel1118	658[0n]	HQ580931	-		Research Collection of Tomas Melichar	*Hippotion boerhaviae*		India, Karnataka
SPTMB058-09	BC-Mel 1070	658[0n]	GU704520	-		Research Collection of Tomas Melichar	*Hippotion boerhaviae*		India, Karnataka
SPTMB665-11	BC-Mel1667	658[0n]	JN281154	-		Research Collection of Tomas Melichar	*Hippotion boerhaviae*		India, Karnataka
SPTMB060-09	BC-Mel 1072	658[0n]	GU704518	-		Research Collection of Tomas Melichar	*Hippotion boerhaviae*		India, Karnataka
SPTMB042-09	BC-Mel 1054	658[0n]	GU704535	-		Research Collection of Tomas Melichar	*Hippotion boerhaviae*		India, Karnataka
SPTMB115-10	BC-Mel1117	658[0n]	HQ580930	-		Research Collection of Tomas Melichar	*Hippotion boerhaviae*		India, Karnataka
SPTMB114-10	BC-Mel1116	658[0n]	HQ580929	-		Research Collection of Tomas Melichar	*Hippotion boerhaviae*		India, Karnataka
SPHAP045-06	MA05-08-23-74	603[0n]	KJ168258	-		Research Collection of Morton Sam Adams	*Hippotion boerhaviae*		India, Maharashtra
SPTMB661-11	BC-Mel1663	658[0n]	JN281152	-		Research Collection of Tomas Melichar	*Hippotion boerhaviae*		Indonesia, Bali
SPTMB660-11	BC-Mel1662	658[0n]	JN281151	-		Research Collection of Tomas Melichar	*Hippotion boerhaviae*		Indonesia, Bali
SPTMB662-11	BC-Mel1664	658[0n]	JN281153	-		Research Collection of Tomas Melichar	*Hippotion boerhaviae*		Indonesia, Bali
SOWF232-12	BC-Hax5090	658[0n]	KJ168533	-		Research Collection of Jean Haxaire	*Hippotion boerhaviae*		Laos, Khammouan
SOWF109-12	BC-Hax4967	658[0n]	KJ168544	-		Research Collection of Jean Haxaire	*Hippotion boerhaviae*		New Caledonia, South
SOWF110-12	BC-Hax4968	658[0n]	KJ168298	-		Research Collection of Jean Haxaire	*Hippotion boerhaviae*		New Caledonia, South
SPTMB666-11	BC-Mel1668	658[0n]	JN281155	-		Research Collection of Tomas Melichar	*Hippotion boerhaviae*		Philippines, Mindoro
SPTMB667-11	BC-Mel1669	658[0n]	JN281156	-		Research Collection of Tomas Melichar	*Hippotion boerhaviae*		Philippines, Negros
SOWE238-07	BC-Hax4137	610[0n]	KJ168439	557[1n]	KJ168588	Research Collection of Jean Haxaire	*Hippotion joiceyi*		Indonesia, Papua
SOWE239-07	BC-Hax4138	639[0n]	JN678025	571[0n]	KJ168579	Research Collection of Jean Haxaire	*Hippotion joiceyi*		Indonesia, Papua
SPTMA198-07	BC-Mel 0276	658[0n]	KJ168349	599[0n]	KJ168583	Research Collection of Tomas Melichar	*Hippotion joiceyi*		Indonesia, Papua
HCPN025-03	84093	596[5n]	KJ168377	-		Smithsonian Institution	*Macroglossum troglodytus papuanum*		Papua New Guinea, Madang
HCPN027-03	USNM ENT 00676004	596[0n]	KJ168440	-		Smithsonian Institution	*Macroglossum troglodytus papuanum*		Papua New Guinea, Madang

HCPN028-03	83080	569[3n]	KJ168359	-		Smithsonian Institution	*Macroglossum troglodytus papuanum*		Papua New Guinea, Madang
SPUEB594-09	BC-EMEM1534	649[0n]	GU704632	-		Entomologisches Museum Eitschberger	*Psilogramma anne*	Holotype	Indonesia, Papua
SPZSM066-08	BC-ZSMRR0066	566[92n]	KJ168412	-		Bavarian State Collection of Zoology	*Psilogramma bartschereri*	Holotype	Sri Lanka
SPUEB605-09	BC-EMEM1545	658[0n]	GU704640	-		Entomologisches Museum Eitschberger	*Psilogramma choui*	Holotype	China, Zhejiang
SPZSM067-08	BC-ZSMRR0067	658[0n]	KJ168551	-		Bavarian State Collection of Zoology	*Psilogramma danneri*	Holotype	India
SPZSM061-08	BC-ZSMRR0061	457[192n]	KJ168529	-		Bavarian State Collection of Zoology	*Psilogramma gerstmeieri*	Holotype	China
SPUEB607-09	BC-EMEM1547	658[0n]	GU704643	-		Entomologisches Museum Eitschberger	*Psilogramma hainanensis*	Holotype	China, Hainan
SPUEB609-09	BC-EMEM1549	658[0n]	GU704645	-		Entomologisches Museum Eitschberger	*Psilogramma hauensteini*	Allotype	China, Guangxi
SPUEB602-09	BC-EMEM1542	658[0n]	GU704642	-		Entomologisches Museum Eitschberger	*Psilogramma mastrigti*	Holotype	Indonesia, Papua
SPUEB580-09	BC-EMEM1520	658[0n]	GU704620	-		Entomologisches Museum Eitschberger	*Psilogramma stameri*	Holotype	Indonesia, Sumatera Utara
SPUEB570-09	BC-EMEM1510	658[0n]	GU704612	-		Entomologisches Museum Eitschberger	*Psilogramma surholti*	Allotype	Vietnam
SPUEB569-09	BC-EMEM1509	658[0n]	GU704613	-		Entomologisches Museum Eitschberger	*Psilogramma surholti*	Holotype	Vietnam
SPTMC295-12	BC-Mel2296	658[0n]	KJ168465	-		Research Collection of Tomas Melichar	*Rethera komarovi*		Armenia
SPTMC296-12	BC-Mel2297	658[0n]	KJ168552	-		Research Collection of Tomas Melichar	*Rethera komarovi*		Armenia
SPTMC284-12	BC-Mel2285	658[0n]	KJ168158	-		Research Collection of Tomas Melichar	*Rethera komarovi*		Iran
SPTMC283-12	BC-Mel2284	617[0n]	KJ168451	-		Research Collection of Tomas Melichar	*Rethera komarovi*		Iran
SPTMC290-12	BC-Mel2291	552[0n]	KJ168418	-		Research Collection of Tomas Melichar	*Rethera komarovi*		Turkey, Karasu
SPTMC289-12	BC-Mel2290	658[0n]	KJ168517	-		Research Collection of Tomas Melichar	*Rethera komarovi*		Turkey, Karasu
SOWD528-06	BC-Hax3427	608[0n]	KJ168513	-		Research Collection of Jean Haxaire	*Rethera komarovi*		Turkey, Malatya
SOWD529-06	BC-Hax3428	654[0n]	KJ168241	-		Research Collection of Jean Haxaire	*Rethera komarovi*		Turkey, Malatya
SPTMB235-10	BC-Mel1237	658[0n]	HQ977211	-		Research Collection of Tomas Melichar	*Theretra insignis*		Indonesia, Maluku
SPTMB234-10	BC-Mel1236	658[0n]	HQ977210	-		Research Collection of Tomas Melichar	*Theretra insignis*		Indonesia, Maluku
SPTMB237-10	BC-Mel1239	658[0n]	HQ977213	-		Research Collection of Tomas Melichar	*Theretra insignis*		Indonesia, Nusa Tenggara Barat
SPTMB238-10	BC-Mel1240	658[0n]	HQ977214	-		Research Collection of Tomas Melichar	*Theretra insignis*		Indonesia, Nusa Tenggara Barat
SPTMB233-10	BC-Mel1235	658[0n]	HQ977209	-		Research Collection of Tomas Melichar	*Theretra insignis*		Indonesia, Nusa Tenggara Timur
SPTMB232-10	BC-Mel1234	658[0n]	HQ977208	-		Research Collection of Tomas Melichar	*Theretra insignis*		Indonesia, Nusa Tenggara Timur
SPTMB236-10	BC-Mel1238	658[0n]	HQ977212	-		Research Collection of Tomas Melichar	*Theretra insignis*		Indonesia, Nusa Tenggara Timur
SPTMA240-07	BC-Mel 0318	658[0n]	JN678619	601[0n]	KJ168596	Research Collection of Tomas Melichar	*Theretra muricolor*		Papua New Guinea, West New Britain
