# Supplementary material for: Australian Sphingidae – DNA Barcodes Challenge Current Species Boundaries and Distributions
Source: PLoS One. 2014 Jul 2;9(7):e101108. doi: 10.1371/journal.pone.0101108 (PMC4079597; doi:10.1371/journal.pone.0101108)
Supplement: Table S4 — List of endemic species and subspecies of Sphingidae in Australia. (PDF) [file pone.0101108.s011.pdf]

Table S4. List of endemic species and subspecies of Sphingidae in Australia.

Endemic species

Macroglossinae

Acosmeryx near *anceus* (see Fig. 3)
Acosmeryx miskini (Murray, 1873)
Cephonodes kingii (W.S. Macleay, 1826)
Cizara ardeniae (Lewin, 1805)
Gnathothlibus australiensis Lachlan, 2004
Hyles livornicoides (Lucas, 1892)
Macroglossum joannisi Rothschild & Jordan, 1903
Macroglossum troglodytus Boisduval, [1875]
Theretra inornata (Walker, 1865)
Theretra margarita (Kirby, 1877)
Theretra queenslandi (Lucas, 1891)
Theretra turneri (Lucas, 1891)
Zacria vojtechi Haxaire & Melichar 2003

Smerinthinae

Coequosa australasiae (Donovan, 1805)
Coequosa triangularis (Donovan, 1805)
Imber tropicus Moulds, 1983

Sphinginae

Agrius godarti (W.S. Macleay, 1826)
Coenotes eremophilae (Lucas, 1891)
Coenotes arida Moulds & Melichar, [2014]
Hopliocnema brachycera (Lower, 1897)
Hopliocnema lacunosa Tuttle, Moulds & Lane, 2012
Hopliocnema ochra Tuttle, Moulds & Lane, 2012
Leucomonia bethia (Kirby, 1877)
Psilogramma argos Moulds & Lane, 1999
Psilogramma casuarinae (Walker, 1856)
Psilogramma exigua Brechlin, Lane & Kitching, 2010
Psilogramma maxmouldsi Eitschberger, 2001
Psilogramma penumbra Lane, Moulds & Tuttle, 2011
Synoecha marmorata (Lucas, 1891)
Tetrachroa edwardsi (Olliff, 1890)

Endemic subspecies

Macroglossinae

Cephonodes hylas australis Kitching & Cadiou, 2000
Cephonodes janus janus Miskin, 1891
Macroglossum divergens queenslandi Clark, 1927
Theretra indistincta indistincta (Butler, 1877)
