# Supplementary material for: Australian Sphingidae – DNA Barcodes Challenge Current Species Boundaries and Distributions
Source: PLoS One. 2014 Jul 2;9(7):e101108. doi: 10.1371/journal.pone.0101108 (PMC4079597; doi:10.1371/journal.pone.0101108)
Supplement: Table S5 — DNA barcode matches at subspecies and species levels. (PDF) [file pone.0101108.s012.pdf]

Table S5. DNA barcode matches at subspecies and species level for Australian Sphingidae: minimum genetic distances (K2P) to the nearest hetero-subspecific record (Δssp to NN.ssp) and to the nearest hetero-specific record (Δsp to NN.sp); exact matches at subspecies level and Δ distances below 2% with non-Australian species are highlighted in white/black cells. The last two columns list the relevant taxa included, and the unsampled subspecies. PT=paratype, HT=holotype.

#	Species	Δ_{ssp}	NN.ssp[*]	Δ_{sp}	NN.sp[*]	Relevant taxa sampled	Unsampled subspecies
1	*Acosmeryx anceus*	2.23	*A. a. subdentata*	3.98	*A. omissa*	*A. anceus subdentata*	-
2	*Acosmeryx miskini*	N/A	N/A	3.21	*A. miskinoides*	*A. miskinoides (PT)*	-
3	*Angonyx papuana*	N/A	N/A	4.68	*A. meeki*	*A. meeki, A. testacea*	ssp. *bismarcki*
4	*Cephonodes hylas australis*	2.41	*C. h. virescens*	3.82	*C. janus*	ssp. *hylas, melanogaster (PT), virescens*	-
5	*Cephonodes janus*	N/A	N/A	3.37	*C. xanthus*	-	ssp. *austrosundanus, simplex*
6	*Cephonodes kingii*	N/A	N/A	4.59	*Hemaris aksana*	-	-
7	*Cephonodes picus*	N/A	N/A	2.26	*C. xanthus*	-	-
8	*Cizara ardeniae*	N/A	N/A	6.79	*Rethera komarovi*	*C. sculpta, Rethera komarovi*	-
9	*Daphnis dohertyi*	N/A	N/A	4.33	*D. protrudens*	-	ssp. *callusia*
10	*Daphnis moorei*	N/A	N/A	3.62	*D. hypothous*	-	-
11	*Daphnis placida*	1.84	*D. p. salomonis*	3.98	*D. protrudens*	ssp. *salomonis*	-
12	*Daphnis protrudens*	1.07	*D. p. lecourti*	3.53	*D. torenia*	ssp. *lecourti*	-
13	*Eupanacra splendens*	5.96	*E. s. paradoxa*	4.13	*E. busiris*	ssp. *paradoxa*	-
14	*Gnathothlibus australiensis*	N/A	N/A	3.48	*G. eras*	-	-
15	*Gnathothlibus eras*	N/A	N/A	0.16	*G. vanuatuensis*	*G. saccoi, G. vanuatuensis*	-
16	*Hippotion brennus*	N/A	N/A	0.33	*H. joiceyi*	*H. joiceyi*	-
17	*Hippotion celerio*	N/A	N/A	2.3	*H. aporodes*	-	-
18	*Hippotion rosetta*	N/A	N/A	0	*H. boerhaviae*	*H. boerhaviae*	-
19	*Hippotion scrofa*	N/A	N/A	3.37	*H. brunnea*	-	-
20	*Hippotion velox*	N/A	N/A	2.14	*H. aporodes*	-	-
21	*Hyles livornicoides*	N/A	N/A	2.21	*H. zygophylli*	-	-
22	*Macroglossum alcedo*	N/A	N/A	4.23	*M. troglodytus papuanum*	*M. t. papuanum*	-
23	*Macroglossum corythus*	0.41	*M. c. pylene*	2.85	*M. semifasciata*	ssp. *fuscicauda, fulvicaudata, luteata, pylene*	ssp. *novebudensis, novirlandum, platyxanthum, xanthurus*
24	*Macroglossum dohertyi doddi*	0.46	*M. d. dohertyi*	3.89	*M. hirundo*	*M. d. dohertyi*	-
25	*Macroglossum divergens queenslandi*	-	-	-	-	*M. d. divergens, M. d. heliophila*	-

#	Species	Δssp	NN.ssp*	Δsp	NN.sp*	Relevant taxa sampled	Unsampled subspecies
26	*Macroglossum hirundo errans*	2.14	*M. h. hirundo*	1.19	*M. rectans*	*M. h. lifuensis, M. h. hirundo*	-
27	*Macroglossum joannisi*	N/A	N/A	4.81	*M. divergens heliophila*	-	-
28	*Macroglossum micacea*	N/A	N/A	5.95	*M. obscura*	-	ssp. *albibase*
29	*Macroglossum nubilum*	N/A	N/A	3.5	*M. prometheus lineata*	-	-
30	*Macroglossum prometheus lineata*	2.2	*M. p. prometheus*	2.91	*M. paukstadtorum*	ssp. *prometheus*	-
31	*Macroglossum rectans*	N/A	N/A	1.18	*M. hirundo errans*	-	-
32	*Macroglossum tenebrosa*	N/A	N/A	2.81	*M. corythus*	-	-
33	*Macroglossum troglodytus*	3.66	*M. t. papuanum*	2.92	*M. insipida*	ssp. *papuanum*	-
34	*Macroglossum vacillans*	N/A	N/A	3.13	*M. belis*	-	-
35	*Nephele hespera*	N/A	N/A	2.43	*N. joiceyi*	-	-
36	*Nephele subvaria*	N/A	N/A	2.14	*N. monostigma*	-	-
37	*Pseudoangonyx excellens*	N/A	N/A	5.97	*Macroglossum faro cottoni*	-	-
38	*Theretra celata*	1.23	*T. c. babarensis*	2.55	*T. rhesus*	ssp. *babarensis*	-
39	*Theretra indistincta*	0	*T. i. papuensis, T. i. manuselensis*	3.18	*T. insularis*	ssp. *papuensis, manuselensis*	ssp. *bismarcki*
40	*Theretra inornata*	N/A	N/A	3.37	*T. clotho*	-	-
41	*Theretra latreillii*	3.32	*T. l. lucasii*	4.59	*T. sugii*	ssp. *lucasii, prattorum*	-
42	*Theretra margarita*	N/A	N/A	4.41	*Xylophanes tyndarus*	-	-
43	*Theretra nessus*	0.56	*T. n. albata*	5.7	*Xylophanes turbata*	ssp. *albata*	-
44	*Theretra oldenlandiae lewini*	0.76	*T. o. oldenlandiae*	0	*T. insignis*	ssp. *fuscata, oldenlandiae & T. insignis*	ssp. *samoana*
45	*Theretra queenslandi*	N/A	N/A	0	*T. radiosa*	*T. muricolor*	-
46	*Theretra radiosa*	N/A	N/A	-	-	-	-
47	*Theretra silhetensis intersecta*	2.62	*T. s. silhetensis*	4.62	*Centroctena rutherfordi*	ssp. *silhetensis*	-
48	*Theretra tryoni*	N/A	N/A	3.1	*T. rhesus*	-	-
49	*Theretra turneri*	N/A	N/A	2.89	*T. insignis kuehni*	-	-
50	*Zacria vojtechi*	N/A	N/A	5.62	*Proserpinus proserpina*	-	-
51	*Ambulyx dohertyi*	2.45	*A. d. novobritannica*	5.2	*A. moorei*	ssp. *novoirlandensis (HT), novobritannica (HT), dohertyi, salomonis*	-
52	*Ambulyx wildei*	N/A	N/A	0	*A. rudloffi, A. ceramensis*	*A. rudloffi (HT), A. ceramensis*	-
53	*Coequosa australasiae*	N/A	N/A	5.74	*Pseudopolyptychus foliaceus*	-	-
54	*Coequosa triangularis*	N/A	N/A	7.48	*C. australasiae*	-	-

#	Species	Δ_{ssp}	NN.ssp*	Δ_{sp}	NN.sp*	Relevant taxa sampled	Unsampled subspecies
55	*Imber tropicus*	N/A	N/A	8.19	*Adhemarius gannascus*	-	-
56	*Agrius convolvuli*	N/A	N/A	4.98	*A. godarti*	-	-
57	*Agrius godarti*	N/A	N/A	4.98	*A. convolvuli*	-	-
58	*Cerberonoton rubescens severina*	5.5	*M. r. thielei*	5.35	*M. loeffleri*	ssp. *rubescens, thielei, titan, amboinicus*	ssp. *philippinensis*
59	*Coenotes eremophilae*	N/A	N/A	0.31	*C. jakli*	*C. jakli (PT)*	-
60	*Hopliocnema brachycera*	N/A	N/A	3.05	*H. lacunosa*	-	-
61	*Hopliocnema lacunosa*	N/A	N/A	3.05	*H. brachycera*	-	-
62	*Hopliocnema ochra*	N/A	N/A	3.69	*H. lacunosa*	-	-
63	*Leucomonia bethia*	N/A	N/A	4.83	*Psilogramma increta*	-	-
64	*Psilogramma argos*	N/A	N/A	5.2	*P. sulawesica*	-	-
65	*Psilogramma casuarinae*	N/A	N/A	3.06	*P. lifuense*	*P. hausmanni (HT)*	-
66	*Psilogramma exigua*	N/A	N/A	3.2	*P. lifuense*	-	-
67	*Psilogramma maxmouldsi*	N/A	N/A	3.05	*P. vanuatui*	-	-
68	*Psilogramma menephron*	N/A	N/A	0	*P. anne, P. bartschereri*	*P. bartschereri (HT), P. anne (HT), P. gloriosa (HT), P. choui (HT), P. danneri (HT), P. gerstmeieri (HT), P. hainanensis (HT), P. stameri (HT), P. surholti (HT)*	-
69	*Psilogramma papuensis*	N/A	N/A	2.14	*P. manusensis*	*P. mastrigti (HT), P. koalae (HT)*	-
70	*Psilogramma penumbra*	N/A	N/A	2.25	*P. vanuatui*	-	-
71	*Synoecha marmorata*	N/A	N/A	3.36	*Coenotes eremophilae*	-	-
72	*Tetrachroa edwardsi*	N/A	N/A	5.57	*Manduca leucospila*	-	-

* These columns list taxa retrieved as closest match on BOLD (www.boldsystems.org) through its ID-engine used on October, 1st 2013. The results may change with completion of the reference library and should not be interpreted as indicative of relatedness between the species/subspecies tested and its best close match.
