# Supplementary material for: Australian Sphingidae – DNA Barcodes Challenge Current Species Boundaries and Distributions
Source: PLoS One. 2014 Jul 2;9(7):e101108. doi: 10.1371/journal.pone.0101108 (PMC4079597; doi:10.1371/journal.pone.0101108)
Supplement: Appendix S1 — Sequencing methods. (DOCX) [file pone.0101108.s013.docx]

**Appendix S1 : Sequencing methods**

*Generation of DNA Barcodes*

DNA was extracted from a single leg of each specimen using an automated silica-based 96-well extraction protocol [[1](#_ENREF_1)]. Extracts were re-suspended in 40 μL of dH_2_O and a 658bp region of COI near the 5’ end of the COI gene, aka the standard DNA barcode region for animals, was amplified with the primer pair LepF1/LepR1 [[2](#_ENREF_2)]. If no product was generated, samples were amplified with LepF1/EnhLepR1 [[3](#_ENREF_3)], which targets a 609bp fragment of COI. Failures with this primer set were then amplified with two primer pairs (LepF1/MLepR1 and MLepF1/LepR1) that target partially overlapping DNA fragments of 307bp and 407bp respectively. Finally, in a very few cases of taxa lacking barcode coverage despite these three trials, six short overlapping fragments (100 to 150bp long) were targeted for amplification [[4](#_ENREF_4)]. PCR reactions were carried out in 96-well plates in 12.5 μL reaction volumes containing: 2.5 mM MgCl_2_, 5 pmol of each primer, 20 μM dNTPs, 10 mM Tris-HCl (pH 8.3), 50 mM KCl, 10-20 ng (1-2 μL) of genomic DNA, and 1 unit of high-fidelity Platinum TaqDNA polymerase (Invitrogen, Carlsbad, CA) using a thermocycling profile of 1 cycle of 1 min at 94°C, 5 cycles of 40 sec at 94°C, 40 sec at 45°C, and 1 min at 72°C, followed by 35 cycles of 40 sec at 94°C, 40 sec at 51°C, and 1 min at 72°C, with a final step of 5 min at 72°C. PCR products were checked on a 2% Agarose E-Gel® 96 (Invitrogen). Unpurified PCR fragments were sequenced in both directions (except for PCR products generated with MLep primer pairs) using BigDye version 3.1 on an ABI 3730XL DNA Analyser (Applied Biosystems, Foster City, CA). Contigs were assembled using Sequencher 4.5 (Gene Code Corporation, Ann Arbor, MI), and were subsequently aligned using Bioedit version 7.0.5.3 [[5](#_ENREF_5)]. Sequence records were checked to determine whether any derived from nuclear copies of mitochondrial DNA (NUMTs) by screening for stop codons, frame-shift mutations, heterozygosity or unusual amino-acid substitutions, especially in cases of high intraspecific divergence.

*Additional Marker*

In four species where high intraspecific variation suggested the possibility of overlooked species, the D2 expansion segment of the ribosomal large subunit (28S) was amplified using the primer pair D2B and D3Ar [[6](#_ENREF_6)]. This gene region is about 600bp long; it has a relatively fast substitution rate and has proven efficacious for the investigation of cryptic diversity in invertebrates, including Lepidoptera [[7](#_ENREF_7), [8](#_ENREF_8)]. The PCR cocktail was similar to that used for COI, but a different thermocycling regime was employed: 1 cycle of 2 min at 94°C, 35 cycles of 30 sec at 94°C, 30 sec at 56°C, and 2 min at 72°C, with a final step of 2 min at 72°C.

**References**

1. Ivanova NV, deWaard JR and Hebert PDN (2006) An inexpensive, automation-friendly protocol for recovering high-quality DNA. Mol Ecol Notes 6: 998-1002.

2. Hebert PDN, Penton EH, Burns JM, Janzen DH and Hallwachs W (2004) Ten species in one: DNA barcoding reveals cryptic species in the neotropical skipper butterfly *Astraptes fulgerator*. Proceedings of the National Academy of Sciences USA 101: 14812-14817.

3. Hajibabaei M, Janzen DH, Burns JM, Hallwachs W and Hebert PDN (2006) DNA barcodes distinguish species of tropical Lepidoptera. Proceedings of the National Academy of Sciences USA 103: 968-971.

4. Lees DC, Rougerie R, Zeller-Lukashort C and Kristensen NP (2010) DNA mini-barcodes in taxonomic assignment: a morphologically unique new homoneurous moth clade from the Indian Himalayas described in *Micropterix* (Lepidoptera, Micropterigidae). Zoologica Scripta 39: 642-661.

5. Hall TA (1999) BioEdit: a user-friendly biological sequence alignment editor and analysis program for Windows 95/98/NT. Nucleic Acids Symposium Series 41: 95-98.

6. Saux C, Fisher BL and Spicer GS (2004) *Dracula* ant phylogeny as inferred by nuclear 28S rDNA sequences and implications for ant systematics (Hymenoptera: Formicidae: Amblyoponinae). Molecular Phylogenetics and Evolution 33: 457-468.

7. Rougerie R, Naumann S and Nässig WA (2012) Morphology and molecules reveal unexpected cryptic diversity in the enigmatic genus *Sinobirma* Bryk, 1944 (Lepidoptera: Saturniidae). PloS one 7: e43920.

8. Smith MA, Wood DM, Janzen DH, Hallwachs W and Hebert PDN (2007) DNA barcodes affirm that 16 species of apparently generalist tropical parasitoid flies (Diptera, Tachinidae) are not all generalists. Proceedings of the National Academy of Sciences USA 104: 4967-4972.
